# HebbPlot: an intelligent tool for learning and visualizing chromatin mark signatures

**DOI:** 10.1186/s12859-018-2312-1

**Published:** 2018-09-03

**Authors:** Hani Z. Girgis, Alfredo Velasco, Zachary E. Reyes

**Affiliations:** Tandy School of Computer Science, University of Tulsa, 800 South Tucker Drive, Tulsa, 74104-9700 OK USA

**Keywords:** Histone marks, Chromatin modifications, Epigenetic signatures, Visualization, Artificial neural networks, Hebbian learning, Associative learning

## Abstract

**Background:**

Histone modifications play important roles in gene regulation, heredity, imprinting, and many human diseases. The histone code is complex and consists of more than 100 marks. Therefore, biologists need computational tools to characterize general signatures representing the distributions of tens of chromatin marks around thousands of regions.

**Results:**

To this end, we developed a software tool, HebbPlot, which utilizes a Hebbian neural network in learning a general chromatin signature from regions with a common function. Hebbian networks can learn the associations between tens of marks and thousands of regions. HebbPlot presents a signature as a digital image, which can be easily interpreted. Moreover, signatures produced by HebbPlot can be compared quantitatively. We validated HebbPlot in six case studies. The results of these case studies are novel or validating results already reported in the literature, indicating the accuracy of HebbPlot. Our results indicate that promoters have a directional chromatin signature; several marks tend to stretch downstream or upstream. H3K4me3 and H3K79me2 have clear directional distributions around active promoters. In addition, the signatures of high- and low-CpG promoters are different; H3K4me3, H3K9ac, and H3K27ac are the most different marks. When we studied the signatures of enhancers active in eight tissues, we observed that these signatures are similar, but not identical. Further, we identified some histone modifications — H3K36me3, H3K79me1, H3K79me2, and H4K8ac — that are associated with coding regions of active genes. Other marks — H4K12ac, H3K14ac, H3K27me3, and H2AK5ac — were found to be weakly associated with coding regions of inactive genes.

**Conclusions:**

This study resulted in a novel software tool, HebbPlot, for learning and visualizing the chromatin signature of a genetic element. Using HebbPlot, we produced a visual catalog of the signatures of multiple genetic elements in 57 cell types available through the Roadmap Epigenomics Project. Furthermore, we made a progress toward a functional catalog consisting of 22 histone marks. In sum, HebbPlot is applicable to a wide array of studies, facilitating the deciphering of the histone code.

**Electronic supplementary material:**

The online version of this article (10.1186/s12859-018-2312-1) contains supplementary material, which is available to authorized users.

## Background

Understanding the effects of histone modifications will provide answers to important questions in biology and will help with finding cures to several diseases including cancer. Carey highlights several functions of epigenetic factors including Cytosine methylation and histone modifications [1]. It was reported that methylation of CpG islands inhibit transcription [2], whereas the complex histone code has a wide range of regulatory functions [3, 4]. Additionally, epigenetic marks may affect body weight and metabolism [5]. Interestingly, chromatin marks may explain how some traits acquired due to exposure to some toxins and obesity are passed from one generation to the next (Lamarckian inheritance) [6–9]. Further, epigenetics may explain how two identical twins have different disease susceptibilities [10]. Epigenetic factors play a role in imprinting, in which a chromosome, or a part of it, carries a maternal or a paternal mark(s) [11, 12]. Defects in the imprinting process may lead to several disorders [13–18], and may increase the “birth defects” rate of assisted reproduction [19]. Furthermore, chromatin marks play a role in cell differentiation by selectively activating and deactivating certain genes [20, 21]. Some chromatin marks take part in deactivating one of the X chromosomes [22]. It has been observed in multiple types of cancer that some tumor suppressor genes were deactivated by hypermethylating their promoters [23–25], the removal of activating chromatin marks [26, 27], or adding repressive chromatin marks [28]. Utilizing such knowledge, anti-cancer drugs that target the epigenome [29–31] have been designed.

Pioneering computational and statistical methods for deciphering the histone code have been developed. Some tools are designed for profiling and visualizing the distribution of a chromatin mark(s) around multiple regions [32, 33]. Additionally, a tool for clustering and visualizing genomic regions based on their chromatin marks has been developed [34]. Several systems are available for characterizing histone codes/states in an epigenome [35–43]. Further, an alphabet system for histone codes was proposed [44]. Other tools can recognize and classify the chromatin signature associated with a specific genetic element [45–55]. Furthermore, methods that compare the chromatin signature of healthy and sick individuals are currently available [56].

Scientists have identified about 100 histone marks [37]. Additionally, there will be a large number of future studies, in which scientists need to characterize the pattern of chromatin marks around a set of regions in the genome. Therefore, scientists need an automated framework to (i) automatically characterize the chromatin signature of a set of sequences that have a common function, e.g. coding regions, promoters, or enhancers; and (ii) visualize the identified signature in a simple intuitive form. To meet these needs, we designed and developed a software tool called HebbPlot. This tool allows average users, without extensive computational knowledge, to characterize and visualize the chromatin signature associated with a genetic element automatically.

HebbPlot includes the following four innovative approaches in an area that has become the frontier of medicine and biology:

- HebbPlot can learn the chromatin signature of a set of regions automatically. Sequences that have the same function in a specific cell type are expected to have similar marks. The learned signature represents these marks around all of the regions. *HebbPlot differs from the other tools in its ability to learn one signature representing the distributions of all available chromatin marks around thousands of regions.*
- This is the first application of Hebbian neural networks in the epigenetics field. These networks are capable of learning associations; therefore, they are well suited for learning the associations among tens of marks and genetic elements.
- The framework enables average users to train artificial neural networks *automatically*. Users are not burdened with the training process. Self-trained systems for analyzing protein structures and sequence data have been proposed [57–61]. HebbPlot is the analogous system for analyzing chromatin marks.
- HebbPlot is the first system that integrates the tasks of learning and visualizing a chromatin signature. Once the signature is learned, the marks are clustered and displayed as a digitized image. This image shows one pattern representing thousands of regions. The distributions of the marks appear around one region; however, they are learned from all regions.

We have applied our tool to learning and visualizing the chromatin signatures of several active and inactive genetic elements in 57 tissues/cell types. These case studies demonstrate the applicability of HebbPlot to many interesting problems in molecular biology, facilitating the deciphering of the histone code.

## Implementation

In this section, we describe the computational principles of our software tool, HebbPlot. The core of the tool is an unsupervised neural network, which relies on Hebbian learning rules.

### Region representation

**Fig. 1:**
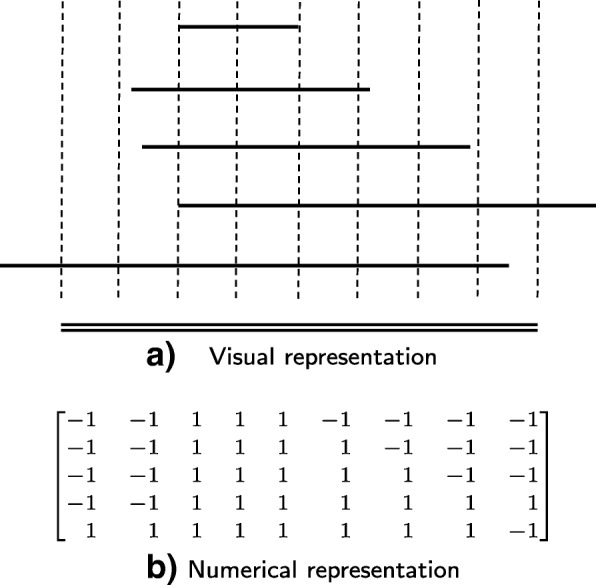
Representations of a group of chromatin marks overlapping a region. **a** Horizontal double lines represent a region of interest. Horizontal single lines represent the marks. Vertical lines are spaced equally and bounded by the region. **b** The intersections between the marks and the vertical lines are encoded as a matrix where rows represent the marks and columns represent the vertical lines. If a vertical line intersects a mark, the corresponding entry in the matrix is 1, otherwise it is -1

To represent a group of histone marks overlapping a region, these marks are arranged according to their genomic locations on top of each other and the region. Then equally-spaced vertical lines are superimposed on the stack of the marks and the region. The numerical representation of this group of marks is a matrix. A row of the matrix represents a mark. A column of the matrix represents a vertical line. If the *i*^*th*^ mark intersects the *j*^*th*^ vertical line, the entry *i* and *j* in the matrix is 1, otherwise it is -1. Figure 1 shows the graphical and the numerical representations of a region and the overlapping marks. Finally, the two-dimensional matrix is converted to a one dimensional vector called the epigenetic vector. The number of vertical lines is determined experimentally — 41 and 91 lines were used in our case studies. This number should be adjusted according to the average size of a region. One may think of this number as the resolution level, the more the vertical lines, the higher the resolution.

### The dotsim function

The dot product of two vectors indicates how close they are to each other in space. When these vectors are normalized, i.e. each element is divided by the vector norm, the dot product is between 1 and -1. The dotsim function (Eq. 1) normalizes the vectors and calculates their dot product.

1 $$  dotsim(x,y) = \frac{x}{ \|x\|} \cdot \frac{y}{ \|y\| }  $$

Here, x and y are vectors; ∥*x*∥ and ∥*y*∥ are the norms of these vectors; the · symbol is the dot product operator. If the two vectors are very similar to each other, the dotsim value approaches 1. If the values at the same index of the two vectors are opposite of each other, i.e. 1 and -1, the value of dotsim approaches -1.

### Data preprocessing

Preprocessing input data is a standard procedure in machine learning. During this procedure, the noise in the input data is reduced. First, vectors that consist mainly of -1’s are removed — a dotsim value of at least 0.8 with the negative-ones vector. These regions are very likely false positives. Then, each epigenetics vector is compared to two other vectors selected randomly from the same set. The value of an entry in the vector is kept if it is the same in the three vectors, otherwise it is set to zero. For example, consider the vector [1 1 -1]. Suppose that the vectors [1 -1 -1] and [1 -1 -1] were selected randomly. The result would be [1 0 -1] because the first and the third elements are the same in the three vectors, but the second element is not.

### Hebb’s network

Associative learning, also known as Hebbian learning, is inspired by biology. “When an axon of cell A is near enough to excite a cell B and repeatedly or persistently takes part in firing it, some growth process or metabolic change takes place in one or both cells such that A’s efficiency, as one of the cells firing B, is increased” [62]. Hebb’s artificial neural networks aims at associating two stimuli: unconditioned and conditioned. After training, the response to either the conditioned stimulus or the unconditioned one is the same as the response to both stimuli combined [63]. In the context of epigenetics, the unconditioned stimulus, *b*, is a one-dimensional vector representing the distributions of histone marks over a sequence e.g. one tissue-specific enhancer. This vector is referred to as the epigenetic vector; it is obtained as outlined earlier in this section. The conditioned stimulus is always the one vector, which include ones in all entries. We would like to train the network to give a response when it is given the ones vector, whether or not the epigenetic vector is provided. The response of the network is a prototype/signature representing the distributions of histone marks over the entire set of genomic locations, e.g. all enhancers of a specific tissue.

Equations 2 and 3 define how the response of a Hebbian network is calculated. The training of the network is given by Eq. 4 [63].

2 $$  satlins(x) = \left\{ \begin{array}{lll} +1 & \quad \text{if}\ x \geq 1 \\ x & \quad \text{if}\ -1 < x < 1 \\ -1 & \quad \text{if}\ x \leq -1 \\ \end{array} \right.  $$

Equation 2 defines a transformation function. This function ensures that the response of the network is similar to the unconditioned stimulus, i.e. each element of the response is between 1 and -1. If x is a vector, the function is applied component wise.

3 $$  a(b, w, p) = satlins(b + w \odot p)  $$

**Fig. 2:**
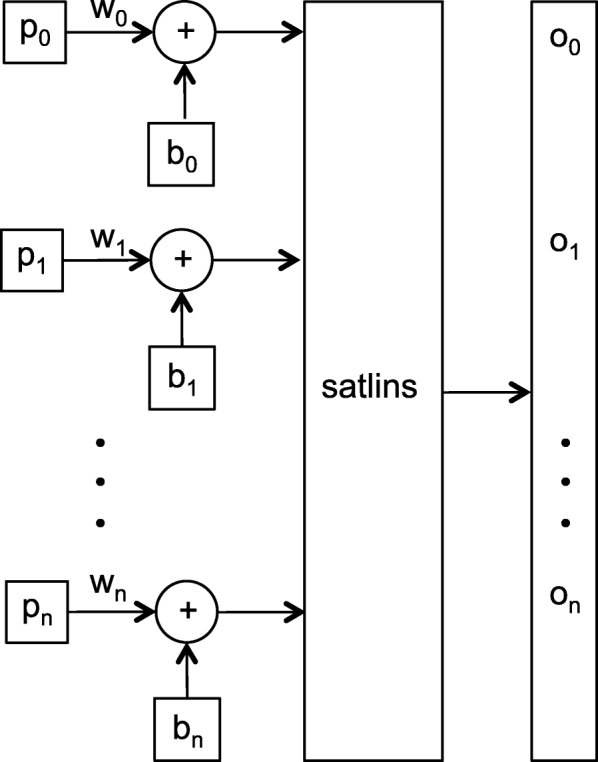
Unsupervised Hebb’s network: *w* is the weight vector, which represents the learned signature; *b* is an epigentic vector; *p* is the ones vector; satlins is the activation/transformation function (Eq. 2); *o* is the output of the network; and *n* is the size of *p*, *b*, *w*, and *o*

Equation 3 describes how a Hebbian network responds to the two stimuli (Fig. 2). The response of the network is transformed using Eq. 2. In Eq. 3, *b* is the unconditioned stimulus, e.g. an epigenetic vector; *w* is the weights vector, which is the prototype/signature learned so far; and *p* is the conditioned stimulus, e.g. the one vector. The operator ⊙ represents the component wise multiplication of two vectors. In the current adaptation, if the network is presented with an epigenetic vector and the one vector, the response is the sum of the prototype learned so far and the epigenetic vector. In the absence of the epigenetic vector, i.e. all-zeros *b*, the response of the network is the prototype, demonstrating the ability of the network to learn associations.

4 $$  w^{i} = w^{i-1} + \alpha \left(a\left(b^{i}, w^{i-1}, p^{i}\right) - w^{i-1}\right) \odot p^{i}  $$

Equation 4 defines Hebb’s unsupervised learning rule. Here, *w*^*i*^ and *w*^*i*−1^ are the prototype vectors learned in iterations *i* and *i*−1. The *i*^*th*^ pair of unconditioned and conditioned stimuli is *b*^*i*^ and *p*^*i*^. Learning occurs, i.e. the prototype changes, only when the *i*^*th*^ conditioned stimulus, *p*^*i*^, has non-zero components. This is the case here because *p*^*i*^ is always the ones vector. Due to a small *α*, which represents the learning and the decay rates, the prototype vector changes a little bit in each iteration when learning occurs; it moves closer to the response of the network to the *i*^*th*^ pair of stimuli.

### Comparing two signatures

One of the main advantages of the proposed method is that two signatures can be compared quantitatively. The dotsim function can be applied to the whole epigenetic vector or to the part representing a specific mark. When comparing the chromatin signatures of two sets of regions, a mark with a dotsim value approaching 1 is common in the two signatures. A mark with a dotsim value approaching -1 has opposite distributions, distinguishing the signatures. Marks with dotsim values approaching zero do not have consistent distribution(s) in one or both sets; these marks should not be considered while comparing the two signatures.

### Visualizing a chromatin signature

Row vectors representing different marks are clustered according to their similarity to each other. We used hierarchical clustering in grouping marks with similar distributions. The applied hierarchical clustering algorithm is an iterative bottom-up approach, in which the closest two items/groups are merged at each iteration. The algorithm requires a pair wise distance function and a cluster wise distance function. For the pair wise distance function, we utilized the city block function to determine the distance between two vectors representing marks. For the group wise distance function, we applied the weighted pair group method with arithmetic mean [64]. A digitized image represents the chromatin signature of a genetic element. A one-unit-by-one-unit square in the image represents an entry in the matrix representing the signature. A row of these squares represents one mark. The color of a square is a shade between red and blue if the entry value is less than 1 and greater than -1; the closer the value to 1 (-1), the closer its color to red (blue).

Up to this point, we discussed the computational principles of our software tool, HebbPlot. Next, we illustrate the data used in validating the tool.

### Data

We used HebbPlot in visualizing chromatin signatures characterizing multiple genetic elements. Specifically, we applied HebbPlot to:

- Active promoters — 400 base pairs (bp);
- Active promoters on the positive strand — 4400 bp;
- Active promoters on the negative strand — 4400 bp;
- High-CpG active promoters — 400 bp;
- Low-CpG active promoters — 400 bp;
- Active enhancers — 400 bp and variable size;
- Coding regions of active genes — variable size;
- Coding regions of inactive genes — variable size; and
- Random genomic locations — 1000 bp.

The Roadmap Epigenomics Project provides tens of marks for more than 100 tissues/cell types [65]. Active genes were determined according to gene expression levels, which were obtained from the Expression Atlas [66] and the Roadmap Epigenomics Project [67]. The coding regions were obtained from the University of California Santa Cruz Genome Browser [68]. The Ensemble genes for the hg19 human genome assembly were used in this study. A gene with expression level at least 1 is considered active, whereas inactive genes have expression levels of 0. Active promoters are those associated with active genes. A promoter region is defined as the 400-nucleotides-long region centered on the transcription start site — except in one case study, in which the promoter size was 4400 nucleotides. To divide the promoters into high- and low-CpG groups, we calculated the CpG content according to the method described by Saxonov et al. [69]. Enhancers active in H1 and IMR90 were obtained from a study by Rajagopal et al. [54]; this study provides the P300 peaks. We considered the enhancers to be the 400-nucleotides-long regions centered on the P300 peaks. Regions of enhancers active in liver, foetal brain, foetal small intestine, left ventricle, lung, and pancreas were obtained from the Fantom Project [70] — these have variable sizes.

Once the locations of a genetic element were determined, they are processed further. If the number of the regions, e.g. tissue-specific enhancers, was more than 10,000 regions, we uniformly sampled 500 regions from each chromosome. Each region was expanded by 10% on each end to study how chromatin marks differ from/resemble the surrounding regions. Overlapping regions, if any, were merged. We used 41 vertical lines for all case studies except the study comparing the promoters on the positive and the negative strands (91 lines were used in that study).

In this section, we discussed the computational method and the data. Next, we apply HebbPlot in six case studies.

## Results

### Case study: signature of H1-specific enhancers

**Fig. 3:**
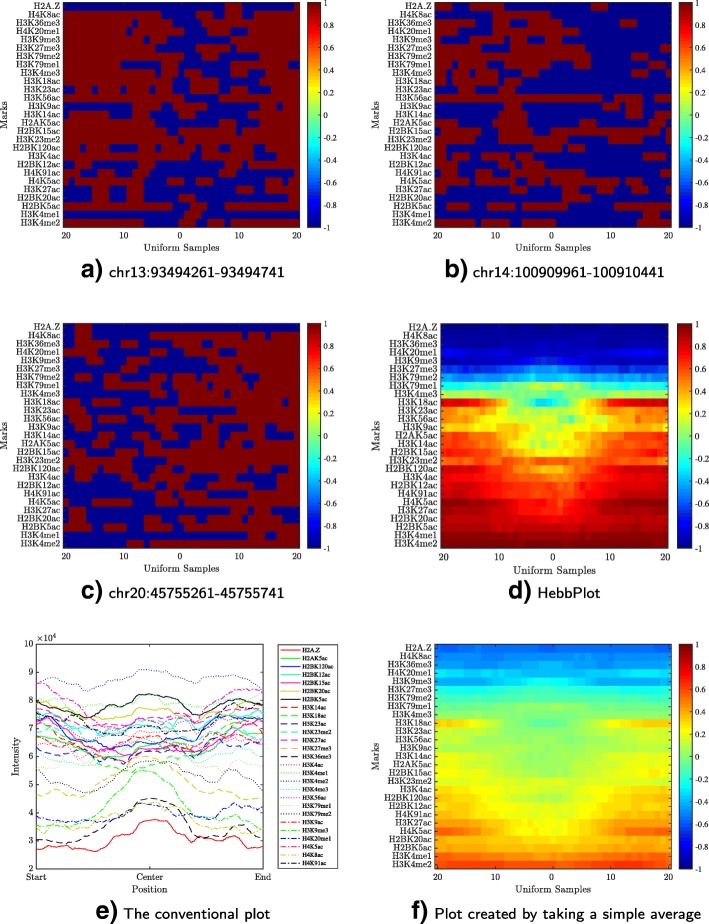
Retrieving the chromatin signature of the H1-specific enhancers. Three examples of enhancers are shown in Parts **a**–**c**. A row in one of these plots represents the distribution of one mark around a region; red (blue) color indicates the presence (absence) of a mark. It is hard to see a common pattern in these three examples. The signature learned by the Hebbian network is captured by the HebbPlot shown in Part **d**. A row in the HebbPlot represents the distribution of a mark around all enhancers in the data set. The closer the color to red, the higher the certainty of the presence of a mark around the corresponding sub-region. The HebbPlot is characterized by four zones. The top most zone represents chromatin marks that are absent from the enhancer regions, whereas the next three zones represent the present marks with increasing certainty. A conventional plot of the intensities of all marks around every region in the data set in shown in Part **e**. Many marks show depressions near the center of the plot; however, some peaks are mixed with these depressions in the conventional plot. In contrast, these depressions correspond to the ellipse in the middle of the third zone of the HebbPlot. This ellipse is very clear. Further, marks of similar intensities obstruct one another in the conventional plot. This is not the case with HebbPlot because every mark is represented by a separate row. An average plot is displayed in Part **f**. This plot shows a similar — but fuzzy — pattern to the one found by the network

**Fig. 4:**
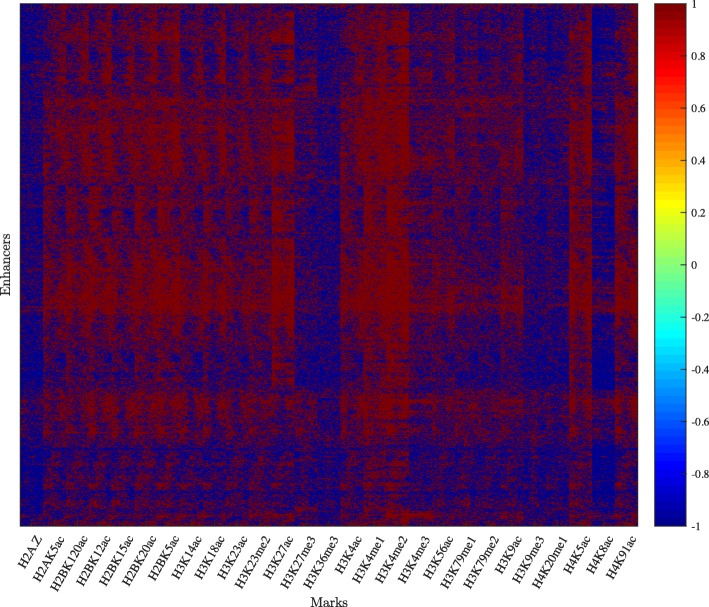
Hierarchical clustering of histone marks around 5899 H1-specific enhancers. The epigenetic vectors, except they are filled row-wise not column-wise, are clustered. This figure shows that certain marks have clear consistent pattern around these regions. However, the specific signature of these marks is not easily interpreted

We studied multiple enhancers active in the H1 cell line (human embryonic stem cells) obtained from a study conducted by Rajagopal et al. [54]. These enhancers were detected using P300 ChIP-Seq. This data set contains 5899 enhancers and 27 histone marks. To begin, we plotted tens of these enhancers; three of these plots are shown in Fig. 3a–c. No clear signature appears in these plots. After that, a HebbPlot representing the signature of H1-specific enhancers was generated (Fig. 3d) using an unsupervised hebbian network. For comparison purposes, we generated a conventional plot (Fig. 3e). To generate this plot, the middle points of all regions are aligned. Then the intensity of a mark at each nucleotide is calculated as the number of times the mark is present at this nucleotide. Figure 3f shows the average plot of the epigenetic vectors of all regions. Finally, we clustered all of the epigenetic vectors (except now the vector is filled row-wise not column-wise from the matrix) using hierarchical clustering (Fig. 4).

The HebbPlot shows four zones representing the absent marks, and the present ones with different confidence levels. For example, the top zone shows four marks (H2A.Z, H4K8ac, H3K36me3, and H4K20me1) that are absent from the H1 enhancers. The second zone from the top shows marks with very weak intensities including H3K9me3, H3K27me3, H3K79me2, and H3K79me1. The third zone has an ellipse, which is cooler — less red — than the surrounding area, implying that the signals of the marks within the ellipse are weaker than the surroundings. The bottom zone shows two marks (H3K4me1 and H3K4me2) that are present around these enhancers consistently.

In the upper part of the conventional plot, a large number of marks show depressions near the middle of the plot. However, these depressions are mixed with few peaks, making them hard to view. These depressions correspond to the fragments near the centers of the individual plots and the ellipse in the middle of the third zone of the HebbPlot. The ellipse in the third zone of the HebbPlot captures this pattern much better than the conventional plot. Further, marks with similar intensities overlap each other in the conventional plot, obstructing one another — the more the marks, the worse the obstruction. To illustrate, this figure was generated using 27 marks; there are about 100 known histone marks; therefore, using these conventional figures may not be the best way to visualize the intensities of a large number of marks. In contrast, HebbPlot can handle a large number of marks efficiently because each mark has its own row. Furthermore, no noise-removal process was applied while constructing the conventional figure. In contrast, only regions, or sub-regions, that are recognized by the network contribute to the HebbPlot.

The average plot shows similar zones to the ones shown in the HebbPlot; however, they are very fuzzy. One area of comparison is the ellipse in the third zone. In the average plot, this ellipse is spanning almost the entire zone, implying that these marks are weakly present around the 400-nucleotides-long enhancers. In contrast, the ellipse is smaller in the HebbPlot, suggesting that these marks are weakly present around the center of these enhancers, not the entire regions. The differences between the average plot and the HebbPlot are due to the network selectivity to which regions or sub-regions are utilized in learning the signature. Not all regions, or sub-regions, contribute to the learned signature. Regions and sub-regions that cause the network to fire, i.e. they are recognized by the network, contribute to the learned signatures (Eqs. 2, 3, and 4). These results suggest that HebbPlot produces more accurate and more biologically relevant results.

Hierarchical clustering has been a common method in analyzing and visualizing histone data. This method is very useful in identifying the number of signatures present in the data, but the displayed clusters, which represent the found signatures, are not easy to be interpreted. On the other hand, the current version of HebbPlot can characterize only one signature — not multiple signatures as the hierarchical clustering. However, a HebbPlot is intuitive and can be easily interpreted. These two methods can be used together when the data contains multiple signatures, which does not appear to be the situation in this case study. First, a user may use hierarchical clustering, or any clustering algorithm, to identify different clusters. Then the user can generate a HebbPlot from each cluster.

In sum, HebbPlot has advantages to plots based on the average, conventional plots, and plots based on clustering the underlying histone data.

Next, we study the signatures of enhancers, promoters, and coding regions of active genes in the liver.

### Case study: histone signatures of different active elements in liver

**Fig. 5:**
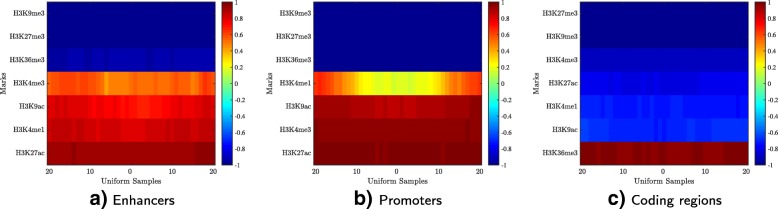
Liver chromatin signatures representing **a** active enhancers, **b** active promoters, and **c** coding regions of active genes. The three signatures have similarities and differences. They are similar in that H3K9me3 and H3K27me3 are absent from all of them. H3K36me3 is the strongest mark of coding regions, whereas H3K27ac is the strongest mark of promoters and enhancers. H3K4me1 is stronger than H3K4me3 in enhancers; this relation is reversed in promoters, where H3K4me1 is weak around transcription start sites

Seven histone marks of the human liver epigenome are available. We obtained 5005 enhancers, 13,688 promoters, and 12,484 coding regions of active genes in liver. In addition, we selected 10,000 locations sampled uniformly from all chromosomes of the human genome as controls. Then we trained four Hebbian networks to learn the chromatin signature of each genetic element. As expected, the HebbPlot representing the random genomic locations displays a deep-blue box (not shown), indicating that no chromatin mark is distributed consistently around these regions. Figure 5 shows three HebbPlots of the enhancers, the promoters, and the coding regions. The three signatures have similarities and differences. Two marks, H3K9me3 and H3K27me3, are absent from the three signatures. However, the three signatures are distinguishable. H3K36me3 is the strongest mark of the coding regions, whereas it is absent from the promoters and the enhancers. On the other hand, H3K27ac is the strongest mark on the promoters and the enhancers, but almost absent from the coding regions. H3K4me1 is stronger than H3K4me3 around the enhancers, but H3K4me3 is stronger than H3K4me1 around the promoters. Both of these marks are absent from the coding regions. These plots demonstrate that HebbPlot is able to learn the chromatin signature from a group of regions with the same function. In addition, the chromatin signatures of the promoters, the enhancers, and the coding regions have similarities and differences.

### Case study: The directional signature of active promoters

**Fig. 6:**
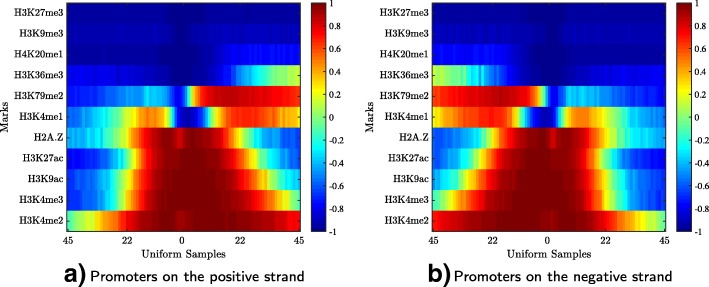
HebbPlots of active promoters in HeLa-S3 cervical carcinoma cell line. These promoters were separated into two groups according to their strands. The size of a promoter is 4400 nucloetides. The two HebbPlots of the promoters on the positive and the negative strands are mirror images of each other. Multiple marks including H3K36me3, H3K79me2, H3K4me1, H2A.Z, H3K27ac, H3K9ac, H3K4me3, and H3K4me2 are distributed in a direction specific way. H2A.Z tends to stretch upstream, whereas the rest of these directional marks tend to stretch downstream from the promoters toward their coding regions. **a** Promoters on the positive strand, **b** Promoters on the negative strand

Because promoters are upstream from their genes, some marks may indicate the direction of the transcription. To determine whether or not marks have direction, active promoters (4400 nucleotides long) were separated according to the positive and the negative strands into two groups. We trained two Hebbian networks to learn the chromatin signatures of active promoters on the positive and the negative strands. Figure 6 shows the HebbPlots of the positive and the negative promoters active in HeLa-S3 cervical carcinoma cell line. These two plots are mirror images of each other, showing H3K36me3, H3K79me2, H3K4(me1,me2,me3), H3K27ac, and H3K9ac stretching more downstream than upstream and H2A.Z in the opposite direction.

Then we generated HebbPlots for the positive (Additional file 1) and the negative (Additional file 2) promoters of 57 tissues, for which we know their gene expression levels. The directional signature of promoters is very consistent in these tissues. After that, we determined quantitatively which marks having directional preferences in the 57 tissues/cell types. To determine directional marks, the learned prototype of a mark over the upstream third of the promoter region was compared to the prototype of the same mark over the downstream third. If the dotsim value between the two prototypes is negative, this mark is considered directional. We list the results in Table 1. H3K4me3 and H3K79me2 show directional preferences in 72% and 71% of the tissues. Additional 12 marks show directional preferences in 50–70% of the tissues. These results indicate that active promoters have a directional chromatin signature.

**Table 1:** Promoters — 4400 nucleotides long — were separated according to the strand to positive and negative groups

Mark	Known	Directional	Percentage (%)
H3K4me3	57	41	72
H3K79me2	14	10	71
H3K4me2	16	11	69
H2AK5ac	6	4	67
H3K18ac	6	4	67
H2A.Z	14	9	64
H3K4me1	57	35	61
H2BK12ac	5	3	60
H3K14ac	5	3	60
H3K9ac	24	13	54
H2BK5ac	6	3	50
H3K23ac	6	3	50
H3K4ac	6	3	50
H3K79me1	6	3	50
H3K27ac	49	22	45
H4K91ac	5	2	40
H4K8ac	6	2	33
H2BK120ac	6	1	17
H4K20me1	12	2	17
H3K36me3	57	6	11

Mark vectors over the upstream and the downstream thirds of the promoters on the positive strand were compared. A mark is considered directional if these two vectors have a negative dotsim value. The number of cell types, for which a mark was determined, is listed under “Known.” The number of cell types, in which a mark has directional preference around the promoter regions, is listed under “Directional.” The percentage of times a mark showed directional preference is listed under “Percentage.” Only marks determined for at least five tissues were considered

### Case study: The signatures of high- and low-CpG promoters

**Fig. 7:**
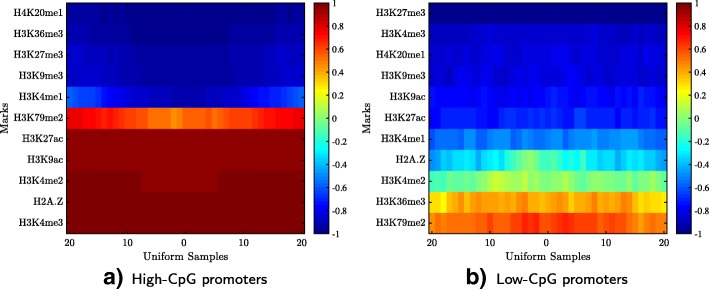
Promoters active in skeletal muscle myoblasts cells were separated into high- and low-CpG groups. A HebbPlot was generated from each group. Clearly, the two signatures are different. Specifically, H3K4me3, H3K9ac, and H3K27ac are present around the high-CpG promoters, whereas they are very weak or absent from the low-CpG promoters. In contrast, H3K36me3 is absent from the high group, but present around the low-CpG promoters. In general, marks present around the high-CpG promoters are stronger than those present around the low-CpG ones. **a** High-CpG promoters, **b** Low-CpG promoters

It has been reported in the literature that the chromatin signature of high-CpG promoters is different from the signature of low-CpG promoters [47]. In this case study, we used HebbPlot to demonstrate this phenomenon. To this end, we divided promoters active in skeletal muscle myoblasts cells into high-CpG and low-CpG groups using the method proposed by Saxonov et al. [69]. The high-CpG group consists of 12825 promoters and the low-CpG group consists of 2712 promoters. After that, we generated two HebbPlots from these two groups (Fig. 7).

The two signatures are very different. The high-CpG HebbPlot has more red bands than that of the low-CpG group, indicating that these histone marks are consistently distributed around the high-CpG promoters. Few marks distinguish the two signatures. The high-CpG group is characterized by the presence of H3K4me3, H3K9ac, and H3K27ac, which are very weak or absent from the low-CpG promoters. The low-CpG group is characterized by the presence of H3K36me3, which is absent from the high-CpG promoters. These two signatures are different from those reported by Karlic et al. [47]. Two factors may cause these differences. First, the size of the promoter region differs between the two studies. In our study, the size of the promoter is 400 base pairs, while it is defined as 3500 base pairs long (−500 to + 3000) in the other study. This longer region is likely to overlap with untranslated and coding regions, whereas it is less likely that the 400-base-pairs-long promoter to overlap with these regions. The second factor is that the other study focuses on the correlation between histone marks and expression level, whereas the main purpose of our case study is to visualize the signature of the promoters. Therefore, our definition is more relevant to the visualization task.

Next, we performed quantitative comparisons to see if these marks are distributed differently around high- and low-CpG promoters in a consistent way in the 57 tissues. A main advantage of HebbPlots is that they can be compared quantitatively. HebbPlots were generated from the high-CpG promoters (Additional file 3) and the low-CpG promoters (Additional file 4) in the 57 cell types/tissues. We calculated the average dotsim of the two vectors representing a mark around high- and low-CpG promoters in the 57 tissues. Table 2 shows the results. These results confirm that H3K4me3, H3K9ac, and H3K27ac are consistently different around high- and low-CpG promoters (average dotsim value < -0.5). However, H3K36me3 is not different overall (average dotsim value of 0.65). Further, this analysis reveals that H2BK120ac and H4K91ac are also distributed differently around the two groups (average dotsim < -0.5); their signals are stronger around the high-CpG group than the low group.

**Table 2:** High-CpG promoters have a different signature from that of low-CpG promoters

Mark	Known	Average dotsim
H3K4me3	57	-0.98452
H3K9ac	24	-0.82137
H3K27ac	49	-0.72655
H2BK120ac	6	-0.53278
H4K91ac	5	-0.48083
H3K4me2	16	-0.33263
H3K23ac	6	-0.32737
H2A.Z	14	-0.27855
H2BK12ac	5	-0.20927
H2BK5ac	6	-0.15632
H3K4ac	6	-0.15405
H4K8ac	6	-0.12716
H2AK5ac	6	-0.11522
H3K14ac	5	-0.03981
H3K18ac	6	0.14699
H3K4me1	57	0.24636
H3K79me1	6	0.35168
H3K79me2	14	0.62139
H3K36me3	57	0.65545
H4K20me1	12	0.82929
H3K27me3	57	0.92651
H3K9me3	57	0.97729

Active promoters in 57 tissues/cell types were divided into two groups according to their CpG contents. Then two networks were trained on the two groups, producing two signatures for each tissue/cell type. The two signatures of a mark in the same tissue were compared using the dotsim function. The average dotsim values are listed under “Average dotsim.” Not all marks were determined for all tissues. The number of tissues/cell types, for which a mark was determined, is listed under the column titled “Known”

In sum, the chromatin signatures of high- and low-CpG promoters are different. Five marks are present around high-CpG promoters, whereas they are absent from or very weak around low-CpG promoters.

### Case study: signature of active enhancers

Here, we demonstrate HebbPlot’s applicability to visualizing the chromatin signatures of enhancers in multiple tissues. To this end, we collected active enhancers from two sources. Enhancers active in H1 (5899 regions) and IMR90 (14073 regions) were obtained from a study by Rajagopal et al. [54]. Enhancers active in other six tissues were obtained from the Fantom Project. We selected these tissues because they were common to the Fantom and the Roadmap Epigenomics Projects. These enhancers include 5005 regions for liver, 1476 regions for foetal brain, 5991 regions for foetal small intestine, 1619 regions for left ventricle, 11003 regions for lung, and 2225 regions for pancreas.

**Fig. 8:**
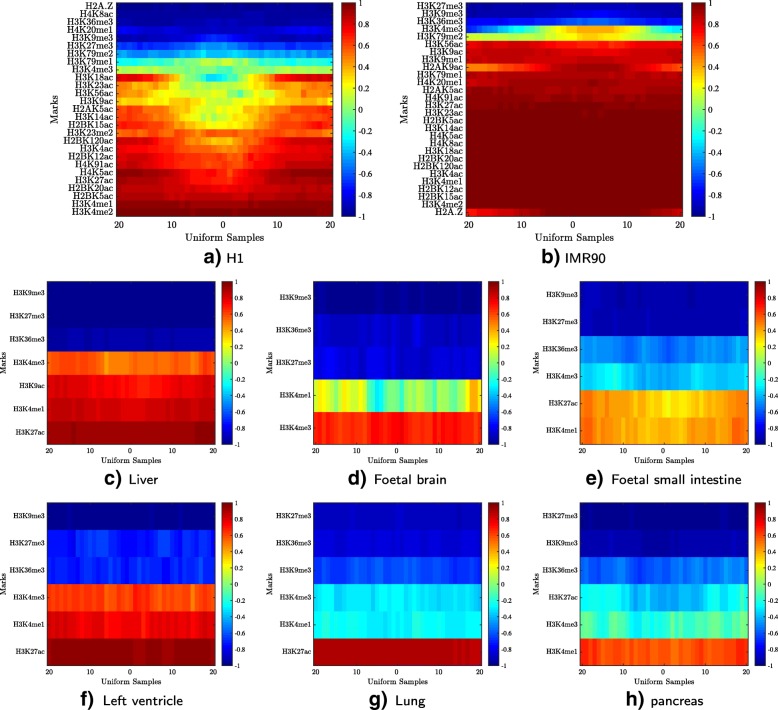
Signatures of active enhancers. Enhancers were collected from a study by Rajagopal et al. [54] and from the Fantom Project. A HebbPlot was generated from the enhancers of each tissue. The HebbPlots of H1 and IMR90, for which more than 20 marks are known, show that several marks are present around active enhancers. Usually, H3K4me1 has a stronger signal around enhancers than H3K4me3; however there are some exceptions, e.g. foetal brain. H3K9ac and H3K27ac are present around enhancers, but H3K9me3, H3K27me3, and H3K36me3 are very weak or absent from enhancers. These plots show that chromatin signatures of enhancers active in different tissues are similar, but not identical. **a** H1, **b** IMR90, **c** Liver, **d** Foetal brain, **e** Foetal small intestine, **f** Left ventricle, **g** Lung, **h** pancreas

Next, we generated a HebbPlot from the enhancers of each tissue/cell type (Additional file 5). Figure 8 show the eight HebbPlots. The HebbPlots of the enhancers active in H1 and IMR90, for which more than 20 marks have been determined, show that multiple marks are abundant around enhancer regions. Similar to what has been reported in the literature, we observed that H3K4me1 is usually stronger than H3K4me3 around enhancers [71]; however there are some exceptions, e.g. foetal brain and lung. H3K27ac and H3K9ac are also present around enhancers, but H3K9me3, H3K27me3, and H3K36me3 are very weak or absent from enhancers. Further, these HebbPlots suggest that the chromatin signatures of enhancers active in different tissues are similar; however, they are not identical. For example, H3K27ac is the predominant mark around lung enhancers; H3K4me1 and H3K4me3 are also present, but their signals are weak. In contrast, H3K27ac and H3K4me1 have comparable signals, which are stronger than H3K4me3, around enhancers of foetal small intestine.

### Case study: signatures of coding regions of active and inactive genes

**Fig. 9:**
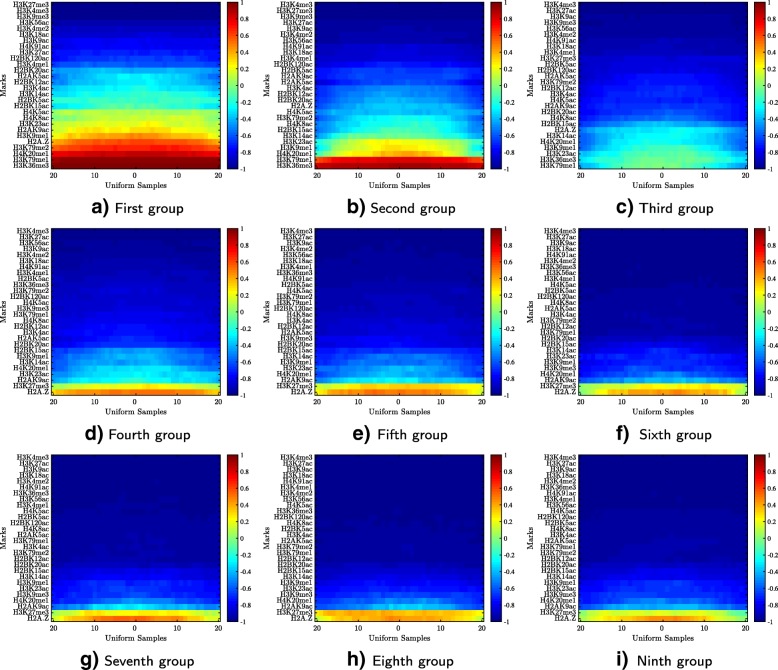
Histone marks are highly associated with gene expression levels in IMR90. Genes were divided into nine groups according to their expression levels. A HebbPlot was generated from the coding regions of each group. In general, a HebbPlot cools down — becomes bluer — as the expression level decreases. The more red a row is, the more consistent its mark is distributed around the set of regions. H3K36me3 and H3K79me1 mark the coding regions of active genes in IMR90, whereas the repressive modification, H3K27me3, marks the inactive coding regions. H2A.Z is ubiquitous. **a** First group, **b** Second group, **c** Third group, **d** Fourth group, **e** Fifth group, **f** Sixth group, **g** Seventh group, **h** Eighth group, **i** Ninth group

Multiple studies indicate that histone marks are associated with gene expression levels [52, 72, 73]. In this case study, we demonstrate the usefulness of HebbPlot in identifying histone marks associated with high and low expression levels. Genes were divided into nine groups based on their expression levels in IMR90 (Additional file 6). A HebbPlot was generated from the coding regions of each of these groups (Fig. 9). We found that H3K36me3 and H3K79me1 mark the top two groups. On the lowest six groups, which represent coding regions of inactive genes, these two marks are absent, whereas H3K27me3 is present. H2A.Z is present in all groups. Generally, the heat — demonstrated by red — of a HebbPlot decreases as the gene expression levels decrease. These results show that HebbPlot can help with identifying marks associated with coding regions of active and inactive genes.

After that, we asked whether these marks consistently mark active and inactive coding regions in other tissues/cell types. To answer this question, we generated HebbPlots of coding regions of active (Additional file 7) and inactive (Additional file 8) genes in the 57 tissues/cell types. We calculated the average dotsim values of each mark in the two signatures in the tissues/cell types, for which this mark has been determined. H3K36me3 and H3K79me1 are very different around active and inactive coding regions (average dotsim: -0.86 and -0.64). H3K27me3 is also different (average dotsim: 0.44), but the difference is not as strong as H3K36me3 and H3K79me1. After that we asked which other marks are distributed differently around coding regions of active and inactive genes. We found that H3K79me2 consistently marks active coding regions (average dotsim: -0.38). Additionally, we found H4K8ac weakly marks active coding regions (average dotsim: 0.45). Regarding the marks of inactive coding regions, H4K12ac was found to mark these regions (dotsim: -0.67) — this mark has been determined for one tissue only. H4K14ac and H2AK5ac were found to weakly mark inactive coding regions (average dotsim: 0.34 and 0.46). Generally, the active marks are stronger than the inactive marks.

### Toward a functional catalog of histone marks

Table 3 shows a summary of the findings of this study.

**Table 3:** A catalog of functions of histone marks in this study

Mark	Function	Literature support
H2A.Z	Directional around promoters stretching upstream.	Associated with trascription start sites [39] and promoters [36].
H2AK5ac	Directional around promoters stretching downstream and weakly associated with coding regions of inactive genes.	–
H2BK5ac	Directional around promoters stretching downstream.	Associated with promoters [36, 47].
H2BK12ac	Directional around promoters stretching downstream.	–
H2BK120ac	Associated with high-CpG promoters.	Associated with promoters and CpG islands [36].
H3K4ac	Directional around promoters stretching downstream.	Associated with promoters [36].
H3K4me1	Directional around promoters stretching downstream, absent around transcription start sites, and associated with enhancers.	Associated with enhancers [37, 39].
H3K4me2	Directional around promoters stretching downstream and associated with enhancers.	Associated with promoters [74] and enhancers [36].
H3K4me3	Directional around promoters stretching downstream, associated with high-CpG promoters, and associated with enhancers — usually weaker than H3K4me1.	Associated with trascription start sites [39], promoters [36, 37, 74, 75], CpG islands [36], and enhancers [36, 39].
H3K8ac	Weakly associated with coding regions of active genes.	–
H3K9ac	Directional around promoters stretching downstream, associated with high-CpG promoters, and associated with enhancers.	Associated with promoters [74] and CpG islands [36].
H3K9me3	Weakly associated with coding regions of inactive genes, and very weak/absent from enhancers, and very weak/absent from promoters.	Associate with “repressed regions” [37, 72].
H3K14ac	Directional around promoters stretching downstream and weakly associated with coding regions of inactive genes.	–
H3K18ac	Directional around promoters stretching downstream.	–
H3K23ac	Directional around promoters stretching downstream.	–
H3K27ac	Associated with high-CpG promoters and enhancers.	Associated with trascription start sites [39], promoters [36, 74], high-CpG promoters [47], CpG islands [36], and enhancers [36, 39].
H3K27me3	Weakly associated with coding regions of inactive genes, very weak/absent from enhancers, and very weak/absent from promoters.	“Repressive mark” [37, 72, 75].
H3K36me3	Associated with coding regions and very weak/absent from enhancers.	Associated with and directional around “transcriped gene bodies" [75]; associated with “transcribed regions” [37] and highly expressed genes [72].
H3K79me1	Directional around promoters stretching downstream and associated with coding regions of active genes.	Associated with promoters active in CD4+ [47] and “transcribed regions” [37].
H3K79me2	Directional around promoters stretching downstream and associated with coding regions of active genes.	Associated with “transcribed regions” [37].
H4K12ac	Associated with coding regions of inactive genes — this mark is known in one tissue only.	–
H4K91ac	Associated with high-CpG promoters.	Associated with promoters [36] and CpG islands [36].

Up to this point, we demonstrated the usefulness of HebbPlot in six case studies. Next, we discuss the similarities and the differences between HebbPlot and other visualization tools.

## Discussion

### Comparison to related tools

Visualization of chromatin marks and their associations with thousands of elements active in a specific cell type is critical to deciphering the function(s) of these marks. Extracting trends and patterns by mere inspection is essentially impossible given that there are more than 100 known chromatin marks and thousands of sequences. As such, it is vital for biologists to have visualization tools to aid in these tasks. To this end, several tools — Chromatra, ChAsE, and DGW — have been developed. In addition, we have created our own visualization technique, HebbPlot. Unlike the other three tools, which cluster genomic regions according to histone modifications, HebbPlot uses an artificial neural network to summarize the data in a form that is convenient for biologists. The following is a brief discussion about HebbPlot and its characteristics that differ from the aforementioned utilities.

Chromatra is a visualization tool that displays chromatin mark enrichment of subregions of each of the input regions. Since it is a plug-in for the well-supported Galaxy platform, it is simple for a user to add it to his or her list of tools. Additionally, this tool is comprised of two modules for chromatin mark analysis. The first module calculates the enrichment scores of a given chromatin mark on a given set of genomic locations of interest. The second module, while similar to the first, adds the additional functionality of clustering the results by additional parameters, e.g. gene expression levels. All results of these modules are then projected onto a heat map, which can be exported for further research. While Chromatra’s ease-of-use and versatility are common characteristics between it and HebbPlot, HebbPlot takes a dramatically different approach to how it clusters data. Whereas Chromatra handles enrichment levels in genomic regions of variable length through binning, HebbPlot will extract the same number of points for any region. HebbPlot will then utilize an artificial neural network to derive a representative pattern for the chromatin marks across all of the points in every region. Our tool proceeds to cluster the patterns for each chromatin mark according to their similarity to each other, and then produces a heat map of the results. Therefore, rather than evaluate genomic regions that have been mapped to chromatin marks, HebbPlot summarizes the distribution of each chromatin mark across a “representative” region. This allows researchers to only have to view one heat map before acquiring a solid understanding of how the histone modifications are represented across the regions.

ChAsE and HebbPlot have their basis in displaying information clearly and easily to the user. Their design philosophy is rooted in the fact that many visualization tools demand a high amount of technical knowledge that is unreasonable to expect from researchers. With this said, HebbPlot and ChAsE also diverge significantly in how they cluster the input and how they present their results. Similar to Chromatra, ChAsE will cluster regions together based on the abundance of chromatin marks (or any genomic area of interest) in each region. Afterwards, ChAsE allows the user the flexibility to inspect the clusters further via methods like K-Means clustering and signal queries. HebbPlot, as explained before, samples a fixed number of points in each given region of interest. These samples, and the overlapping marks, are then processed by an artificial neural network to determine a motif for each histone modification that is illustrative of its distribution in all given regions. The motifs for each considered modification is then clustered in a hierarchy so that all modifications of similar enrichment levels are placed together. A digital image of this detailed clustering is then produced, providing researchers with a way to quickly understand how histone marks are distributed across a representative regions.

DGW is a tool that consists of two modules. The first is an alignment and clustering module, whereas the second is a visualizer for the results. DGW is designed to “rescale and align” the histone marks of genomic regions (such as transcription start sites and splicing sites). Additionally, it hierarchically clusters the aligned marks into distinct groups. Regarding the visualization module, DGW creates heat maps and dendrograms of chromatin marks of a set of genomic locations. There are several notable similarities and differences between DGW and HebbPlot. HebbPlot is similar to DGW in that it scales the regions. However, HebbPlot implements it using a different idea. Specifically, HebbPlot samples a fixed number of equally spaced points from each region regardless of the region length. HebbPlot learns a general pattern of chromatin marks summarizing all of the input regions as one representative region. Unlike DGW, hebbPlot does not cluster the input regions based on the distribution of a mark. Hierarchical clustering is utilized in HebbPlot not to cluster the regions according to the enrichment of a mark, but to cluster all marks according to their distributions around the representative region. The amount of details produced by DGW can be inappropriate in the presence a large number of marks and regions. HebbPlot on the other hand, is built specifically to make large amounts of data manageable and meaningful for biologists through its summarization technique.

Our comparisons regarding these four tools makes it clear that the advantages provided by HebbPlot are not well represented among related tools. There are numerous tools for clustering regions according to the abundance of chromatin marks, but besides conventional plots, there are hardly any techniques for determining the patterns of marks across all regions. This means it is important for HebbPlot to coexist among other popular visualization tools. Its unique and concise summarization of data is vital to evaluating a large number of chromatin signals across thousands of regions. This is not to say that the level of description provided by other tools is not useful. Indeed, biologists need to be able to see the specific results that other utilities facilitate. However, what HebbPlot offers is a look at the “big picture” of the data.

### Selection of region size in our case studies

Two reasons led us to choose 400 base pairs (bp) as the size of enhancers and promoters in some case studies. Frist, the average size of the enhancers obtained from the Fantom project is around 400 bp. In the Fantom project, the whole region was determined according to eRNA (enhancer RNA) not only the peak as with the P300. Second, this size was necessary in some case studies; for example, to make sure that the promoter signature is as accurate as possible, we needed to limit the size to 400 bp to minimize the overlap with untranslated and coding regions. However, in other case studies such as the one involving the directionality of the promoter signature, we used 4400 bp to see outside the promoter regions. Additionally, HebbPlot can process regions of any size. We have conducted some experiments using sizes ranging from 200 bp to 5000 bp. See Additional file 9: Figure S1 and Additional file 10: Figure S2. The two figures suggest that 400 bp are reasonable to show the signature of promoters and enhancers active in H1.

### Handling regions with variable sizes

**Fig. 10:**
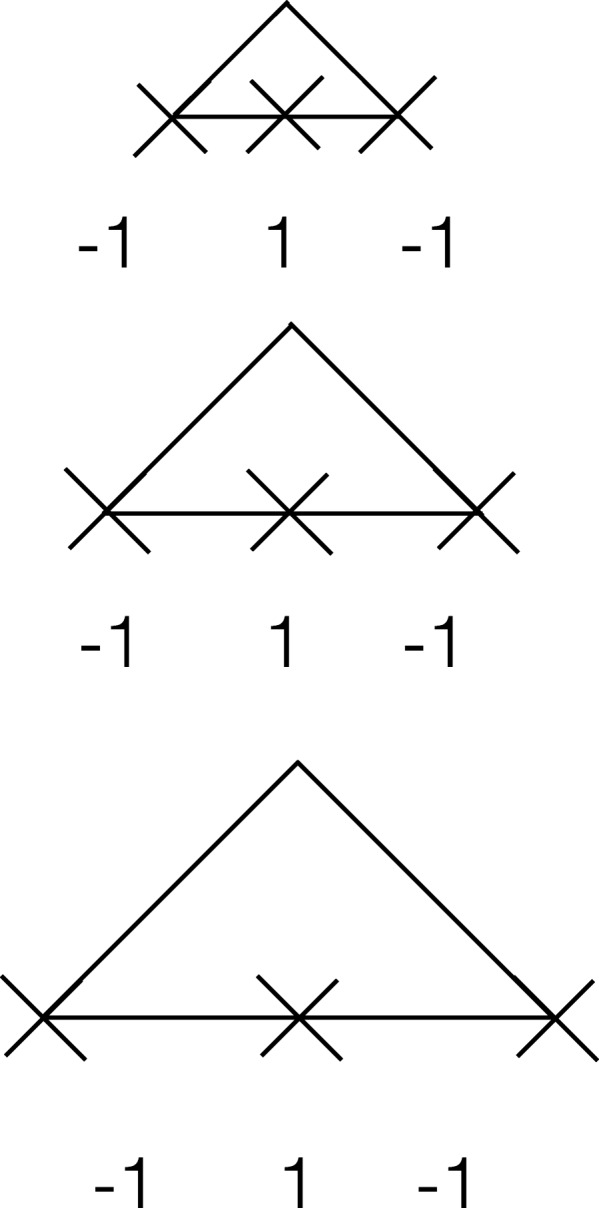
The advantage of HebbPlot is clear when looking at variable-sized regions. Each triangle represents the distributions of chromatin marks around a region. The three equally-spaced samples (X) obtained from each region give a rise to a pattern of low signal (-1), high signal (1), and low signal (-1). Conventional plots wouldn’t detect this pattern because of the differences in length. Hebbplot, however, will rescale these triangles and present the correct signature

Handing regions of the same size, e.g. promoters, is straight forward; however, handling regions of variable sizes, e.g. coding regions, requires rescaling. One drawback of conventional plots is that they do not account for length difference, resulting in an artificial peak(s) due to small regions. Our approach to sample fixed number of points from each region in a data set works on regions that have variable or similar lengths and is supported by the histone code hypothesis. If histone marks are distributed in a similar way around regions that have the same function, then sampling equally-spaced points from these regions should capture the histone signature. In some sense, this is a rescaling process. To illustrate, imagine three triangles of different sizes (Fig. 10) representing the distributions of chromatin marks around three regions. If we take three equally-spaced samples from each region then these samples should capture a simple, yet accurate, representation of the chromatin signature — low signal, high signal, followed by low signal. Using more samples should result in a better representation of a signature. In sum, our approach, which is supported by the histone code hypothesis, allows for extracting signatures from regions with variable or fixed lengths.

## Conclusions

In this manuscript, we described a new software tool, HebbPlot, for learning and visualizing the chromatin signature of a genetic element. HebbPlot produces an image that can be interpreted easily. Signatures learned by HebbPlot can be compared quantitatively. We validated HebbPlot in six case studies using 57 human tissues and cell types. The results of these case studies are novel or confirming to previously reported results in the literature, indicating the accuracy of HebbPlot. We found that active promoters have a directional chromatin signature; specifically, H3K4me3 and H3K79me2 tend to stretch downstream, whereas H2A.Z tends to stretch upstream. Our results confirm that high-CpG and low-CpG promoters have different chromatin signatures. When we compared the signatures of enhancers active in eight tissues/cell types, we found that they are similar, but not identical. Contrasting the signatures of coding regions of active and inactive genes revealed that certain modifications— H3K36me3, H3K79(me1,me2), and H4K8ac — mark active coding regions, whereas different modifications — H4K12ac, H3K14ac, H3K27me3 and H2AK5ac — mark coding regions of inactive genes. Our study resulted in a visual catalog of chromatin signatures of multiple genetic elements in 57 human tissues and cell types. Further, we made a progress toward a functional catalog of more than 20 histone marks. Finally, HebbPlot is a general tool that can be applied to a large number of studies, facilitating the understanding of the histone code.

## Availability and requirements

The source code (Perl and Matlab) is available as Additional file 11.

**Project name:** HebbPlot.

**Project home page:** https://github.com/TulsaBioinformaticsToolsmith/HebbPlot

**Operating system(s):** UNIX/Linux/Mac.

**Programming language:** Perl and Matlab.

**Other requirements:** Matlab Statistics and Machine Learning Toolbox and Bedtools (http://bedtools.readthedocs.io/en/latest/).

**License:** Creative Commons license (attribution + non-commercial + no derivative works).

**Any restrictions to use by non-academics:** License needed.

## Additional files

Additional file 1 HebbPlots of active promoters on the positive strand. This compressed file (.tar.gz) includes HebbPlots of promoters on the positive strand active in 57 tissues/cell types. (TAR 2949 kb)

Additional file 2 HebbPlots of active promoters on the negative strand. This compressed file (.tar.gz) includes HebbPlots of promoters on the negative strand active in 57 tissues/cell types. (TAR 2952 kb)

Additional file 3 HebbPlots of high-CpG promoters. This compressed file (.tar.gz) includes HebbPlots of high-CpG promoters active in 57 tissues/cell types. (TAR 2654 kb)

Additional file 4 HebbPlots of low-CpG promoters. This compressed file (.tar.gz) includes HebbPlots of low-CpG promoters active in 57 tissues/cell types. (TAR 2971 kb)

Additional file 5 HebbPlots of active enhancers. This compressed file (.tar.gz) includes HebbPlots of enhancers active in eight tissues/cell types. (TAR 439 kb)

Additional file 6 Gene identifiers. This compressed file (.tar.gz) includes identifiers of nine groups of genes divided according to their gene expression levels in IMR90. (TAR 428 kb)

Additional file 7 HebbPlots of coding regions of active genes. This compressed file (.tar.gz) includes HebbPlots of genes active in 57 tissues/cell types. (TAR 2696 kb)

Additional file 8 HebbPlots of coding regions of inactive genes. This compressed file (.tar.gz) includes HebbPlots of genes inactive in 57 tissues/cell types. (TAR 2715 kb)

Additional file 9 **Figure S1.** HebbPlots of enhancers specific to H1 cell line. These plots were generated from enhancers with different sizes. Each HebbPlot was generated from a set of enhancers, all of which have the same size and are centered on the P300 peaks. (PDF 4881 kb)

Additional file 10 **Figure S2.** HebbPlots of promoters active in H1 cell line. These plots were generated from promoters with different sizes. Each HebbPlot was generated from a set of promoters, all of which have the same size and are centered on the transcription start sites. (PDF 5010 kb)

Additional file 11 HebbPlot Software. This compressed file (.tar.gz) includes the source code (Matlab and Perl) of HebbPlot. (TAR 15 kb)

## Abbreviations

bp: Base pairs
