# Supplementary figures and images for: HebbPlot: an intelligent tool for learning and visualizing chromatin mark signatures (part 1 of 4)
Source: BMC Bioinformatics. 2018 Sep 3;19:310. doi: 10.1186/s12859-018-2312-1 (PMC6122555; doi:10.1186/s12859-018-2312-1)

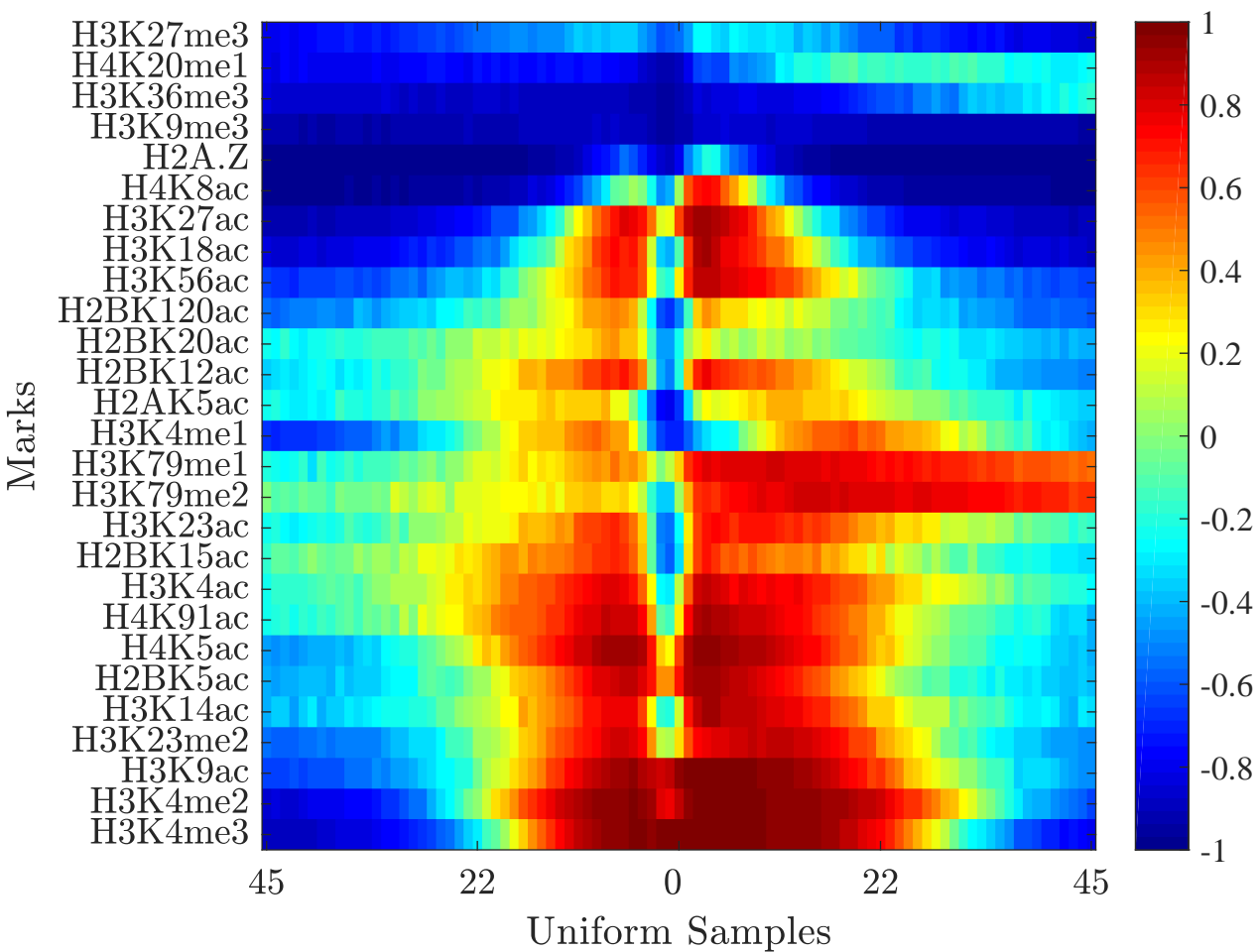

Supplement: Supplementary file 1 — HebbPlots of active promoters on the positive strand. This compressed file (.tar.gz) includes HebbPlots of promoters on the positive strand active in 57 tissues/cell types. (TAR 2949 kb) [file 12859_2018_2312_MOESM1_ESM.tar › file2/E003.pdf]

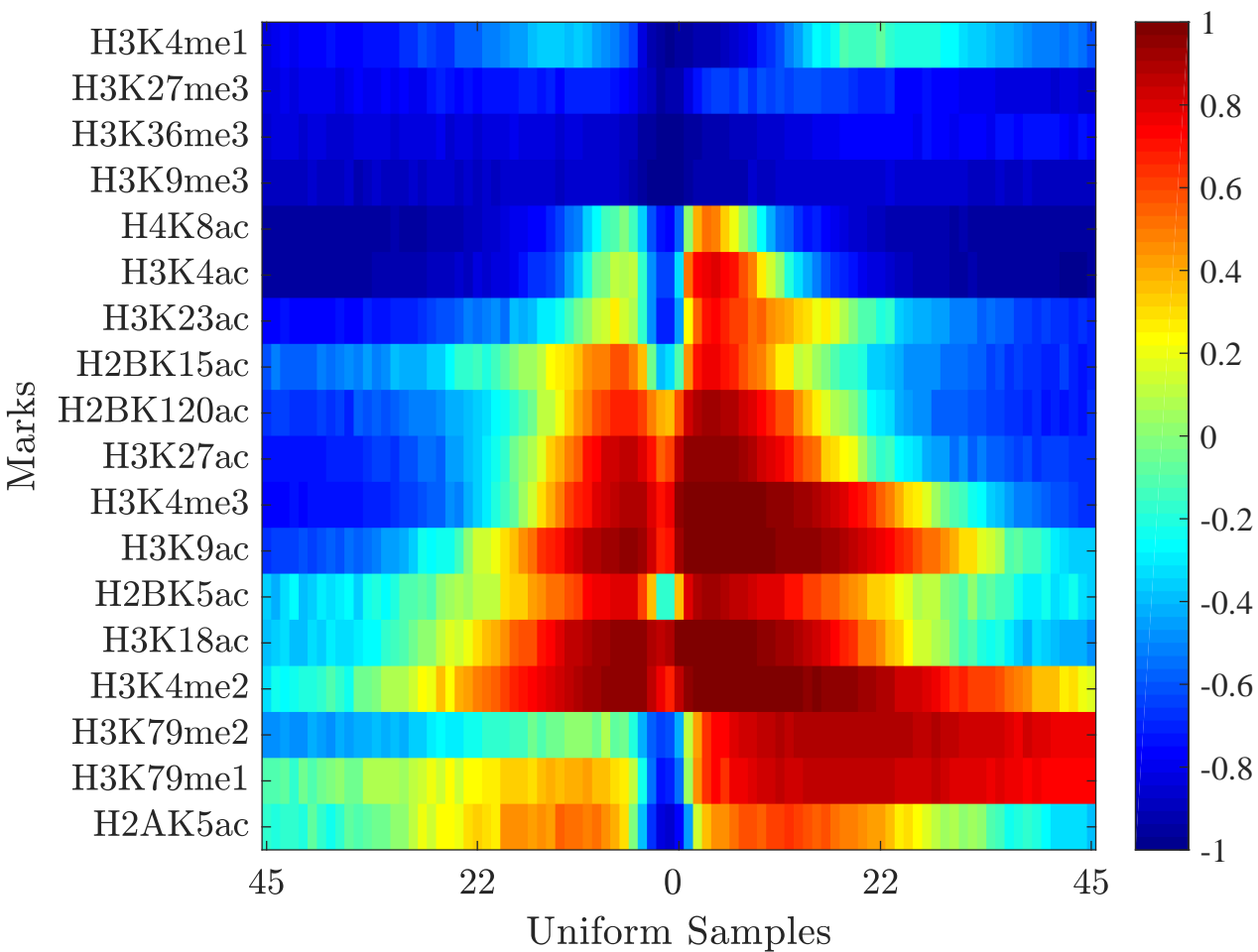

Supplement: Supplementary file 1 — HebbPlots of active promoters on the positive strand. This compressed file (.tar.gz) includes HebbPlots of promoters on the positive strand active in 57 tissues/cell types. (TAR 2949 kb) [file 12859_2018_2312_MOESM1_ESM.tar › file2/E004.pdf]

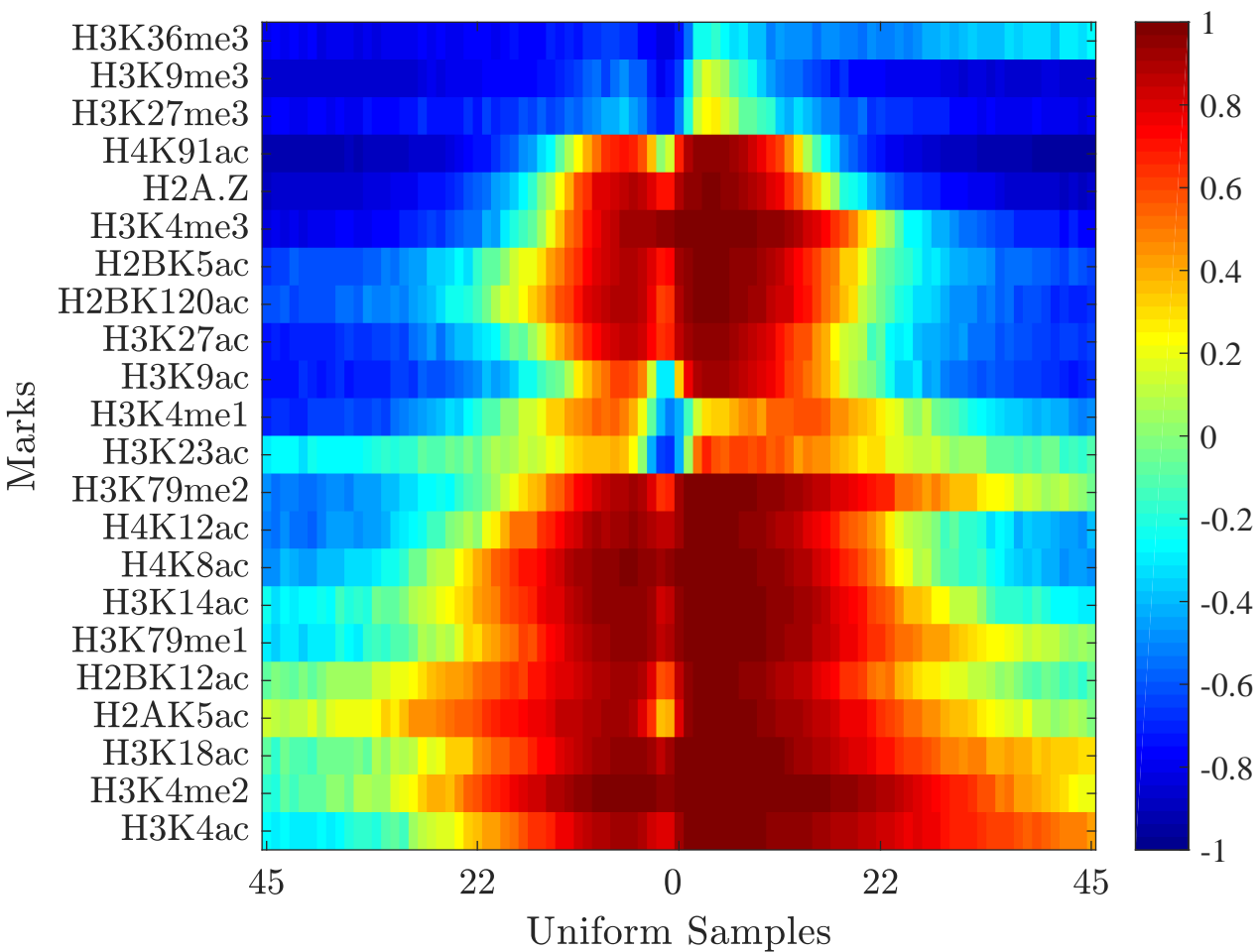

Supplement: Supplementary file 1 — HebbPlots of active promoters on the positive strand. This compressed file (.tar.gz) includes HebbPlots of promoters on the positive strand active in 57 tissues/cell types. (TAR 2949 kb) [file 12859_2018_2312_MOESM1_ESM.tar › file2/E005.pdf]

Marks

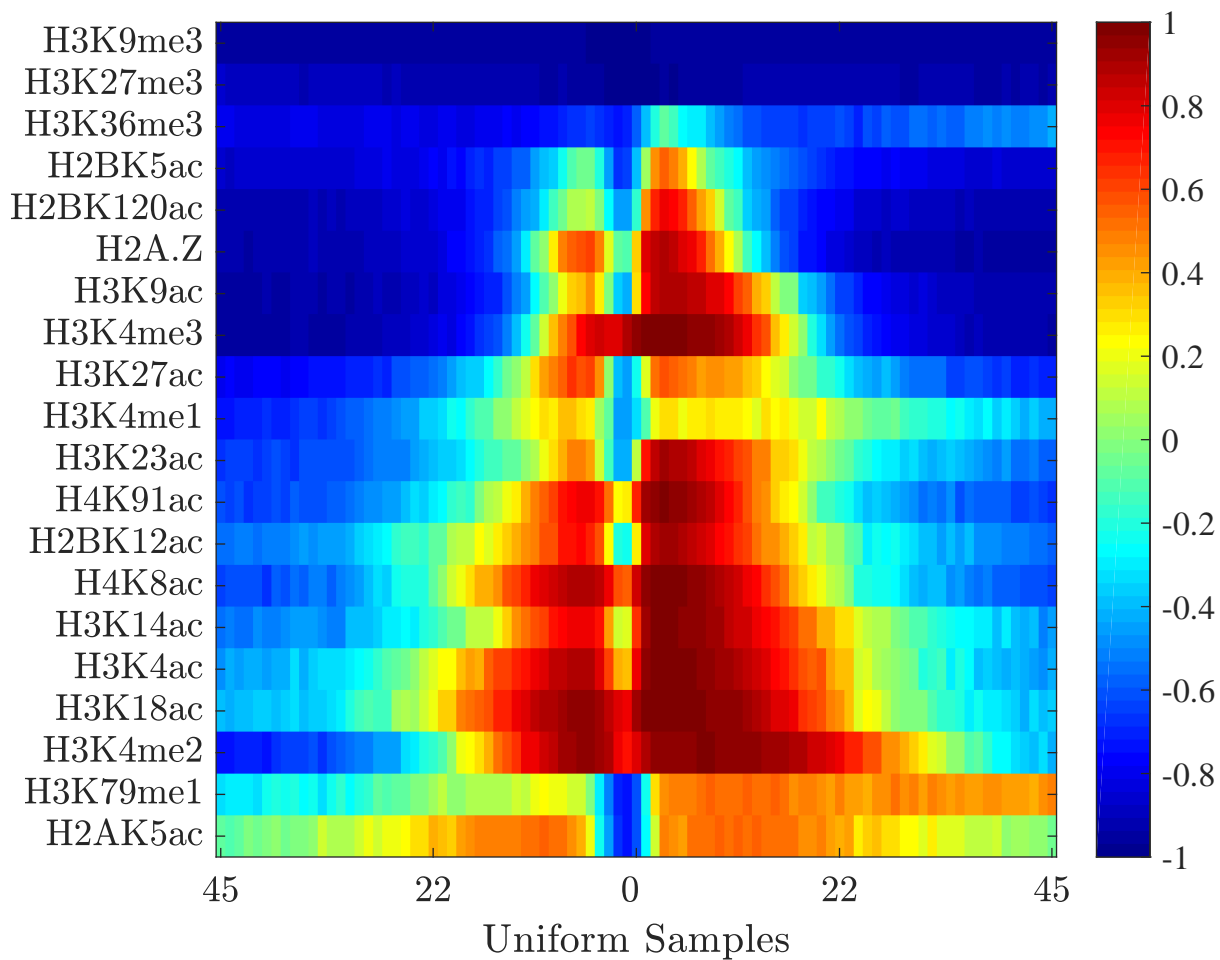

Supplement: Supplementary file 1 — HebbPlots of active promoters on the positive strand. This compressed file (.tar.gz) includes HebbPlots of promoters on the positive strand active in 57 tissues/cell types. (TAR 2949 kb) [file 12859_2018_2312_MOESM1_ESM.tar › file2/E006.pdf]

Marks

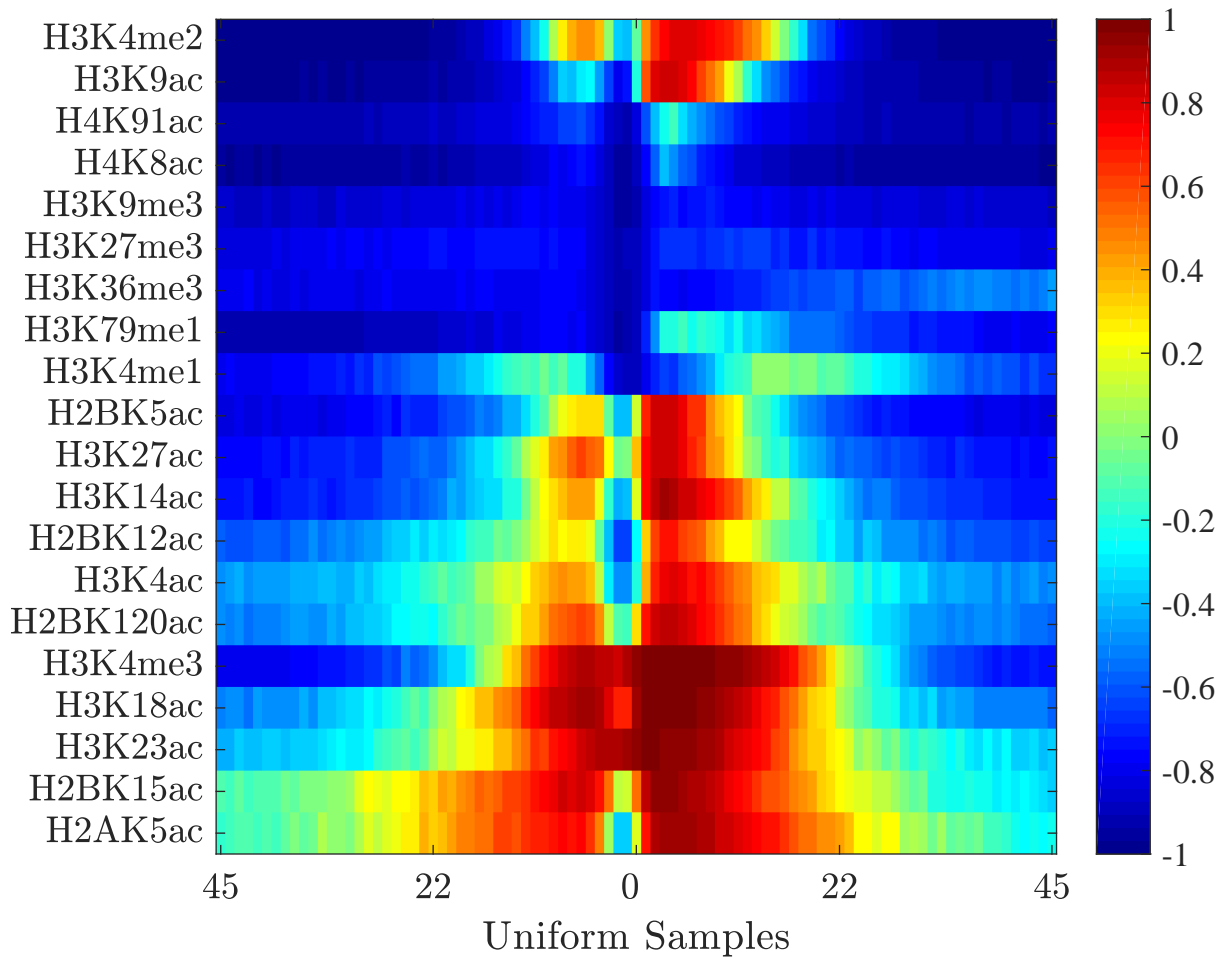

Supplement: Supplementary file 1 — HebbPlots of active promoters on the positive strand. This compressed file (.tar.gz) includes HebbPlots of promoters on the positive strand active in 57 tissues/cell types. (TAR 2949 kb) [file 12859_2018_2312_MOESM1_ESM.tar › file2/E007.pdf]

Marks

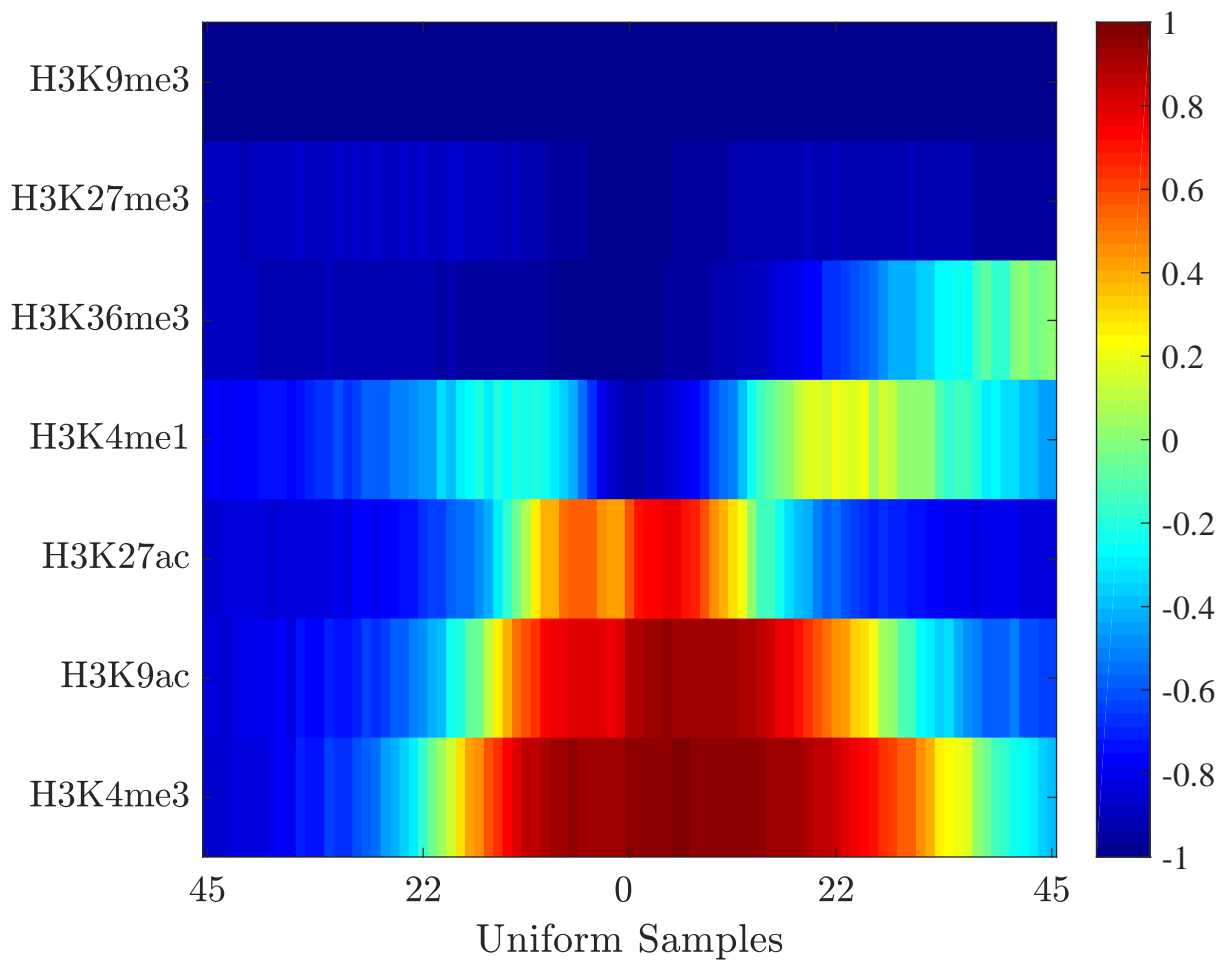

Supplement: Supplementary file 1 — HebbPlots of active promoters on the positive strand. This compressed file (.tar.gz) includes HebbPlots of promoters on the positive strand active in 57 tissues/cell types. (TAR 2949 kb) [file 12859_2018_2312_MOESM1_ESM.tar › file2/E011.pdf]

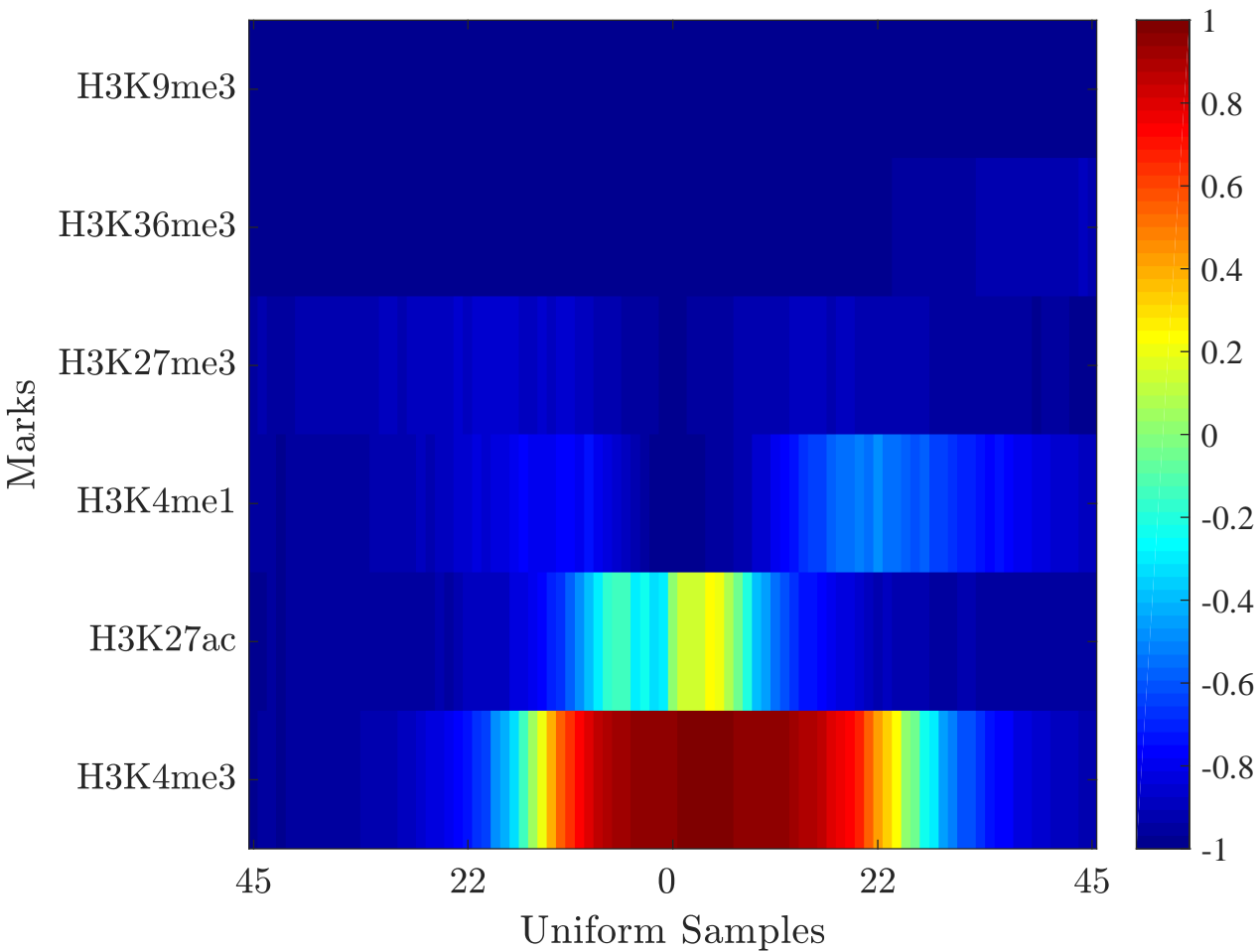

Supplement: Supplementary file 1 — HebbPlots of active promoters on the positive strand. This compressed file (.tar.gz) includes HebbPlots of promoters on the positive strand active in 57 tissues/cell types. (TAR 2949 kb) [file 12859_2018_2312_MOESM1_ESM.tar › file2/E012.pdf]

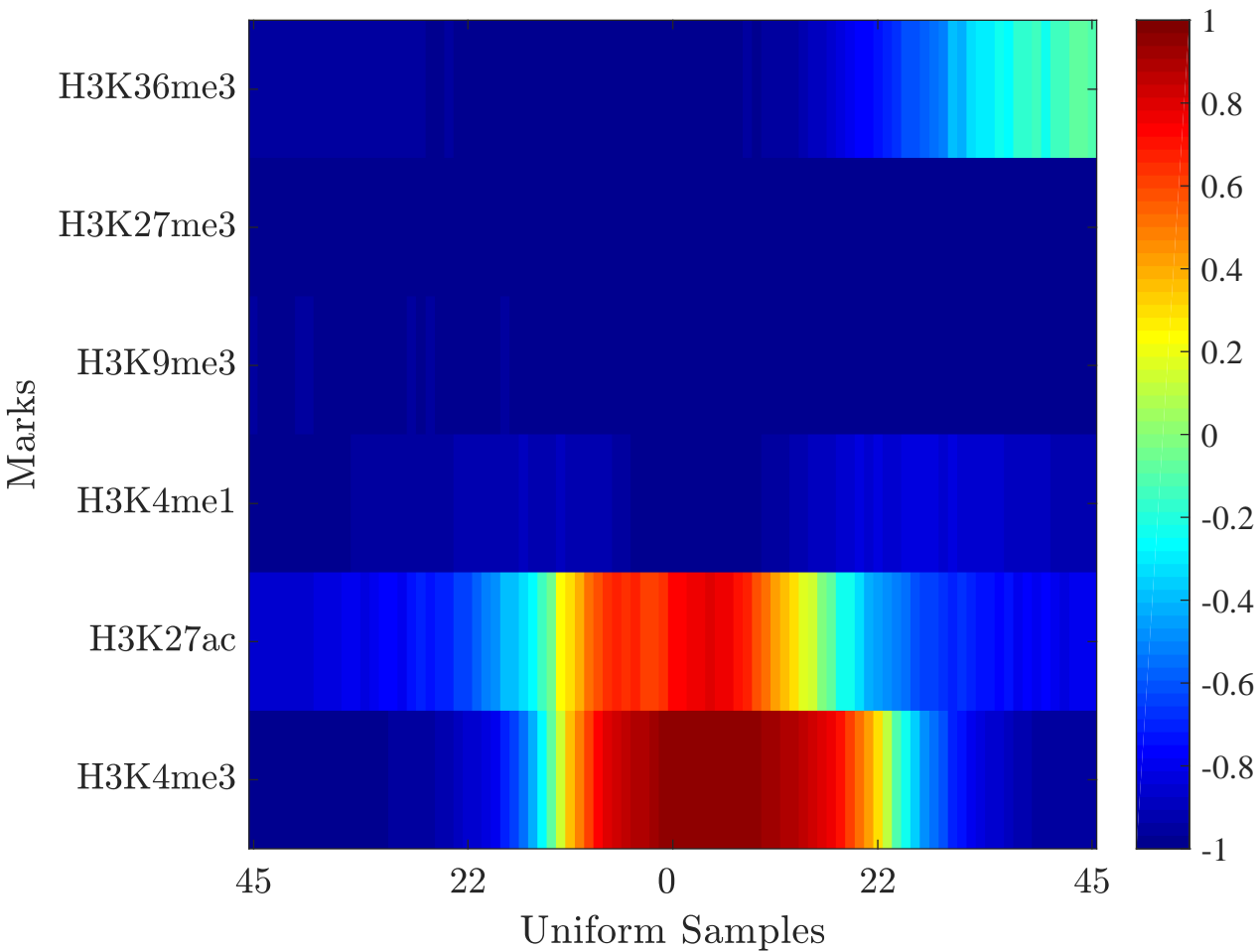

Supplement: Supplementary file 1 — HebbPlots of active promoters on the positive strand. This compressed file (.tar.gz) includes HebbPlots of promoters on the positive strand active in 57 tissues/cell types. (TAR 2949 kb) [file 12859_2018_2312_MOESM1_ESM.tar › file2/E013.pdf]

Marks

H3K36me3

H3K9me3

H3K27me3

H3K4me1

H3K27ac

H3K9ac

H3K4me3

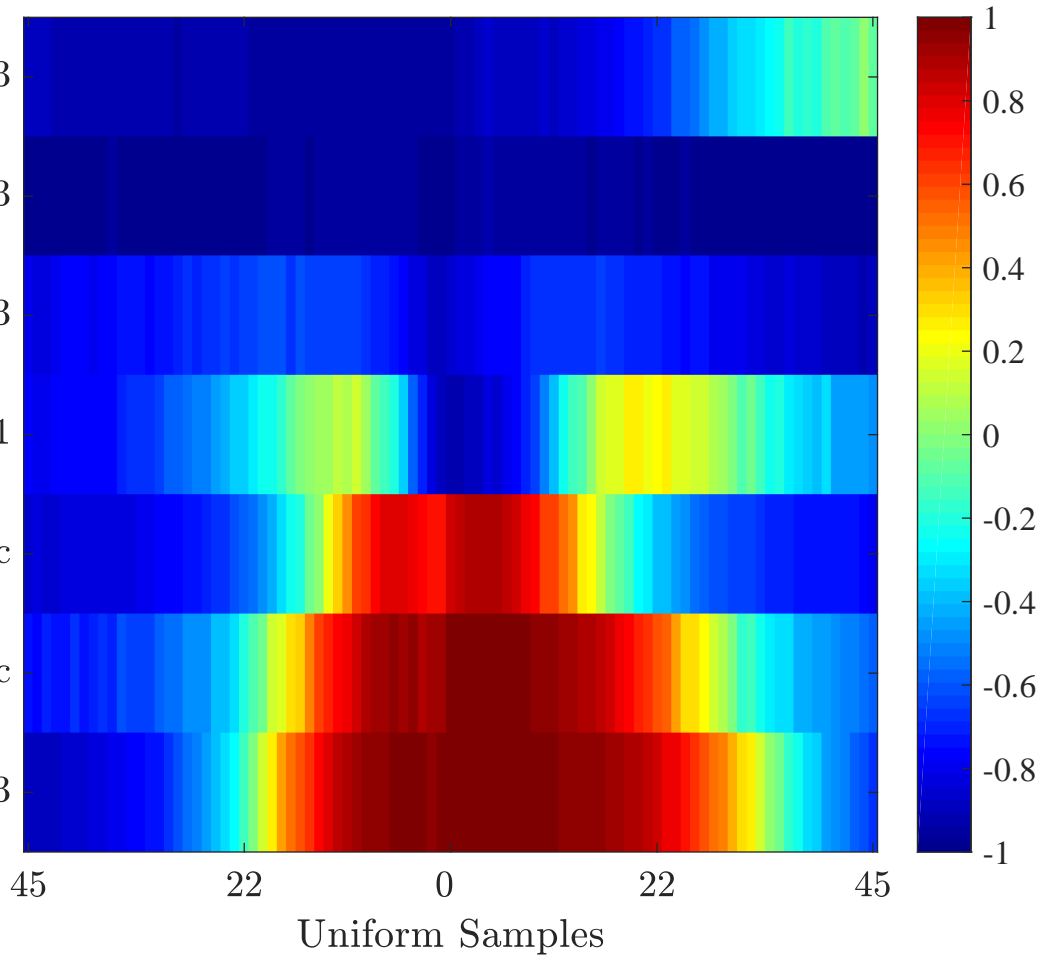

Supplement: Supplementary file 1 — HebbPlots of active promoters on the positive strand. This compressed file (.tar.gz) includes HebbPlots of promoters on the positive strand active in 57 tissues/cell types. (TAR 2949 kb) [file 12859_2018_2312_MOESM1_ESM.tar › file2/E016.pdf]

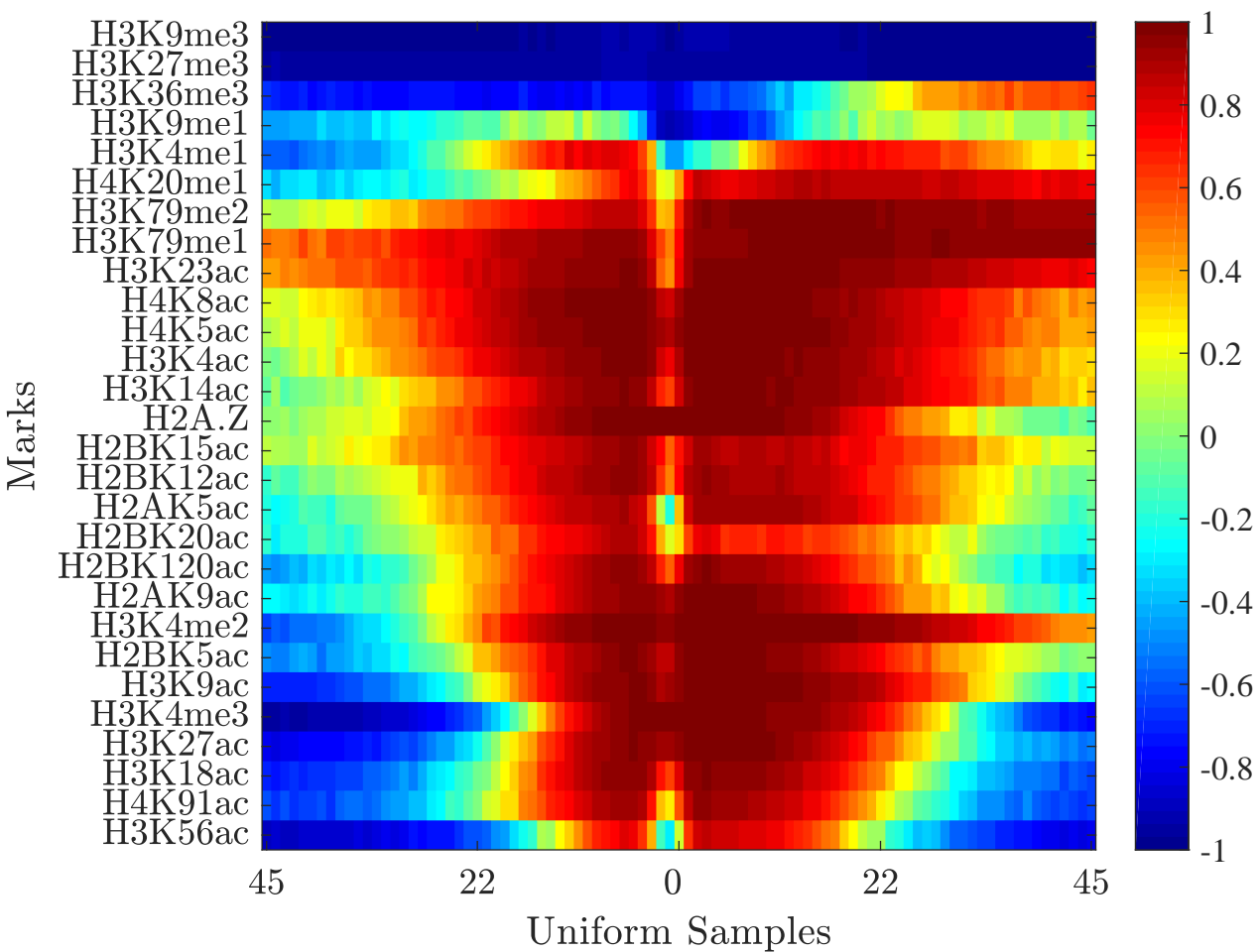

Supplement: Supplementary file 1 — HebbPlots of active promoters on the positive strand. This compressed file (.tar.gz) includes HebbPlots of promoters on the positive strand active in 57 tissues/cell types. (TAR 2949 kb) [file 12859_2018_2312_MOESM1_ESM.tar › file2/E017.pdf]

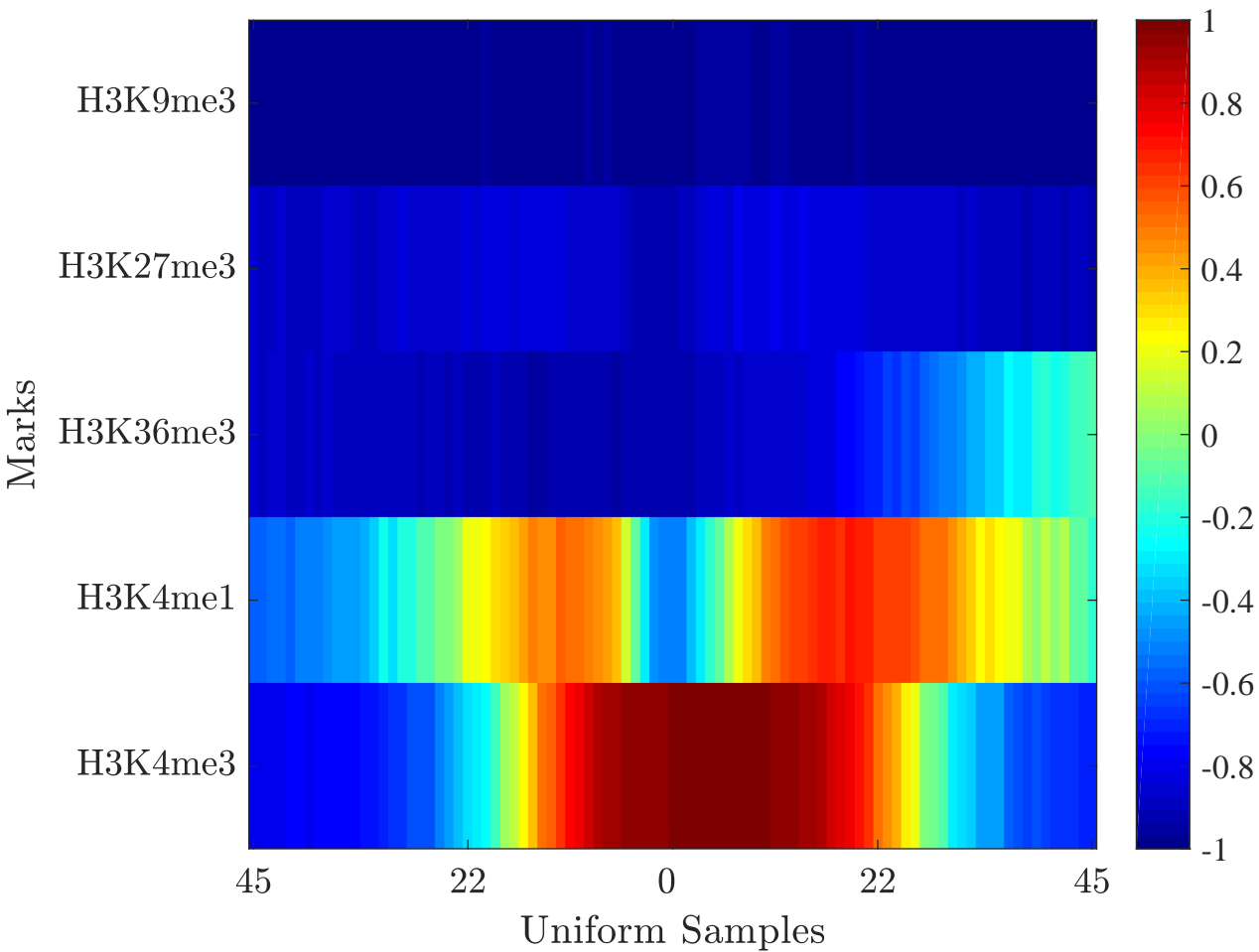

Supplement: Supplementary file 1 — HebbPlots of active promoters on the positive strand. This compressed file (.tar.gz) includes HebbPlots of promoters on the positive strand active in 57 tissues/cell types. (TAR 2949 kb) [file 12859_2018_2312_MOESM1_ESM.tar › file2/E024.pdf]

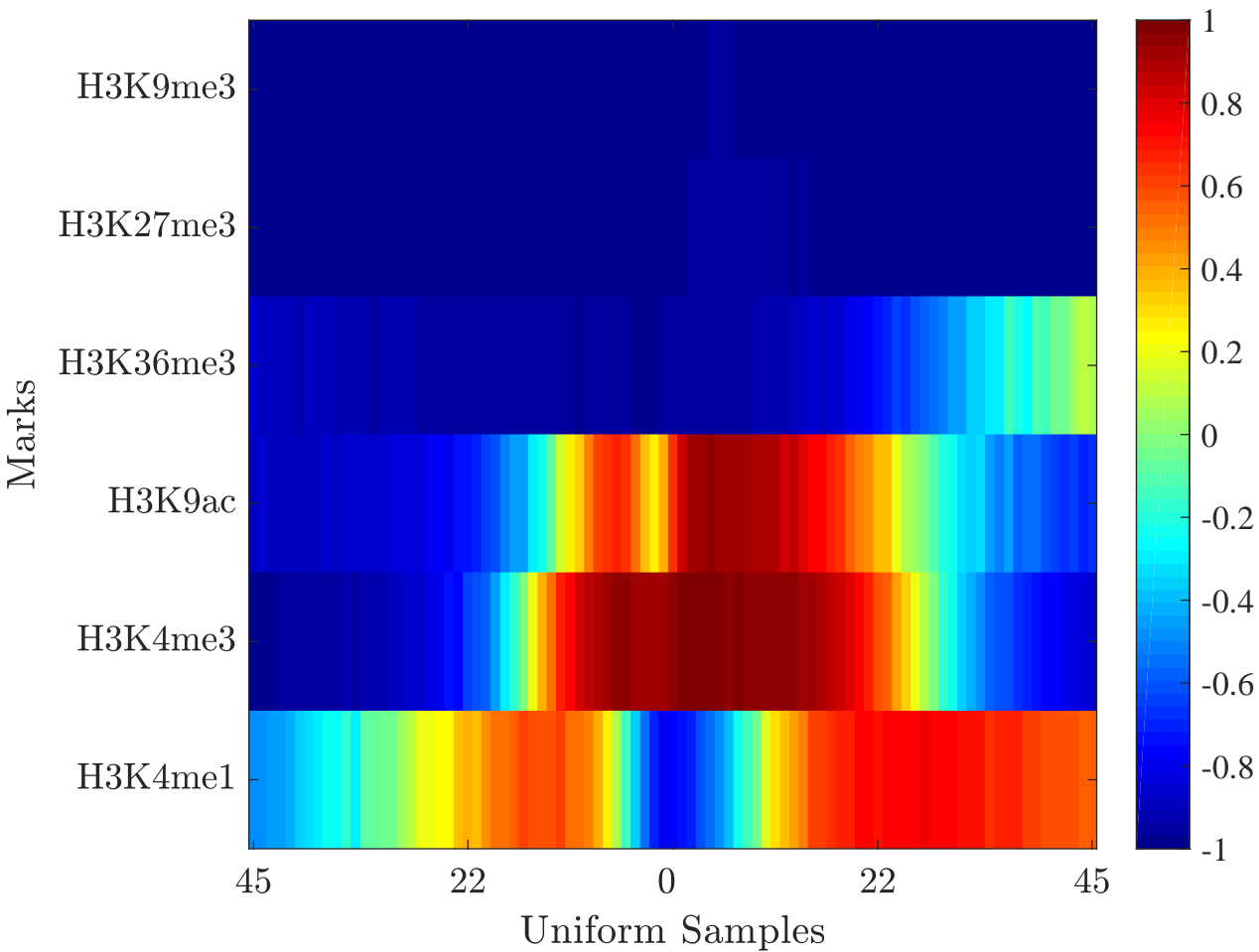

Supplement: Supplementary file 1 — HebbPlots of active promoters on the positive strand. This compressed file (.tar.gz) includes HebbPlots of promoters on the positive strand active in 57 tissues/cell types. (TAR 2949 kb) [file 12859_2018_2312_MOESM1_ESM.tar › file2/E027.pdf]

Marks

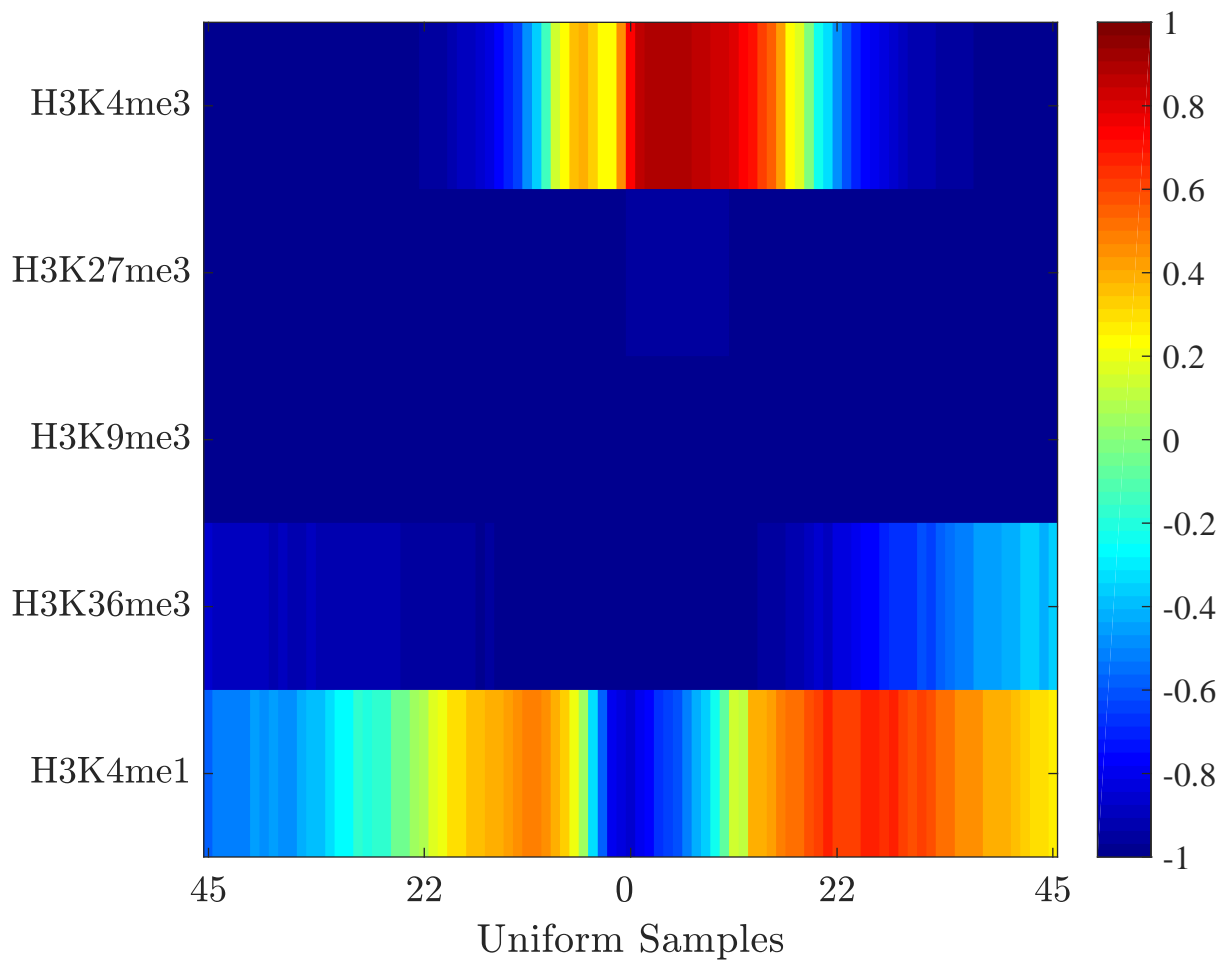

Supplement: Supplementary file 1 — HebbPlots of active promoters on the positive strand. This compressed file (.tar.gz) includes HebbPlots of promoters on the positive strand active in 57 tissues/cell types. (TAR 2949 kb) [file 12859_2018_2312_MOESM1_ESM.tar › file2/E028.pdf]

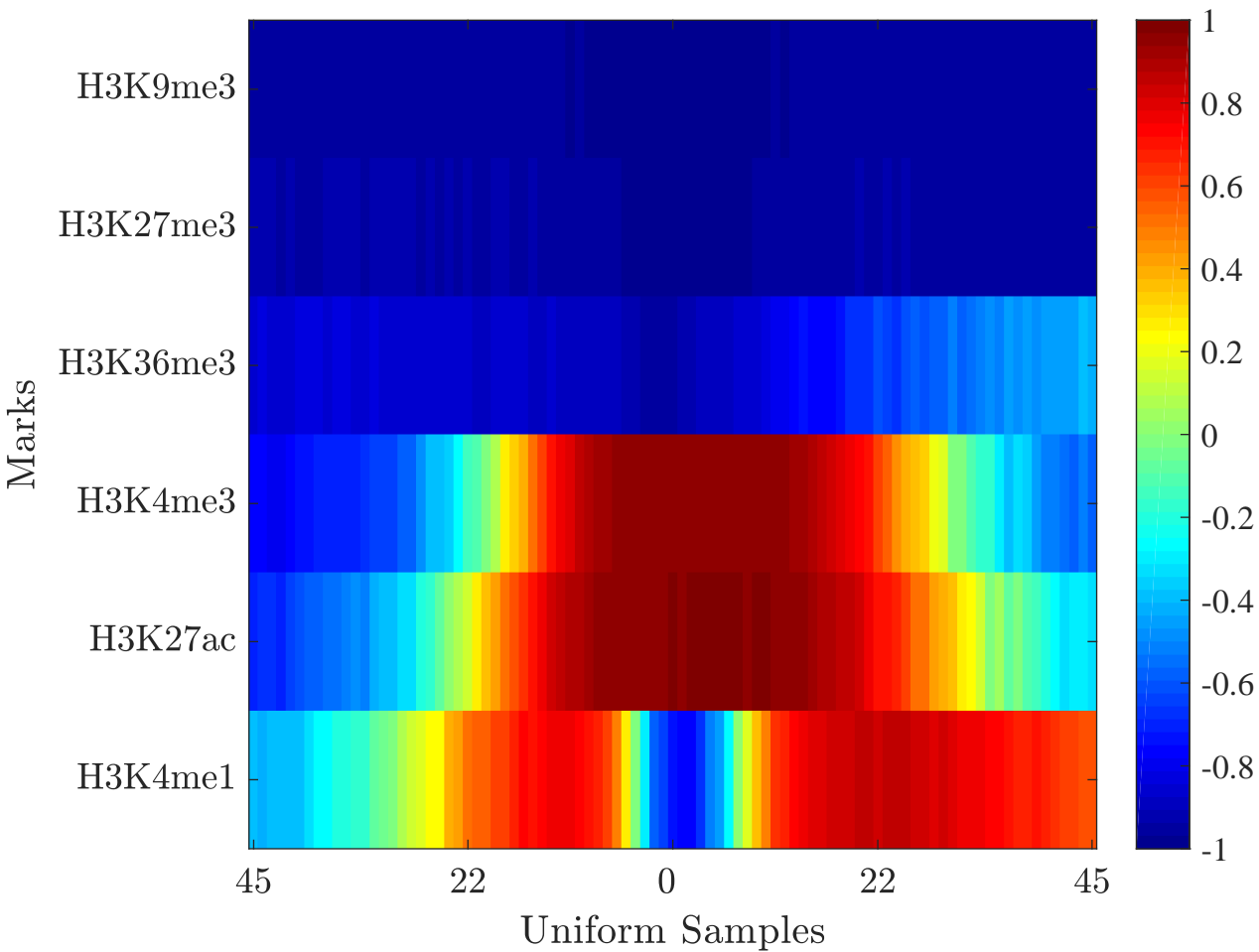

Supplement: Supplementary file 1 — HebbPlots of active promoters on the positive strand. This compressed file (.tar.gz) includes HebbPlots of promoters on the positive strand active in 57 tissues/cell types. (TAR 2949 kb) [file 12859_2018_2312_MOESM1_ESM.tar › file2/E037.pdf]

Marks

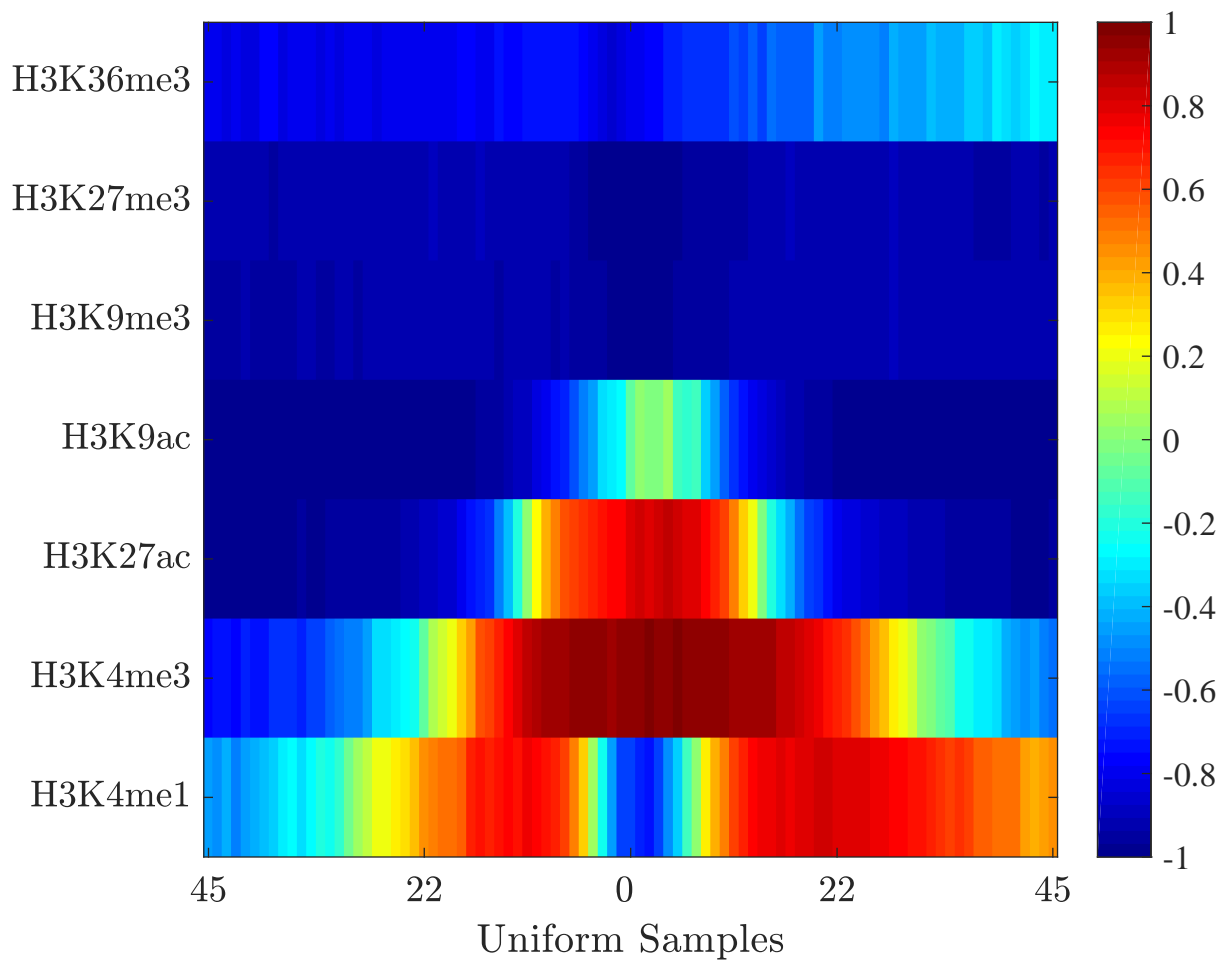

Supplement: Supplementary file 1 — HebbPlots of active promoters on the positive strand. This compressed file (.tar.gz) includes HebbPlots of promoters on the positive strand active in 57 tissues/cell types. (TAR 2949 kb) [file 12859_2018_2312_MOESM1_ESM.tar › file2/E038.pdf]

Marks

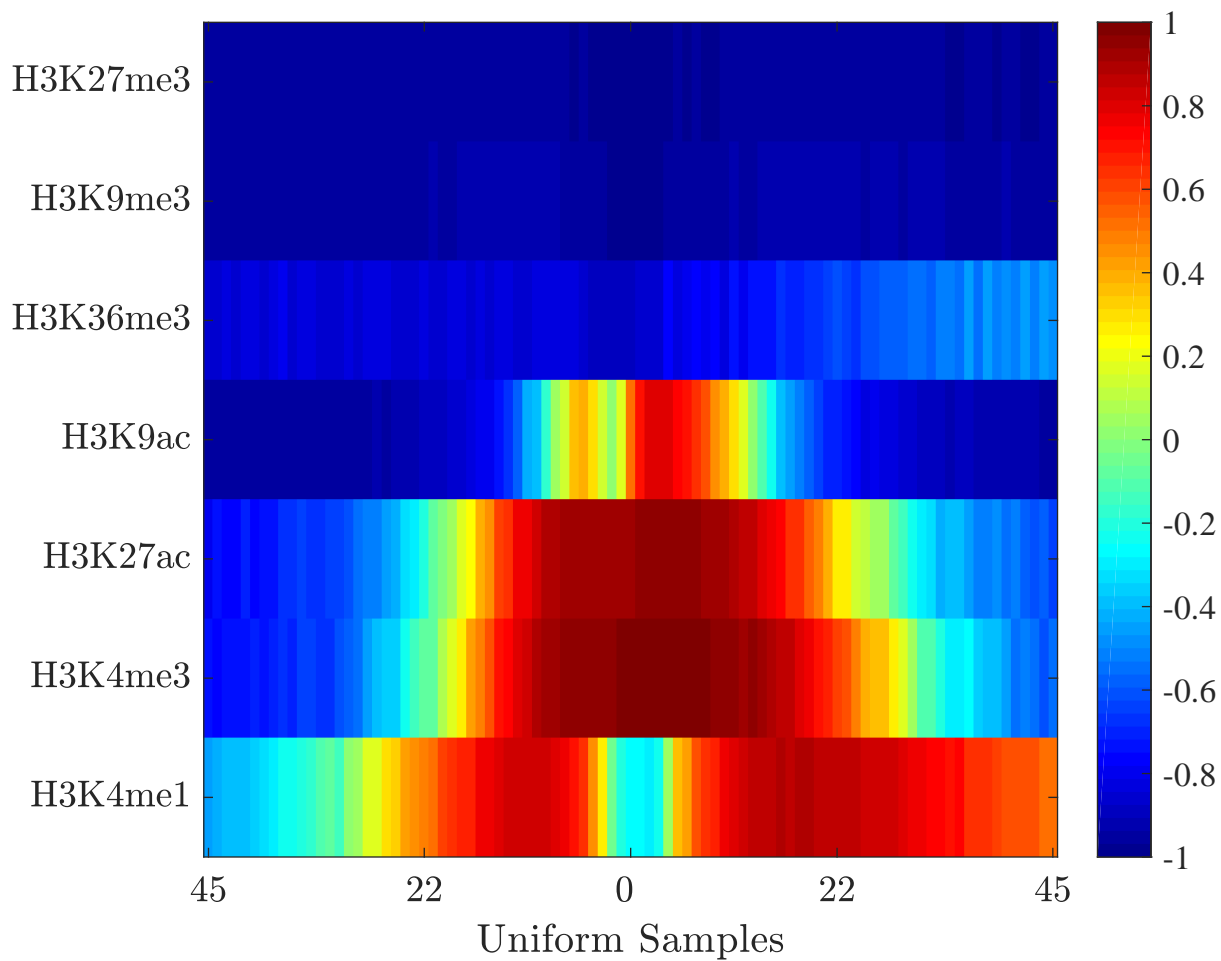

Supplement: Supplementary file 1 — HebbPlots of active promoters on the positive strand. This compressed file (.tar.gz) includes HebbPlots of promoters on the positive strand active in 57 tissues/cell types. (TAR 2949 kb) [file 12859_2018_2312_MOESM1_ESM.tar › file2/E047.pdf]

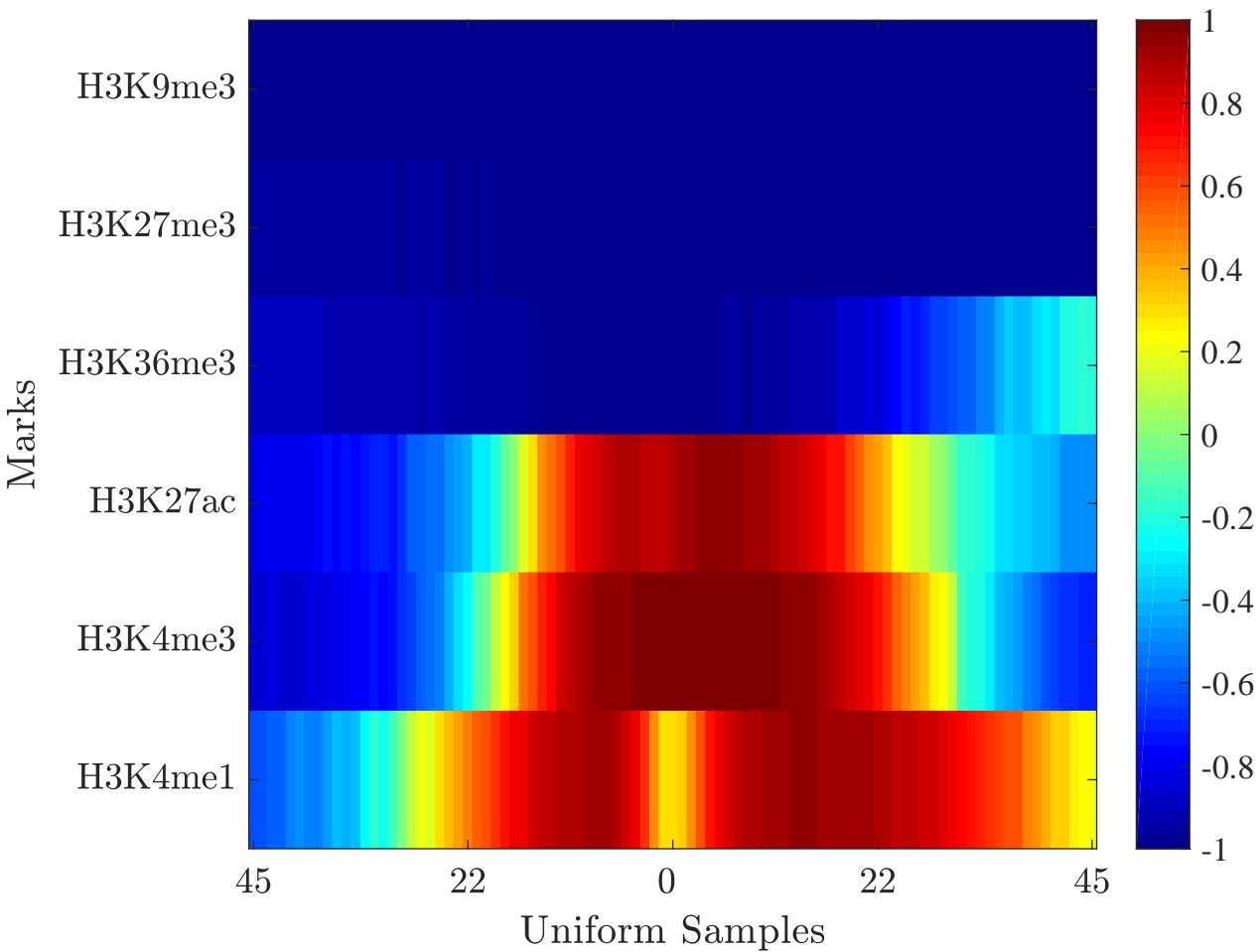

Supplement: Supplementary file 1 — HebbPlots of active promoters on the positive strand. This compressed file (.tar.gz) includes HebbPlots of promoters on the positive strand active in 57 tissues/cell types. (TAR 2949 kb) [file 12859_2018_2312_MOESM1_ESM.tar › file2/E050.pdf]

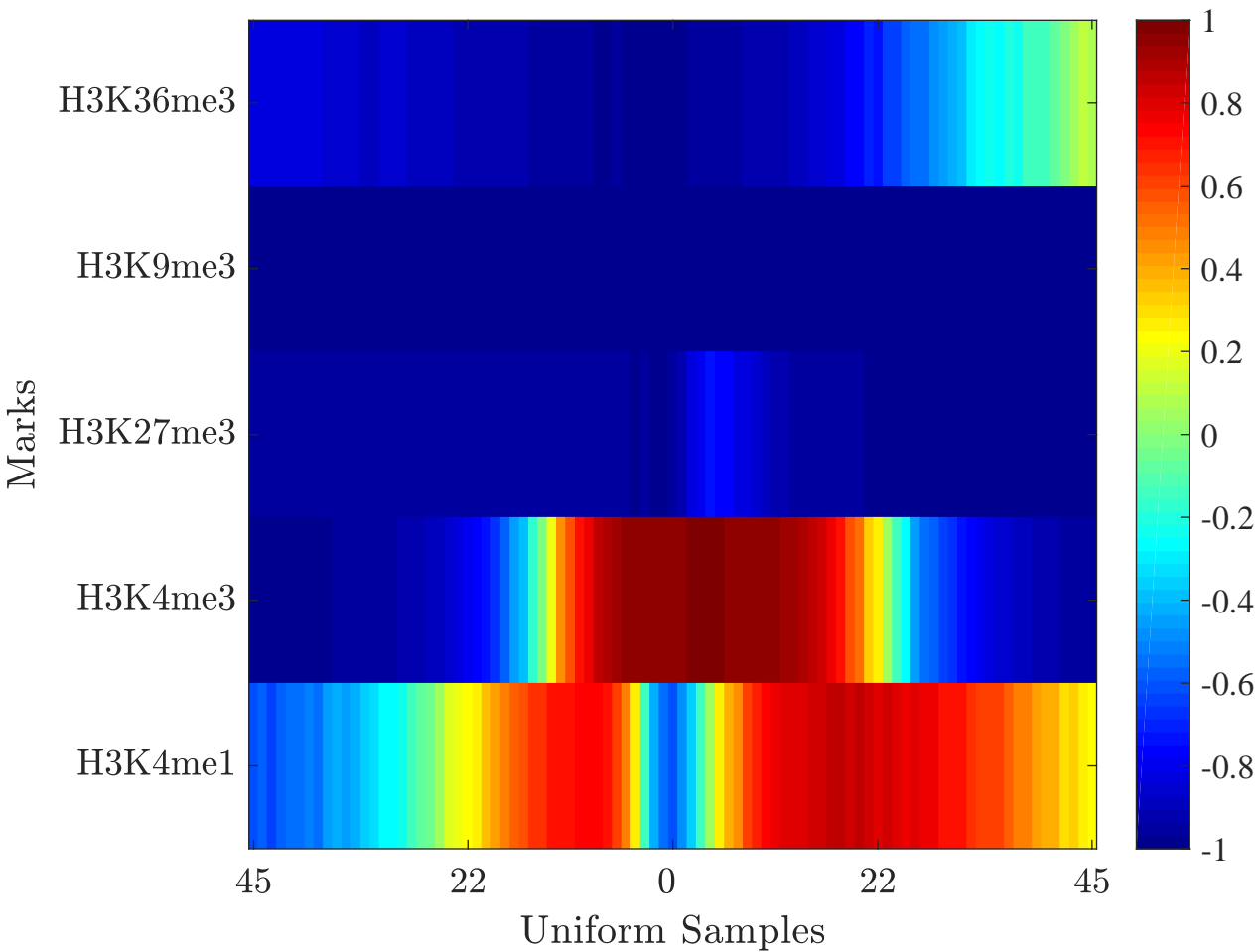

Supplement: Supplementary file 1 — HebbPlots of active promoters on the positive strand. This compressed file (.tar.gz) includes HebbPlots of promoters on the positive strand active in 57 tissues/cell types. (TAR 2949 kb) [file 12859_2018_2312_MOESM1_ESM.tar › file2/E053.pdf]

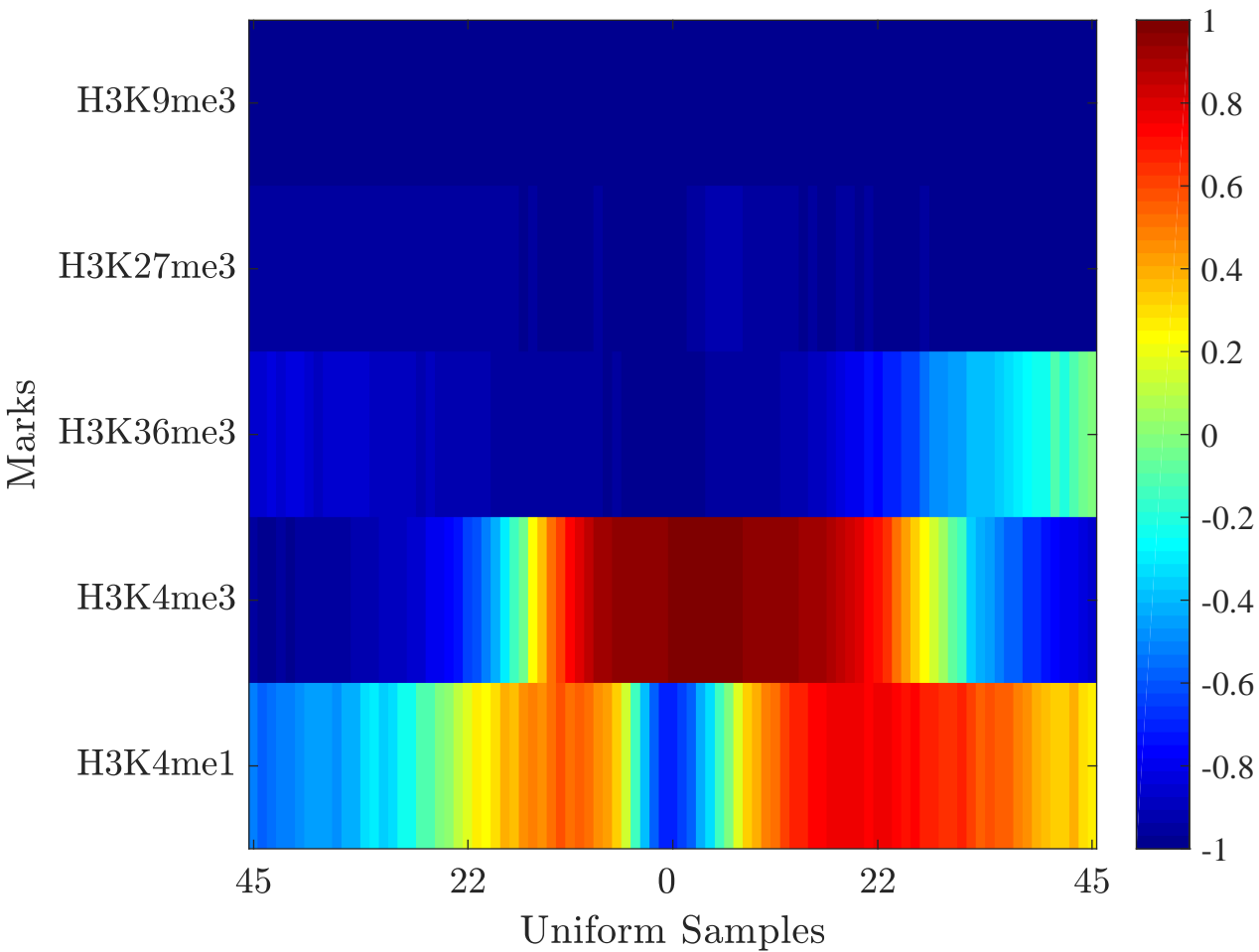

Supplement: Supplementary file 1 — HebbPlots of active promoters on the positive strand. This compressed file (.tar.gz) includes HebbPlots of promoters on the positive strand active in 57 tissues/cell types. (TAR 2949 kb) [file 12859_2018_2312_MOESM1_ESM.tar › file2/E054.pdf]

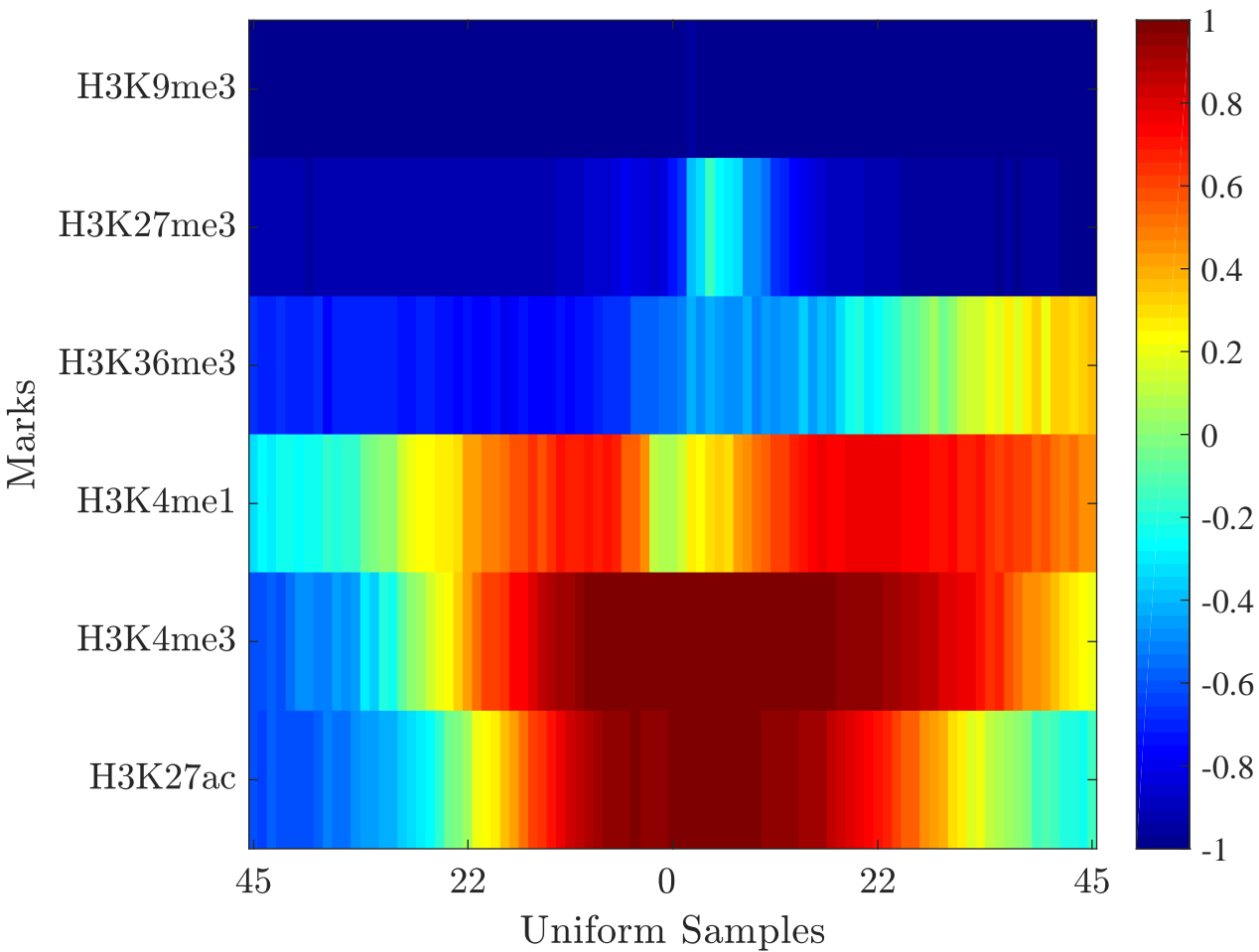

Supplement: Supplementary file 1 — HebbPlots of active promoters on the positive strand. This compressed file (.tar.gz) includes HebbPlots of promoters on the positive strand active in 57 tissues/cell types. (TAR 2949 kb) [file 12859_2018_2312_MOESM1_ESM.tar › file2/E055.pdf]

Marks

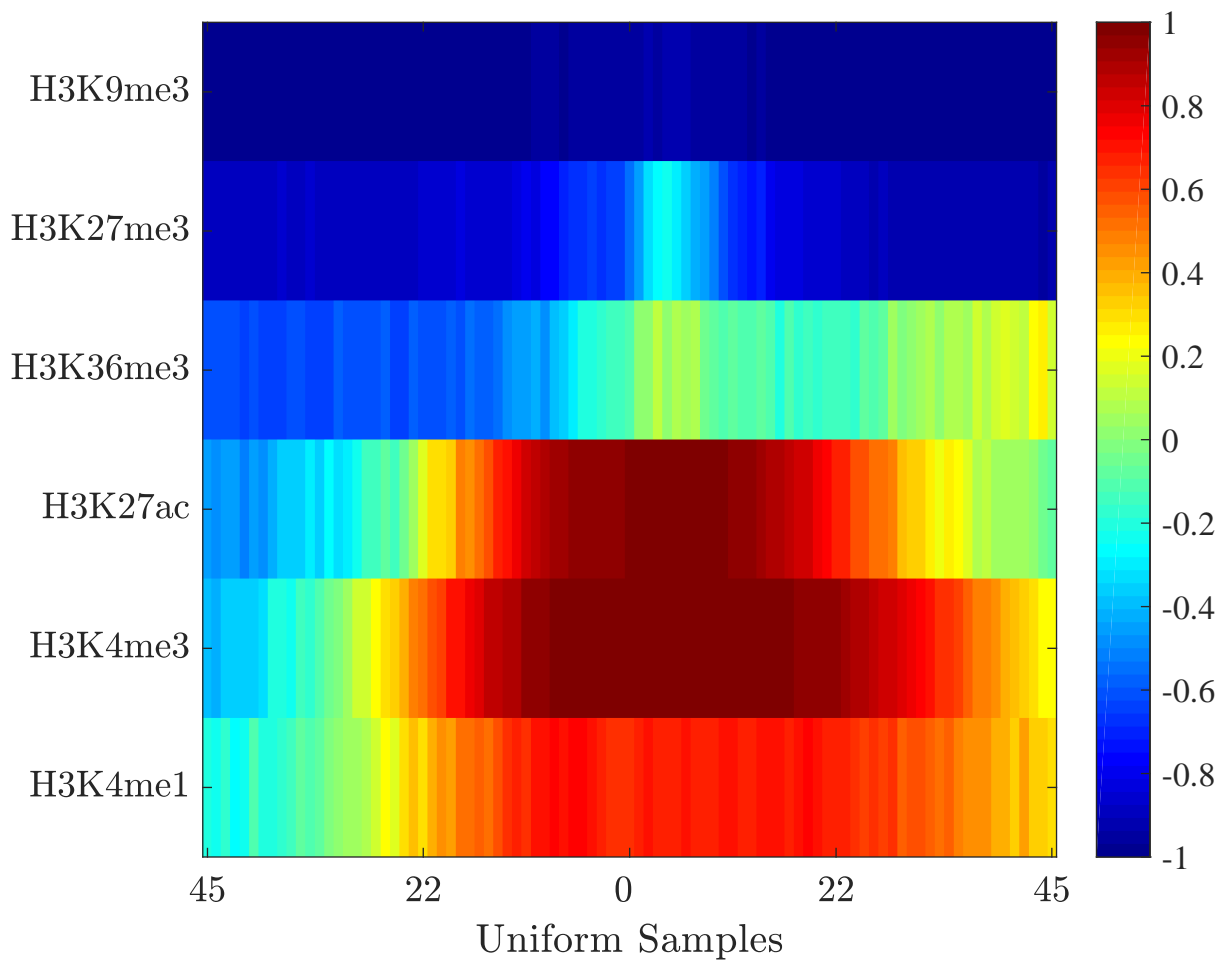

Supplement: Supplementary file 1 — HebbPlots of active promoters on the positive strand. This compressed file (.tar.gz) includes HebbPlots of promoters on the positive strand active in 57 tissues/cell types. (TAR 2949 kb) [file 12859_2018_2312_MOESM1_ESM.tar › file2/E056.pdf]

Marks

H3K27me3

H3K9me3

H3K36me3

H3K4me1

H3K4me3

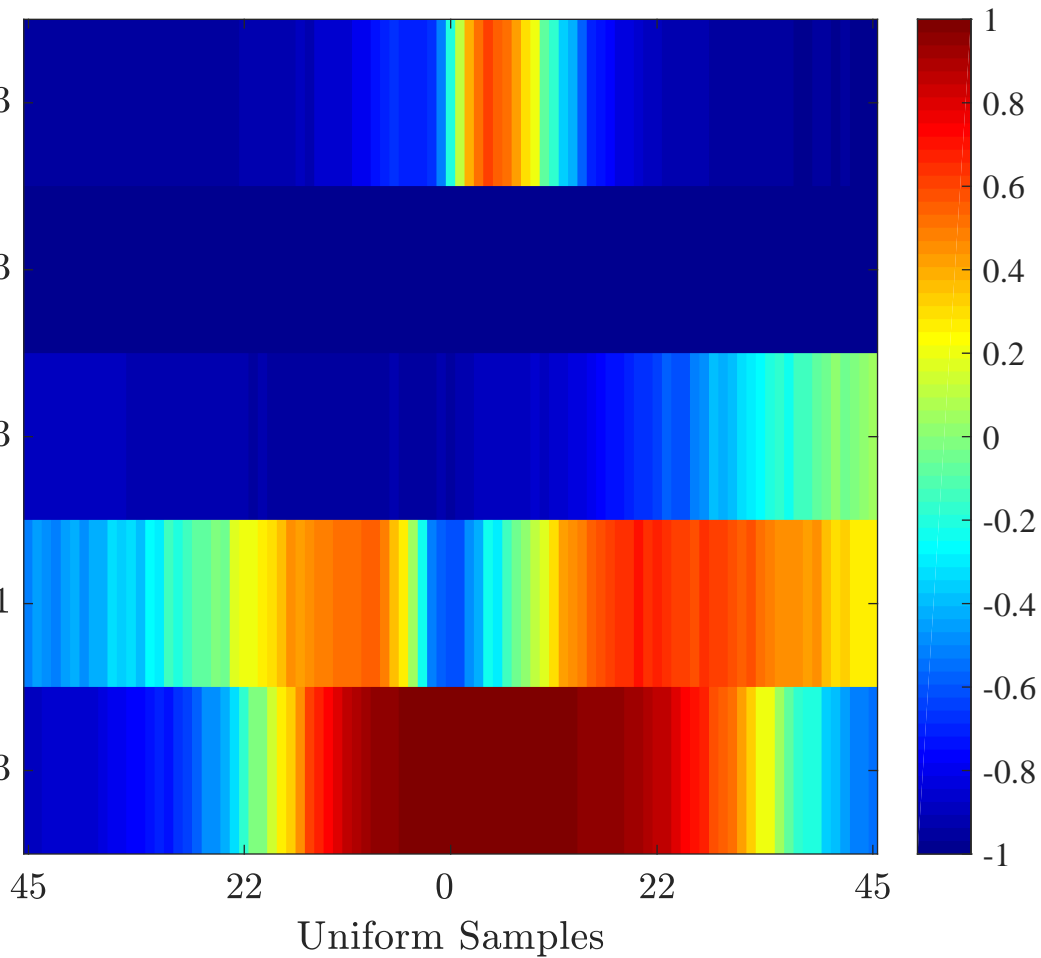

Supplement: Supplementary file 1 — HebbPlots of active promoters on the positive strand. This compressed file (.tar.gz) includes HebbPlots of promoters on the positive strand active in 57 tissues/cell types. (TAR 2949 kb) [file 12859_2018_2312_MOESM1_ESM.tar › file2/E057.pdf]

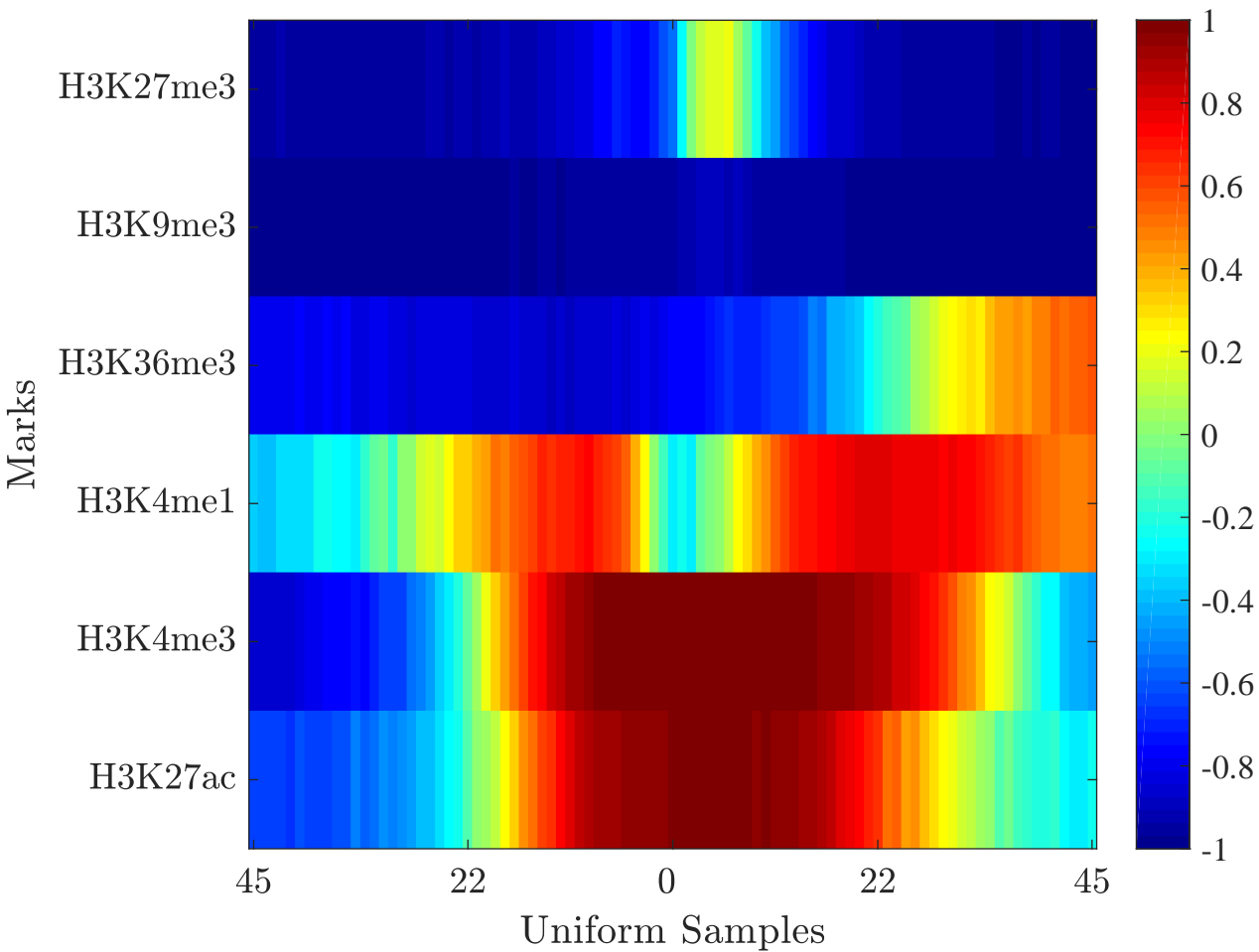

Supplement: Supplementary file 1 — HebbPlots of active promoters on the positive strand. This compressed file (.tar.gz) includes HebbPlots of promoters on the positive strand active in 57 tissues/cell types. (TAR 2949 kb) [file 12859_2018_2312_MOESM1_ESM.tar › file2/E058.pdf]

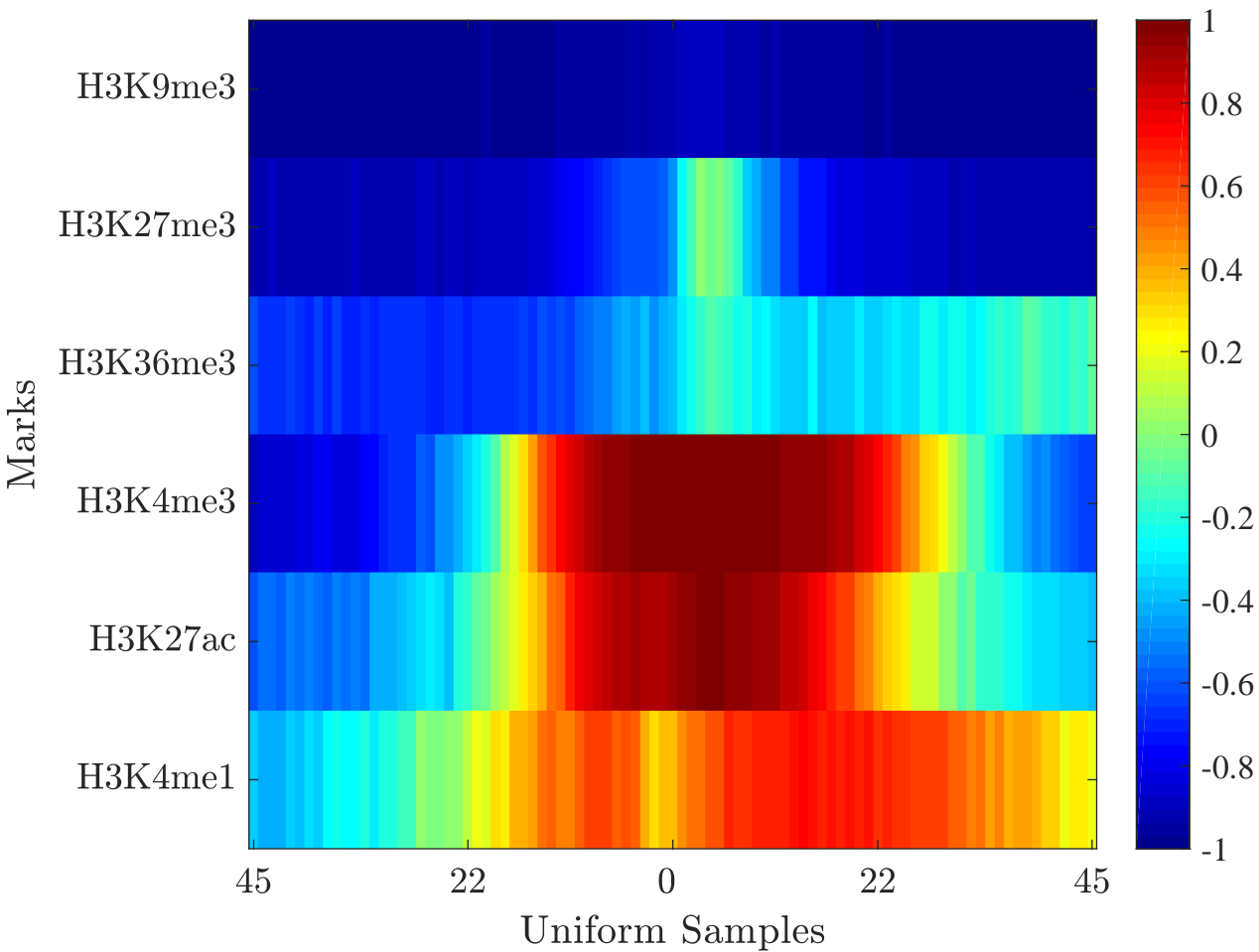

Supplement: Supplementary file 1 — HebbPlots of active promoters on the positive strand. This compressed file (.tar.gz) includes HebbPlots of promoters on the positive strand active in 57 tissues/cell types. (TAR 2949 kb) [file 12859_2018_2312_MOESM1_ESM.tar › file2/E059.pdf]

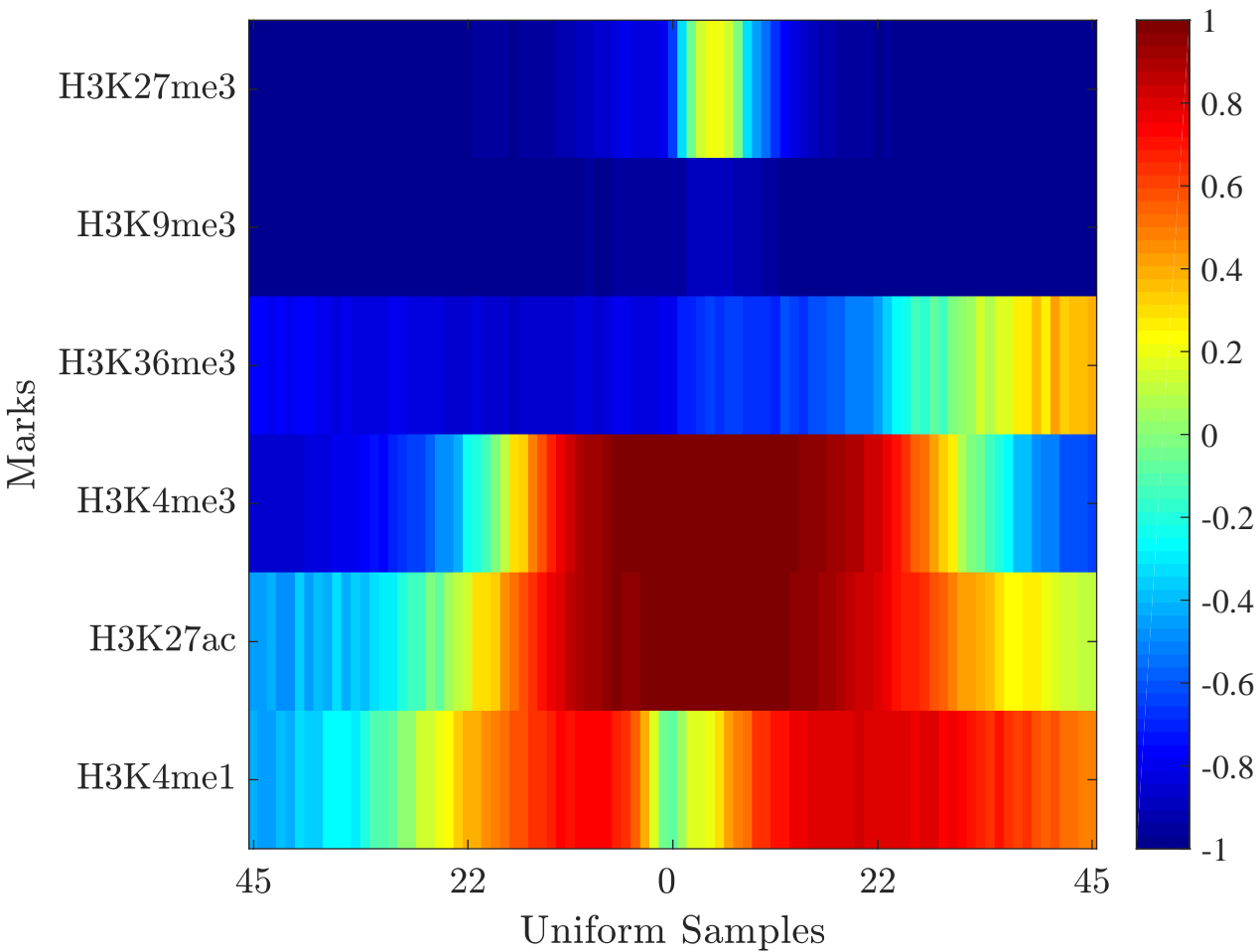

Supplement: Supplementary file 1 — HebbPlots of active promoters on the positive strand. This compressed file (.tar.gz) includes HebbPlots of promoters on the positive strand active in 57 tissues/cell types. (TAR 2949 kb) [file 12859_2018_2312_MOESM1_ESM.tar › file2/E061.pdf]

Marks

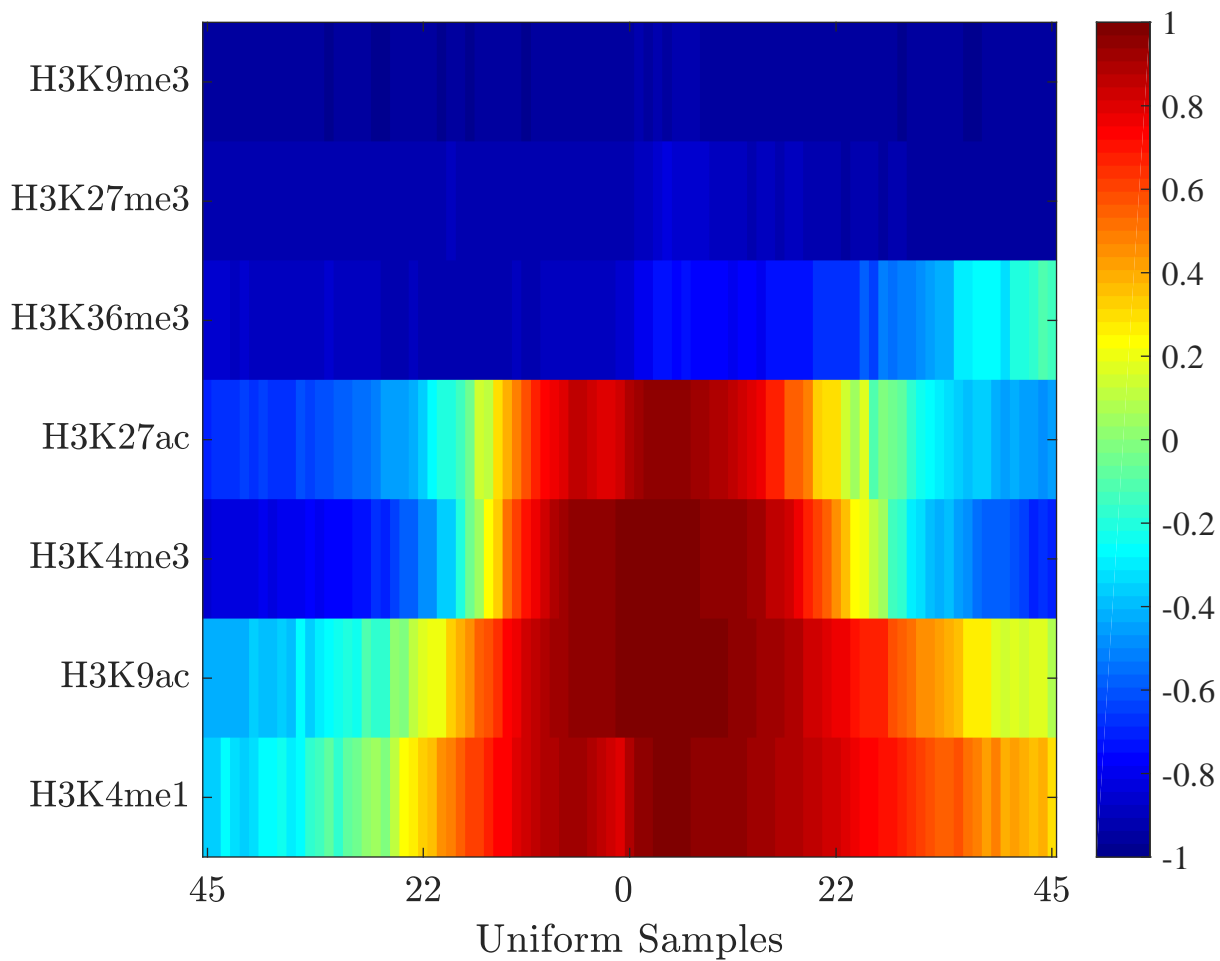

Supplement: Supplementary file 1 — HebbPlots of active promoters on the positive strand. This compressed file (.tar.gz) includes HebbPlots of promoters on the positive strand active in 57 tissues/cell types. (TAR 2949 kb) [file 12859_2018_2312_MOESM1_ESM.tar › file2/E062.pdf]

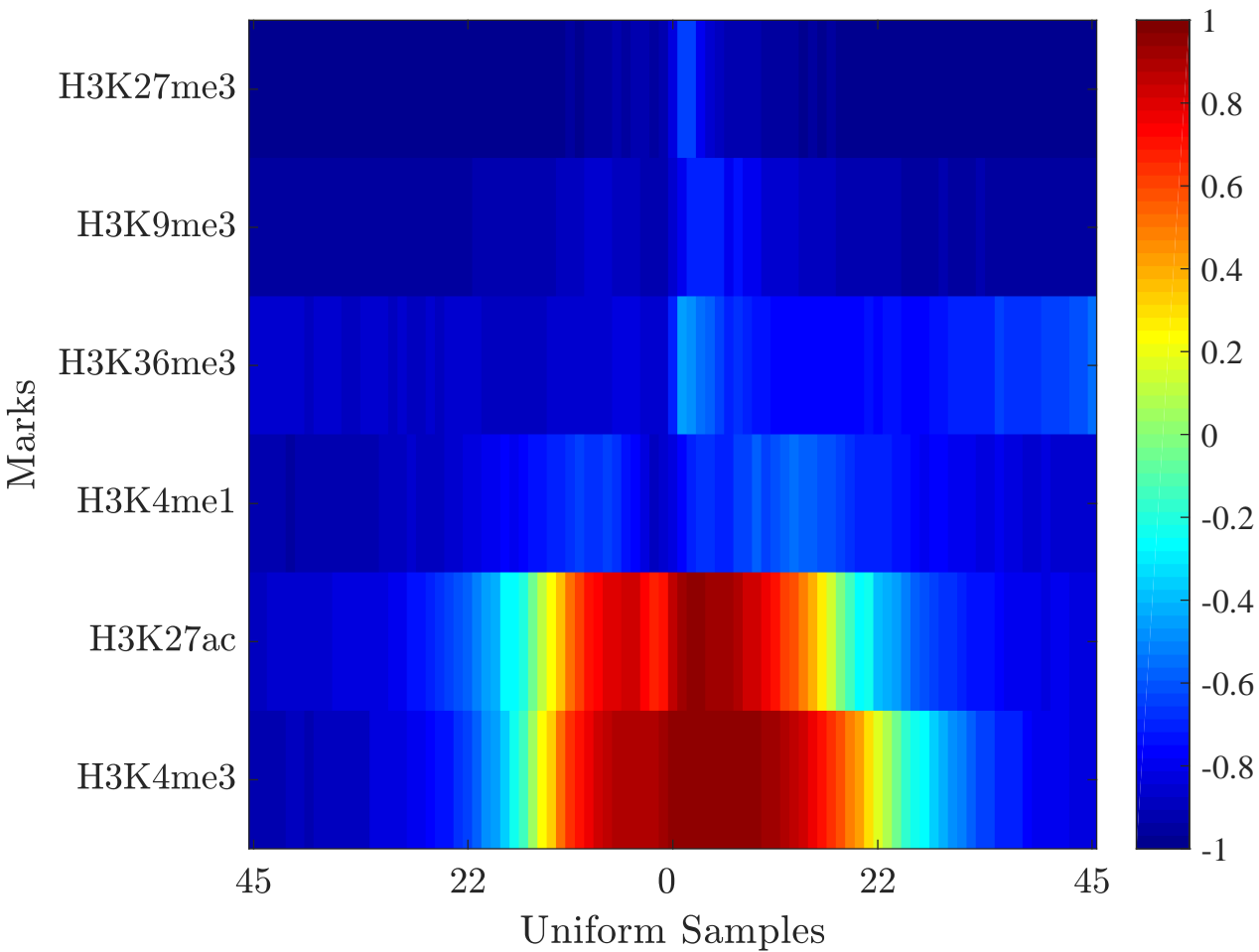

Supplement: Supplementary file 1 — HebbPlots of active promoters on the positive strand. This compressed file (.tar.gz) includes HebbPlots of promoters on the positive strand active in 57 tissues/cell types. (TAR 2949 kb) [file 12859_2018_2312_MOESM1_ESM.tar › file2/E065.pdf]

Marks

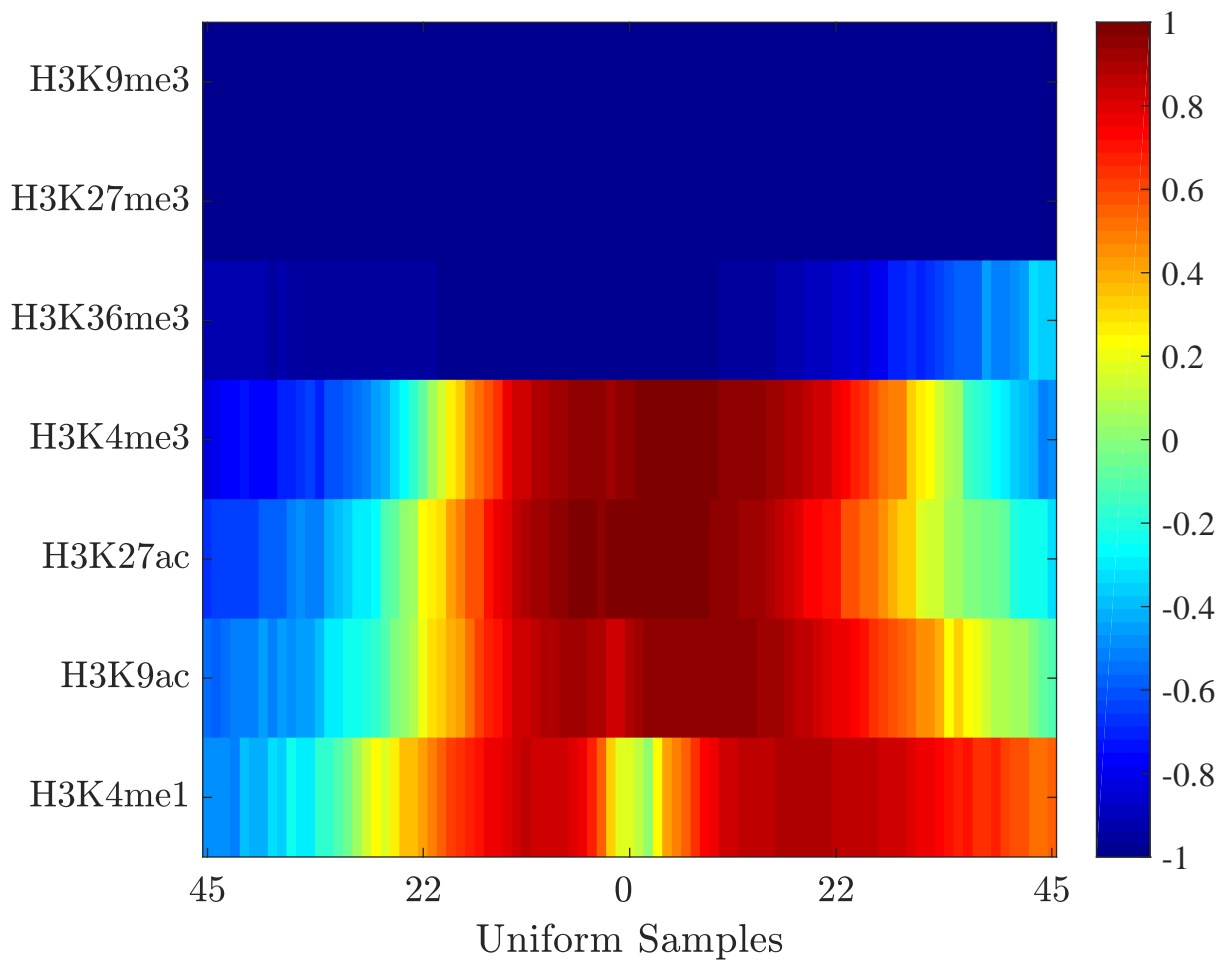

Supplement: Supplementary file 1 — HebbPlots of active promoters on the positive strand. This compressed file (.tar.gz) includes HebbPlots of promoters on the positive strand active in 57 tissues/cell types. (TAR 2949 kb) [file 12859_2018_2312_MOESM1_ESM.tar › file2/E066.pdf]

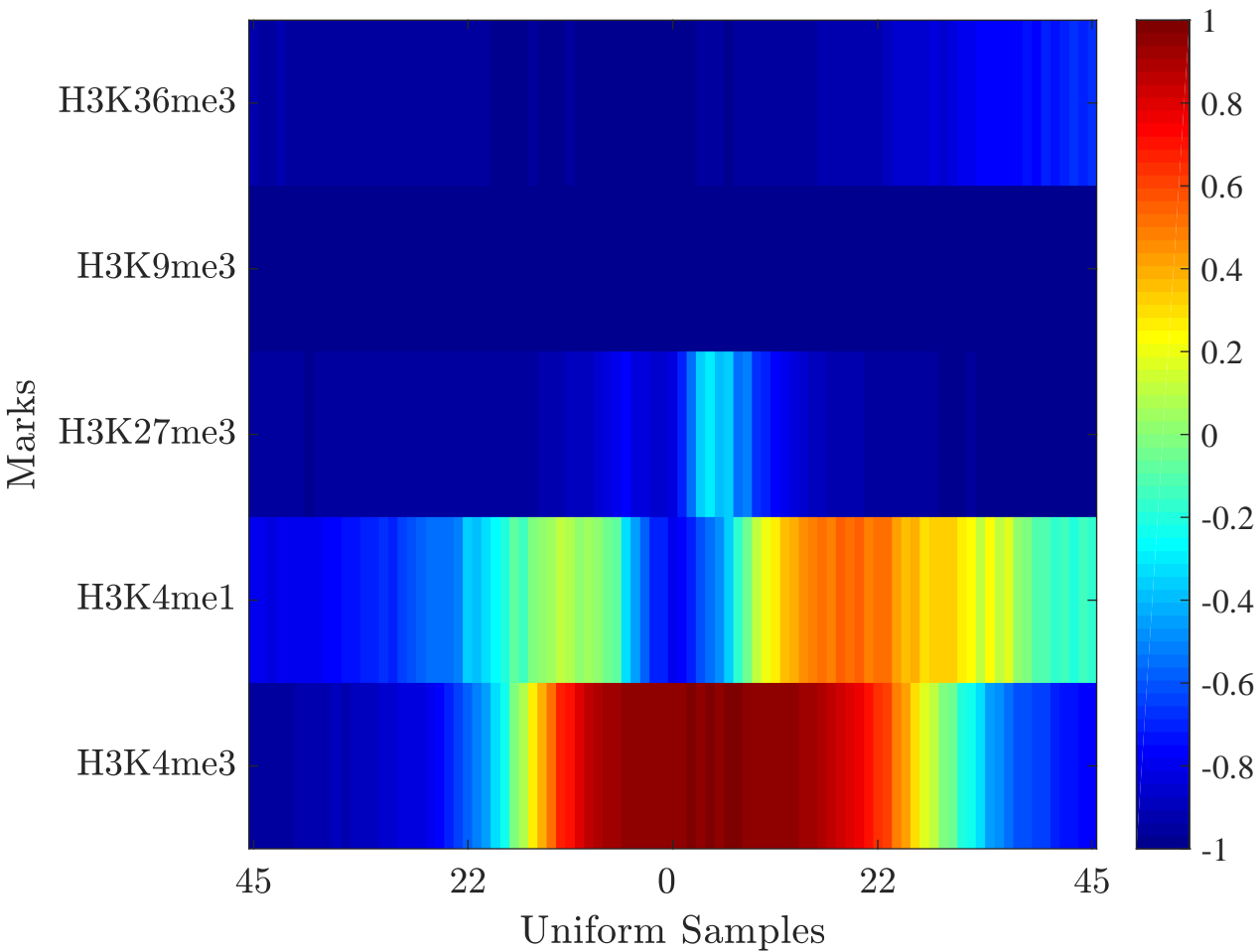

Supplement: Supplementary file 1 — HebbPlots of active promoters on the positive strand. This compressed file (.tar.gz) includes HebbPlots of promoters on the positive strand active in 57 tissues/cell types. (TAR 2949 kb) [file 12859_2018_2312_MOESM1_ESM.tar › file2/E070.pdf]

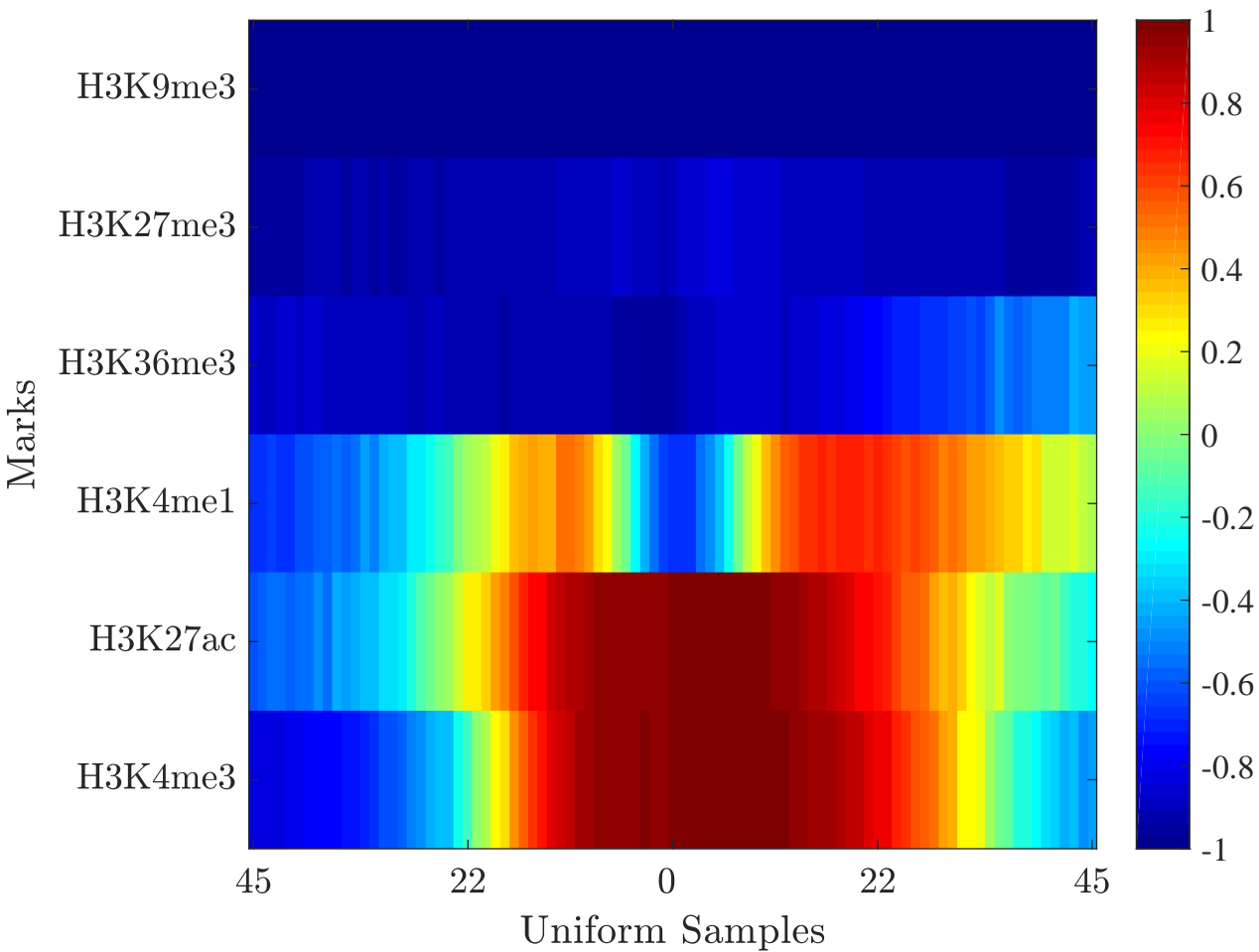

Supplement: Supplementary file 1 — HebbPlots of active promoters on the positive strand. This compressed file (.tar.gz) includes HebbPlots of promoters on the positive strand active in 57 tissues/cell types. (TAR 2949 kb) [file 12859_2018_2312_MOESM1_ESM.tar › file2/E071.pdf]

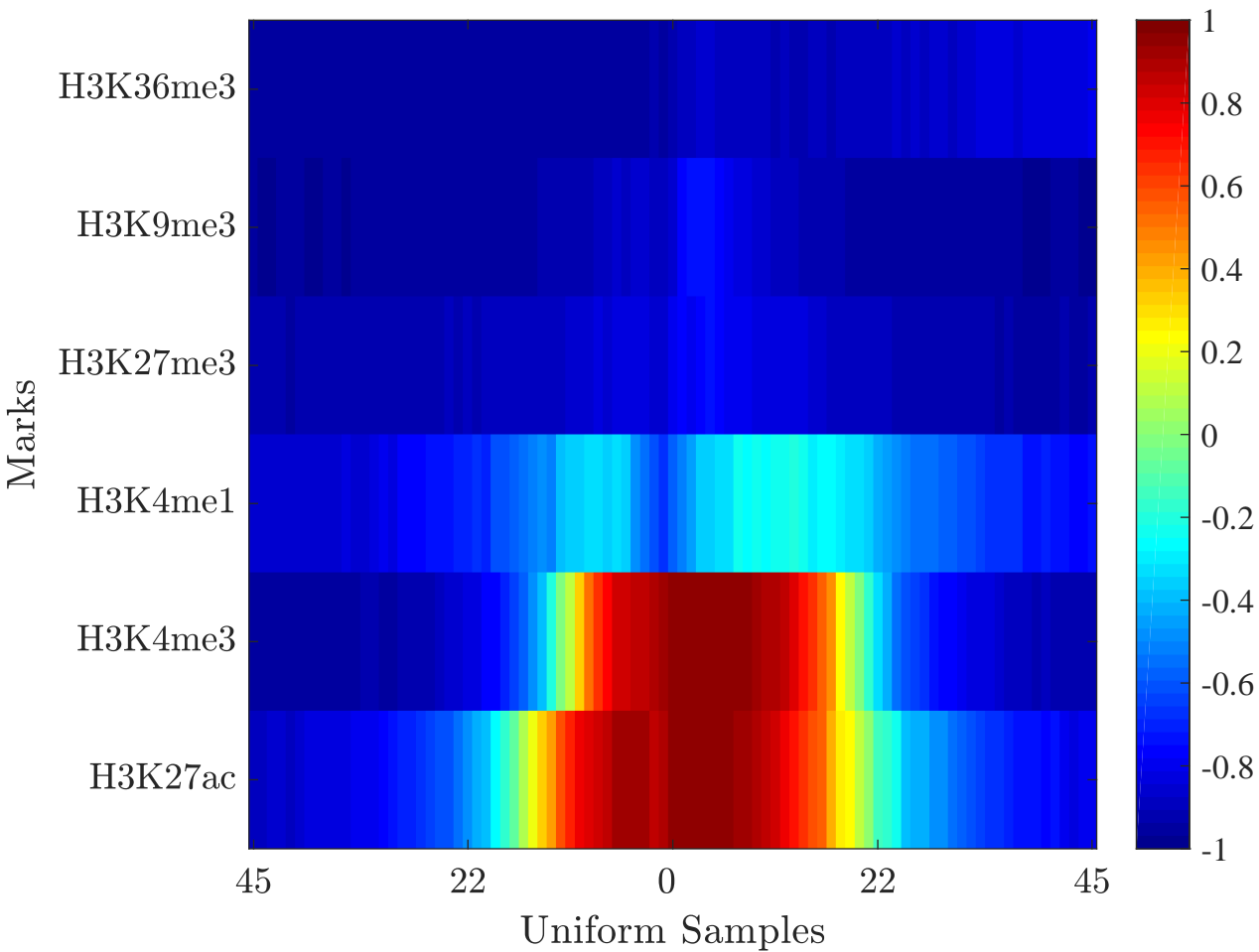

Supplement: Supplementary file 1 — HebbPlots of active promoters on the positive strand. This compressed file (.tar.gz) includes HebbPlots of promoters on the positive strand active in 57 tissues/cell types. (TAR 2949 kb) [file 12859_2018_2312_MOESM1_ESM.tar › file2/E079.pdf]

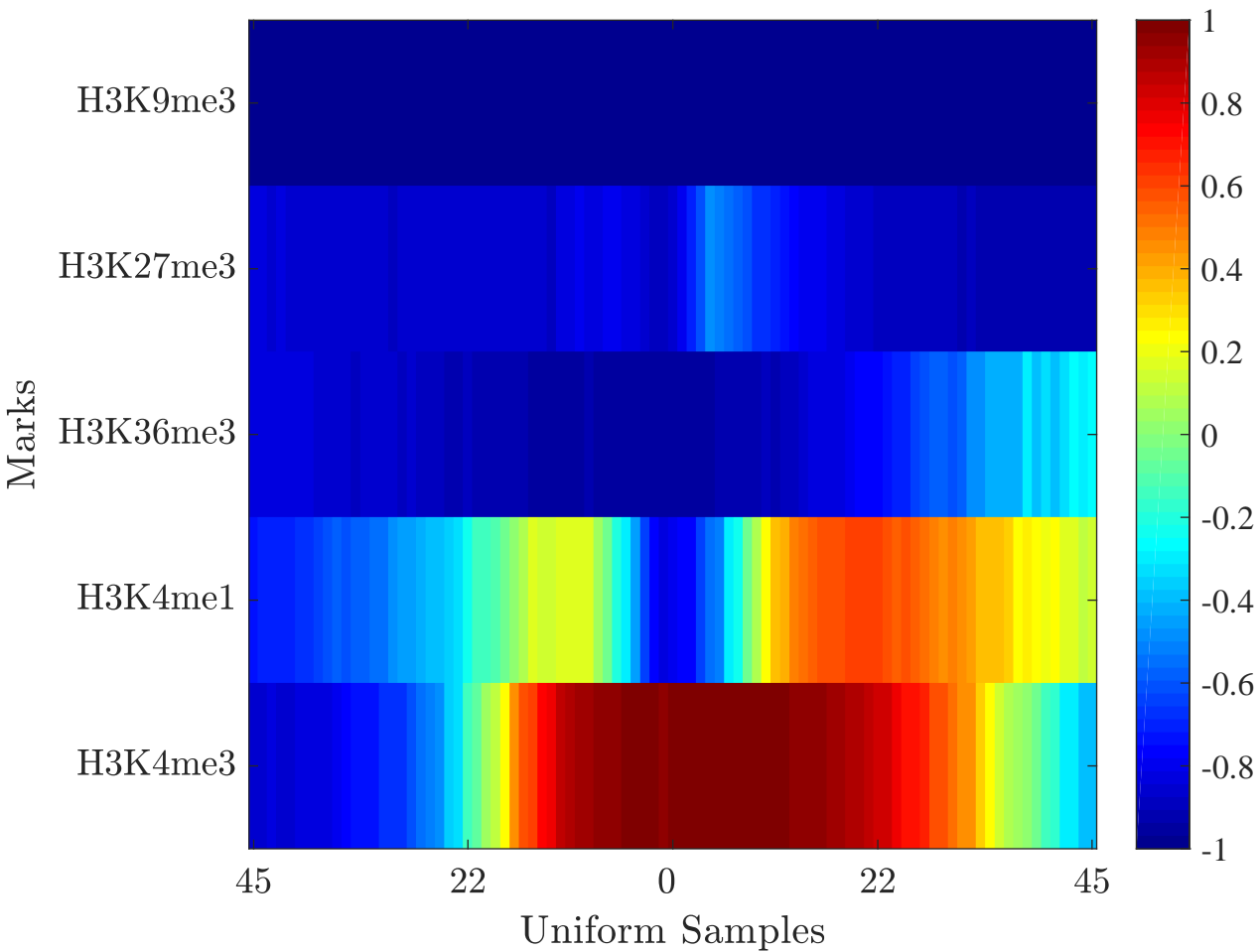

Supplement: Supplementary file 1 — HebbPlots of active promoters on the positive strand. This compressed file (.tar.gz) includes HebbPlots of promoters on the positive strand active in 57 tissues/cell types. (TAR 2949 kb) [file 12859_2018_2312_MOESM1_ESM.tar › file2/E082.pdf]

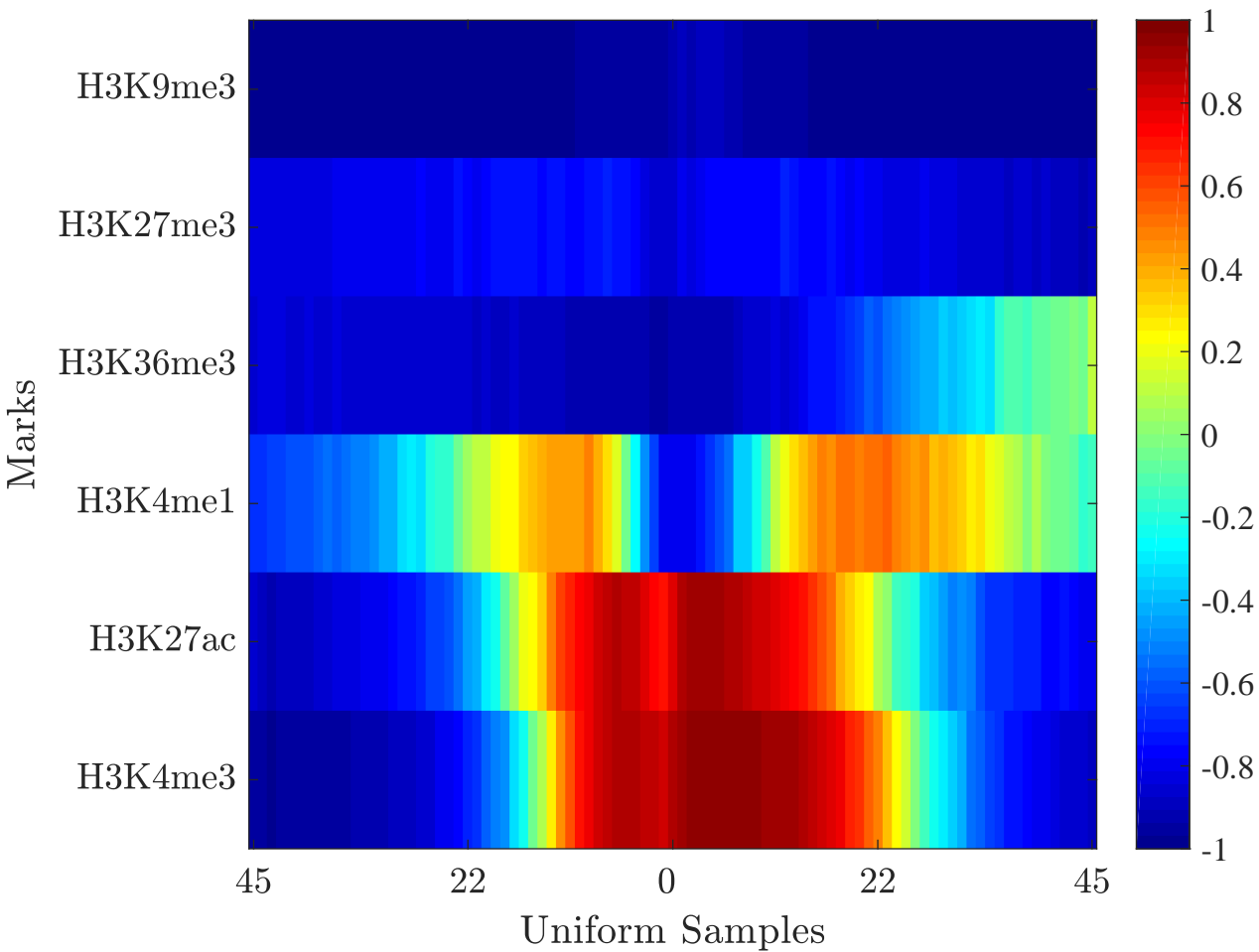

Supplement: Supplementary file 1 — HebbPlots of active promoters on the positive strand. This compressed file (.tar.gz) includes HebbPlots of promoters on the positive strand active in 57 tissues/cell types. (TAR 2949 kb) [file 12859_2018_2312_MOESM1_ESM.tar › file2/E084.pdf]

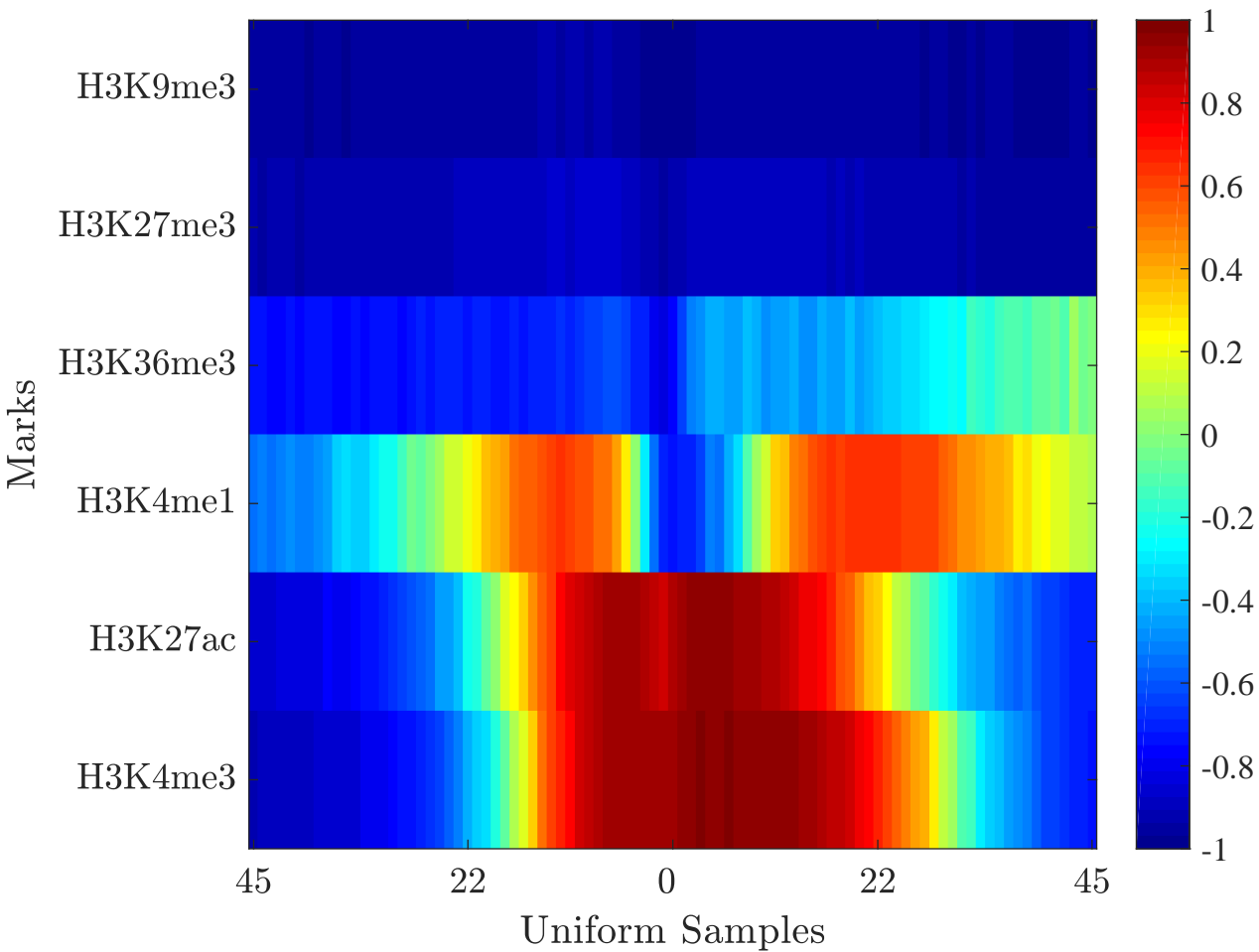

Supplement: Supplementary file 1 — HebbPlots of active promoters on the positive strand. This compressed file (.tar.gz) includes HebbPlots of promoters on the positive strand active in 57 tissues/cell types. (TAR 2949 kb) [file 12859_2018_2312_MOESM1_ESM.tar › file2/E085.pdf]

Marks

H3K36me3

H3K27me3

H3K9me3

H3K4me1

H3K9ac

H3K27ac

H3K4me3

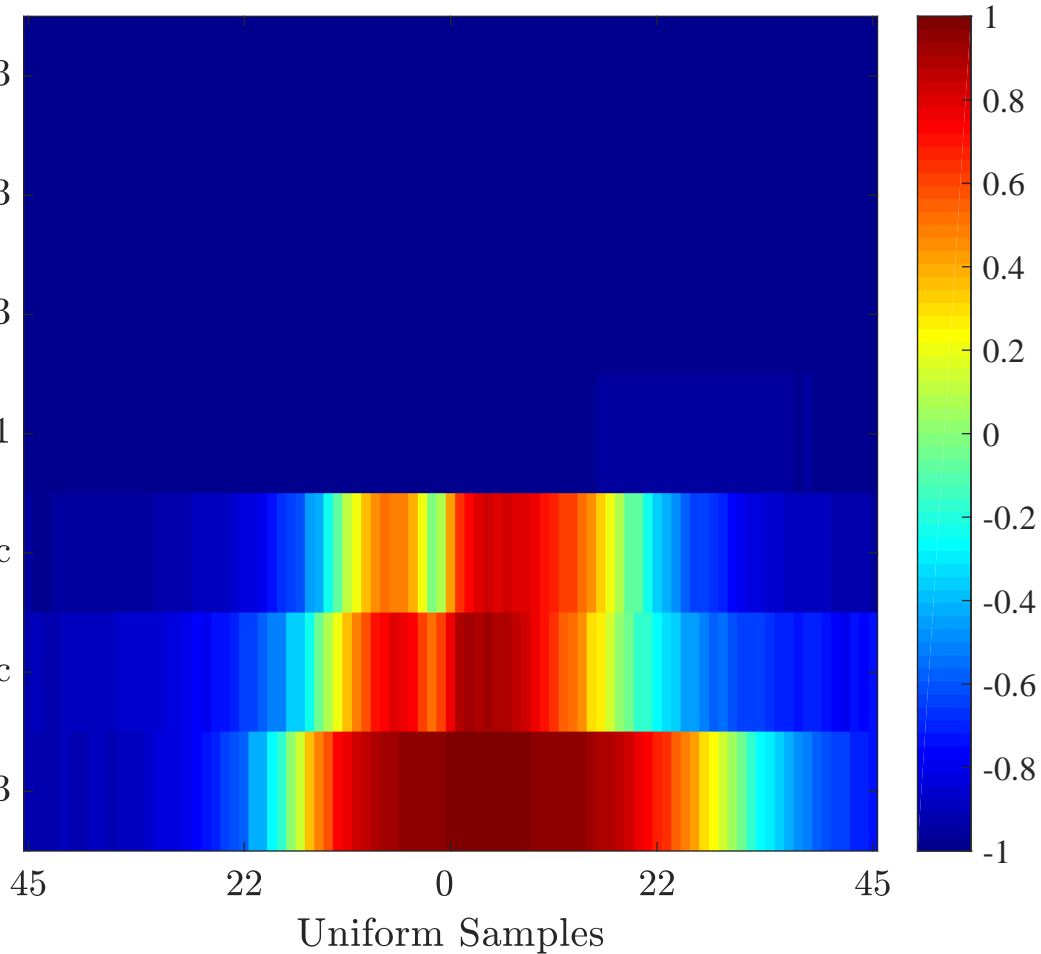

Supplement: Supplementary file 1 — HebbPlots of active promoters on the positive strand. This compressed file (.tar.gz) includes HebbPlots of promoters on the positive strand active in 57 tissues/cell types. (TAR 2949 kb) [file 12859_2018_2312_MOESM1_ESM.tar › file2/E087.pdf]

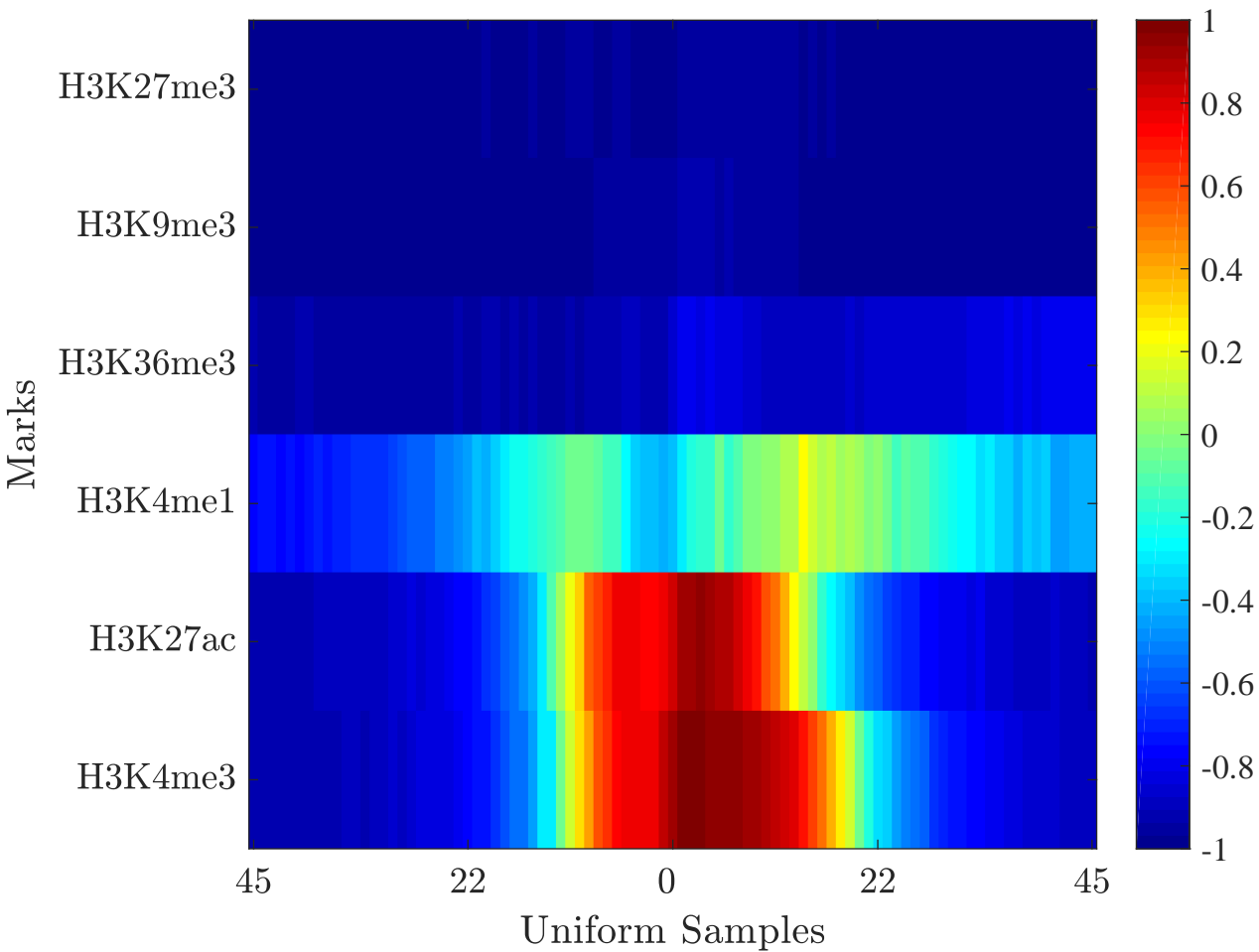

Supplement: Supplementary file 1 — HebbPlots of active promoters on the positive strand. This compressed file (.tar.gz) includes HebbPlots of promoters on the positive strand active in 57 tissues/cell types. (TAR 2949 kb) [file 12859_2018_2312_MOESM1_ESM.tar › file2/E094.pdf]

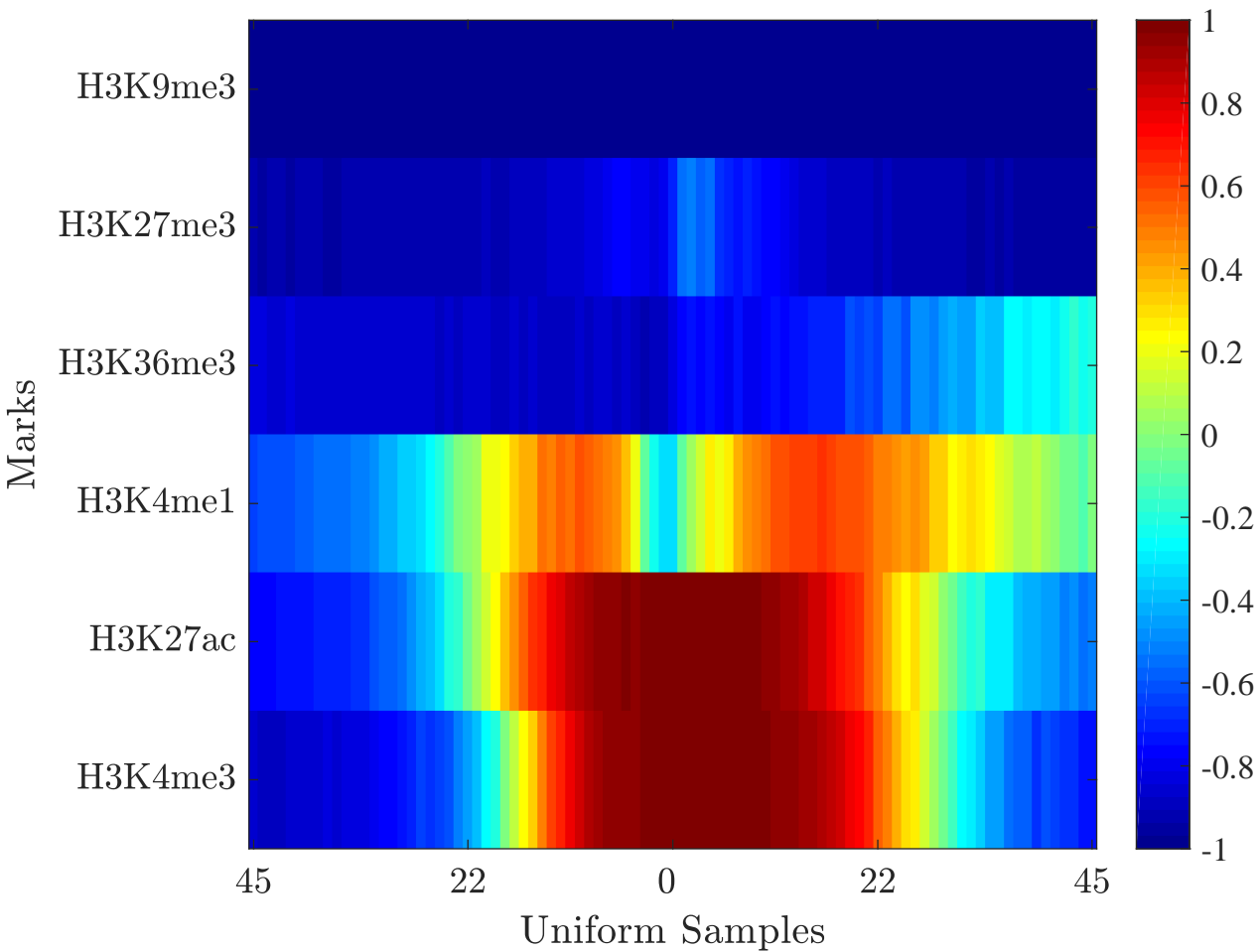

Supplement: Supplementary file 1 — HebbPlots of active promoters on the positive strand. This compressed file (.tar.gz) includes HebbPlots of promoters on the positive strand active in 57 tissues/cell types. (TAR 2949 kb) [file 12859_2018_2312_MOESM1_ESM.tar › file2/E095.pdf]

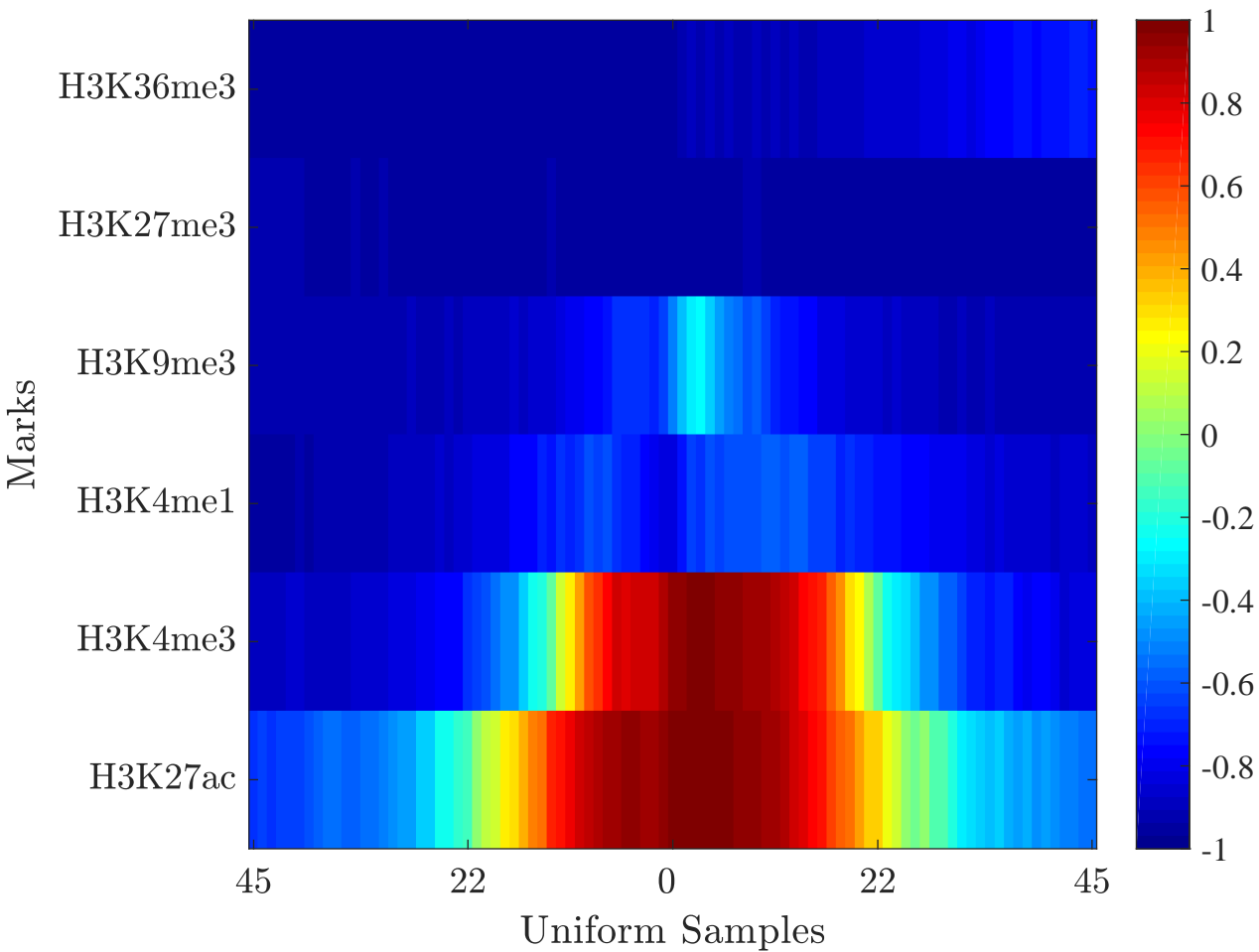

Supplement: Supplementary file 1 — HebbPlots of active promoters on the positive strand. This compressed file (.tar.gz) includes HebbPlots of promoters on the positive strand active in 57 tissues/cell types. (TAR 2949 kb) [file 12859_2018_2312_MOESM1_ESM.tar › file2/E096.pdf]

Marks

H3K27me3

H3K36me3

H3K9me3

H3K27ac

H3K4me3

H3K4me1

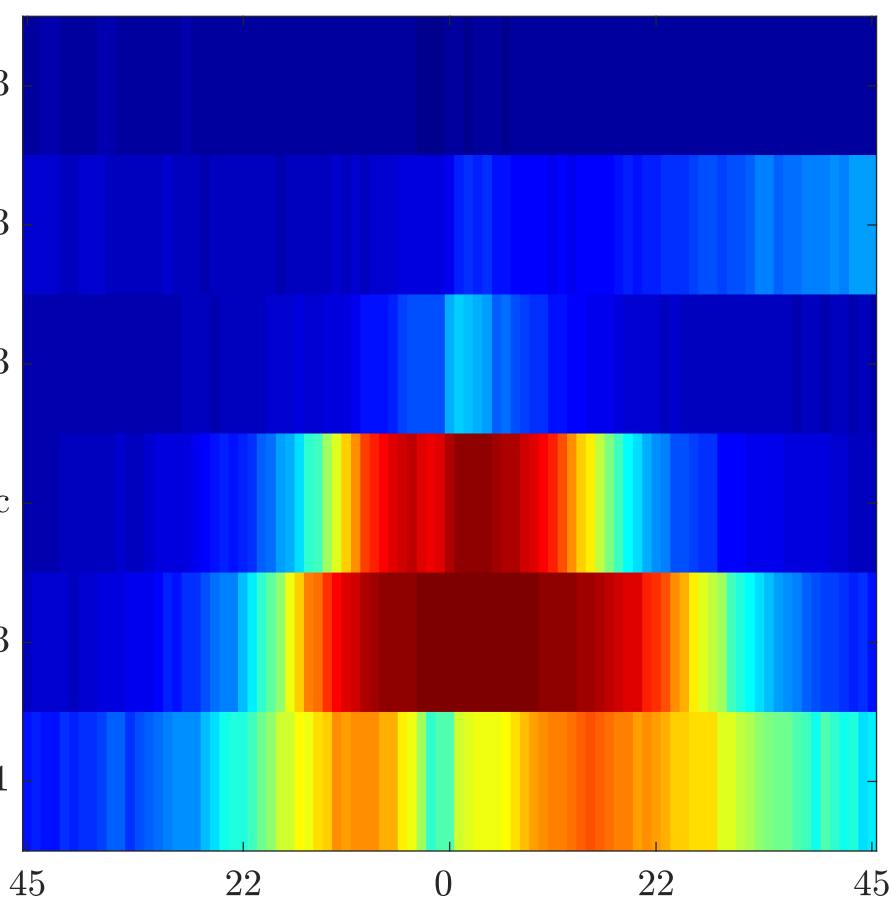

Uniform Samples

Supplement: Supplementary file 1 — HebbPlots of active promoters on the positive strand. This compressed file (.tar.gz) includes HebbPlots of promoters on the positive strand active in 57 tissues/cell types. (TAR 2949 kb) [file 12859_2018_2312_MOESM1_ESM.tar › file2/E097.pdf]

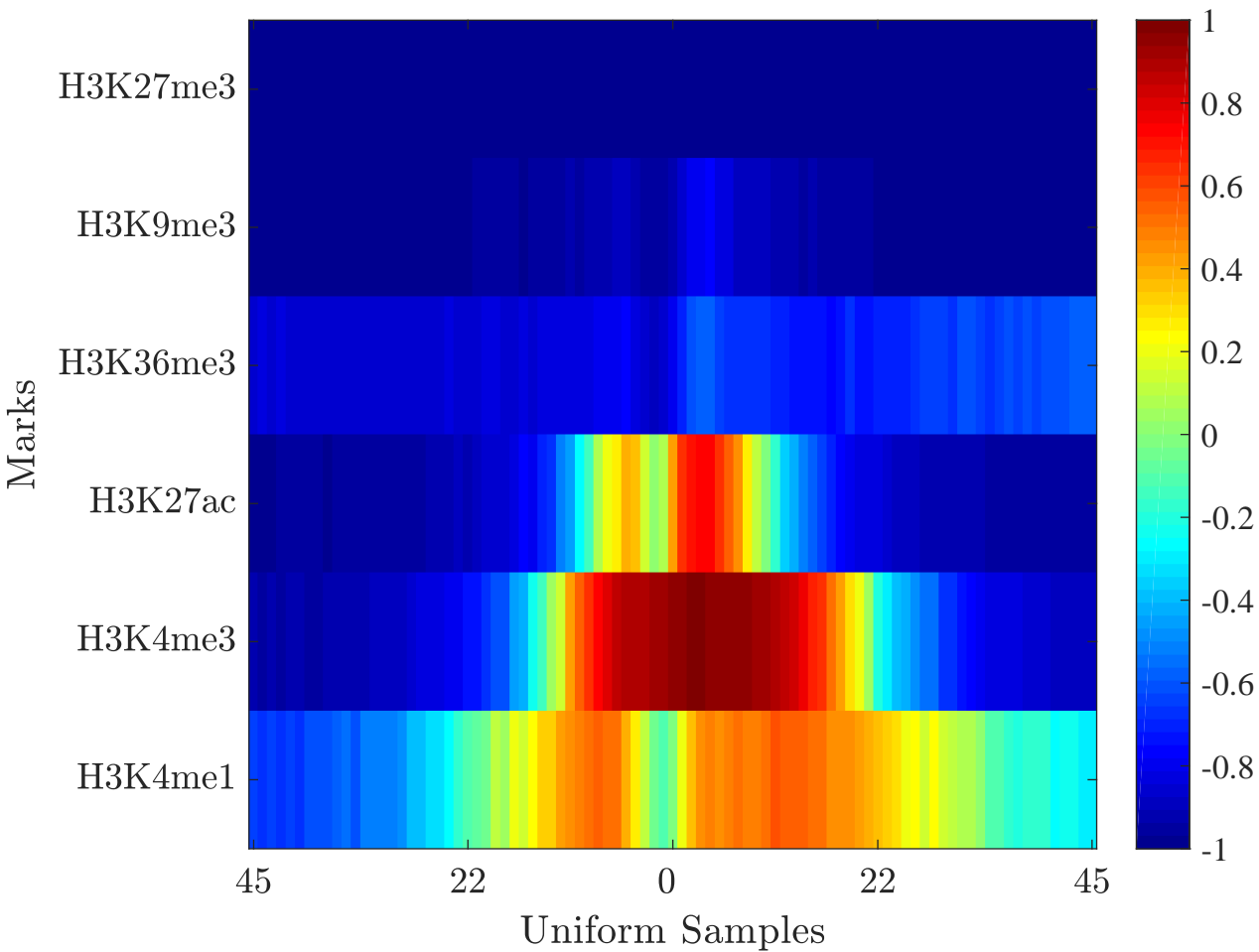

Supplement: Supplementary file 1 — HebbPlots of active promoters on the positive strand. This compressed file (.tar.gz) includes HebbPlots of promoters on the positive strand active in 57 tissues/cell types. (TAR 2949 kb) [file 12859_2018_2312_MOESM1_ESM.tar › file2/E098.pdf]

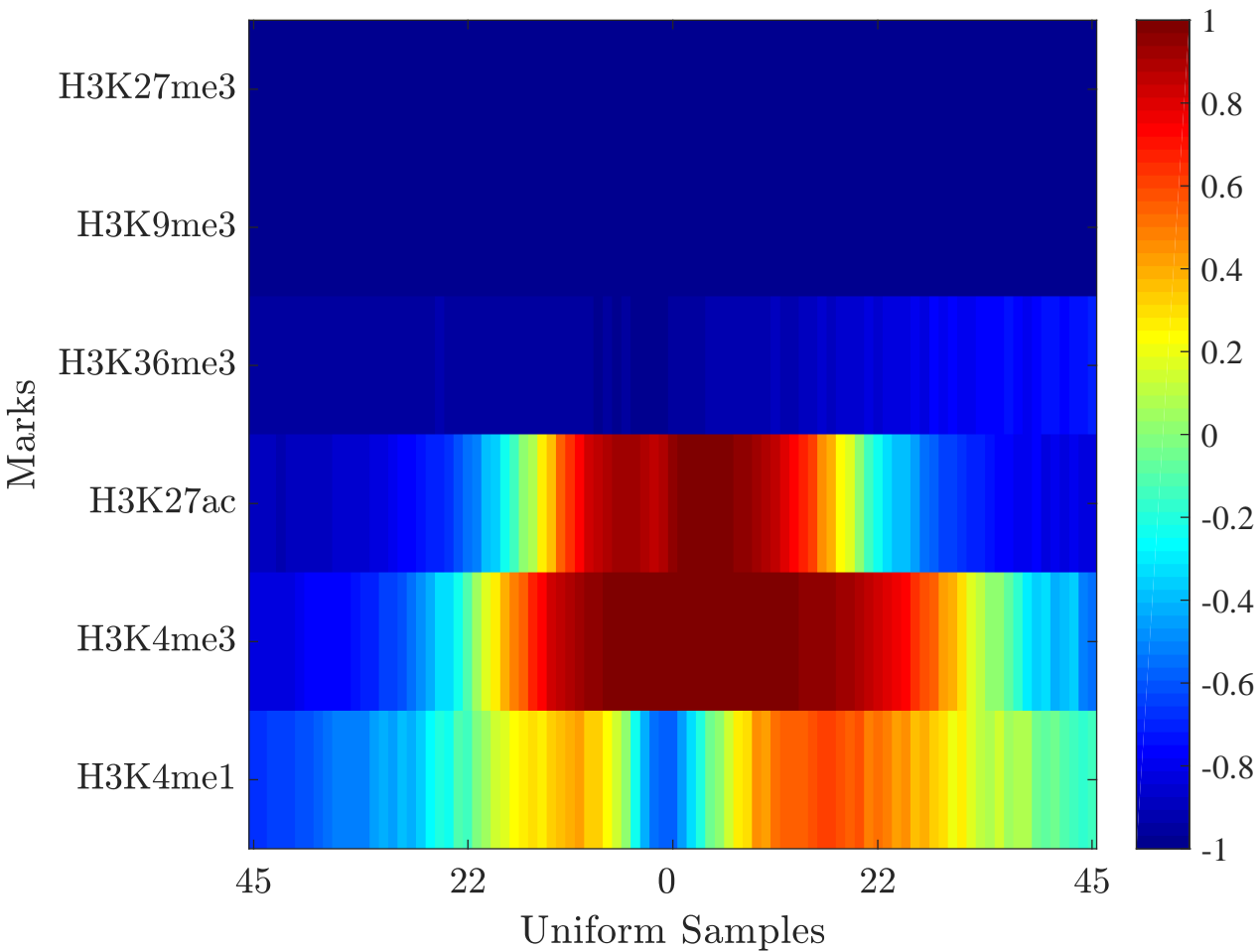

Supplement: Supplementary file 1 — HebbPlots of active promoters on the positive strand. This compressed file (.tar.gz) includes HebbPlots of promoters on the positive strand active in 57 tissues/cell types. (TAR 2949 kb) [file 12859_2018_2312_MOESM1_ESM.tar › file2/E100.pdf]

Marks

H3K27me3

H3K36me3

H3K9me3

H3K4me1

H3K27ac

H3K4me3

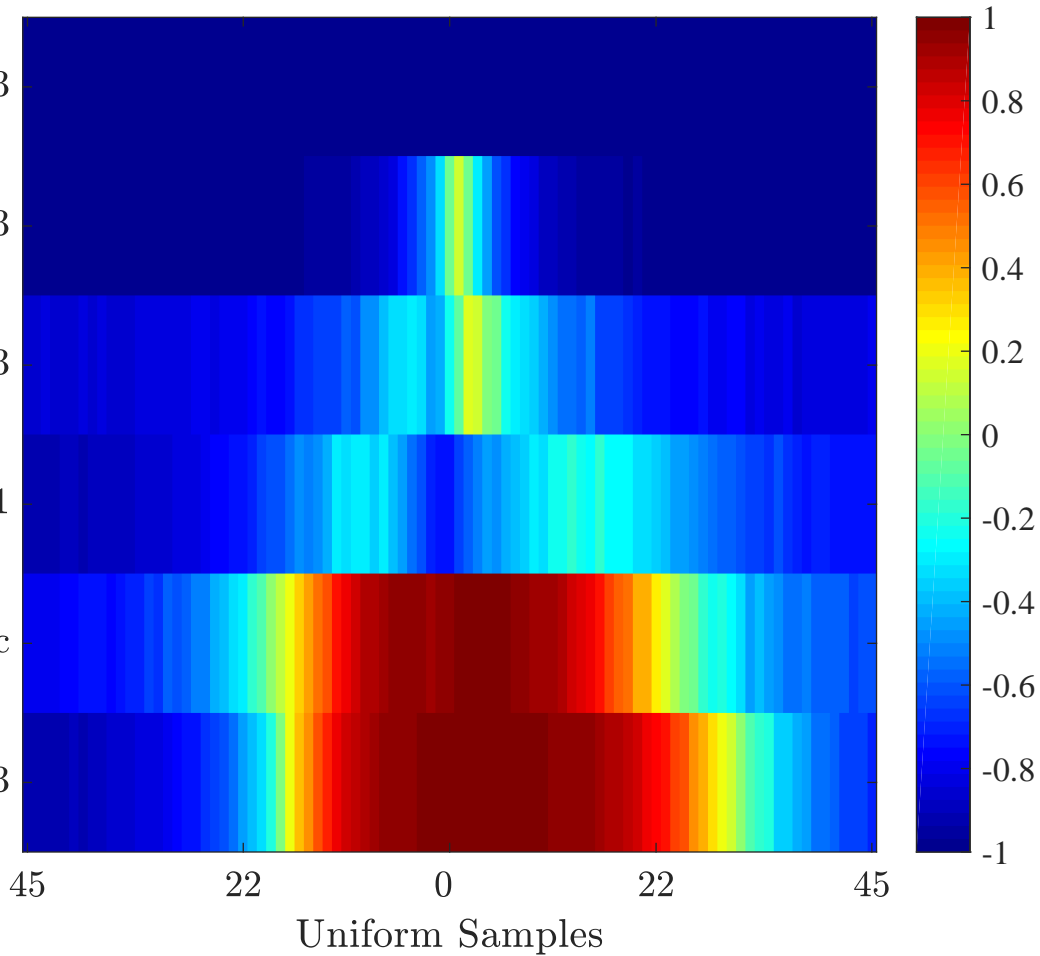

Supplement: Supplementary file 1 — HebbPlots of active promoters on the positive strand. This compressed file (.tar.gz) includes HebbPlots of promoters on the positive strand active in 57 tissues/cell types. (TAR 2949 kb) [file 12859_2018_2312_MOESM1_ESM.tar › file2/E104.pdf]

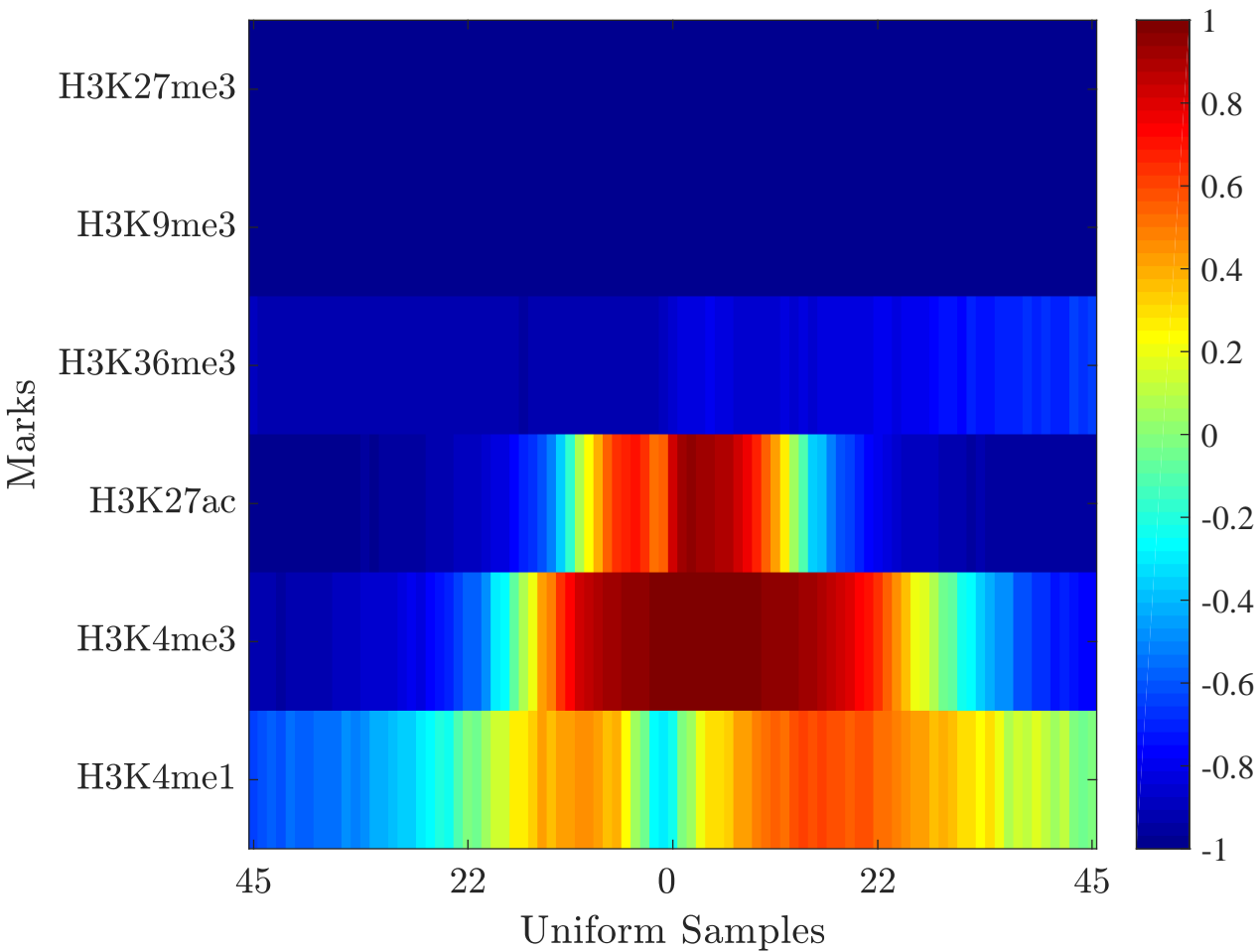

Supplement: Supplementary file 1 — HebbPlots of active promoters on the positive strand. This compressed file (.tar.gz) includes HebbPlots of promoters on the positive strand active in 57 tissues/cell types. (TAR 2949 kb) [file 12859_2018_2312_MOESM1_ESM.tar › file2/E105.pdf]

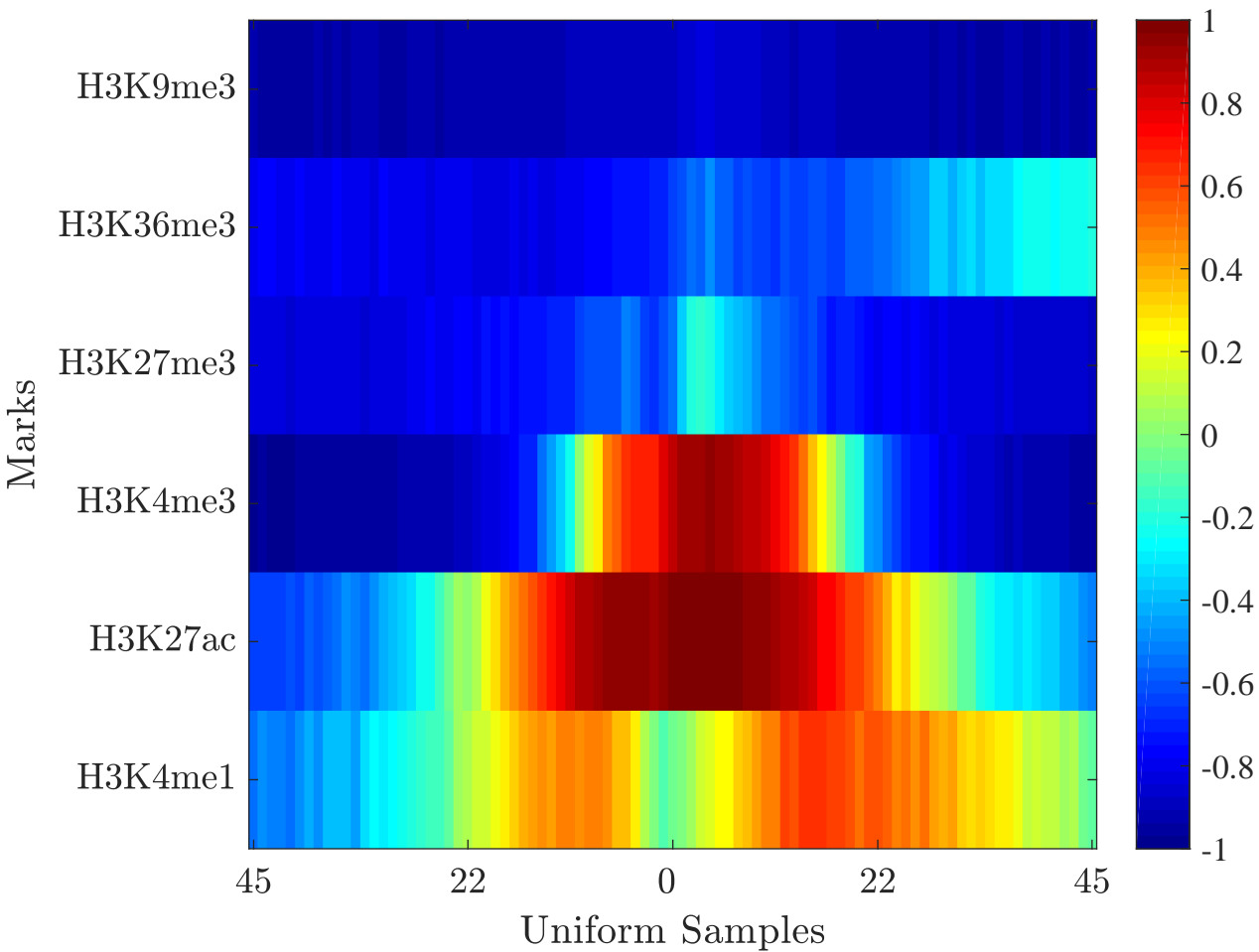

Supplement: Supplementary file 1 — HebbPlots of active promoters on the positive strand. This compressed file (.tar.gz) includes HebbPlots of promoters on the positive strand active in 57 tissues/cell types. (TAR 2949 kb) [file 12859_2018_2312_MOESM1_ESM.tar › file2/E106.pdf]

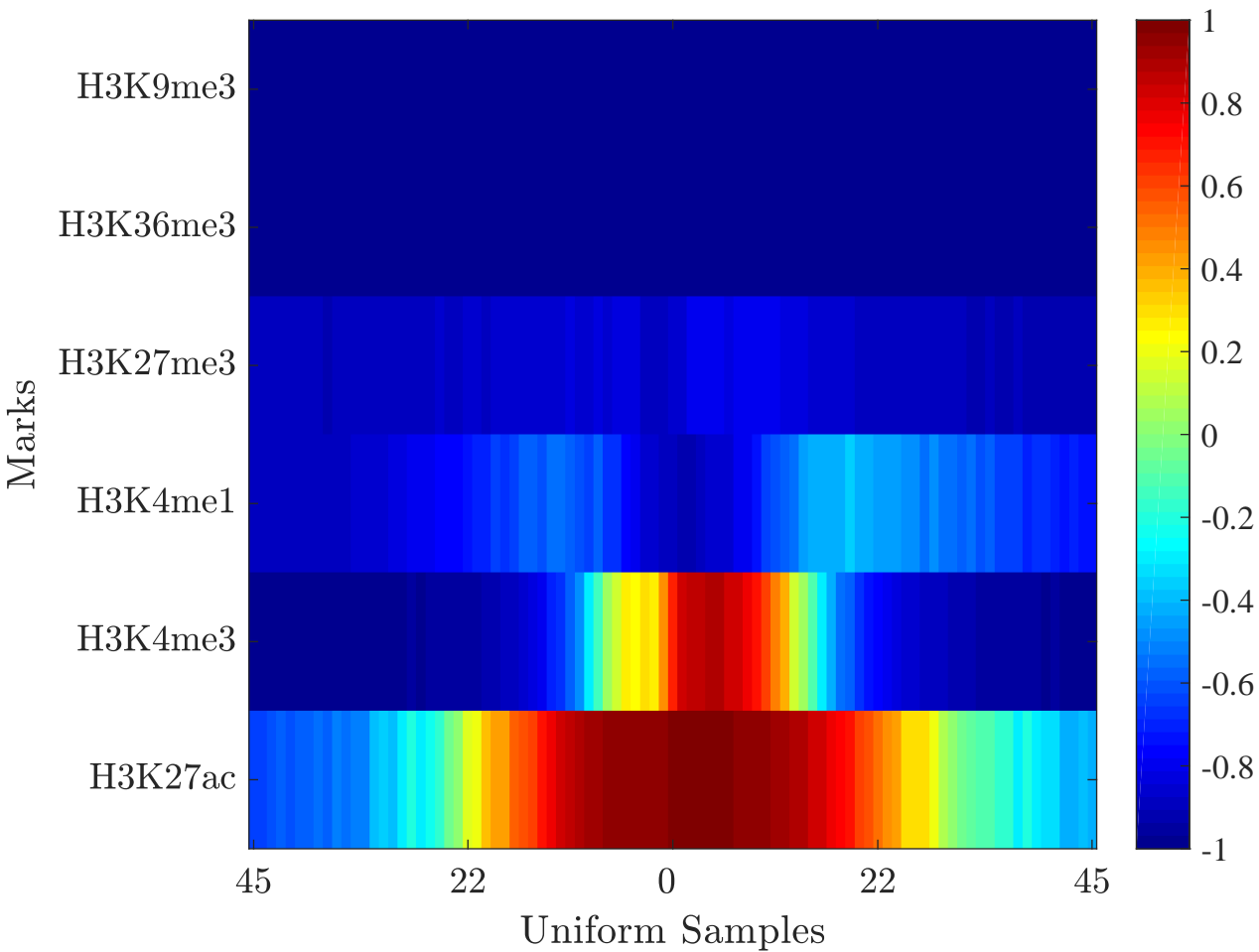

Supplement: Supplementary file 1 — HebbPlots of active promoters on the positive strand. This compressed file (.tar.gz) includes HebbPlots of promoters on the positive strand active in 57 tissues/cell types. (TAR 2949 kb) [file 12859_2018_2312_MOESM1_ESM.tar › file2/E109.pdf]

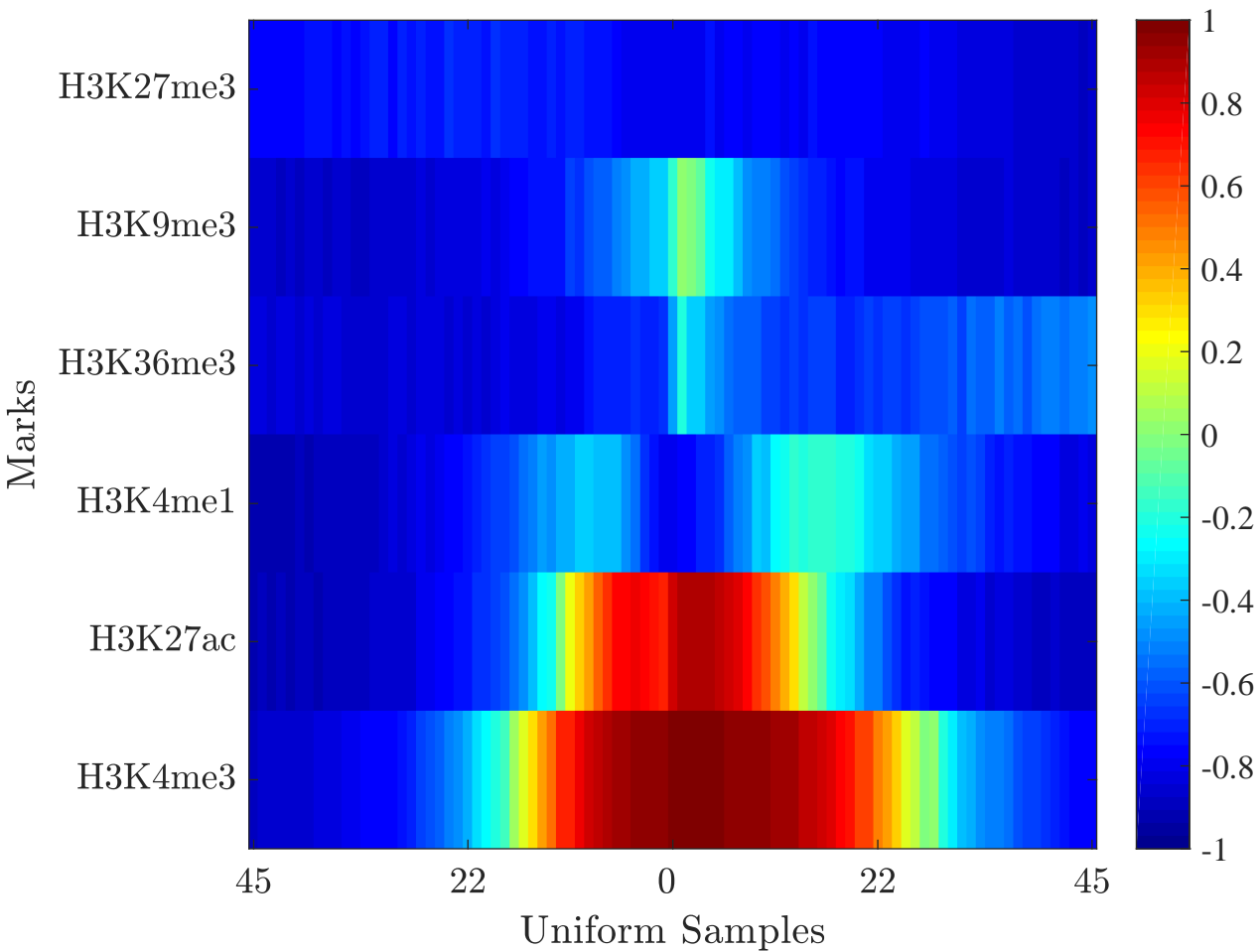

Supplement: Supplementary file 1 — HebbPlots of active promoters on the positive strand. This compressed file (.tar.gz) includes HebbPlots of promoters on the positive strand active in 57 tissues/cell types. (TAR 2949 kb) [file 12859_2018_2312_MOESM1_ESM.tar › file2/E112.pdf]

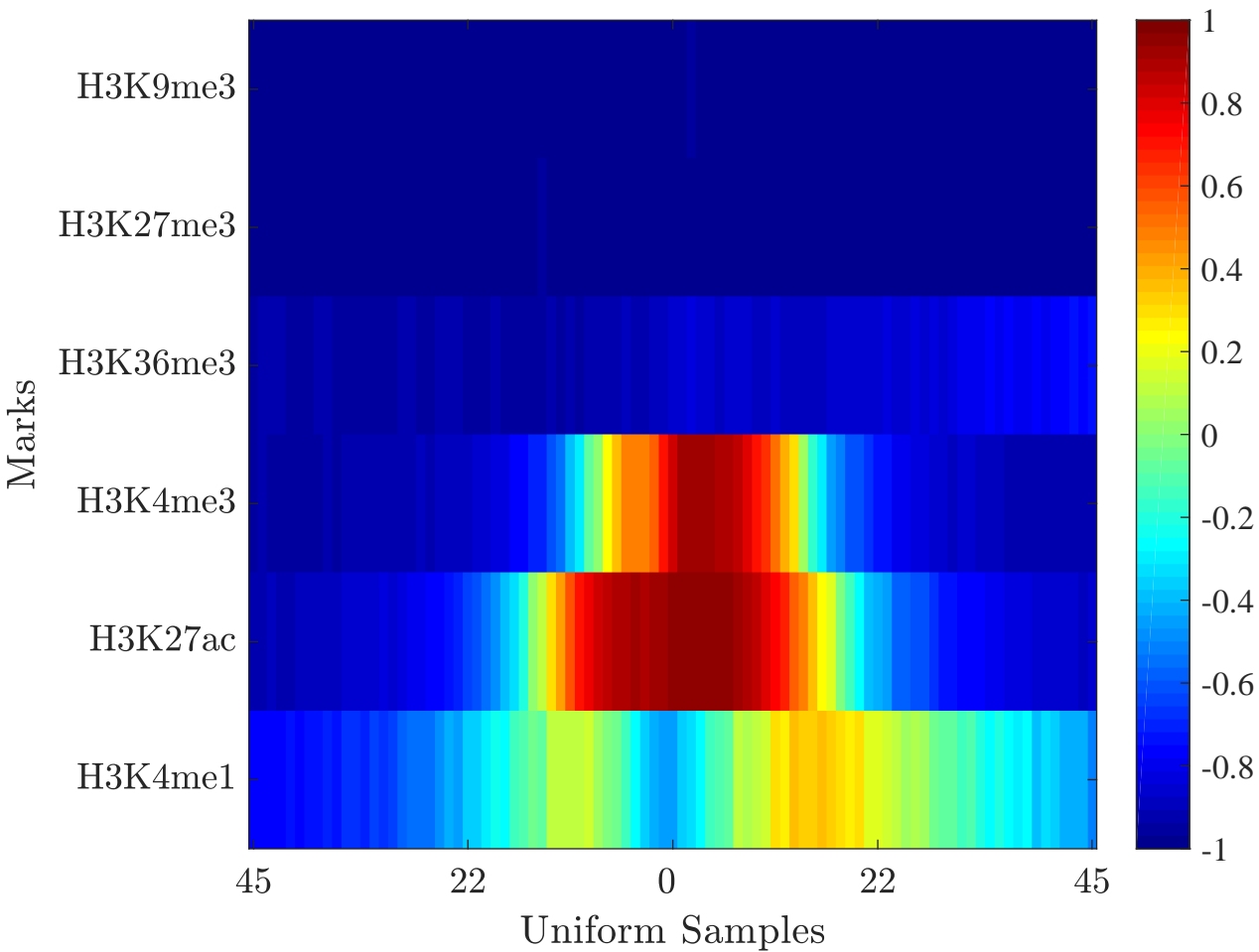

Supplement: Supplementary file 1 — HebbPlots of active promoters on the positive strand. This compressed file (.tar.gz) includes HebbPlots of promoters on the positive strand active in 57 tissues/cell types. (TAR 2949 kb) [file 12859_2018_2312_MOESM1_ESM.tar › file2/E113.pdf]

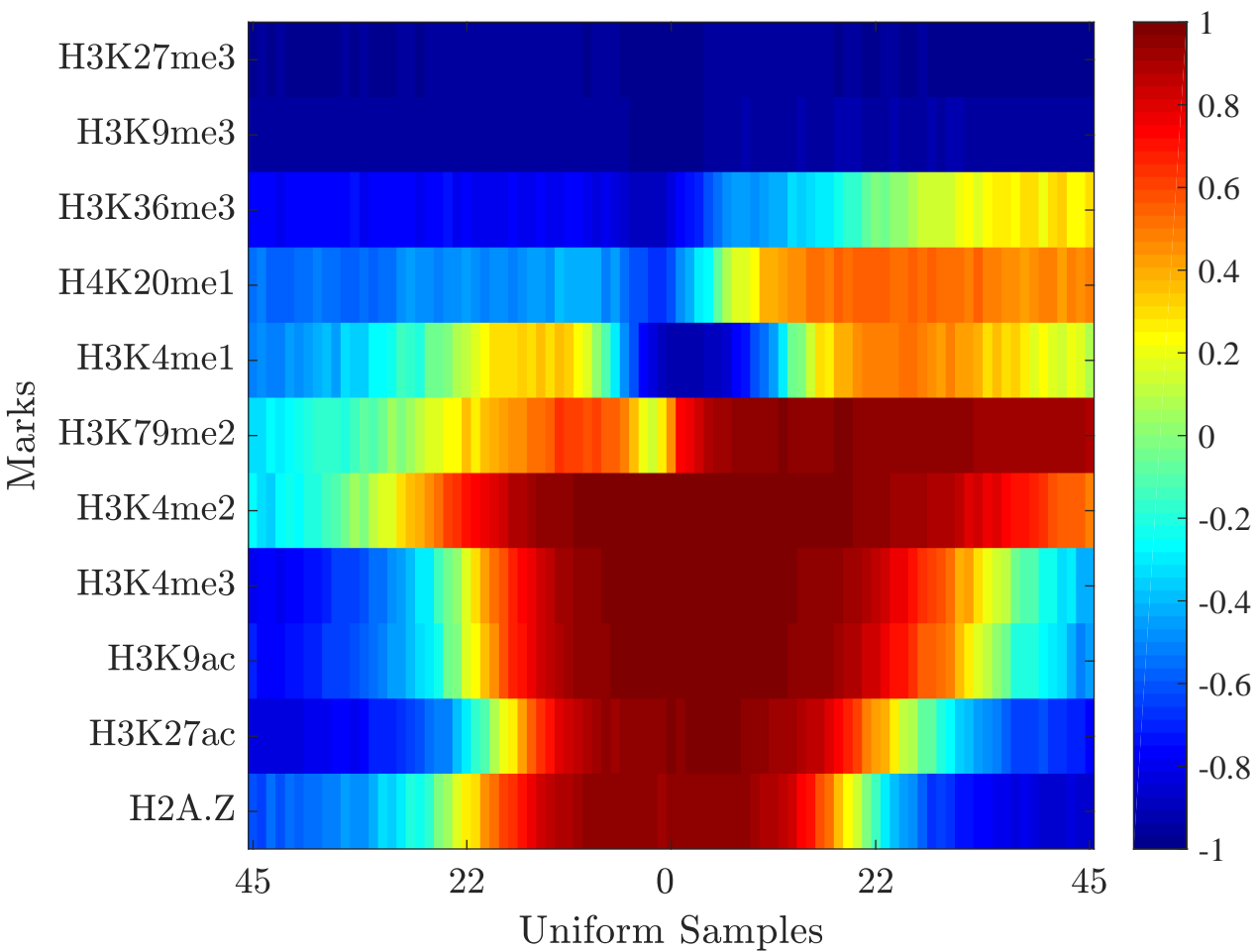

Supplement: Supplementary file 1 — HebbPlots of active promoters on the positive strand. This compressed file (.tar.gz) includes HebbPlots of promoters on the positive strand active in 57 tissues/cell types. (TAR 2949 kb) [file 12859_2018_2312_MOESM1_ESM.tar › file2/E114.pdf]

Marks

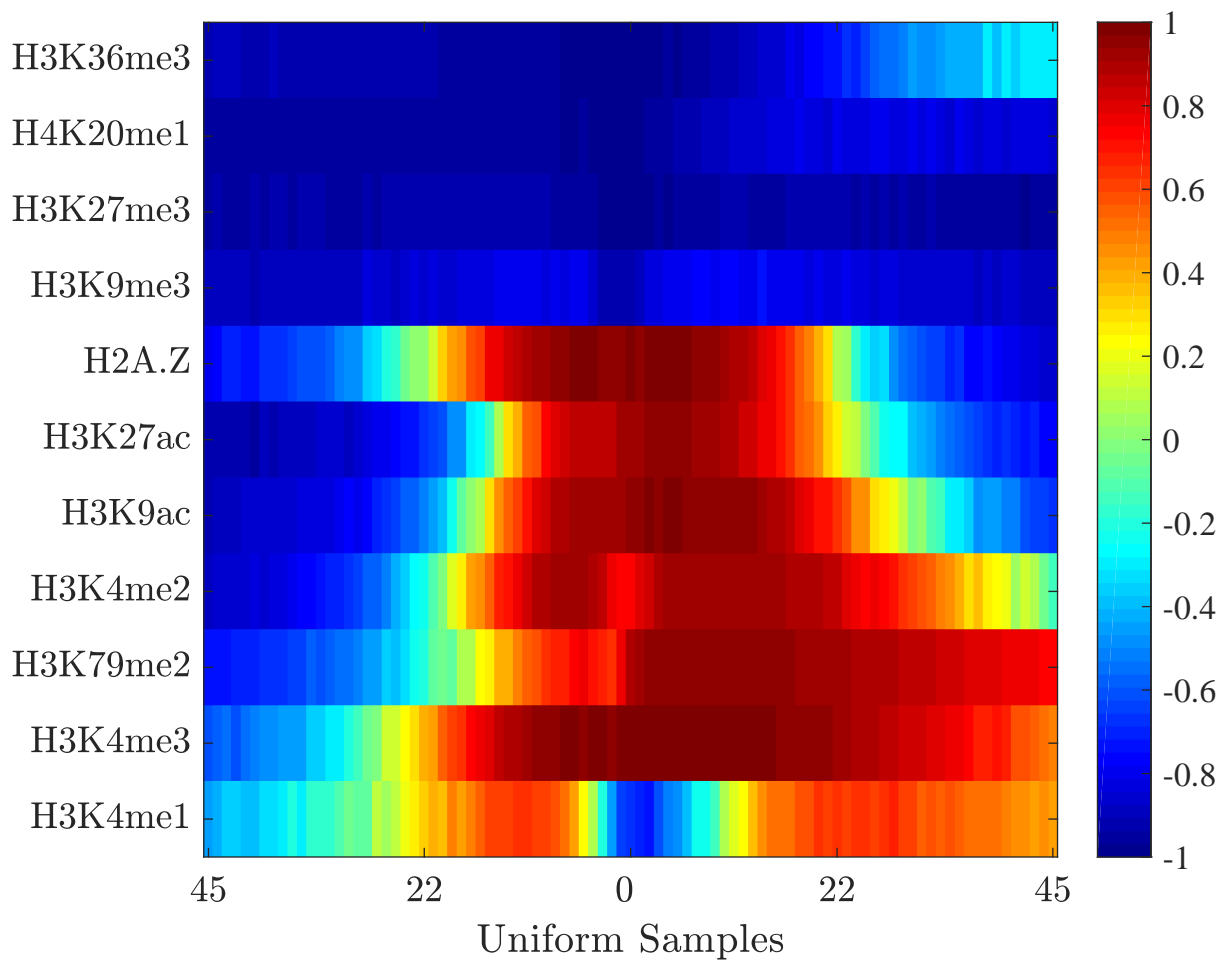

Supplement: Supplementary file 1 — HebbPlots of active promoters on the positive strand. This compressed file (.tar.gz) includes HebbPlots of promoters on the positive strand active in 57 tissues/cell types. (TAR 2949 kb) [file 12859_2018_2312_MOESM1_ESM.tar › file2/E116.pdf]

Marks

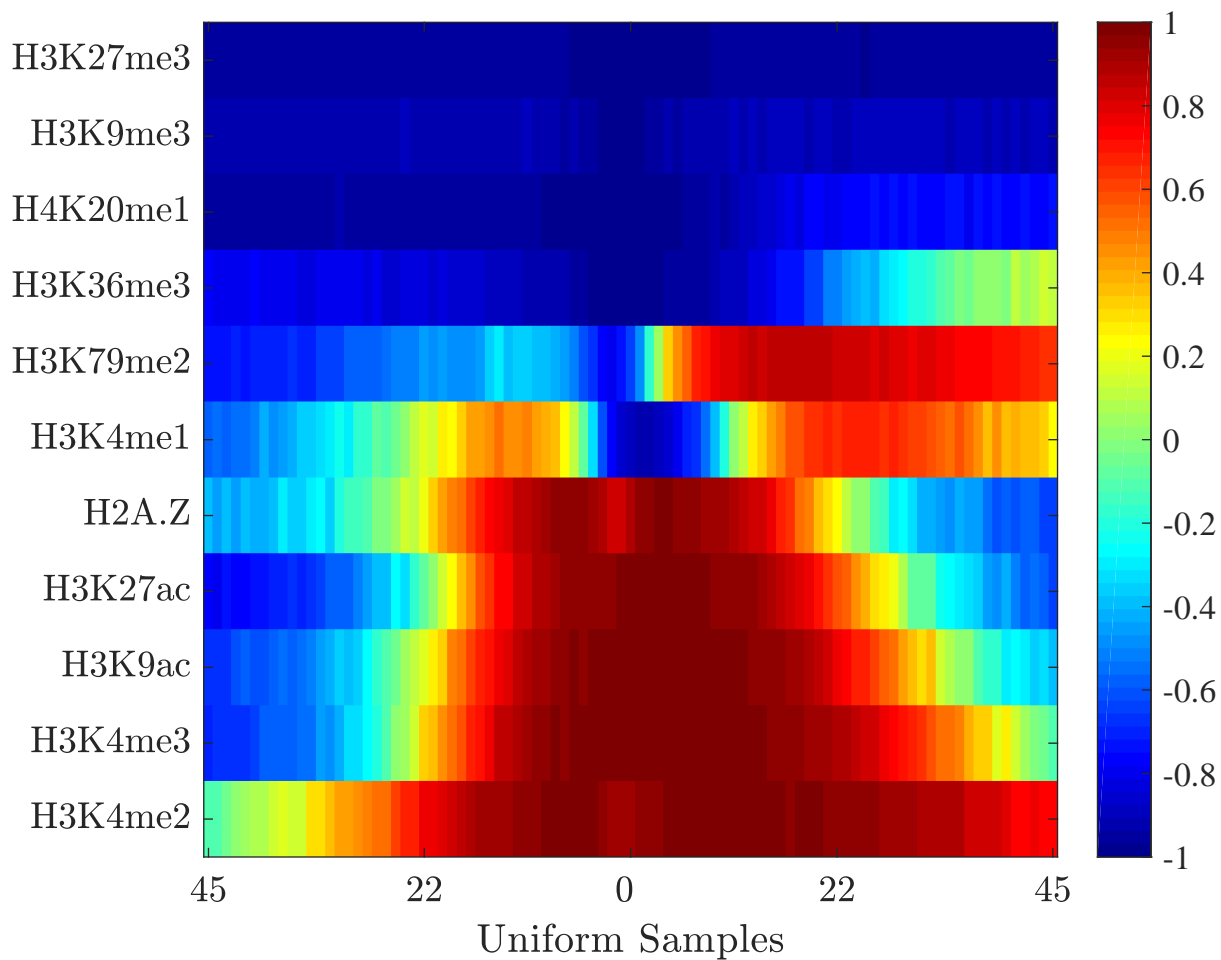

Supplement: Supplementary file 1 — HebbPlots of active promoters on the positive strand. This compressed file (.tar.gz) includes HebbPlots of promoters on the positive strand active in 57 tissues/cell types. (TAR 2949 kb) [file 12859_2018_2312_MOESM1_ESM.tar › file2/E117.pdf]

Marks

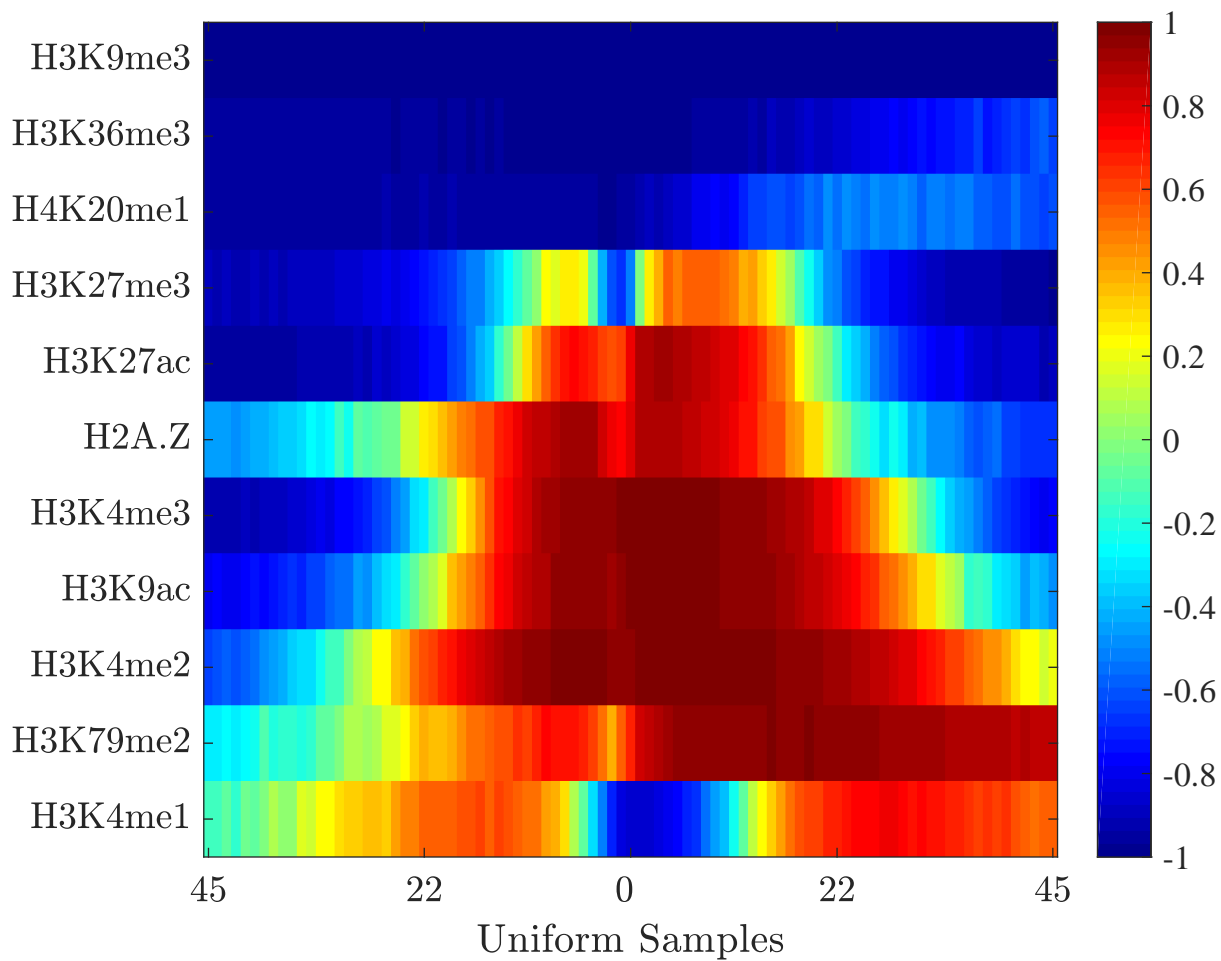

Supplement: Supplementary file 1 — HebbPlots of active promoters on the positive strand. This compressed file (.tar.gz) includes HebbPlots of promoters on the positive strand active in 57 tissues/cell types. (TAR 2949 kb) [file 12859_2018_2312_MOESM1_ESM.tar › file2/E118.pdf]

Marks

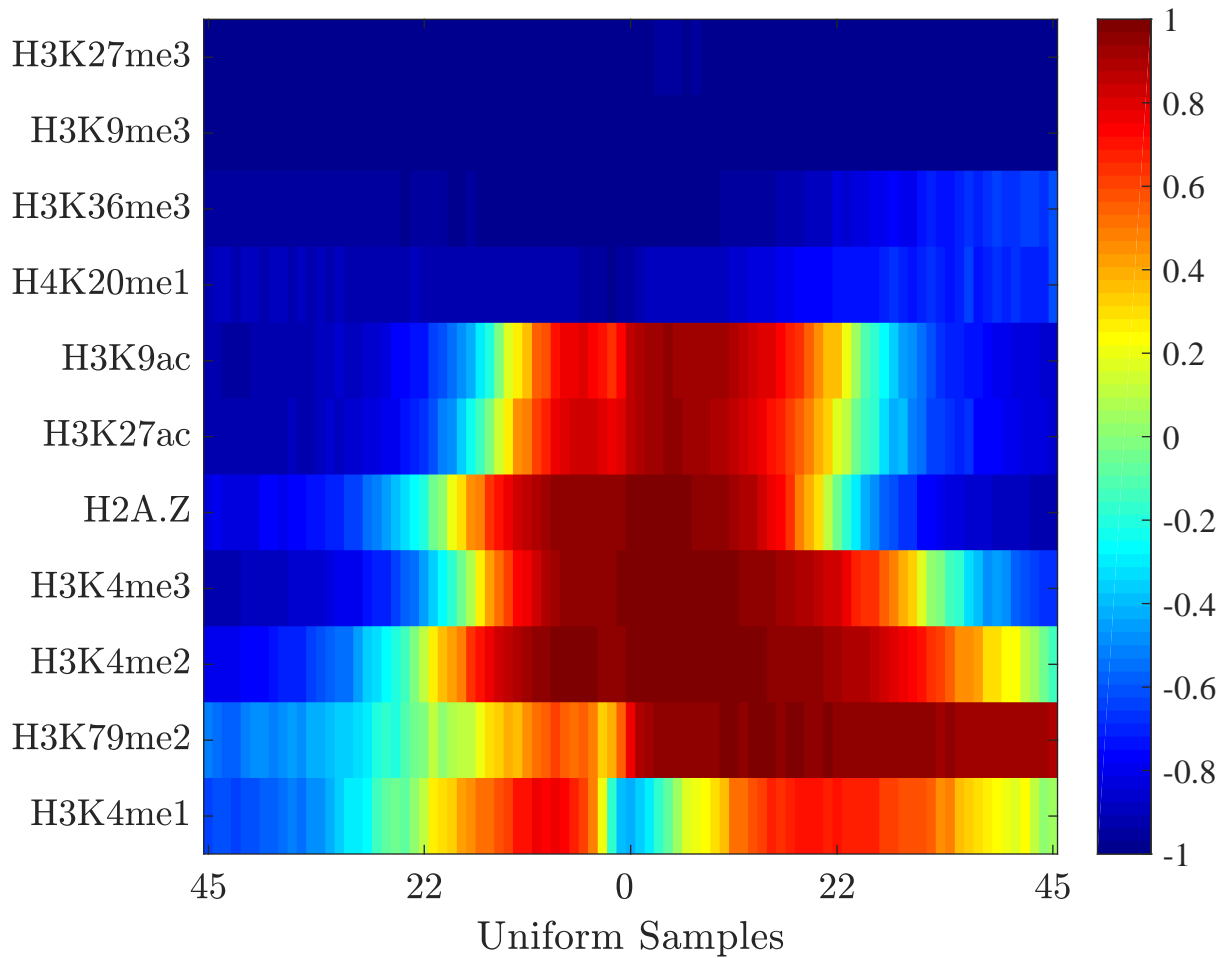

Supplement: Supplementary file 1 — HebbPlots of active promoters on the positive strand. This compressed file (.tar.gz) includes HebbPlots of promoters on the positive strand active in 57 tissues/cell types. (TAR 2949 kb) [file 12859_2018_2312_MOESM1_ESM.tar › file2/E119.pdf]

Marks

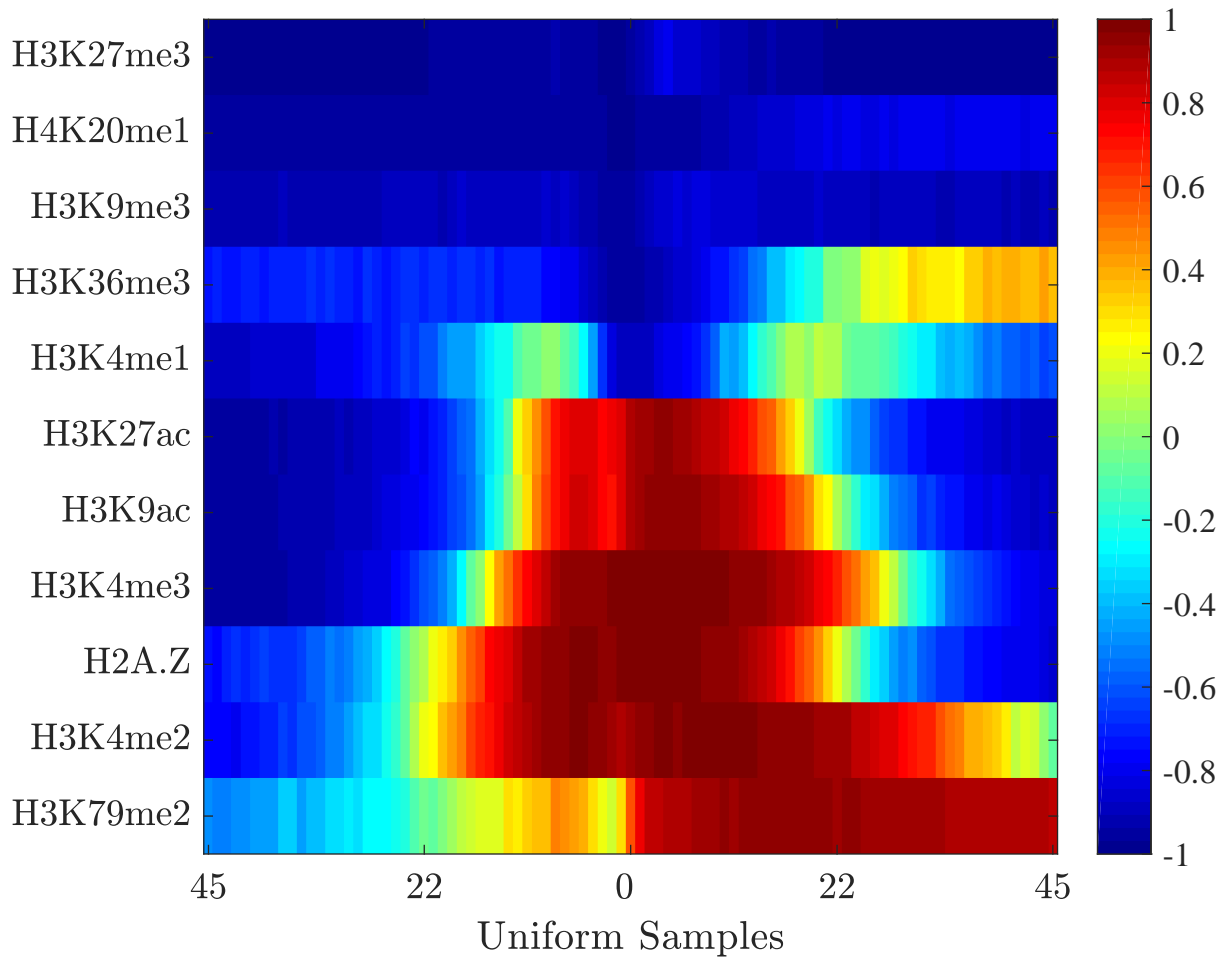

Supplement: Supplementary file 1 — HebbPlots of active promoters on the positive strand. This compressed file (.tar.gz) includes HebbPlots of promoters on the positive strand active in 57 tissues/cell types. (TAR 2949 kb) [file 12859_2018_2312_MOESM1_ESM.tar › file2/E120.pdf]

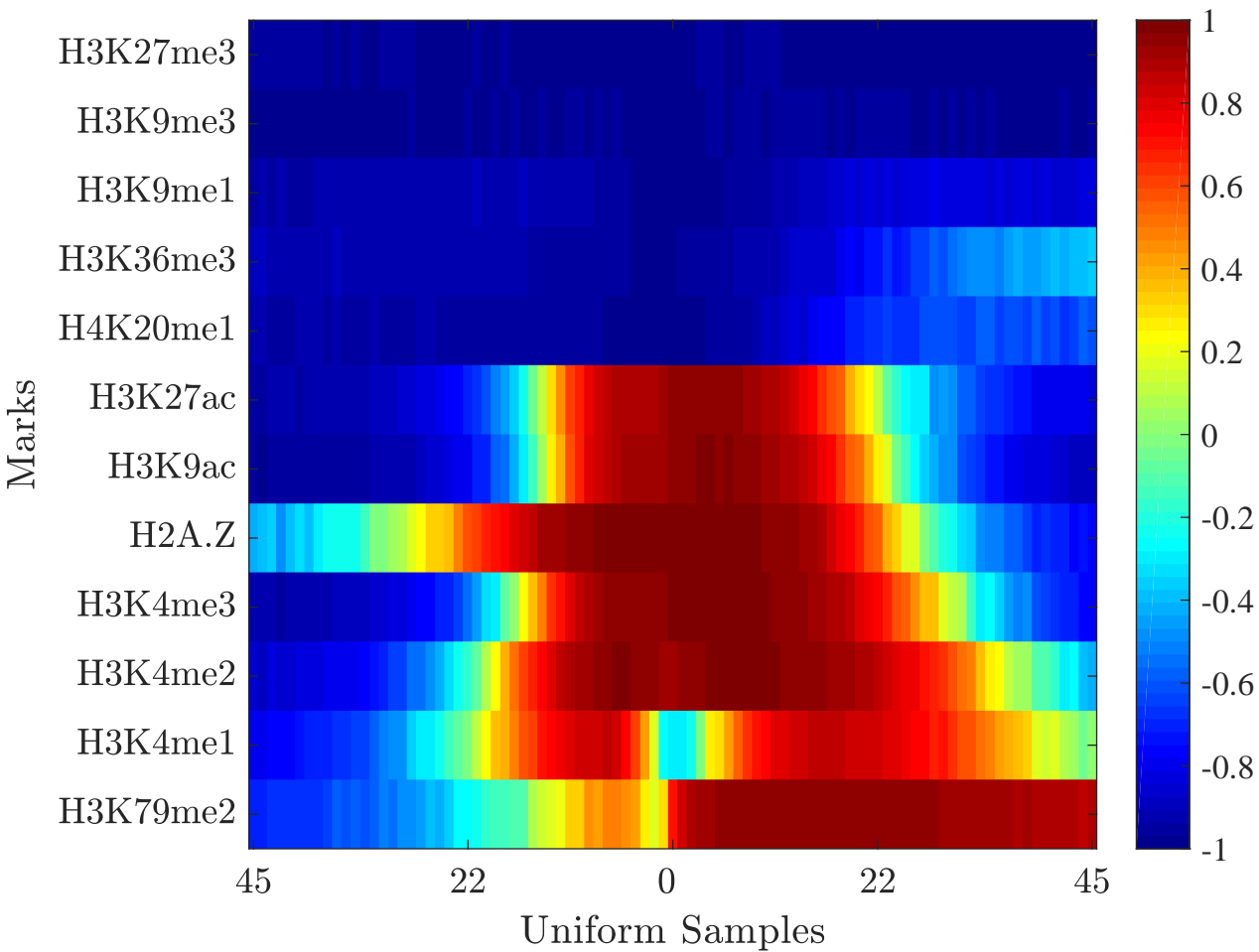

Supplement: Supplementary file 1 — HebbPlots of active promoters on the positive strand. This compressed file (.tar.gz) includes HebbPlots of promoters on the positive strand active in 57 tissues/cell types. (TAR 2949 kb) [file 12859_2018_2312_MOESM1_ESM.tar › file2/E122.pdf]

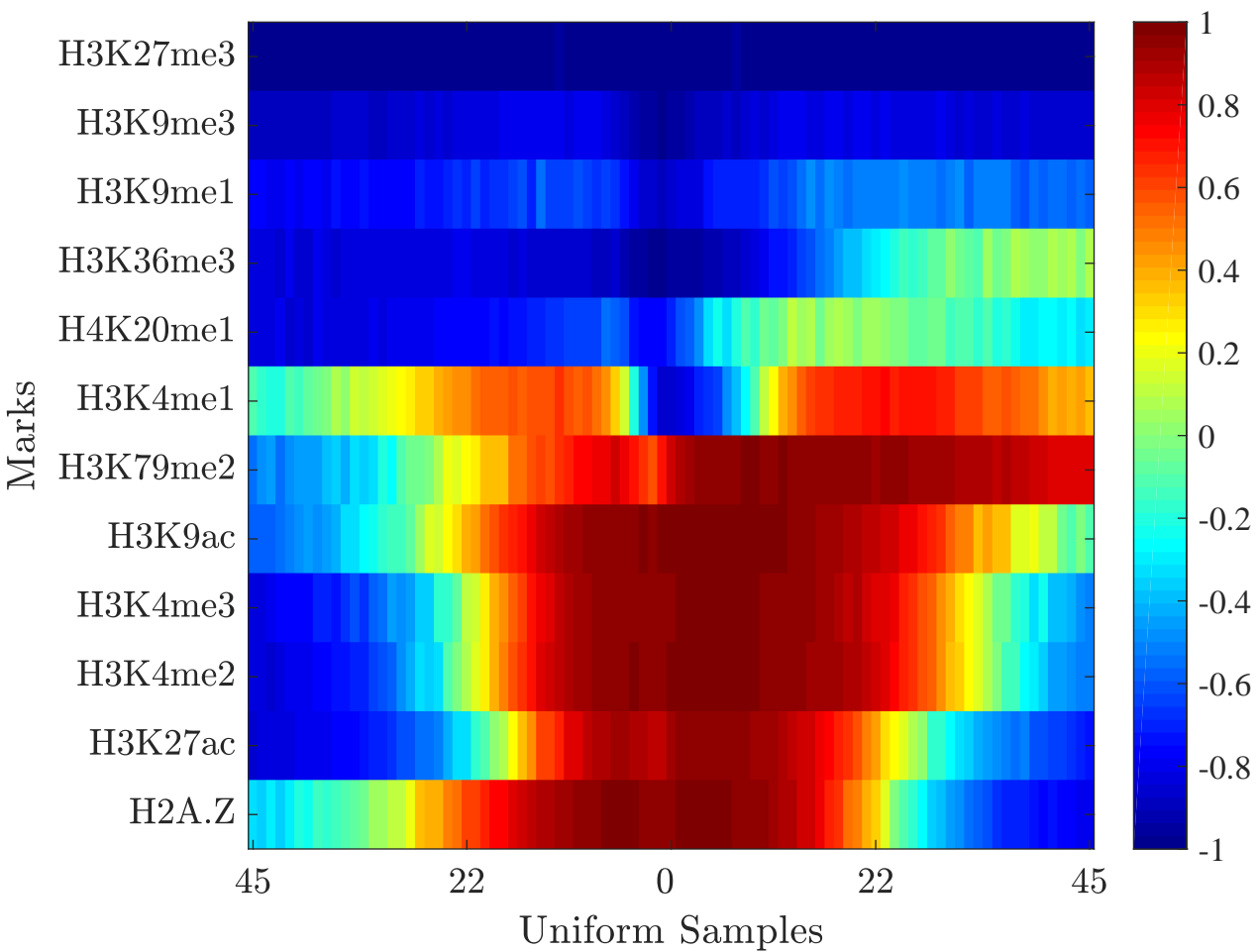

Supplement: Supplementary file 1 — HebbPlots of active promoters on the positive strand. This compressed file (.tar.gz) includes HebbPlots of promoters on the positive strand active in 57 tissues/cell types. (TAR 2949 kb) [file 12859_2018_2312_MOESM1_ESM.tar › file2/E123.pdf]

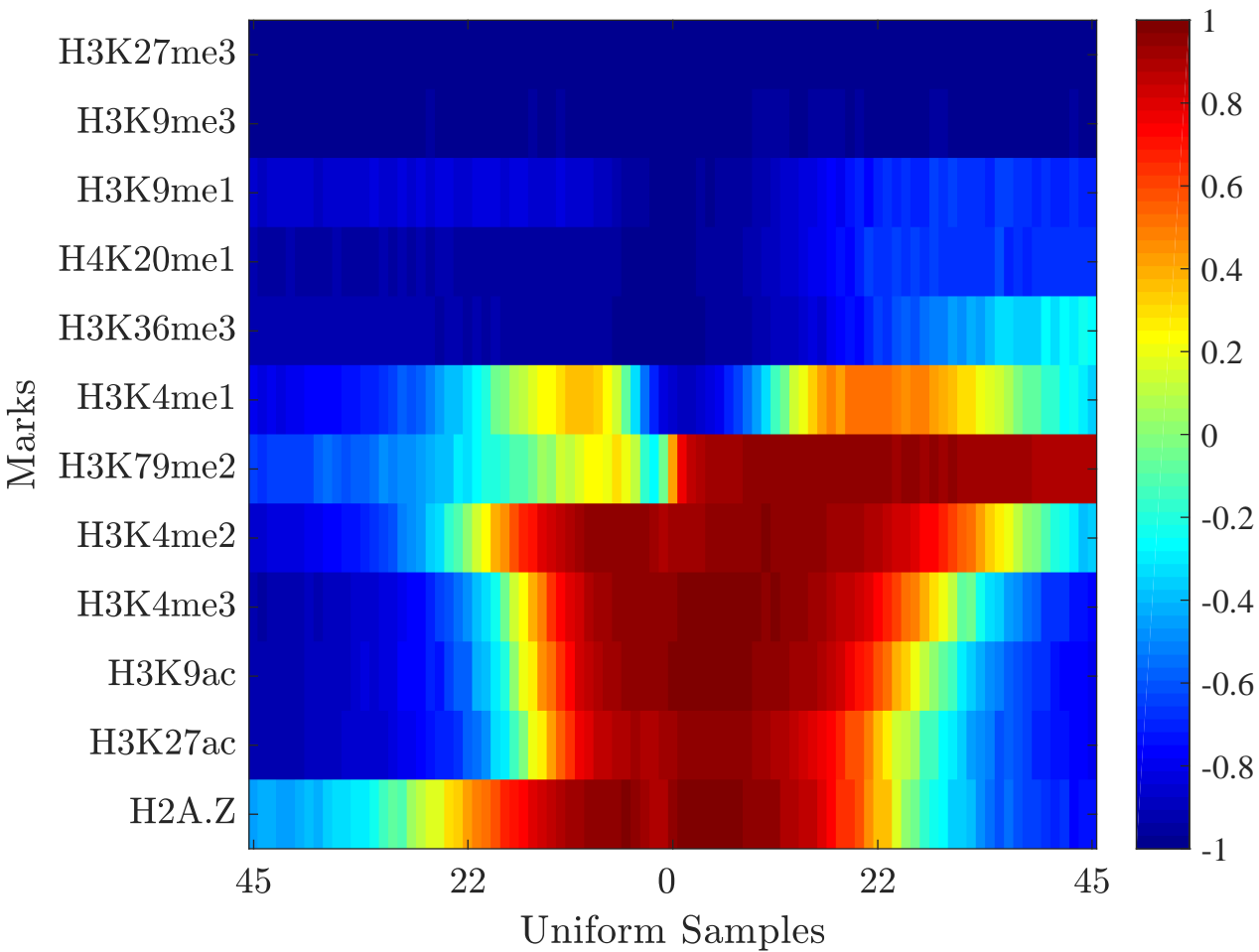

Supplement: Supplementary file 1 — HebbPlots of active promoters on the positive strand. This compressed file (.tar.gz) includes HebbPlots of promoters on the positive strand active in 57 tissues/cell types. (TAR 2949 kb) [file 12859_2018_2312_MOESM1_ESM.tar › file2/E127.pdf]

Marks

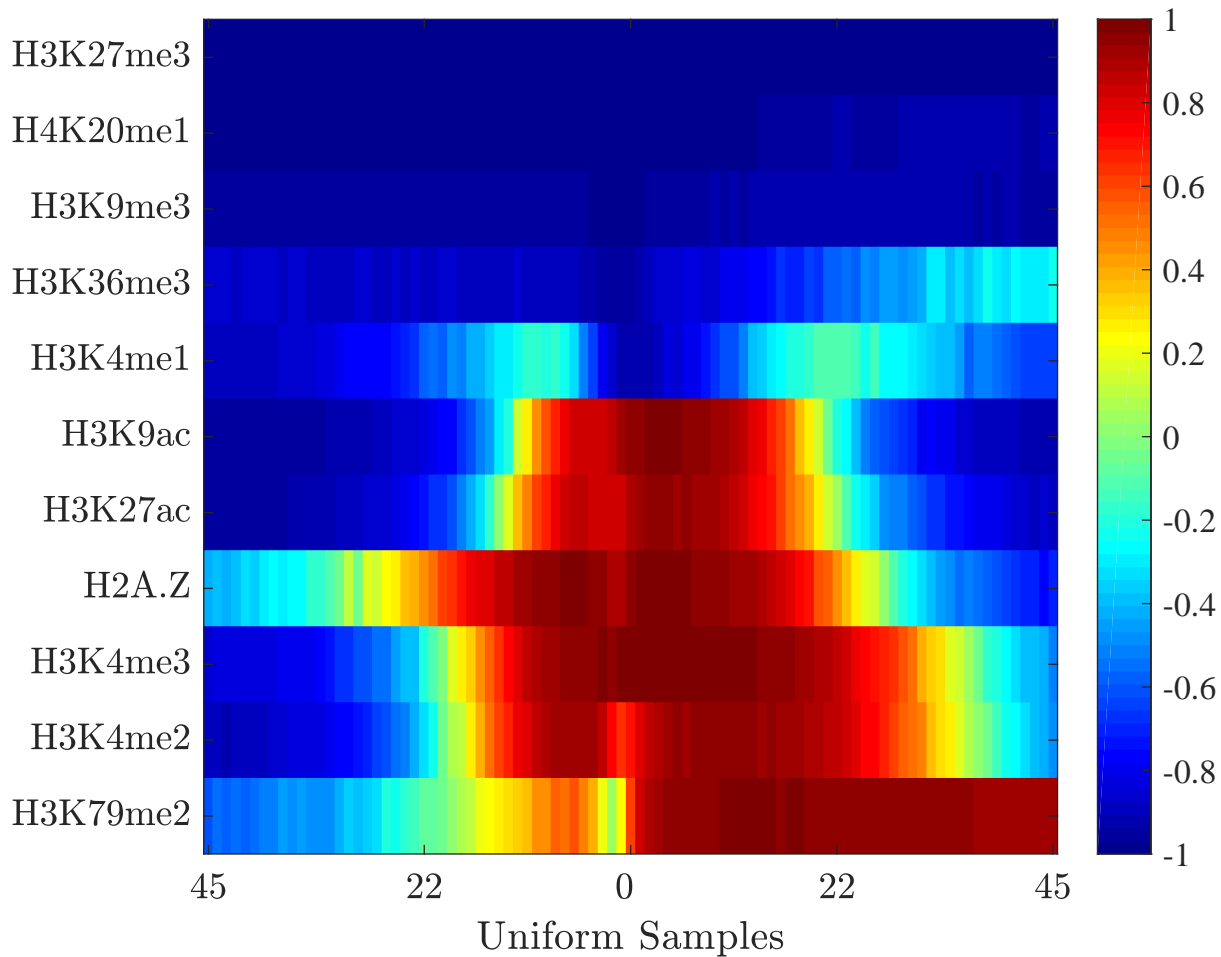

Supplement: Supplementary file 1 — HebbPlots of active promoters on the positive strand. This compressed file (.tar.gz) includes HebbPlots of promoters on the positive strand active in 57 tissues/cell types. (TAR 2949 kb) [file 12859_2018_2312_MOESM1_ESM.tar › file2/E128.pdf]

Marks

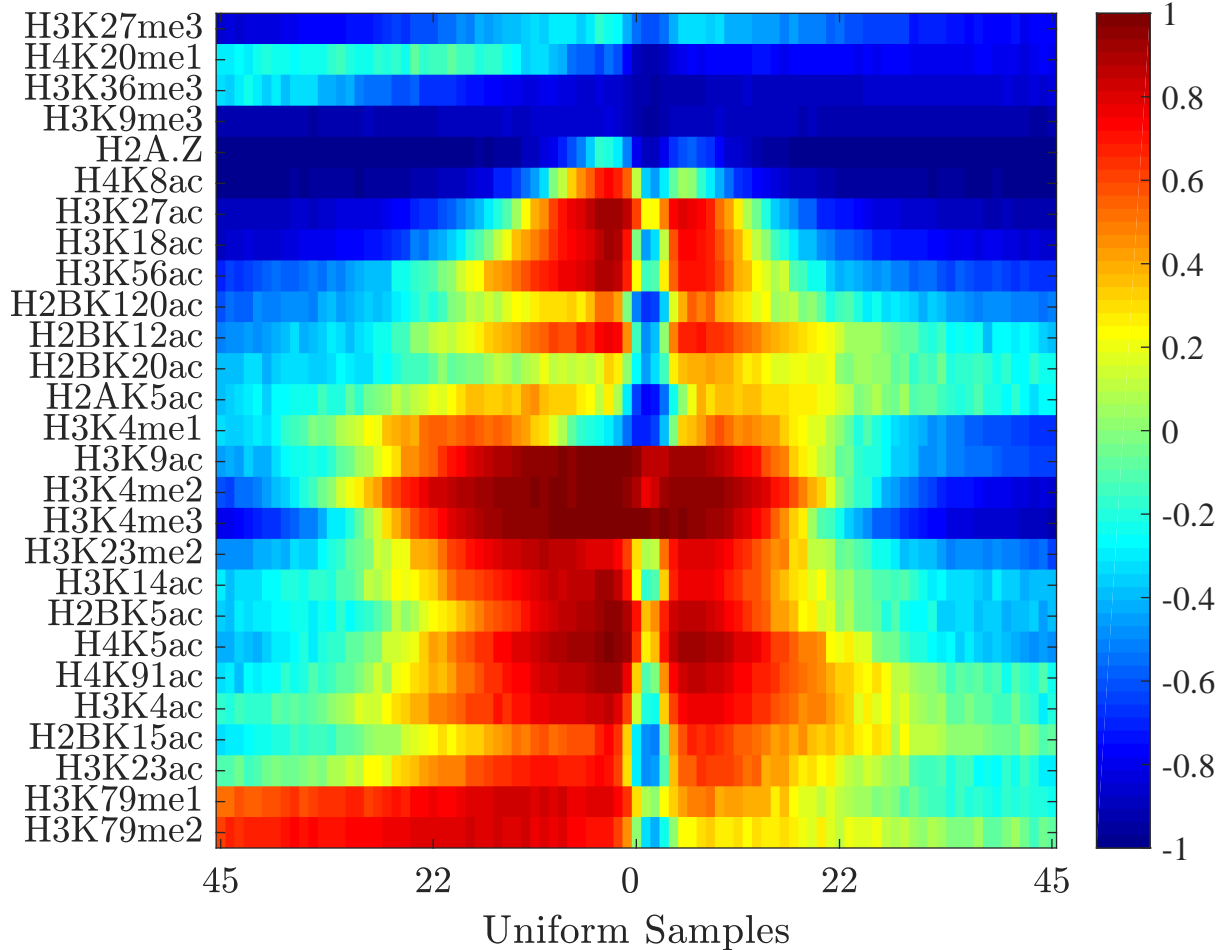

Supplement: Supplementary file 2 — HebbPlots of active promoters on the negative strand. This compressed file (.tar.gz) includes HebbPlots of promoters on the negative strand active in 57 tissues/cell types. (TAR 2952 kb) [file 12859_2018_2312_MOESM2_ESM.tar › file3/E003.pdf]

Marks

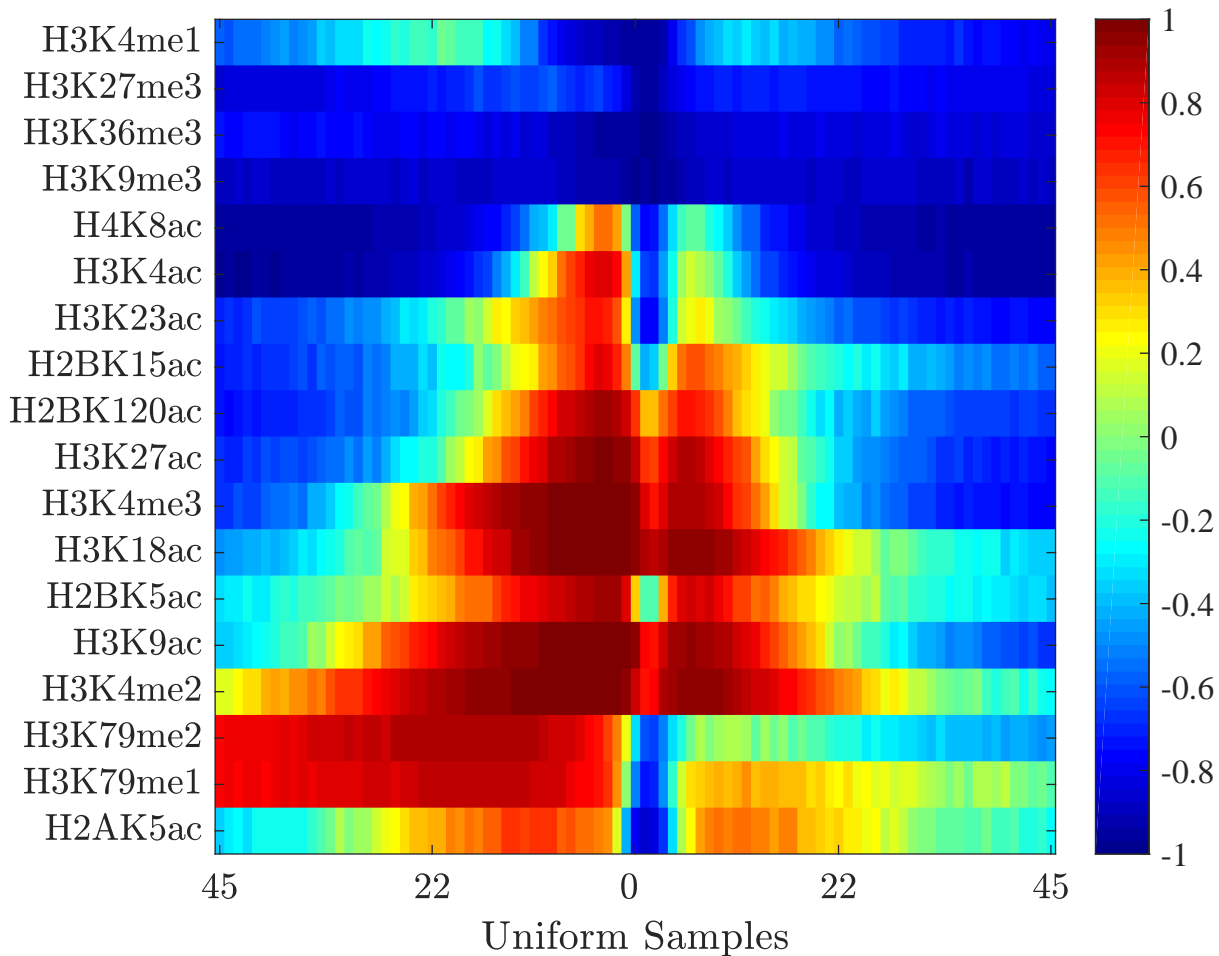

Supplement: Supplementary file 2 — HebbPlots of active promoters on the negative strand. This compressed file (.tar.gz) includes HebbPlots of promoters on the negative strand active in 57 tissues/cell types. (TAR 2952 kb) [file 12859_2018_2312_MOESM2_ESM.tar › file3/E004.pdf]

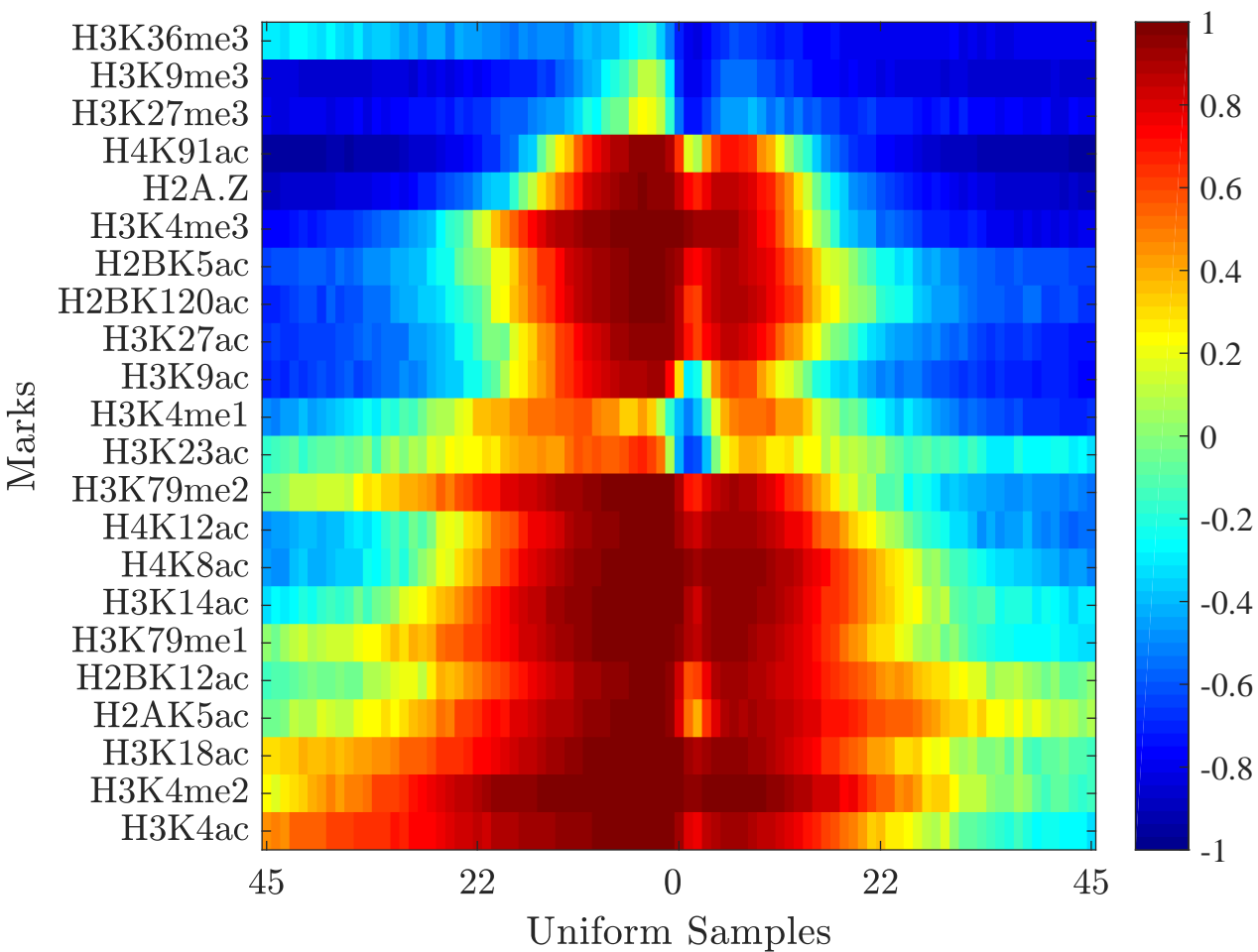

Supplement: Supplementary file 2 — HebbPlots of active promoters on the negative strand. This compressed file (.tar.gz) includes HebbPlots of promoters on the negative strand active in 57 tissues/cell types. (TAR 2952 kb) [file 12859_2018_2312_MOESM2_ESM.tar › file3/E005.pdf]

Marks

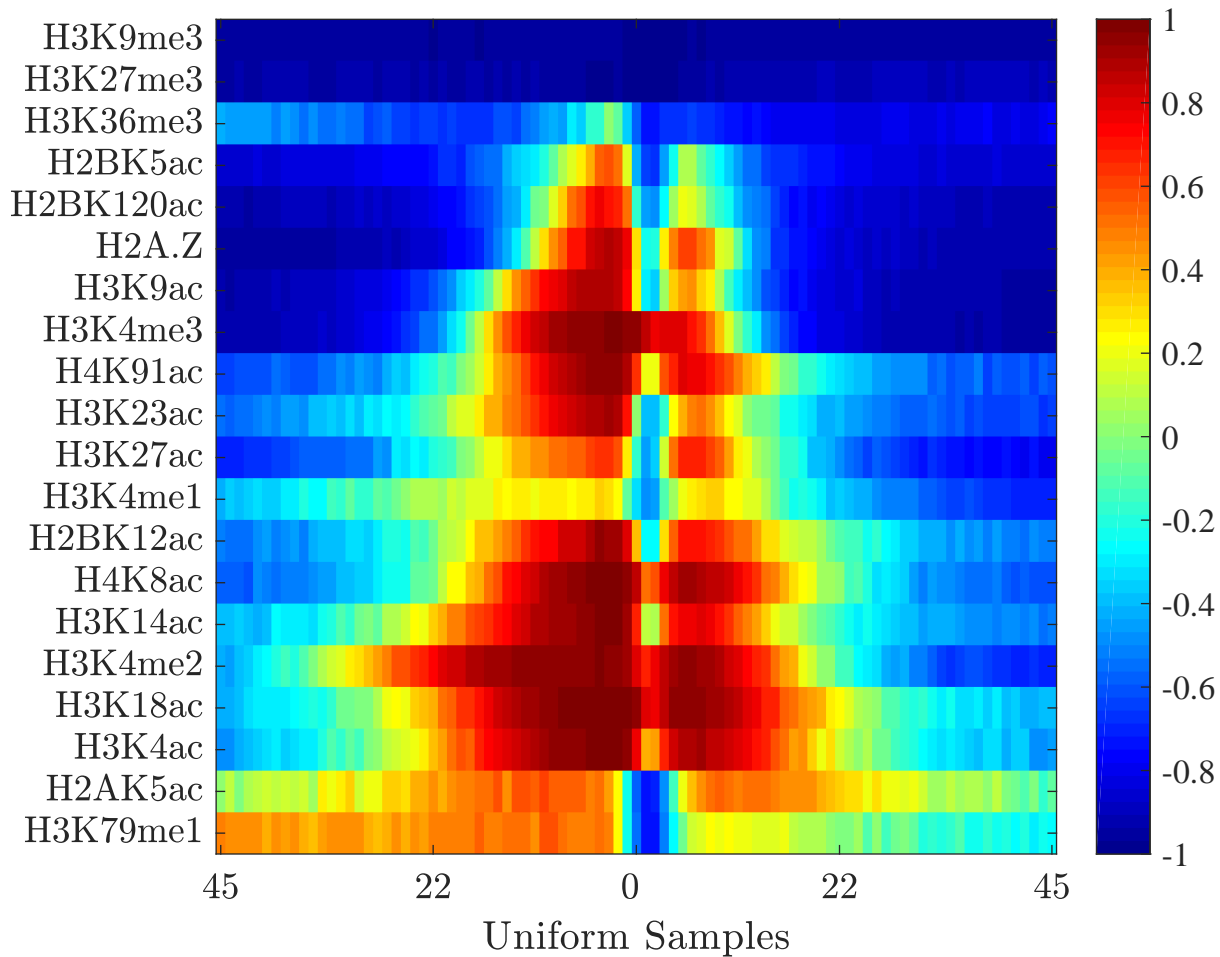

Supplement: Supplementary file 2 — HebbPlots of active promoters on the negative strand. This compressed file (.tar.gz) includes HebbPlots of promoters on the negative strand active in 57 tissues/cell types. (TAR 2952 kb) [file 12859_2018_2312_MOESM2_ESM.tar › file3/E006.pdf]

Marks

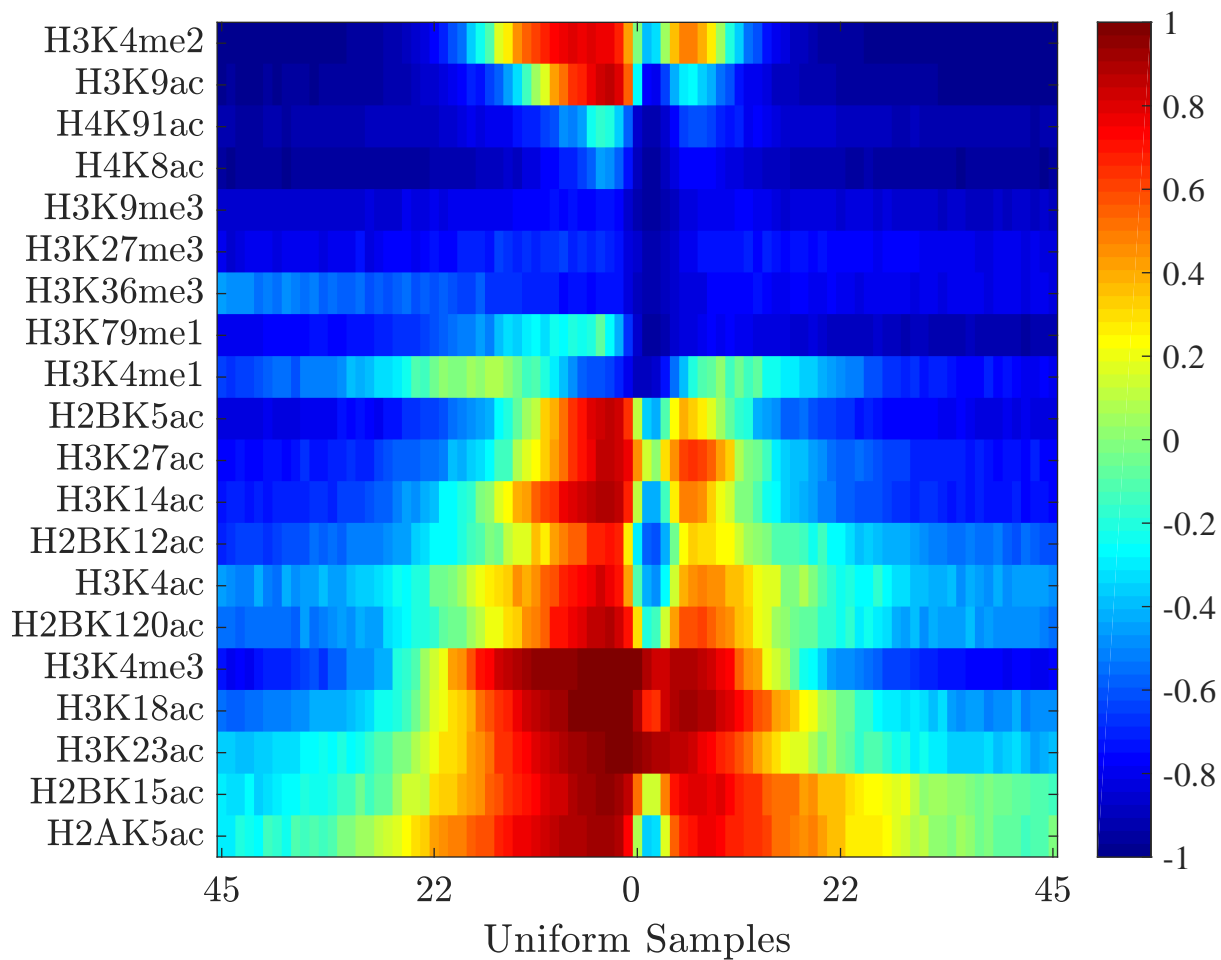

Supplement: Supplementary file 2 — HebbPlots of active promoters on the negative strand. This compressed file (.tar.gz) includes HebbPlots of promoters on the negative strand active in 57 tissues/cell types. (TAR 2952 kb) [file 12859_2018_2312_MOESM2_ESM.tar › file3/E007.pdf]

Marks

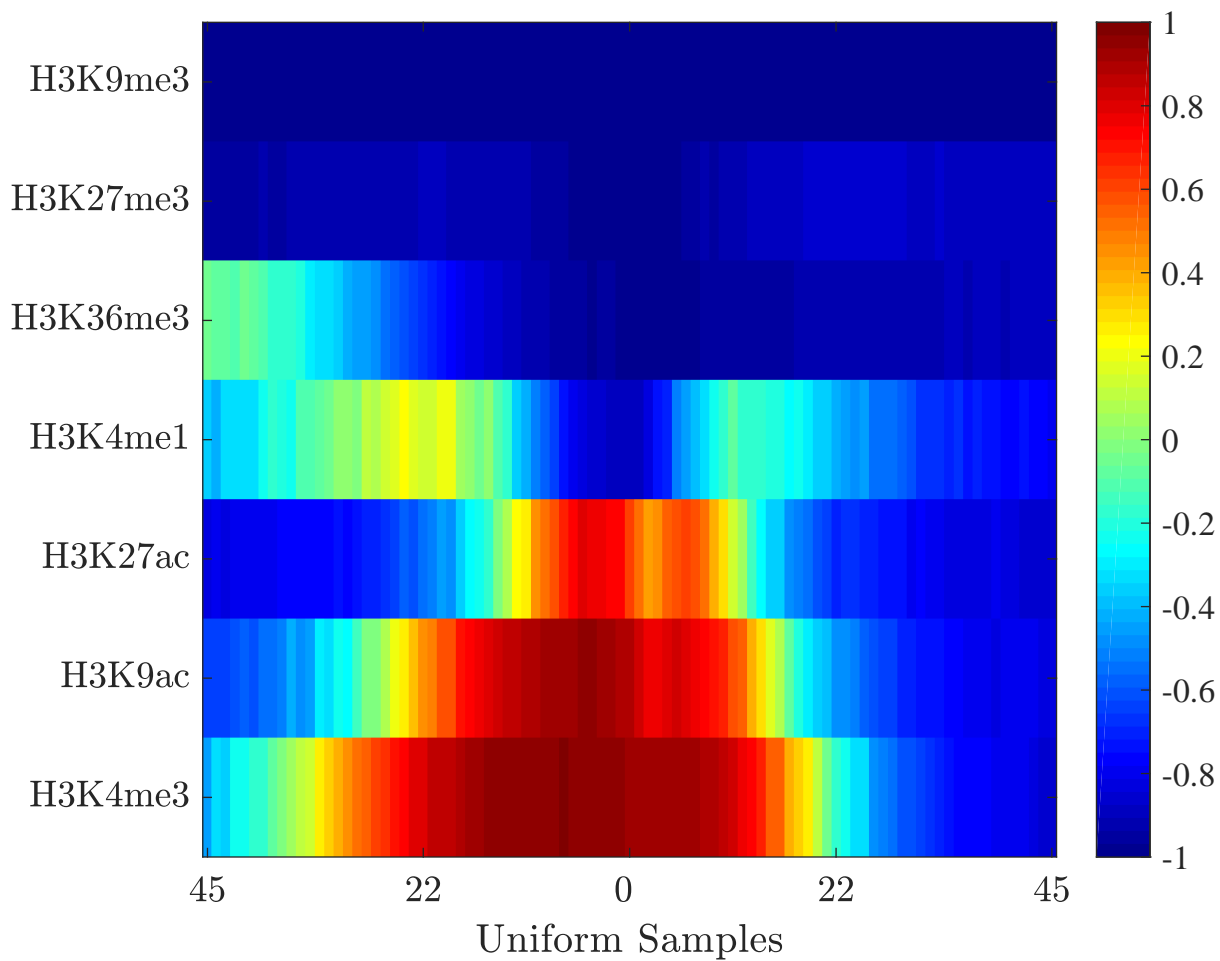

Supplement: Supplementary file 2 — HebbPlots of active promoters on the negative strand. This compressed file (.tar.gz) includes HebbPlots of promoters on the negative strand active in 57 tissues/cell types. (TAR 2952 kb) [file 12859_2018_2312_MOESM2_ESM.tar › file3/E011.pdf]

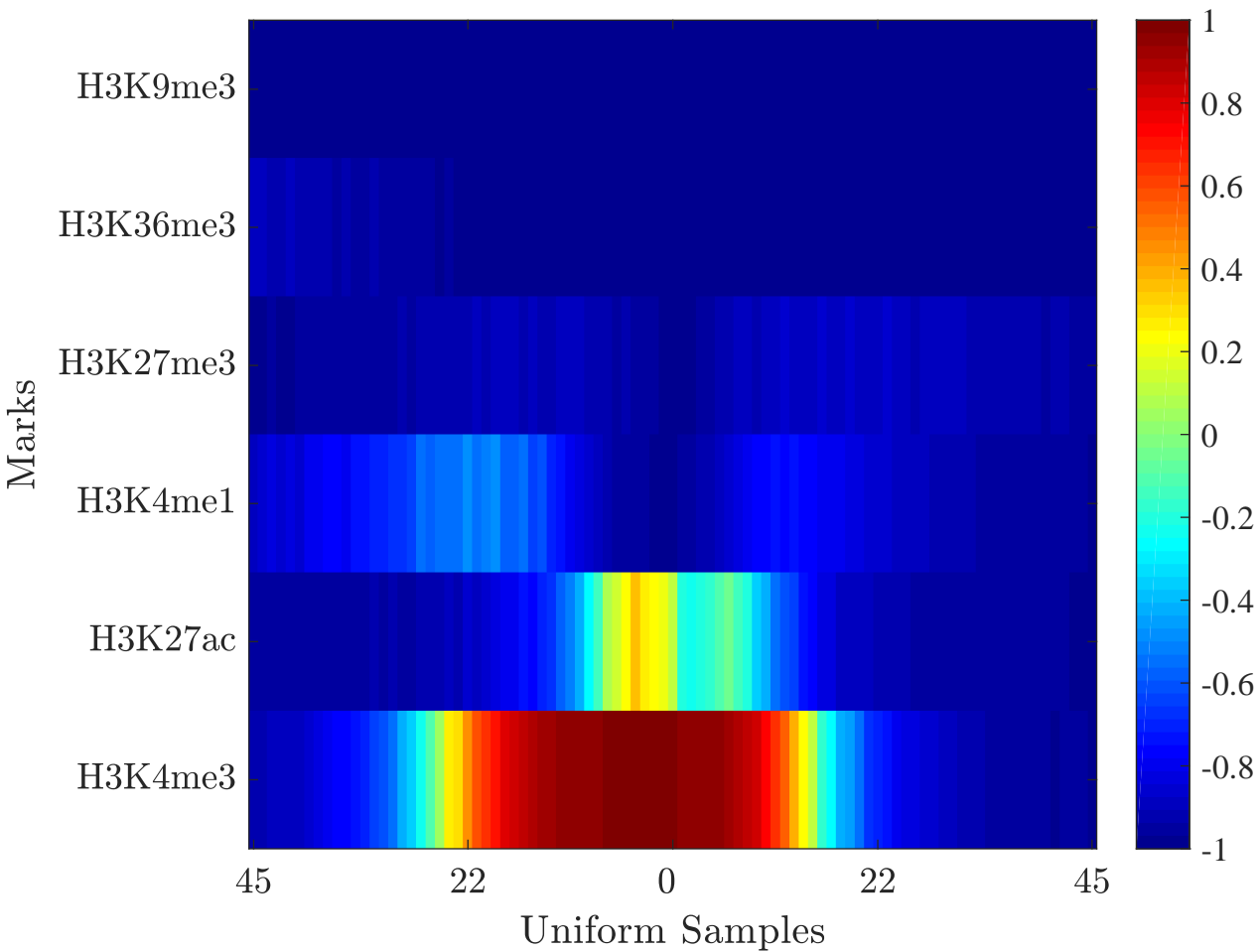

Supplement: Supplementary file 2 — HebbPlots of active promoters on the negative strand. This compressed file (.tar.gz) includes HebbPlots of promoters on the negative strand active in 57 tissues/cell types. (TAR 2952 kb) [file 12859_2018_2312_MOESM2_ESM.tar › file3/E012.pdf]

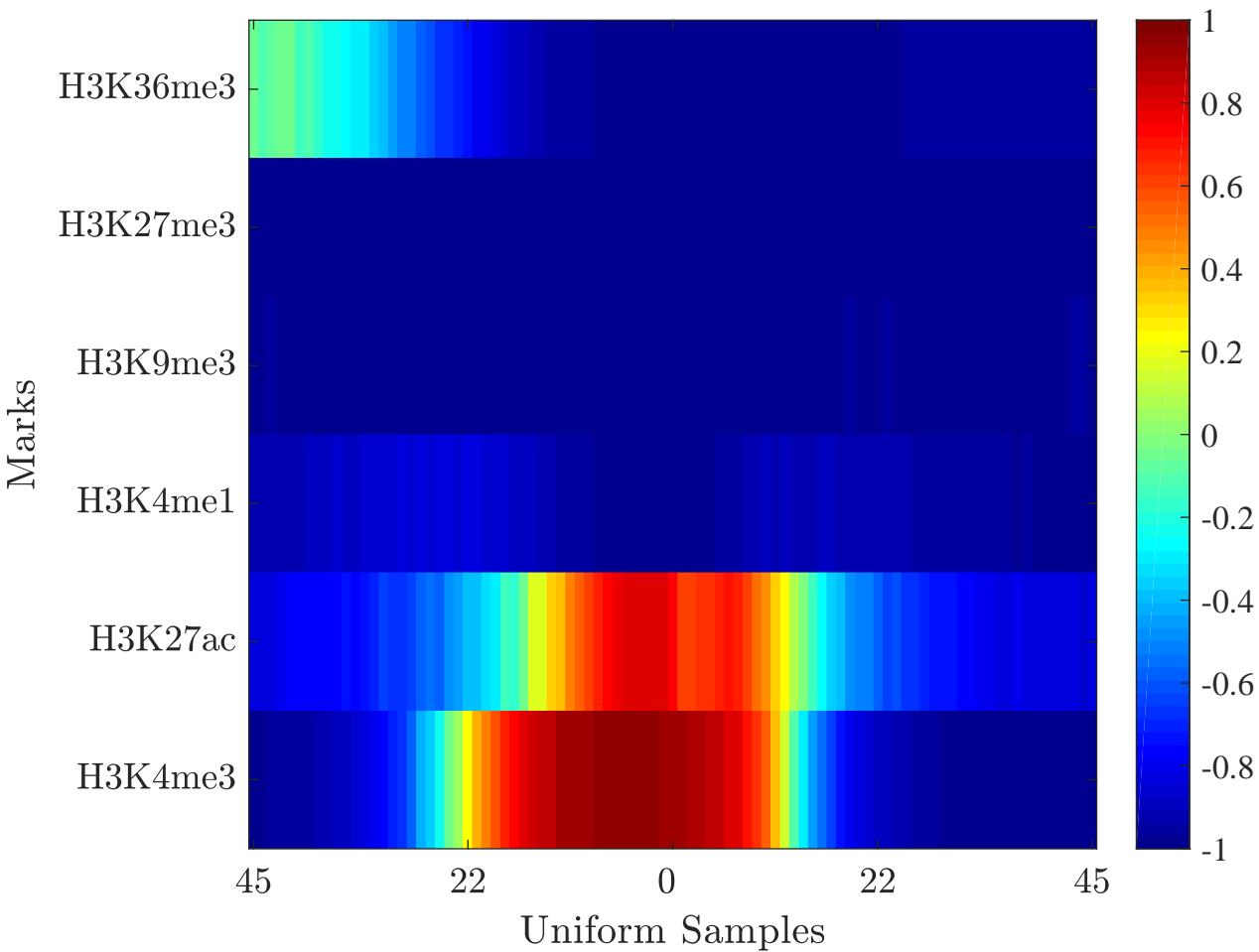

Supplement: Supplementary file 2 — HebbPlots of active promoters on the negative strand. This compressed file (.tar.gz) includes HebbPlots of promoters on the negative strand active in 57 tissues/cell types. (TAR 2952 kb) [file 12859_2018_2312_MOESM2_ESM.tar › file3/E013.pdf]

Marks

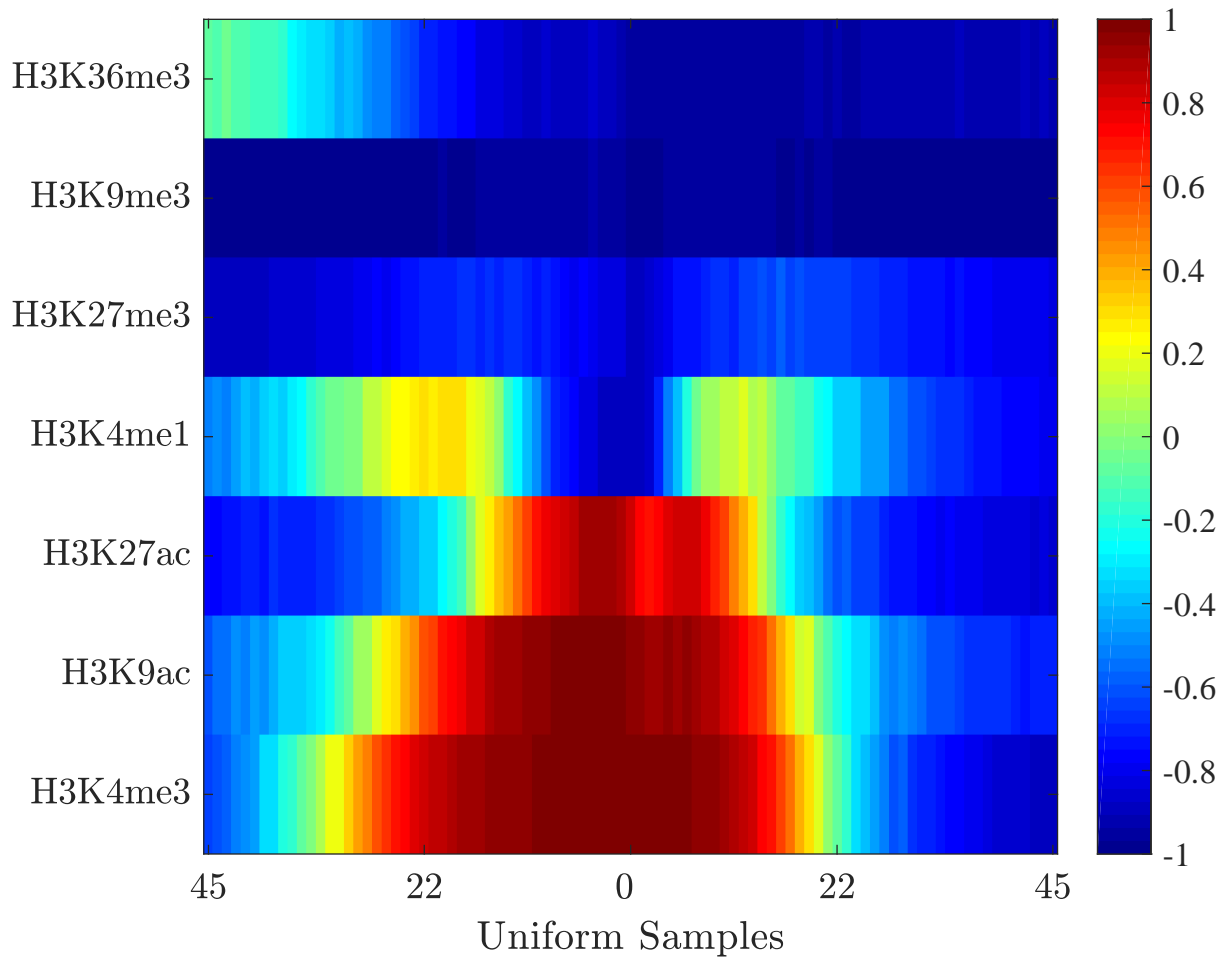

Supplement: Supplementary file 2 — HebbPlots of active promoters on the negative strand. This compressed file (.tar.gz) includes HebbPlots of promoters on the negative strand active in 57 tissues/cell types. (TAR 2952 kb) [file 12859_2018_2312_MOESM2_ESM.tar › file3/E016.pdf]

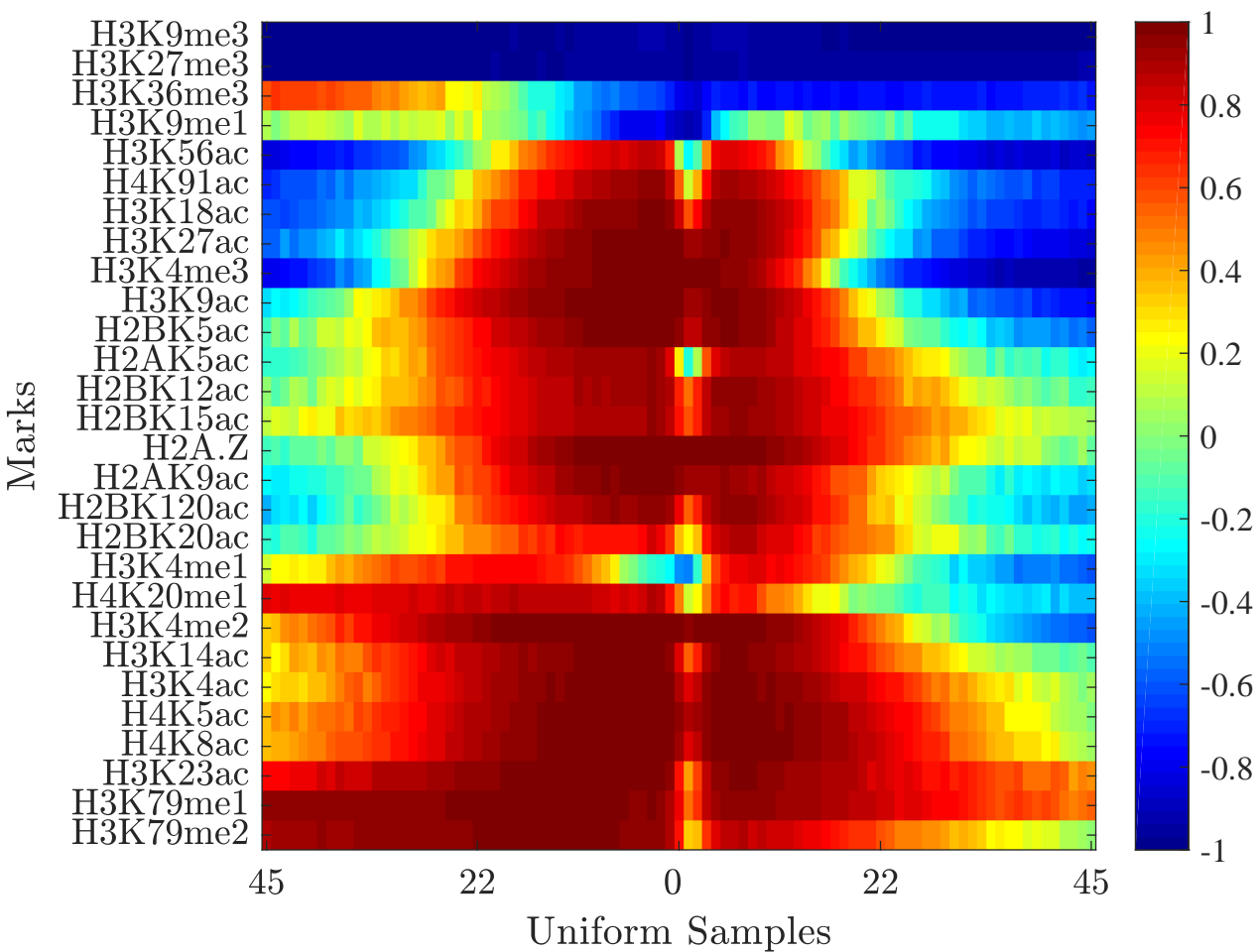

Supplement: Supplementary file 2 — HebbPlots of active promoters on the negative strand. This compressed file (.tar.gz) includes HebbPlots of promoters on the negative strand active in 57 tissues/cell types. (TAR 2952 kb) [file 12859_2018_2312_MOESM2_ESM.tar › file3/E017.pdf]

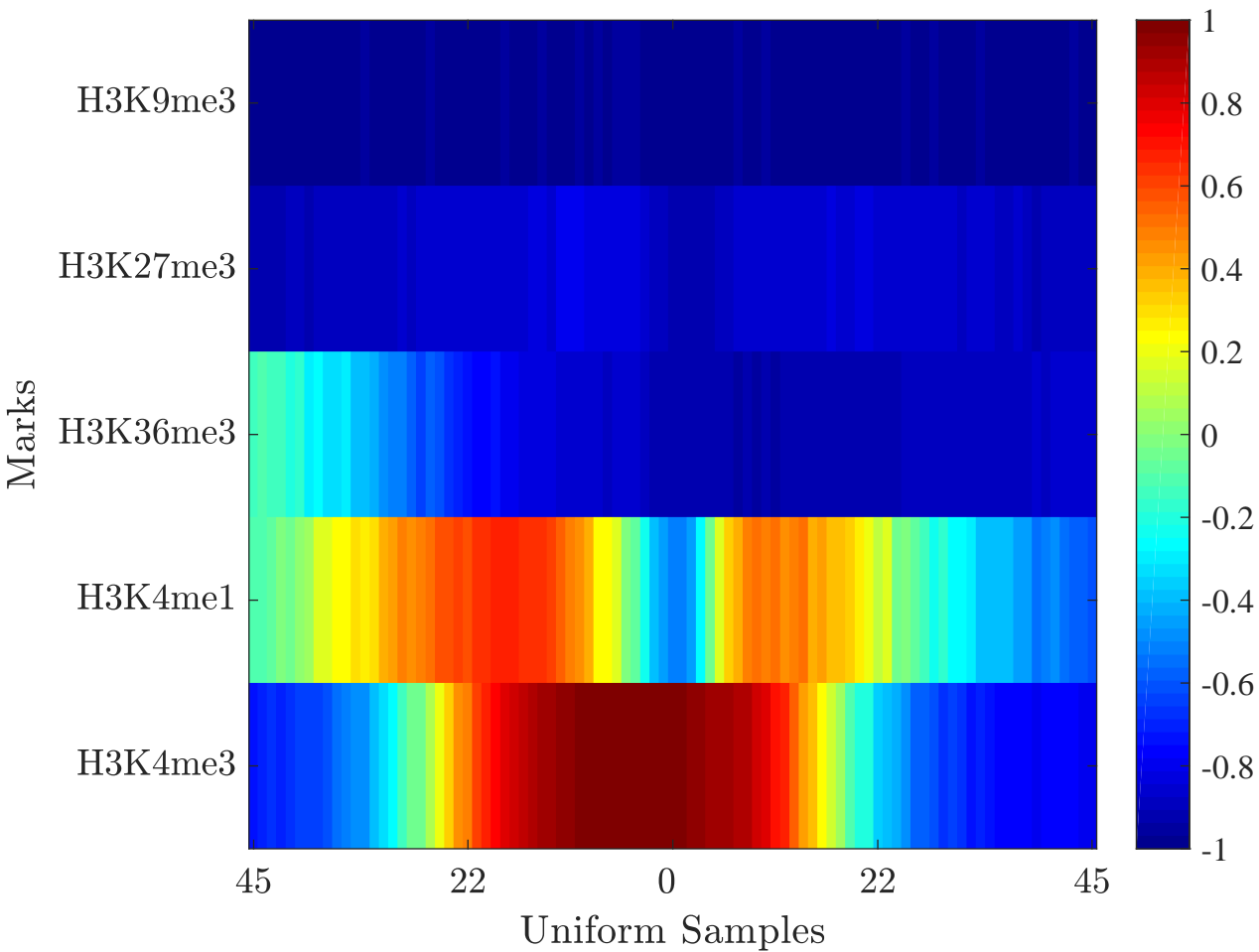

Supplement: Supplementary file 2 — HebbPlots of active promoters on the negative strand. This compressed file (.tar.gz) includes HebbPlots of promoters on the negative strand active in 57 tissues/cell types. (TAR 2952 kb) [file 12859_2018_2312_MOESM2_ESM.tar › file3/E024.pdf]

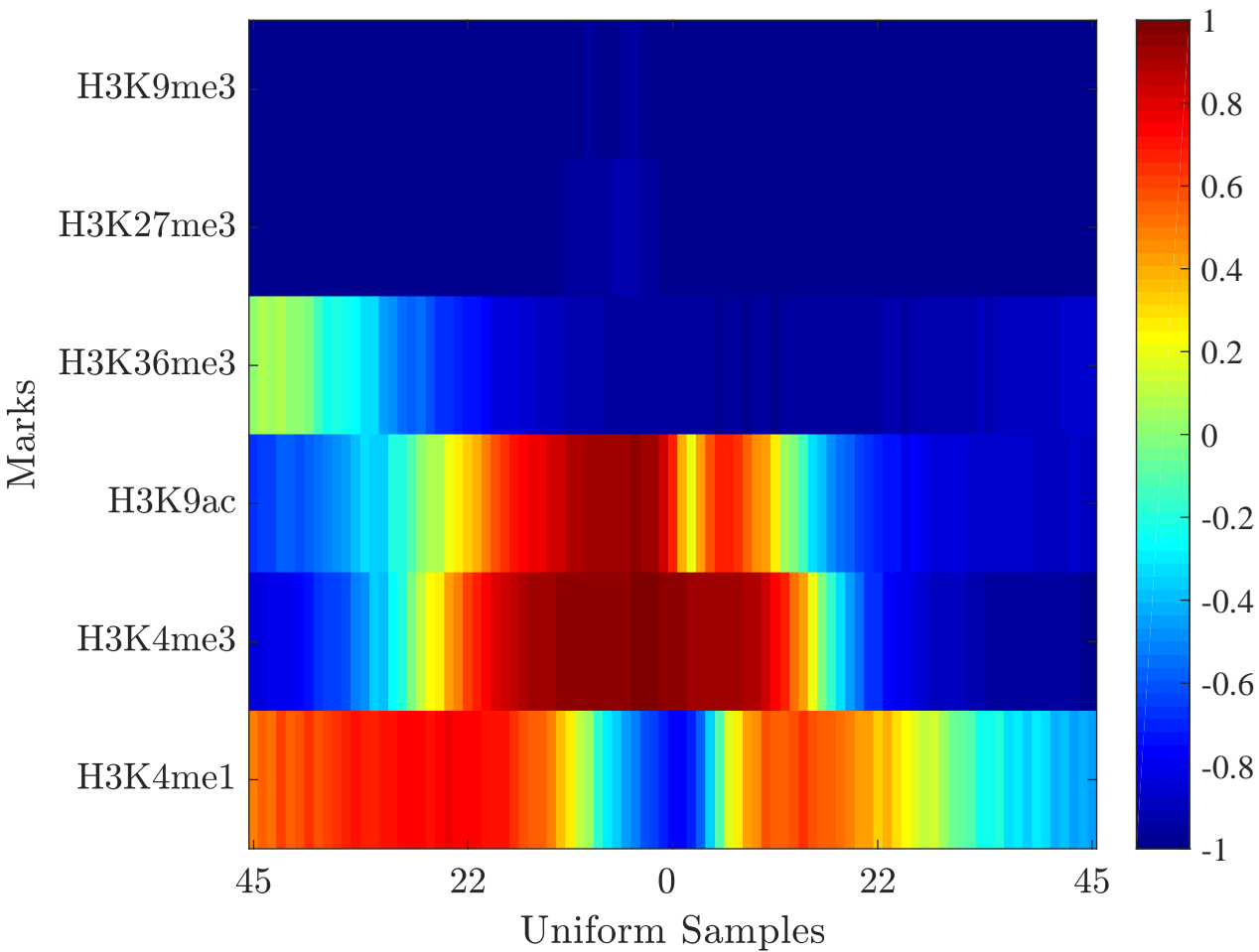

Supplement: Supplementary file 2 — HebbPlots of active promoters on the negative strand. This compressed file (.tar.gz) includes HebbPlots of promoters on the negative strand active in 57 tissues/cell types. (TAR 2952 kb) [file 12859_2018_2312_MOESM2_ESM.tar › file3/E027.pdf]

Marks

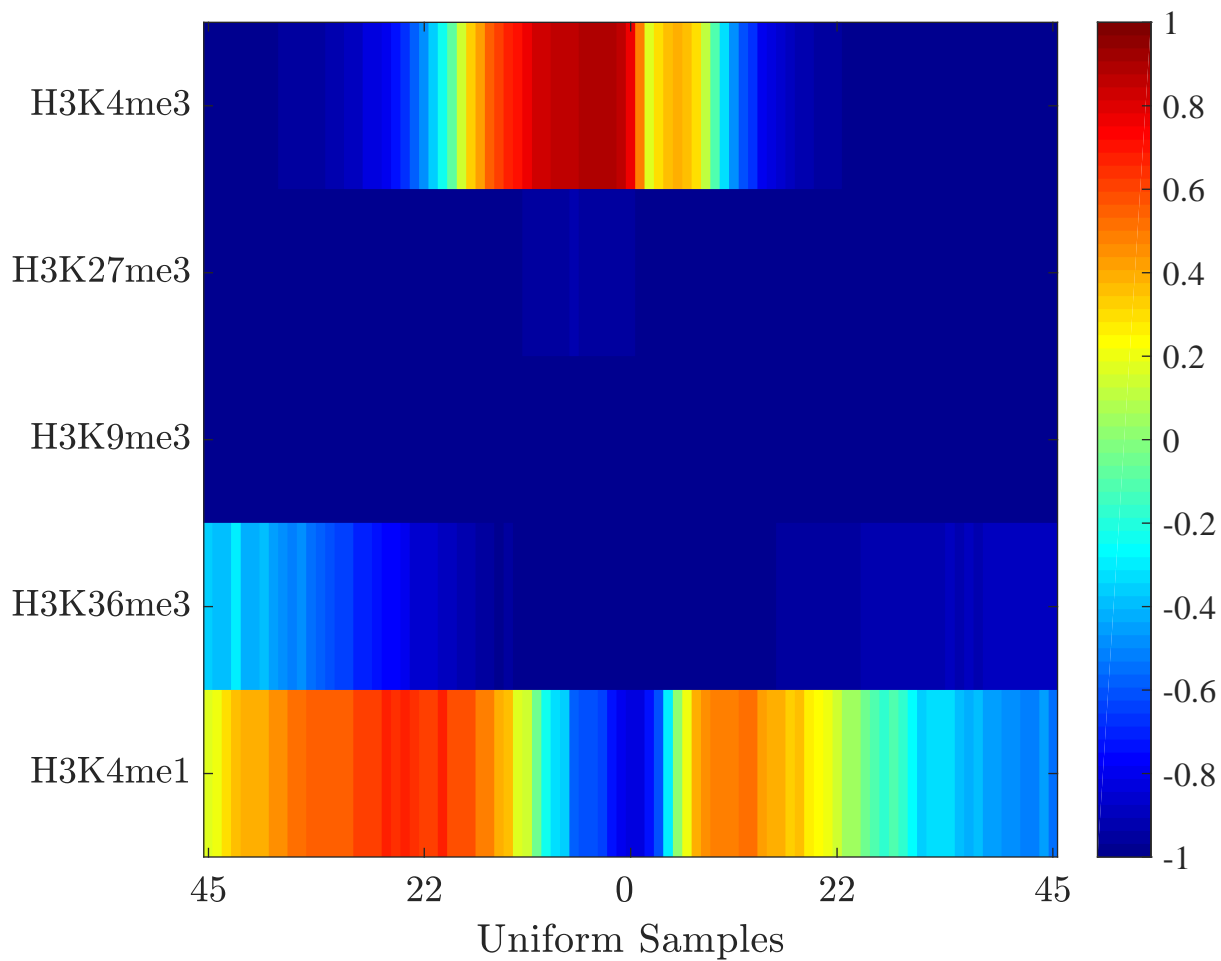

Supplement: Supplementary file 2 — HebbPlots of active promoters on the negative strand. This compressed file (.tar.gz) includes HebbPlots of promoters on the negative strand active in 57 tissues/cell types. (TAR 2952 kb) [file 12859_2018_2312_MOESM2_ESM.tar › file3/E028.pdf]

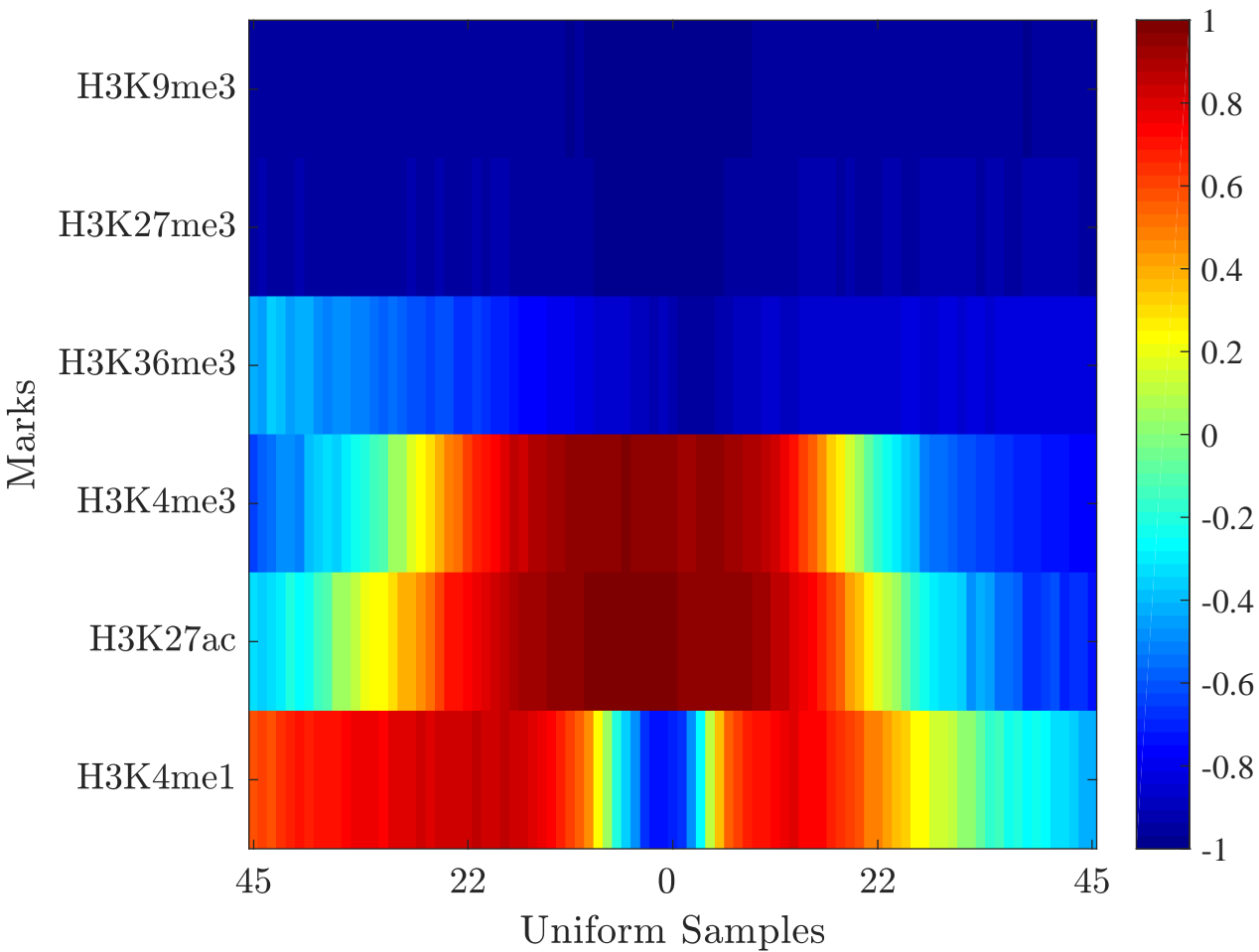

Supplement: Supplementary file 2 — HebbPlots of active promoters on the negative strand. This compressed file (.tar.gz) includes HebbPlots of promoters on the negative strand active in 57 tissues/cell types. (TAR 2952 kb) [file 12859_2018_2312_MOESM2_ESM.tar › file3/E037.pdf]

Marks

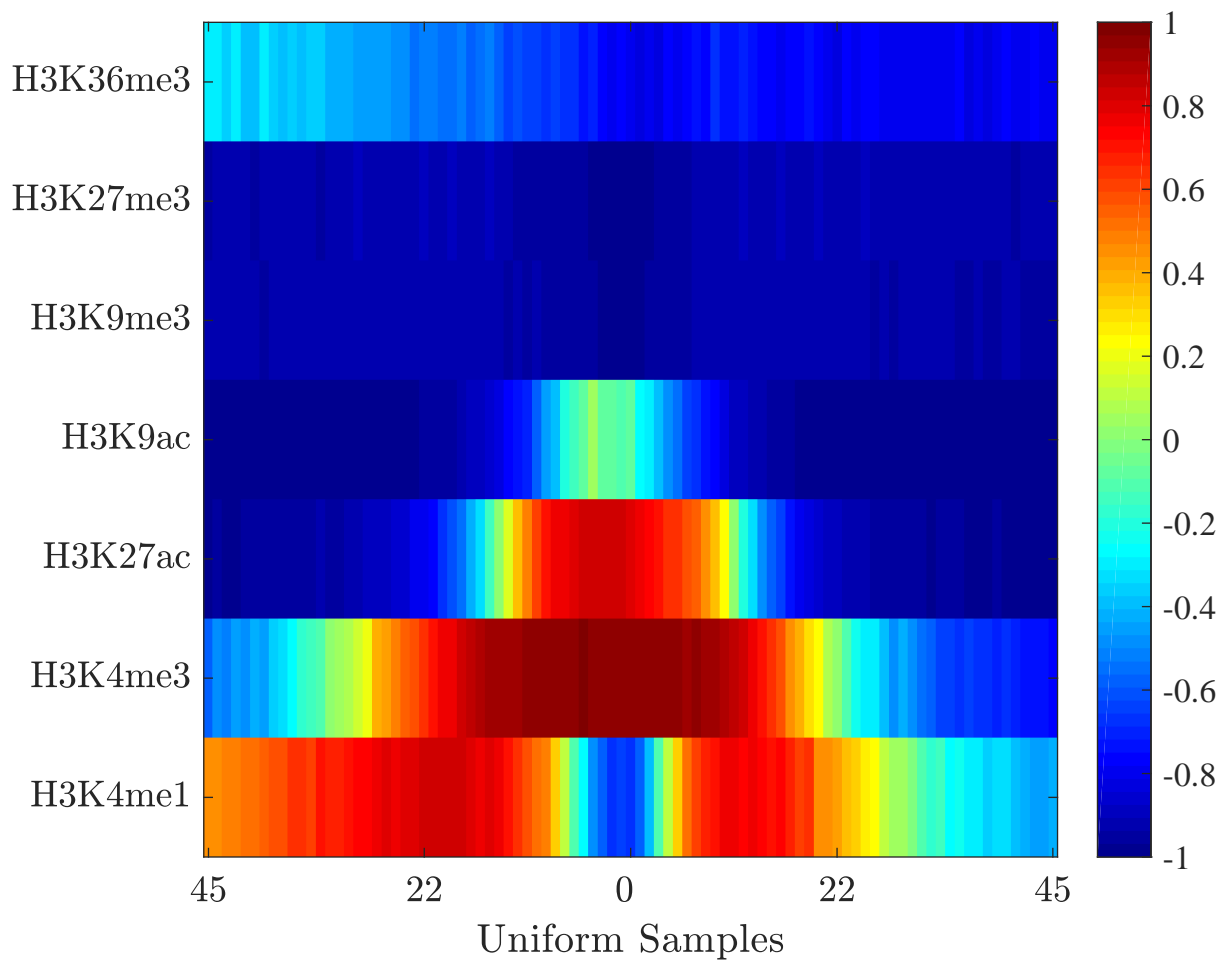

Supplement: Supplementary file 2 — HebbPlots of active promoters on the negative strand. This compressed file (.tar.gz) includes HebbPlots of promoters on the negative strand active in 57 tissues/cell types. (TAR 2952 kb) [file 12859_2018_2312_MOESM2_ESM.tar › file3/E038.pdf]

Marks

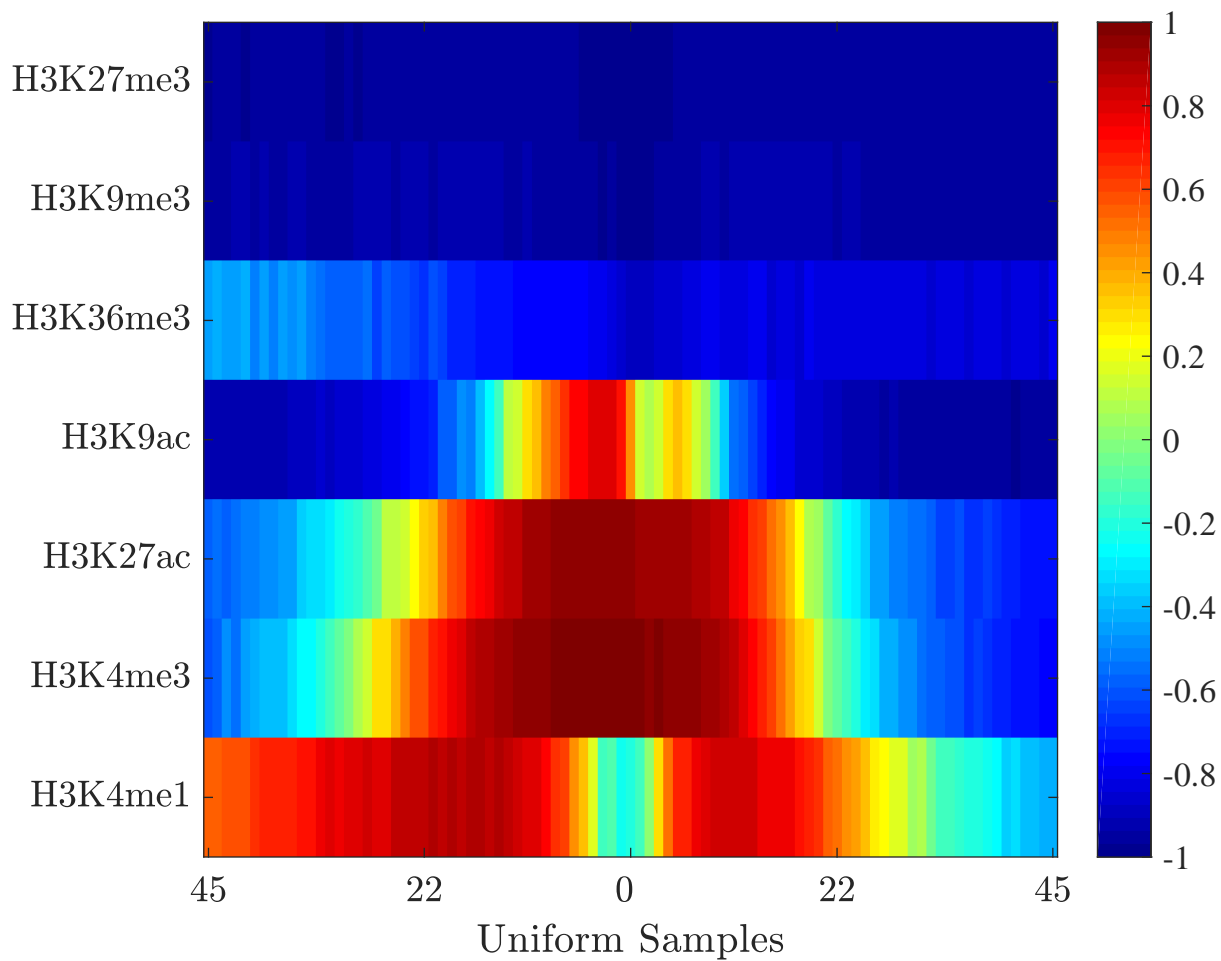

Supplement: Supplementary file 2 — HebbPlots of active promoters on the negative strand. This compressed file (.tar.gz) includes HebbPlots of promoters on the negative strand active in 57 tissues/cell types. (TAR 2952 kb) [file 12859_2018_2312_MOESM2_ESM.tar › file3/E047.pdf]

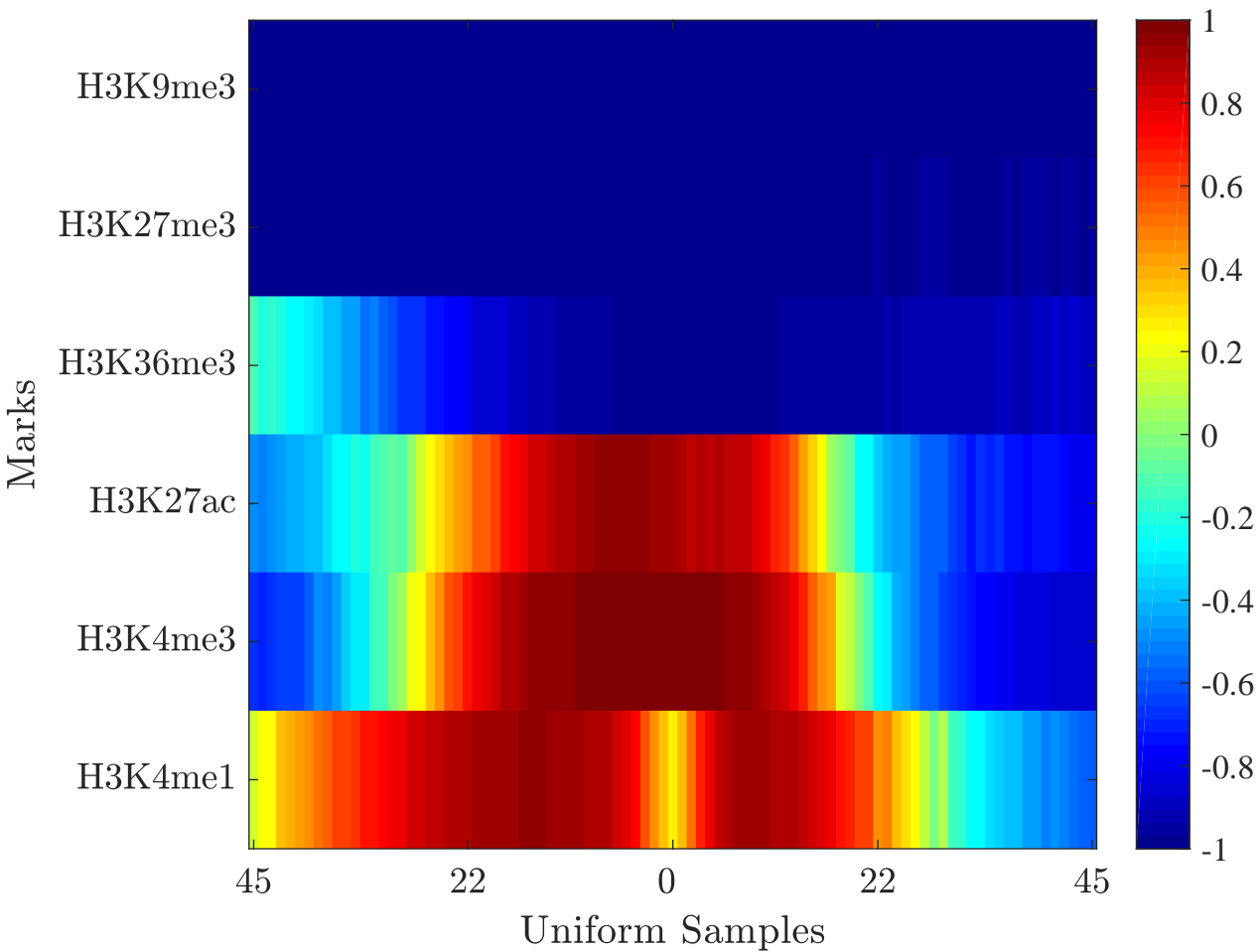

Supplement: Supplementary file 2 — HebbPlots of active promoters on the negative strand. This compressed file (.tar.gz) includes HebbPlots of promoters on the negative strand active in 57 tissues/cell types. (TAR 2952 kb) [file 12859_2018_2312_MOESM2_ESM.tar › file3/E050.pdf]

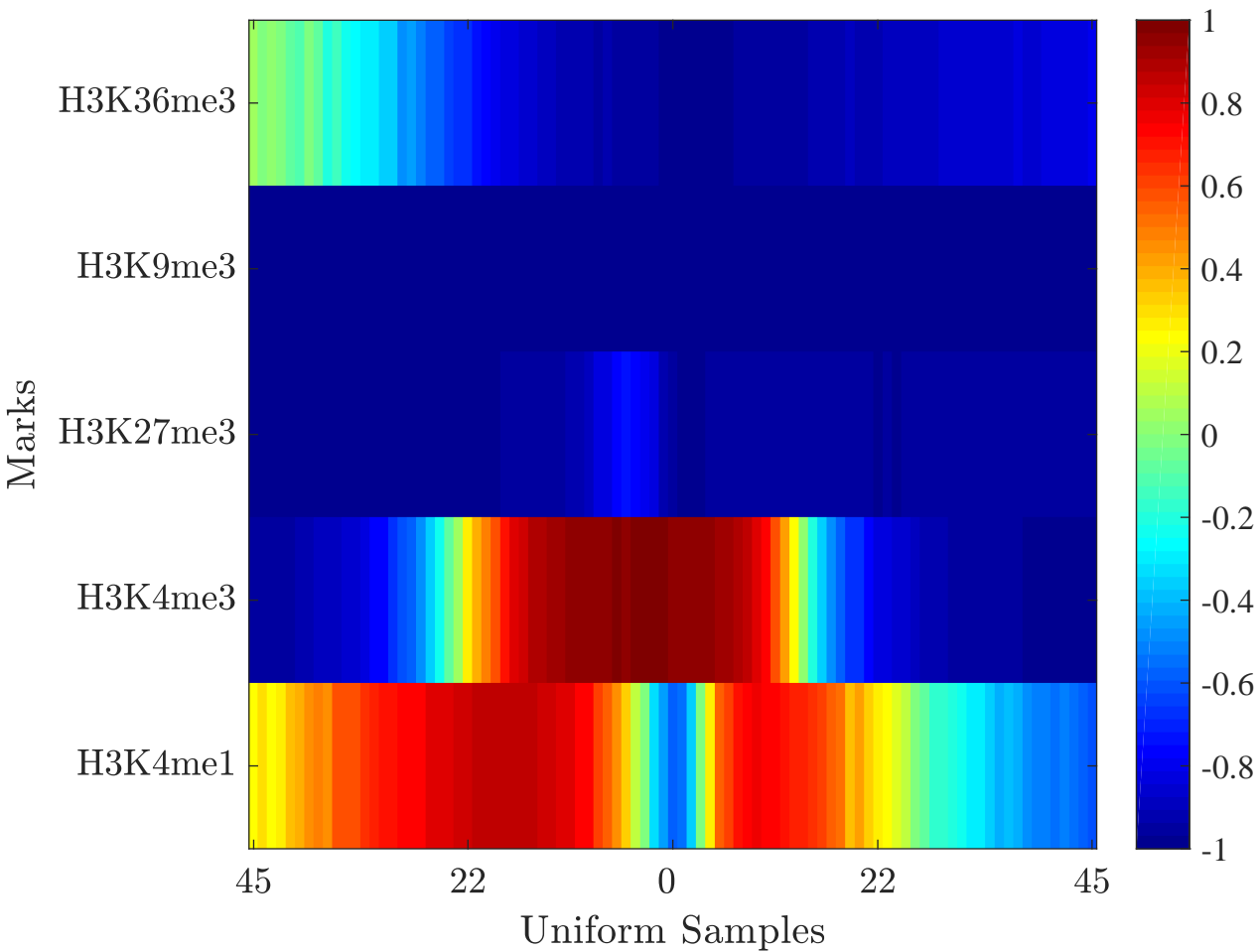

Supplement: Supplementary file 2 — HebbPlots of active promoters on the negative strand. This compressed file (.tar.gz) includes HebbPlots of promoters on the negative strand active in 57 tissues/cell types. (TAR 2952 kb) [file 12859_2018_2312_MOESM2_ESM.tar › file3/E053.pdf]

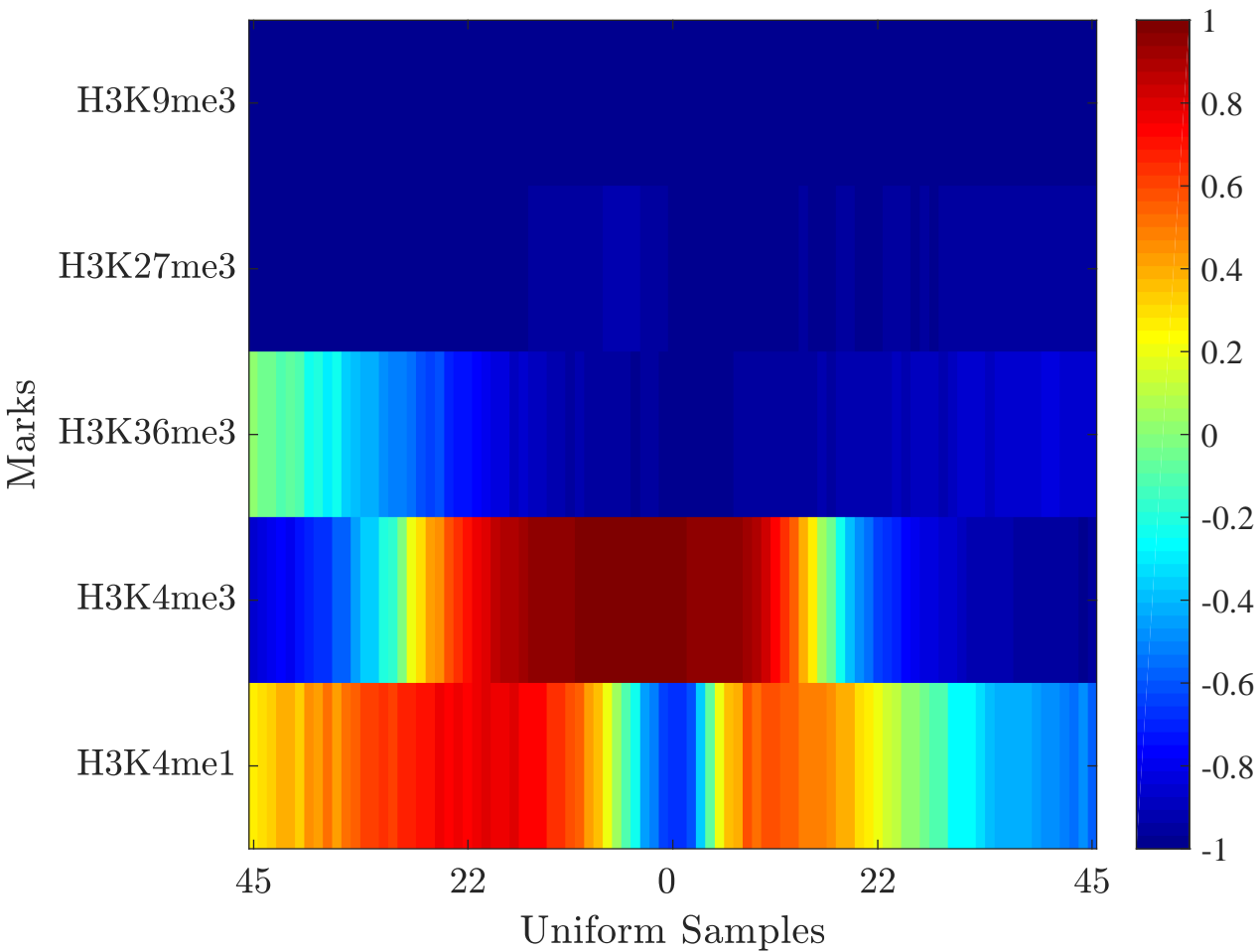

Supplement: Supplementary file 2 — HebbPlots of active promoters on the negative strand. This compressed file (.tar.gz) includes HebbPlots of promoters on the negative strand active in 57 tissues/cell types. (TAR 2952 kb) [file 12859_2018_2312_MOESM2_ESM.tar › file3/E054.pdf]

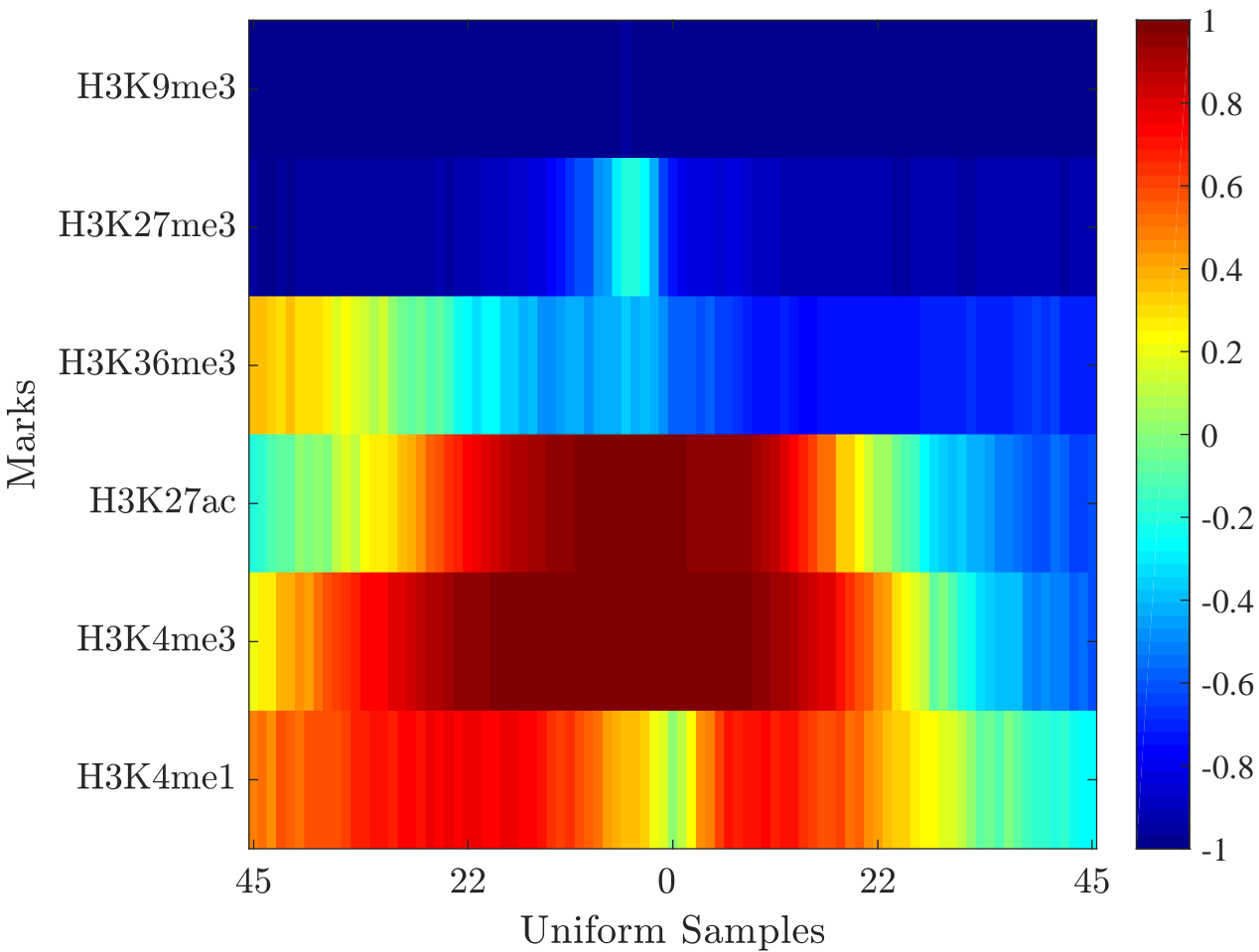

Supplement: Supplementary file 2 — HebbPlots of active promoters on the negative strand. This compressed file (.tar.gz) includes HebbPlots of promoters on the negative strand active in 57 tissues/cell types. (TAR 2952 kb) [file 12859_2018_2312_MOESM2_ESM.tar › file3/E055.pdf]

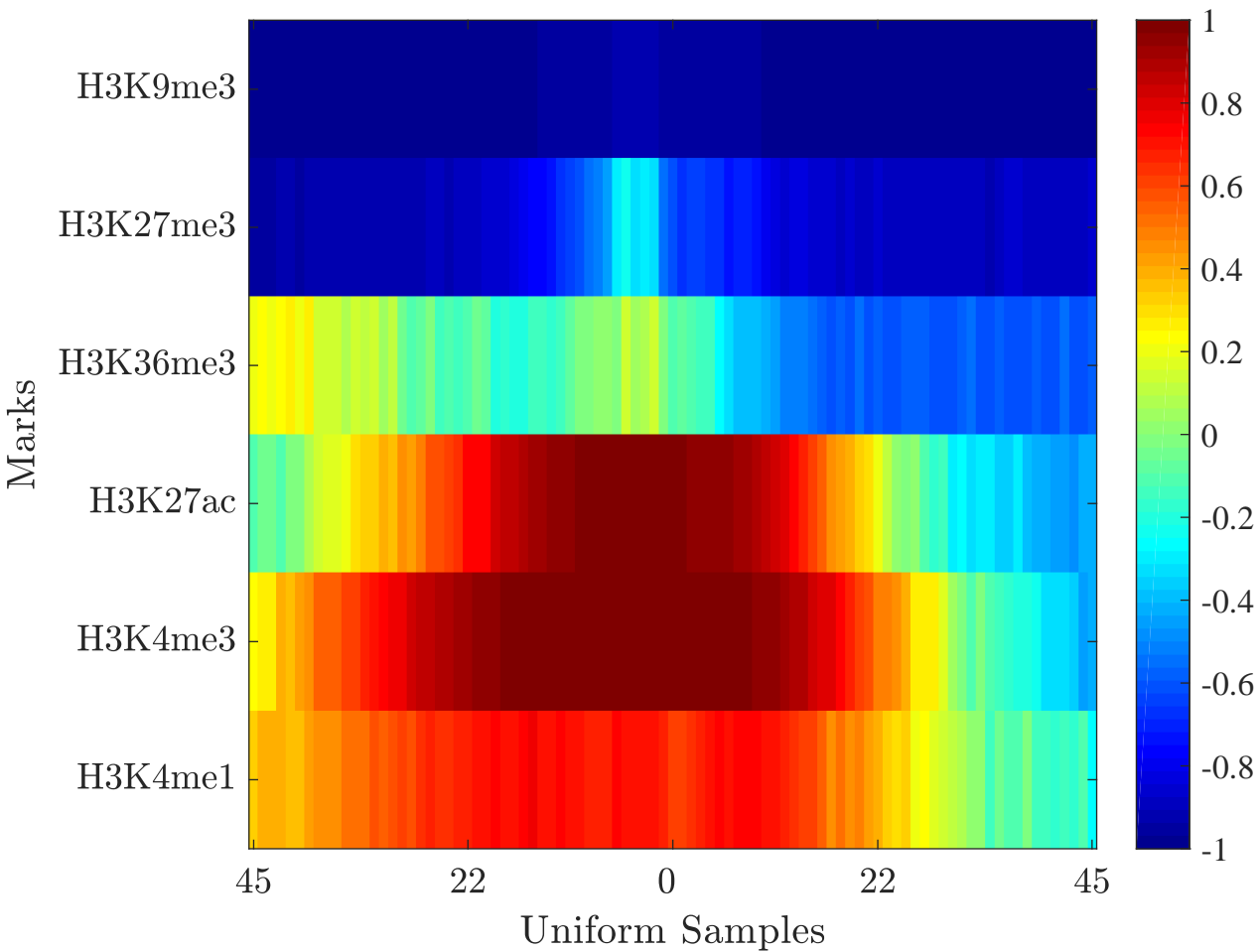

Supplement: Supplementary file 2 — HebbPlots of active promoters on the negative strand. This compressed file (.tar.gz) includes HebbPlots of promoters on the negative strand active in 57 tissues/cell types. (TAR 2952 kb) [file 12859_2018_2312_MOESM2_ESM.tar › file3/E056.pdf]

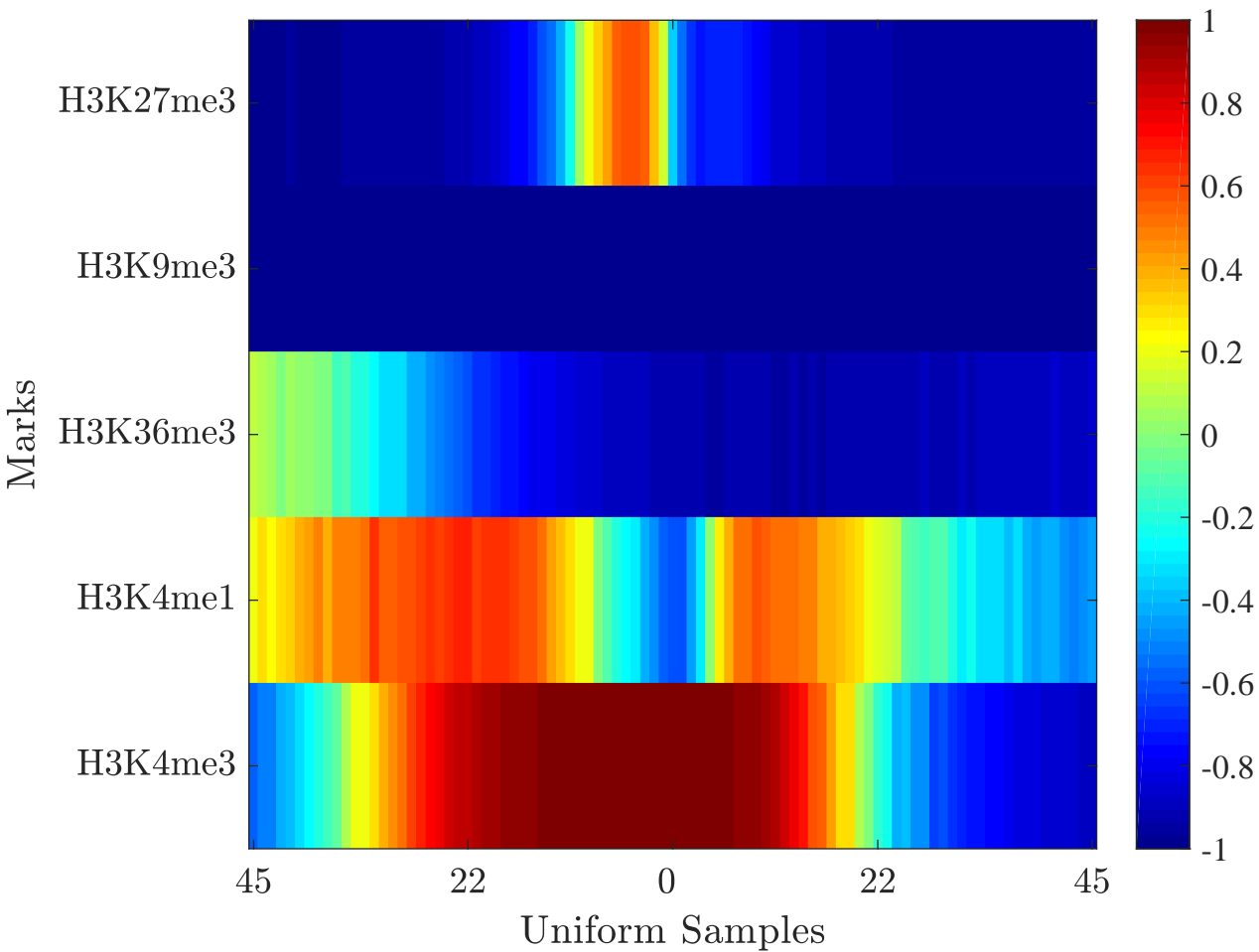

Supplement: Supplementary file 2 — HebbPlots of active promoters on the negative strand. This compressed file (.tar.gz) includes HebbPlots of promoters on the negative strand active in 57 tissues/cell types. (TAR 2952 kb) [file 12859_2018_2312_MOESM2_ESM.tar › file3/E057.pdf]

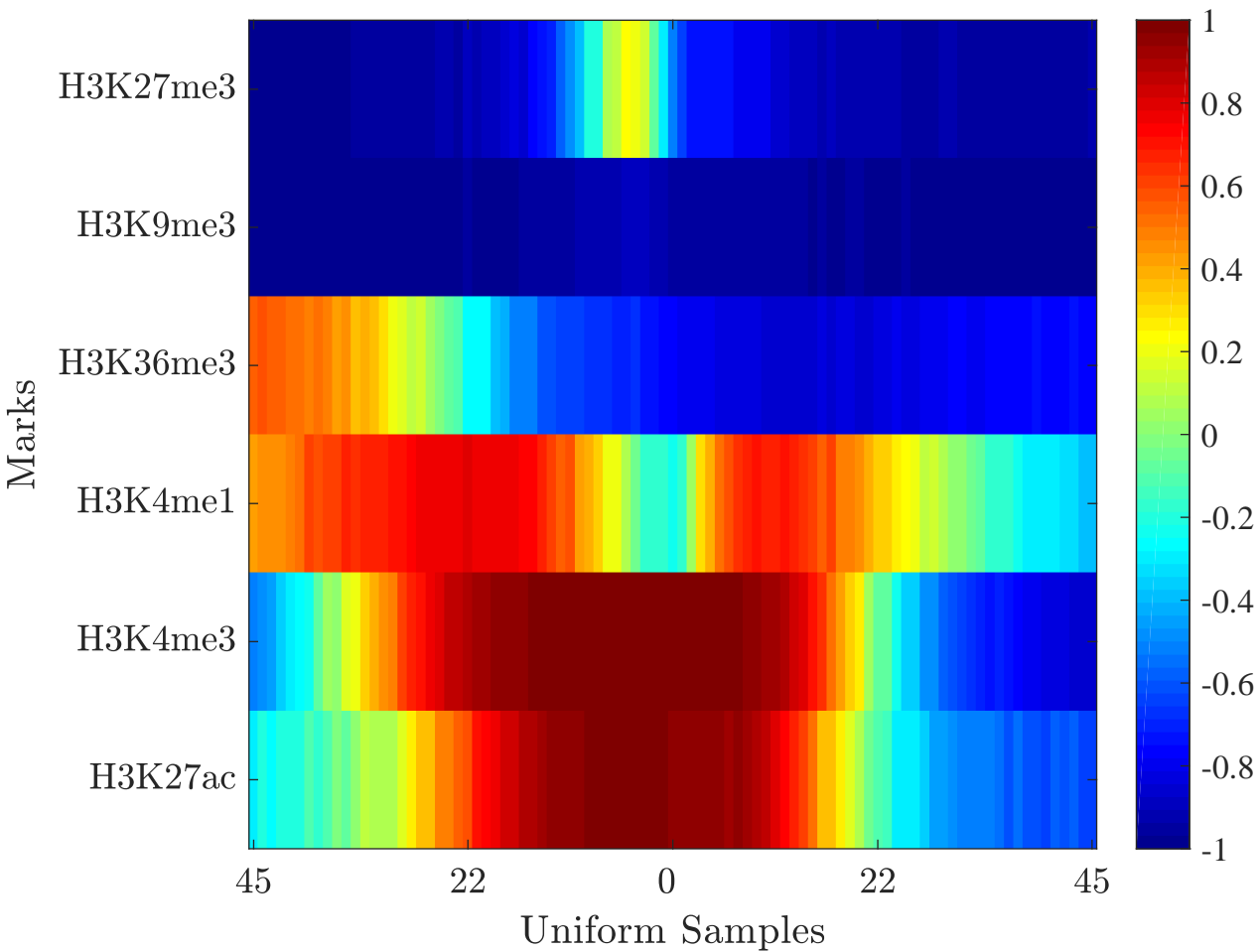

Supplement: Supplementary file 2 — HebbPlots of active promoters on the negative strand. This compressed file (.tar.gz) includes HebbPlots of promoters on the negative strand active in 57 tissues/cell types. (TAR 2952 kb) [file 12859_2018_2312_MOESM2_ESM.tar › file3/E058.pdf]

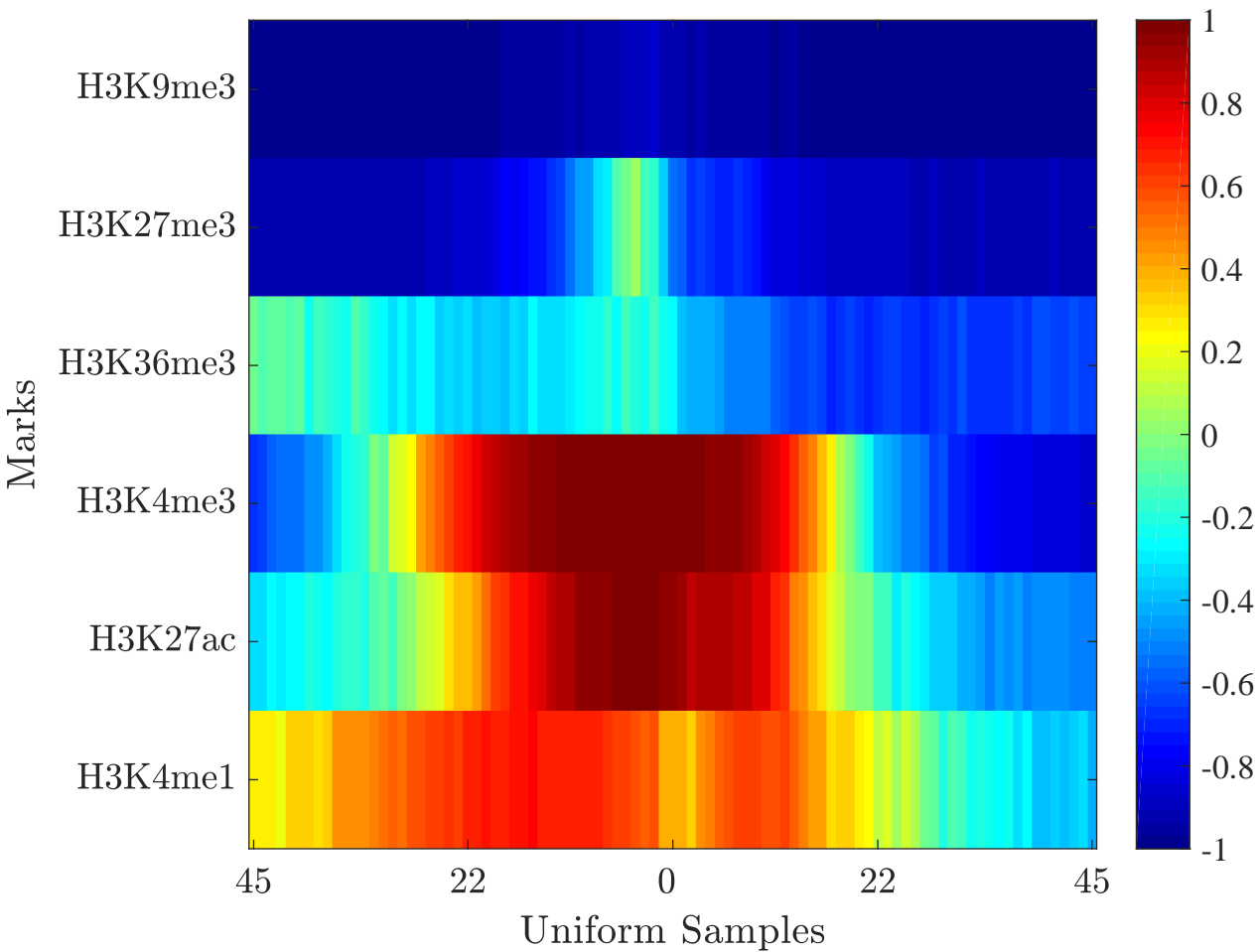

Supplement: Supplementary file 2 — HebbPlots of active promoters on the negative strand. This compressed file (.tar.gz) includes HebbPlots of promoters on the negative strand active in 57 tissues/cell types. (TAR 2952 kb) [file 12859_2018_2312_MOESM2_ESM.tar › file3/E059.pdf]

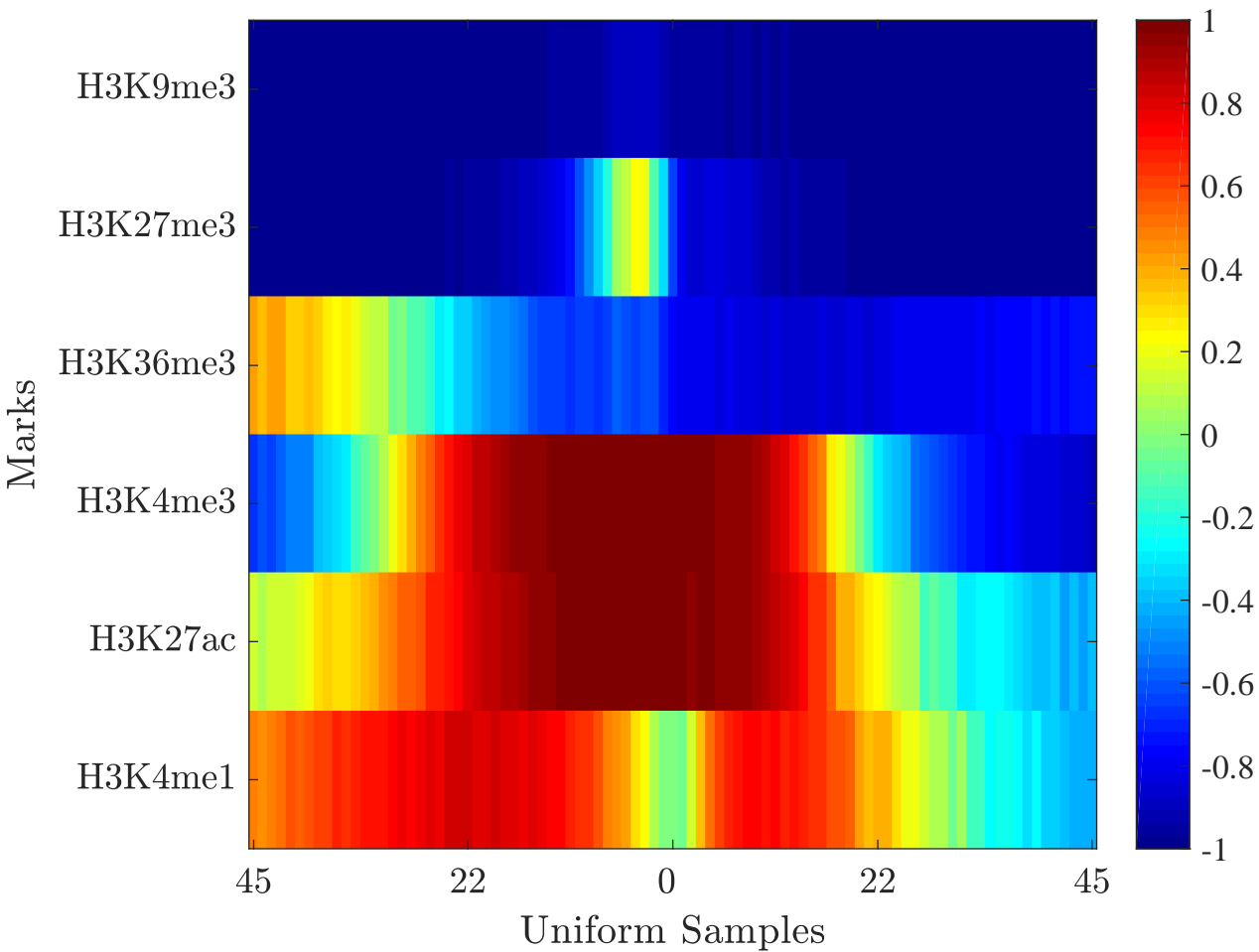

Supplement: Supplementary file 2 — HebbPlots of active promoters on the negative strand. This compressed file (.tar.gz) includes HebbPlots of promoters on the negative strand active in 57 tissues/cell types. (TAR 2952 kb) [file 12859_2018_2312_MOESM2_ESM.tar › file3/E061.pdf]

Marks

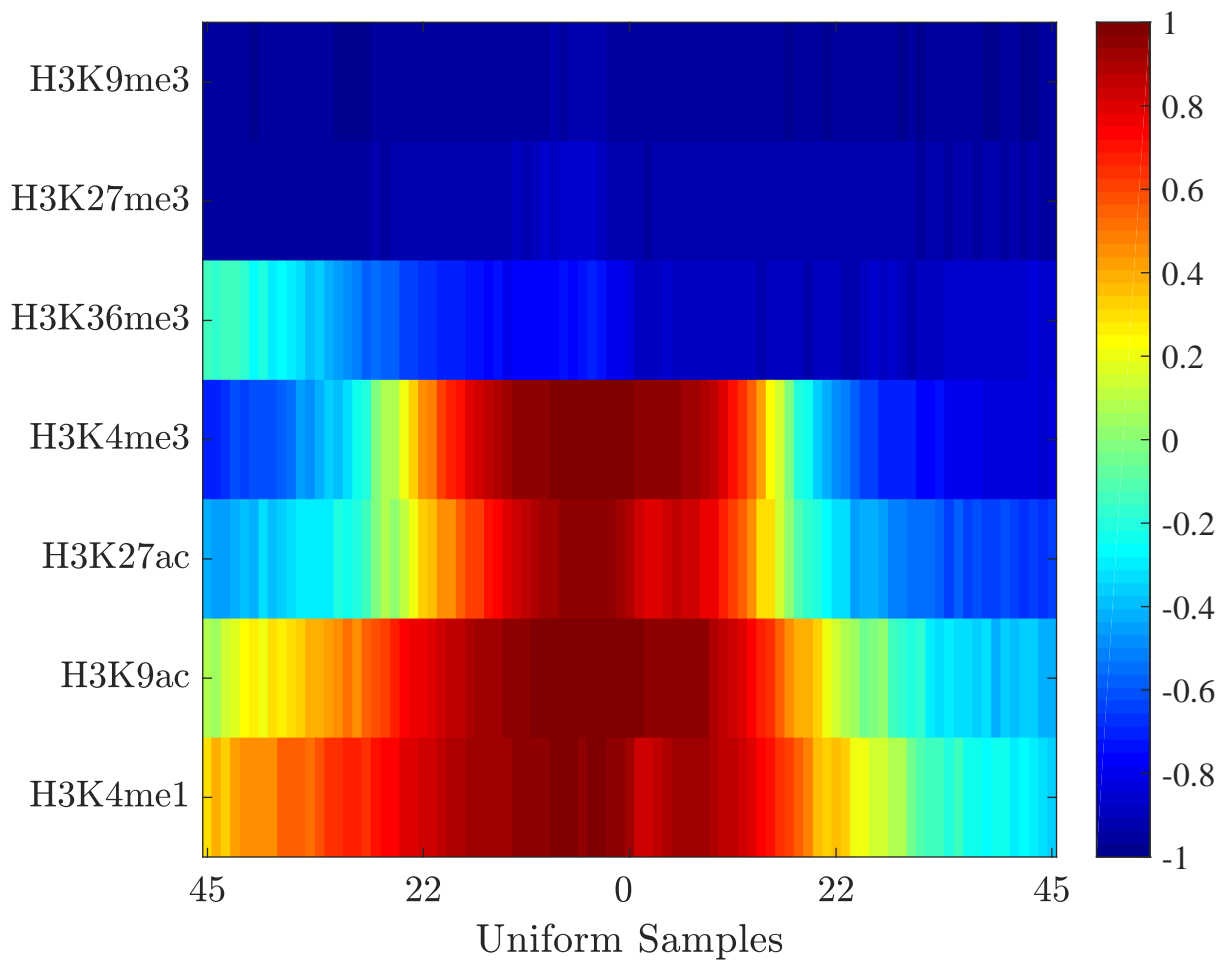

Supplement: Supplementary file 2 — HebbPlots of active promoters on the negative strand. This compressed file (.tar.gz) includes HebbPlots of promoters on the negative strand active in 57 tissues/cell types. (TAR 2952 kb) [file 12859_2018_2312_MOESM2_ESM.tar › file3/E062.pdf]

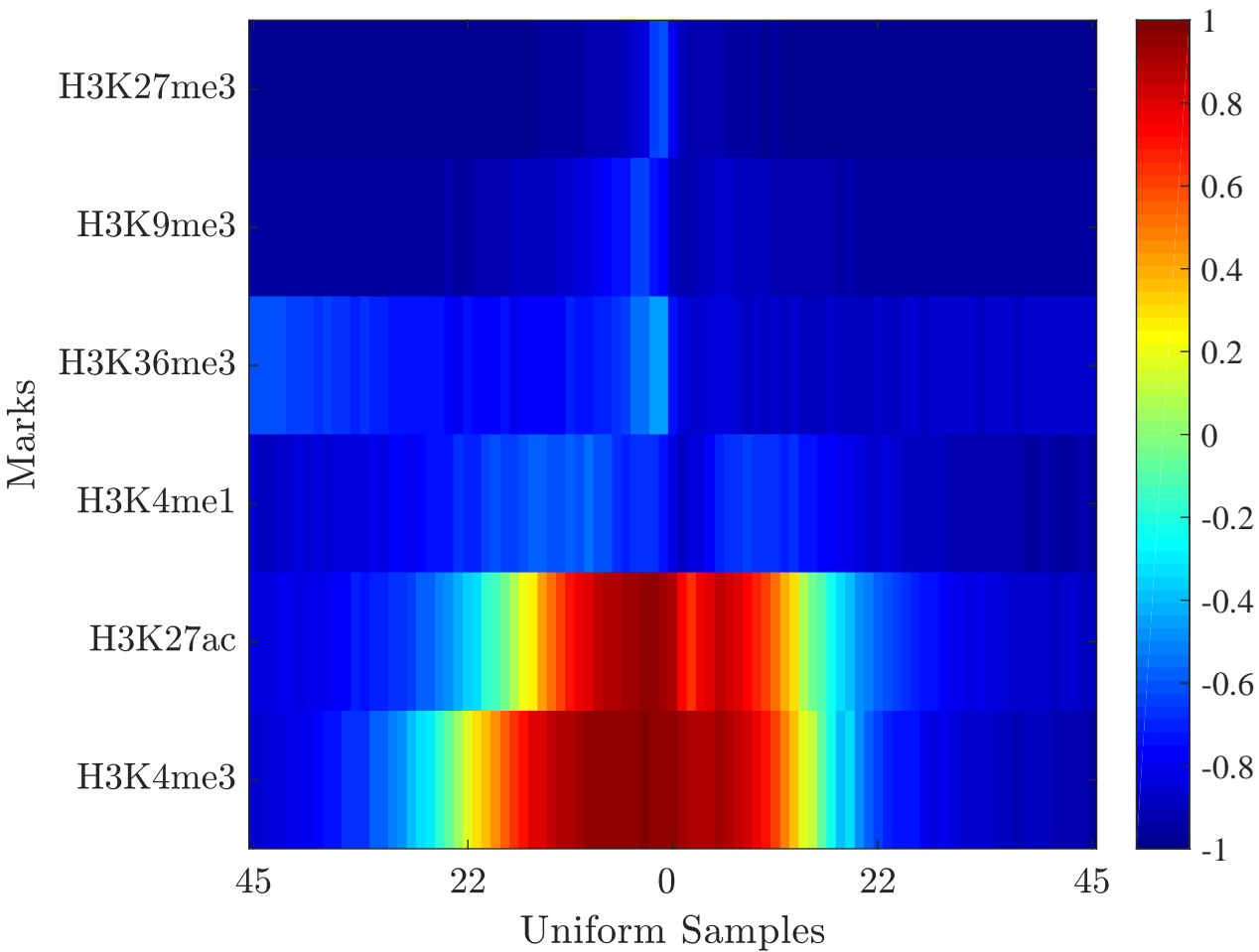

Supplement: Supplementary file 2 — HebbPlots of active promoters on the negative strand. This compressed file (.tar.gz) includes HebbPlots of promoters on the negative strand active in 57 tissues/cell types. (TAR 2952 kb) [file 12859_2018_2312_MOESM2_ESM.tar › file3/E065.pdf]

Marks

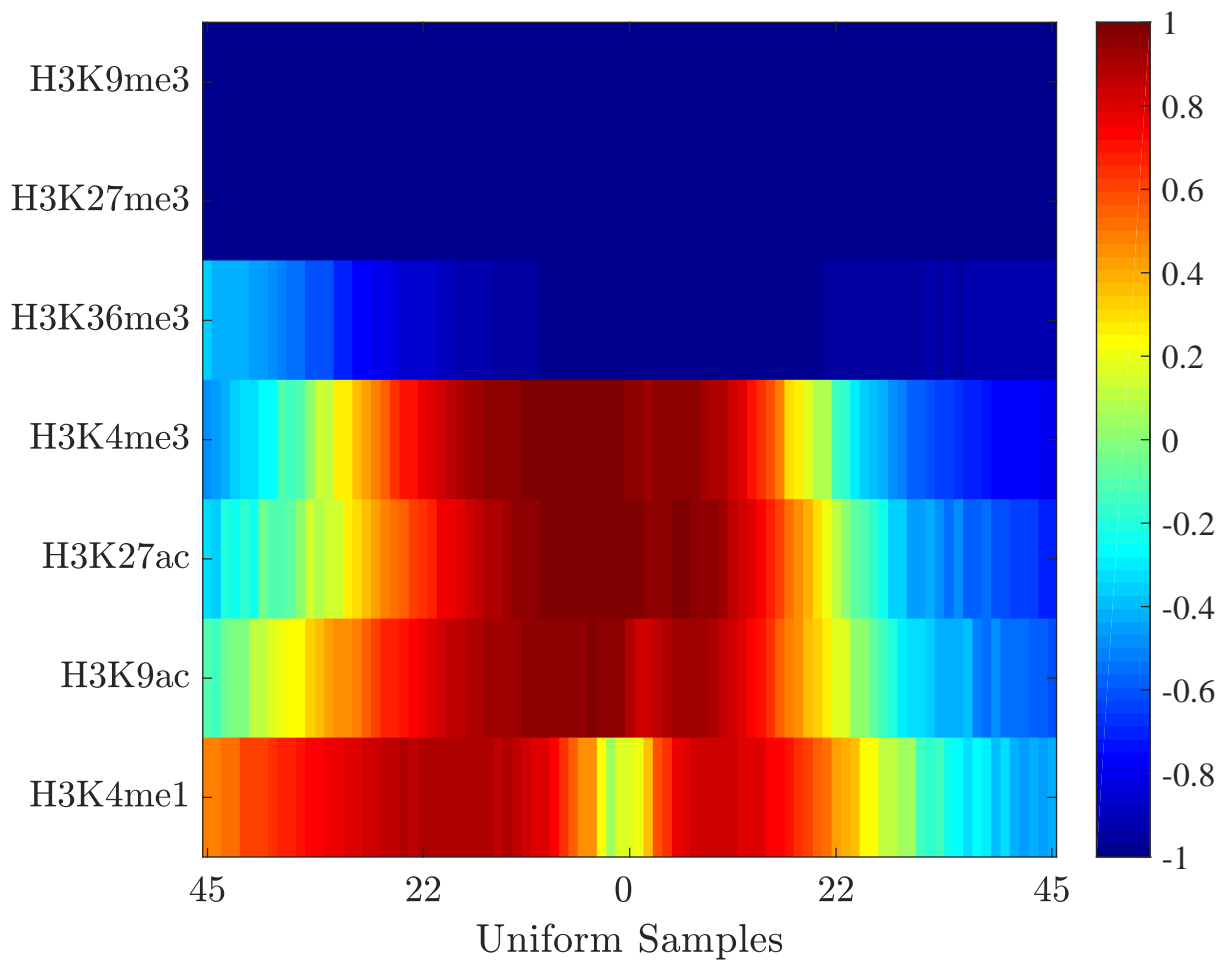

Supplement: Supplementary file 2 — HebbPlots of active promoters on the negative strand. This compressed file (.tar.gz) includes HebbPlots of promoters on the negative strand active in 57 tissues/cell types. (TAR 2952 kb) [file 12859_2018_2312_MOESM2_ESM.tar › file3/E066.pdf]

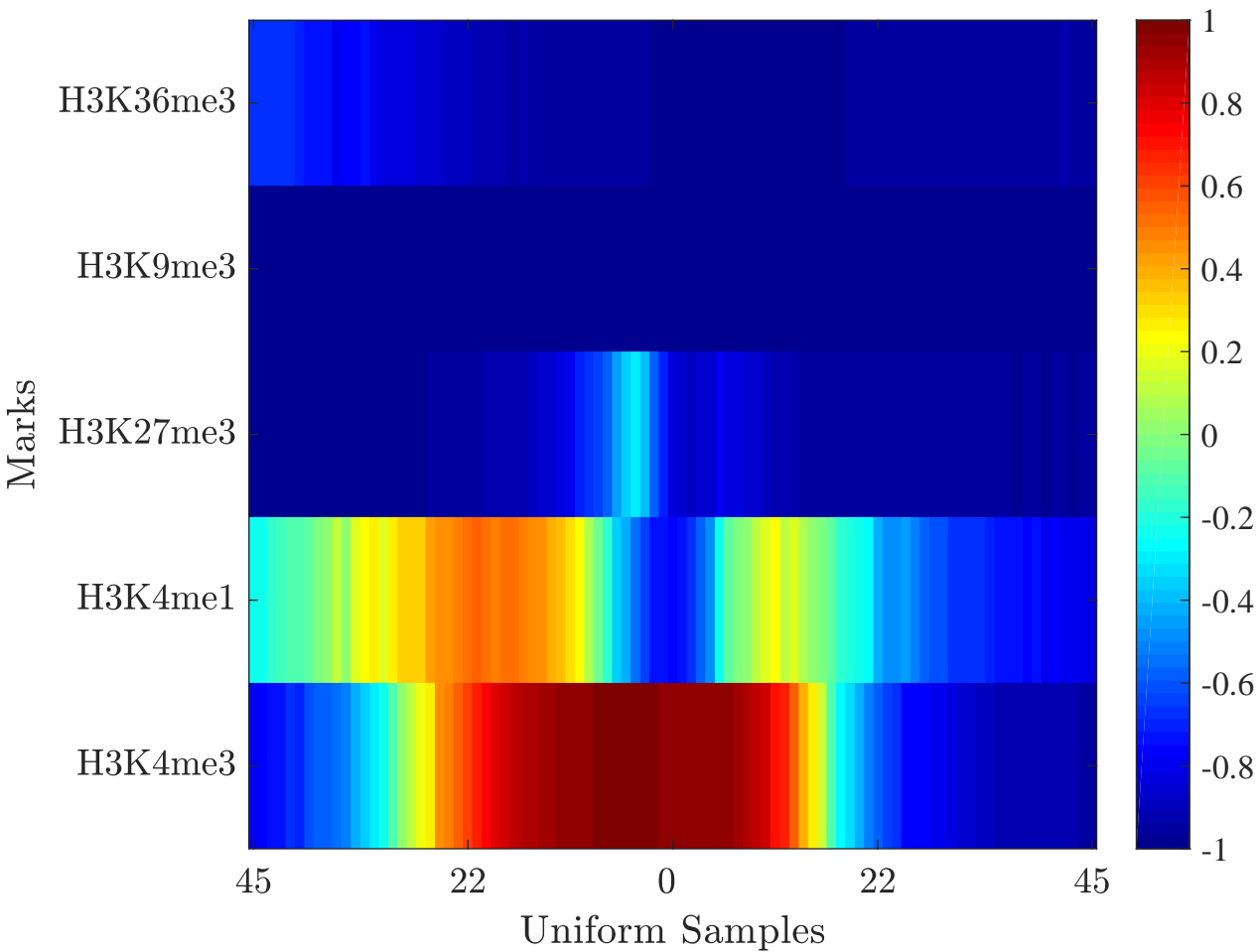

Supplement: Supplementary file 2 — HebbPlots of active promoters on the negative strand. This compressed file (.tar.gz) includes HebbPlots of promoters on the negative strand active in 57 tissues/cell types. (TAR 2952 kb) [file 12859_2018_2312_MOESM2_ESM.tar › file3/E070.pdf]

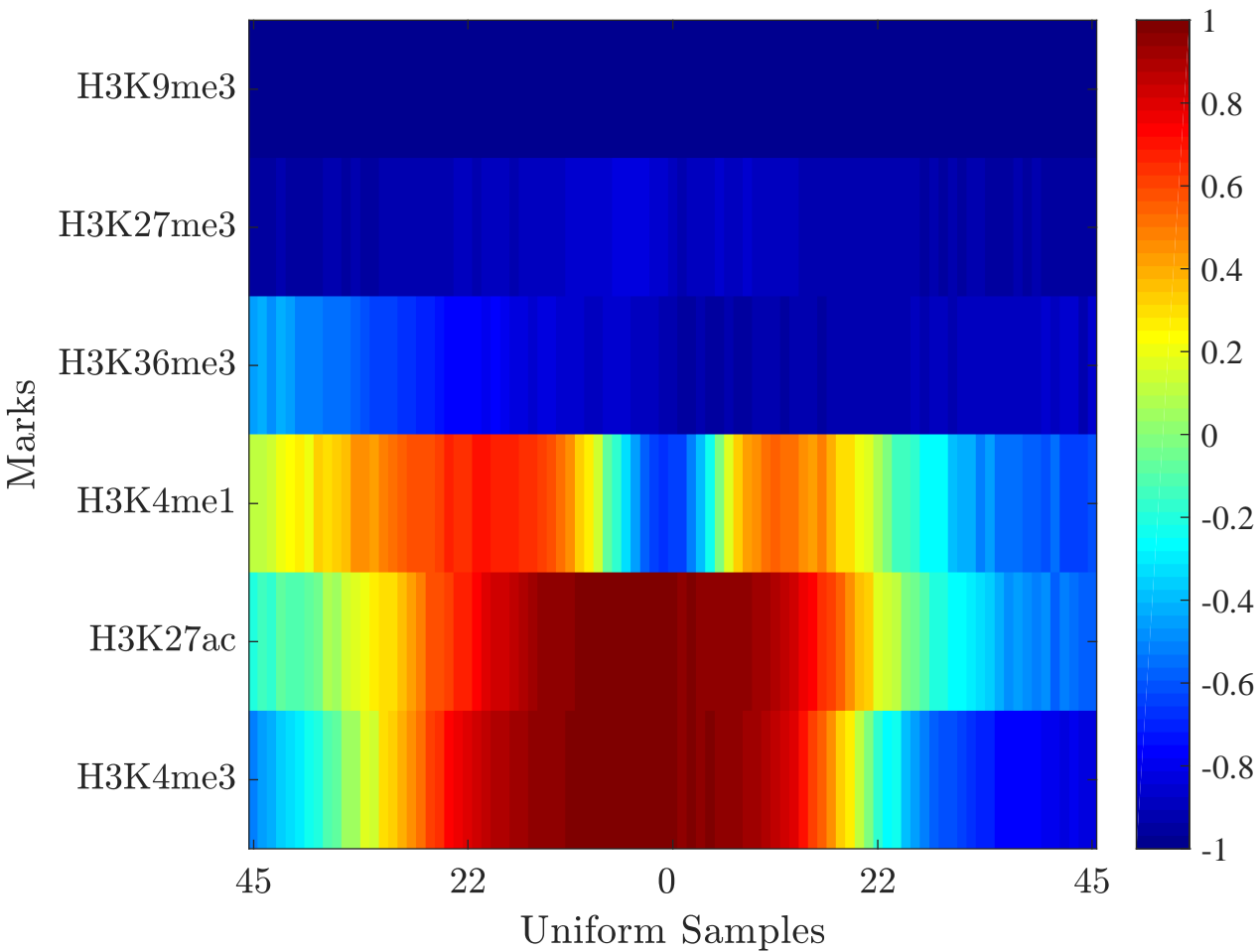

Supplement: Supplementary file 2 — HebbPlots of active promoters on the negative strand. This compressed file (.tar.gz) includes HebbPlots of promoters on the negative strand active in 57 tissues/cell types. (TAR 2952 kb) [file 12859_2018_2312_MOESM2_ESM.tar › file3/E071.pdf]

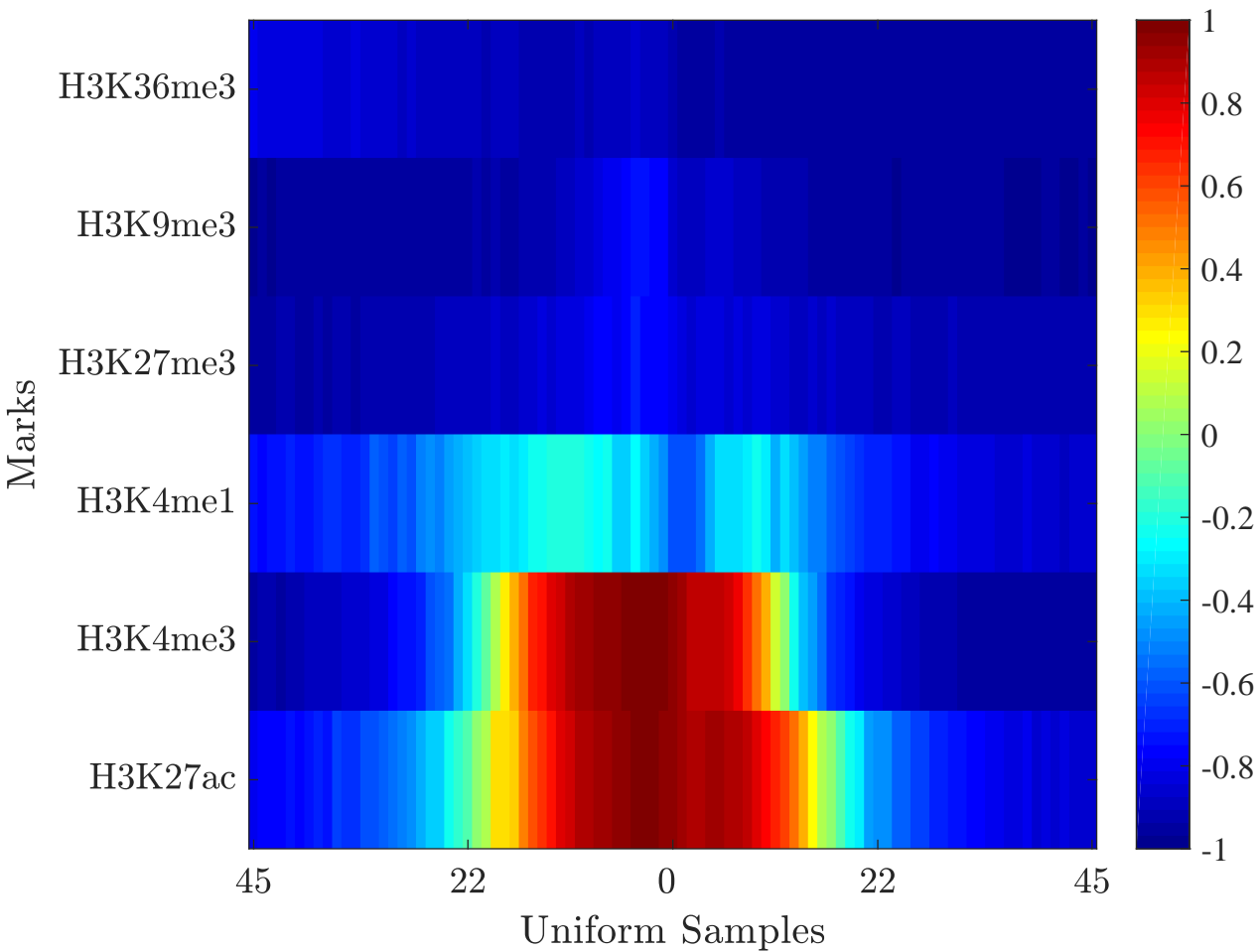

Supplement: Supplementary file 2 — HebbPlots of active promoters on the negative strand. This compressed file (.tar.gz) includes HebbPlots of promoters on the negative strand active in 57 tissues/cell types. (TAR 2952 kb) [file 12859_2018_2312_MOESM2_ESM.tar › file3/E079.pdf]

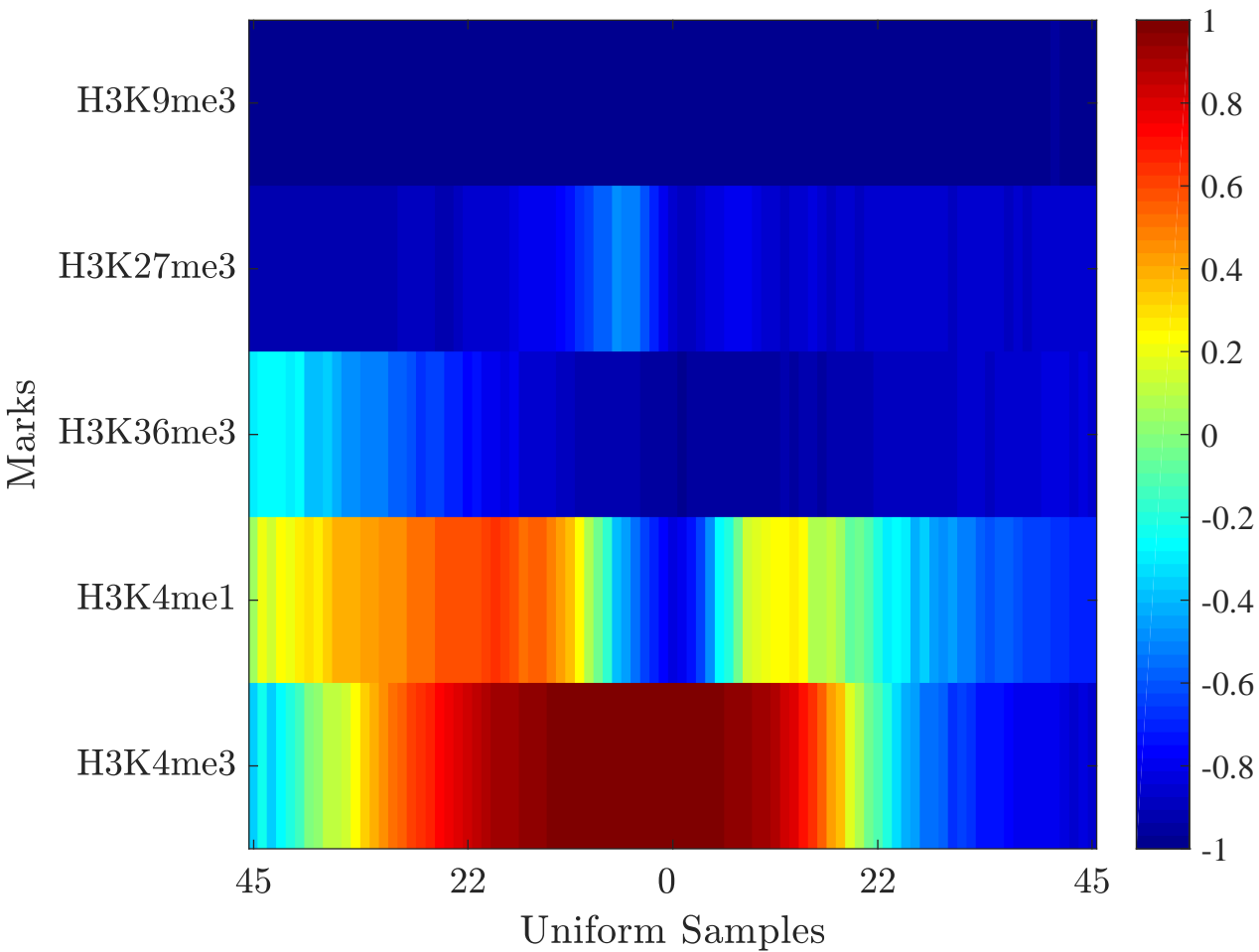

Supplement: Supplementary file 2 — HebbPlots of active promoters on the negative strand. This compressed file (.tar.gz) includes HebbPlots of promoters on the negative strand active in 57 tissues/cell types. (TAR 2952 kb) [file 12859_2018_2312_MOESM2_ESM.tar › file3/E082.pdf]

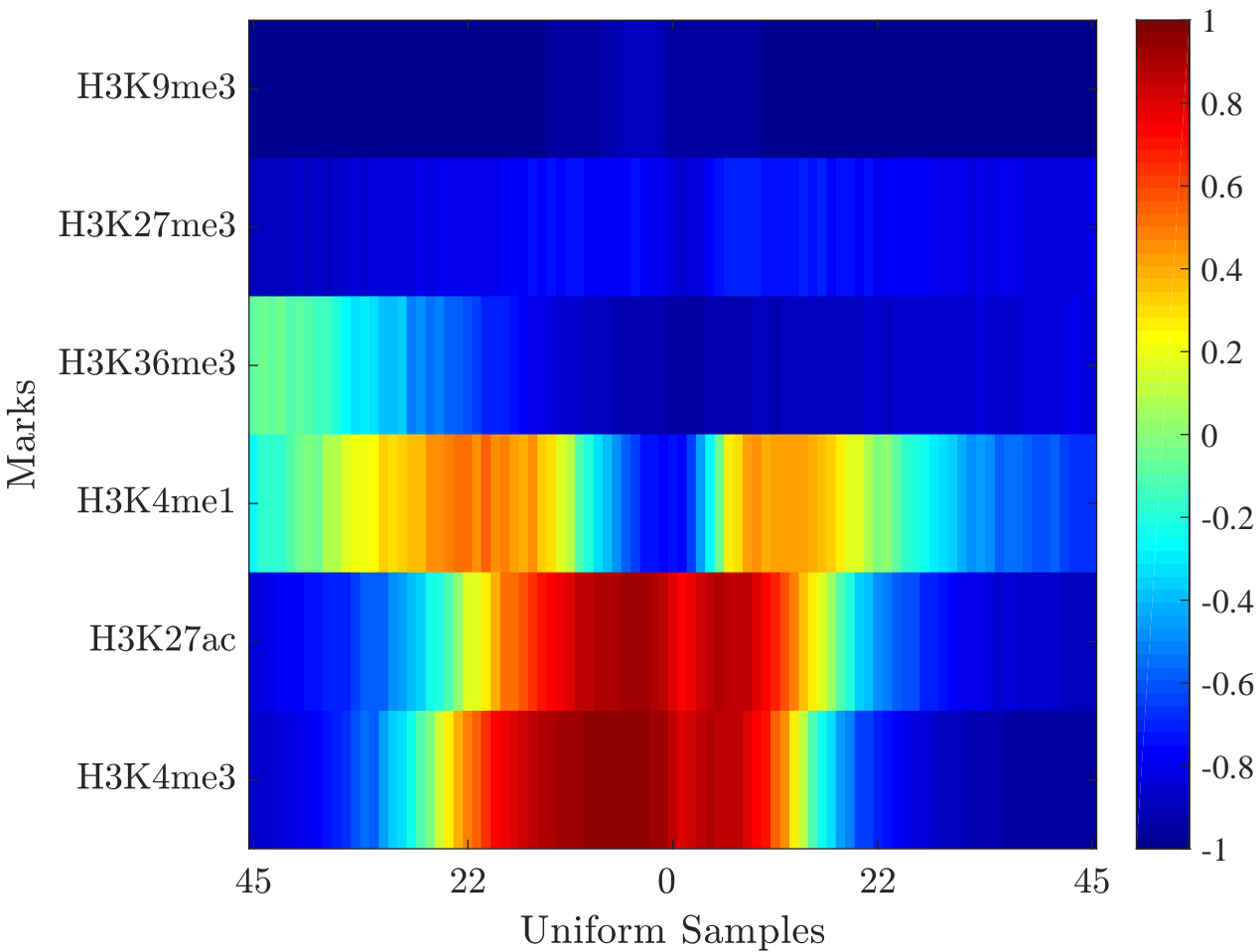

Supplement: Supplementary file 2 — HebbPlots of active promoters on the negative strand. This compressed file (.tar.gz) includes HebbPlots of promoters on the negative strand active in 57 tissues/cell types. (TAR 2952 kb) [file 12859_2018_2312_MOESM2_ESM.tar › file3/E084.pdf]

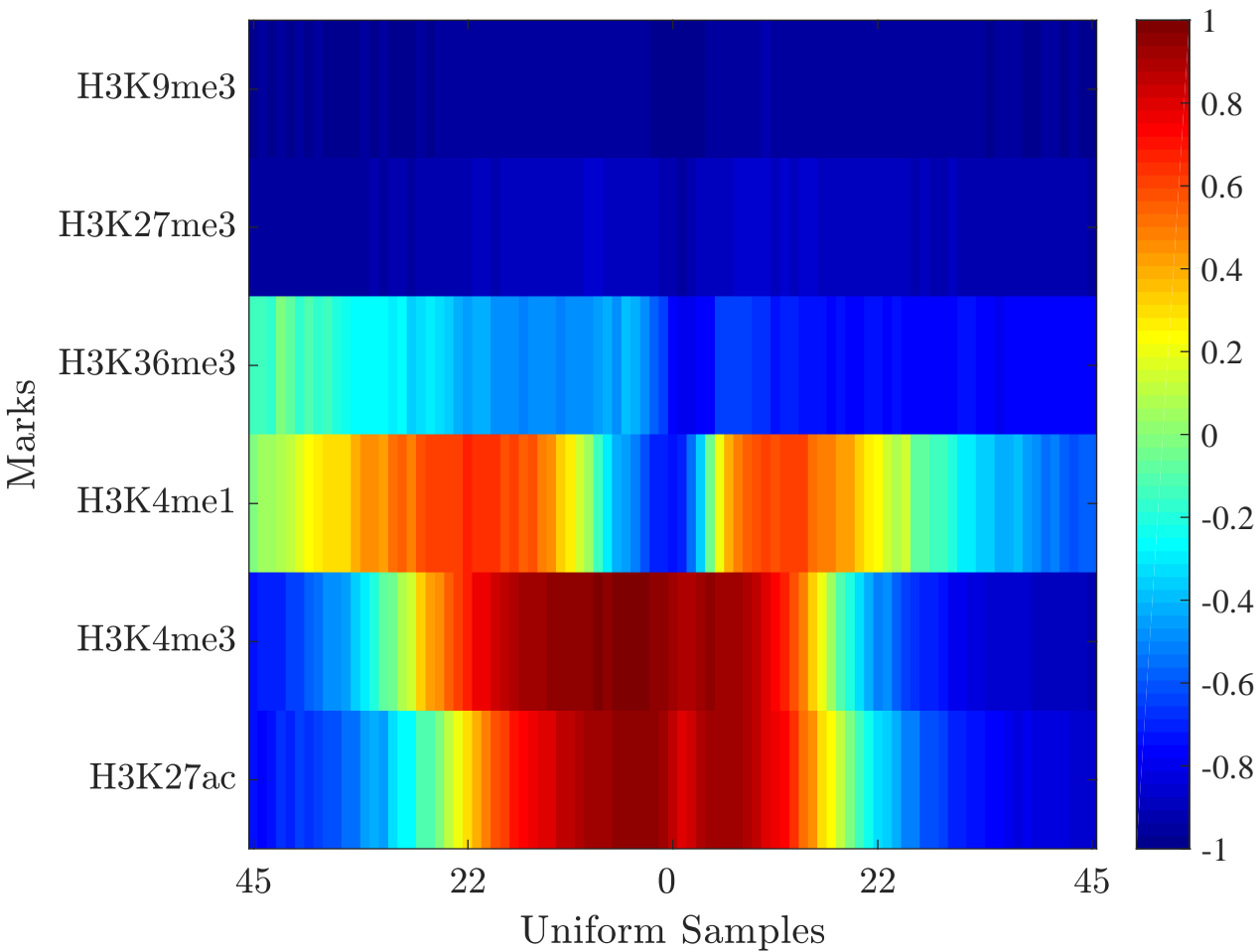

Supplement: Supplementary file 2 — HebbPlots of active promoters on the negative strand. This compressed file (.tar.gz) includes HebbPlots of promoters on the negative strand active in 57 tissues/cell types. (TAR 2952 kb) [file 12859_2018_2312_MOESM2_ESM.tar › file3/E085.pdf]

Marks

H3K36me3

H3K27me3

H3K9me3

H3K4me1

H3K9ac

H3K27ac

H3K4me3

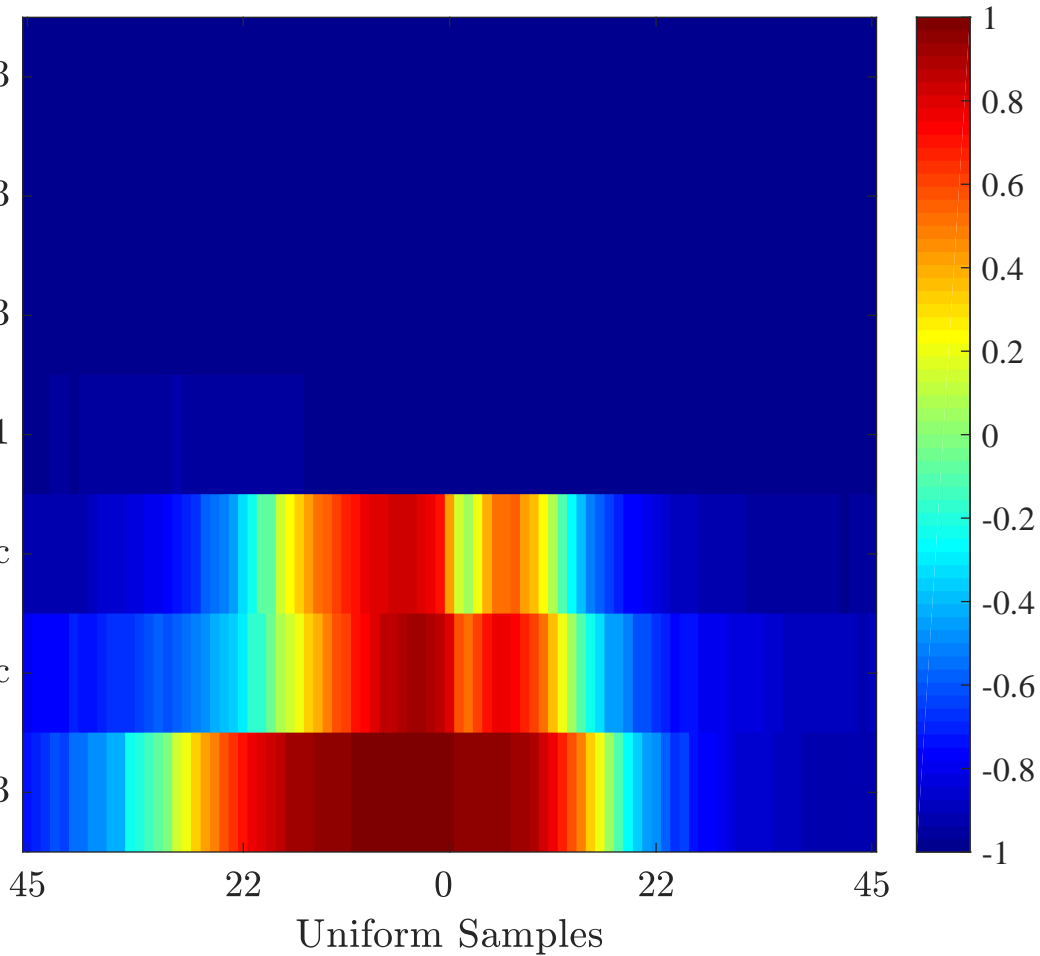

Supplement: Supplementary file 2 — HebbPlots of active promoters on the negative strand. This compressed file (.tar.gz) includes HebbPlots of promoters on the negative strand active in 57 tissues/cell types. (TAR 2952 kb) [file 12859_2018_2312_MOESM2_ESM.tar › file3/E087.pdf]

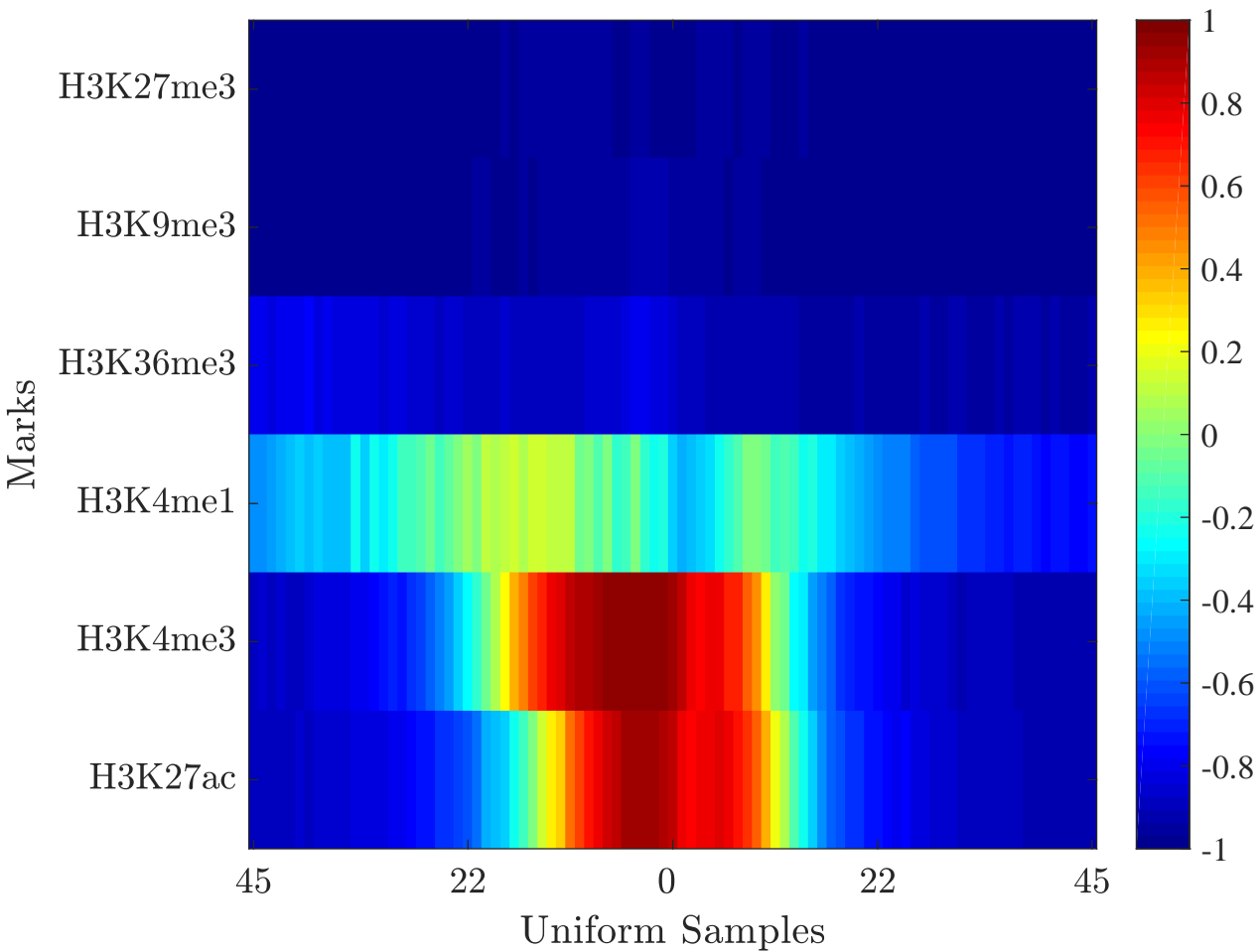

Supplement: Supplementary file 2 — HebbPlots of active promoters on the negative strand. This compressed file (.tar.gz) includes HebbPlots of promoters on the negative strand active in 57 tissues/cell types. (TAR 2952 kb) [file 12859_2018_2312_MOESM2_ESM.tar › file3/E094.pdf]

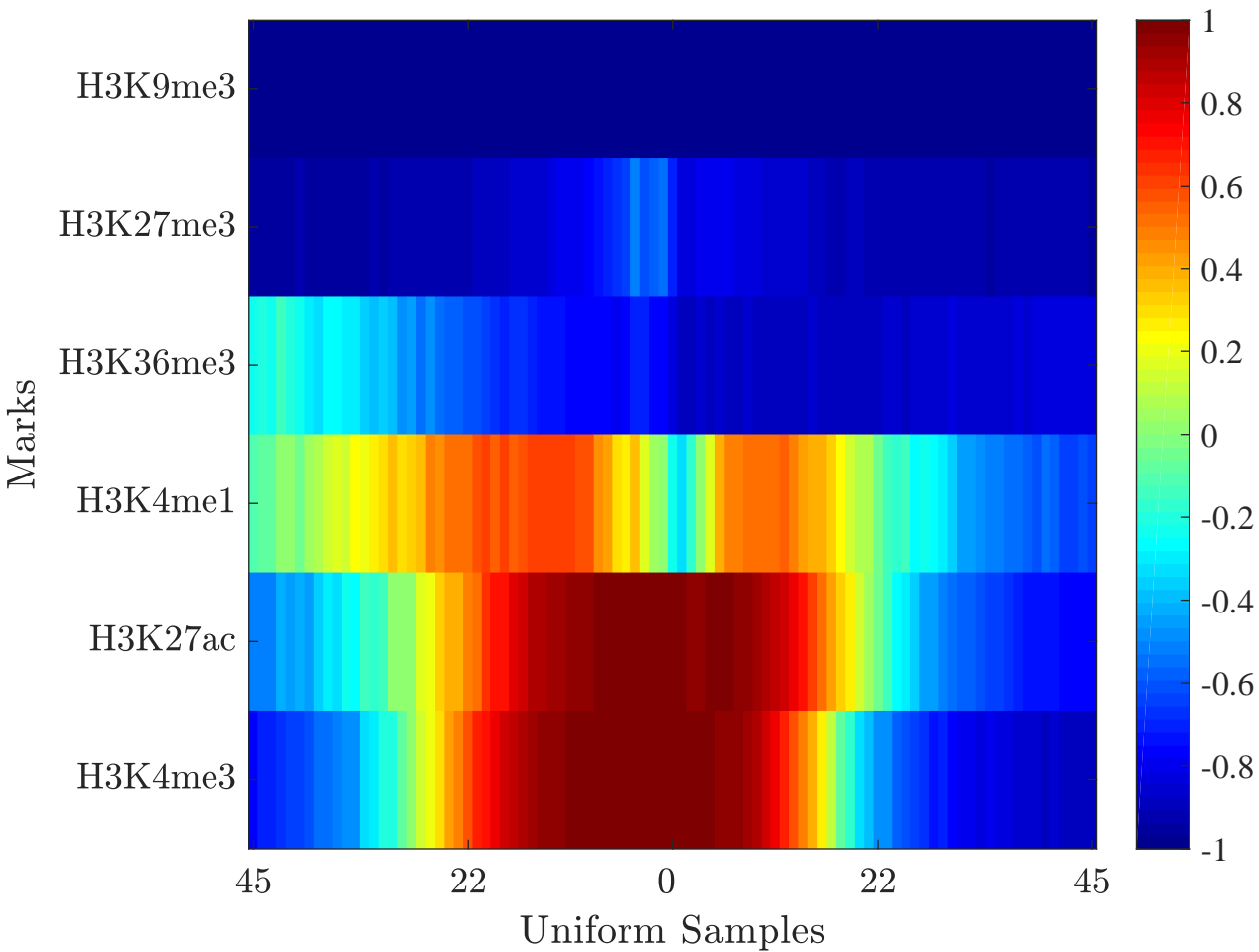

Supplement: Supplementary file 2 — HebbPlots of active promoters on the negative strand. This compressed file (.tar.gz) includes HebbPlots of promoters on the negative strand active in 57 tissues/cell types. (TAR 2952 kb) [file 12859_2018_2312_MOESM2_ESM.tar › file3/E095.pdf]

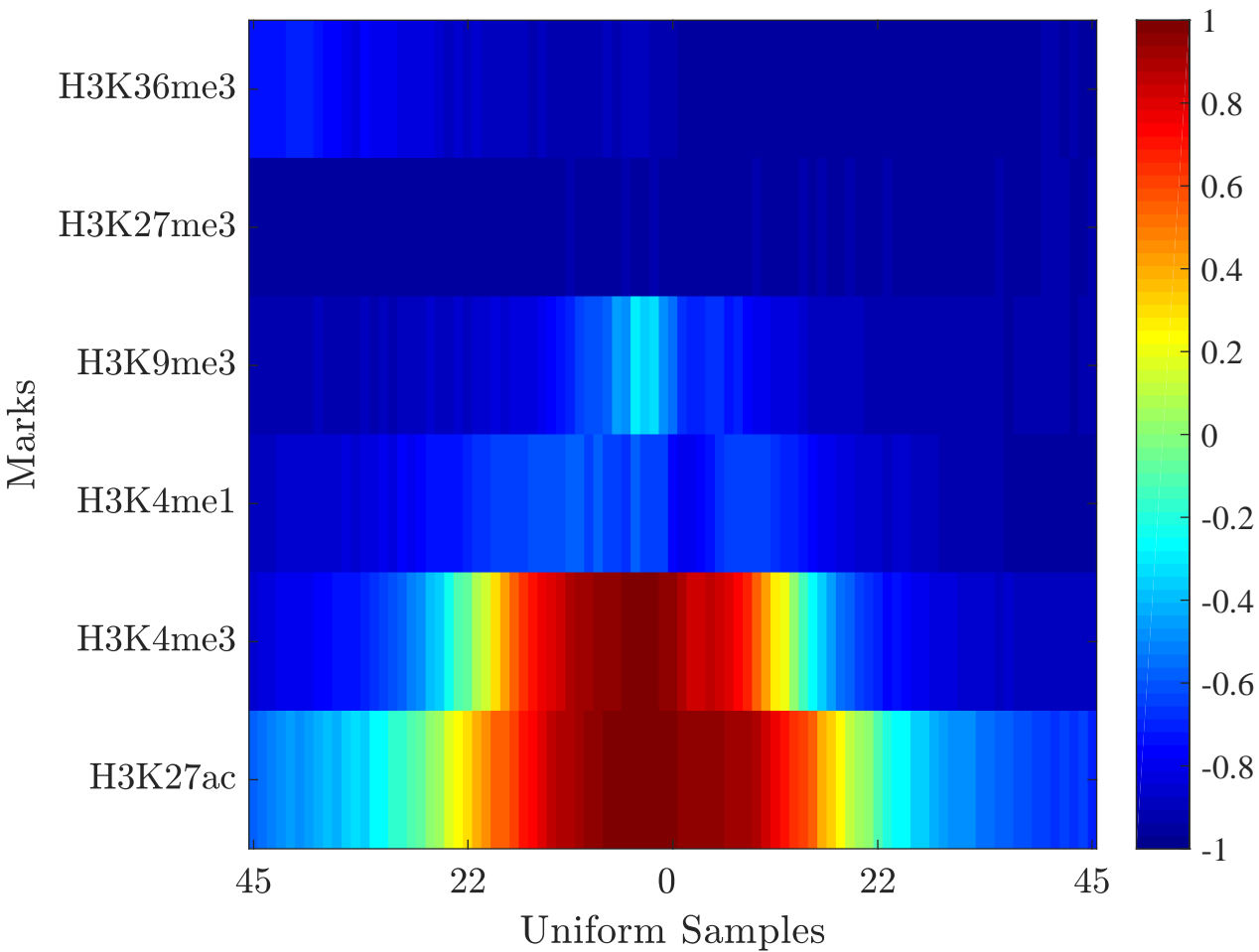

Supplement: Supplementary file 2 — HebbPlots of active promoters on the negative strand. This compressed file (.tar.gz) includes HebbPlots of promoters on the negative strand active in 57 tissues/cell types. (TAR 2952 kb) [file 12859_2018_2312_MOESM2_ESM.tar › file3/E096.pdf]

Marks

H3K27me3

H3K36me3

H3K9me3

H3K27ac

H3K4me3

H3K4me1

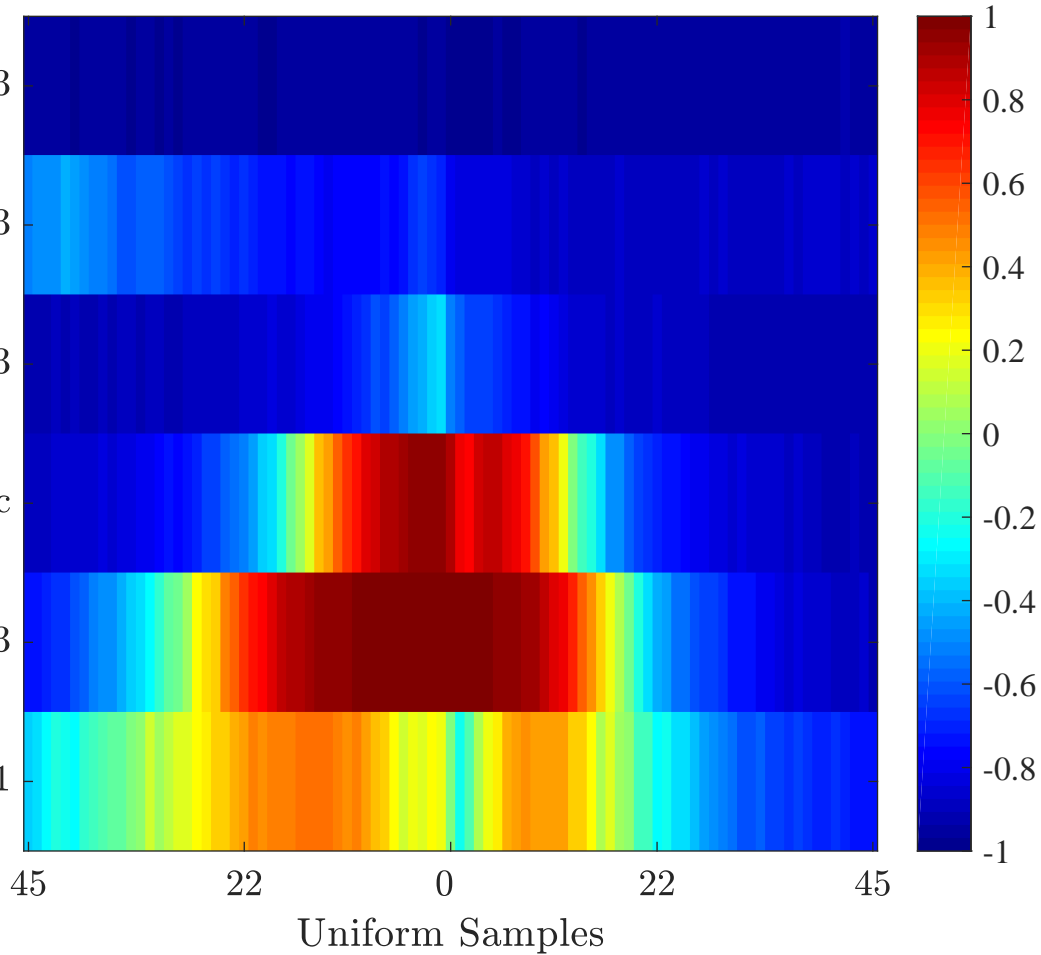

Supplement: Supplementary file 2 — HebbPlots of active promoters on the negative strand. This compressed file (.tar.gz) includes HebbPlots of promoters on the negative strand active in 57 tissues/cell types. (TAR 2952 kb) [file 12859_2018_2312_MOESM2_ESM.tar › file3/E097.pdf]

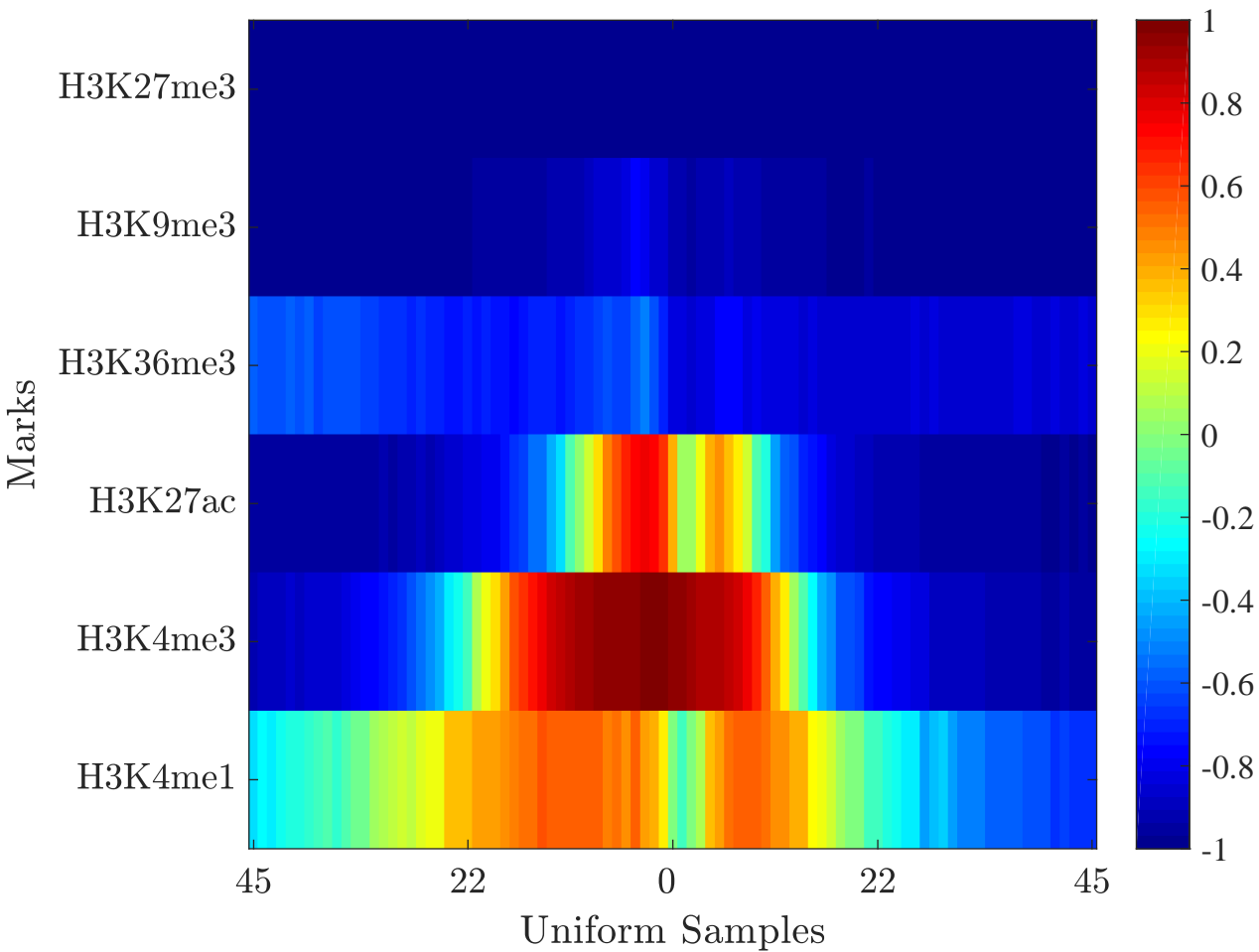

Supplement: Supplementary file 2 — HebbPlots of active promoters on the negative strand. This compressed file (.tar.gz) includes HebbPlots of promoters on the negative strand active in 57 tissues/cell types. (TAR 2952 kb) [file 12859_2018_2312_MOESM2_ESM.tar › file3/E098.pdf]

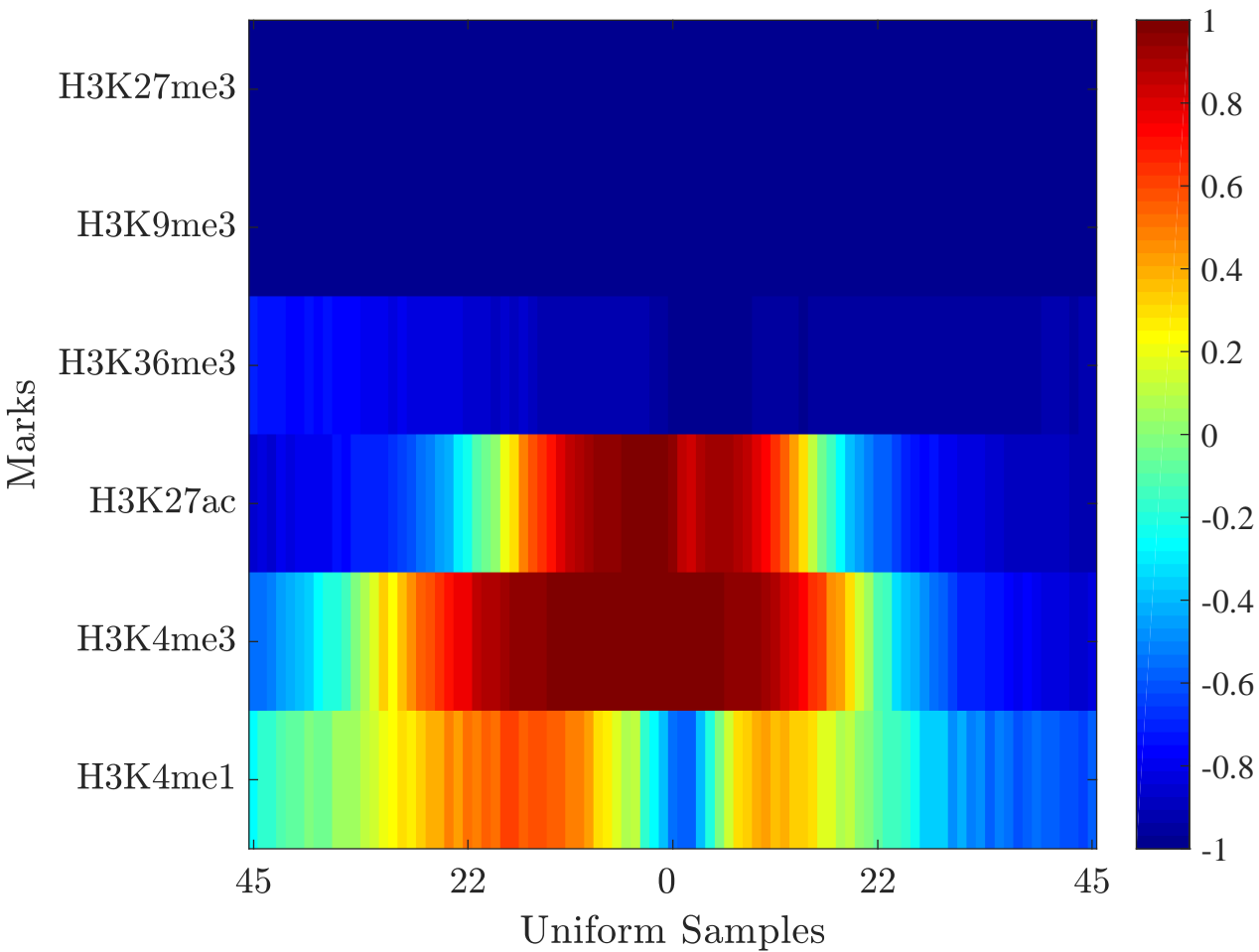

Supplement: Supplementary file 2 — HebbPlots of active promoters on the negative strand. This compressed file (.tar.gz) includes HebbPlots of promoters on the negative strand active in 57 tissues/cell types. (TAR 2952 kb) [file 12859_2018_2312_MOESM2_ESM.tar › file3/E100.pdf]

Marks

H3K27me3

H3K36me3

H3K9me3

H3K4me1

H3K27ac

H3K4me3

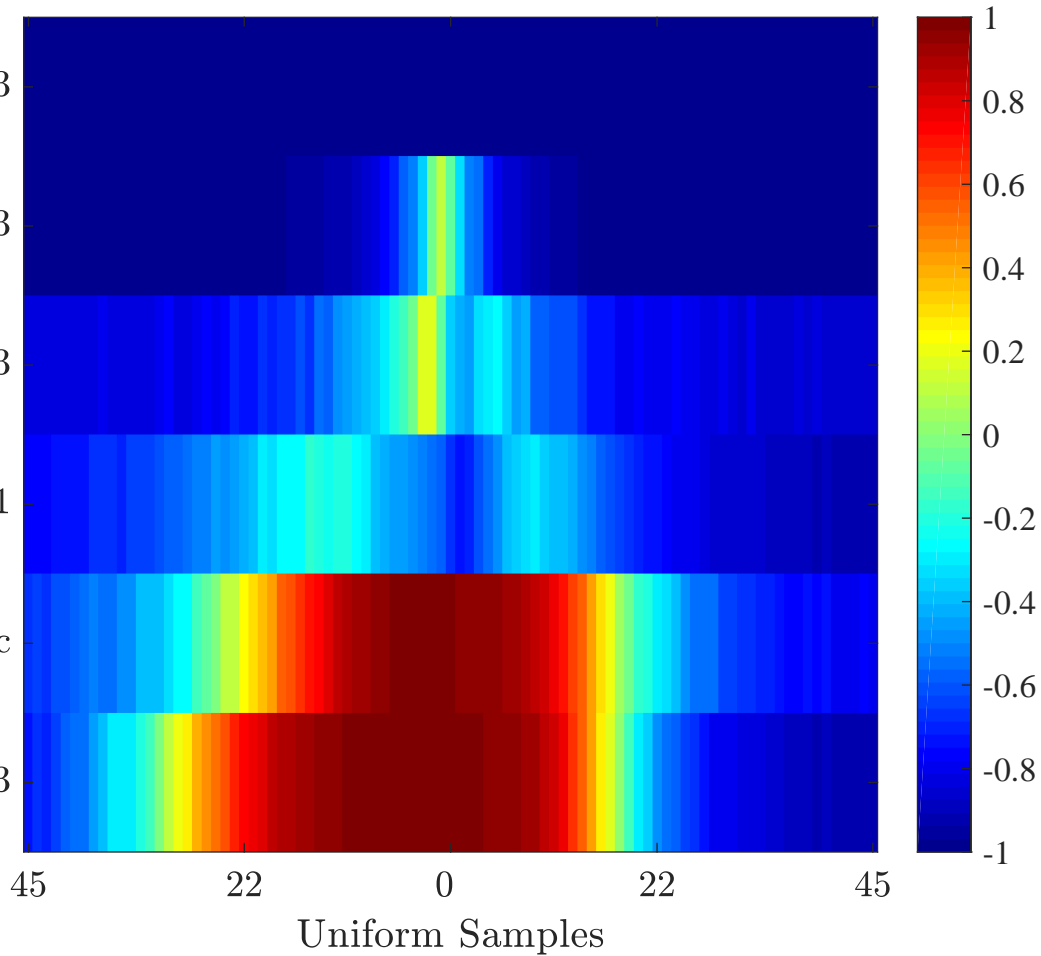

Supplement: Supplementary file 2 — HebbPlots of active promoters on the negative strand. This compressed file (.tar.gz) includes HebbPlots of promoters on the negative strand active in 57 tissues/cell types. (TAR 2952 kb) [file 12859_2018_2312_MOESM2_ESM.tar › file3/E104.pdf]

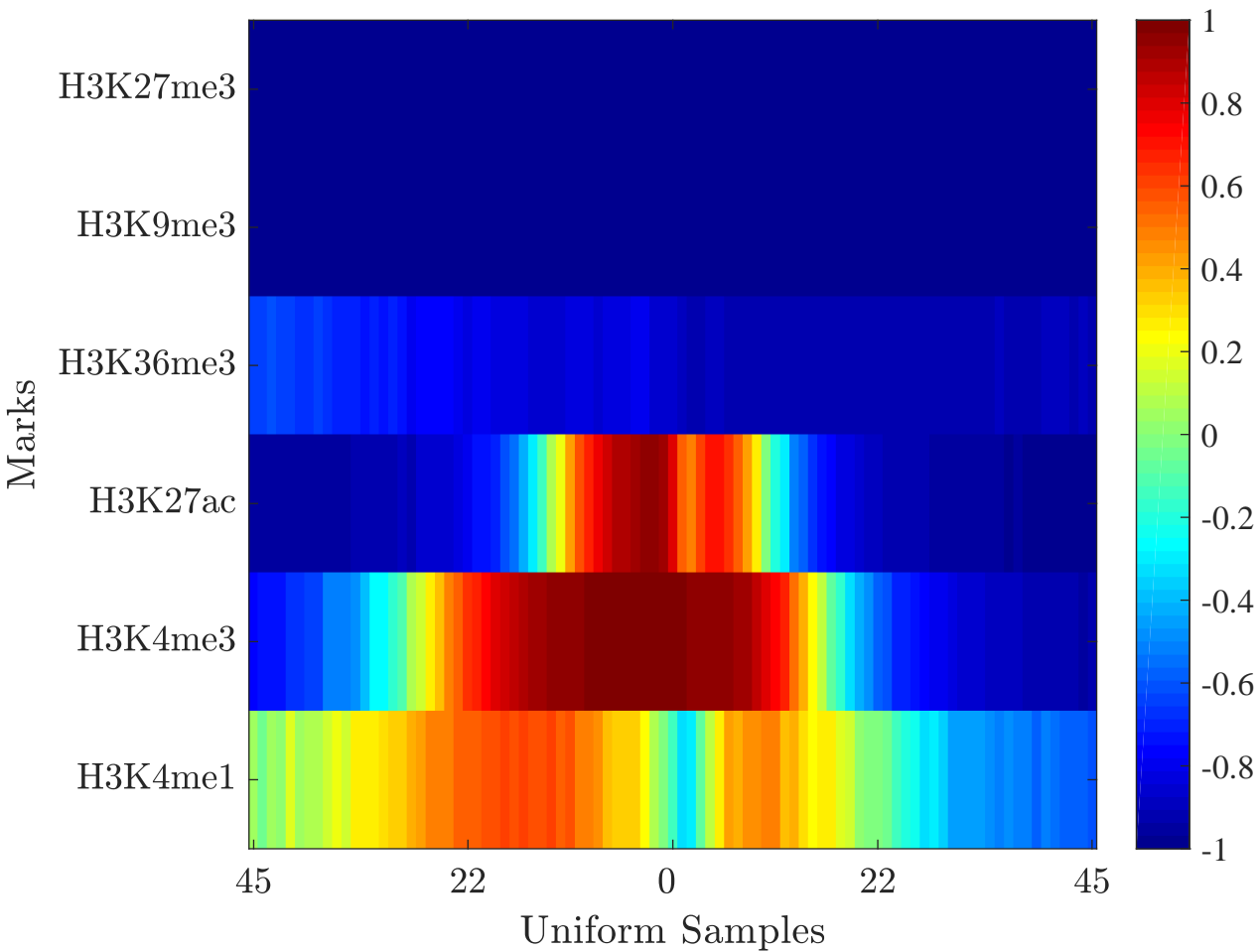

Supplement: Supplementary file 2 — HebbPlots of active promoters on the negative strand. This compressed file (.tar.gz) includes HebbPlots of promoters on the negative strand active in 57 tissues/cell types. (TAR 2952 kb) [file 12859_2018_2312_MOESM2_ESM.tar › file3/E105.pdf]
