# Supplementary figures and images for: HebbPlot: an intelligent tool for learning and visualizing chromatin mark signatures (part 2 of 4)
Source: BMC Bioinformatics. 2018 Sep 3;19:310. doi: 10.1186/s12859-018-2312-1 (PMC6122555; doi:10.1186/s12859-018-2312-1)

Marks

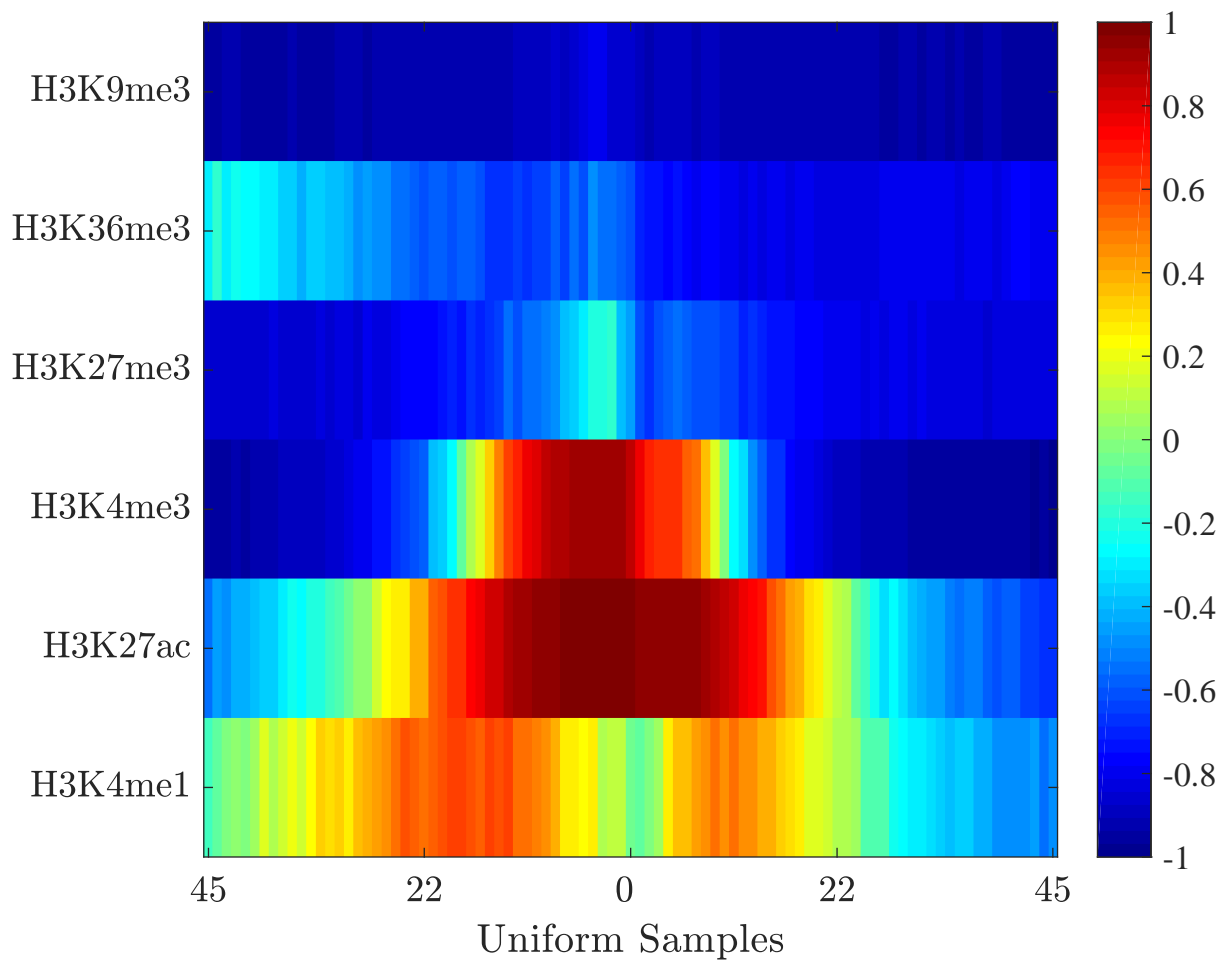

Supplement: Supplementary file 2 — HebbPlots of active promoters on the negative strand. This compressed file (.tar.gz) includes HebbPlots of promoters on the negative strand active in 57 tissues/cell types. (TAR 2952 kb) [file 12859_2018_2312_MOESM2_ESM.tar › file3/E106.pdf]

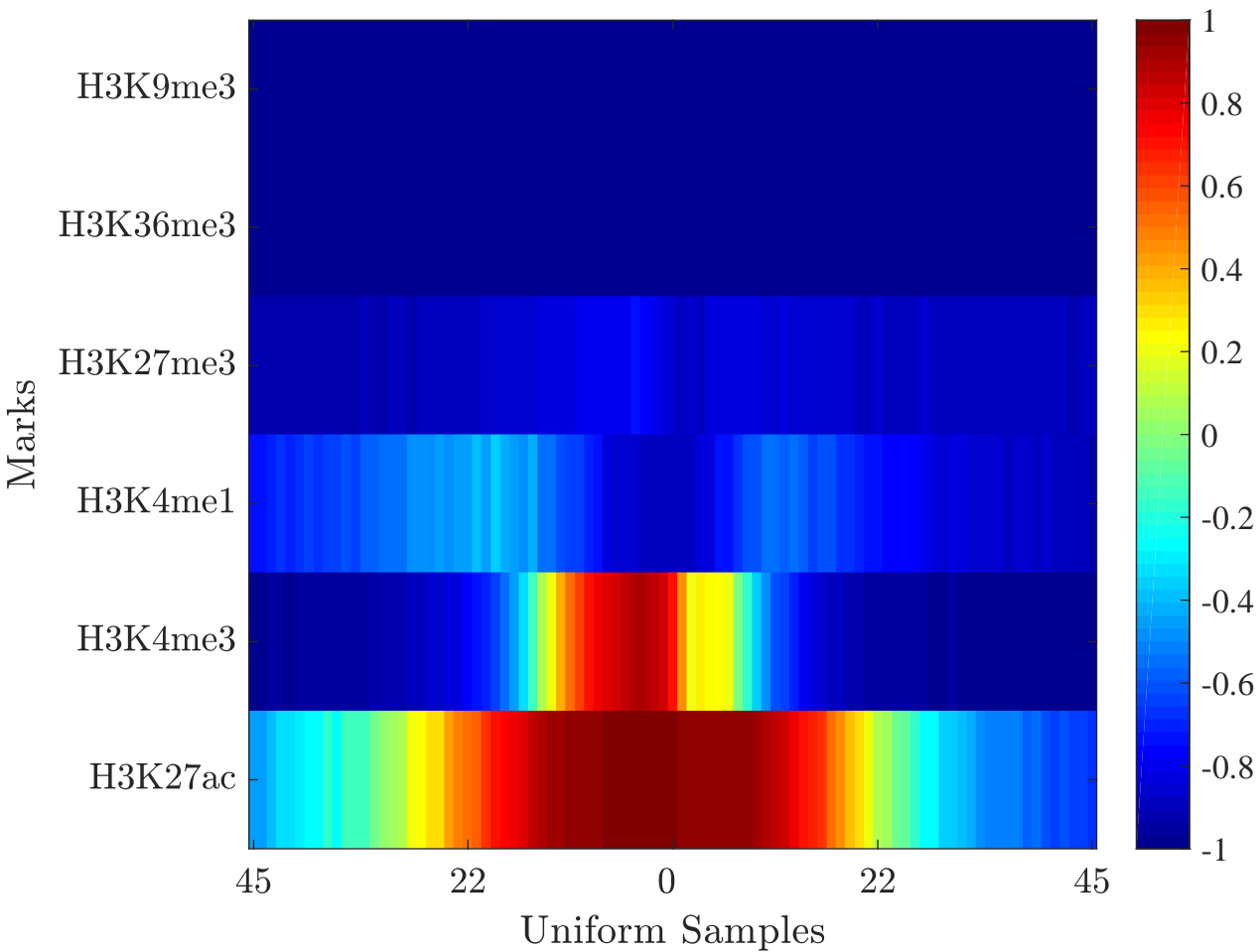

Supplement: Supplementary file 2 — HebbPlots of active promoters on the negative strand. This compressed file (.tar.gz) includes HebbPlots of promoters on the negative strand active in 57 tissues/cell types. (TAR 2952 kb) [file 12859_2018_2312_MOESM2_ESM.tar › file3/E109.pdf]

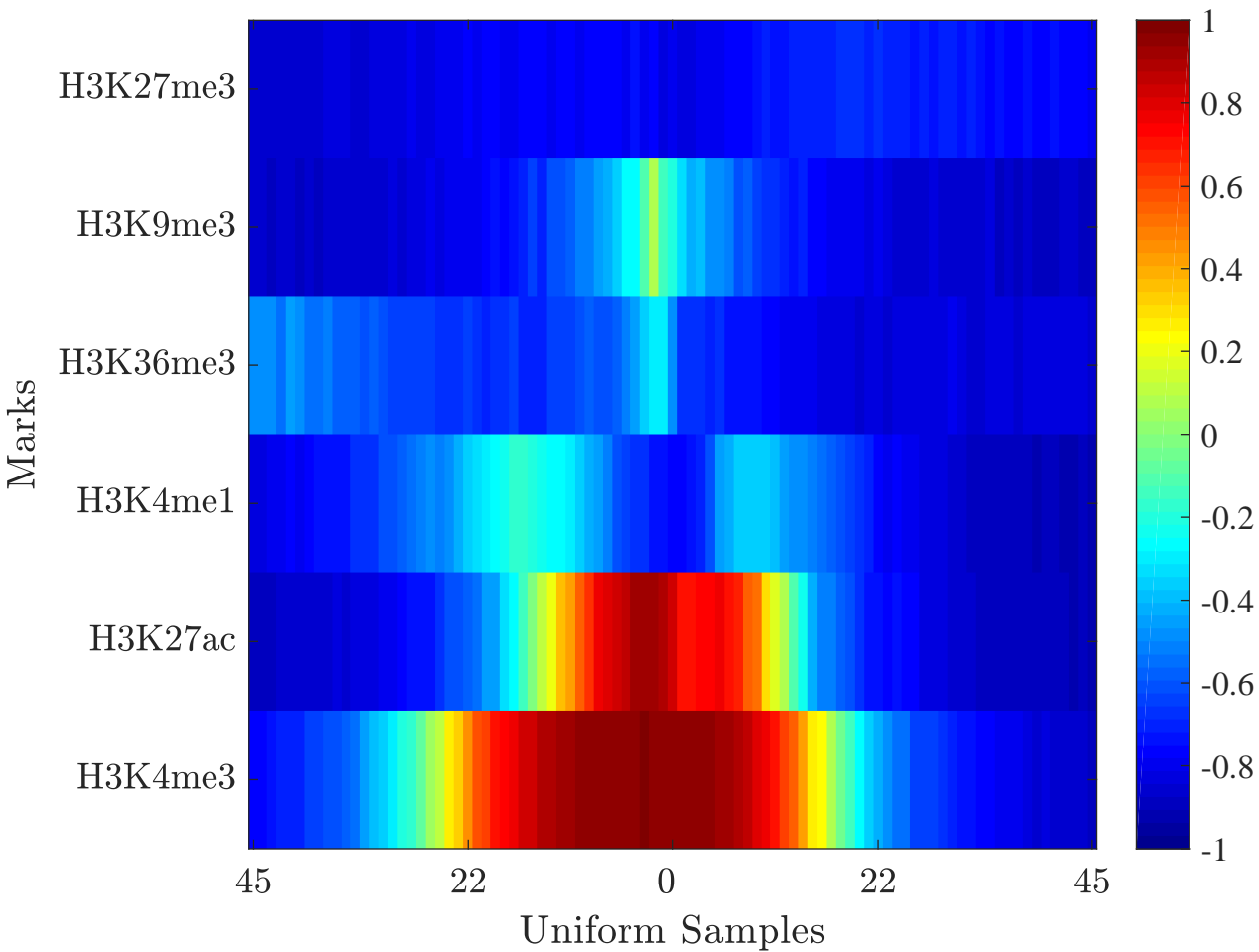

Supplement: Supplementary file 2 — HebbPlots of active promoters on the negative strand. This compressed file (.tar.gz) includes HebbPlots of promoters on the negative strand active in 57 tissues/cell types. (TAR 2952 kb) [file 12859_2018_2312_MOESM2_ESM.tar › file3/E112.pdf]

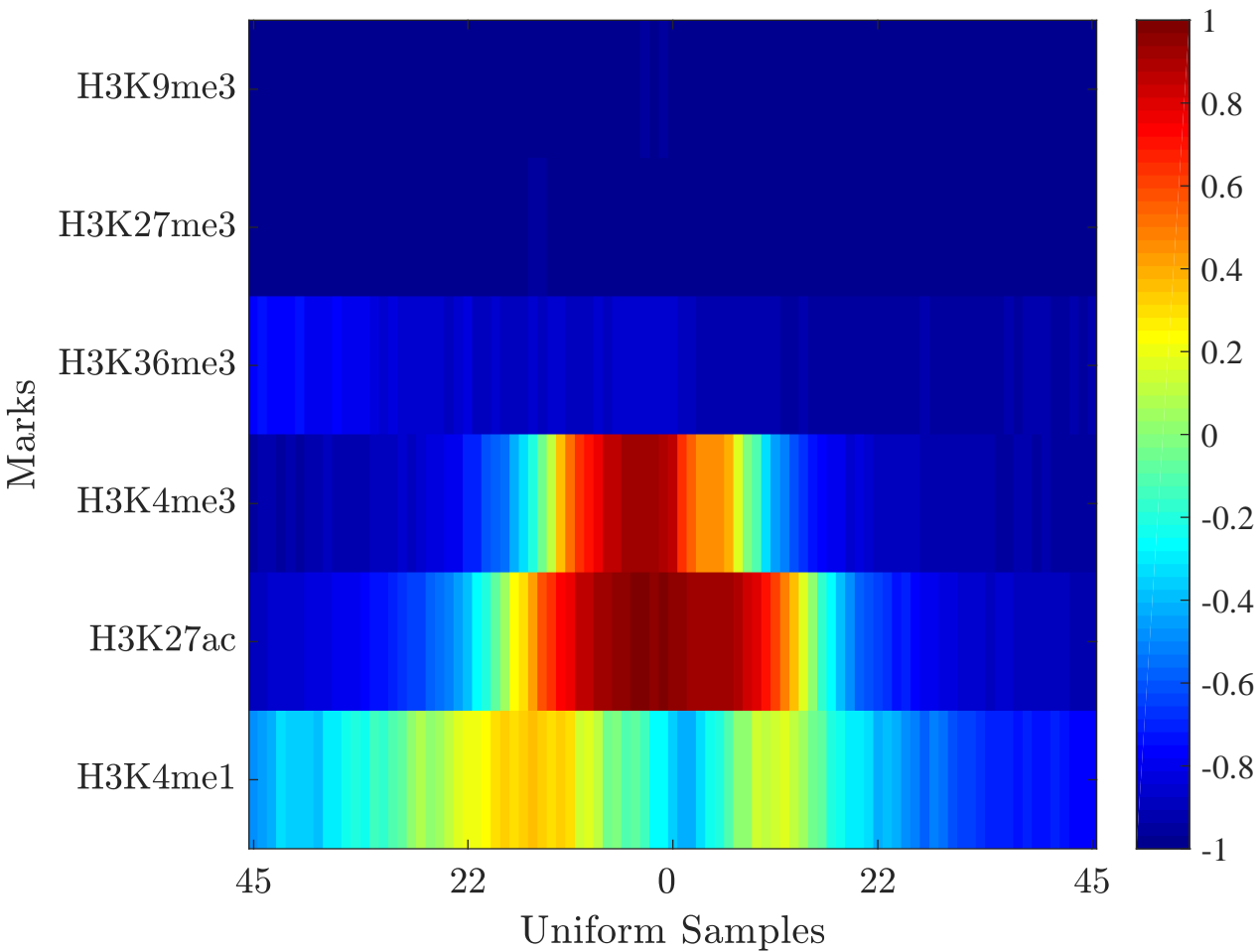

Supplement: Supplementary file 2 — HebbPlots of active promoters on the negative strand. This compressed file (.tar.gz) includes HebbPlots of promoters on the negative strand active in 57 tissues/cell types. (TAR 2952 kb) [file 12859_2018_2312_MOESM2_ESM.tar › file3/E113.pdf]

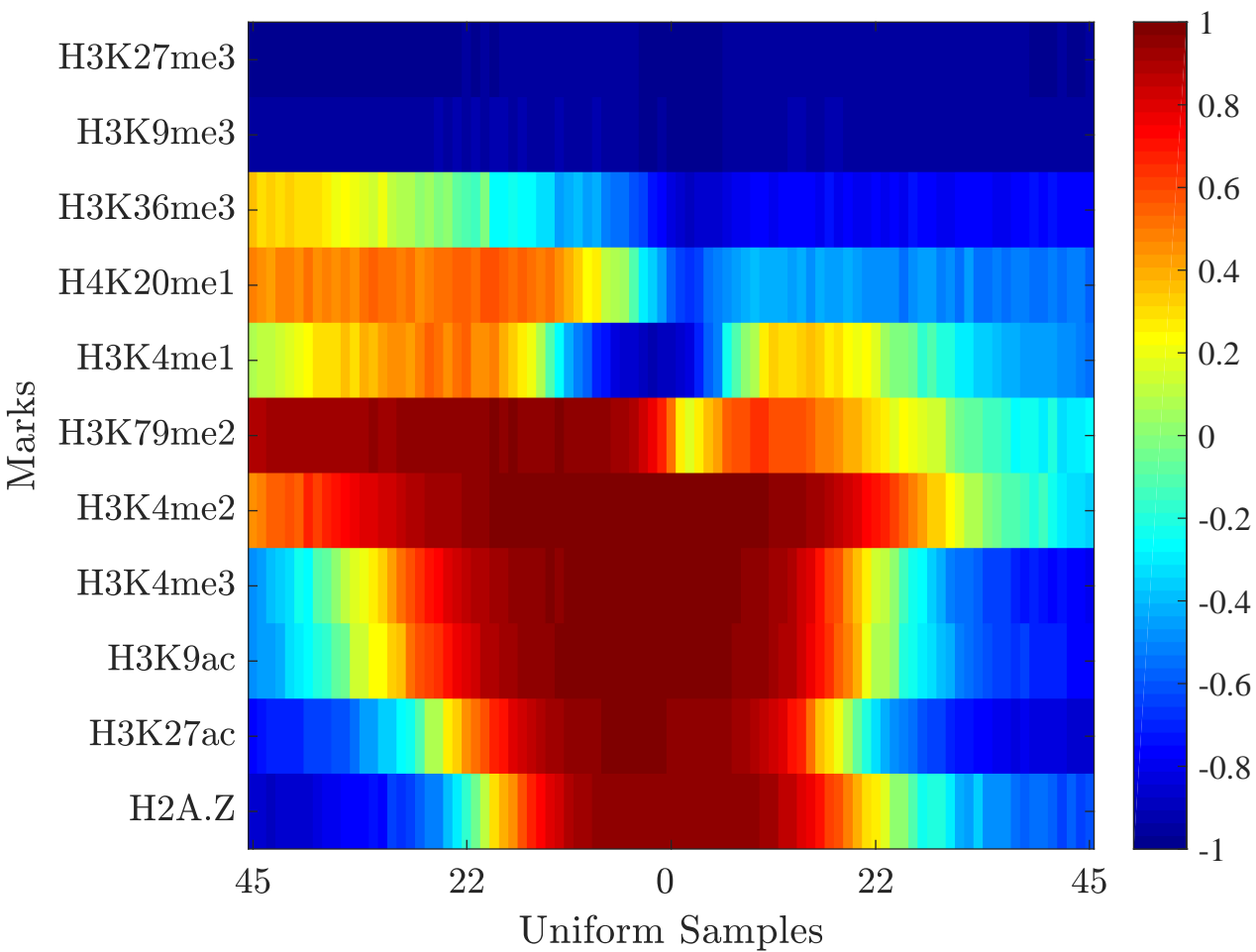

Supplement: Supplementary file 2 — HebbPlots of active promoters on the negative strand. This compressed file (.tar.gz) includes HebbPlots of promoters on the negative strand active in 57 tissues/cell types. (TAR 2952 kb) [file 12859_2018_2312_MOESM2_ESM.tar › file3/E114.pdf]

Marks

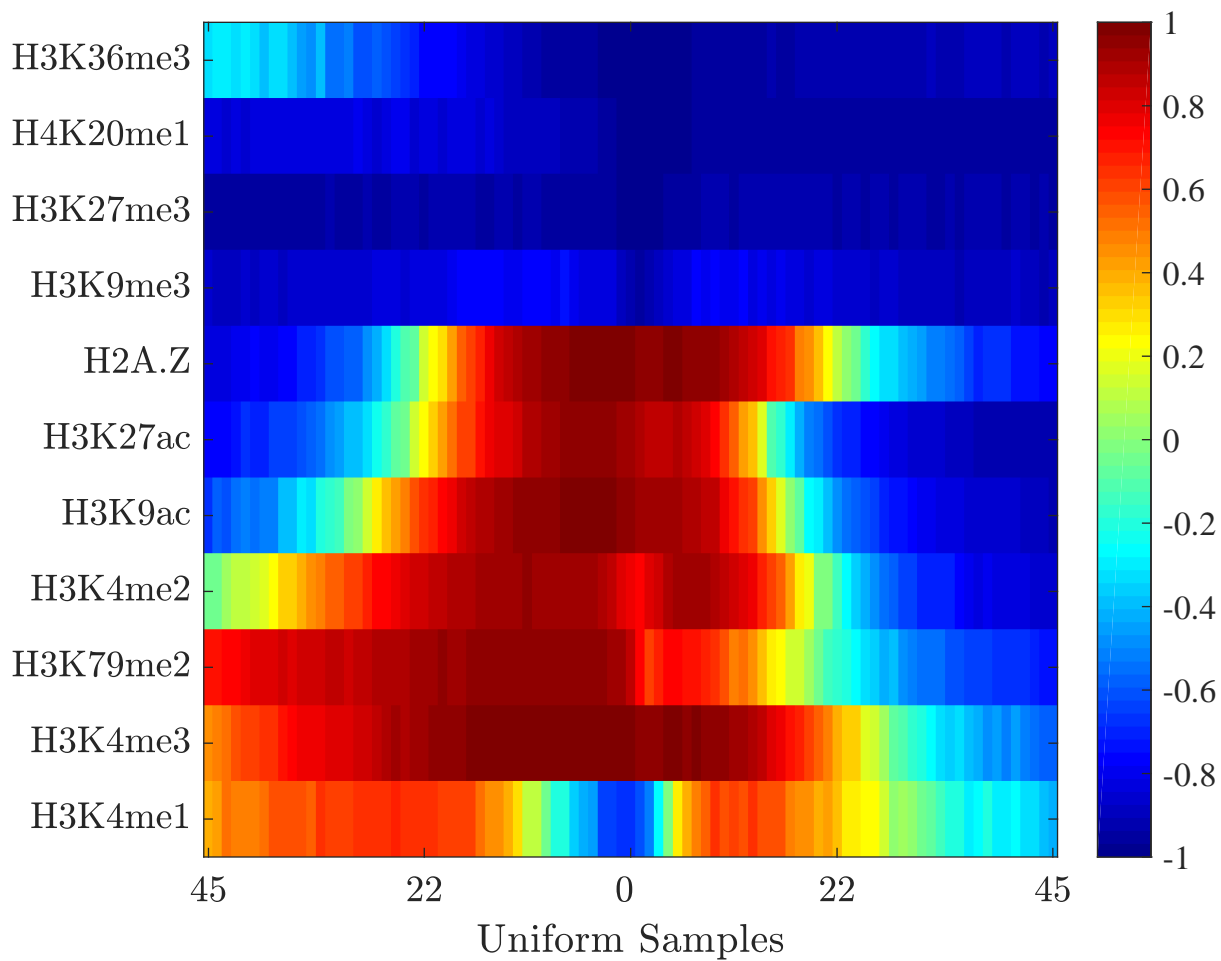

Supplement: Supplementary file 2 — HebbPlots of active promoters on the negative strand. This compressed file (.tar.gz) includes HebbPlots of promoters on the negative strand active in 57 tissues/cell types. (TAR 2952 kb) [file 12859_2018_2312_MOESM2_ESM.tar › file3/E116.pdf]

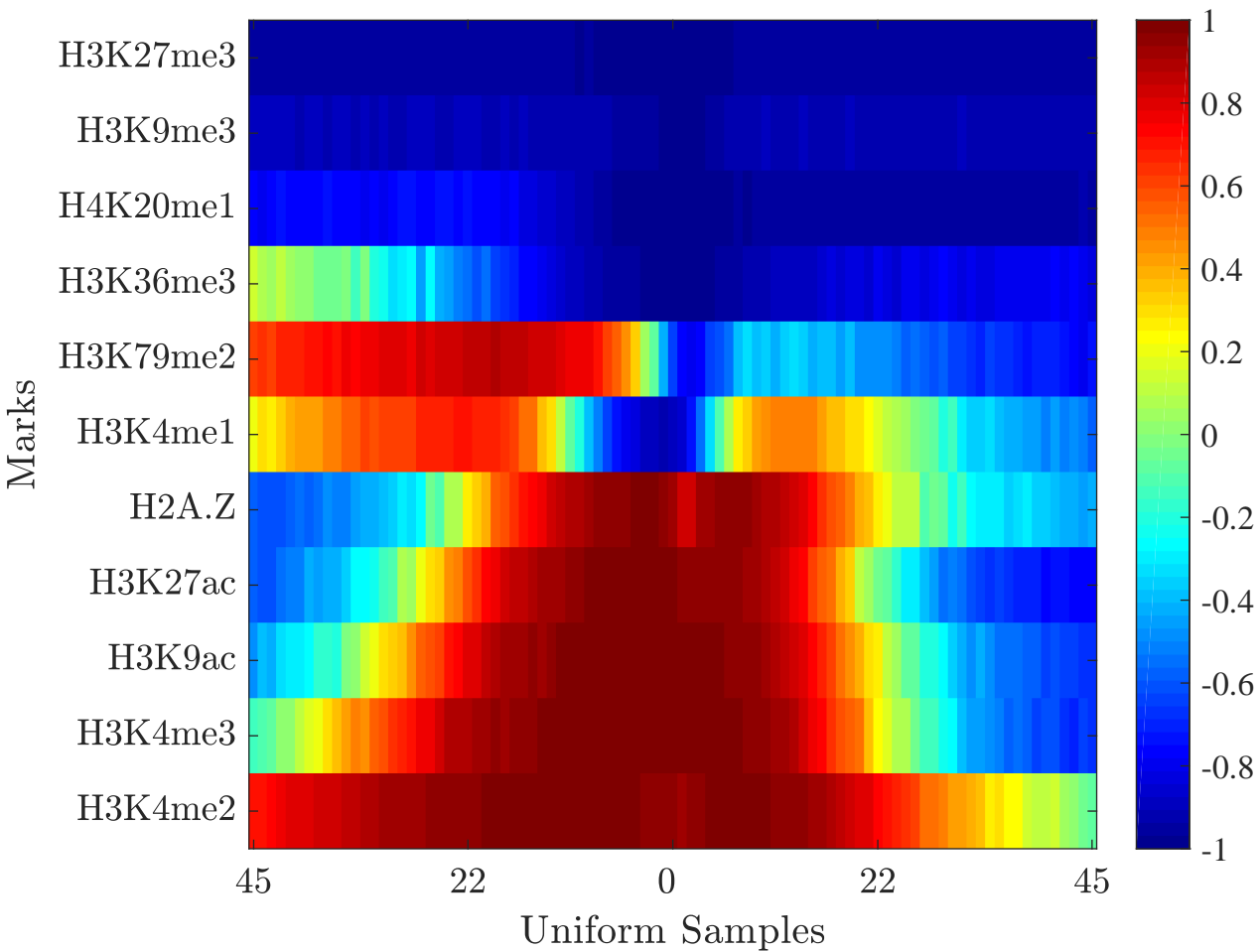

Supplement: Supplementary file 2 — HebbPlots of active promoters on the negative strand. This compressed file (.tar.gz) includes HebbPlots of promoters on the negative strand active in 57 tissues/cell types. (TAR 2952 kb) [file 12859_2018_2312_MOESM2_ESM.tar › file3/E117.pdf]

Marks

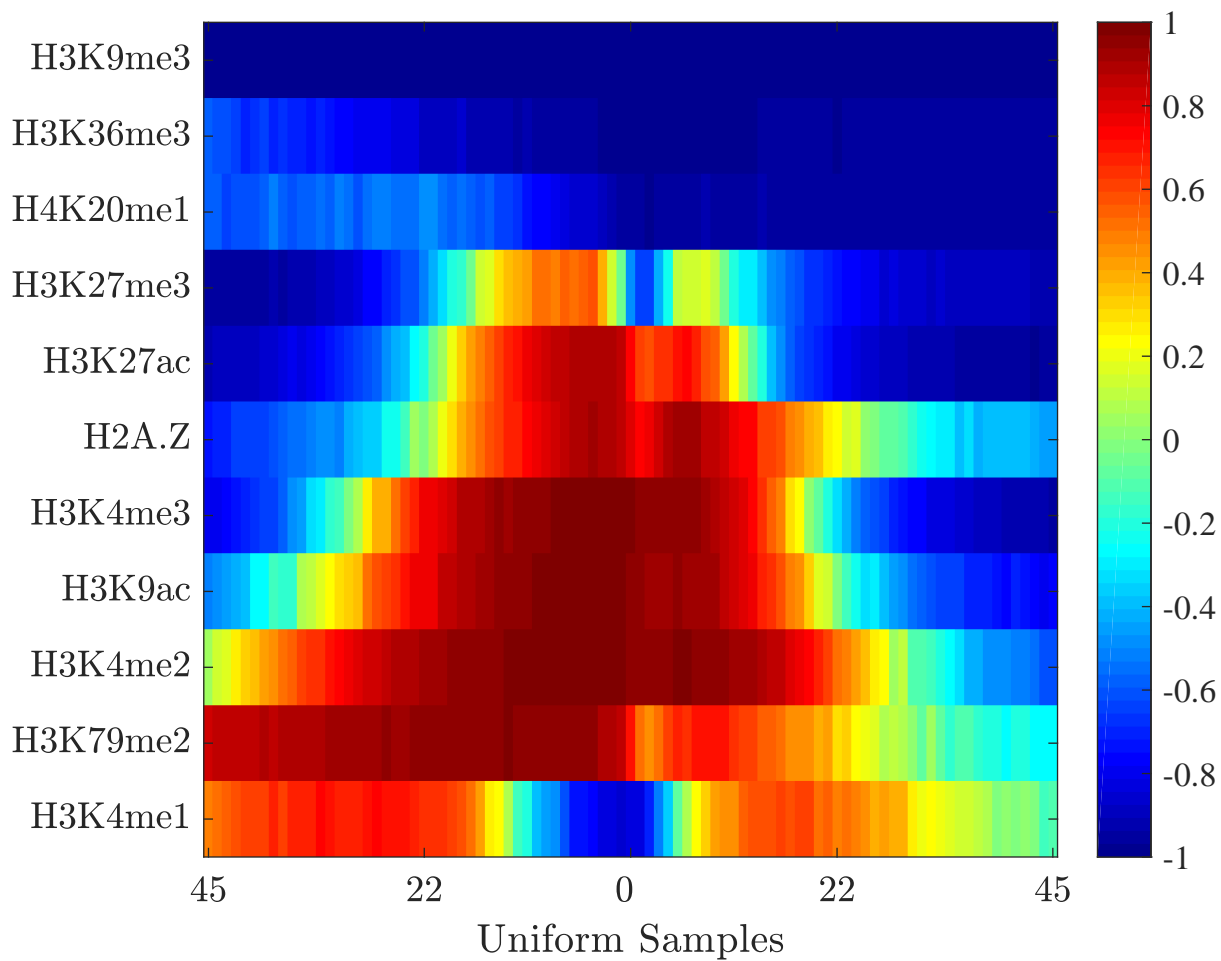

Supplement: Supplementary file 2 — HebbPlots of active promoters on the negative strand. This compressed file (.tar.gz) includes HebbPlots of promoters on the negative strand active in 57 tissues/cell types. (TAR 2952 kb) [file 12859_2018_2312_MOESM2_ESM.tar › file3/E118.pdf]

Marks

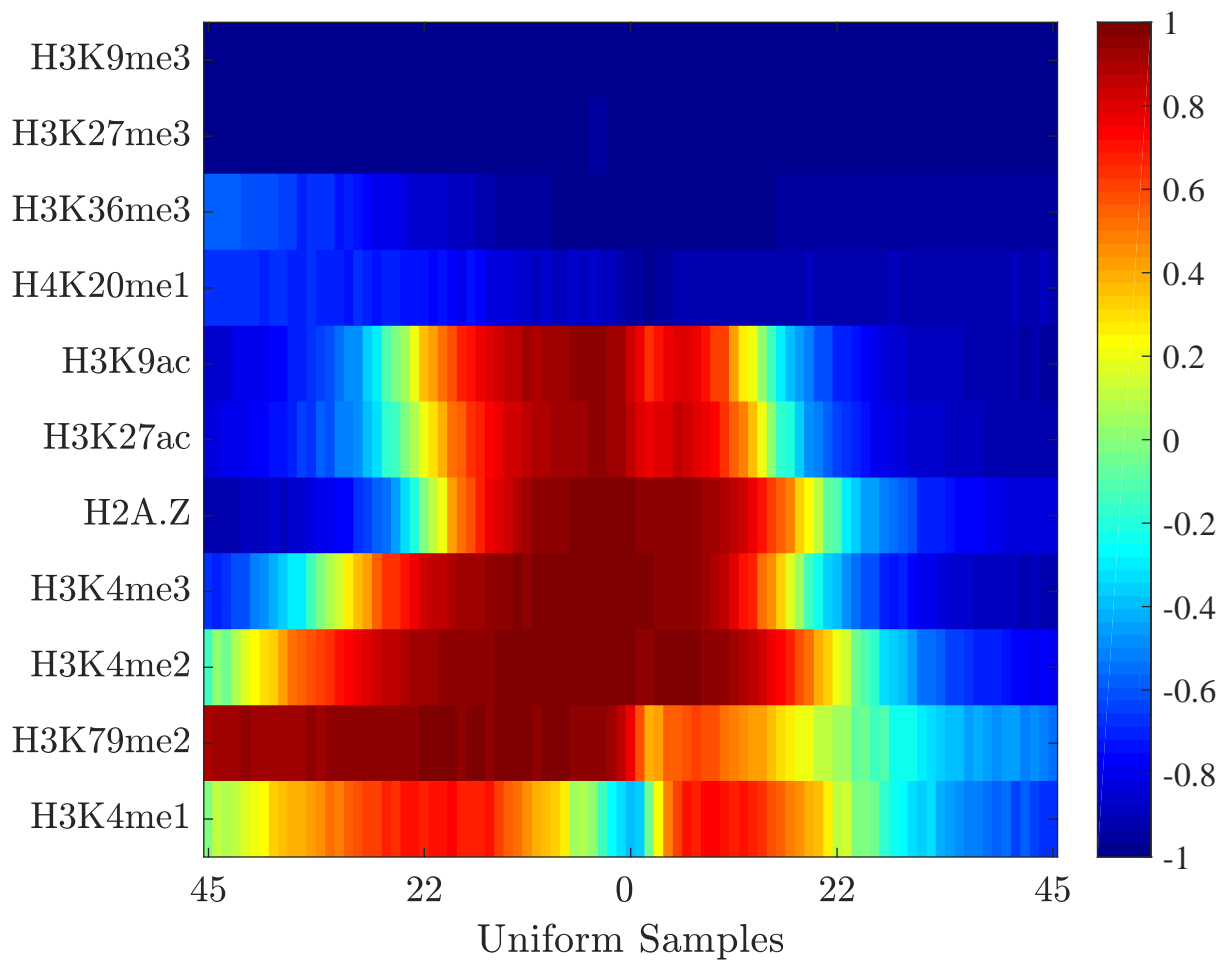

Supplement: Supplementary file 2 — HebbPlots of active promoters on the negative strand. This compressed file (.tar.gz) includes HebbPlots of promoters on the negative strand active in 57 tissues/cell types. (TAR 2952 kb) [file 12859_2018_2312_MOESM2_ESM.tar › file3/E119.pdf]

Marks

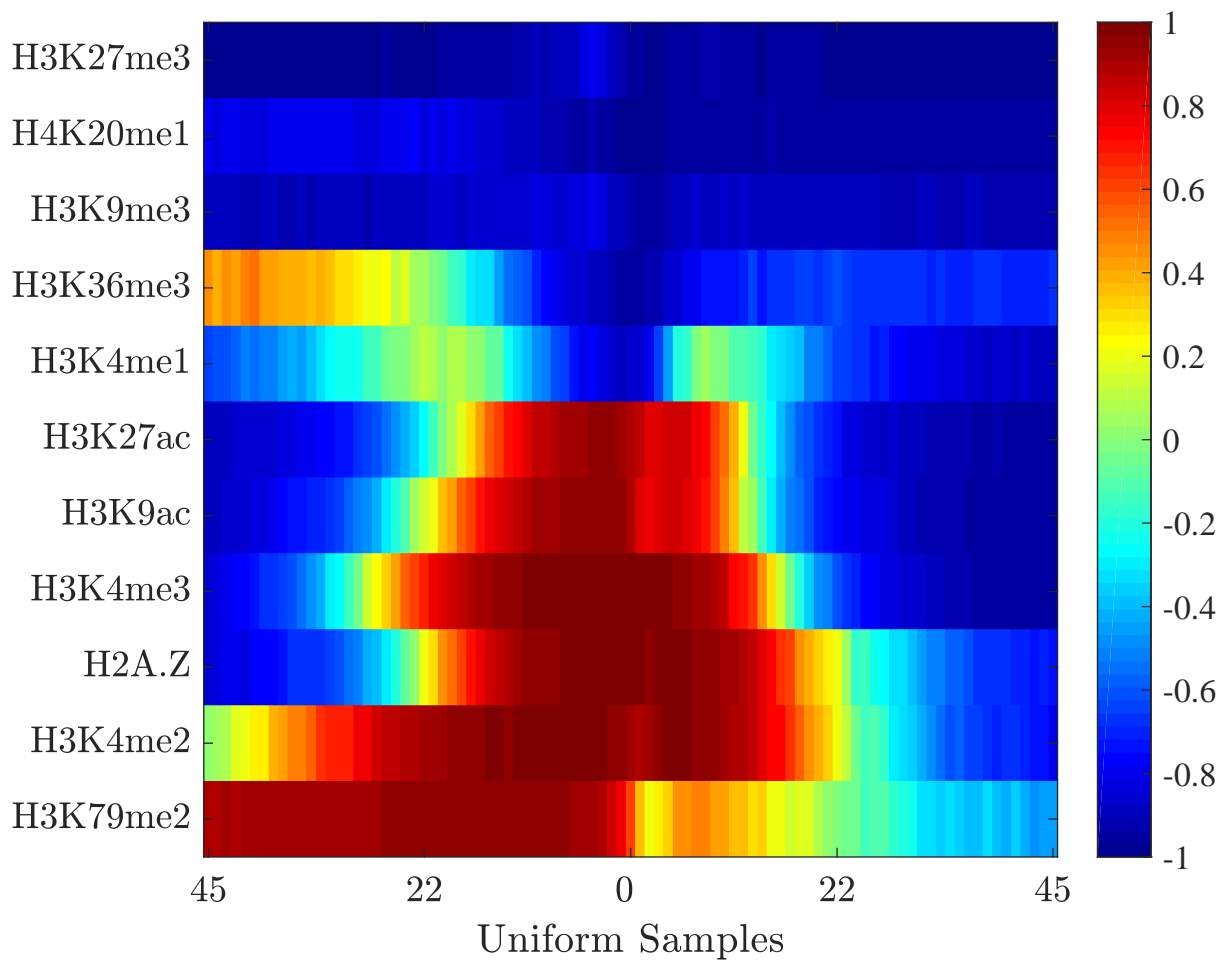

Supplement: Supplementary file 2 — HebbPlots of active promoters on the negative strand. This compressed file (.tar.gz) includes HebbPlots of promoters on the negative strand active in 57 tissues/cell types. (TAR 2952 kb) [file 12859_2018_2312_MOESM2_ESM.tar › file3/E120.pdf]

Marks

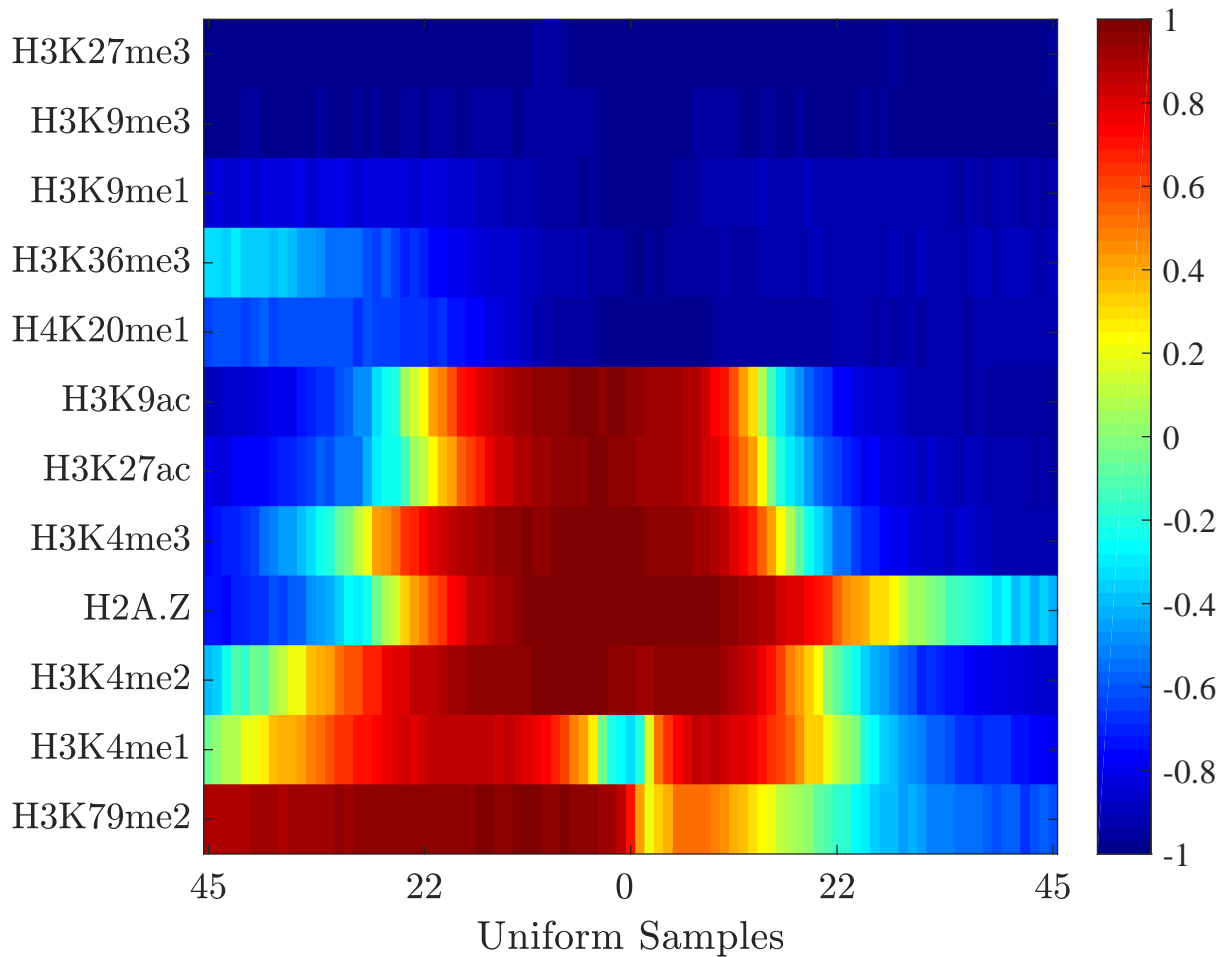

Supplement: Supplementary file 2 — HebbPlots of active promoters on the negative strand. This compressed file (.tar.gz) includes HebbPlots of promoters on the negative strand active in 57 tissues/cell types. (TAR 2952 kb) [file 12859_2018_2312_MOESM2_ESM.tar › file3/E122.pdf]

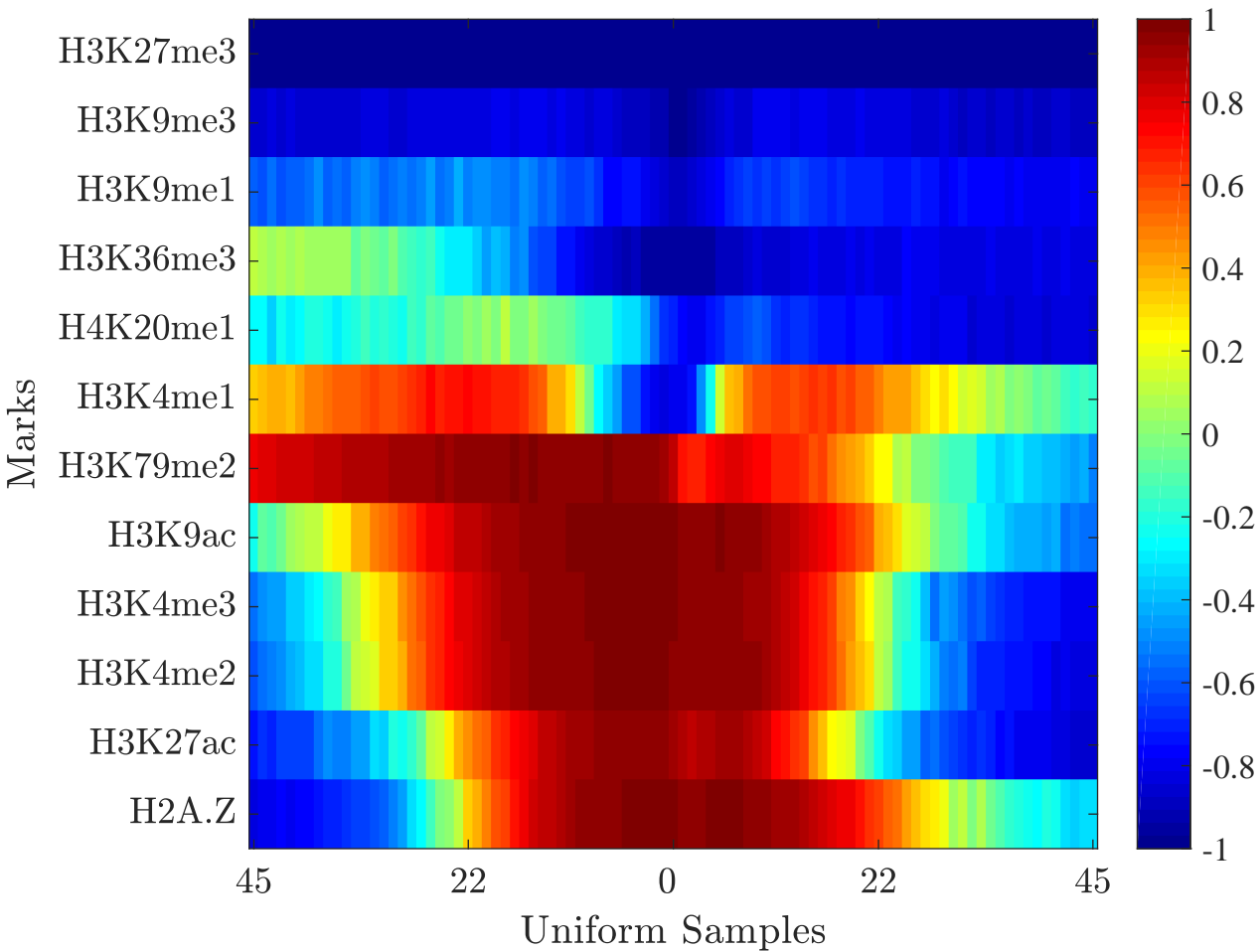

Supplement: Supplementary file 2 — HebbPlots of active promoters on the negative strand. This compressed file (.tar.gz) includes HebbPlots of promoters on the negative strand active in 57 tissues/cell types. (TAR 2952 kb) [file 12859_2018_2312_MOESM2_ESM.tar › file3/E123.pdf]

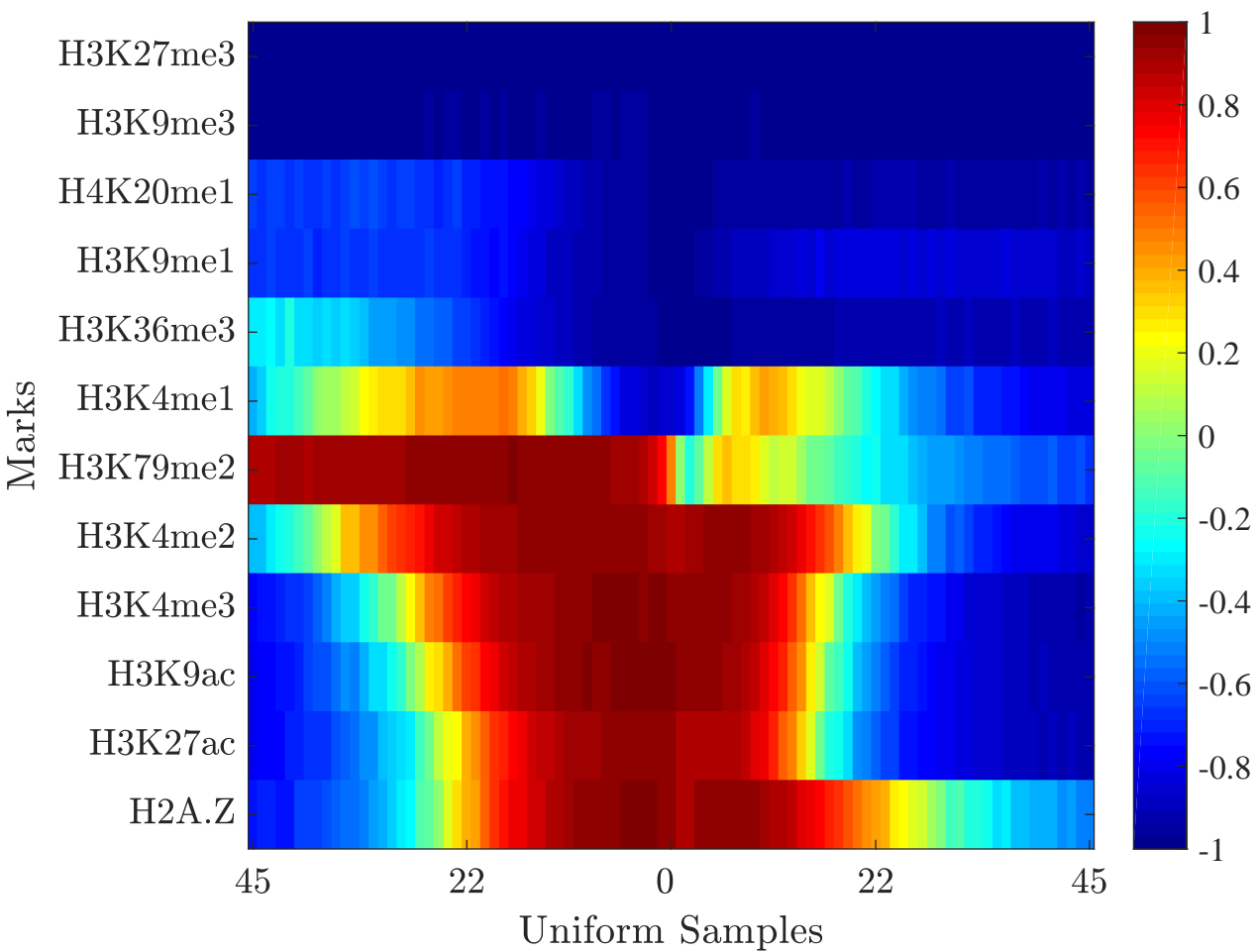

Supplement: Supplementary file 2 — HebbPlots of active promoters on the negative strand. This compressed file (.tar.gz) includes HebbPlots of promoters on the negative strand active in 57 tissues/cell types. (TAR 2952 kb) [file 12859_2018_2312_MOESM2_ESM.tar › file3/E127.pdf]

Marks

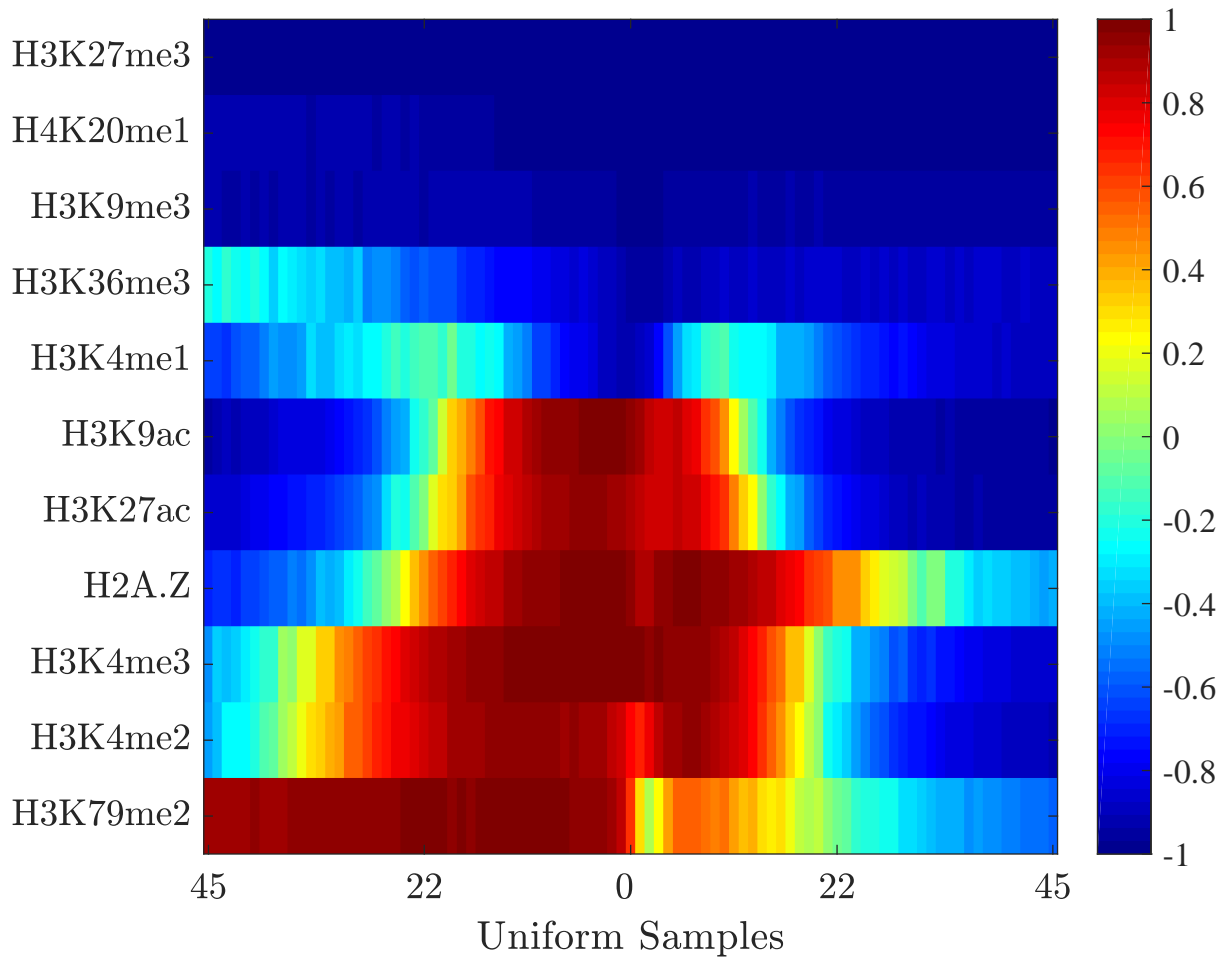

Supplement: Supplementary file 2 — HebbPlots of active promoters on the negative strand. This compressed file (.tar.gz) includes HebbPlots of promoters on the negative strand active in 57 tissues/cell types. (TAR 2952 kb) [file 12859_2018_2312_MOESM2_ESM.tar › file3/E128.pdf]

Marks

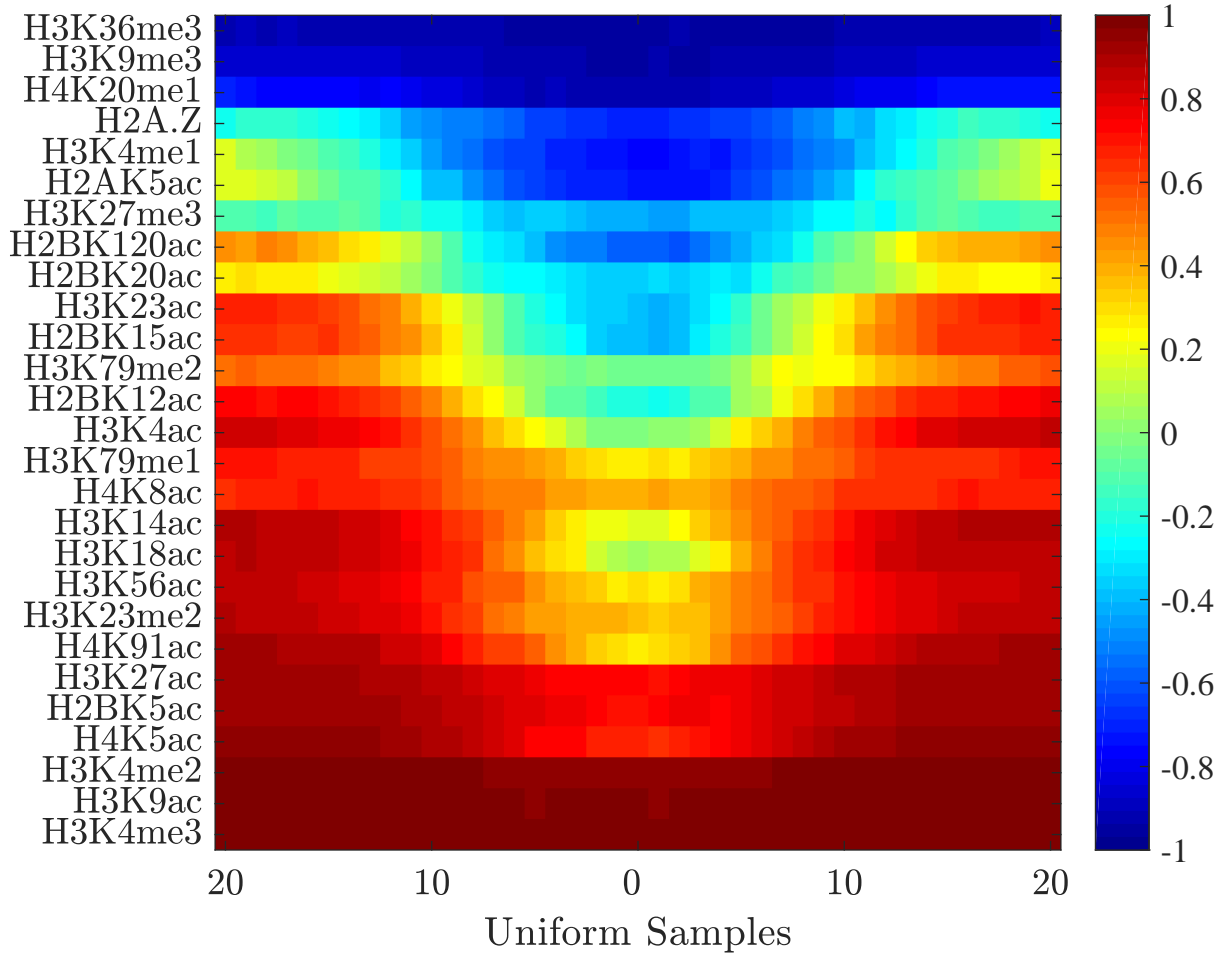

Supplement: Supplementary file 3 — HebbPlots of high-CpG promoters. This compressed file (.tar.gz) includes HebbPlots of high-CpG promoters active in 57 tissues/cell types. (TAR 2654 kb) [file 12859_2018_2312_MOESM3_ESM.tar › file4/E003.pdf]

Marks

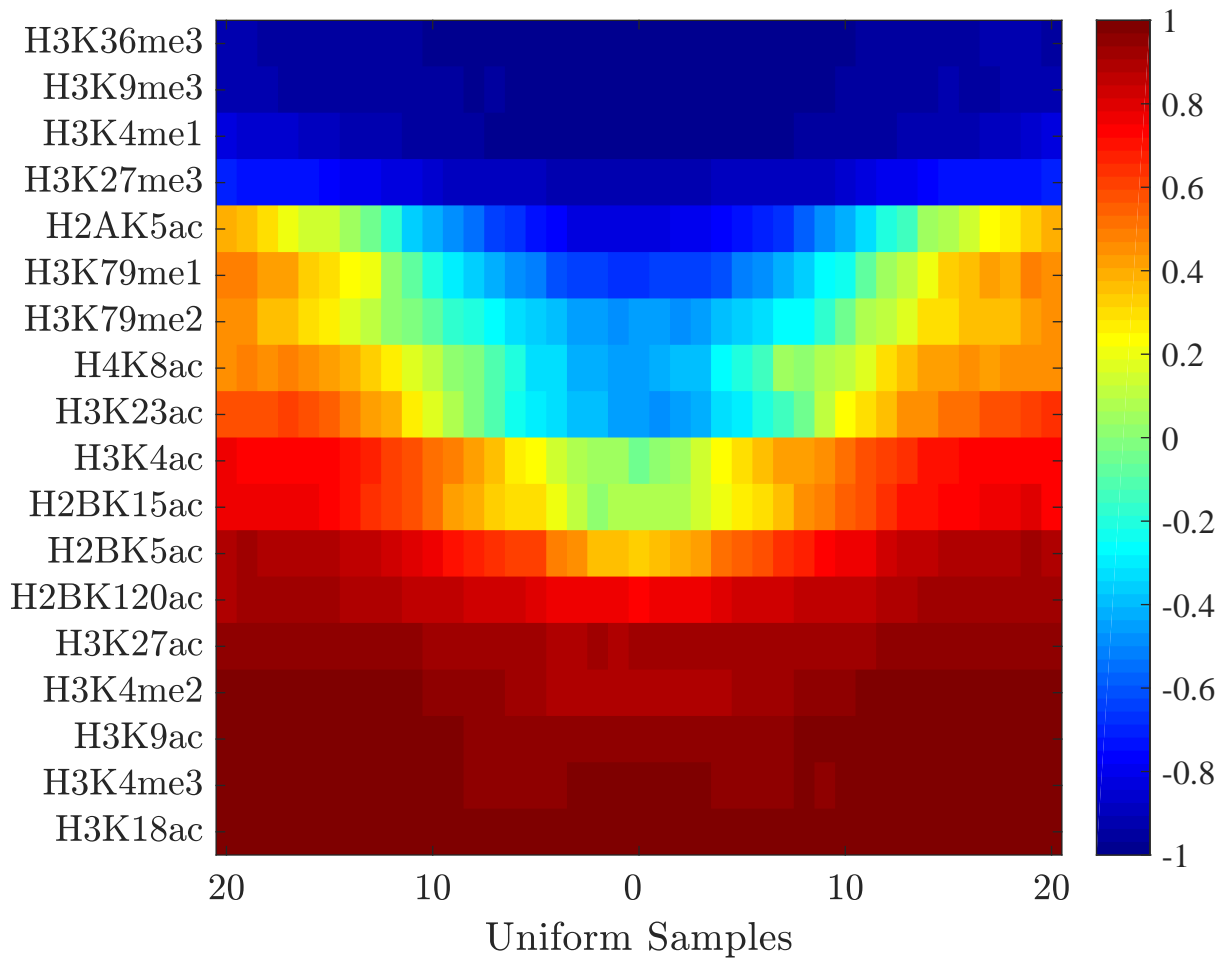

Supplement: Supplementary file 3 — HebbPlots of high-CpG promoters. This compressed file (.tar.gz) includes HebbPlots of high-CpG promoters active in 57 tissues/cell types. (TAR 2654 kb) [file 12859_2018_2312_MOESM3_ESM.tar › file4/E004.pdf]

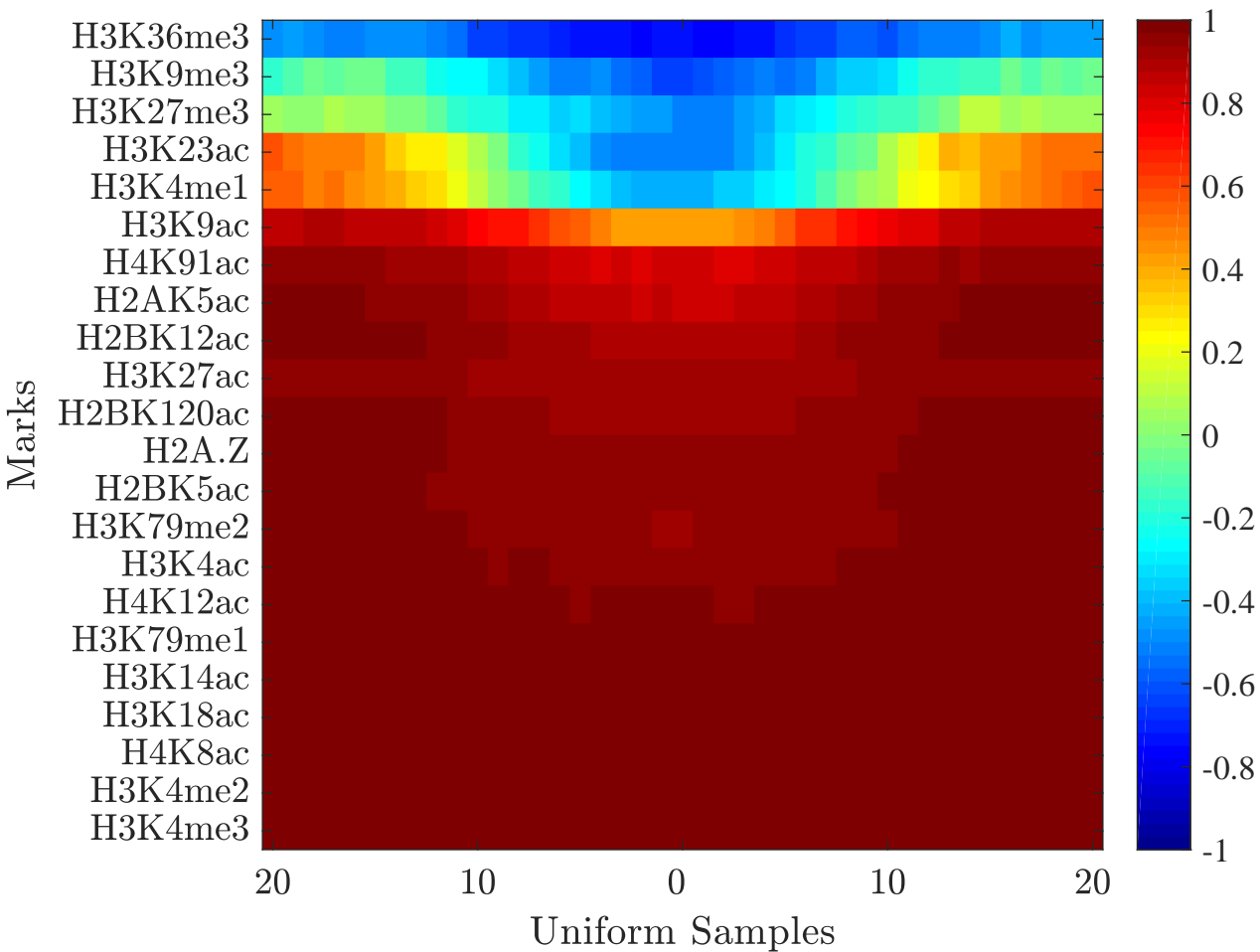

Supplement: Supplementary file 3 — HebbPlots of high-CpG promoters. This compressed file (.tar.gz) includes HebbPlots of high-CpG promoters active in 57 tissues/cell types. (TAR 2654 kb) [file 12859_2018_2312_MOESM3_ESM.tar › file4/E005.pdf]

Marks

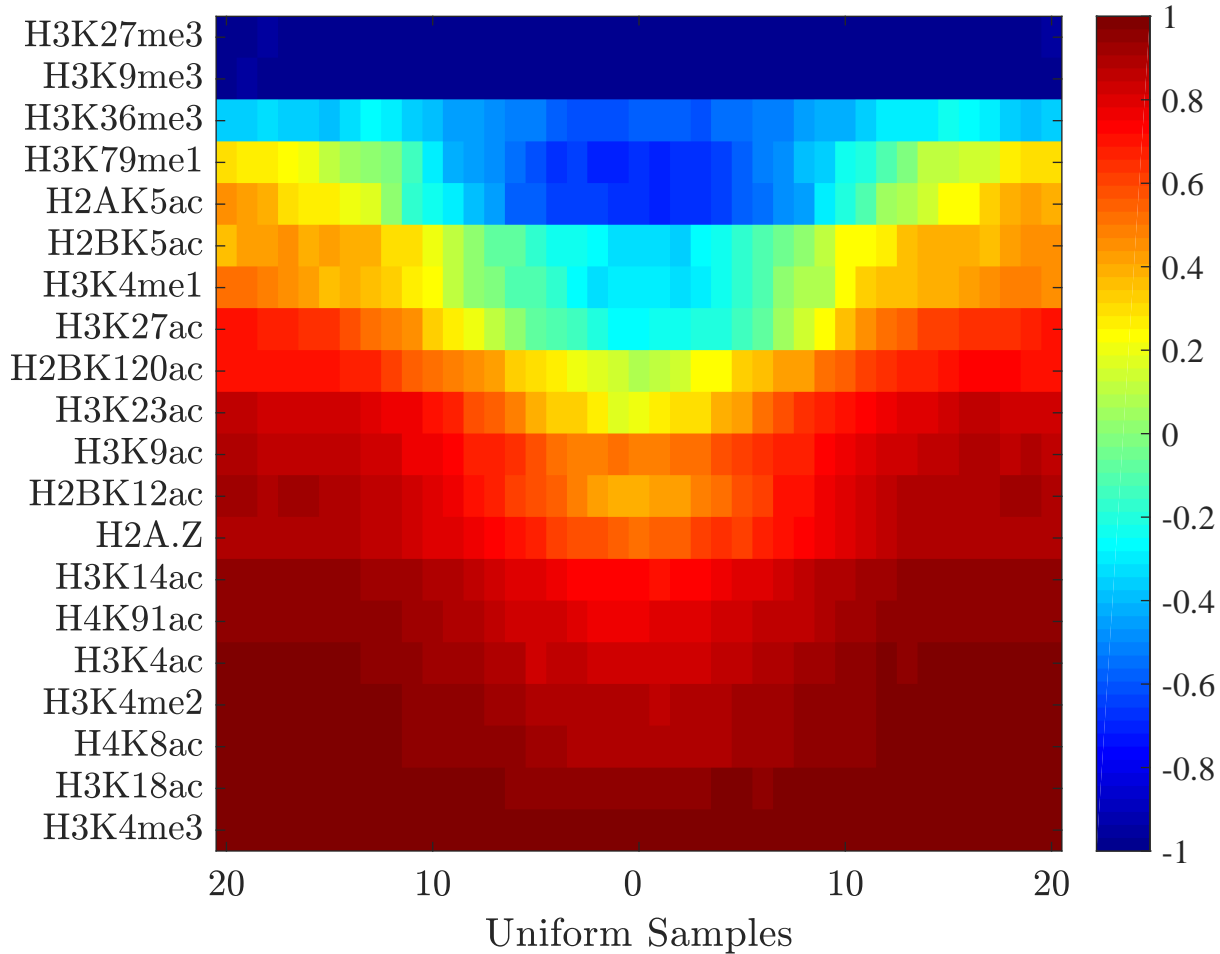

Supplement: Supplementary file 3 — HebbPlots of high-CpG promoters. This compressed file (.tar.gz) includes HebbPlots of high-CpG promoters active in 57 tissues/cell types. (TAR 2654 kb) [file 12859_2018_2312_MOESM3_ESM.tar › file4/E006.pdf]

Marks

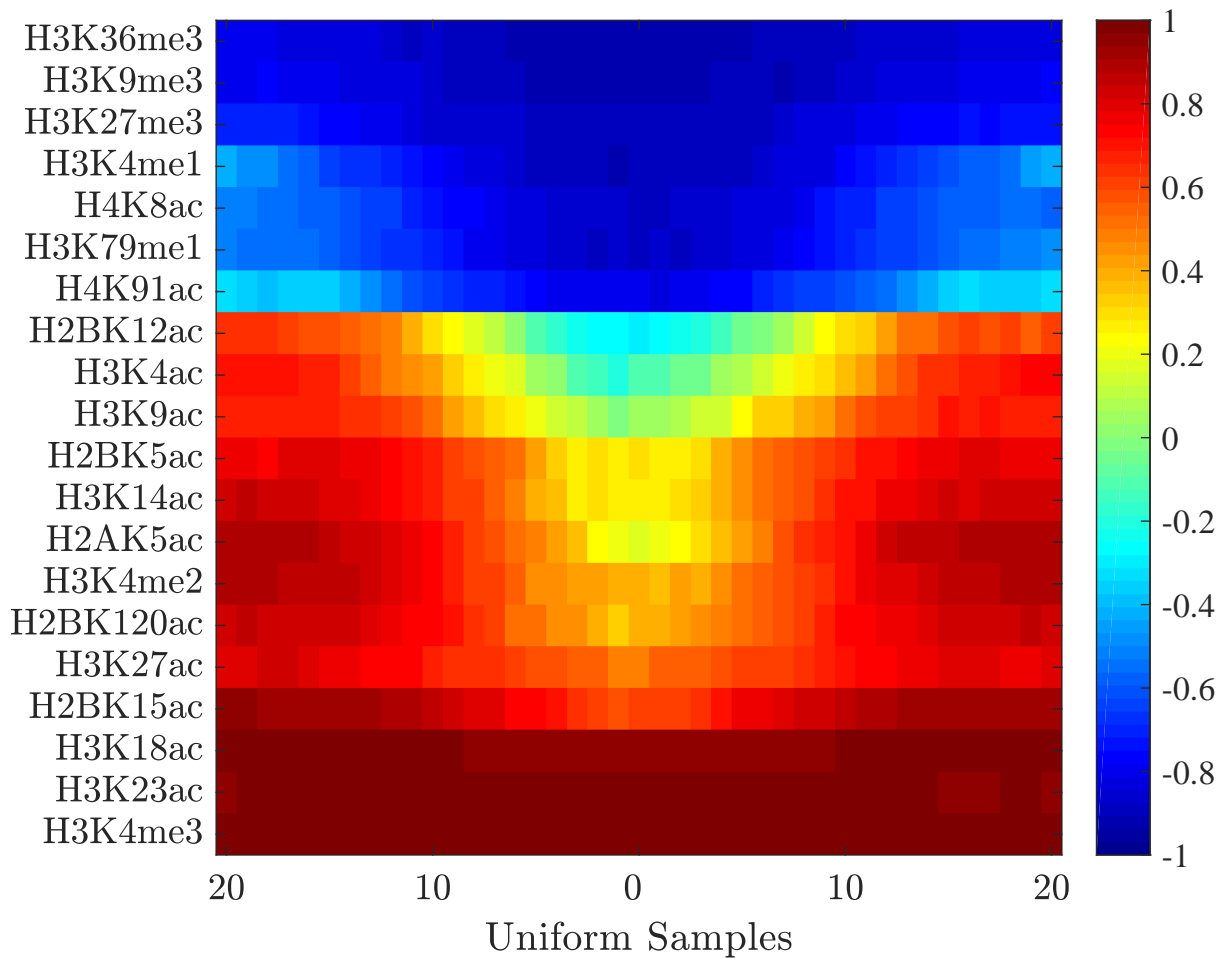

Supplement: Supplementary file 3 — HebbPlots of high-CpG promoters. This compressed file (.tar.gz) includes HebbPlots of high-CpG promoters active in 57 tissues/cell types. (TAR 2654 kb) [file 12859_2018_2312_MOESM3_ESM.tar › file4/E007.pdf]

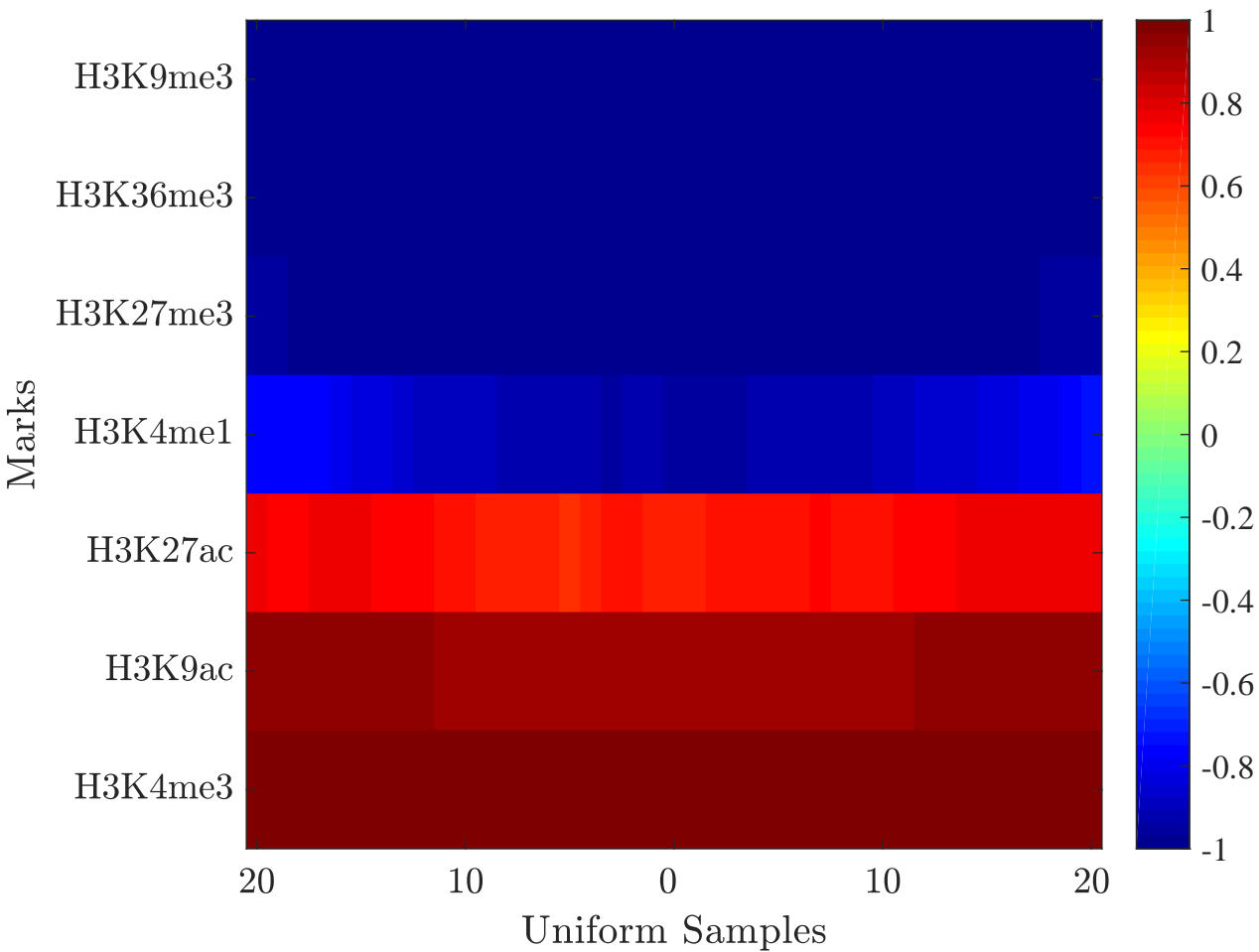

Supplement: Supplementary file 3 — HebbPlots of high-CpG promoters. This compressed file (.tar.gz) includes HebbPlots of high-CpG promoters active in 57 tissues/cell types. (TAR 2654 kb) [file 12859_2018_2312_MOESM3_ESM.tar › file4/E011.pdf]

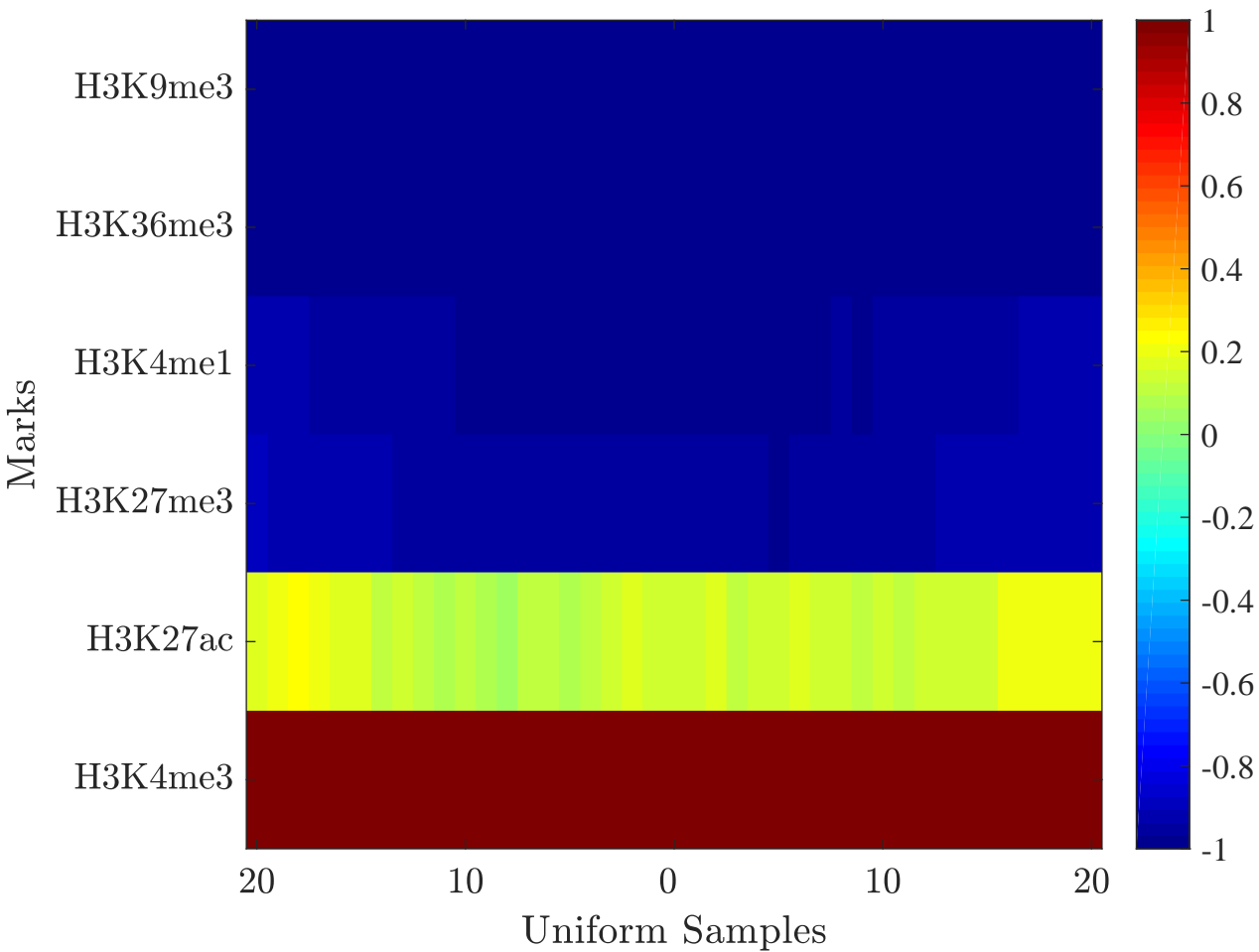

Supplement: Supplementary file 3 — HebbPlots of high-CpG promoters. This compressed file (.tar.gz) includes HebbPlots of high-CpG promoters active in 57 tissues/cell types. (TAR 2654 kb) [file 12859_2018_2312_MOESM3_ESM.tar › file4/E012.pdf]

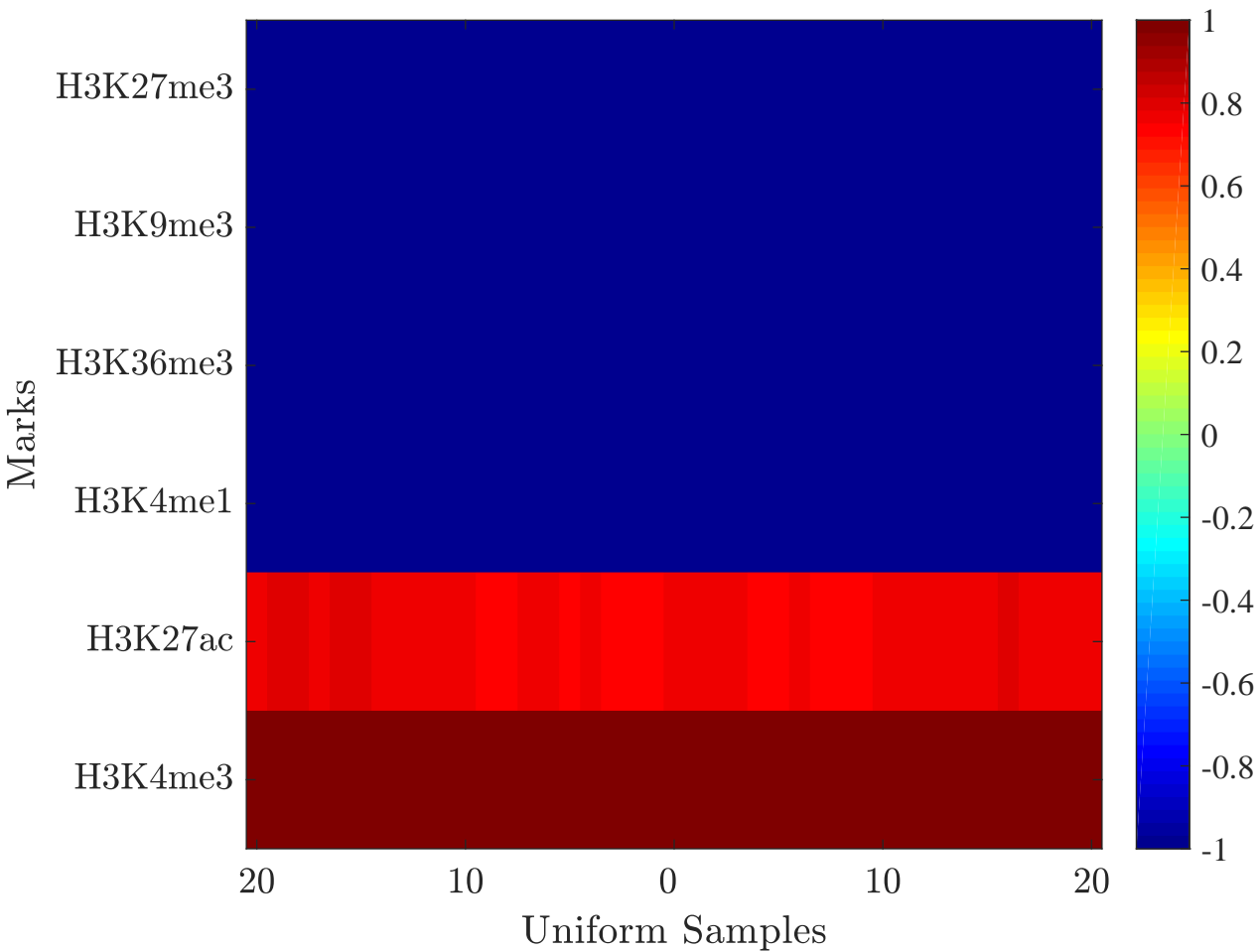

Supplement: Supplementary file 3 — HebbPlots of high-CpG promoters. This compressed file (.tar.gz) includes HebbPlots of high-CpG promoters active in 57 tissues/cell types. (TAR 2654 kb) [file 12859_2018_2312_MOESM3_ESM.tar › file4/E013.pdf]

Marks

H3K36me3

H3K9me3

H3K4me1

H3K27me3

H3K27ac

H3K9ac

H3K4me3

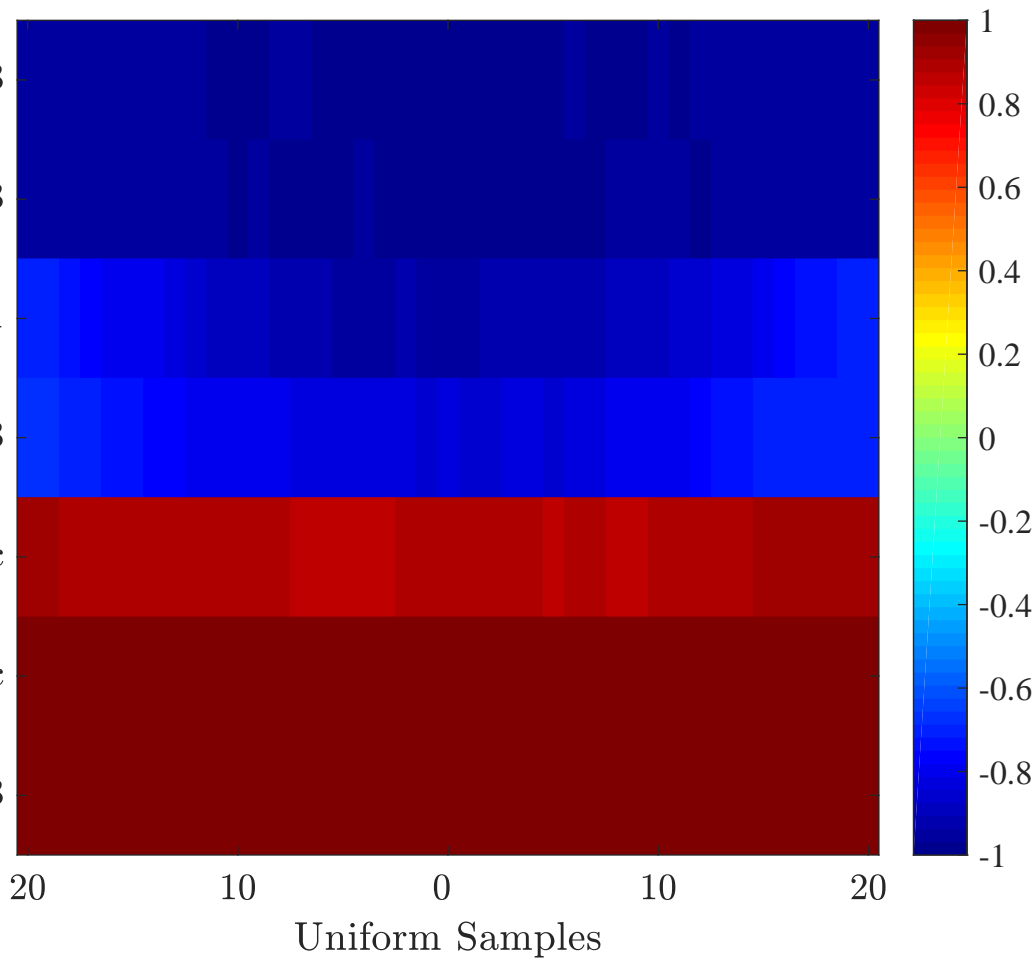

Supplement: Supplementary file 3 — HebbPlots of high-CpG promoters. This compressed file (.tar.gz) includes HebbPlots of high-CpG promoters active in 57 tissues/cell types. (TAR 2654 kb) [file 12859_2018_2312_MOESM3_ESM.tar › file4/E016.pdf]

Marks

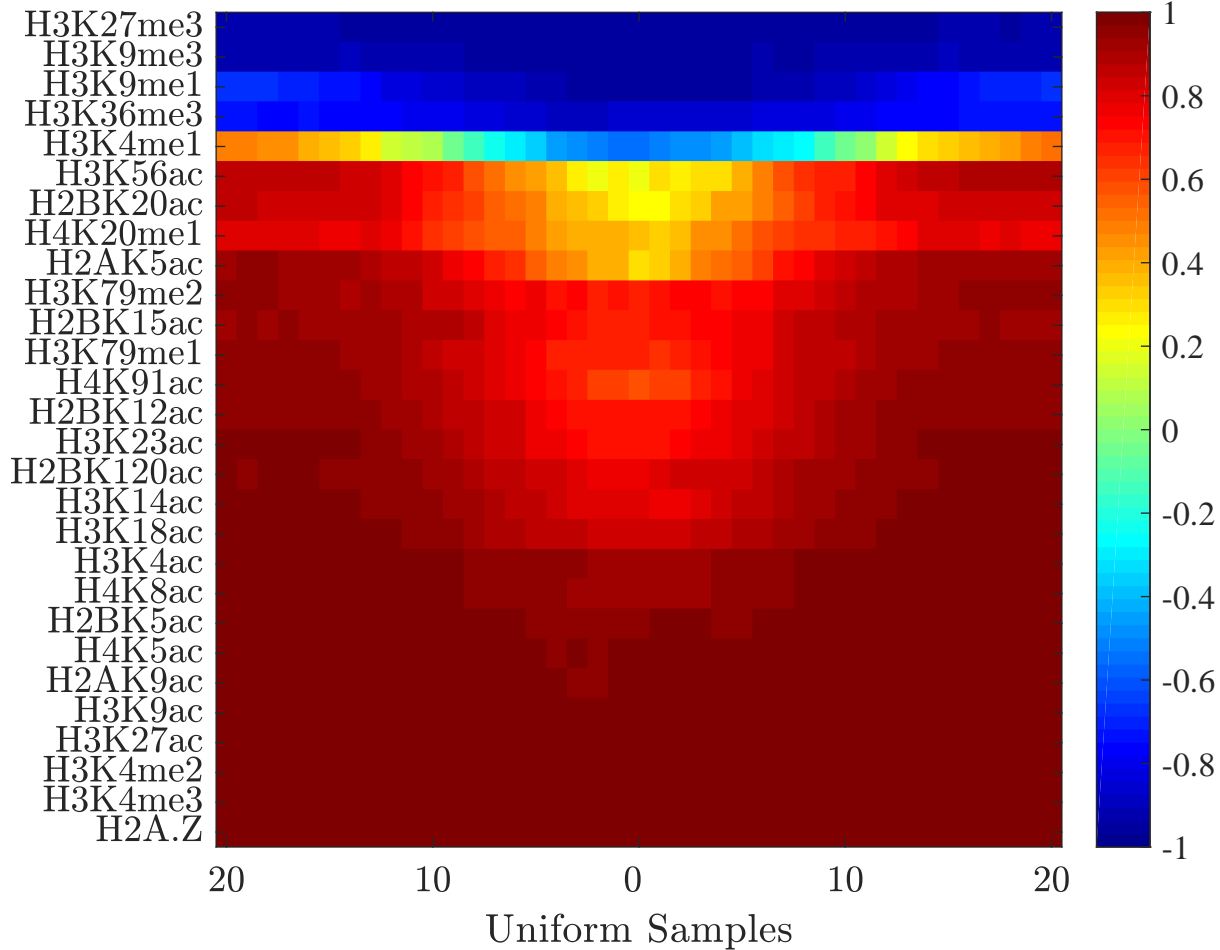

Supplement: Supplementary file 3 — HebbPlots of high-CpG promoters. This compressed file (.tar.gz) includes HebbPlots of high-CpG promoters active in 57 tissues/cell types. (TAR 2654 kb) [file 12859_2018_2312_MOESM3_ESM.tar › file4/E017.pdf]

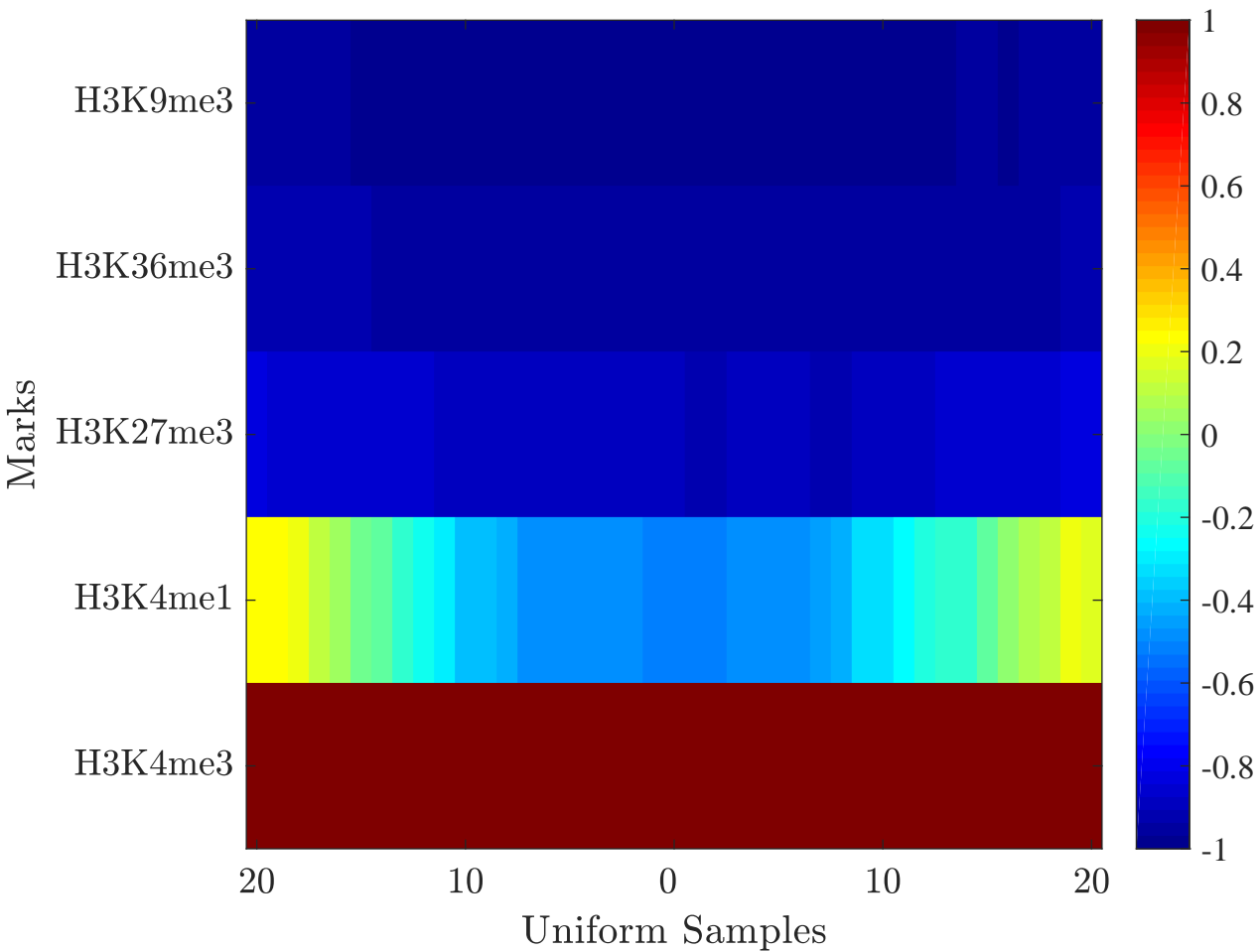

Supplement: Supplementary file 3 — HebbPlots of high-CpG promoters. This compressed file (.tar.gz) includes HebbPlots of high-CpG promoters active in 57 tissues/cell types. (TAR 2654 kb) [file 12859_2018_2312_MOESM3_ESM.tar › file4/E024.pdf]

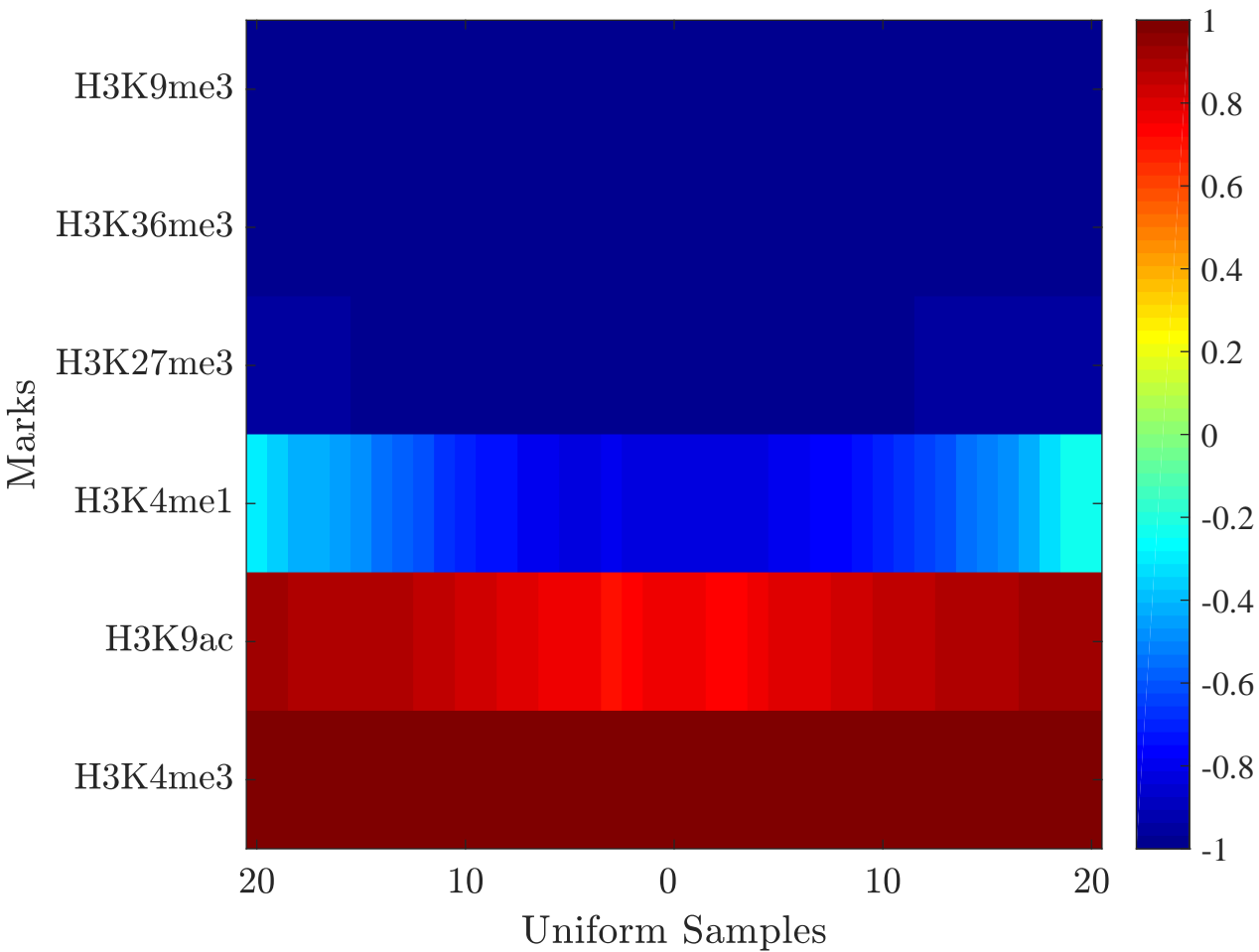

Supplement: Supplementary file 3 — HebbPlots of high-CpG promoters. This compressed file (.tar.gz) includes HebbPlots of high-CpG promoters active in 57 tissues/cell types. (TAR 2654 kb) [file 12859_2018_2312_MOESM3_ESM.tar › file4/E027.pdf]

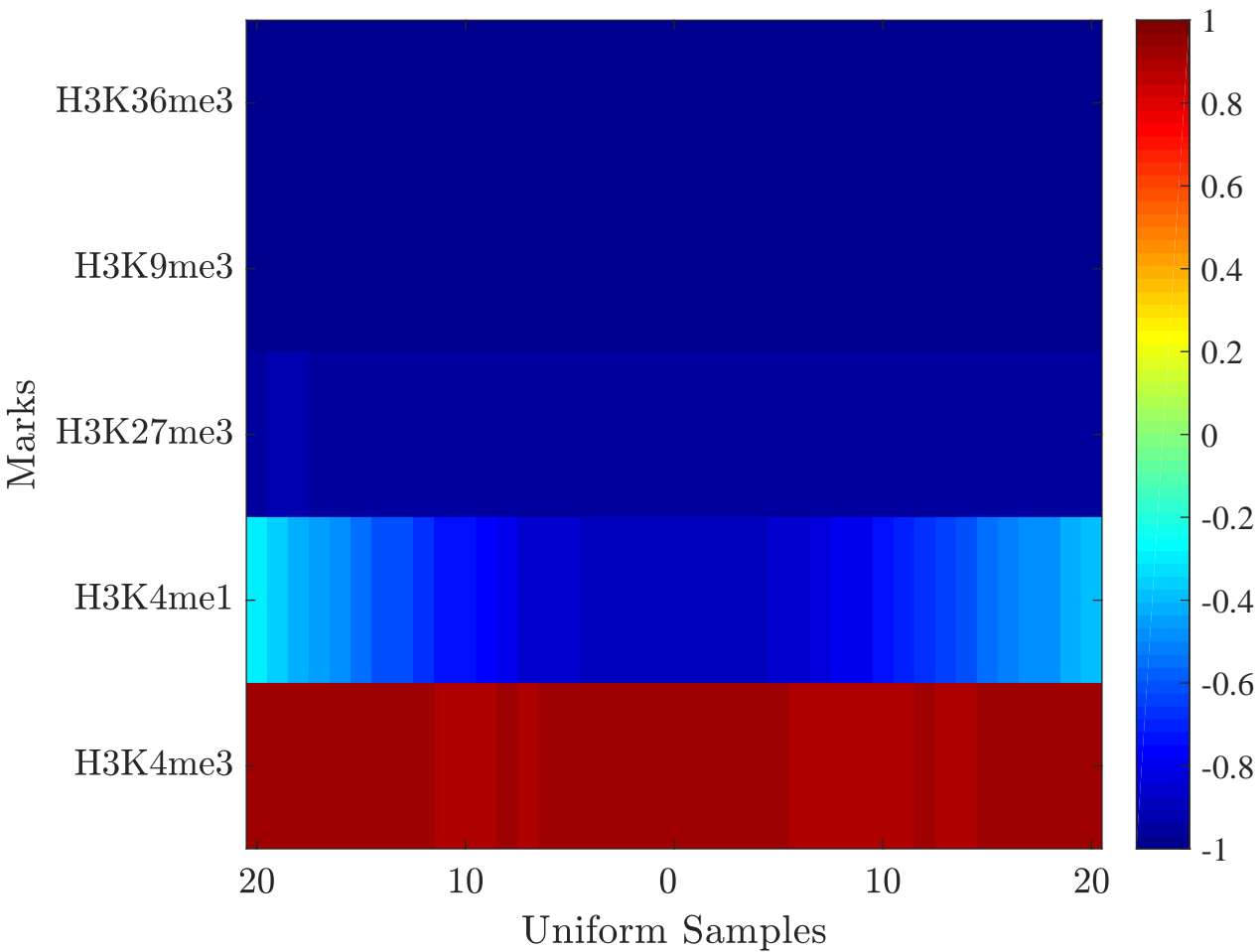

Supplement: Supplementary file 3 — HebbPlots of high-CpG promoters. This compressed file (.tar.gz) includes HebbPlots of high-CpG promoters active in 57 tissues/cell types. (TAR 2654 kb) [file 12859_2018_2312_MOESM3_ESM.tar › file4/E028.pdf]

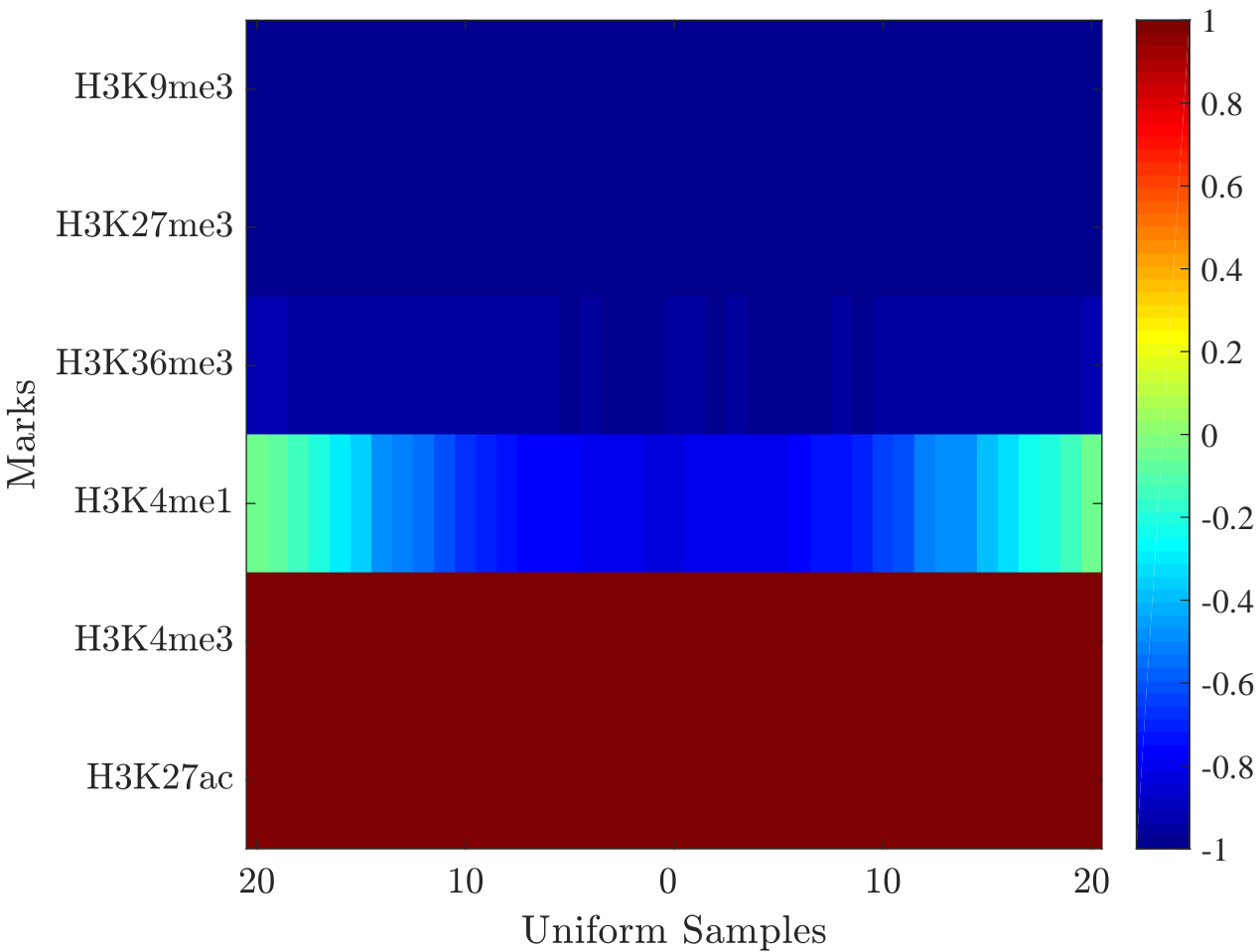

Supplement: Supplementary file 3 — HebbPlots of high-CpG promoters. This compressed file (.tar.gz) includes HebbPlots of high-CpG promoters active in 57 tissues/cell types. (TAR 2654 kb) [file 12859_2018_2312_MOESM3_ESM.tar › file4/E037.pdf]

Marks

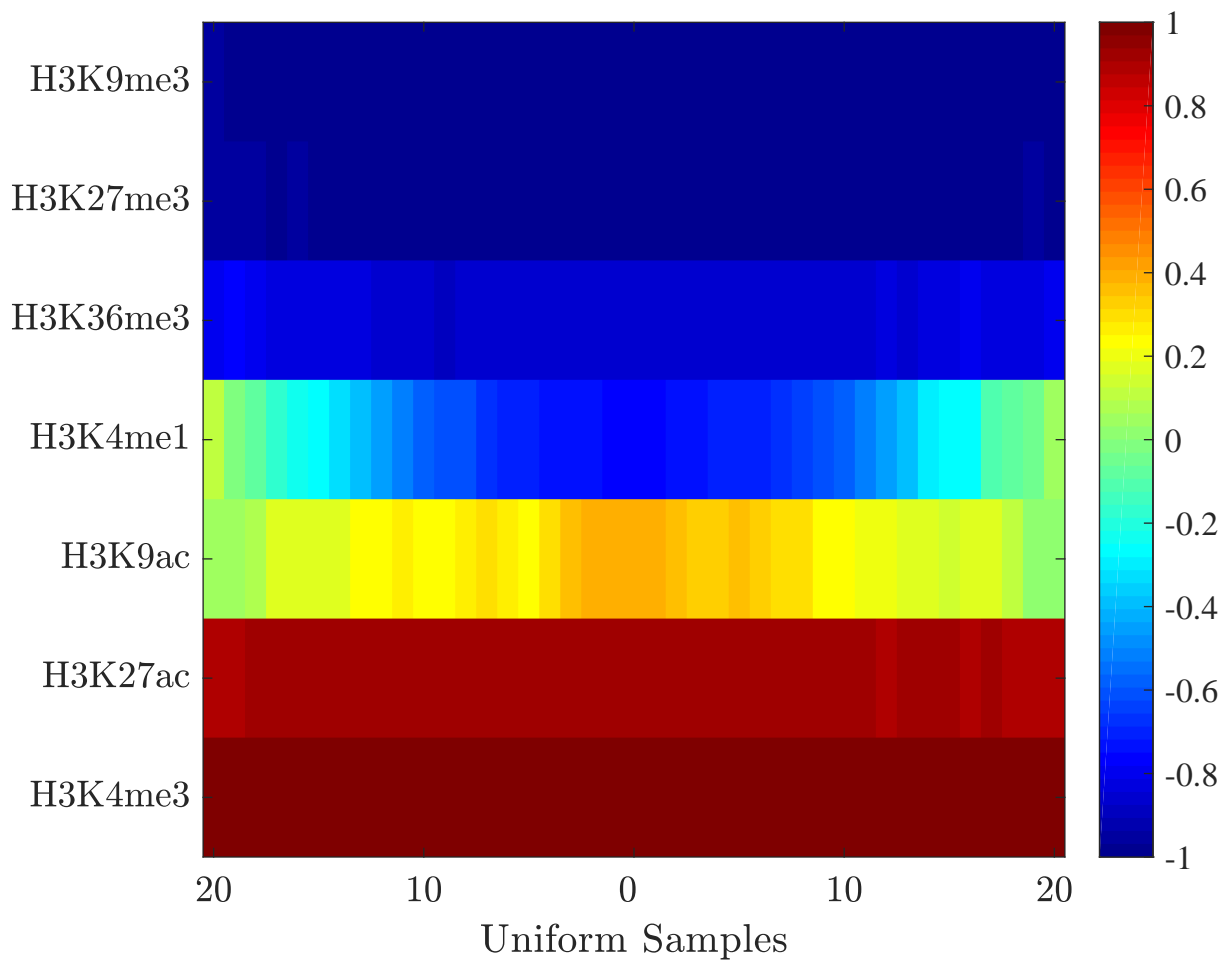

Supplement: Supplementary file 3 — HebbPlots of high-CpG promoters. This compressed file (.tar.gz) includes HebbPlots of high-CpG promoters active in 57 tissues/cell types. (TAR 2654 kb) [file 12859_2018_2312_MOESM3_ESM.tar › file4/E038.pdf]

Marks

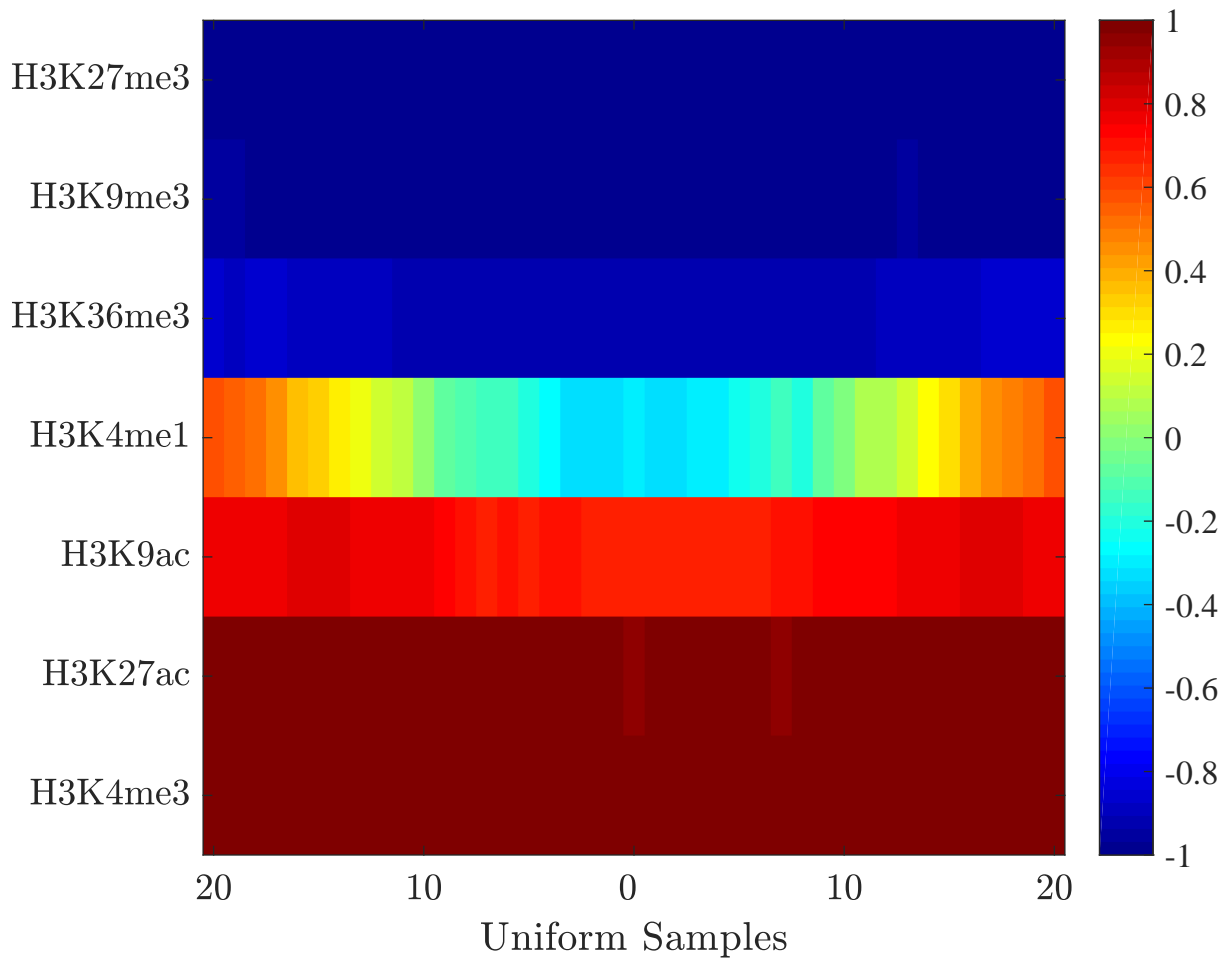

Supplement: Supplementary file 3 — HebbPlots of high-CpG promoters. This compressed file (.tar.gz) includes HebbPlots of high-CpG promoters active in 57 tissues/cell types. (TAR 2654 kb) [file 12859_2018_2312_MOESM3_ESM.tar › file4/E047.pdf]

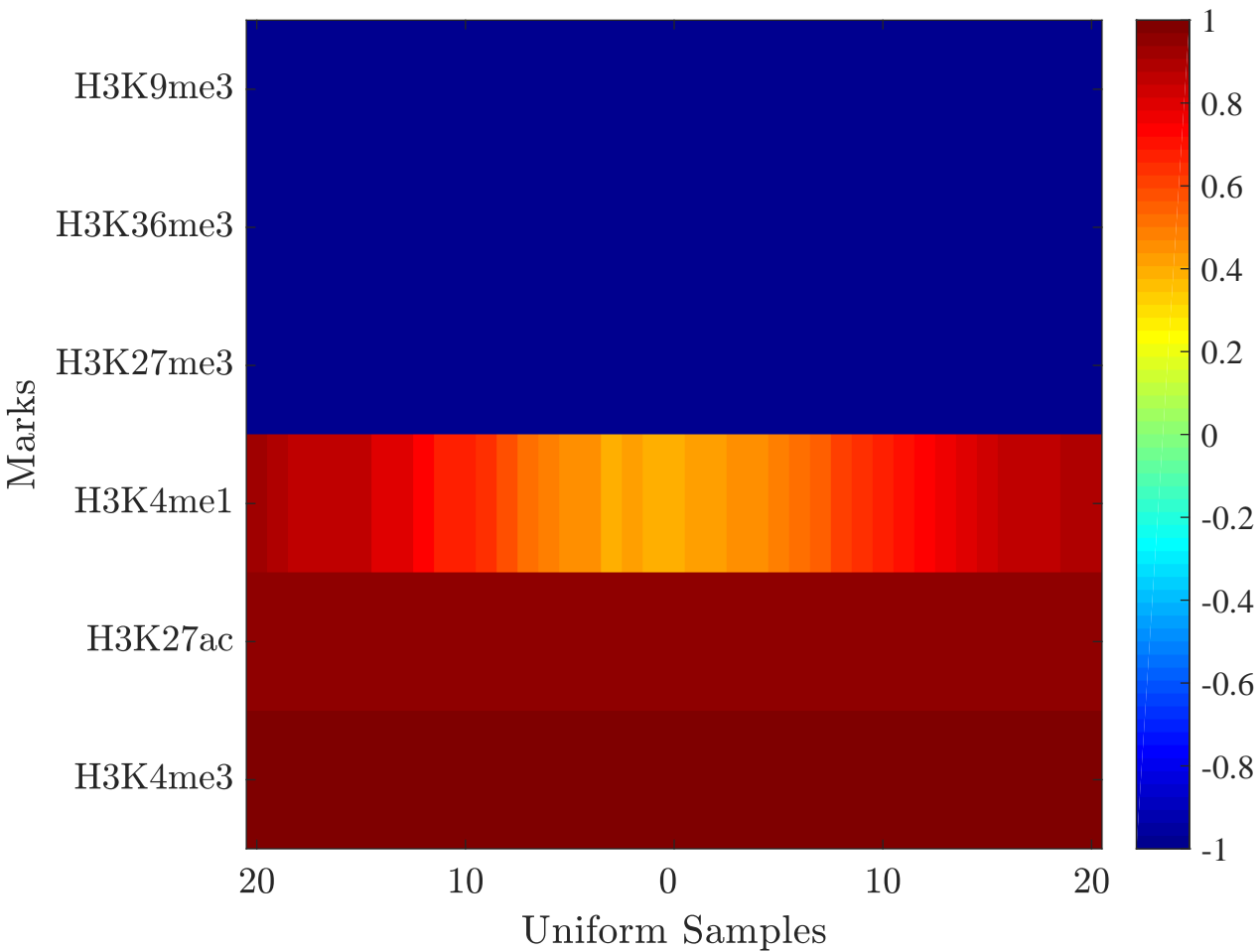

Supplement: Supplementary file 3 — HebbPlots of high-CpG promoters. This compressed file (.tar.gz) includes HebbPlots of high-CpG promoters active in 57 tissues/cell types. (TAR 2654 kb) [file 12859_2018_2312_MOESM3_ESM.tar › file4/E050.pdf]

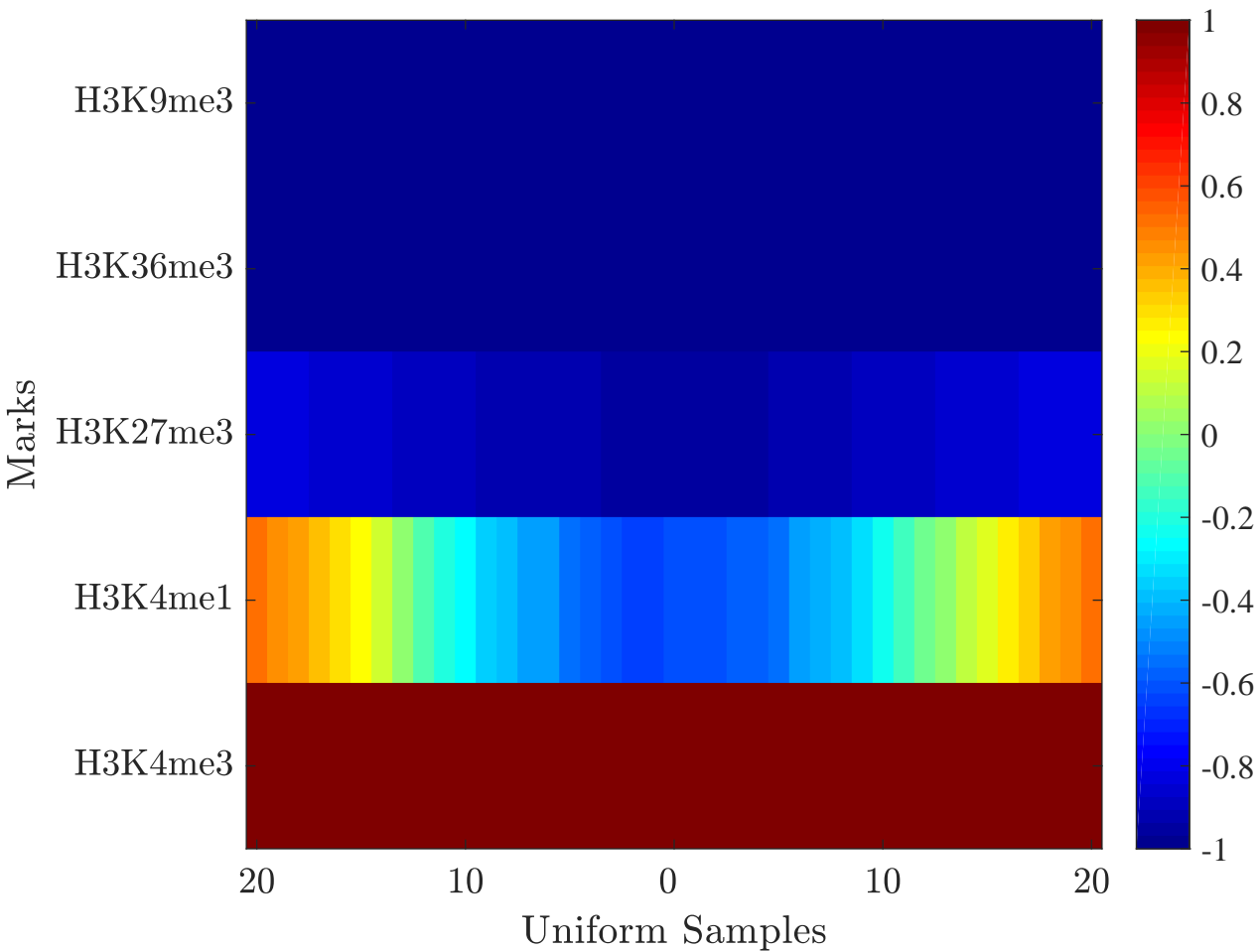

Supplement: Supplementary file 3 — HebbPlots of high-CpG promoters. This compressed file (.tar.gz) includes HebbPlots of high-CpG promoters active in 57 tissues/cell types. (TAR 2654 kb) [file 12859_2018_2312_MOESM3_ESM.tar › file4/E053.pdf]

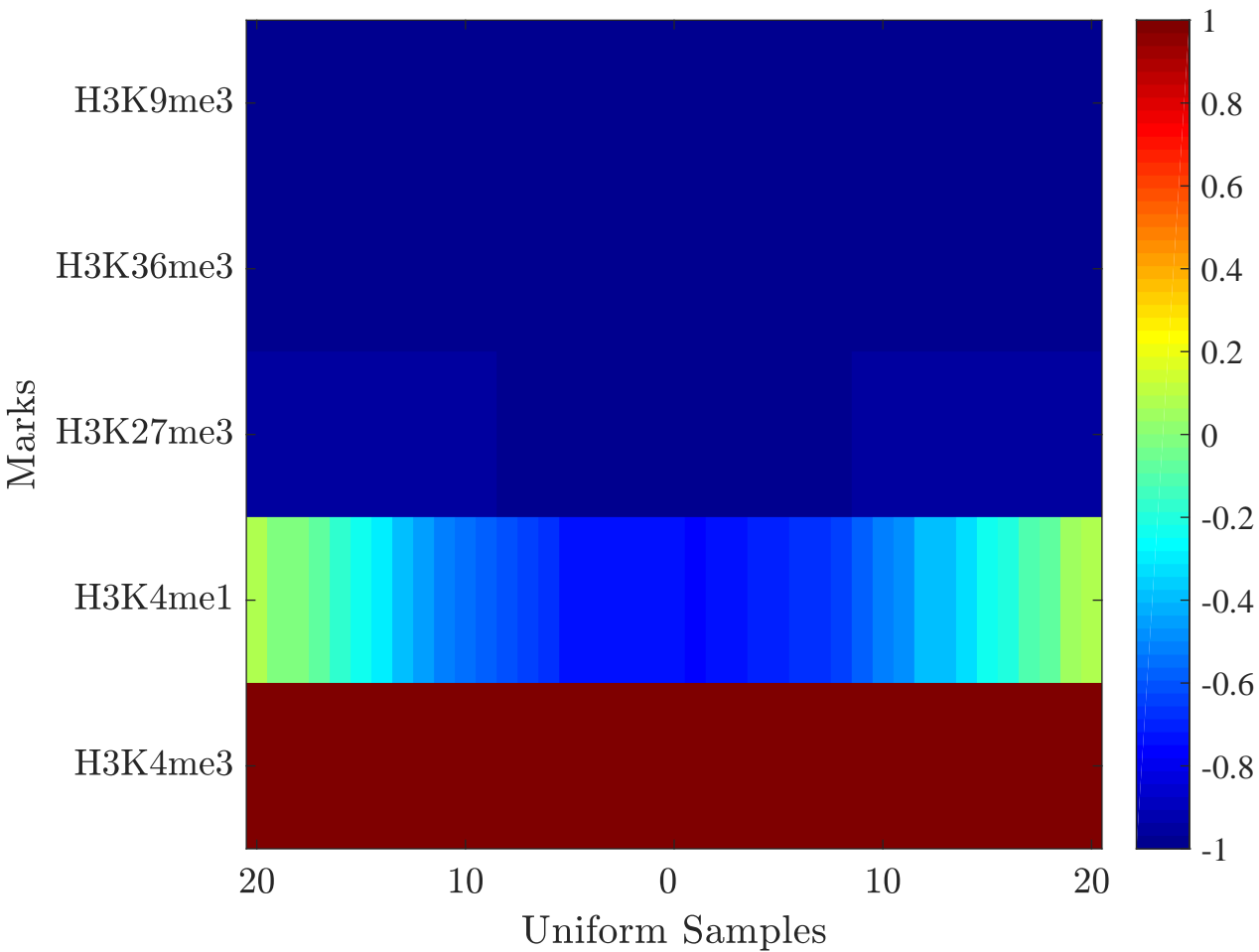

Supplement: Supplementary file 3 — HebbPlots of high-CpG promoters. This compressed file (.tar.gz) includes HebbPlots of high-CpG promoters active in 57 tissues/cell types. (TAR 2654 kb) [file 12859_2018_2312_MOESM3_ESM.tar › file4/E054.pdf]

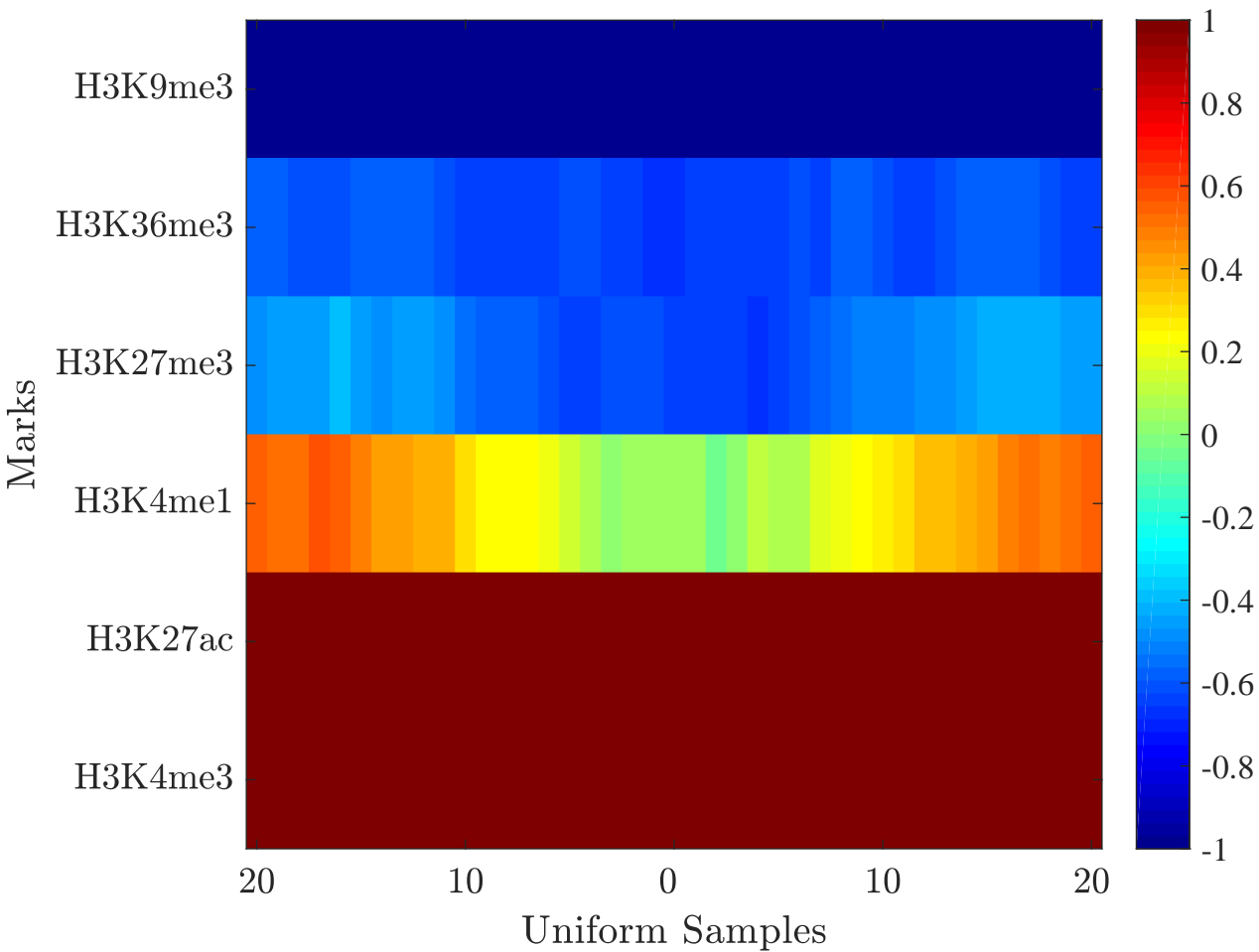

Supplement: Supplementary file 3 — HebbPlots of high-CpG promoters. This compressed file (.tar.gz) includes HebbPlots of high-CpG promoters active in 57 tissues/cell types. (TAR 2654 kb) [file 12859_2018_2312_MOESM3_ESM.tar › file4/E055.pdf]

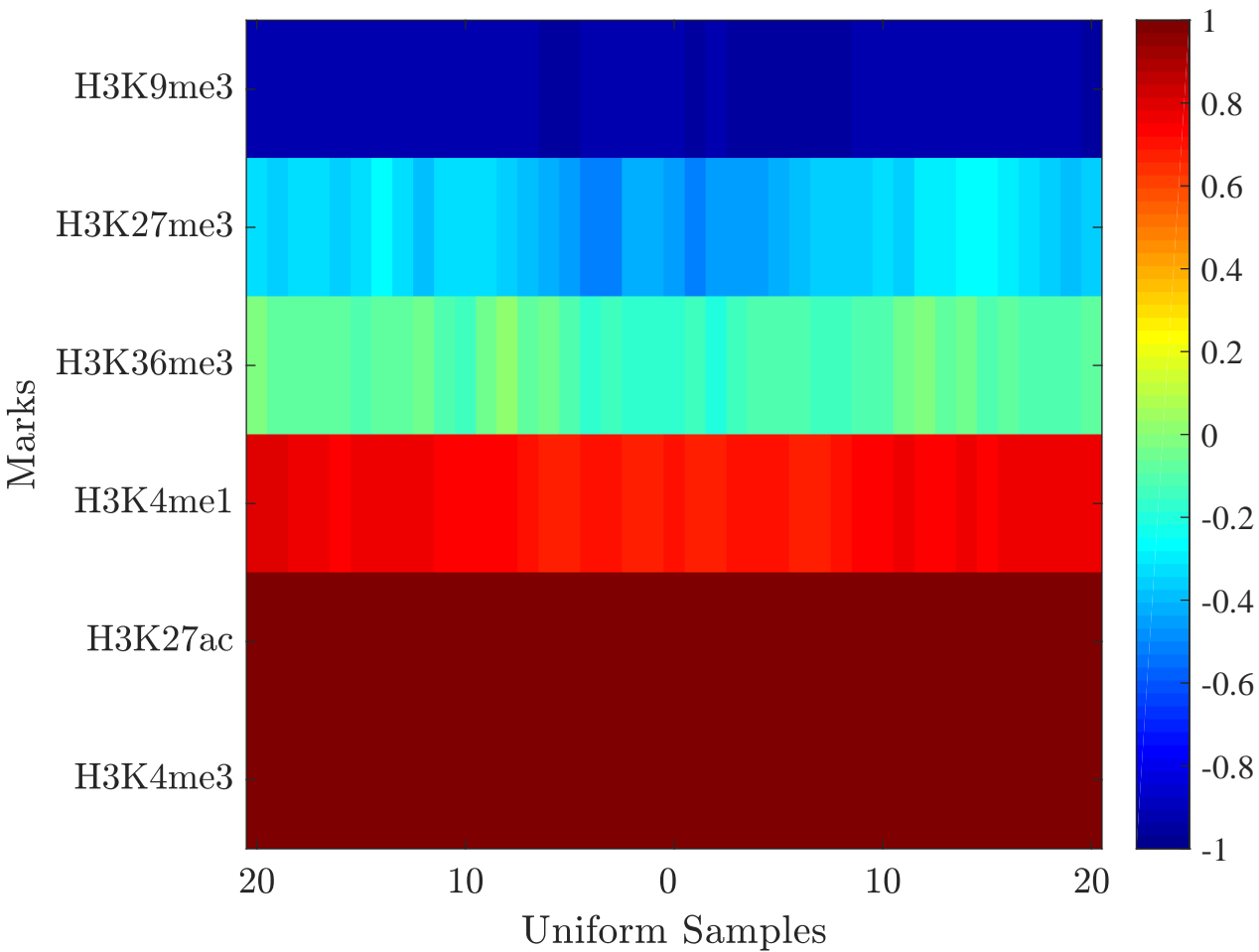

Supplement: Supplementary file 3 — HebbPlots of high-CpG promoters. This compressed file (.tar.gz) includes HebbPlots of high-CpG promoters active in 57 tissues/cell types. (TAR 2654 kb) [file 12859_2018_2312_MOESM3_ESM.tar › file4/E056.pdf]

Marks

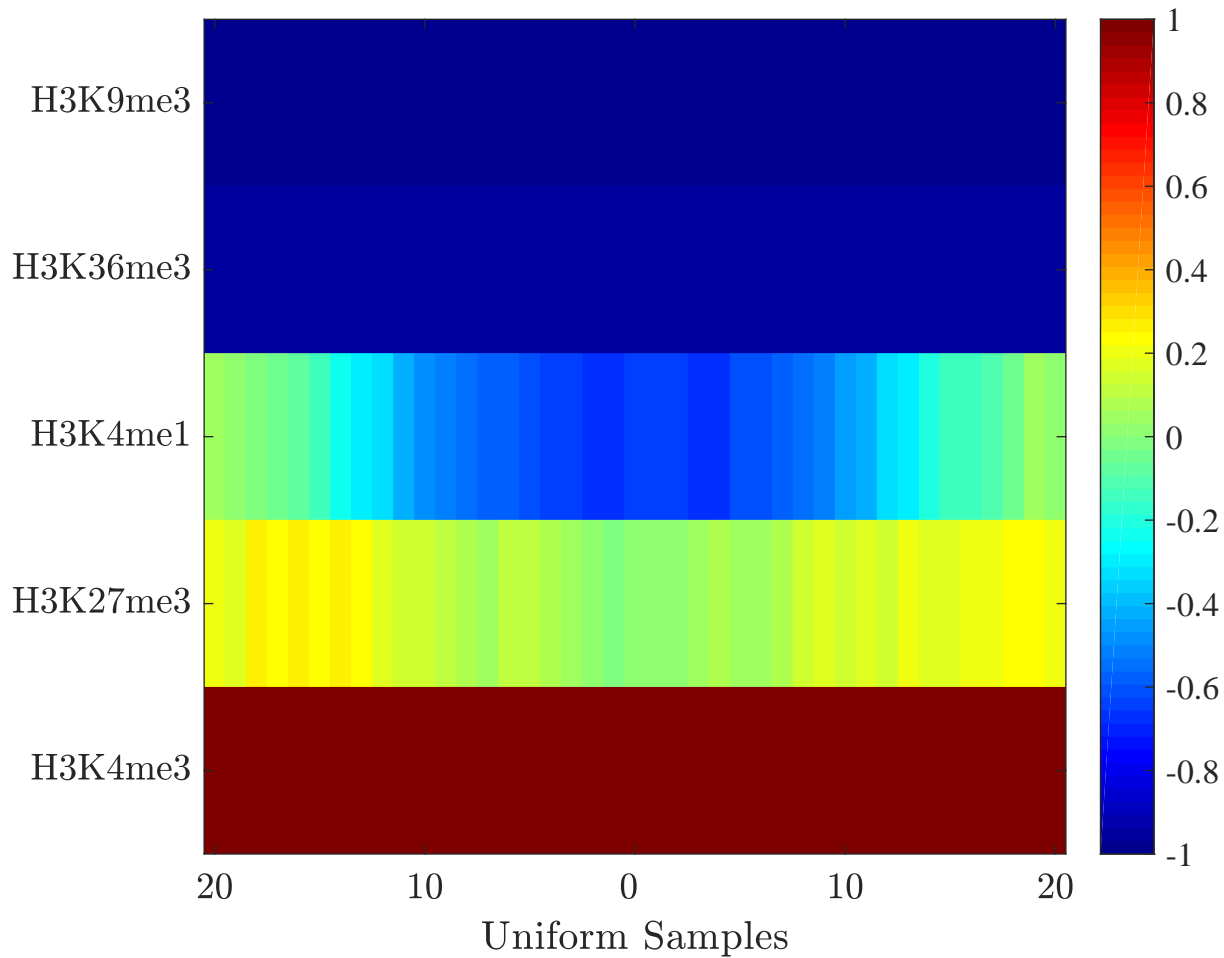

Supplement: Supplementary file 3 — HebbPlots of high-CpG promoters. This compressed file (.tar.gz) includes HebbPlots of high-CpG promoters active in 57 tissues/cell types. (TAR 2654 kb) [file 12859_2018_2312_MOESM3_ESM.tar › file4/E057.pdf]

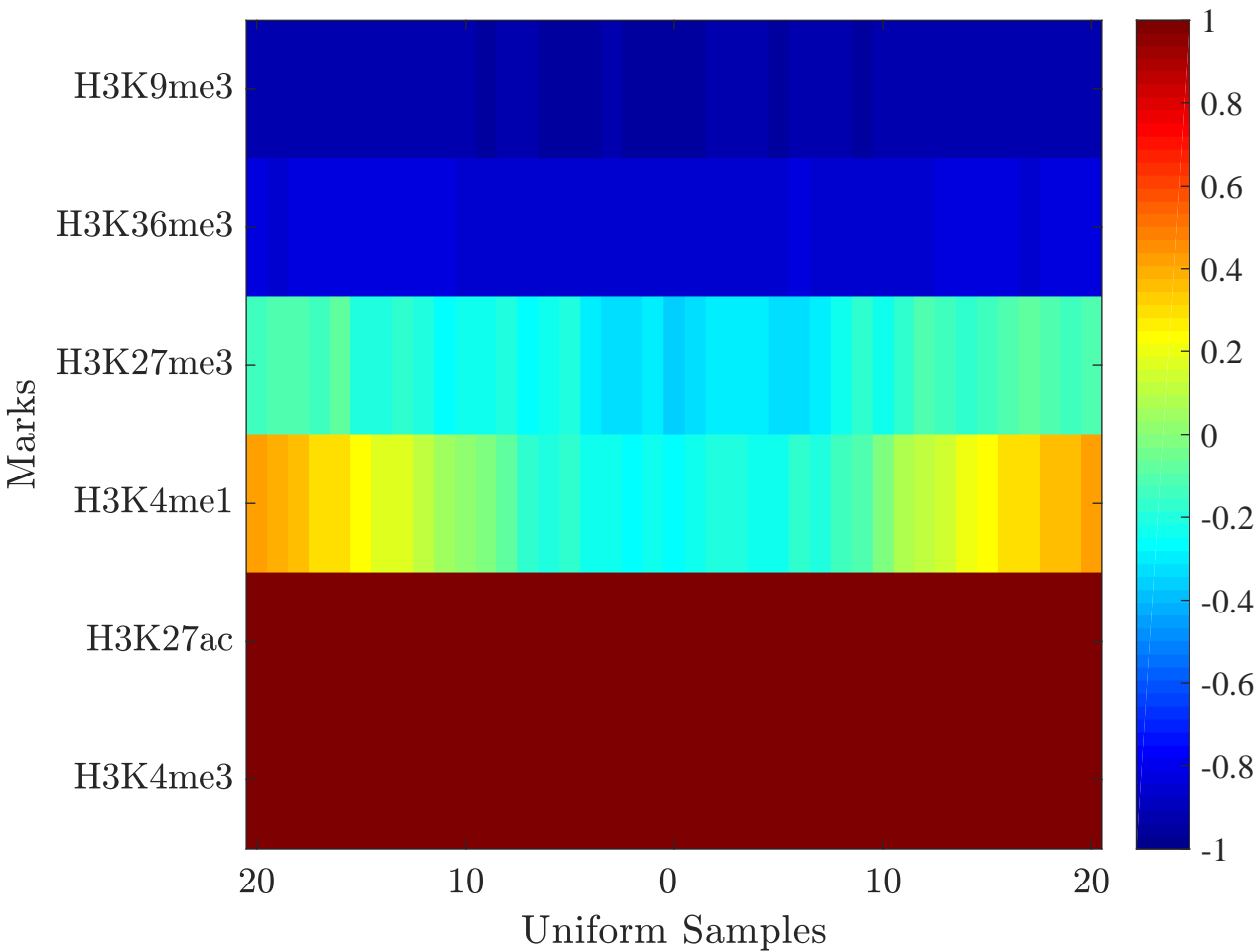

Supplement: Supplementary file 3 — HebbPlots of high-CpG promoters. This compressed file (.tar.gz) includes HebbPlots of high-CpG promoters active in 57 tissues/cell types. (TAR 2654 kb) [file 12859_2018_2312_MOESM3_ESM.tar › file4/E058.pdf]

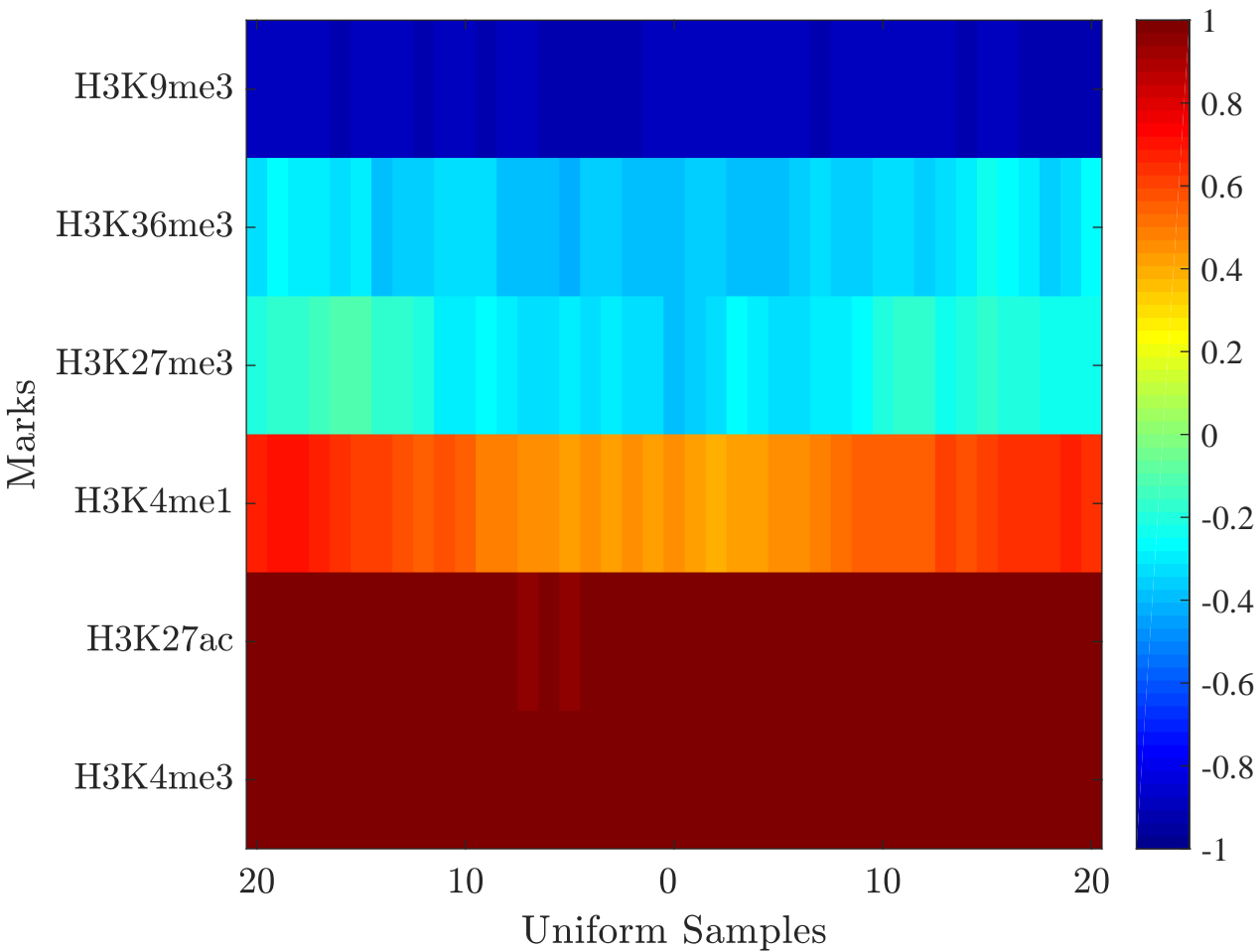

Supplement: Supplementary file 3 — HebbPlots of high-CpG promoters. This compressed file (.tar.gz) includes HebbPlots of high-CpG promoters active in 57 tissues/cell types. (TAR 2654 kb) [file 12859_2018_2312_MOESM3_ESM.tar › file4/E059.pdf]

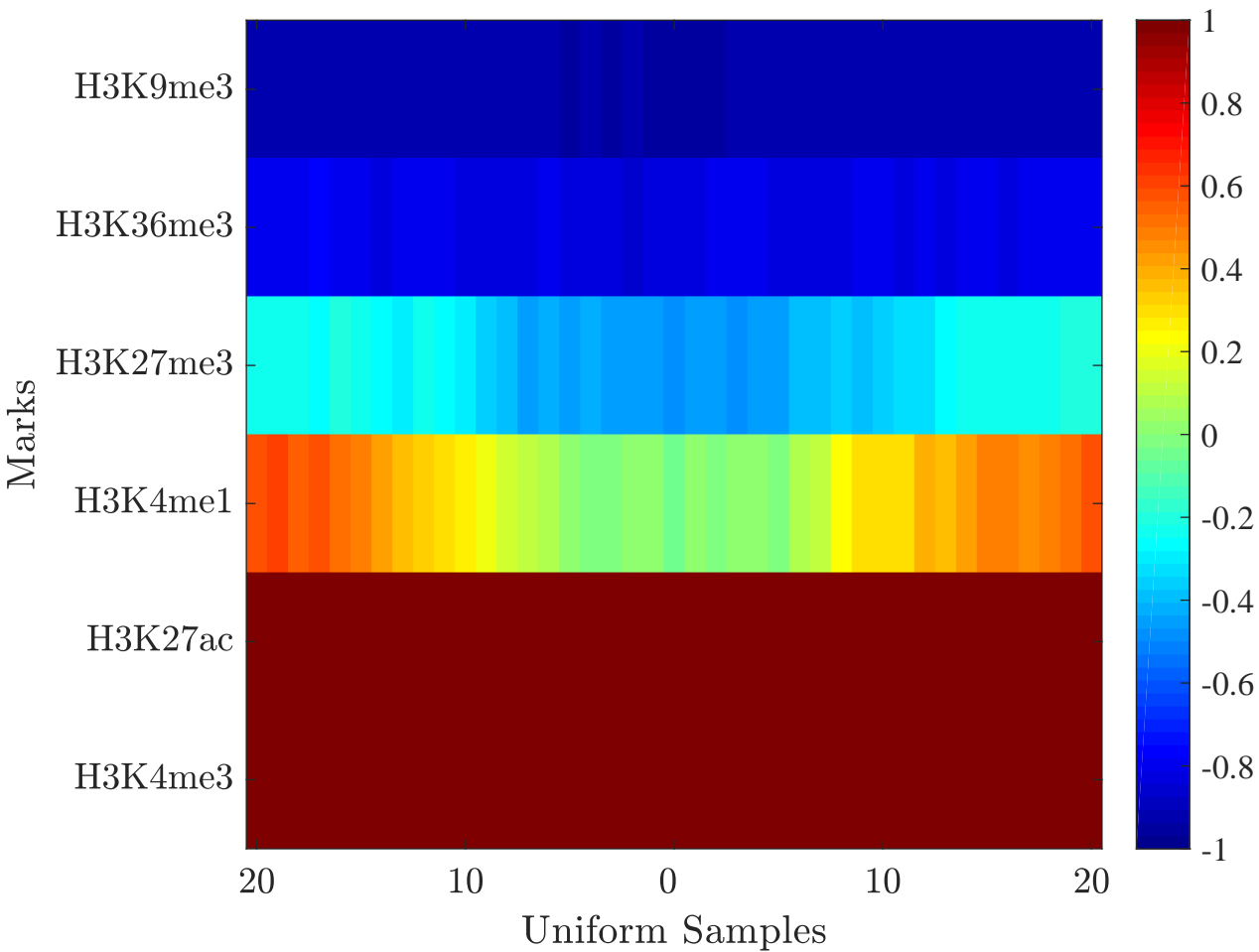

Supplement: Supplementary file 3 — HebbPlots of high-CpG promoters. This compressed file (.tar.gz) includes HebbPlots of high-CpG promoters active in 57 tissues/cell types. (TAR 2654 kb) [file 12859_2018_2312_MOESM3_ESM.tar › file4/E061.pdf]

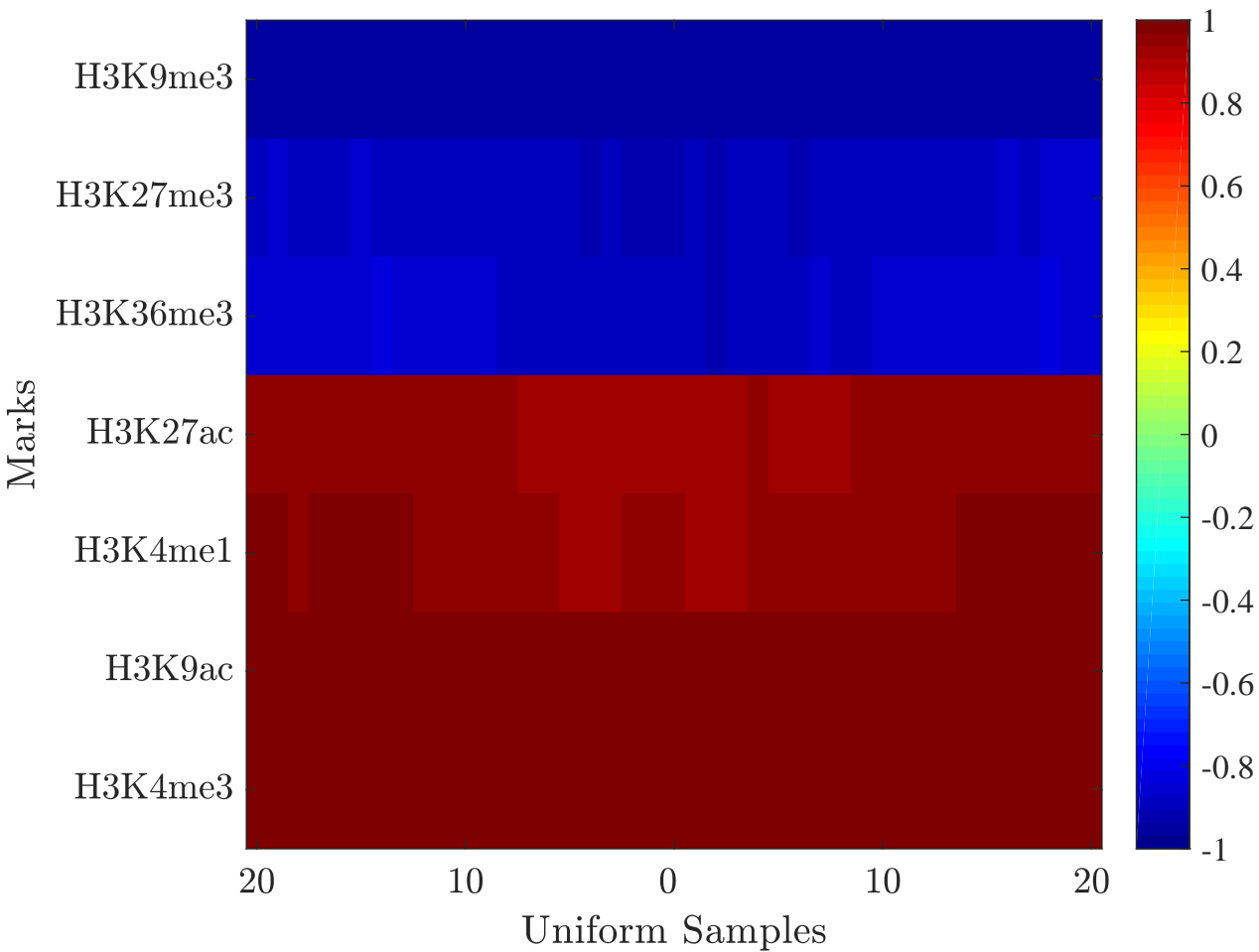

Supplement: Supplementary file 3 — HebbPlots of high-CpG promoters. This compressed file (.tar.gz) includes HebbPlots of high-CpG promoters active in 57 tissues/cell types. (TAR 2654 kb) [file 12859_2018_2312_MOESM3_ESM.tar › file4/E062.pdf]

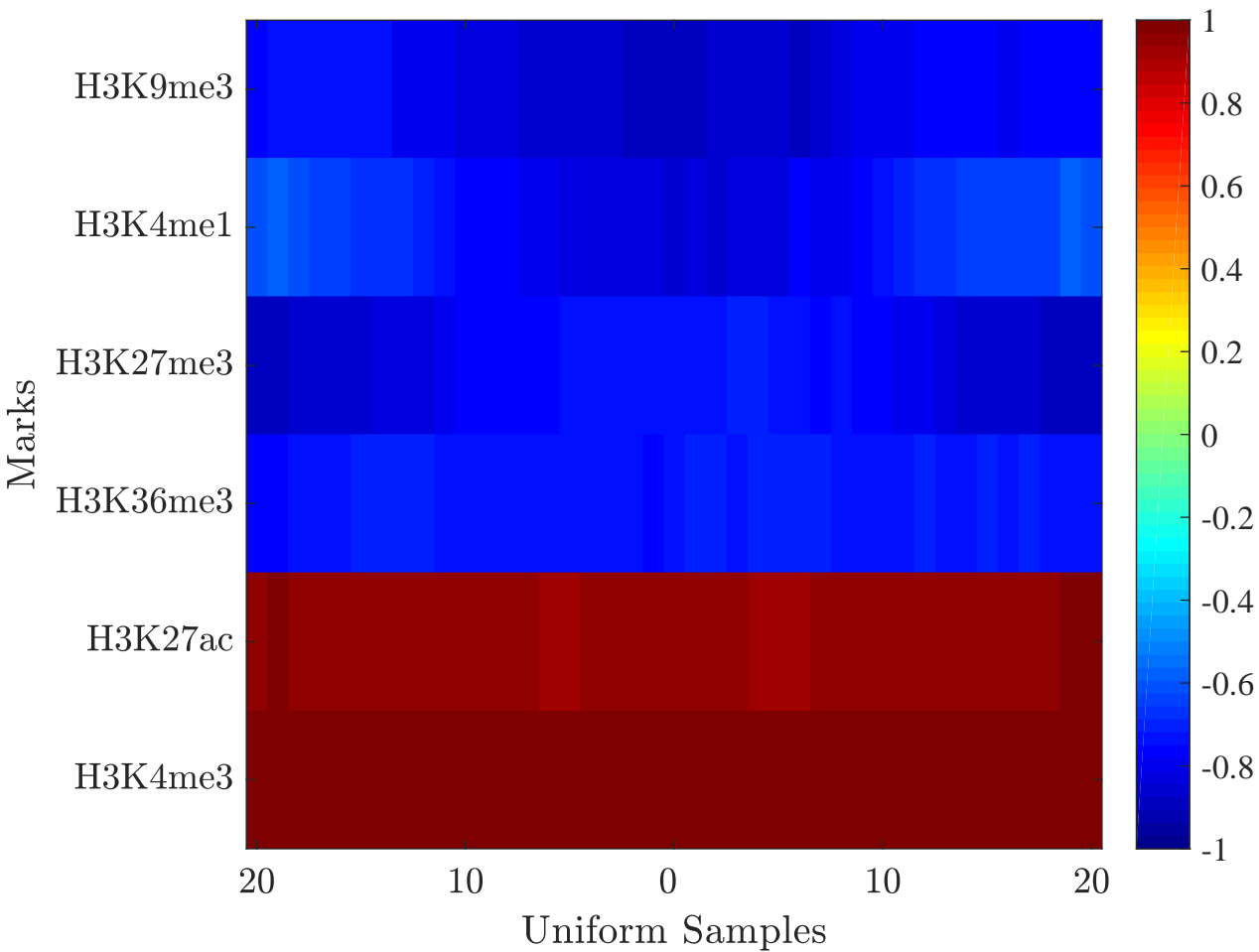

Supplement: Supplementary file 3 — HebbPlots of high-CpG promoters. This compressed file (.tar.gz) includes HebbPlots of high-CpG promoters active in 57 tissues/cell types. (TAR 2654 kb) [file 12859_2018_2312_MOESM3_ESM.tar › file4/E065.pdf]

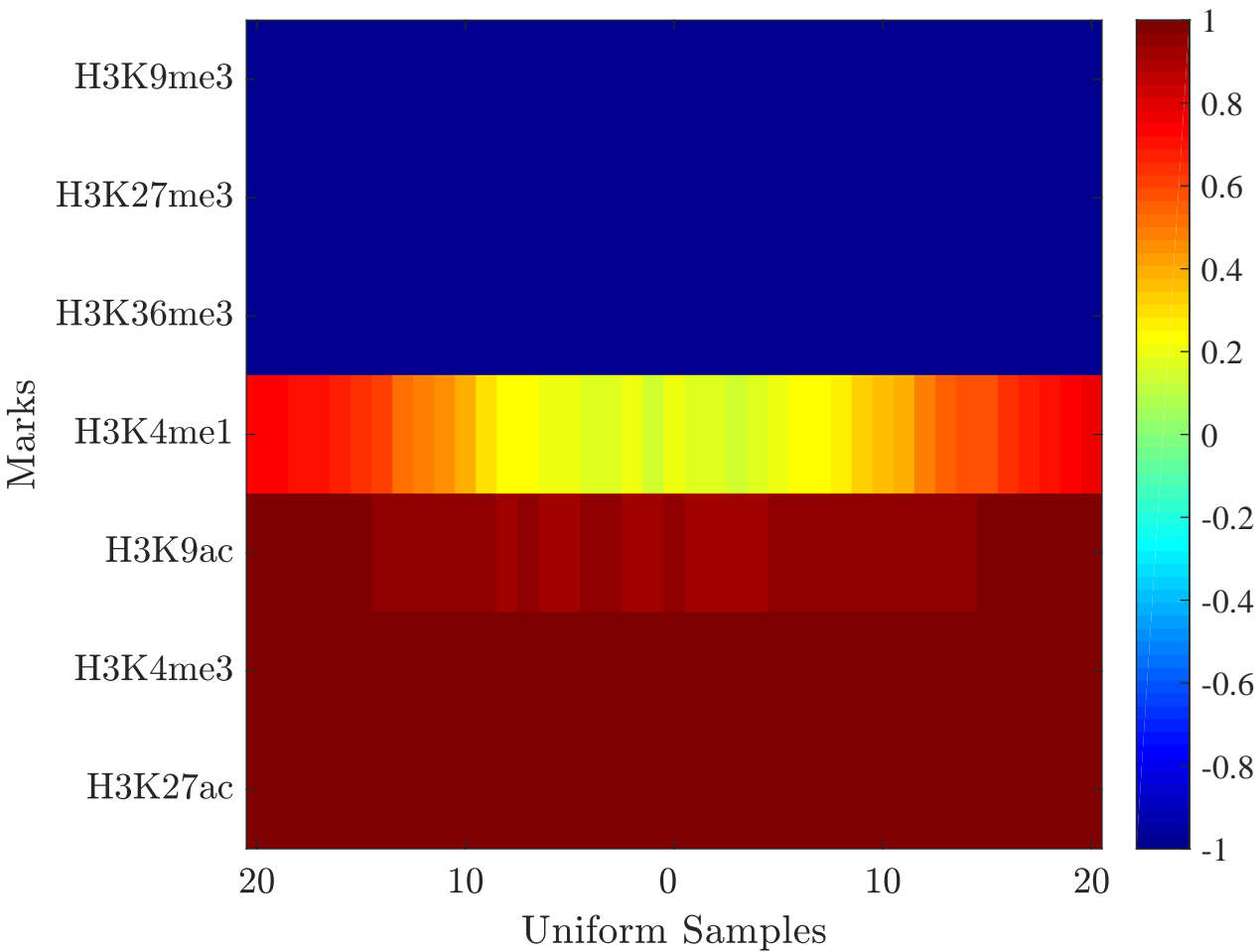

Supplement: Supplementary file 3 — HebbPlots of high-CpG promoters. This compressed file (.tar.gz) includes HebbPlots of high-CpG promoters active in 57 tissues/cell types. (TAR 2654 kb) [file 12859_2018_2312_MOESM3_ESM.tar › file4/E066.pdf]

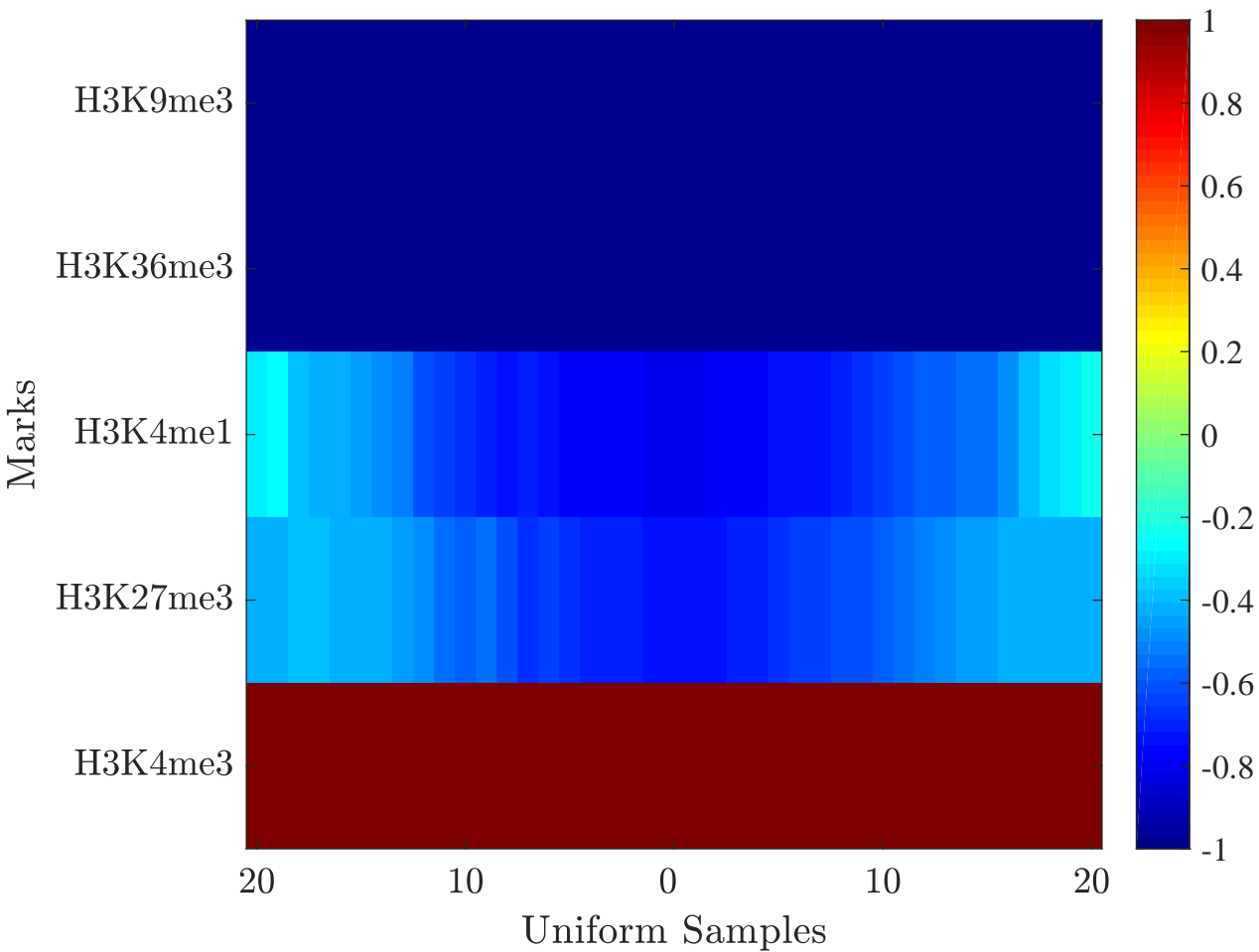

Supplement: Supplementary file 3 — HebbPlots of high-CpG promoters. This compressed file (.tar.gz) includes HebbPlots of high-CpG promoters active in 57 tissues/cell types. (TAR 2654 kb) [file 12859_2018_2312_MOESM3_ESM.tar › file4/E070.pdf]

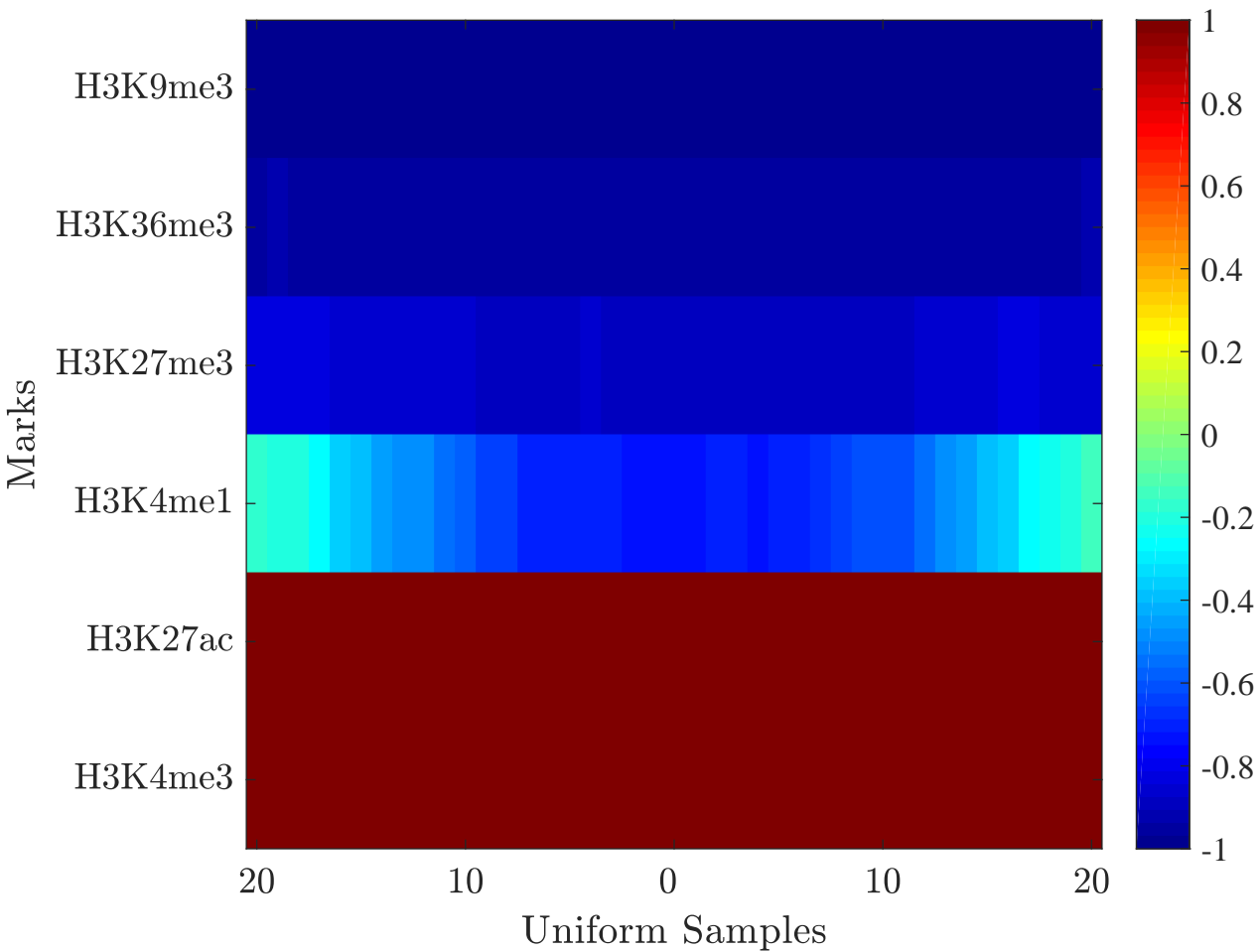

Supplement: Supplementary file 3 — HebbPlots of high-CpG promoters. This compressed file (.tar.gz) includes HebbPlots of high-CpG promoters active in 57 tissues/cell types. (TAR 2654 kb) [file 12859_2018_2312_MOESM3_ESM.tar › file4/E071.pdf]

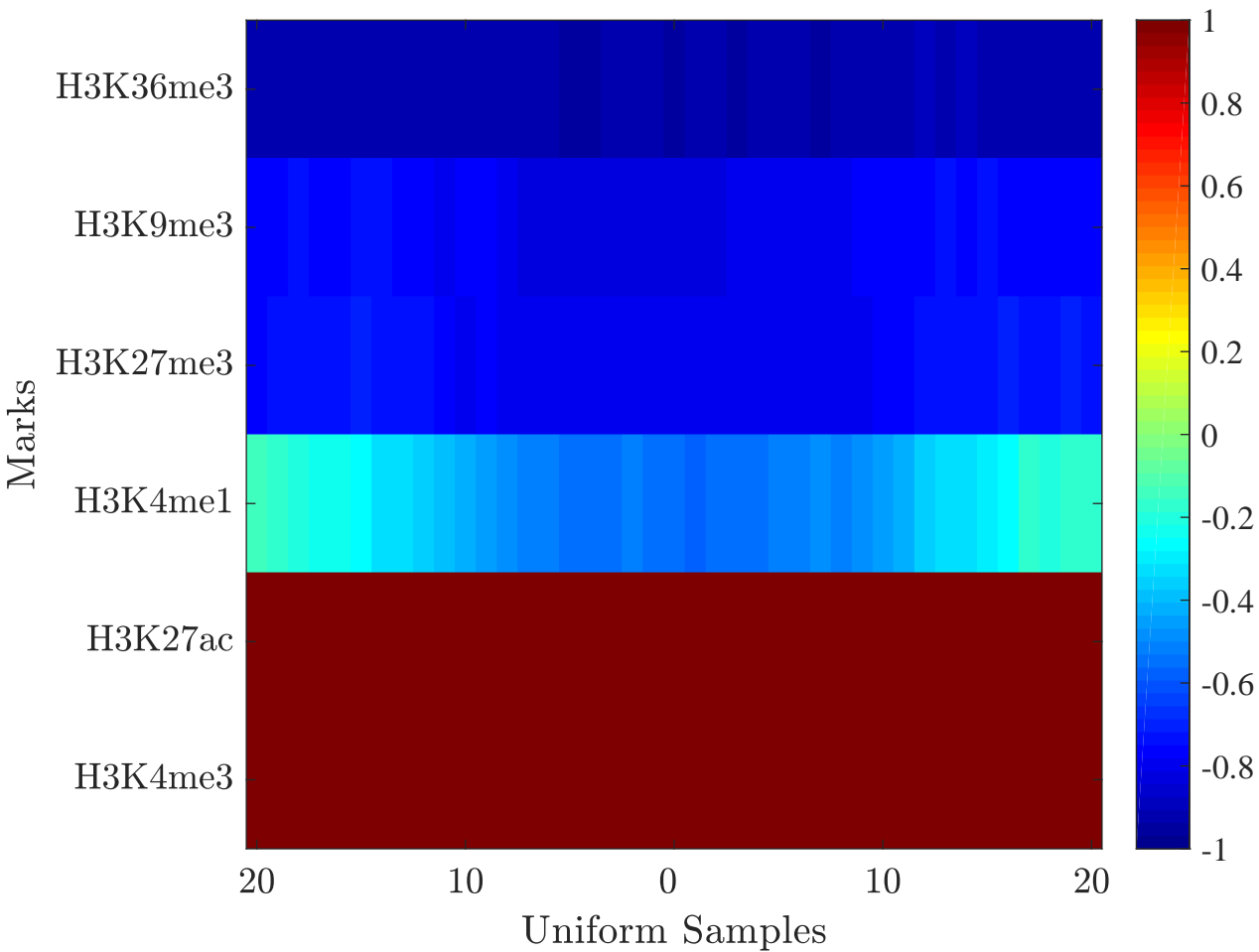

Supplement: Supplementary file 3 — HebbPlots of high-CpG promoters. This compressed file (.tar.gz) includes HebbPlots of high-CpG promoters active in 57 tissues/cell types. (TAR 2654 kb) [file 12859_2018_2312_MOESM3_ESM.tar › file4/E079.pdf]

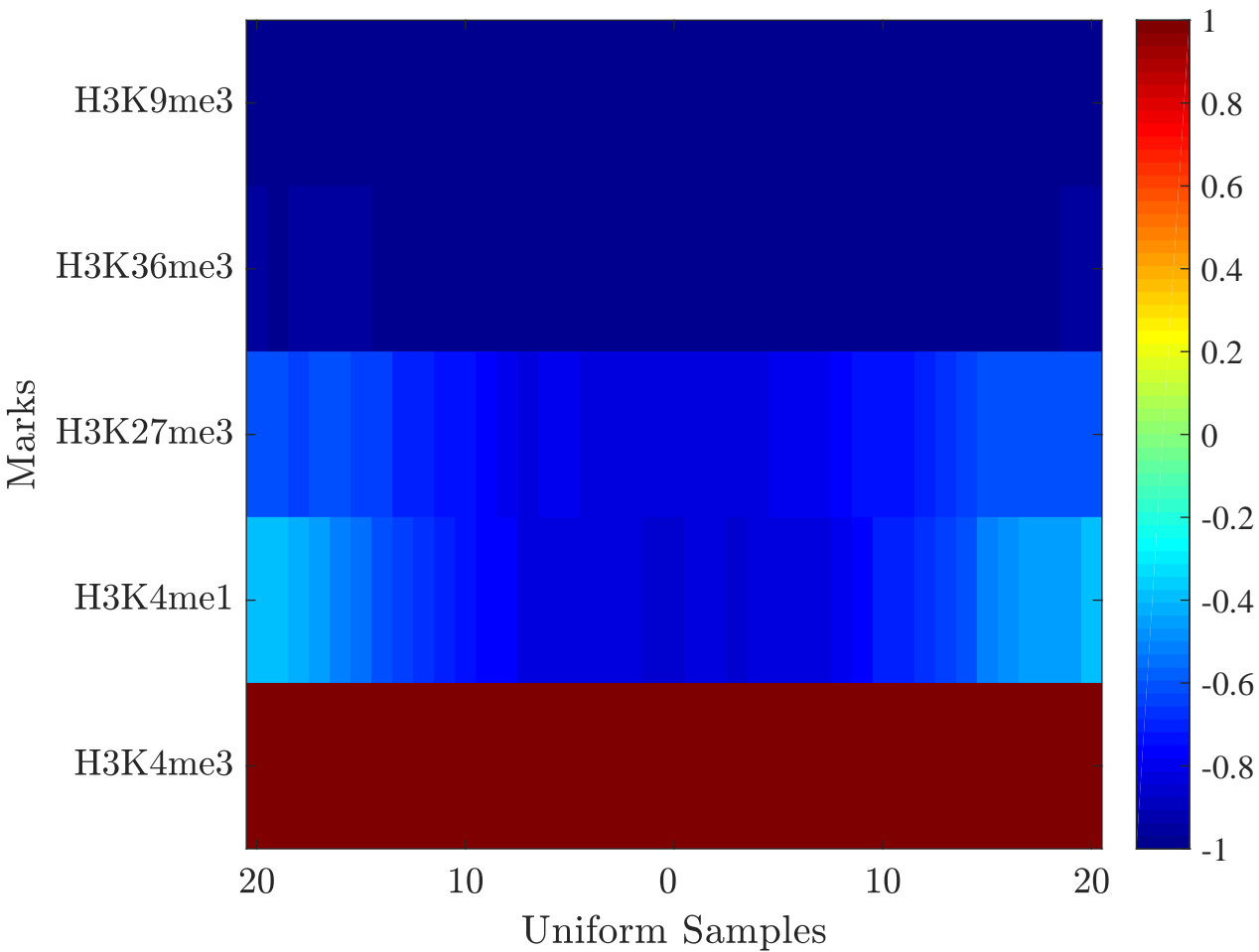

Supplement: Supplementary file 3 — HebbPlots of high-CpG promoters. This compressed file (.tar.gz) includes HebbPlots of high-CpG promoters active in 57 tissues/cell types. (TAR 2654 kb) [file 12859_2018_2312_MOESM3_ESM.tar › file4/E082.pdf]

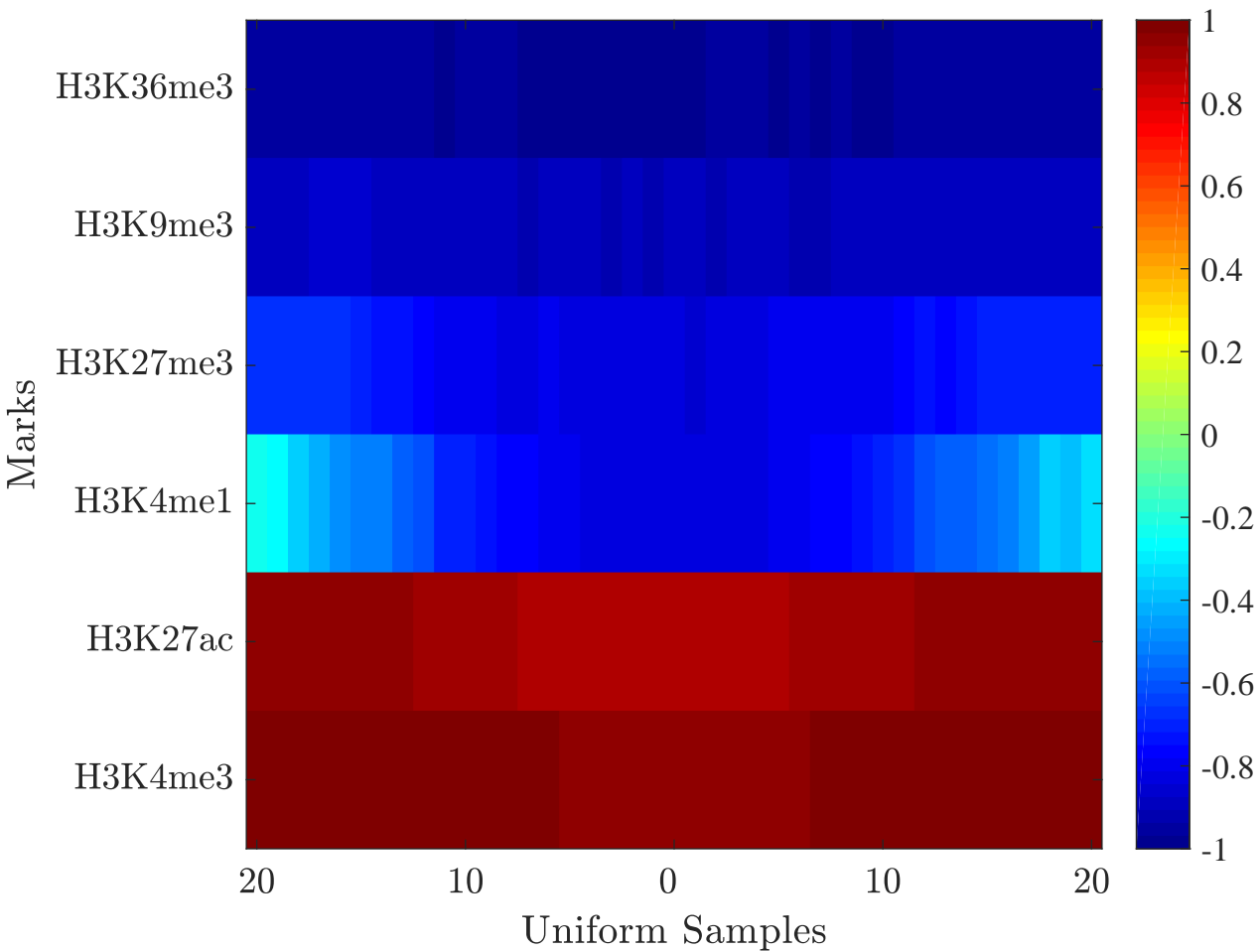

Supplement: Supplementary file 3 — HebbPlots of high-CpG promoters. This compressed file (.tar.gz) includes HebbPlots of high-CpG promoters active in 57 tissues/cell types. (TAR 2654 kb) [file 12859_2018_2312_MOESM3_ESM.tar › file4/E084.pdf]

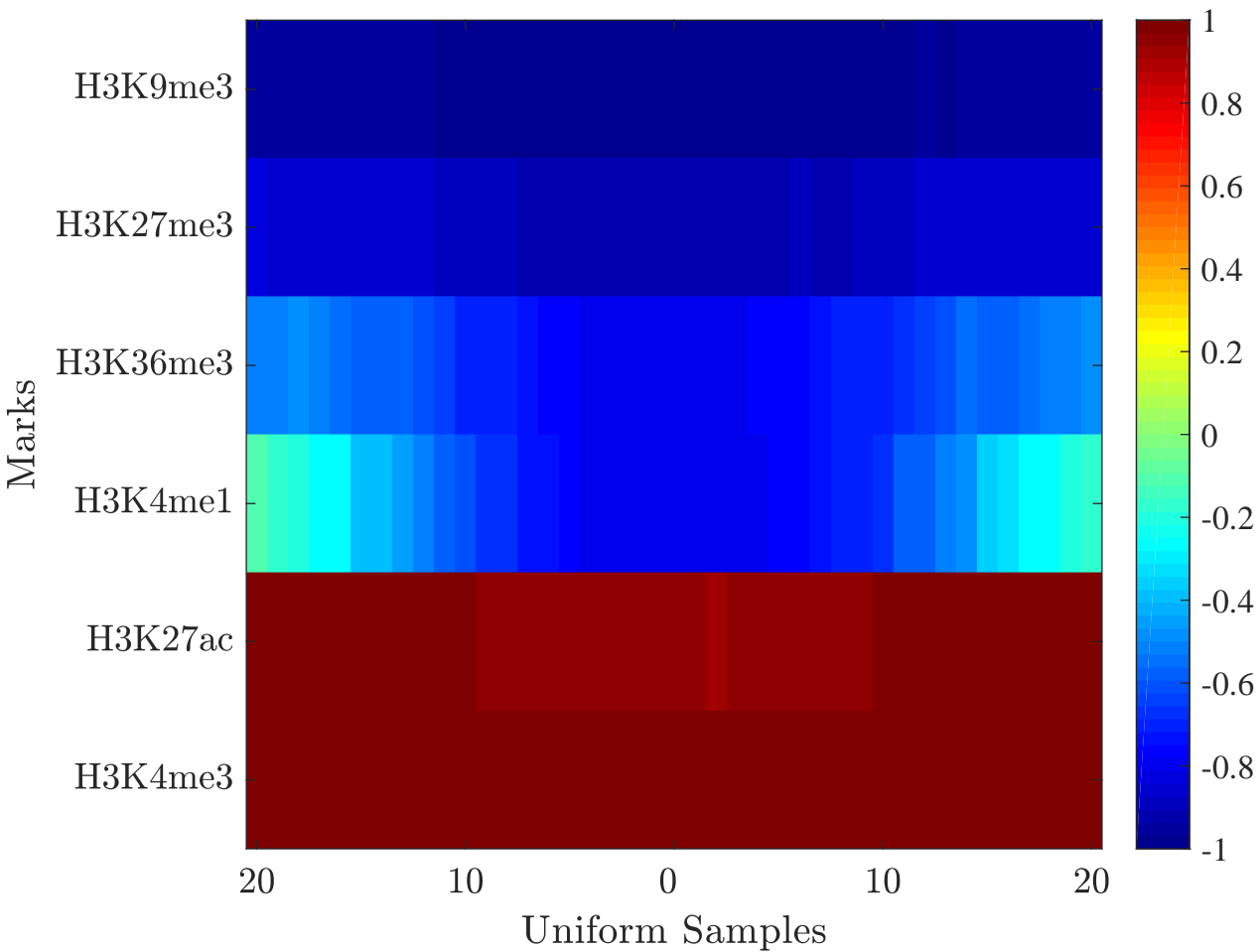

Supplement: Supplementary file 3 — HebbPlots of high-CpG promoters. This compressed file (.tar.gz) includes HebbPlots of high-CpG promoters active in 57 tissues/cell types. (TAR 2654 kb) [file 12859_2018_2312_MOESM3_ESM.tar › file4/E085.pdf]

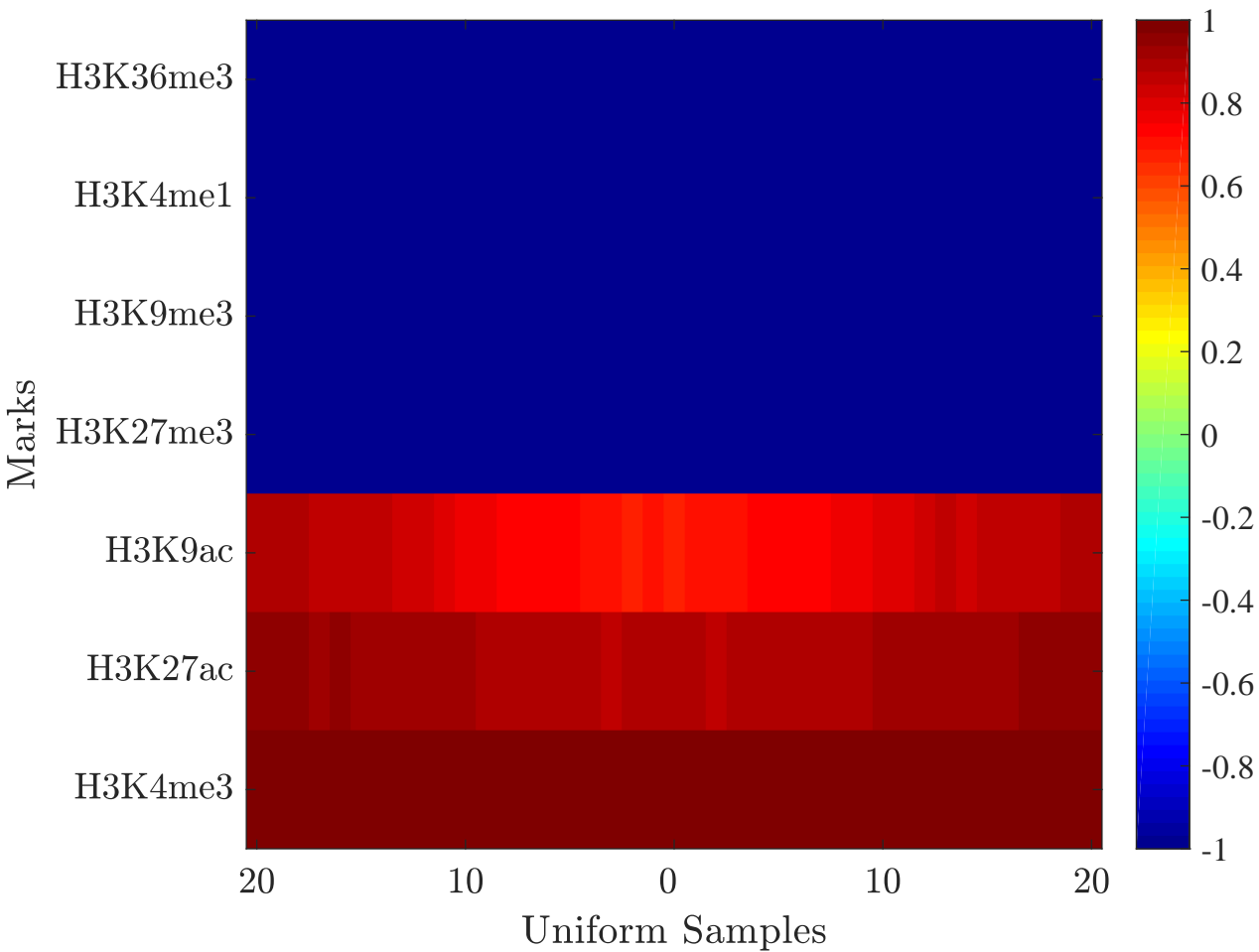

Supplement: Supplementary file 3 — HebbPlots of high-CpG promoters. This compressed file (.tar.gz) includes HebbPlots of high-CpG promoters active in 57 tissues/cell types. (TAR 2654 kb) [file 12859_2018_2312_MOESM3_ESM.tar › file4/E087.pdf]

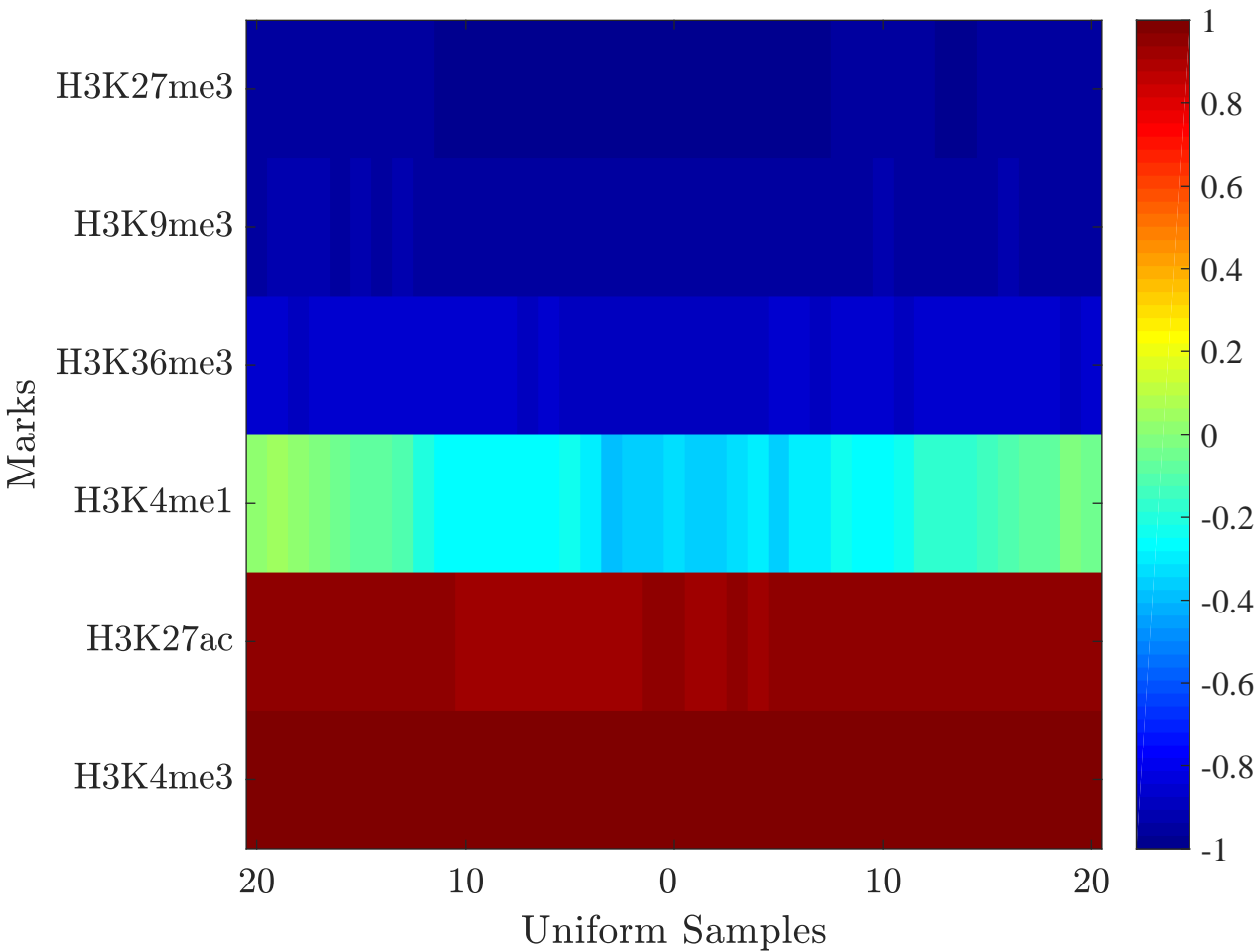

Supplement: Supplementary file 3 — HebbPlots of high-CpG promoters. This compressed file (.tar.gz) includes HebbPlots of high-CpG promoters active in 57 tissues/cell types. (TAR 2654 kb) [file 12859_2018_2312_MOESM3_ESM.tar › file4/E094.pdf]

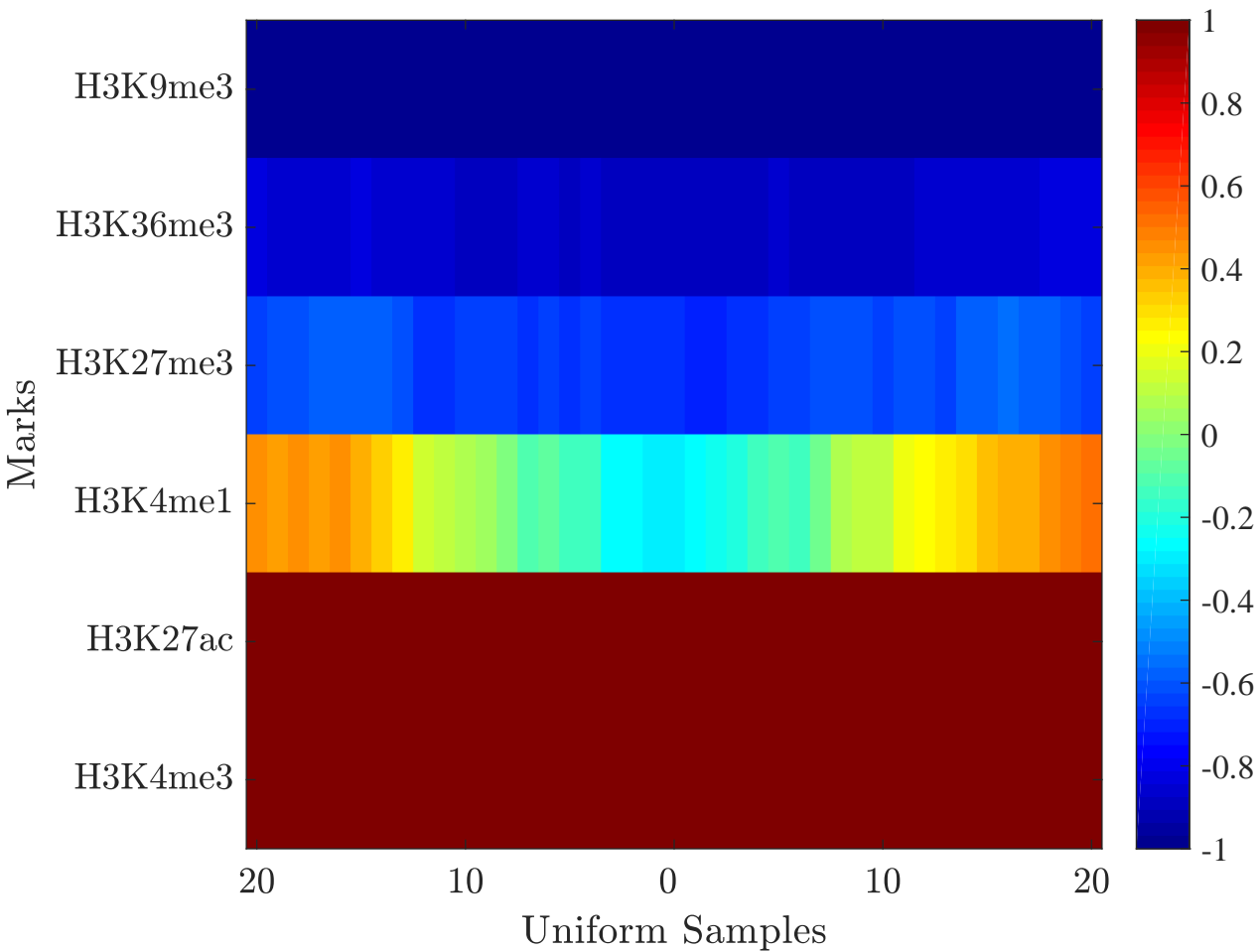

Supplement: Supplementary file 3 — HebbPlots of high-CpG promoters. This compressed file (.tar.gz) includes HebbPlots of high-CpG promoters active in 57 tissues/cell types. (TAR 2654 kb) [file 12859_2018_2312_MOESM3_ESM.tar › file4/E095.pdf]

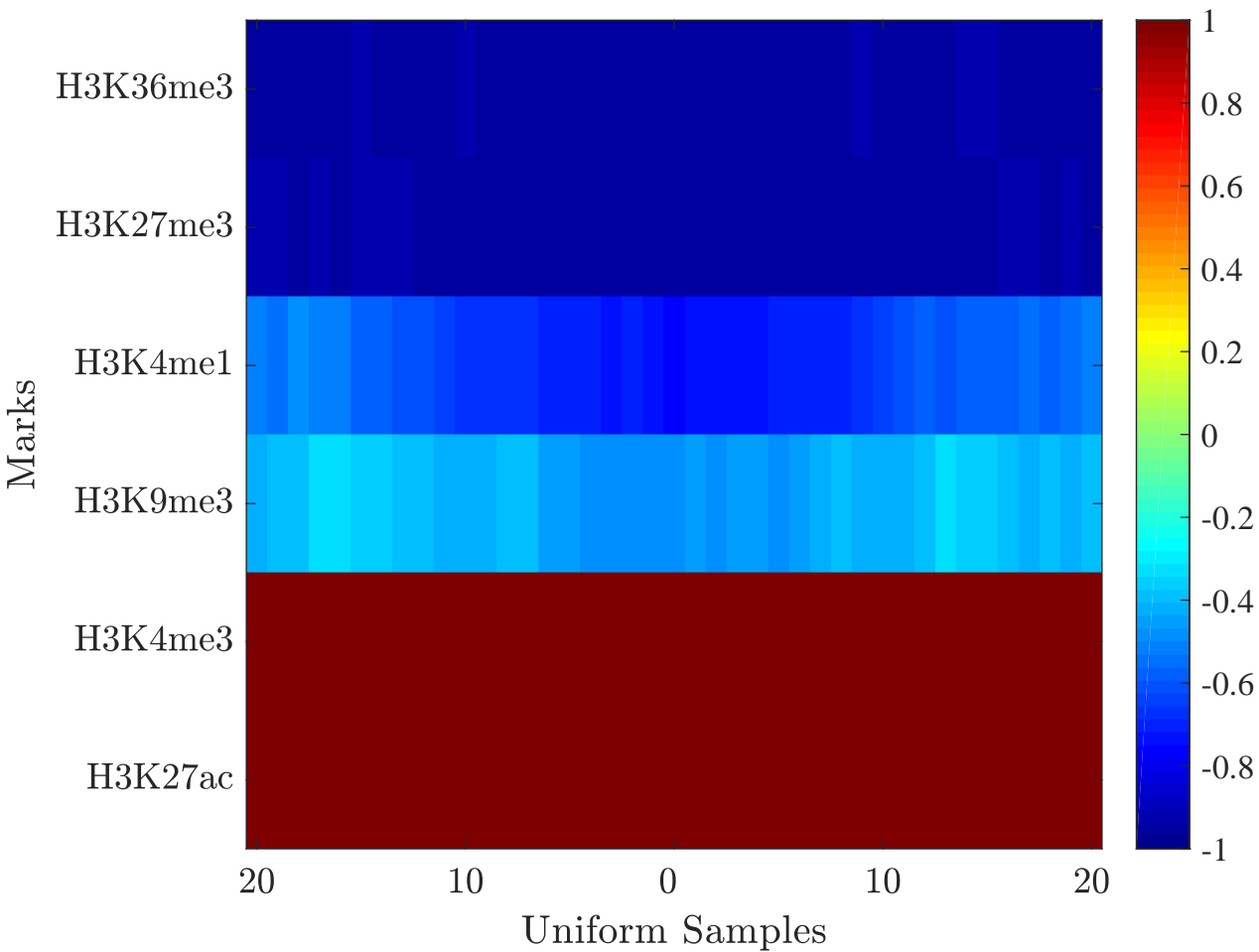

Supplement: Supplementary file 3 — HebbPlots of high-CpG promoters. This compressed file (.tar.gz) includes HebbPlots of high-CpG promoters active in 57 tissues/cell types. (TAR 2654 kb) [file 12859_2018_2312_MOESM3_ESM.tar › file4/E096.pdf]

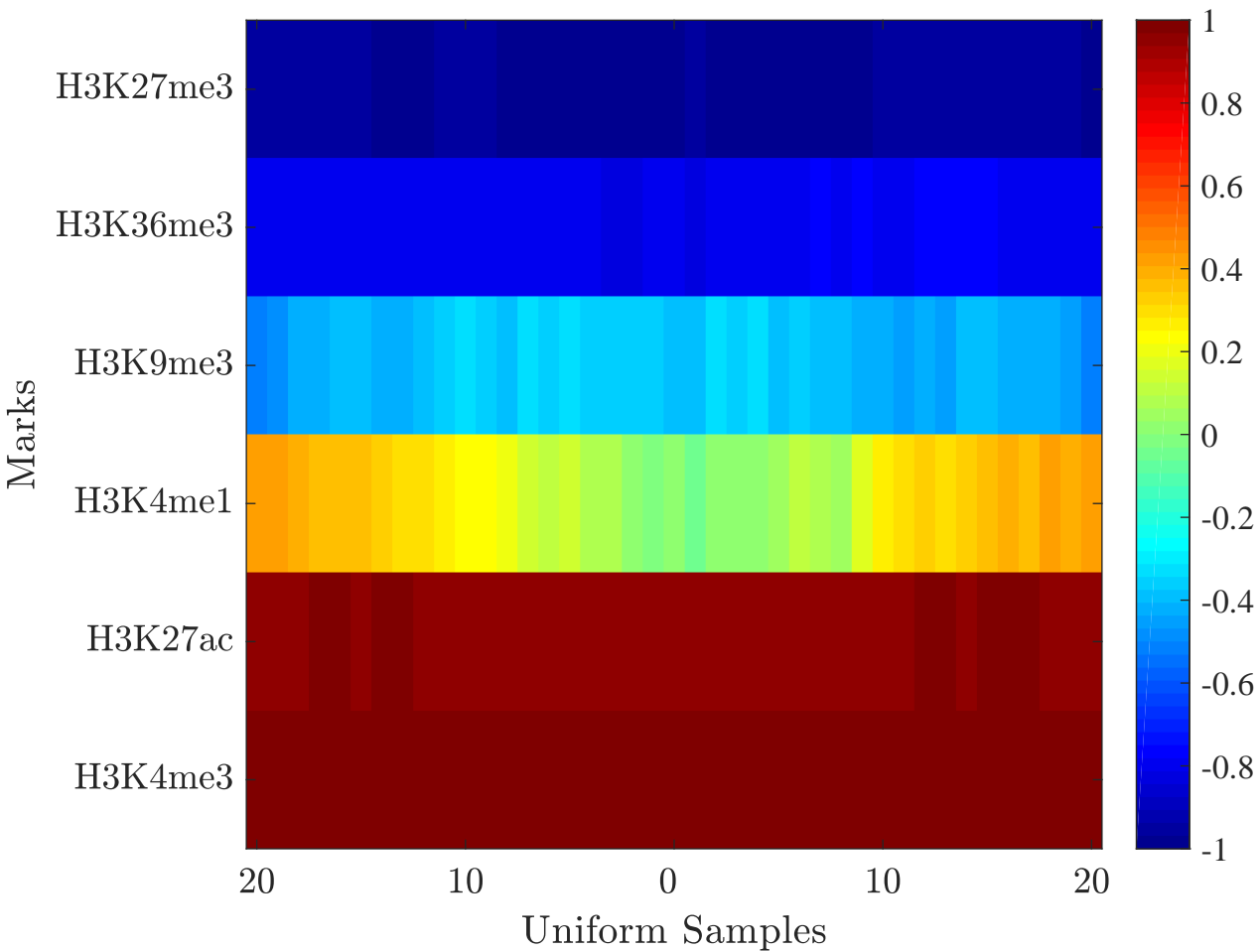

Supplement: Supplementary file 3 — HebbPlots of high-CpG promoters. This compressed file (.tar.gz) includes HebbPlots of high-CpG promoters active in 57 tissues/cell types. (TAR 2654 kb) [file 12859_2018_2312_MOESM3_ESM.tar › file4/E097.pdf]

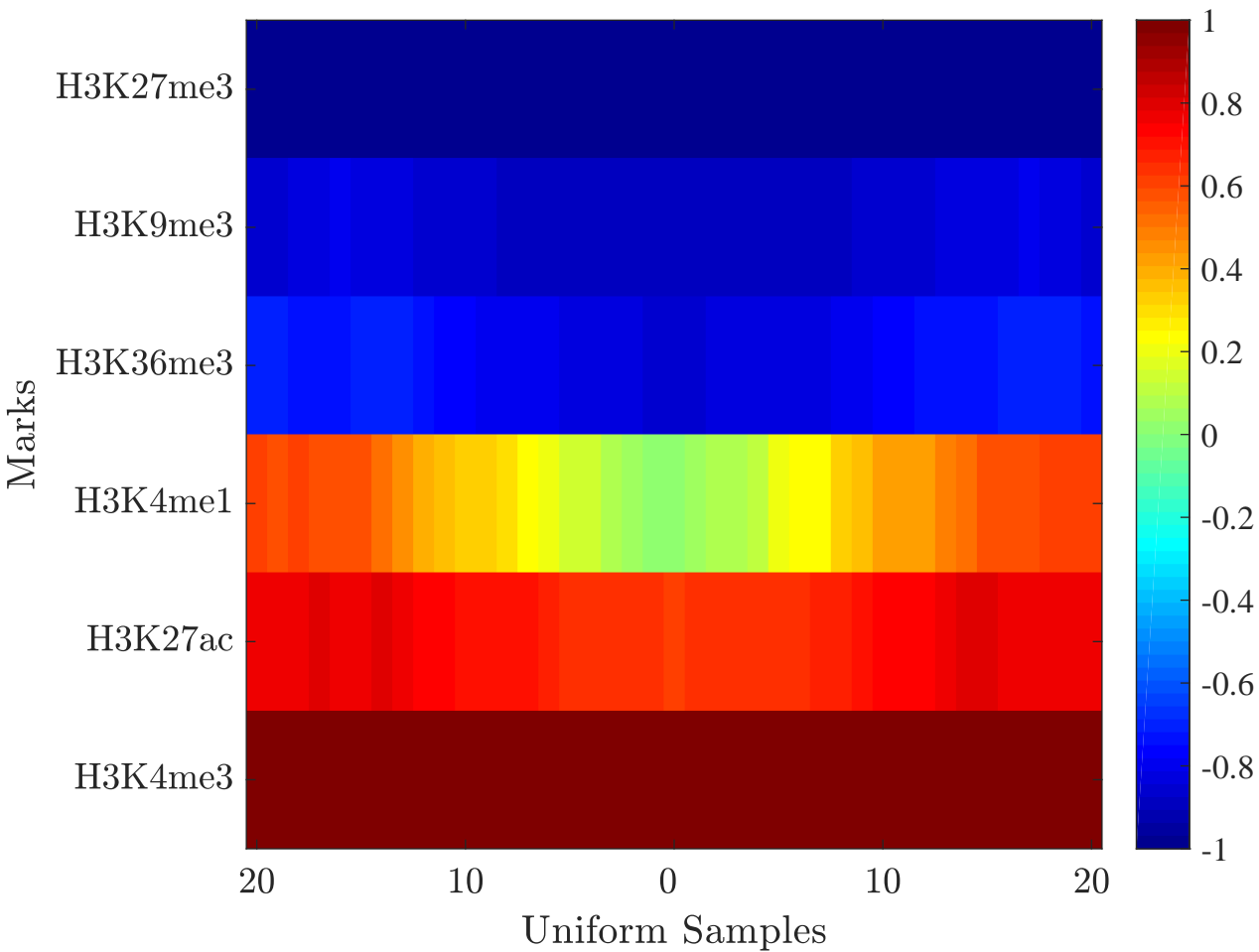

Supplement: Supplementary file 3 — HebbPlots of high-CpG promoters. This compressed file (.tar.gz) includes HebbPlots of high-CpG promoters active in 57 tissues/cell types. (TAR 2654 kb) [file 12859_2018_2312_MOESM3_ESM.tar › file4/E098.pdf]

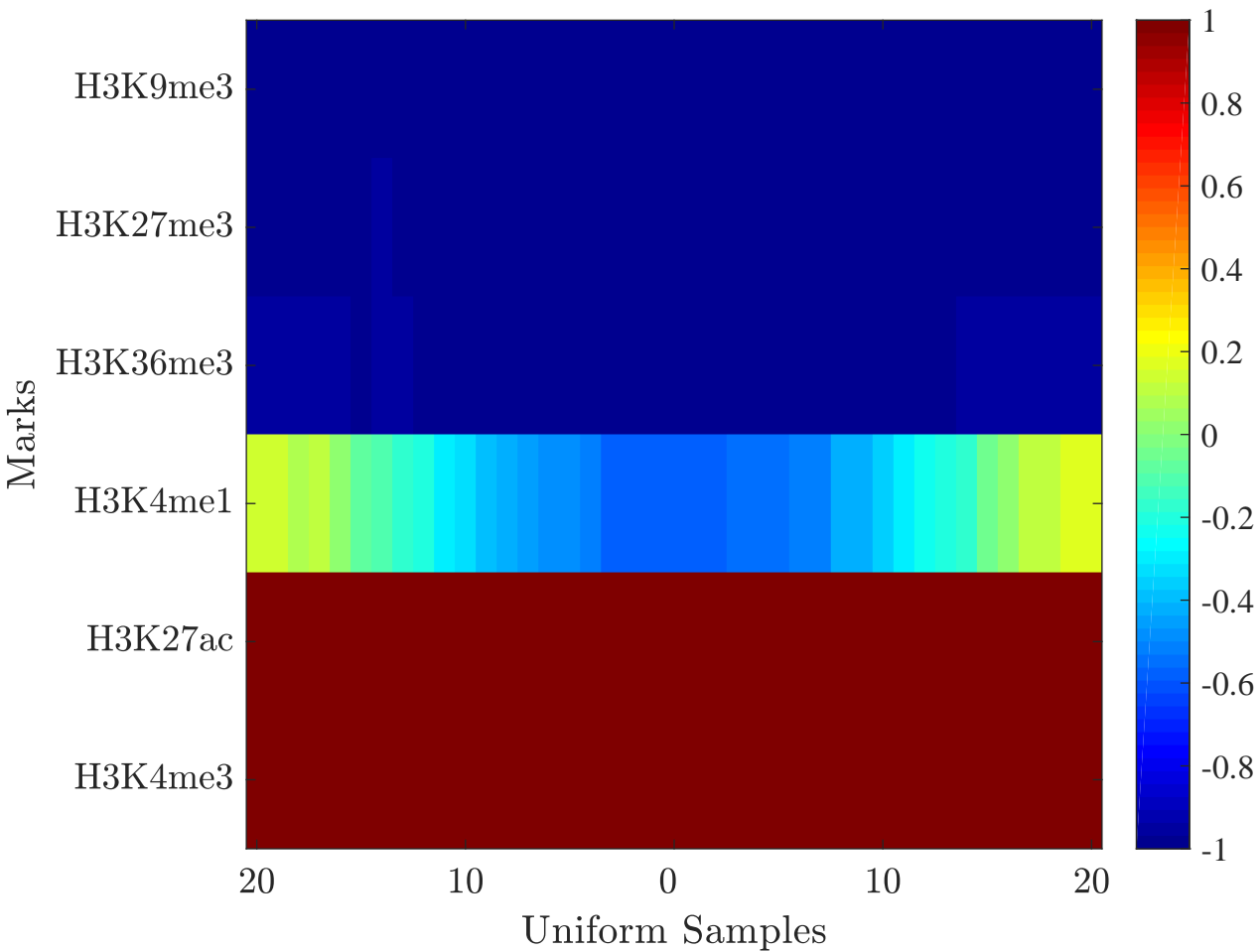

Supplement: Supplementary file 3 — HebbPlots of high-CpG promoters. This compressed file (.tar.gz) includes HebbPlots of high-CpG promoters active in 57 tissues/cell types. (TAR 2654 kb) [file 12859_2018_2312_MOESM3_ESM.tar › file4/E100.pdf]

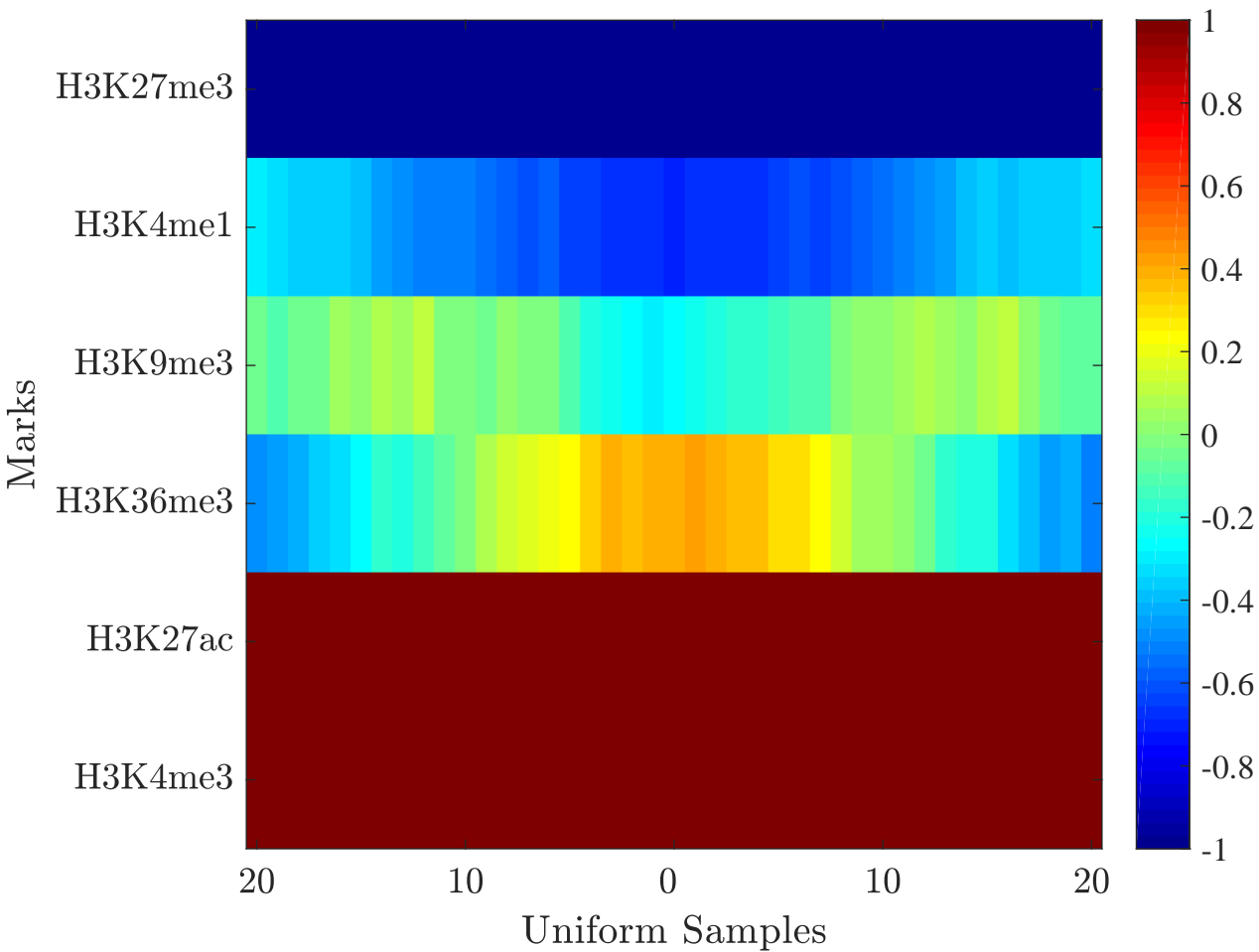

Supplement: Supplementary file 3 — HebbPlots of high-CpG promoters. This compressed file (.tar.gz) includes HebbPlots of high-CpG promoters active in 57 tissues/cell types. (TAR 2654 kb) [file 12859_2018_2312_MOESM3_ESM.tar › file4/E104.pdf]

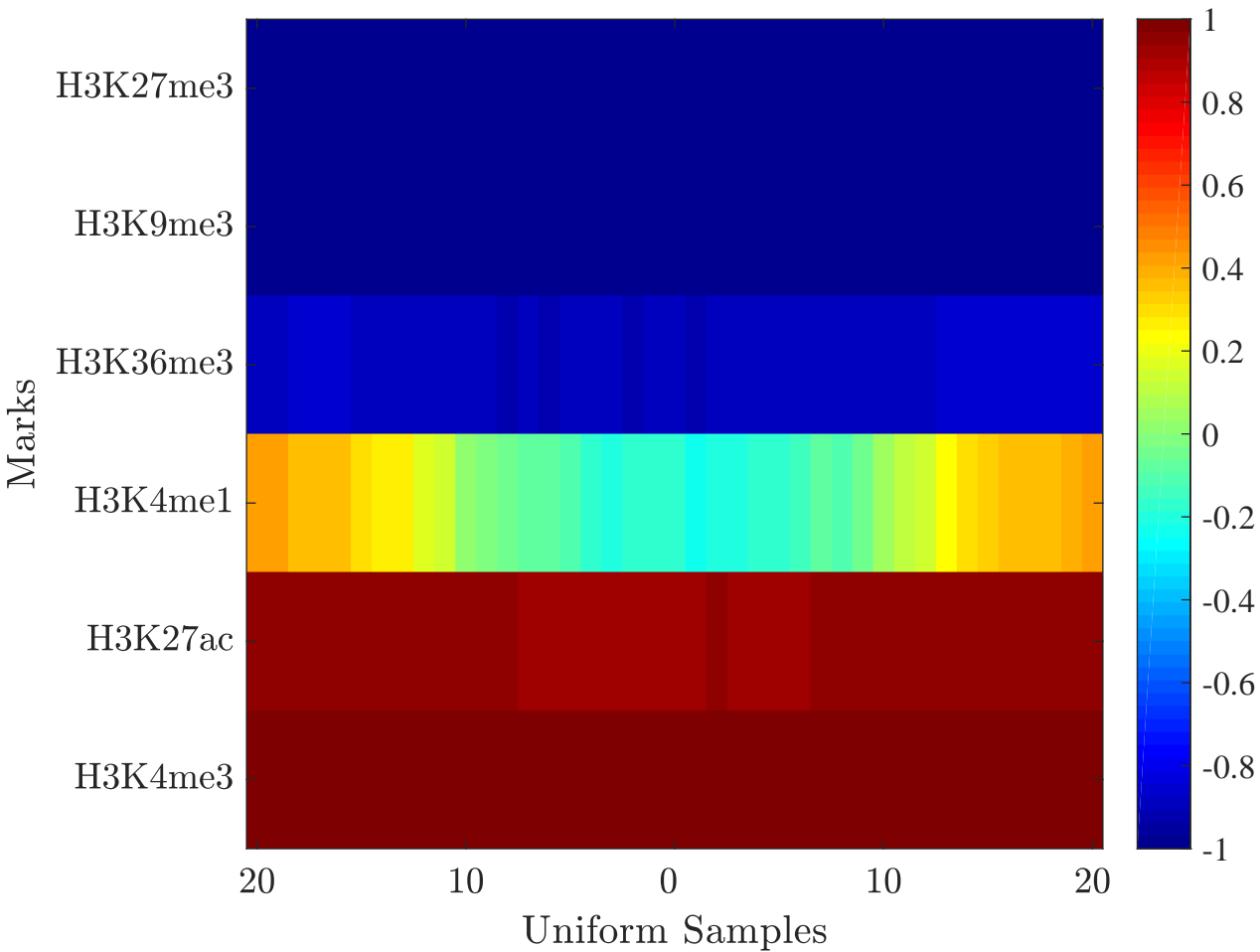

Supplement: Supplementary file 3 — HebbPlots of high-CpG promoters. This compressed file (.tar.gz) includes HebbPlots of high-CpG promoters active in 57 tissues/cell types. (TAR 2654 kb) [file 12859_2018_2312_MOESM3_ESM.tar › file4/E105.pdf]

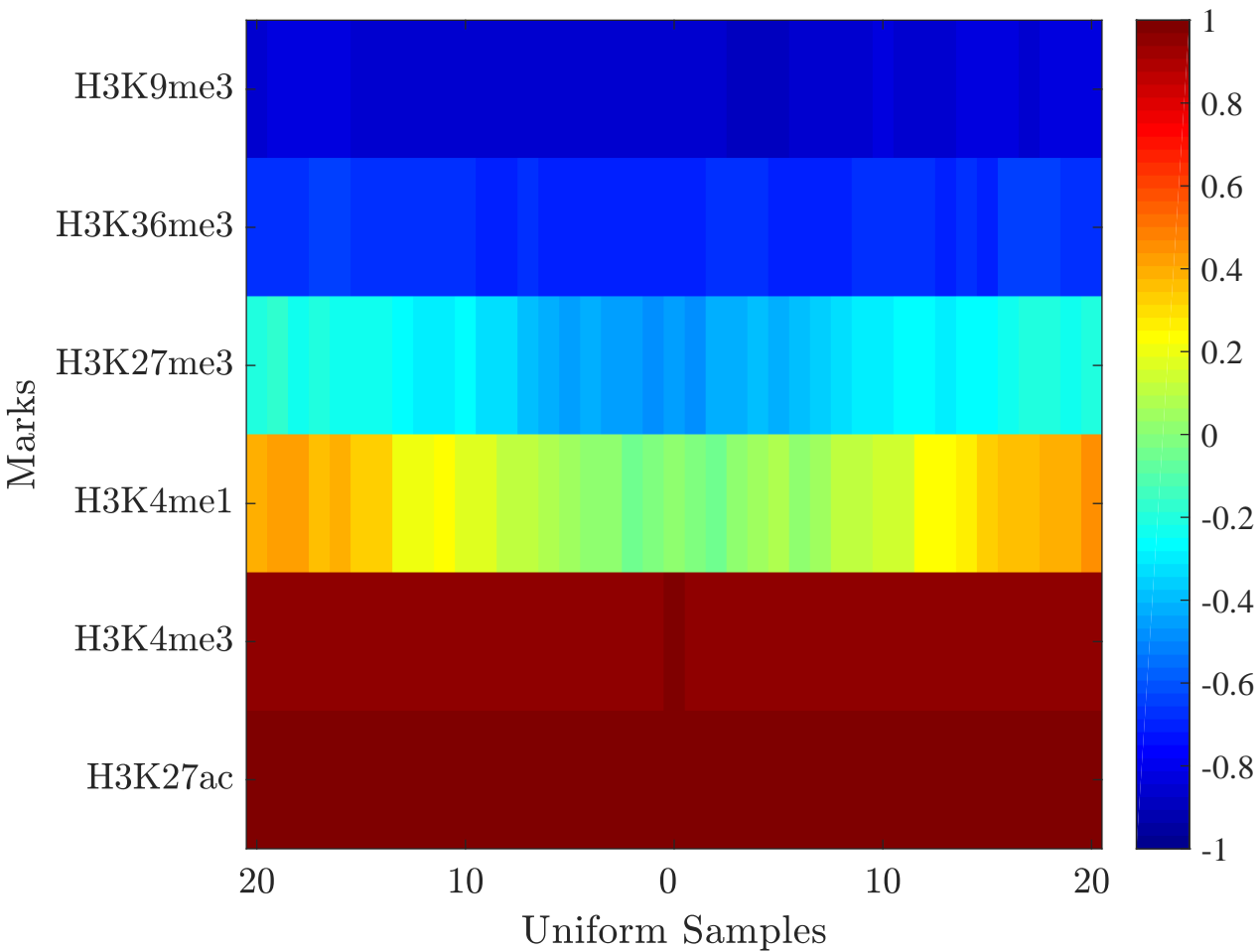

Supplement: Supplementary file 3 — HebbPlots of high-CpG promoters. This compressed file (.tar.gz) includes HebbPlots of high-CpG promoters active in 57 tissues/cell types. (TAR 2654 kb) [file 12859_2018_2312_MOESM3_ESM.tar › file4/E106.pdf]

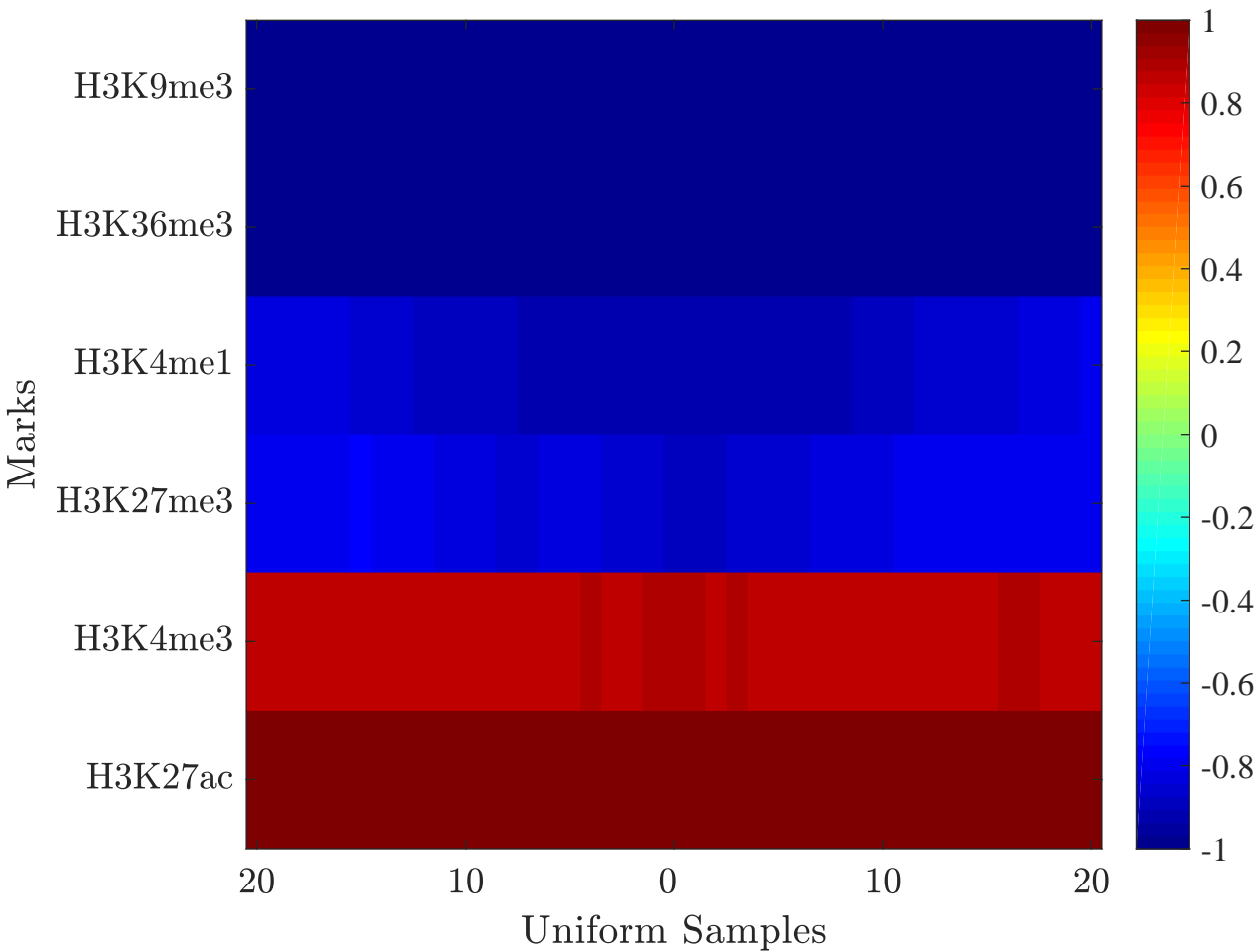

Supplement: Supplementary file 3 — HebbPlots of high-CpG promoters. This compressed file (.tar.gz) includes HebbPlots of high-CpG promoters active in 57 tissues/cell types. (TAR 2654 kb) [file 12859_2018_2312_MOESM3_ESM.tar › file4/E109.pdf]

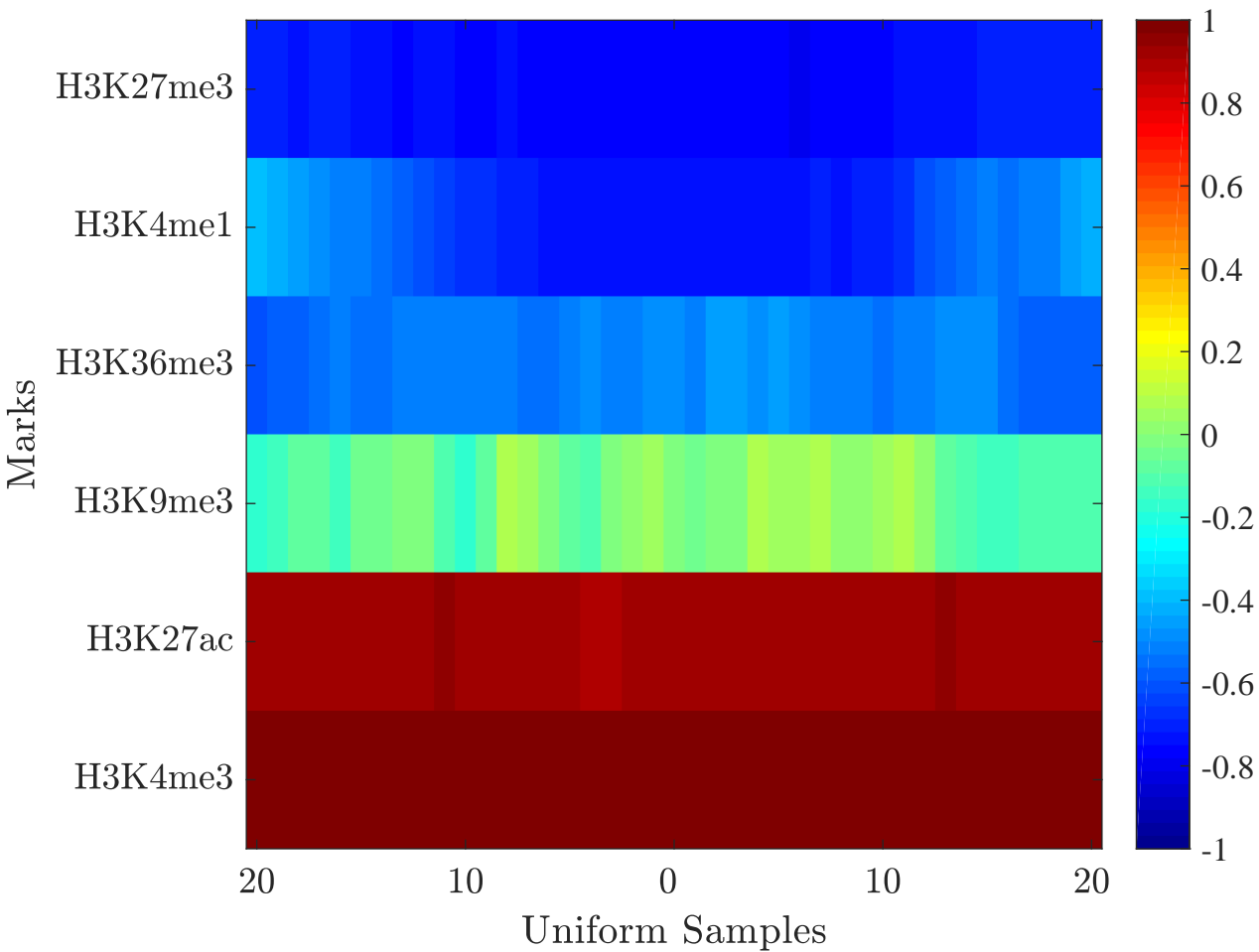

Supplement: Supplementary file 3 — HebbPlots of high-CpG promoters. This compressed file (.tar.gz) includes HebbPlots of high-CpG promoters active in 57 tissues/cell types. (TAR 2654 kb) [file 12859_2018_2312_MOESM3_ESM.tar › file4/E112.pdf]

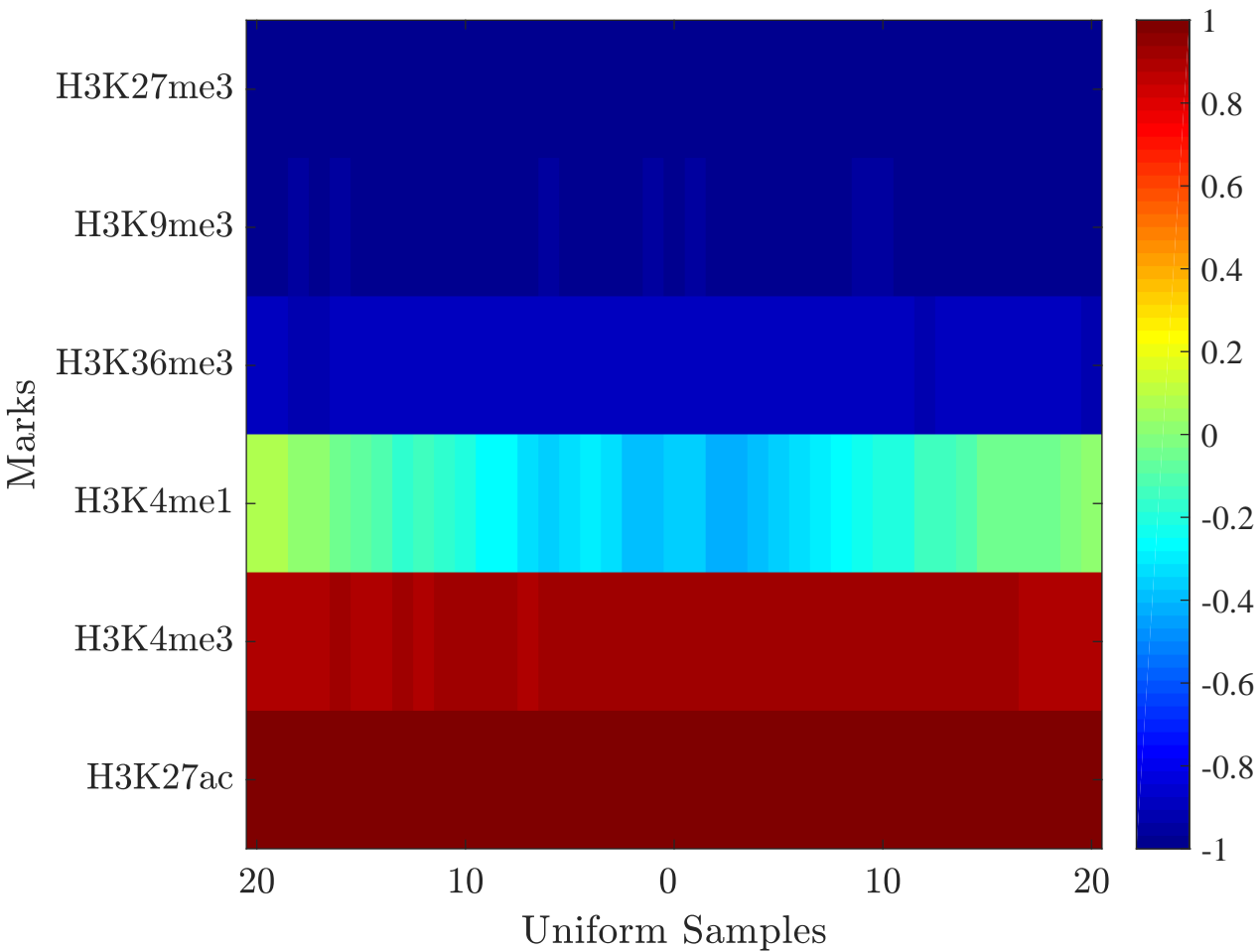

Supplement: Supplementary file 3 — HebbPlots of high-CpG promoters. This compressed file (.tar.gz) includes HebbPlots of high-CpG promoters active in 57 tissues/cell types. (TAR 2654 kb) [file 12859_2018_2312_MOESM3_ESM.tar › file4/E113.pdf]

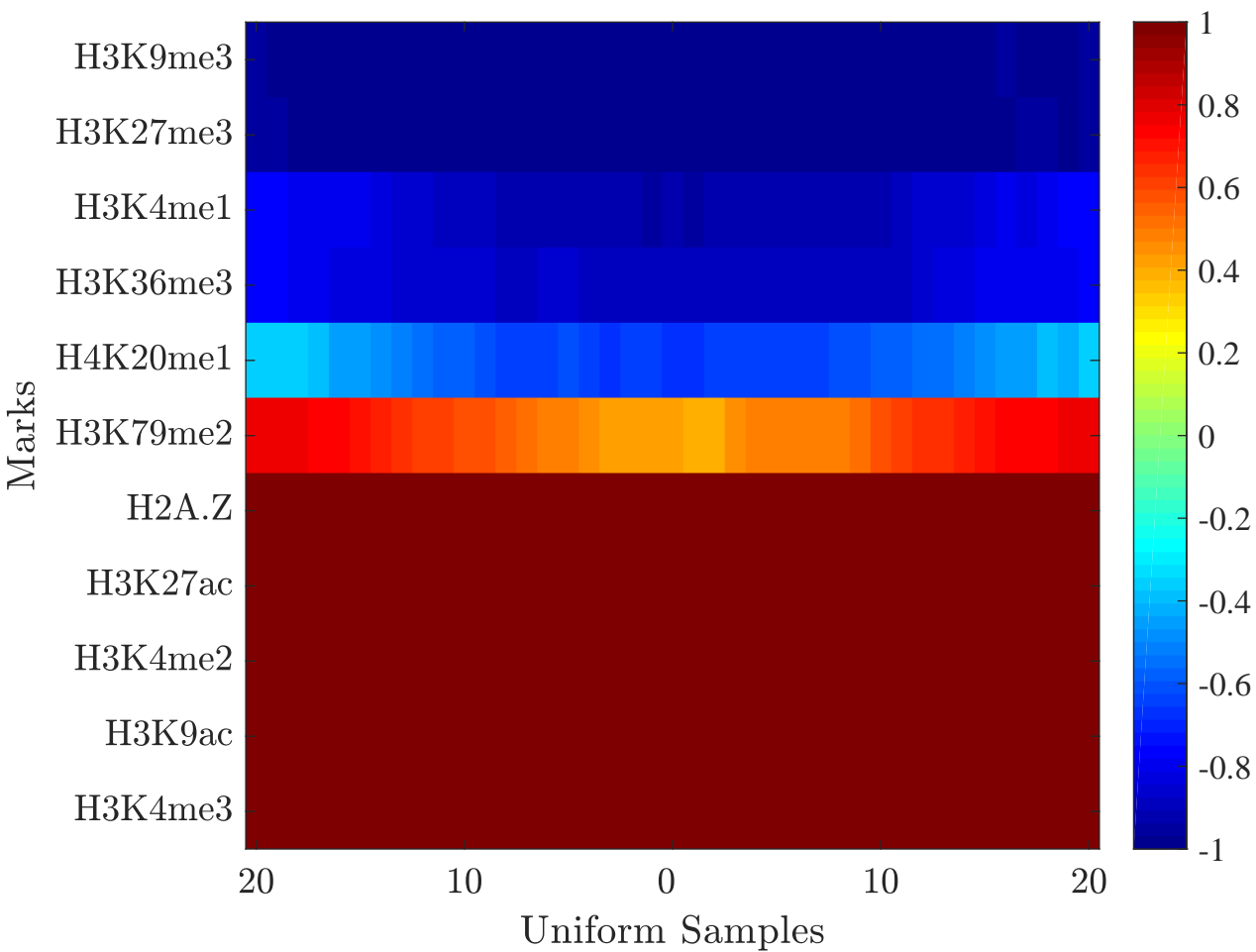

Supplement: Supplementary file 3 — HebbPlots of high-CpG promoters. This compressed file (.tar.gz) includes HebbPlots of high-CpG promoters active in 57 tissues/cell types. (TAR 2654 kb) [file 12859_2018_2312_MOESM3_ESM.tar › file4/E114.pdf]

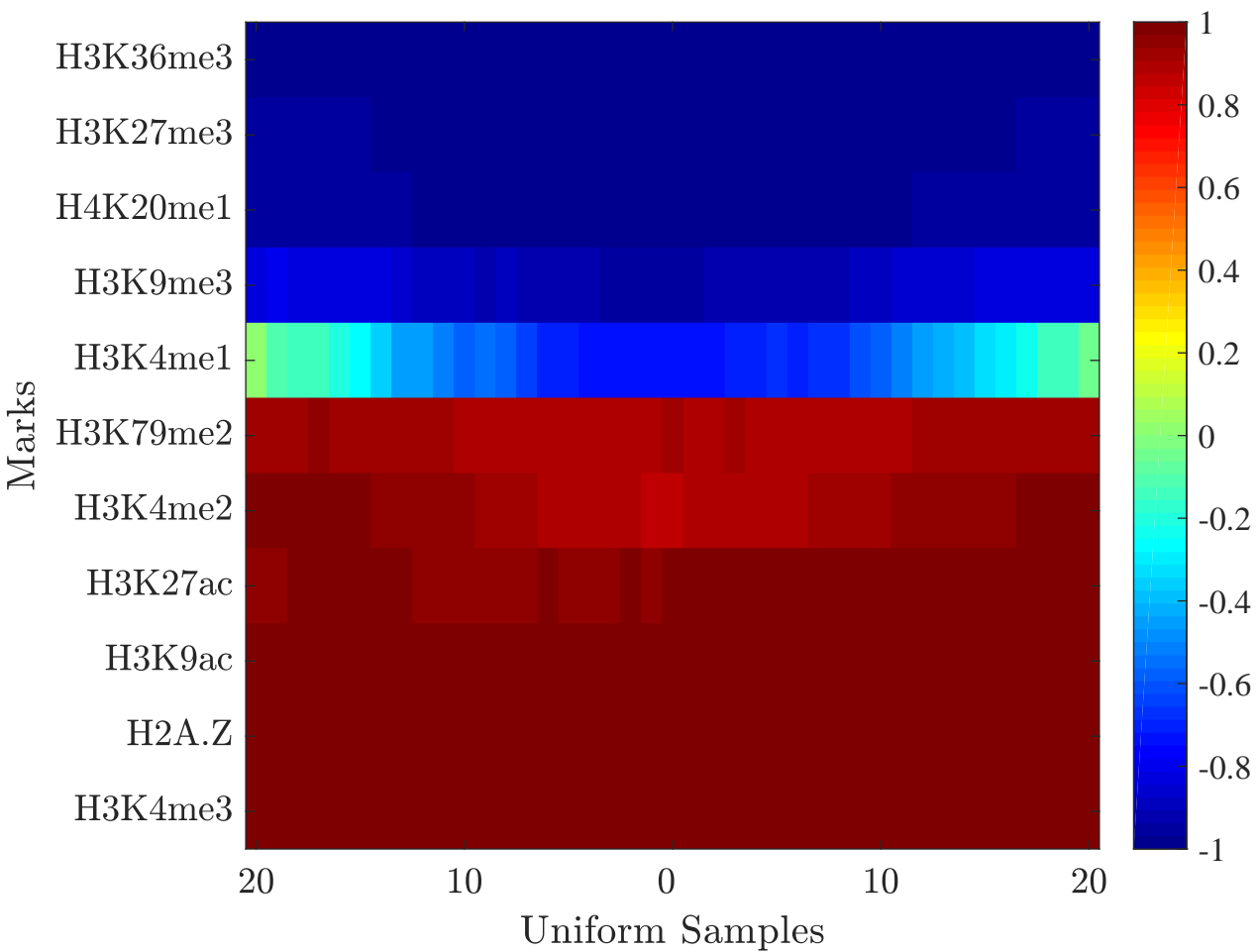

Supplement: Supplementary file 3 — HebbPlots of high-CpG promoters. This compressed file (.tar.gz) includes HebbPlots of high-CpG promoters active in 57 tissues/cell types. (TAR 2654 kb) [file 12859_2018_2312_MOESM3_ESM.tar › file4/E116.pdf]

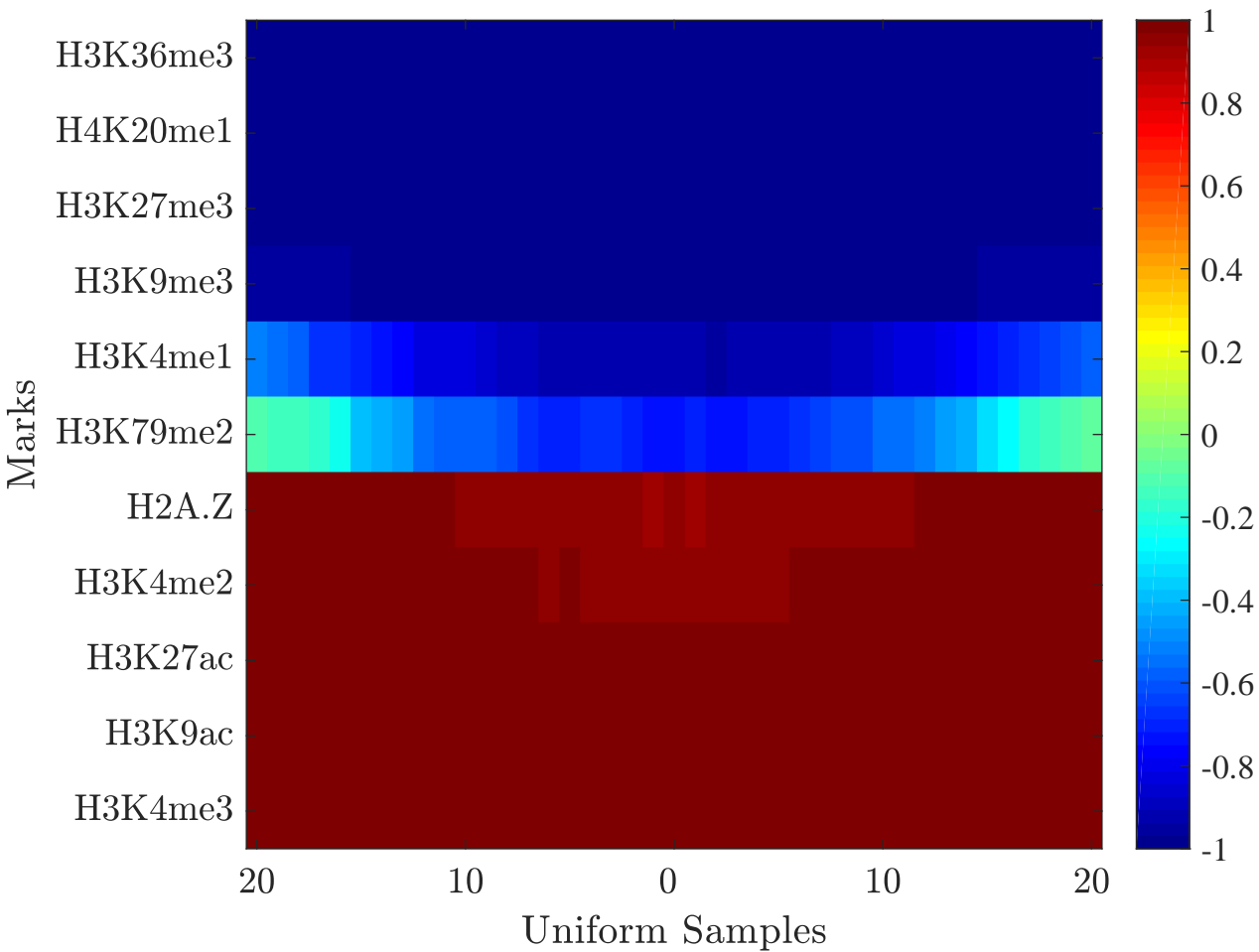

Supplement: Supplementary file 3 — HebbPlots of high-CpG promoters. This compressed file (.tar.gz) includes HebbPlots of high-CpG promoters active in 57 tissues/cell types. (TAR 2654 kb) [file 12859_2018_2312_MOESM3_ESM.tar › file4/E117.pdf]

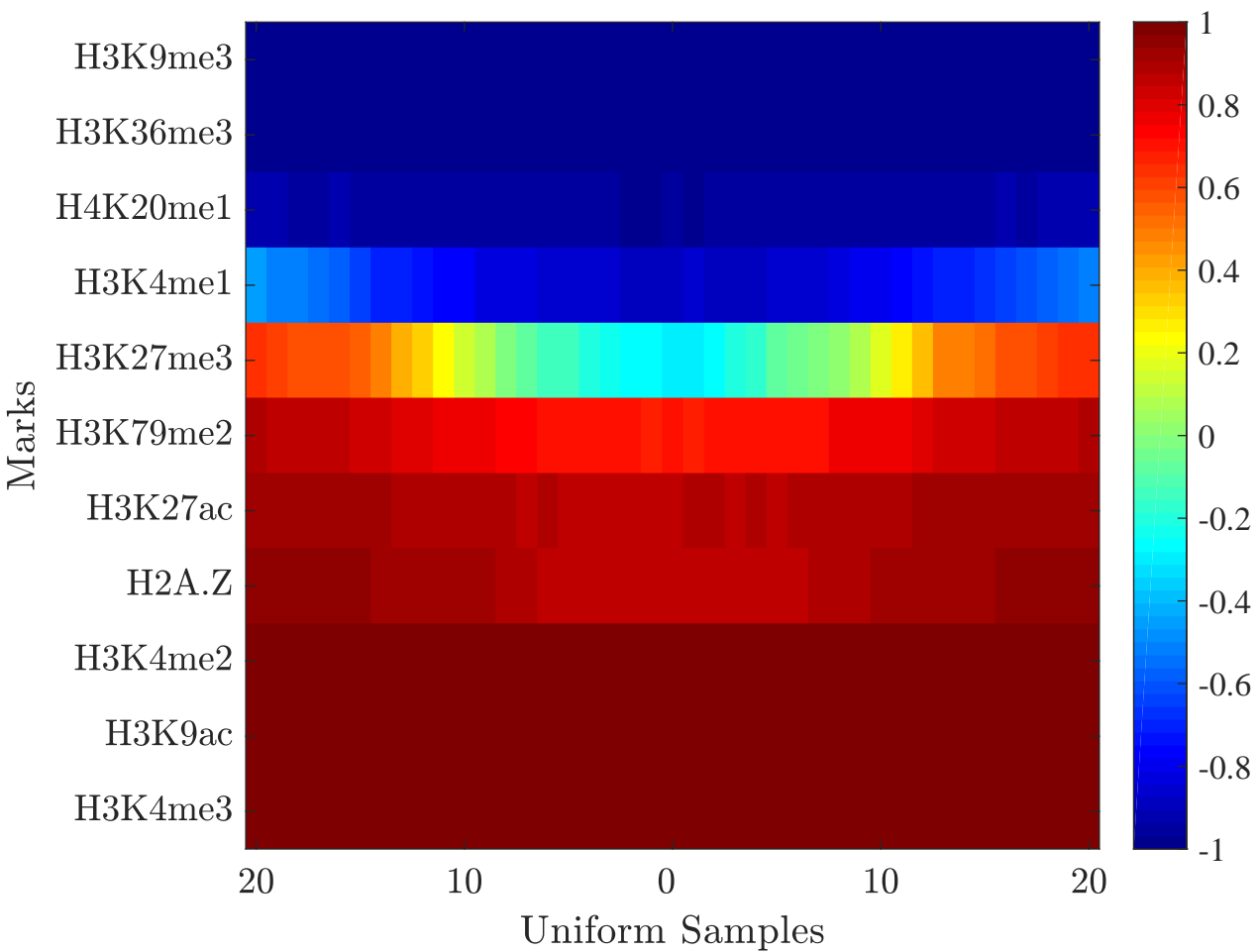

Supplement: Supplementary file 3 — HebbPlots of high-CpG promoters. This compressed file (.tar.gz) includes HebbPlots of high-CpG promoters active in 57 tissues/cell types. (TAR 2654 kb) [file 12859_2018_2312_MOESM3_ESM.tar › file4/E118.pdf]

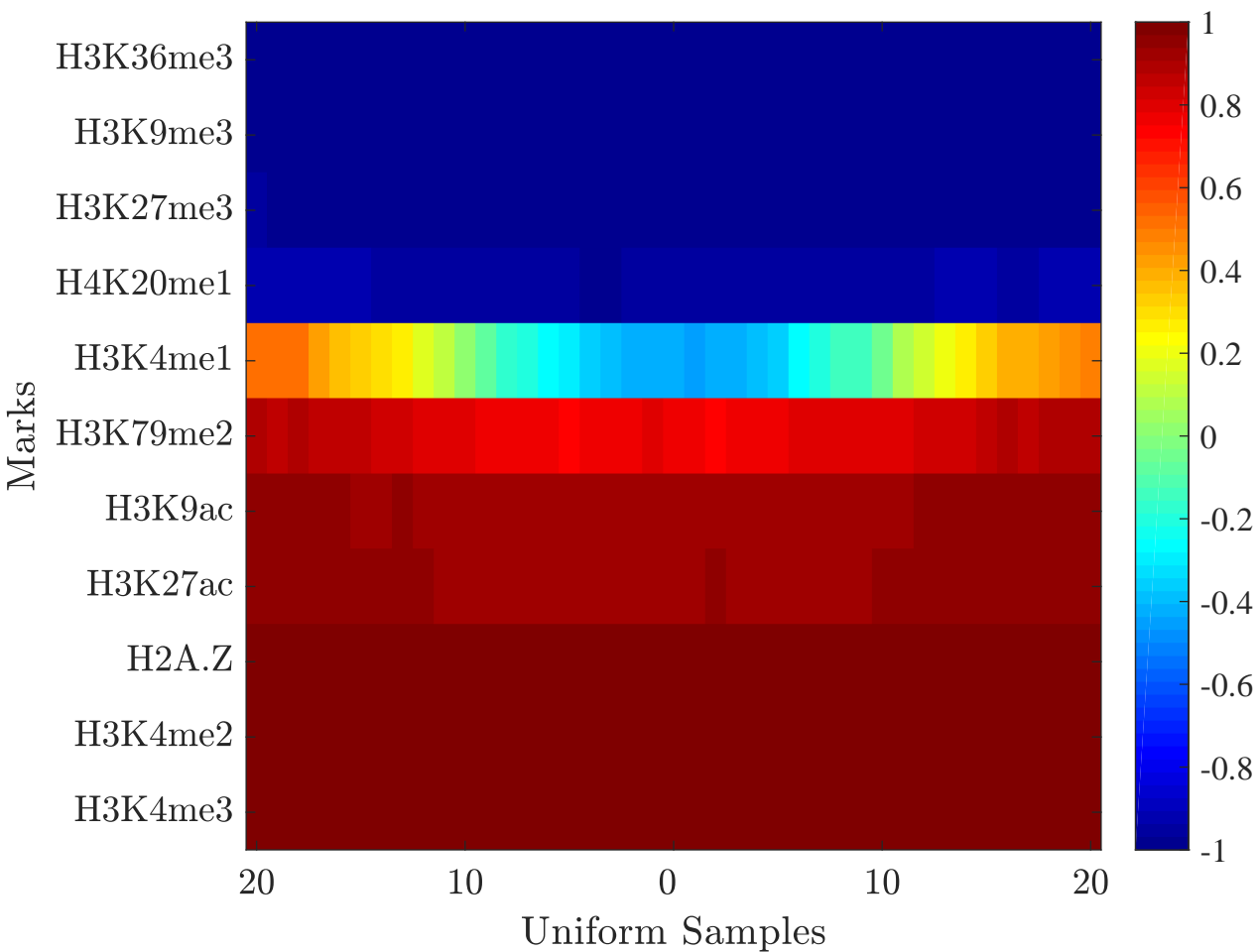

Supplement: Supplementary file 3 — HebbPlots of high-CpG promoters. This compressed file (.tar.gz) includes HebbPlots of high-CpG promoters active in 57 tissues/cell types. (TAR 2654 kb) [file 12859_2018_2312_MOESM3_ESM.tar › file4/E119.pdf]

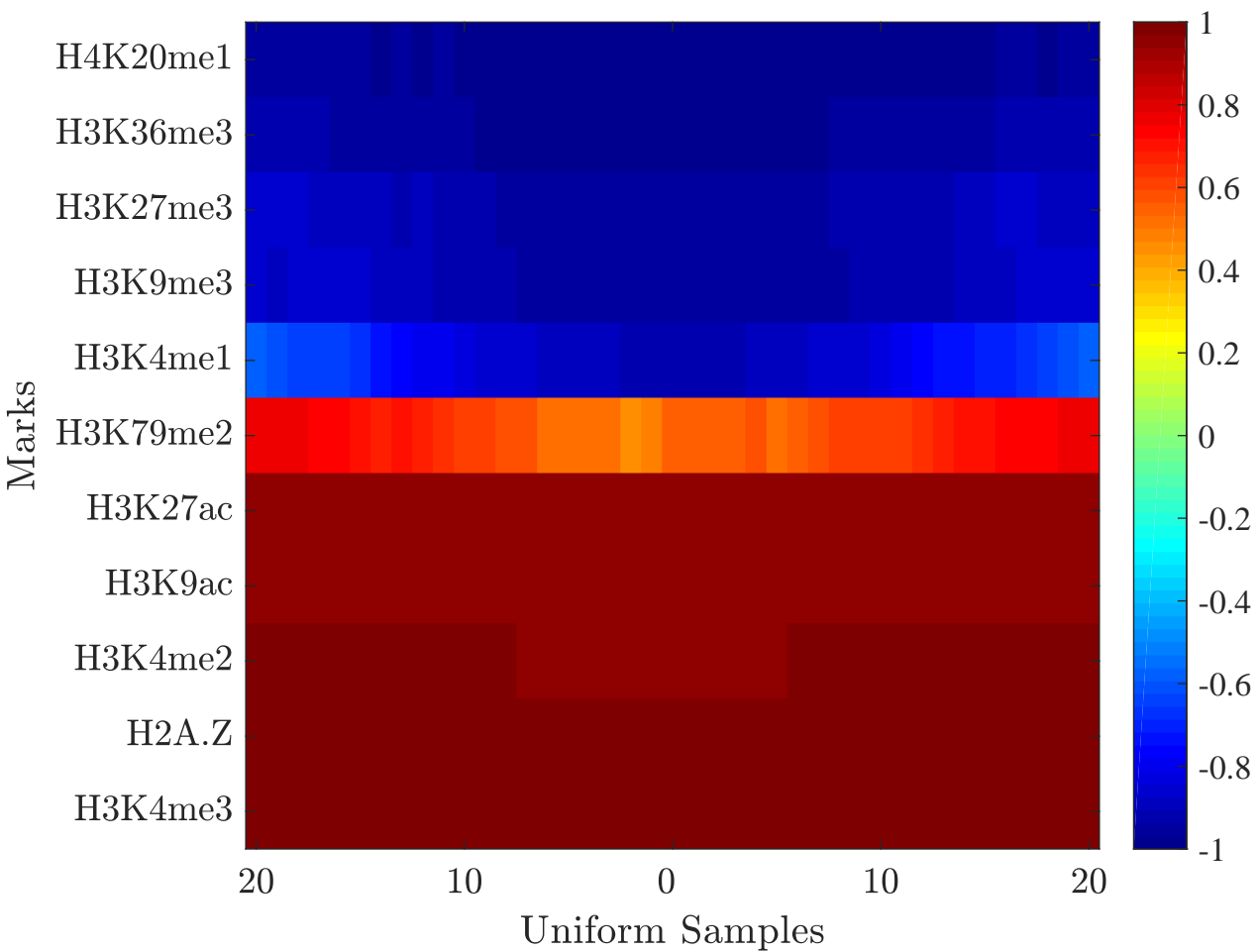

Supplement: Supplementary file 3 — HebbPlots of high-CpG promoters. This compressed file (.tar.gz) includes HebbPlots of high-CpG promoters active in 57 tissues/cell types. (TAR 2654 kb) [file 12859_2018_2312_MOESM3_ESM.tar › file4/E120.pdf]

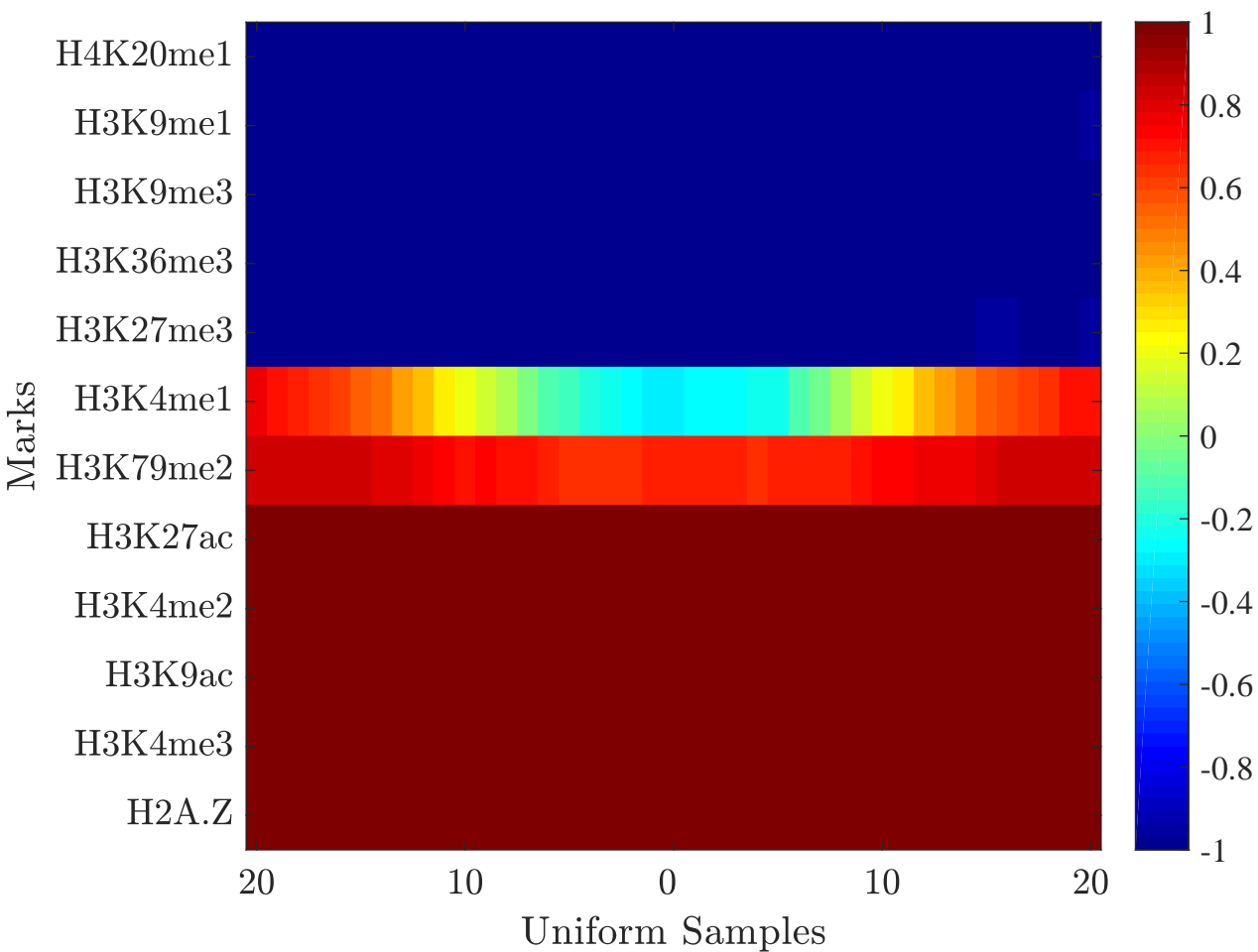

Supplement: Supplementary file 3 — HebbPlots of high-CpG promoters. This compressed file (.tar.gz) includes HebbPlots of high-CpG promoters active in 57 tissues/cell types. (TAR 2654 kb) [file 12859_2018_2312_MOESM3_ESM.tar › file4/E122.pdf]

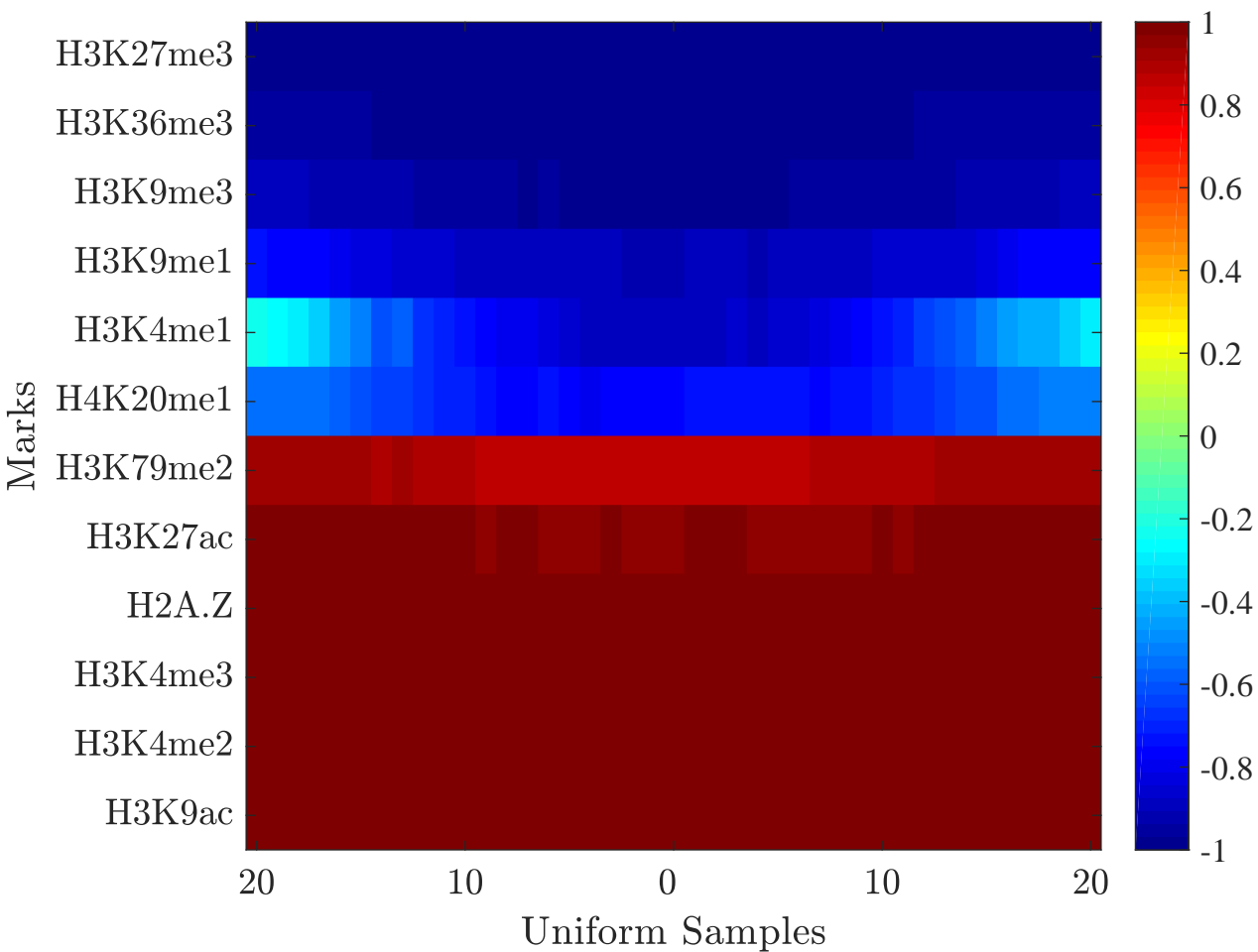

Supplement: Supplementary file 3 — HebbPlots of high-CpG promoters. This compressed file (.tar.gz) includes HebbPlots of high-CpG promoters active in 57 tissues/cell types. (TAR 2654 kb) [file 12859_2018_2312_MOESM3_ESM.tar › file4/E123.pdf]

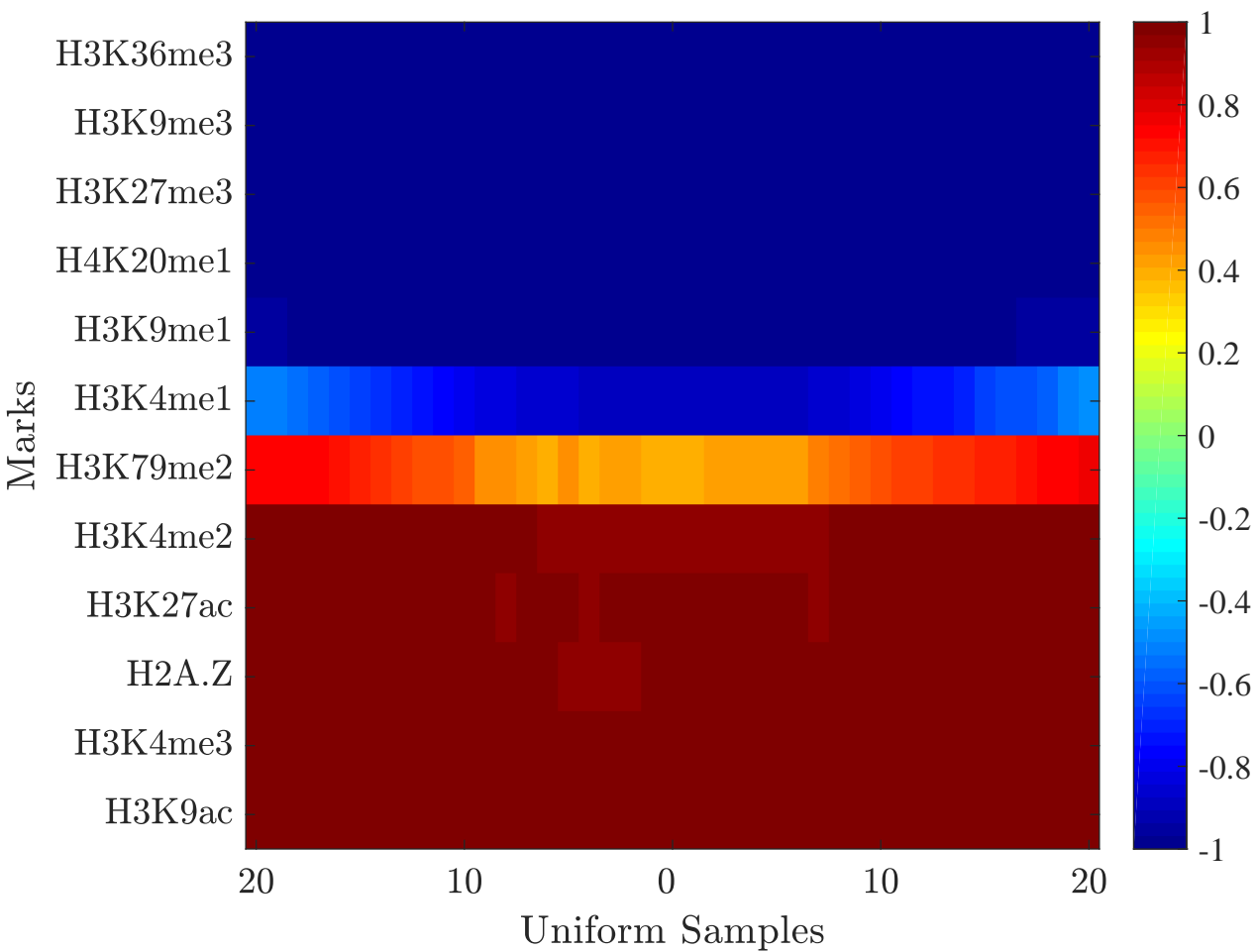

Supplement: Supplementary file 3 — HebbPlots of high-CpG promoters. This compressed file (.tar.gz) includes HebbPlots of high-CpG promoters active in 57 tissues/cell types. (TAR 2654 kb) [file 12859_2018_2312_MOESM3_ESM.tar › file4/E127.pdf]

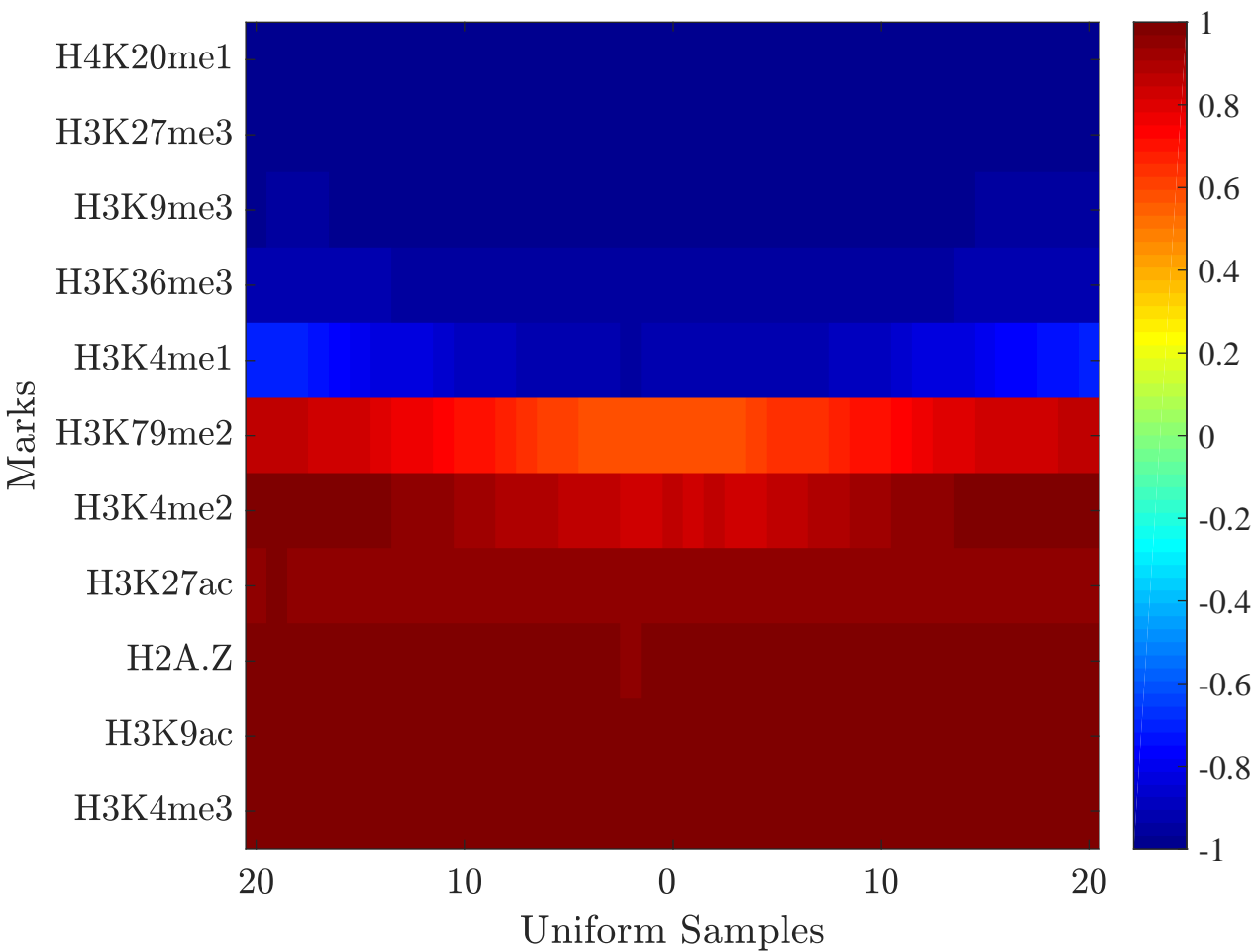

Supplement: Supplementary file 3 — HebbPlots of high-CpG promoters. This compressed file (.tar.gz) includes HebbPlots of high-CpG promoters active in 57 tissues/cell types. (TAR 2654 kb) [file 12859_2018_2312_MOESM3_ESM.tar › file4/E128.pdf]

Marks

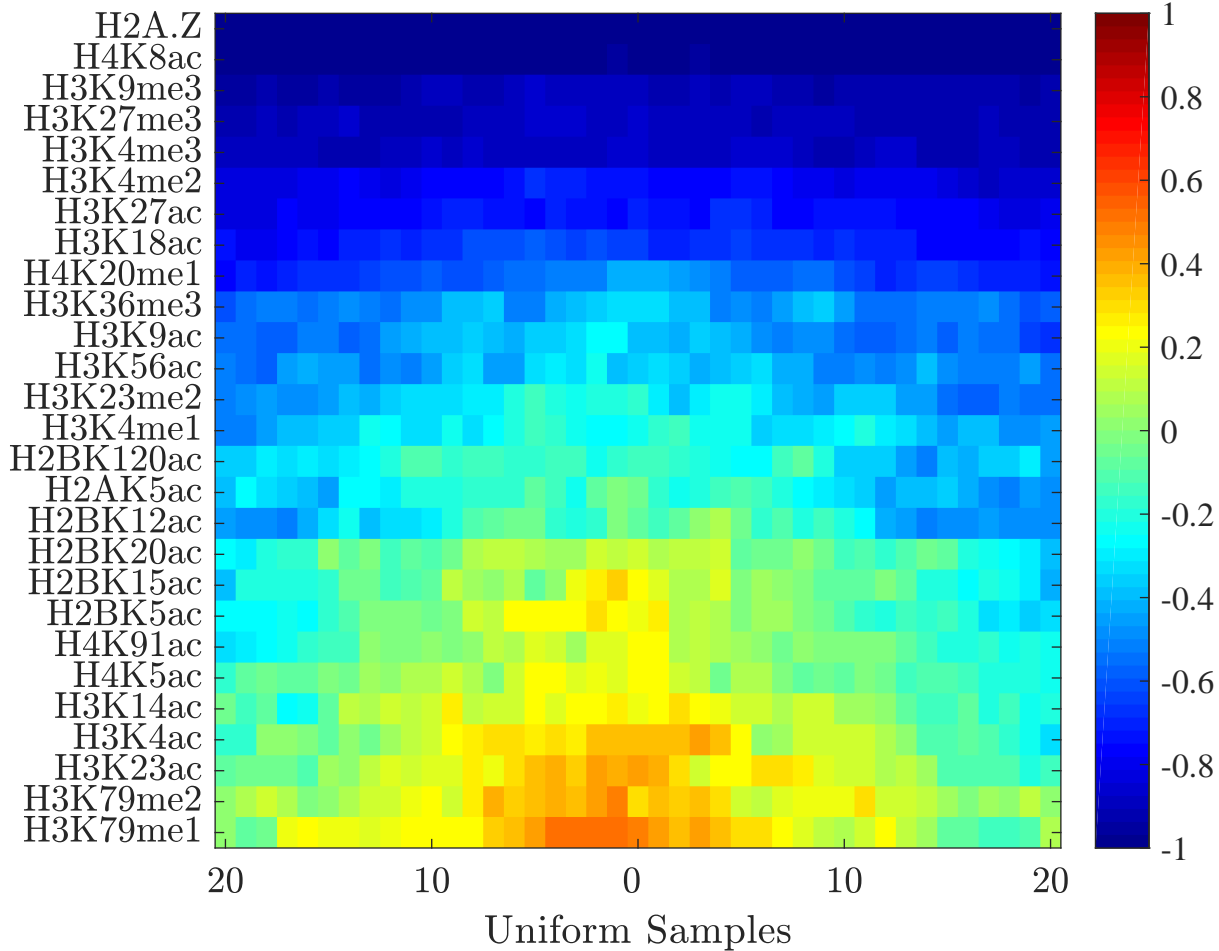

Supplement: Supplementary file 4 — HebbPlots of low-CpG promoters. This compressed file (.tar.gz) includes HebbPlots of low-CpG promoters active in 57 tissues/cell types. (TAR 2971 kb) [file 12859_2018_2312_MOESM4_ESM.tar › file5/E003.pdf]

Marks

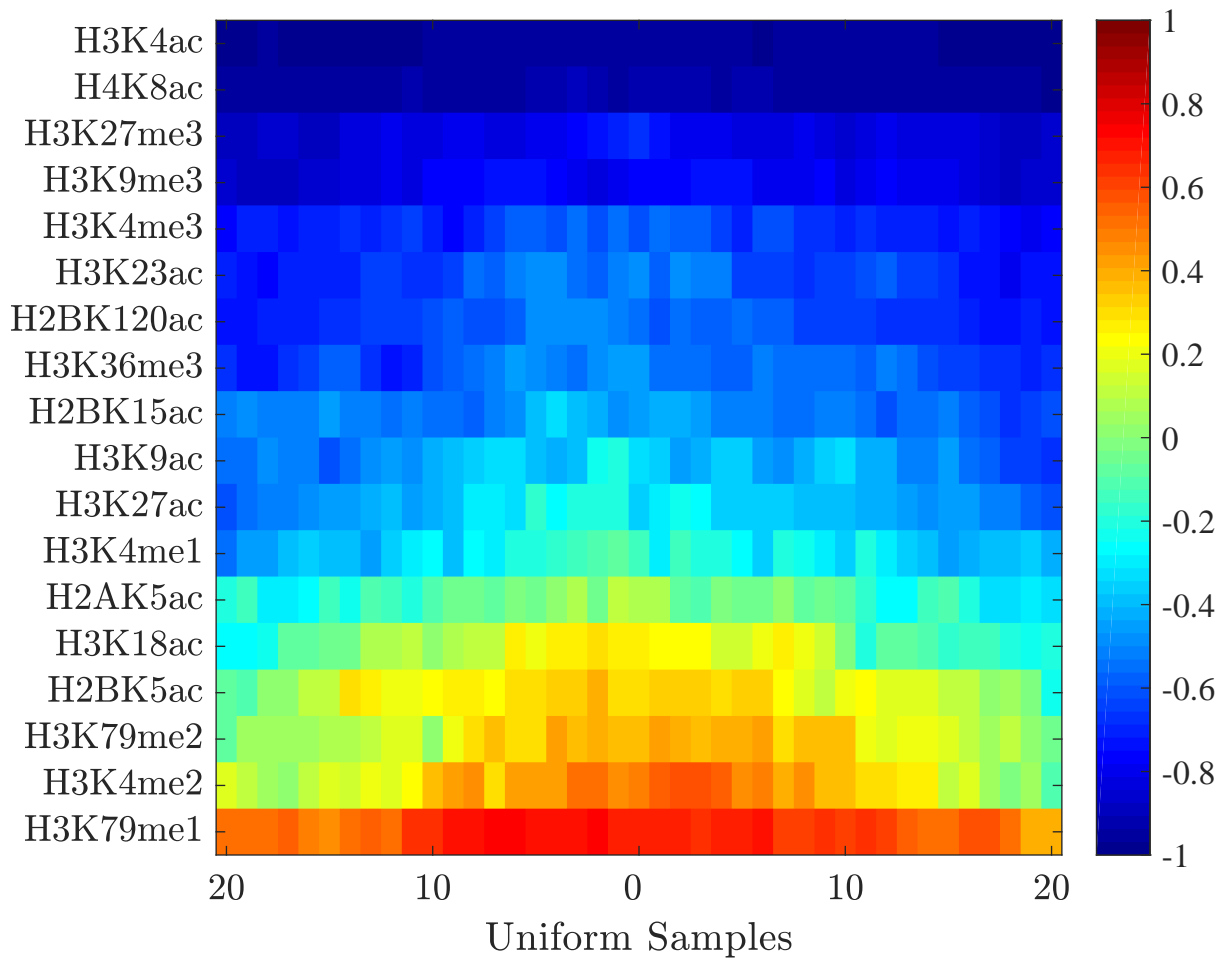

Supplement: Supplementary file 4 — HebbPlots of low-CpG promoters. This compressed file (.tar.gz) includes HebbPlots of low-CpG promoters active in 57 tissues/cell types. (TAR 2971 kb) [file 12859_2018_2312_MOESM4_ESM.tar › file5/E004.pdf]

Marks

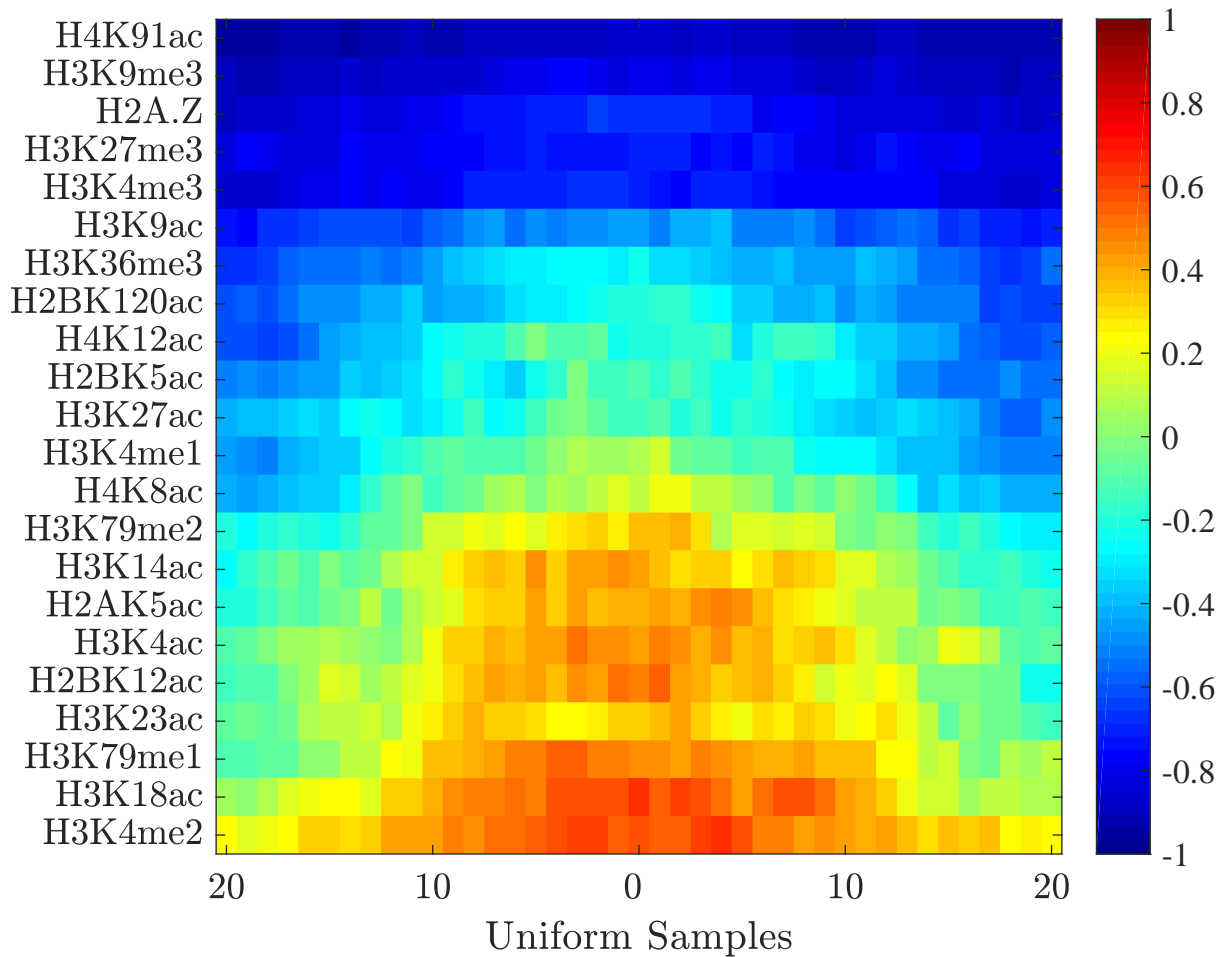

Supplement: Supplementary file 4 — HebbPlots of low-CpG promoters. This compressed file (.tar.gz) includes HebbPlots of low-CpG promoters active in 57 tissues/cell types. (TAR 2971 kb) [file 12859_2018_2312_MOESM4_ESM.tar › file5/E005.pdf]

Marks

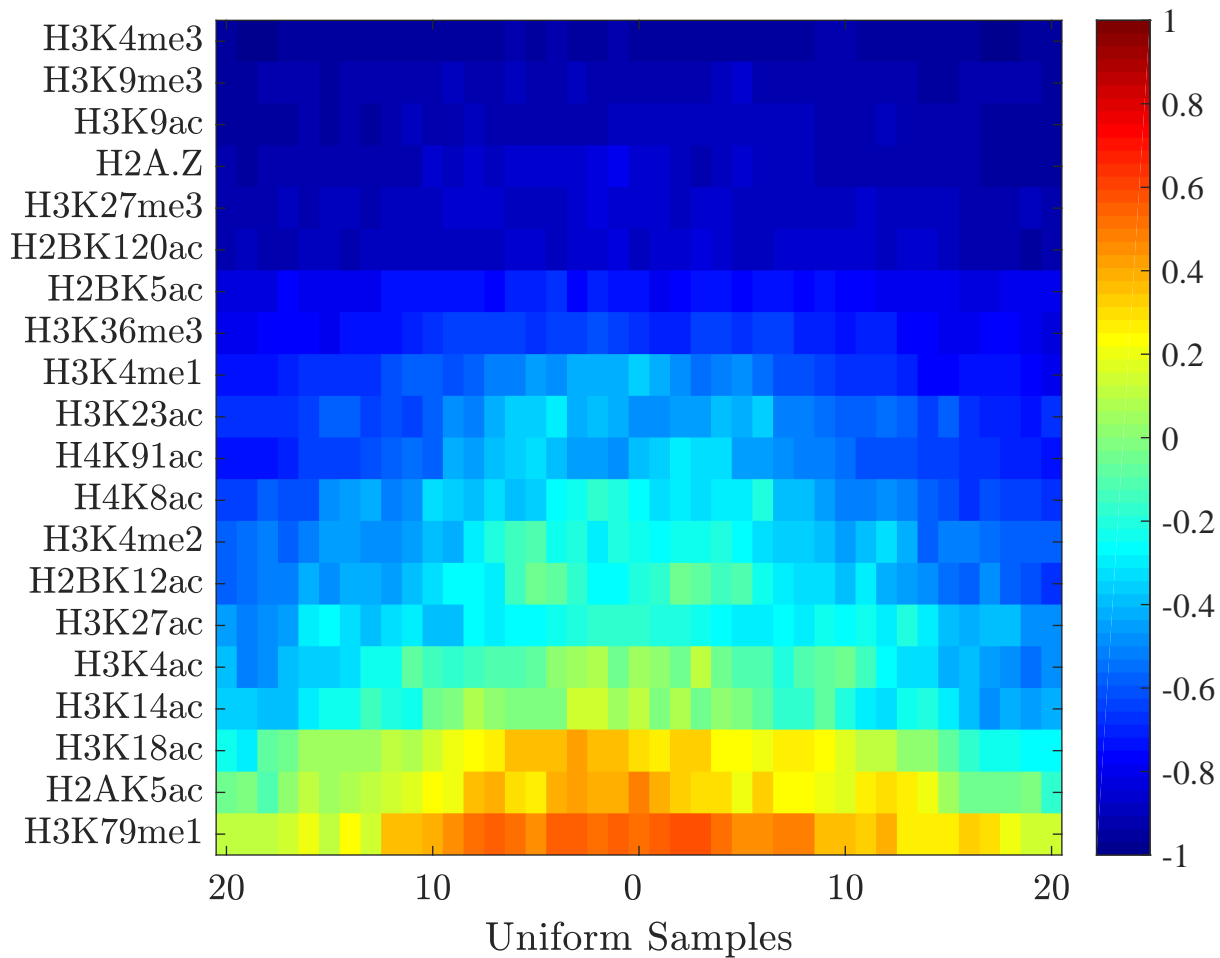

Supplement: Supplementary file 4 — HebbPlots of low-CpG promoters. This compressed file (.tar.gz) includes HebbPlots of low-CpG promoters active in 57 tissues/cell types. (TAR 2971 kb) [file 12859_2018_2312_MOESM4_ESM.tar › file5/E006.pdf]

Marks

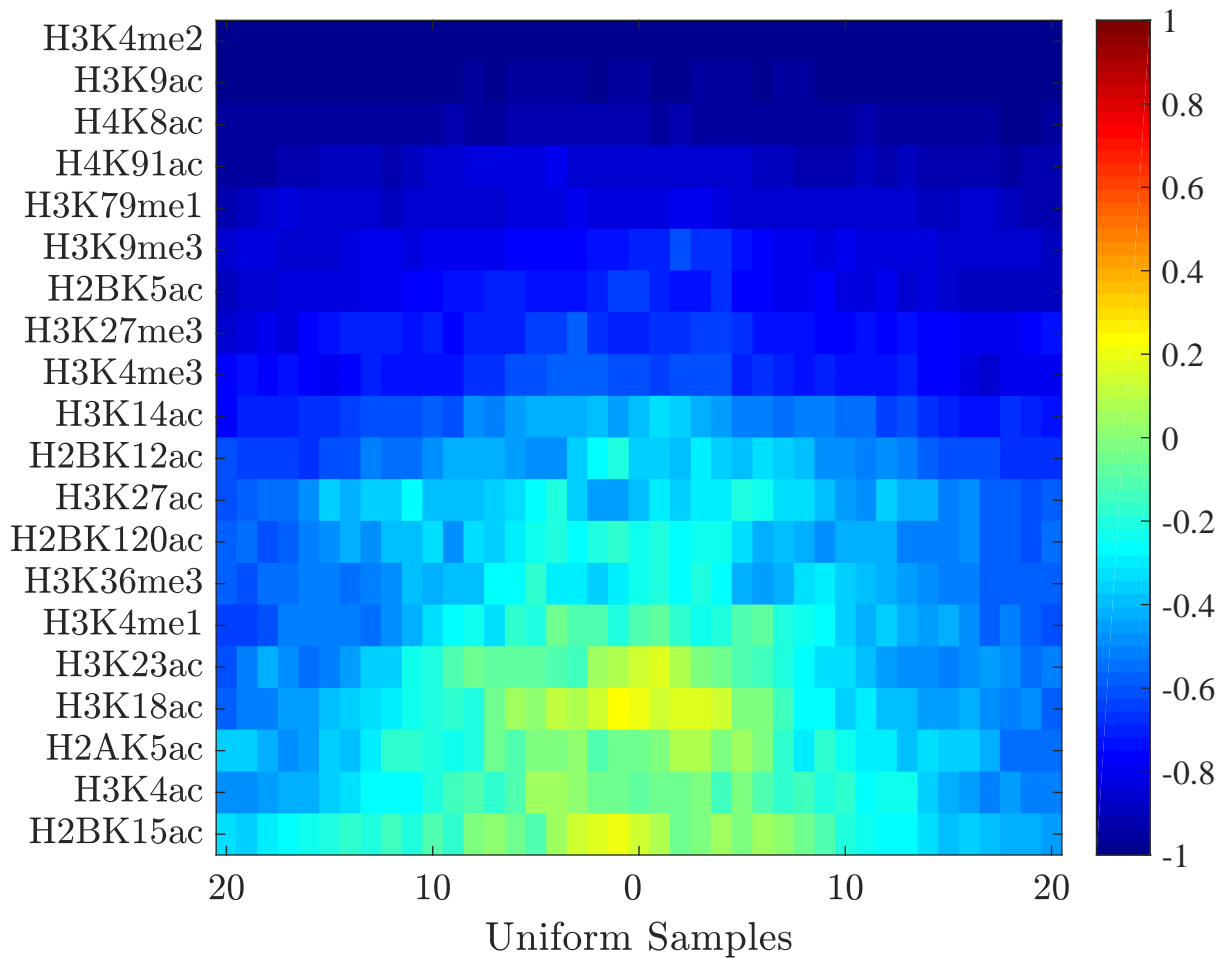

Supplement: Supplementary file 4 — HebbPlots of low-CpG promoters. This compressed file (.tar.gz) includes HebbPlots of low-CpG promoters active in 57 tissues/cell types. (TAR 2971 kb) [file 12859_2018_2312_MOESM4_ESM.tar › file5/E007.pdf]

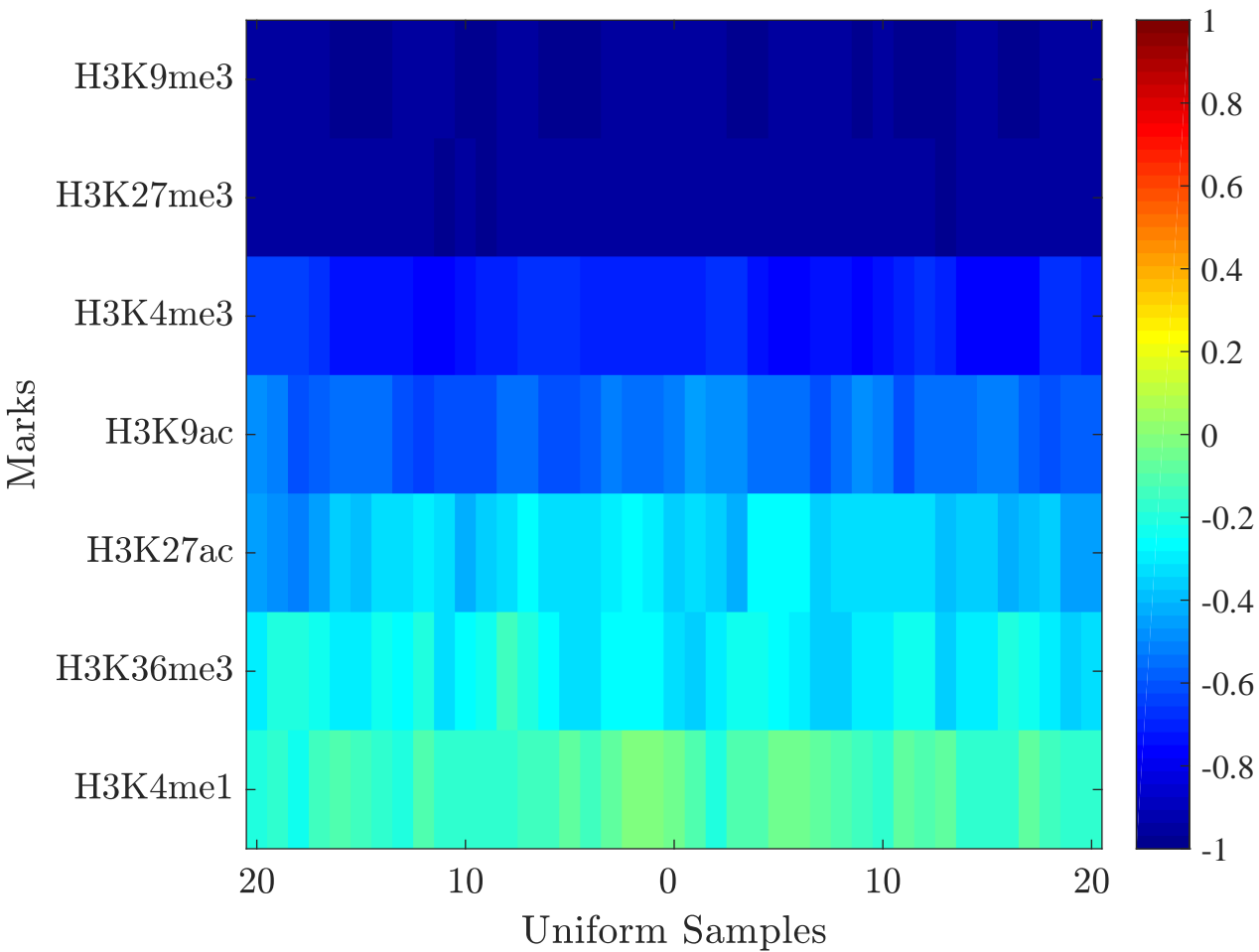

Supplement: Supplementary file 4 — HebbPlots of low-CpG promoters. This compressed file (.tar.gz) includes HebbPlots of low-CpG promoters active in 57 tissues/cell types. (TAR 2971 kb) [file 12859_2018_2312_MOESM4_ESM.tar › file5/E011.pdf]

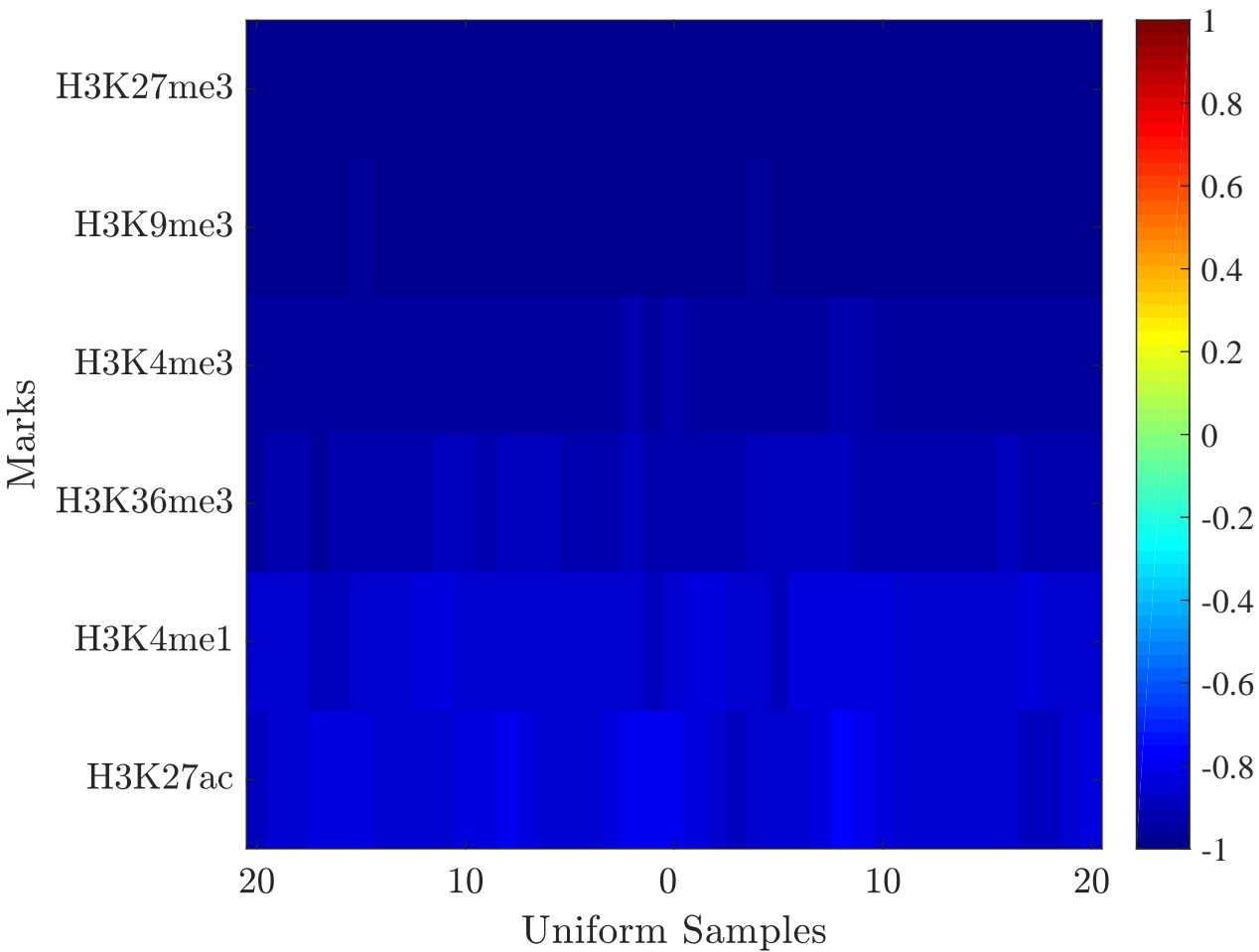

Supplement: Supplementary file 4 — HebbPlots of low-CpG promoters. This compressed file (.tar.gz) includes HebbPlots of low-CpG promoters active in 57 tissues/cell types. (TAR 2971 kb) [file 12859_2018_2312_MOESM4_ESM.tar › file5/E012.pdf]

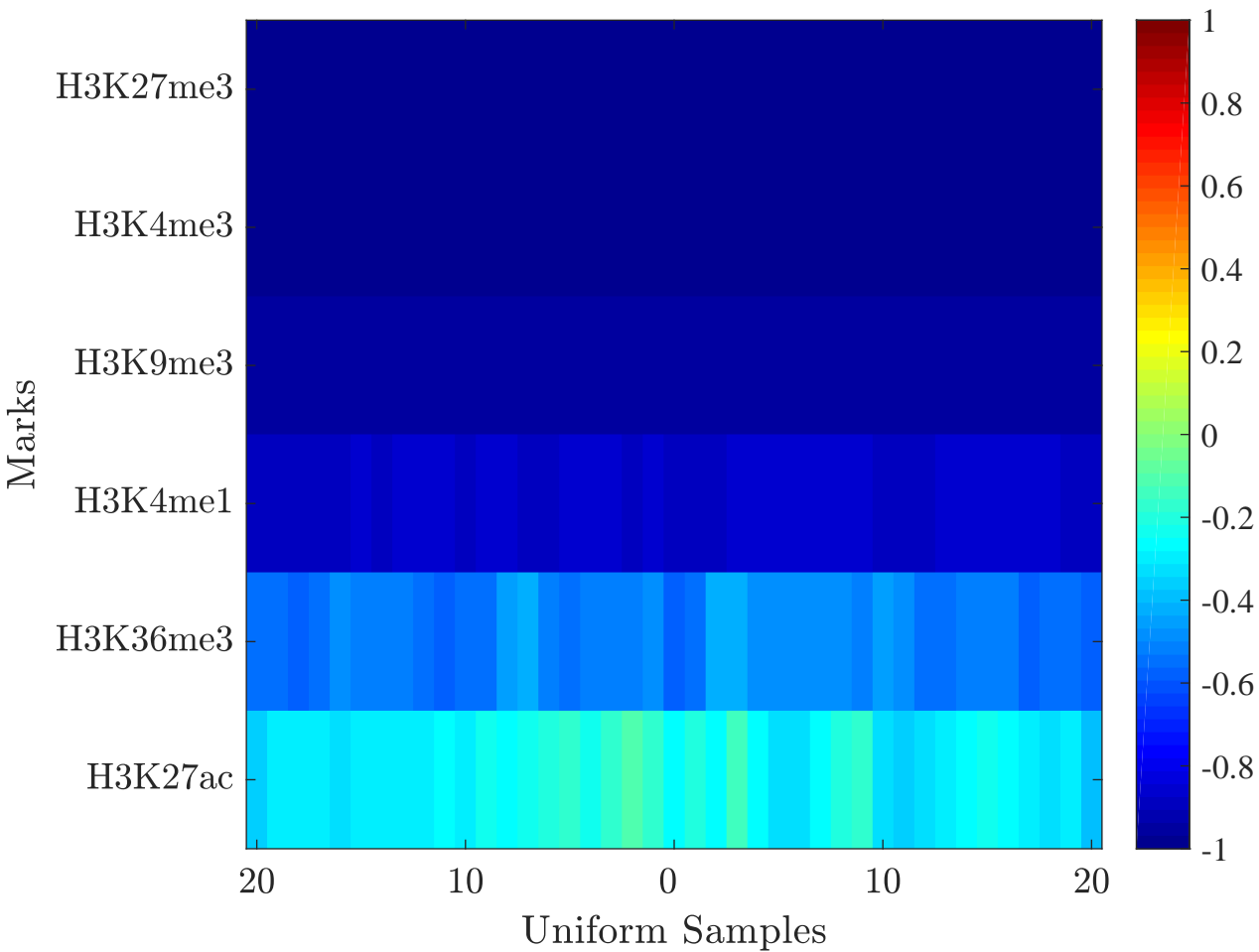

Supplement: Supplementary file 4 — HebbPlots of low-CpG promoters. This compressed file (.tar.gz) includes HebbPlots of low-CpG promoters active in 57 tissues/cell types. (TAR 2971 kb) [file 12859_2018_2312_MOESM4_ESM.tar › file5/E013.pdf]

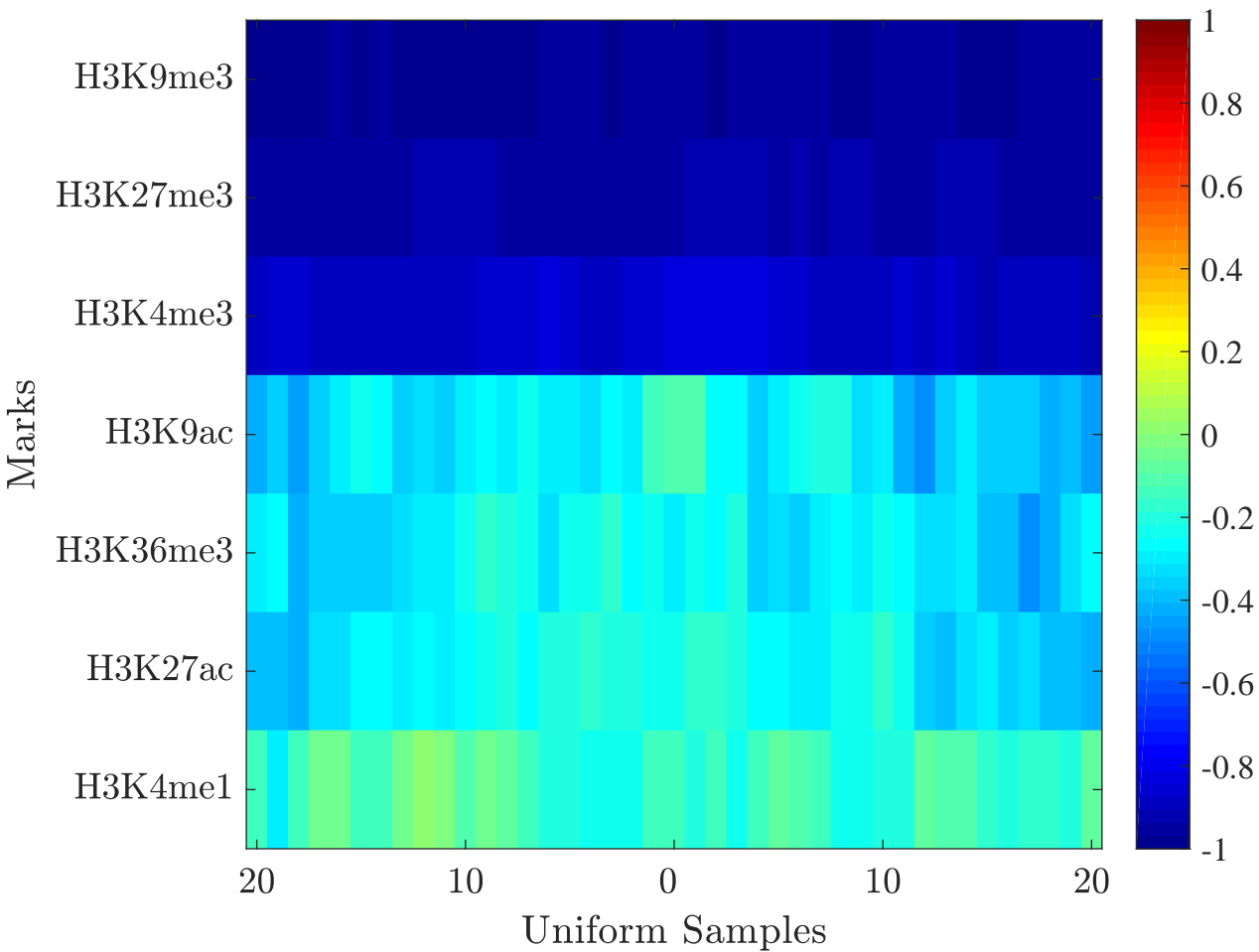

Supplement: Supplementary file 4 — HebbPlots of low-CpG promoters. This compressed file (.tar.gz) includes HebbPlots of low-CpG promoters active in 57 tissues/cell types. (TAR 2971 kb) [file 12859_2018_2312_MOESM4_ESM.tar › file5/E016.pdf]

Marks

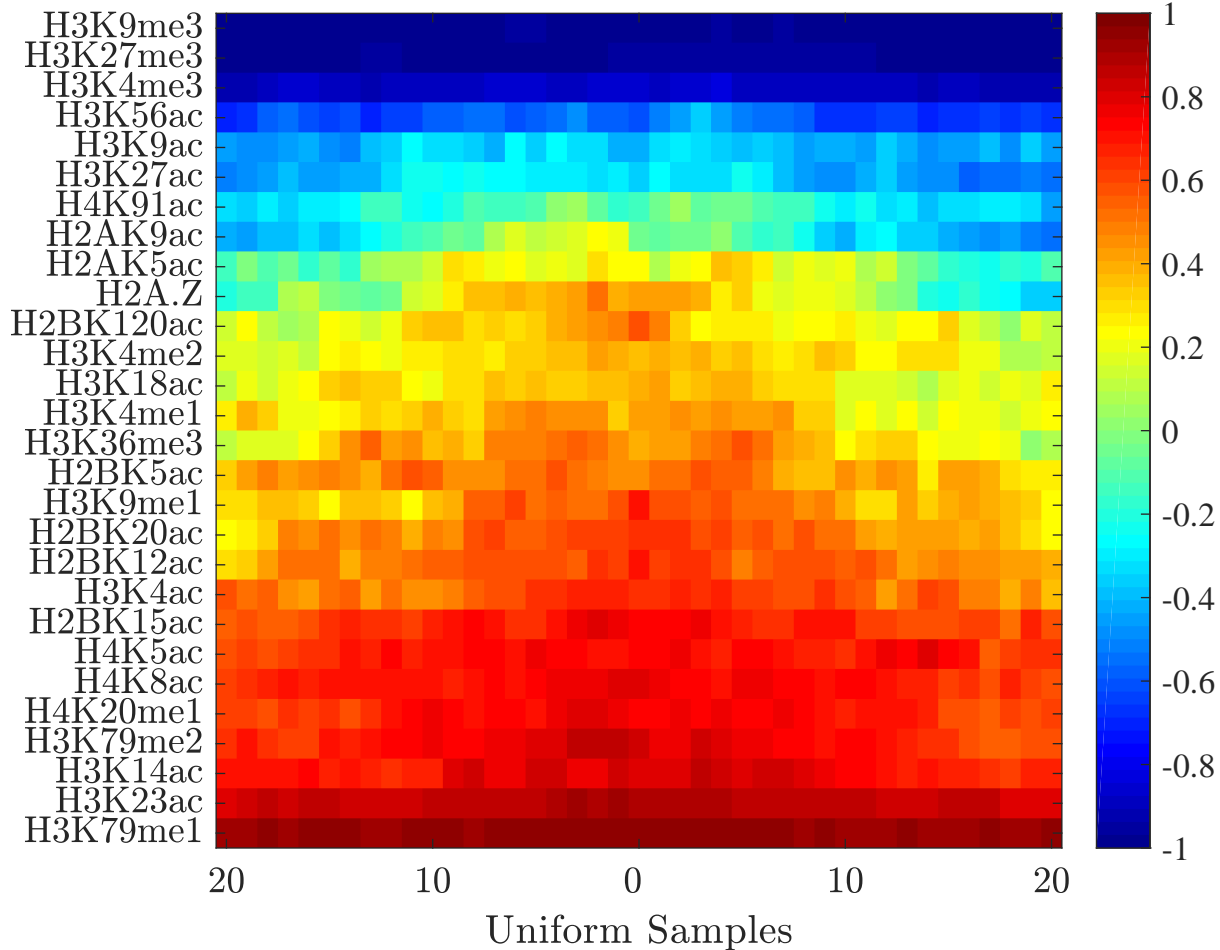

Supplement: Supplementary file 4 — HebbPlots of low-CpG promoters. This compressed file (.tar.gz) includes HebbPlots of low-CpG promoters active in 57 tissues/cell types. (TAR 2971 kb) [file 12859_2018_2312_MOESM4_ESM.tar › file5/E017.pdf]

Marks

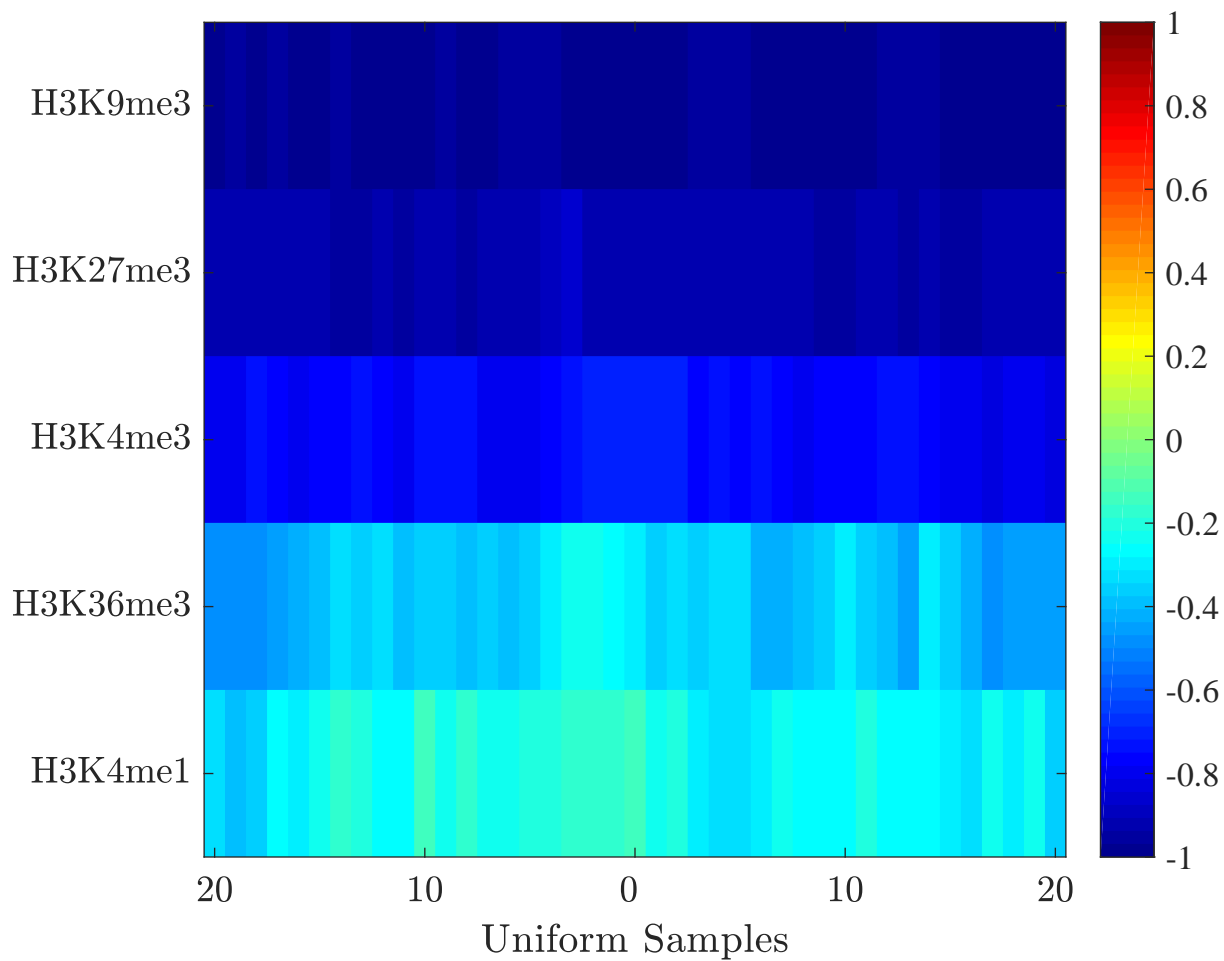

Supplement: Supplementary file 4 — HebbPlots of low-CpG promoters. This compressed file (.tar.gz) includes HebbPlots of low-CpG promoters active in 57 tissues/cell types. (TAR 2971 kb) [file 12859_2018_2312_MOESM4_ESM.tar › file5/E024.pdf]

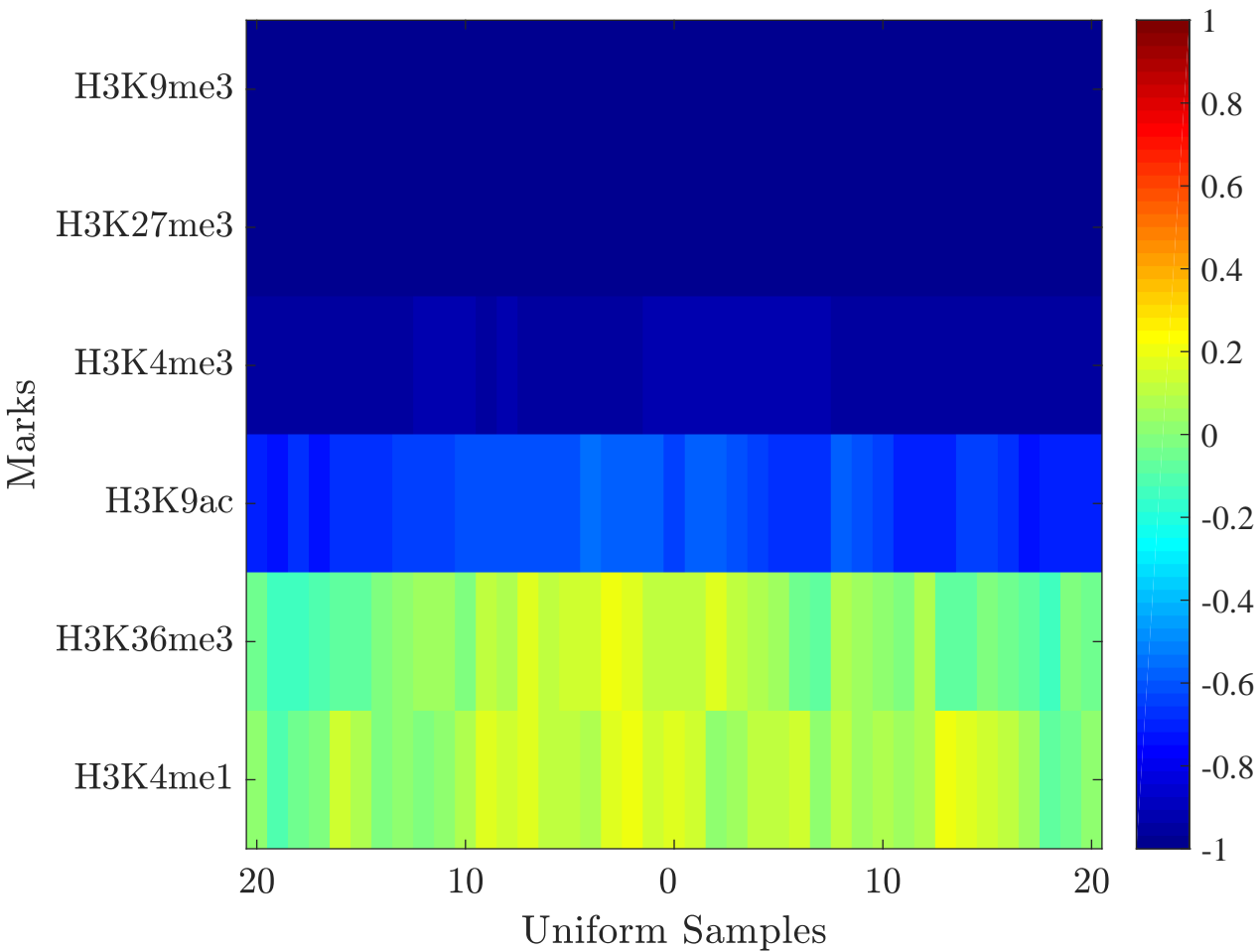

Supplement: Supplementary file 4 — HebbPlots of low-CpG promoters. This compressed file (.tar.gz) includes HebbPlots of low-CpG promoters active in 57 tissues/cell types. (TAR 2971 kb) [file 12859_2018_2312_MOESM4_ESM.tar › file5/E027.pdf]

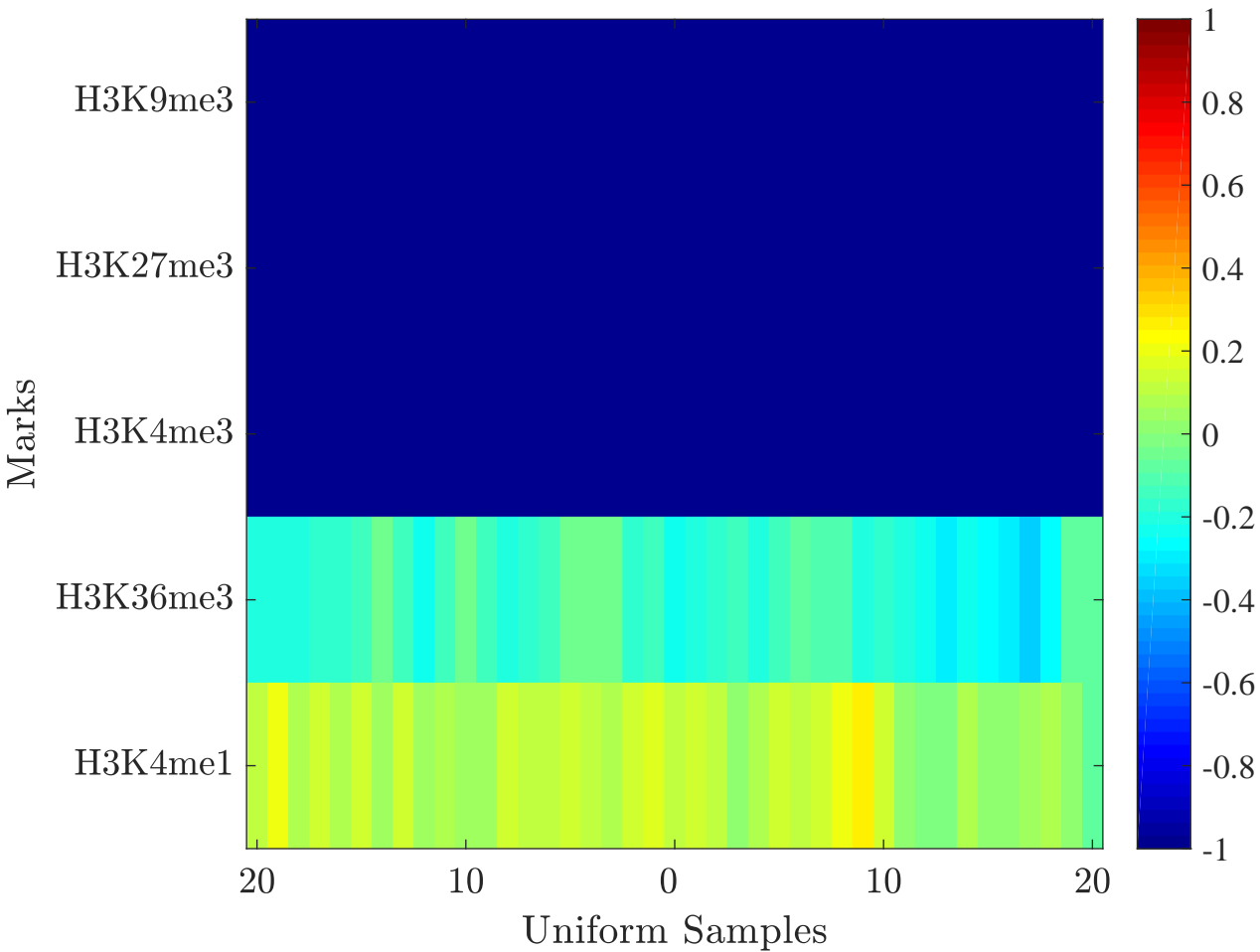

Supplement: Supplementary file 4 — HebbPlots of low-CpG promoters. This compressed file (.tar.gz) includes HebbPlots of low-CpG promoters active in 57 tissues/cell types. (TAR 2971 kb) [file 12859_2018_2312_MOESM4_ESM.tar › file5/E028.pdf]

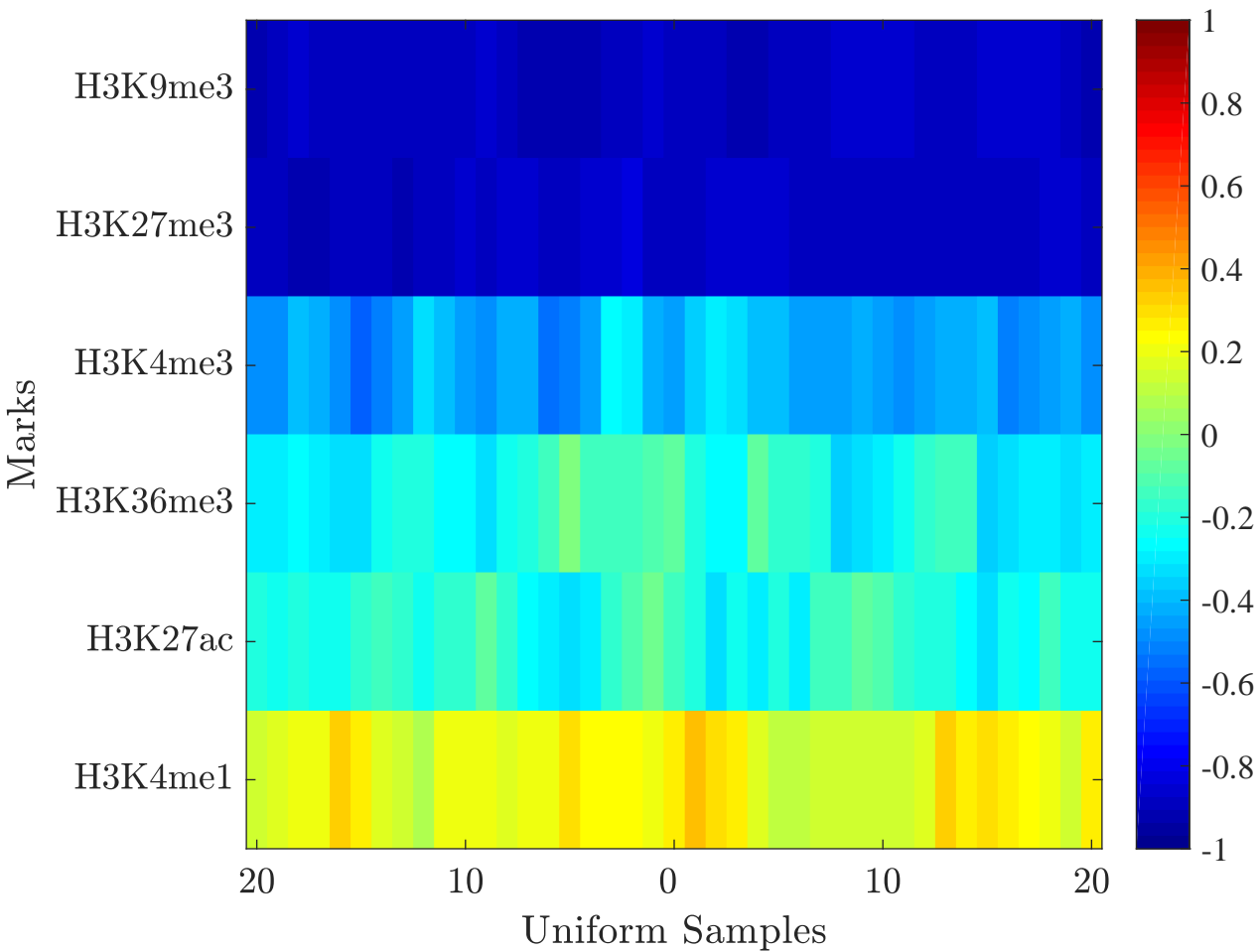

Supplement: Supplementary file 4 — HebbPlots of low-CpG promoters. This compressed file (.tar.gz) includes HebbPlots of low-CpG promoters active in 57 tissues/cell types. (TAR 2971 kb) [file 12859_2018_2312_MOESM4_ESM.tar › file5/E037.pdf]

Marks

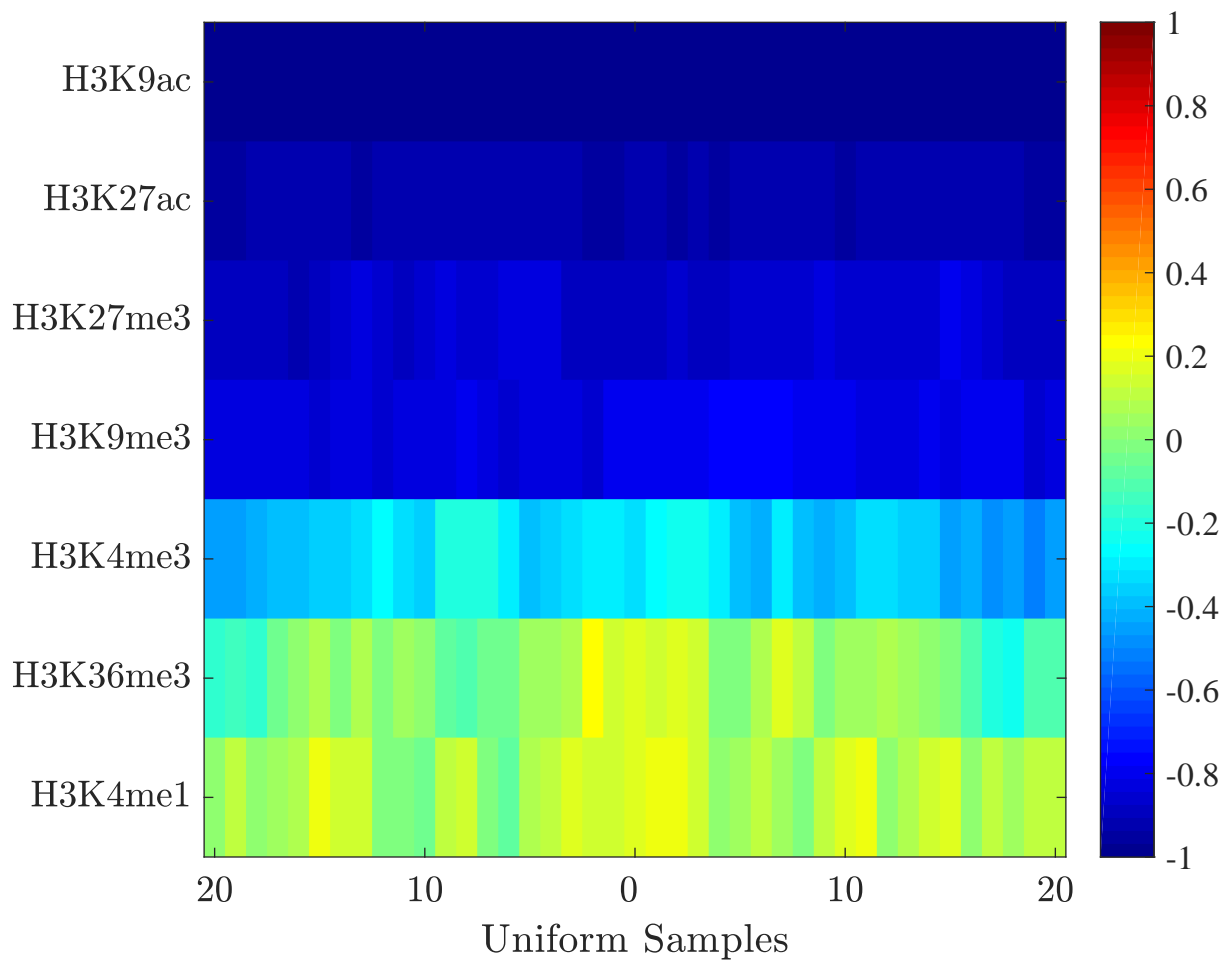

Supplement: Supplementary file 4 — HebbPlots of low-CpG promoters. This compressed file (.tar.gz) includes HebbPlots of low-CpG promoters active in 57 tissues/cell types. (TAR 2971 kb) [file 12859_2018_2312_MOESM4_ESM.tar › file5/E038.pdf]

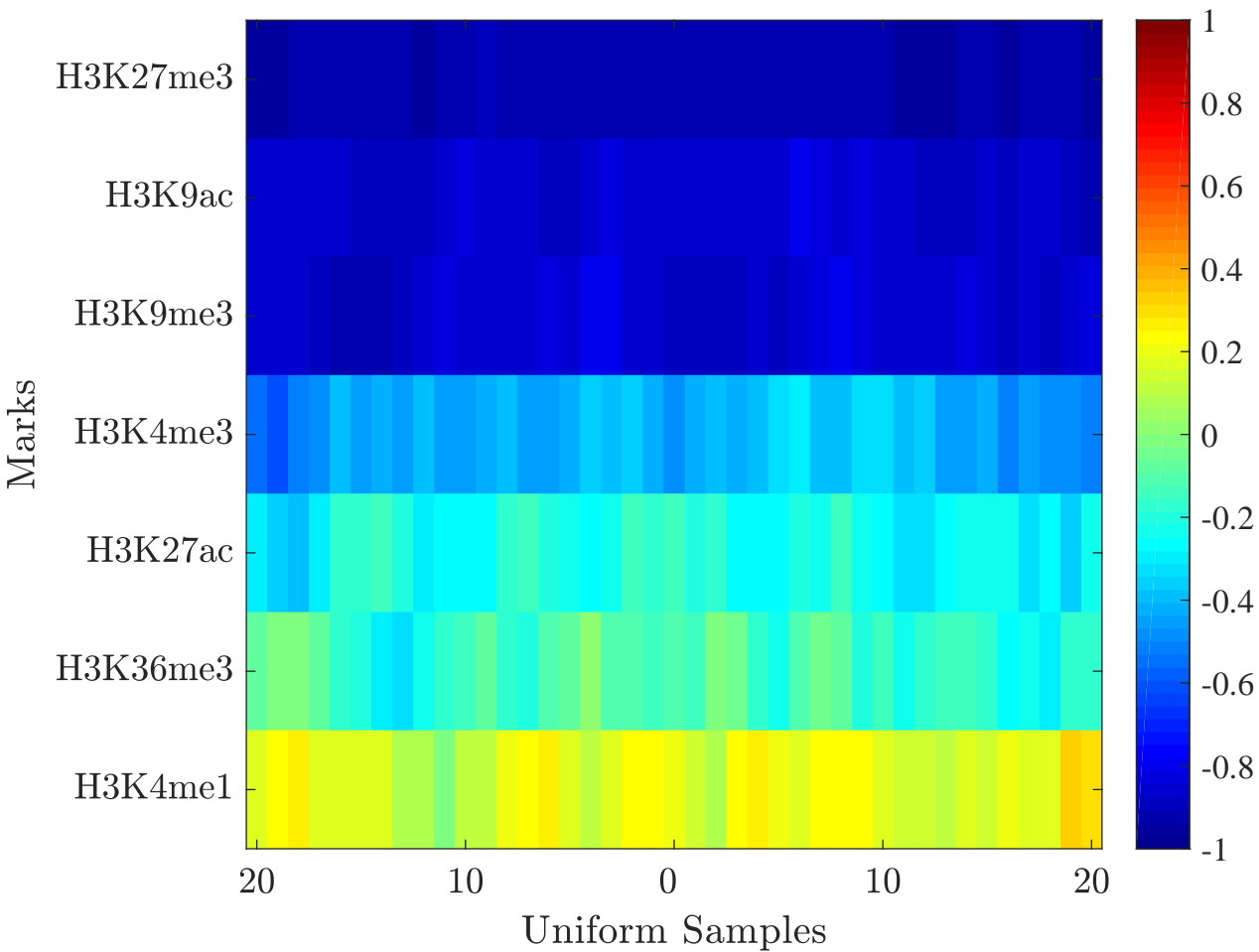

Supplement: Supplementary file 4 — HebbPlots of low-CpG promoters. This compressed file (.tar.gz) includes HebbPlots of low-CpG promoters active in 57 tissues/cell types. (TAR 2971 kb) [file 12859_2018_2312_MOESM4_ESM.tar › file5/E047.pdf]

Marks

H3K27me3

H3K9me3

H3K4me3

H3K27ac

H3K36me3

H3K4me1

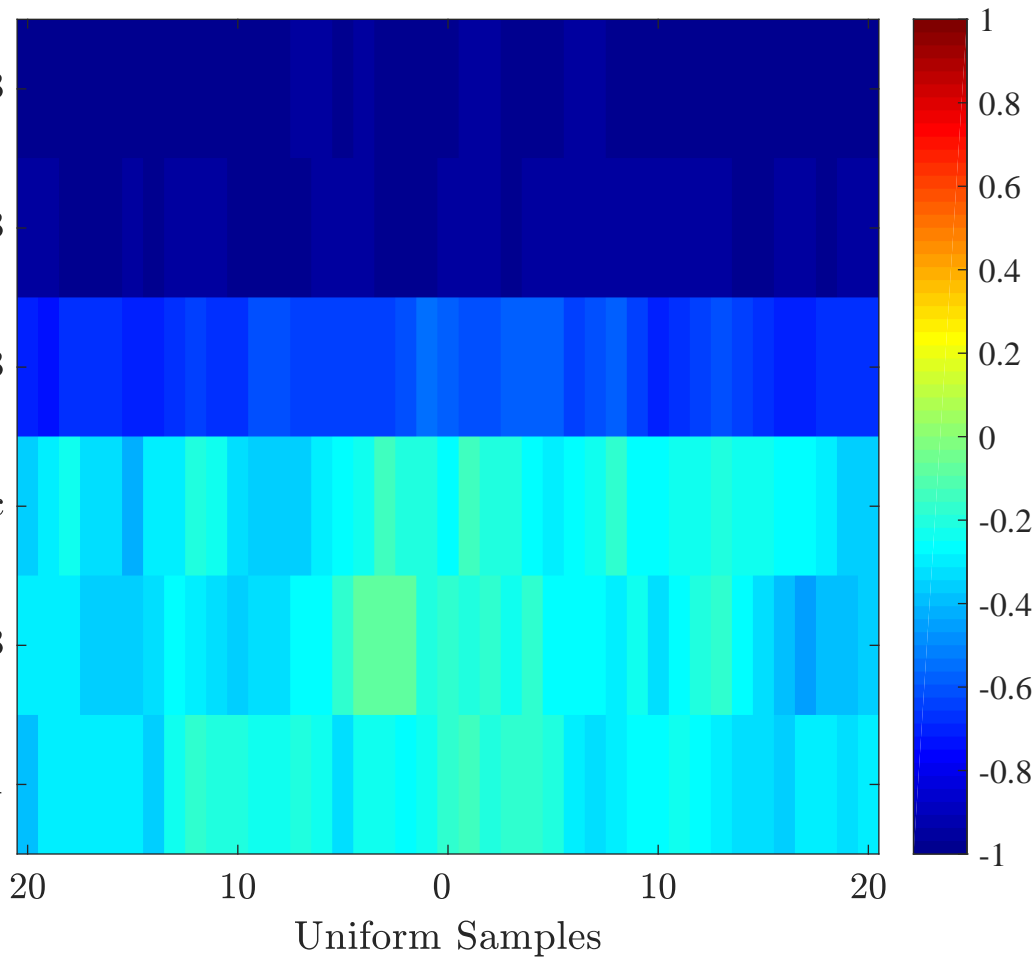

Supplement: Supplementary file 4 — HebbPlots of low-CpG promoters. This compressed file (.tar.gz) includes HebbPlots of low-CpG promoters active in 57 tissues/cell types. (TAR 2971 kb) [file 12859_2018_2312_MOESM4_ESM.tar › file5/E050.pdf]

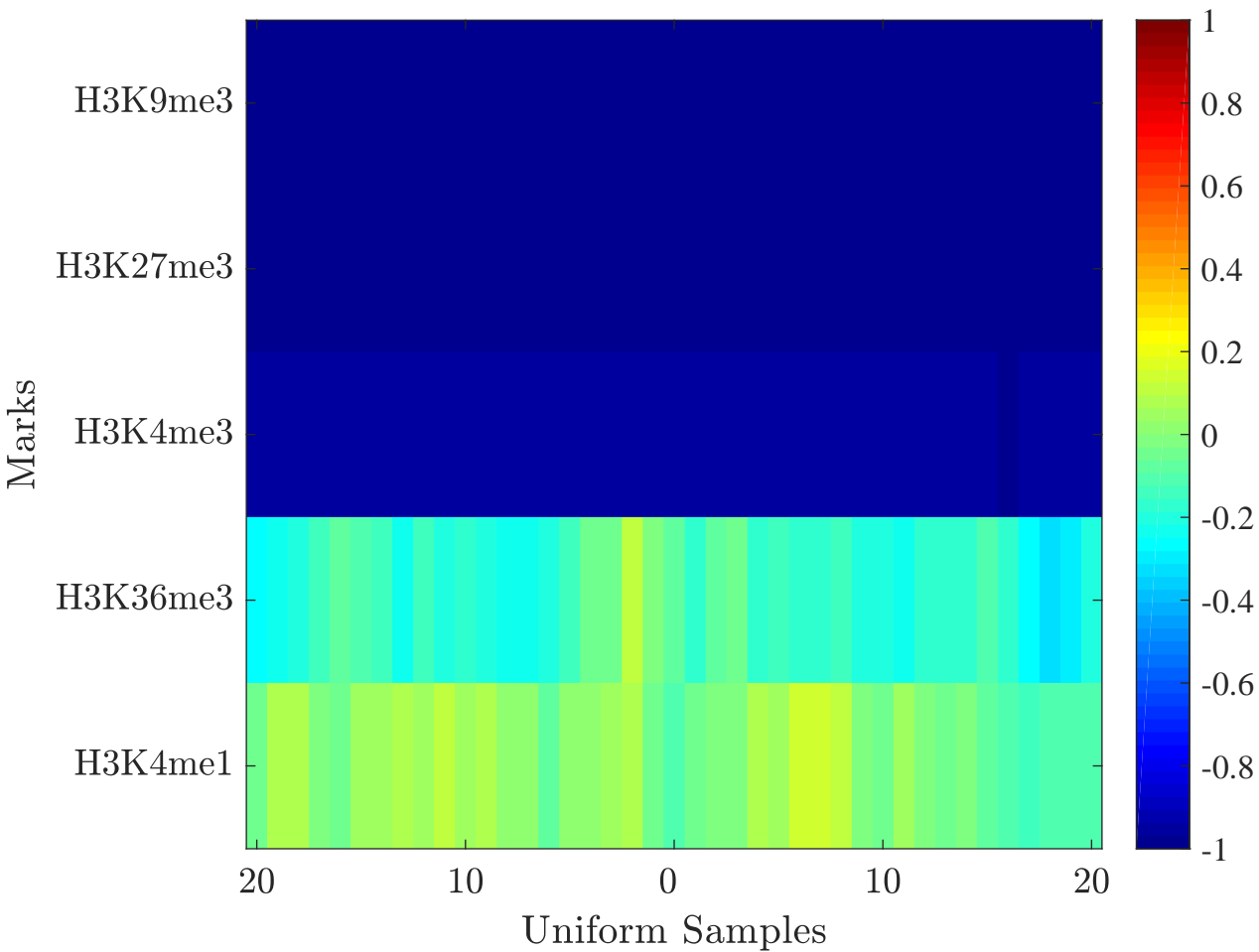

Supplement: Supplementary file 4 — HebbPlots of low-CpG promoters. This compressed file (.tar.gz) includes HebbPlots of low-CpG promoters active in 57 tissues/cell types. (TAR 2971 kb) [file 12859_2018_2312_MOESM4_ESM.tar › file5/E053.pdf]

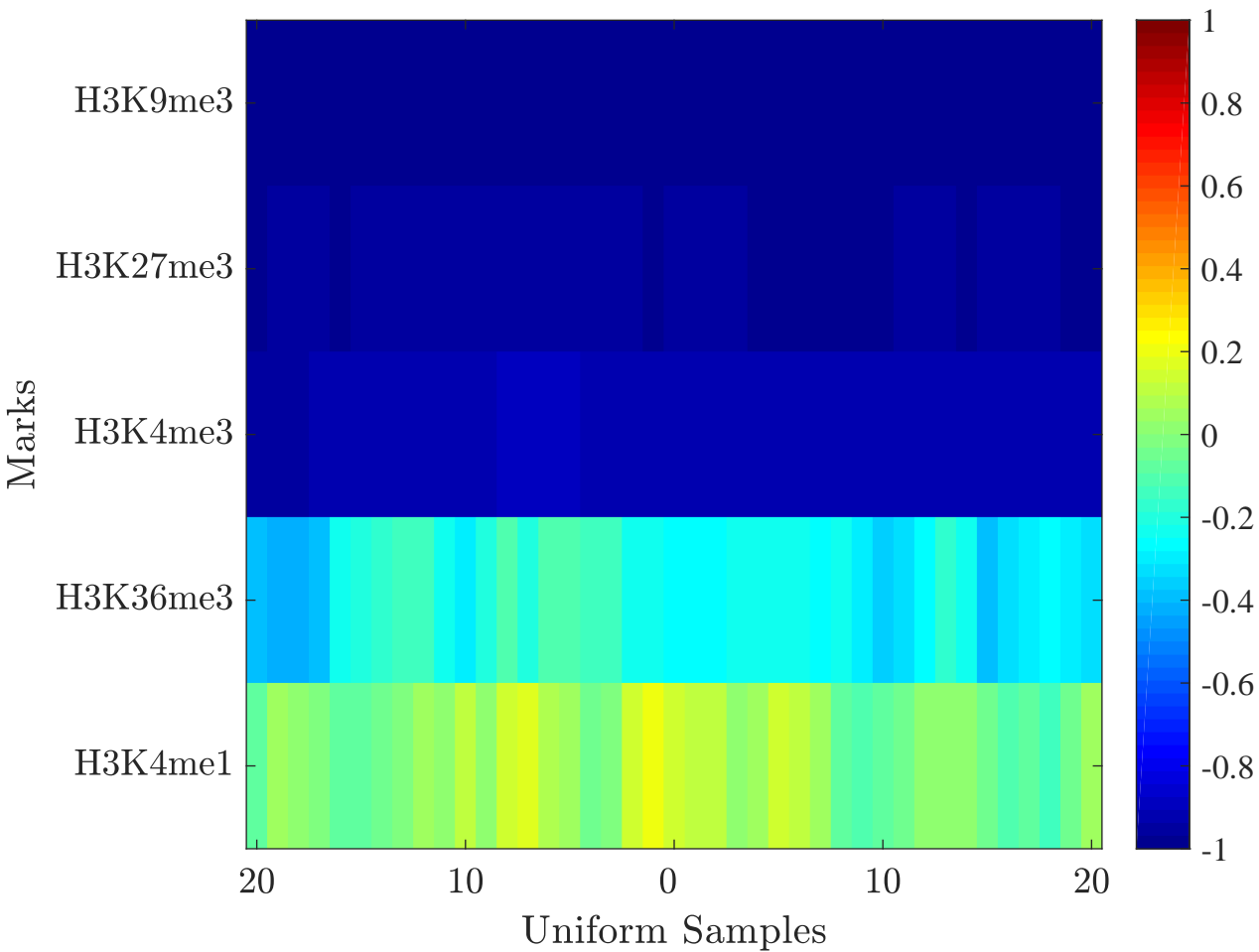

Supplement: Supplementary file 4 — HebbPlots of low-CpG promoters. This compressed file (.tar.gz) includes HebbPlots of low-CpG promoters active in 57 tissues/cell types. (TAR 2971 kb) [file 12859_2018_2312_MOESM4_ESM.tar › file5/E054.pdf]

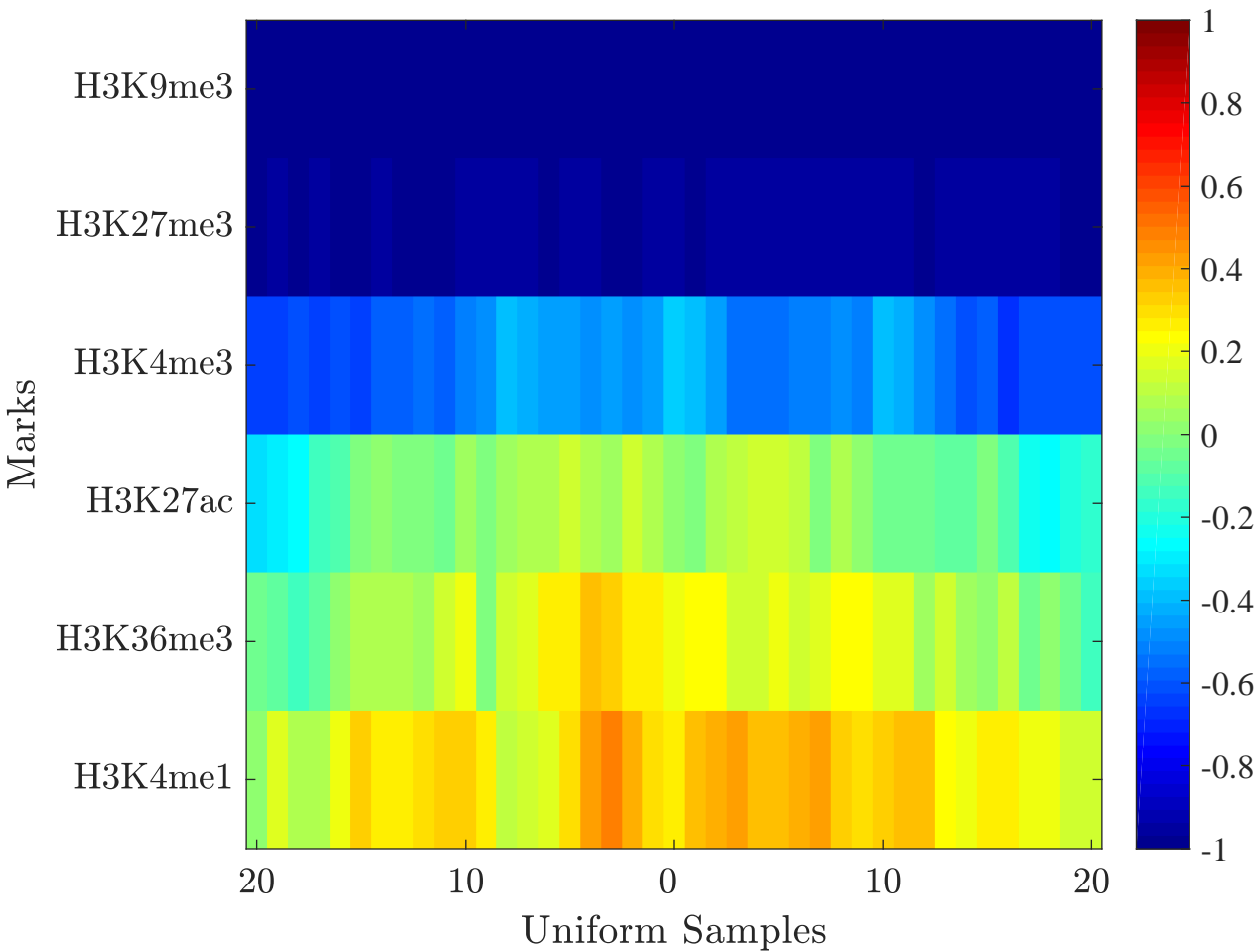

Supplement: Supplementary file 4 — HebbPlots of low-CpG promoters. This compressed file (.tar.gz) includes HebbPlots of low-CpG promoters active in 57 tissues/cell types. (TAR 2971 kb) [file 12859_2018_2312_MOESM4_ESM.tar › file5/E055.pdf]

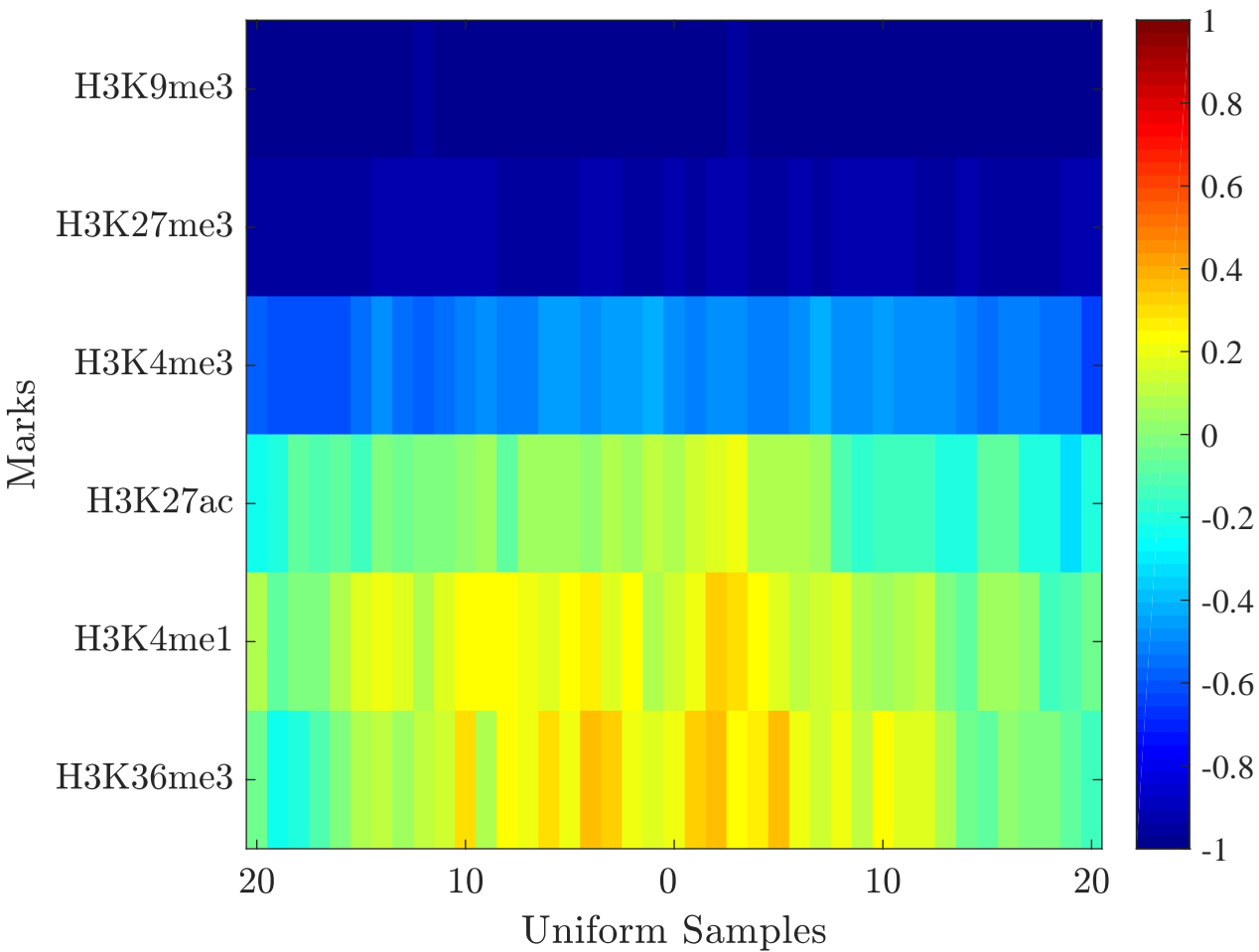

Supplement: Supplementary file 4 — HebbPlots of low-CpG promoters. This compressed file (.tar.gz) includes HebbPlots of low-CpG promoters active in 57 tissues/cell types. (TAR 2971 kb) [file 12859_2018_2312_MOESM4_ESM.tar › file5/E056.pdf]

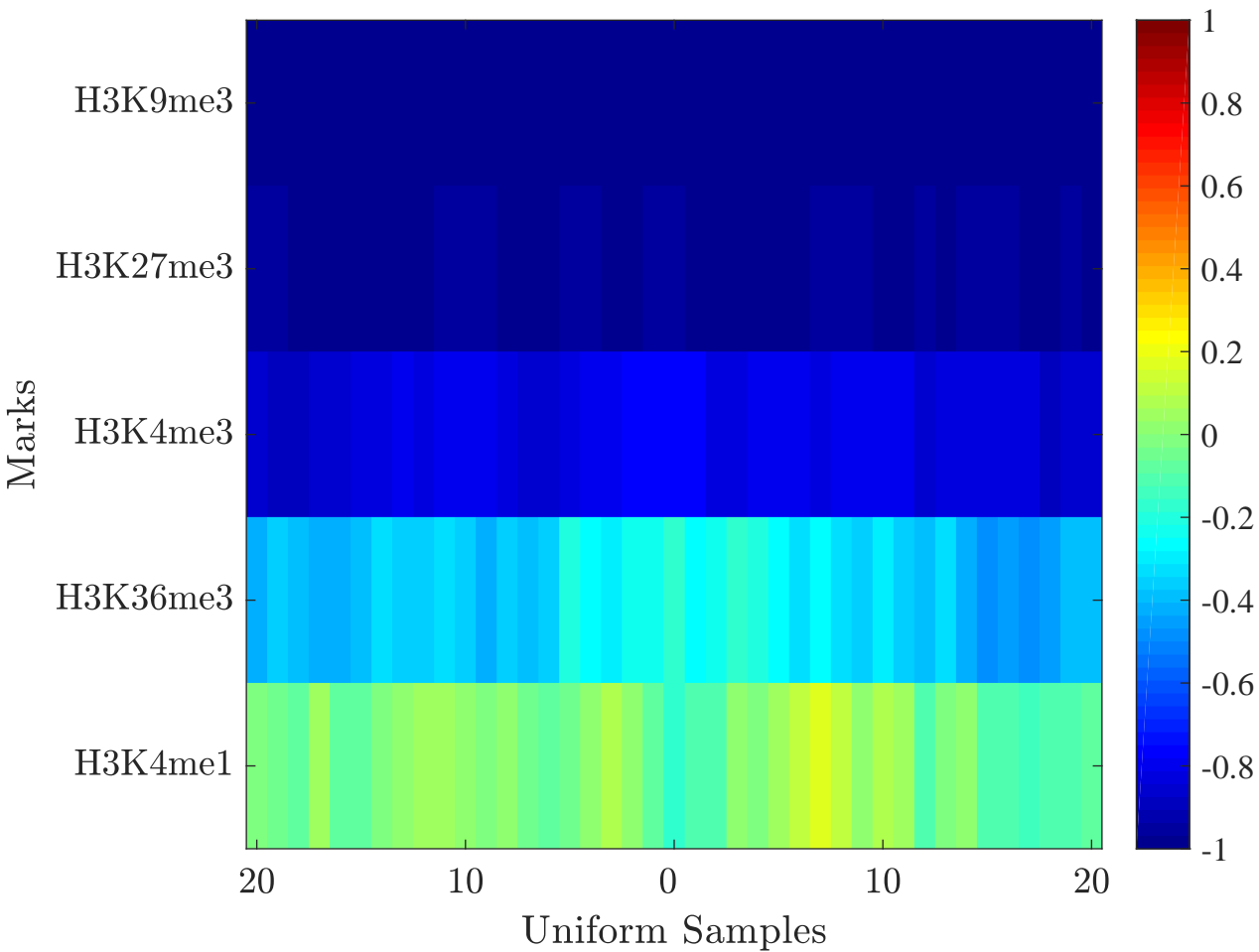

Supplement: Supplementary file 4 — HebbPlots of low-CpG promoters. This compressed file (.tar.gz) includes HebbPlots of low-CpG promoters active in 57 tissues/cell types. (TAR 2971 kb) [file 12859_2018_2312_MOESM4_ESM.tar › file5/E057.pdf]

Marks

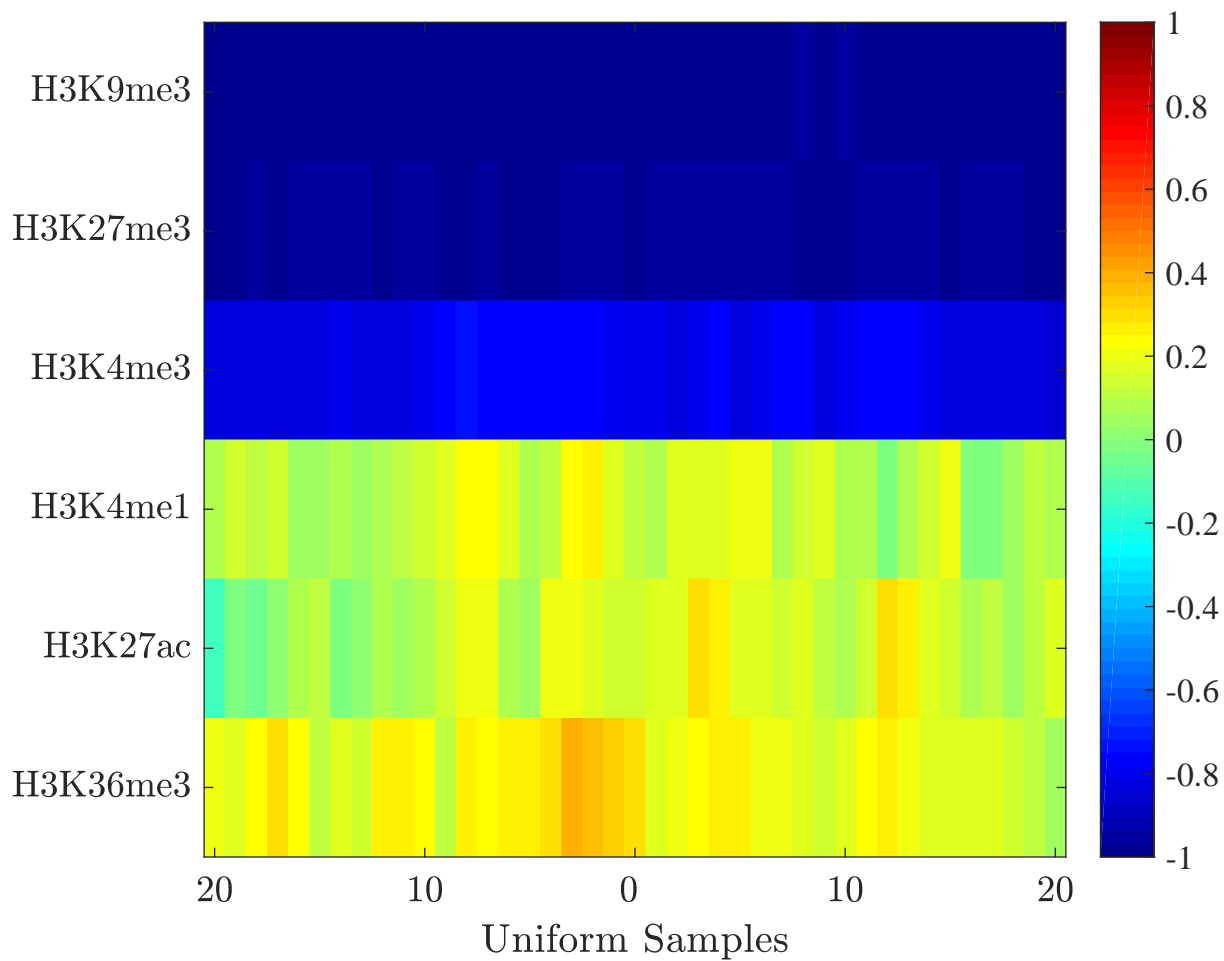

Supplement: Supplementary file 4 — HebbPlots of low-CpG promoters. This compressed file (.tar.gz) includes HebbPlots of low-CpG promoters active in 57 tissues/cell types. (TAR 2971 kb) [file 12859_2018_2312_MOESM4_ESM.tar › file5/E058.pdf]

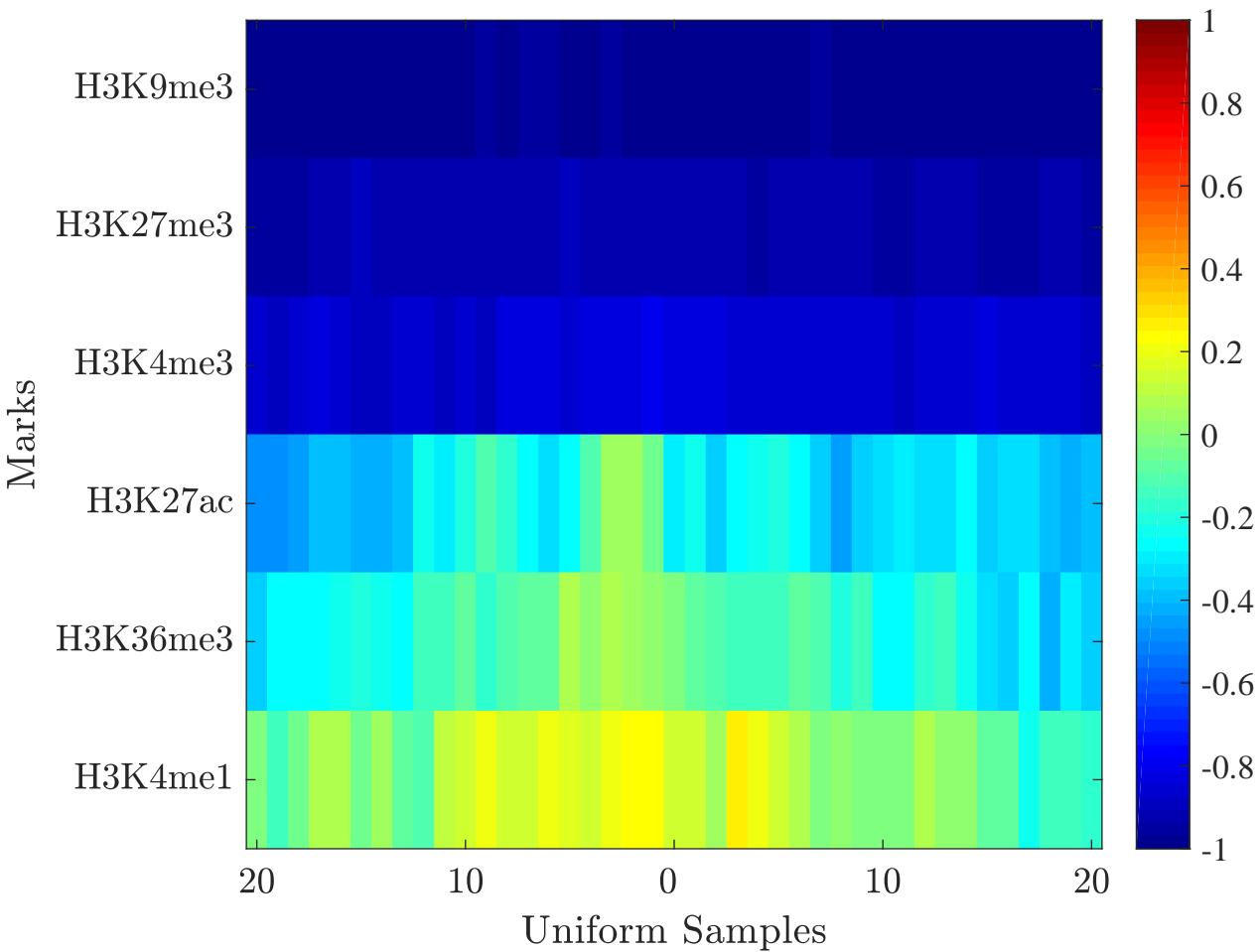

Supplement: Supplementary file 4 — HebbPlots of low-CpG promoters. This compressed file (.tar.gz) includes HebbPlots of low-CpG promoters active in 57 tissues/cell types. (TAR 2971 kb) [file 12859_2018_2312_MOESM4_ESM.tar › file5/E059.pdf]

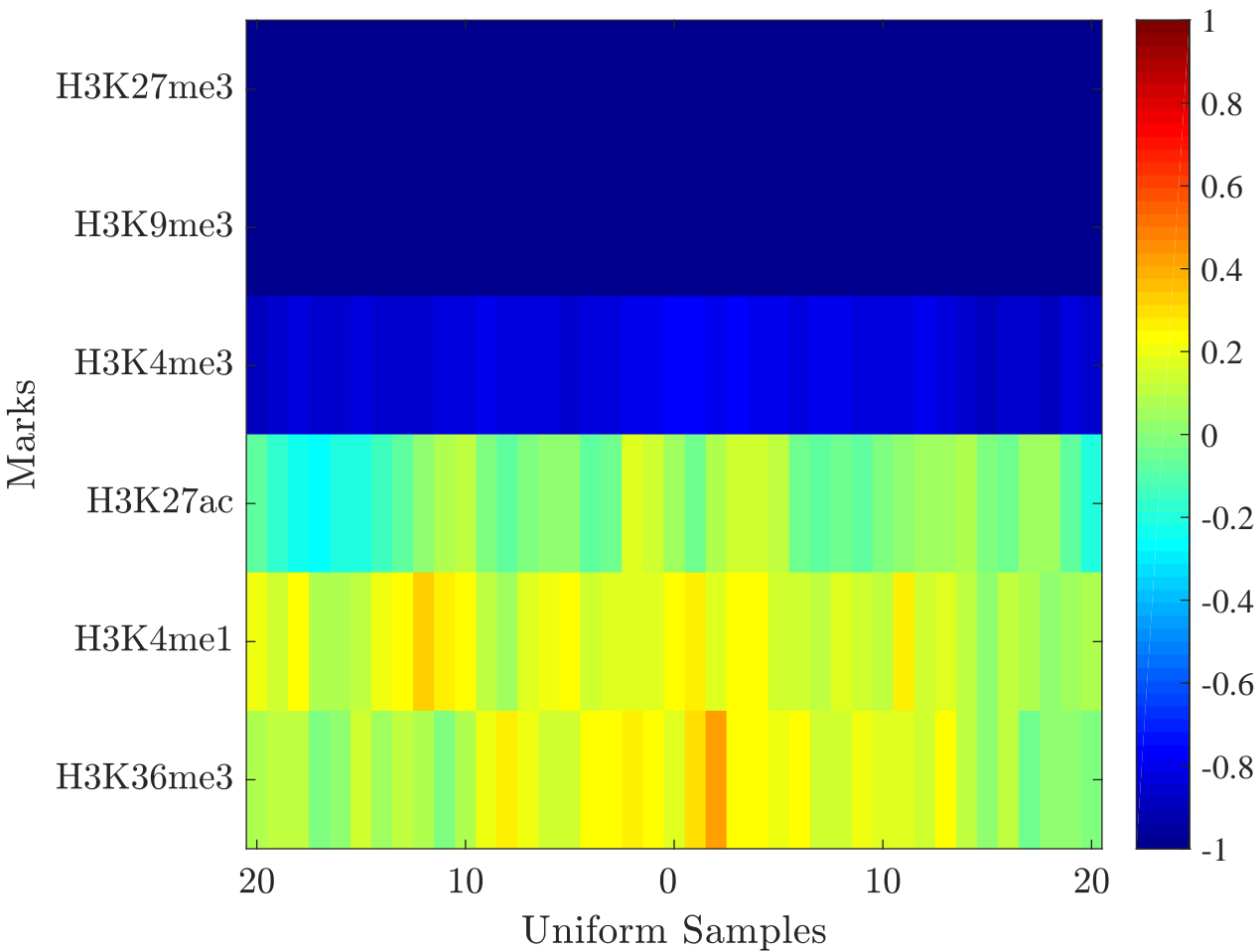

Supplement: Supplementary file 4 — HebbPlots of low-CpG promoters. This compressed file (.tar.gz) includes HebbPlots of low-CpG promoters active in 57 tissues/cell types. (TAR 2971 kb) [file 12859_2018_2312_MOESM4_ESM.tar › file5/E061.pdf]

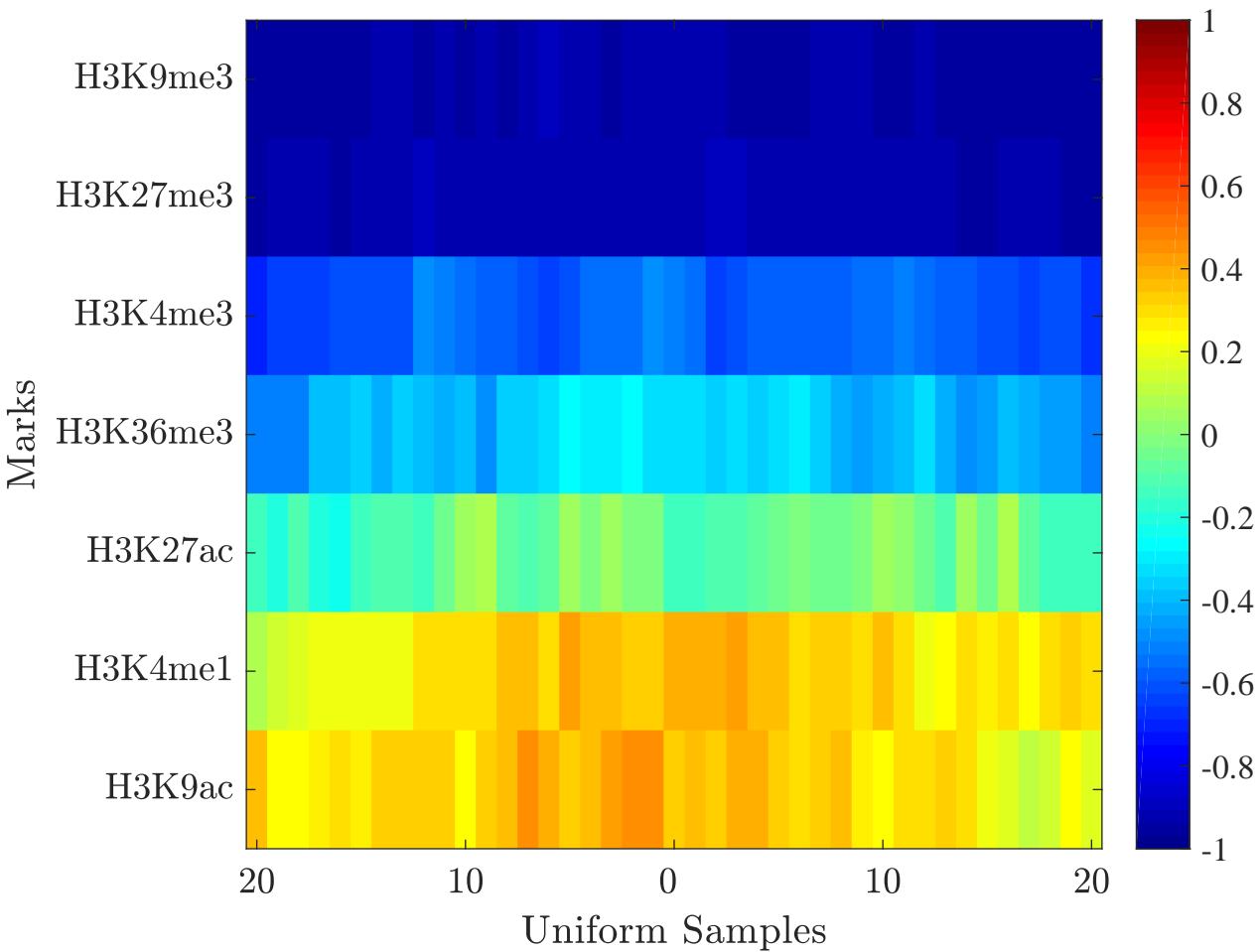

Supplement: Supplementary file 4 — HebbPlots of low-CpG promoters. This compressed file (.tar.gz) includes HebbPlots of low-CpG promoters active in 57 tissues/cell types. (TAR 2971 kb) [file 12859_2018_2312_MOESM4_ESM.tar › file5/E062.pdf]

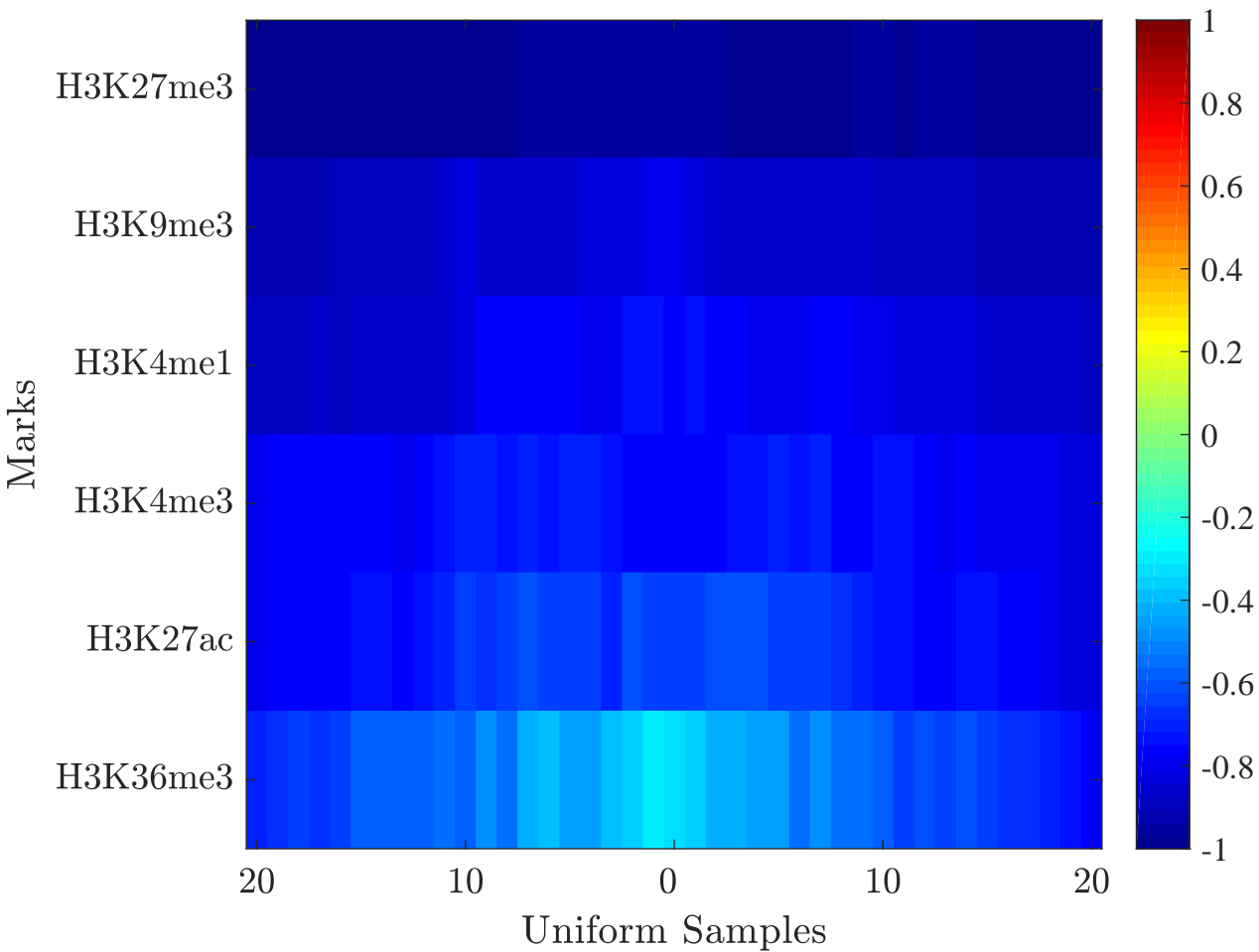

Supplement: Supplementary file 4 — HebbPlots of low-CpG promoters. This compressed file (.tar.gz) includes HebbPlots of low-CpG promoters active in 57 tissues/cell types. (TAR 2971 kb) [file 12859_2018_2312_MOESM4_ESM.tar › file5/E065.pdf]

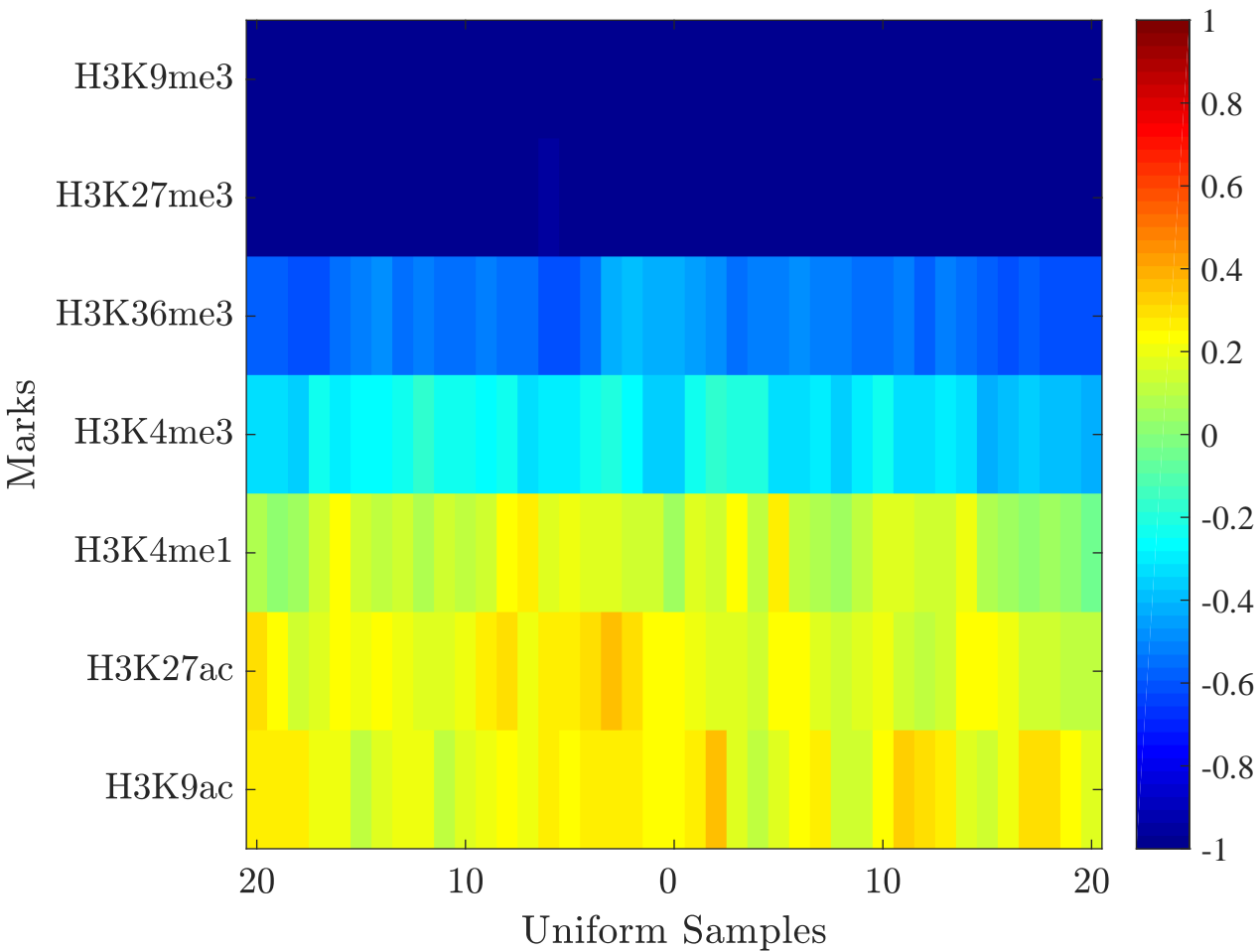

Supplement: Supplementary file 4 — HebbPlots of low-CpG promoters. This compressed file (.tar.gz) includes HebbPlots of low-CpG promoters active in 57 tissues/cell types. (TAR 2971 kb) [file 12859_2018_2312_MOESM4_ESM.tar › file5/E066.pdf]

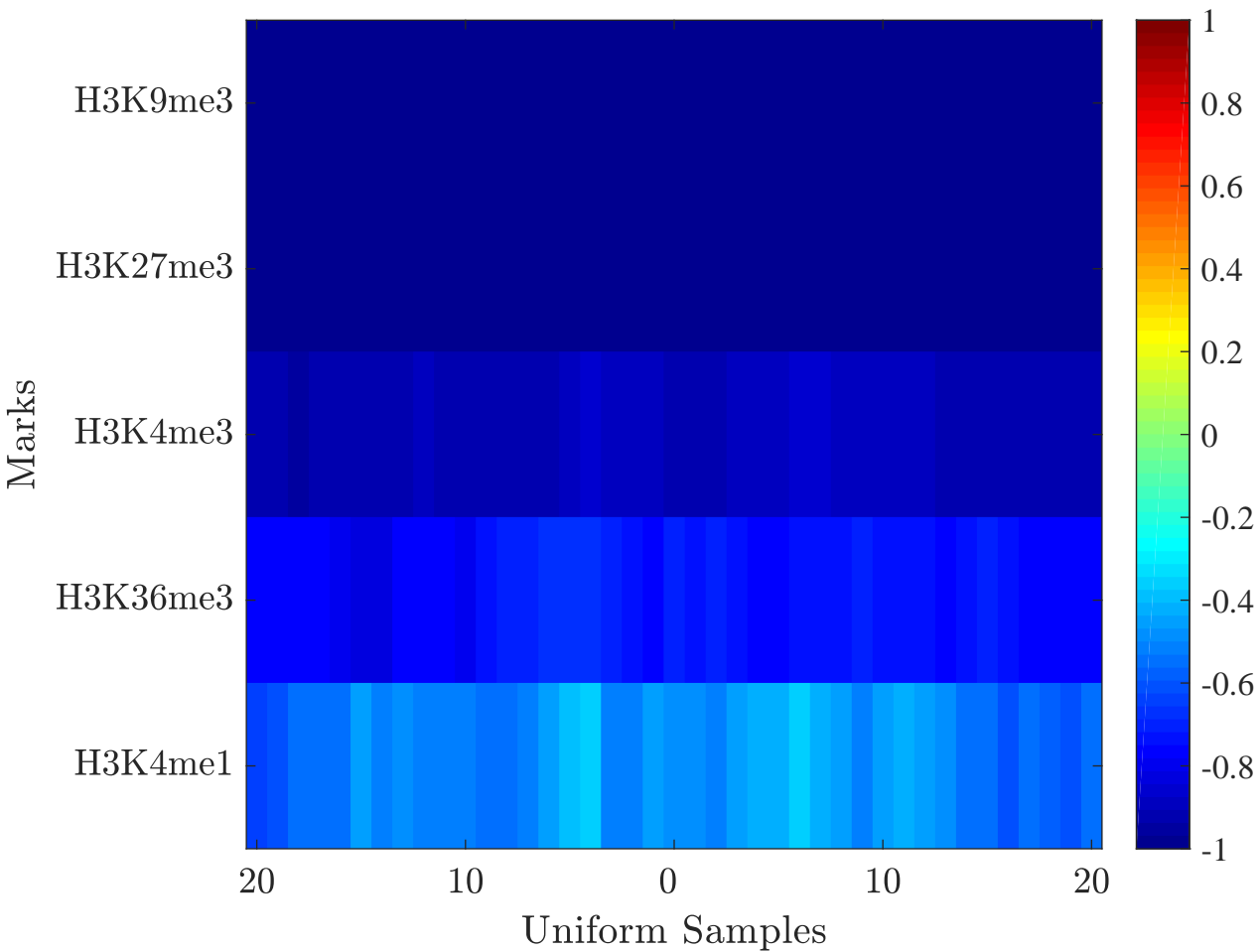

Supplement: Supplementary file 4 — HebbPlots of low-CpG promoters. This compressed file (.tar.gz) includes HebbPlots of low-CpG promoters active in 57 tissues/cell types. (TAR 2971 kb) [file 12859_2018_2312_MOESM4_ESM.tar › file5/E070.pdf]
