# Supplementary figures and images for: HebbPlot: an intelligent tool for learning and visualizing chromatin mark signatures (part 3 of 4)
Source: BMC Bioinformatics. 2018 Sep 3;19:310. doi: 10.1186/s12859-018-2312-1 (PMC6122555; doi:10.1186/s12859-018-2312-1)

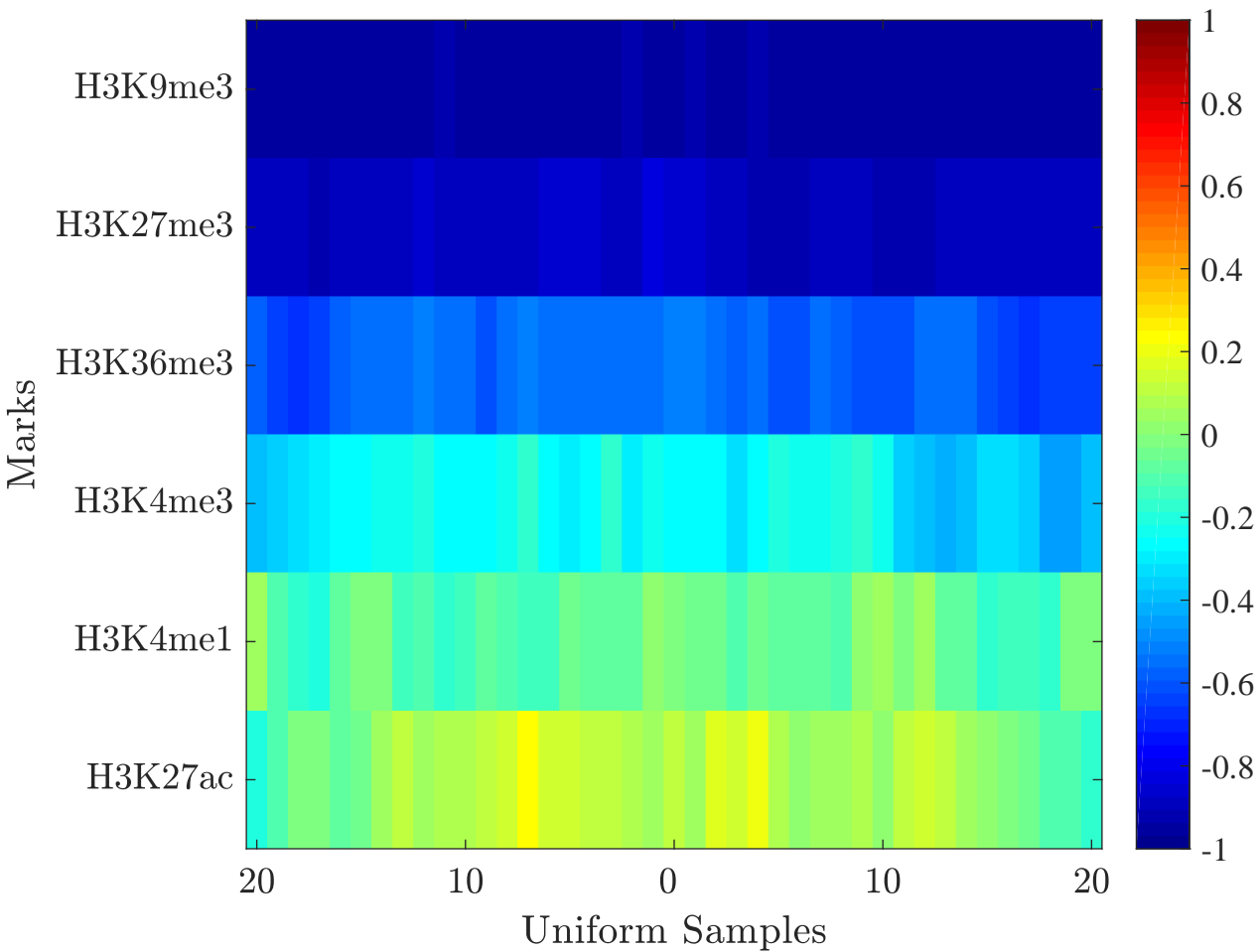

Supplement: Supplementary file 4 — HebbPlots of low-CpG promoters. This compressed file (.tar.gz) includes HebbPlots of low-CpG promoters active in 57 tissues/cell types. (TAR 2971 kb) [file 12859_2018_2312_MOESM4_ESM.tar › file5/E071.pdf]

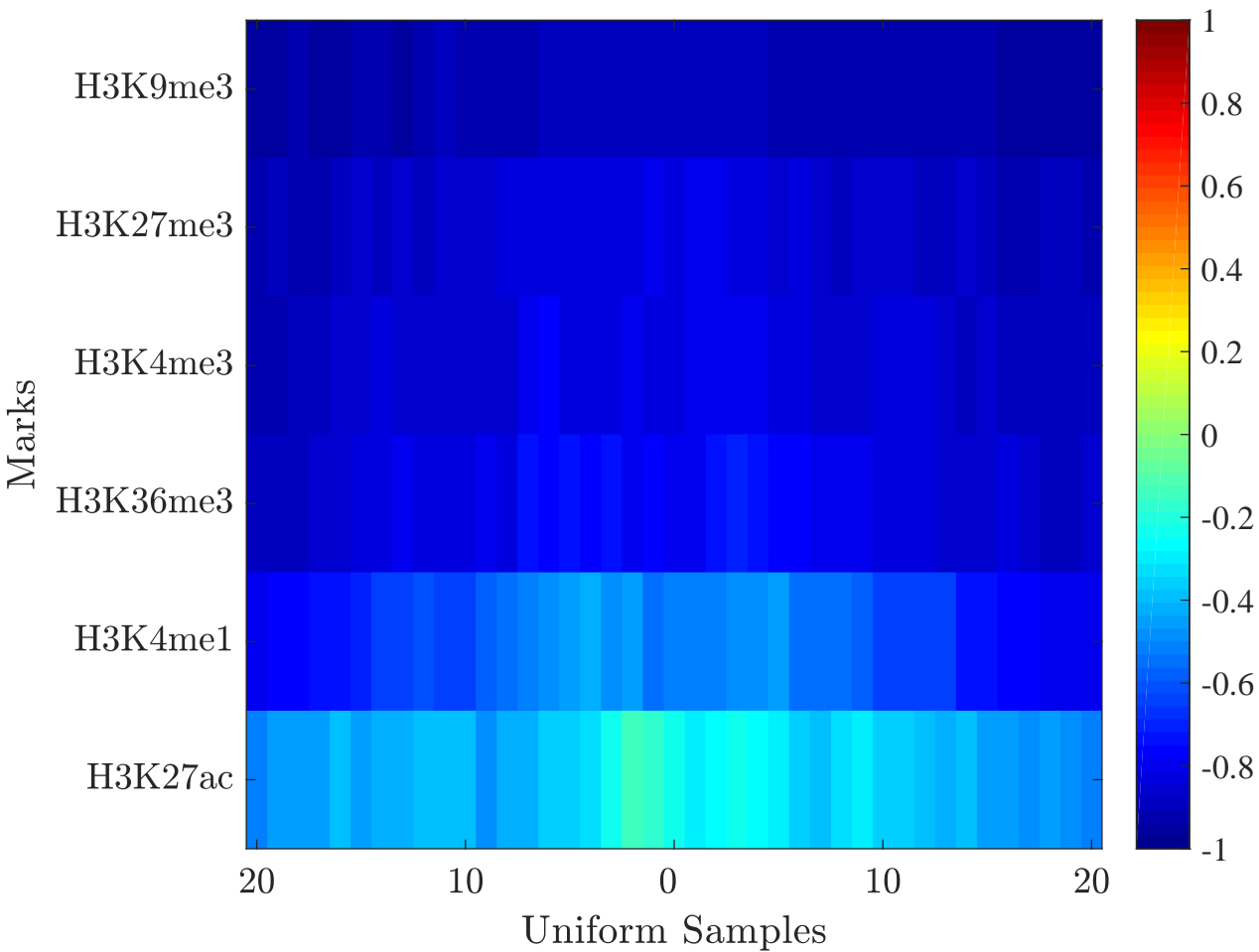

Supplement: Supplementary file 4 — HebbPlots of low-CpG promoters. This compressed file (.tar.gz) includes HebbPlots of low-CpG promoters active in 57 tissues/cell types. (TAR 2971 kb) [file 12859_2018_2312_MOESM4_ESM.tar › file5/E079.pdf]

Marks

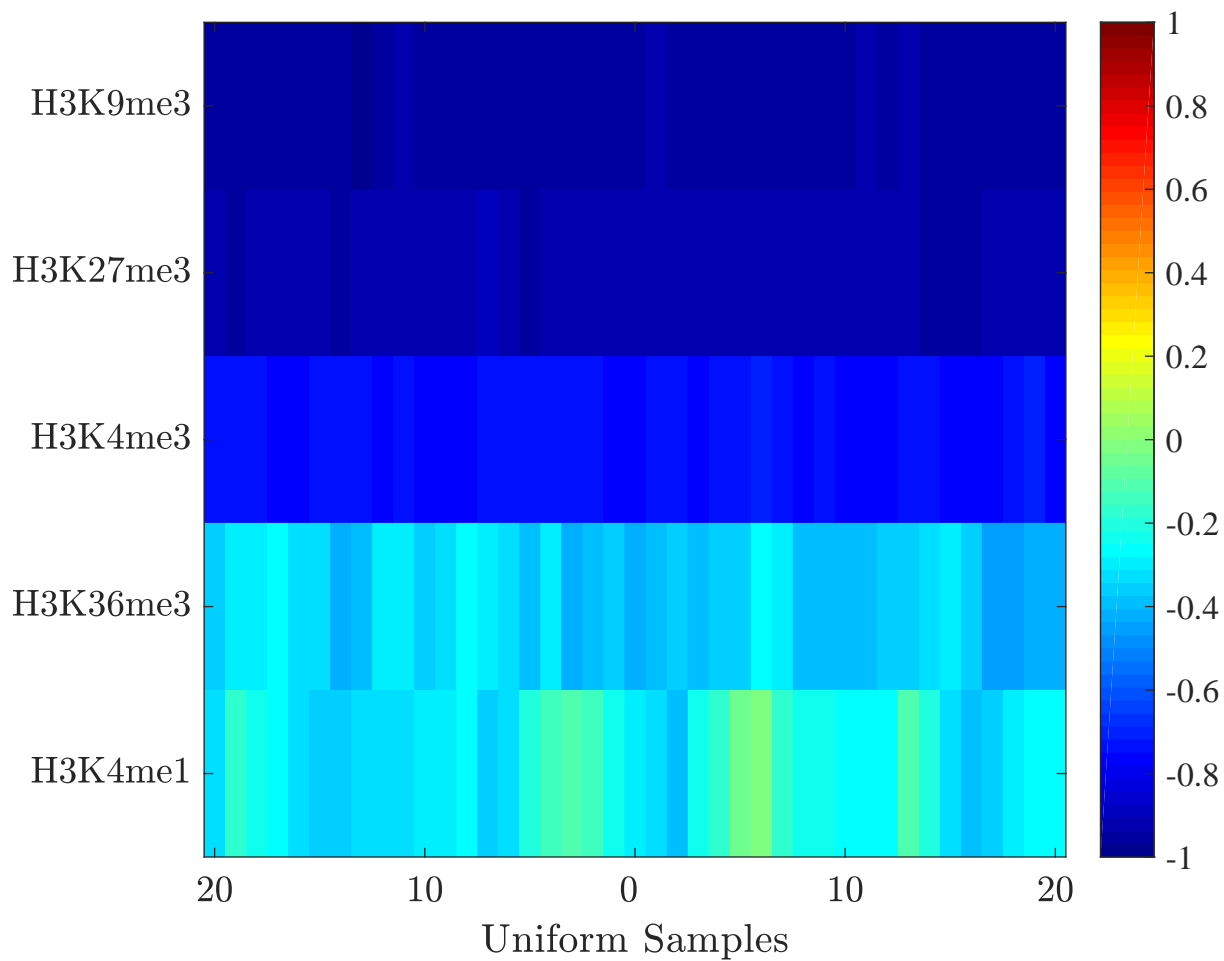

Supplement: Supplementary file 4 — HebbPlots of low-CpG promoters. This compressed file (.tar.gz) includes HebbPlots of low-CpG promoters active in 57 tissues/cell types. (TAR 2971 kb) [file 12859_2018_2312_MOESM4_ESM.tar › file5/E082.pdf]

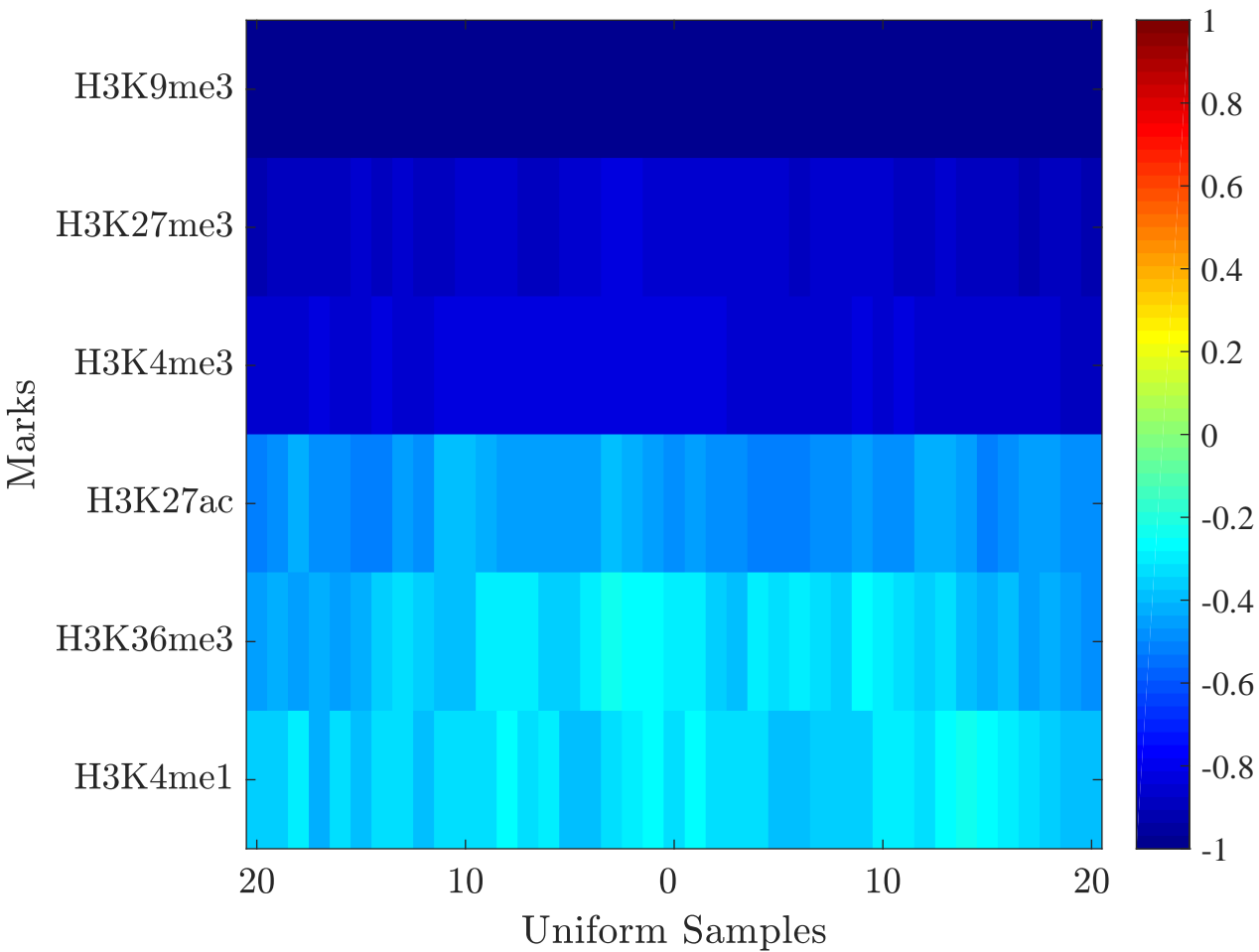

Supplement: Supplementary file 4 — HebbPlots of low-CpG promoters. This compressed file (.tar.gz) includes HebbPlots of low-CpG promoters active in 57 tissues/cell types. (TAR 2971 kb) [file 12859_2018_2312_MOESM4_ESM.tar › file5/E084.pdf]

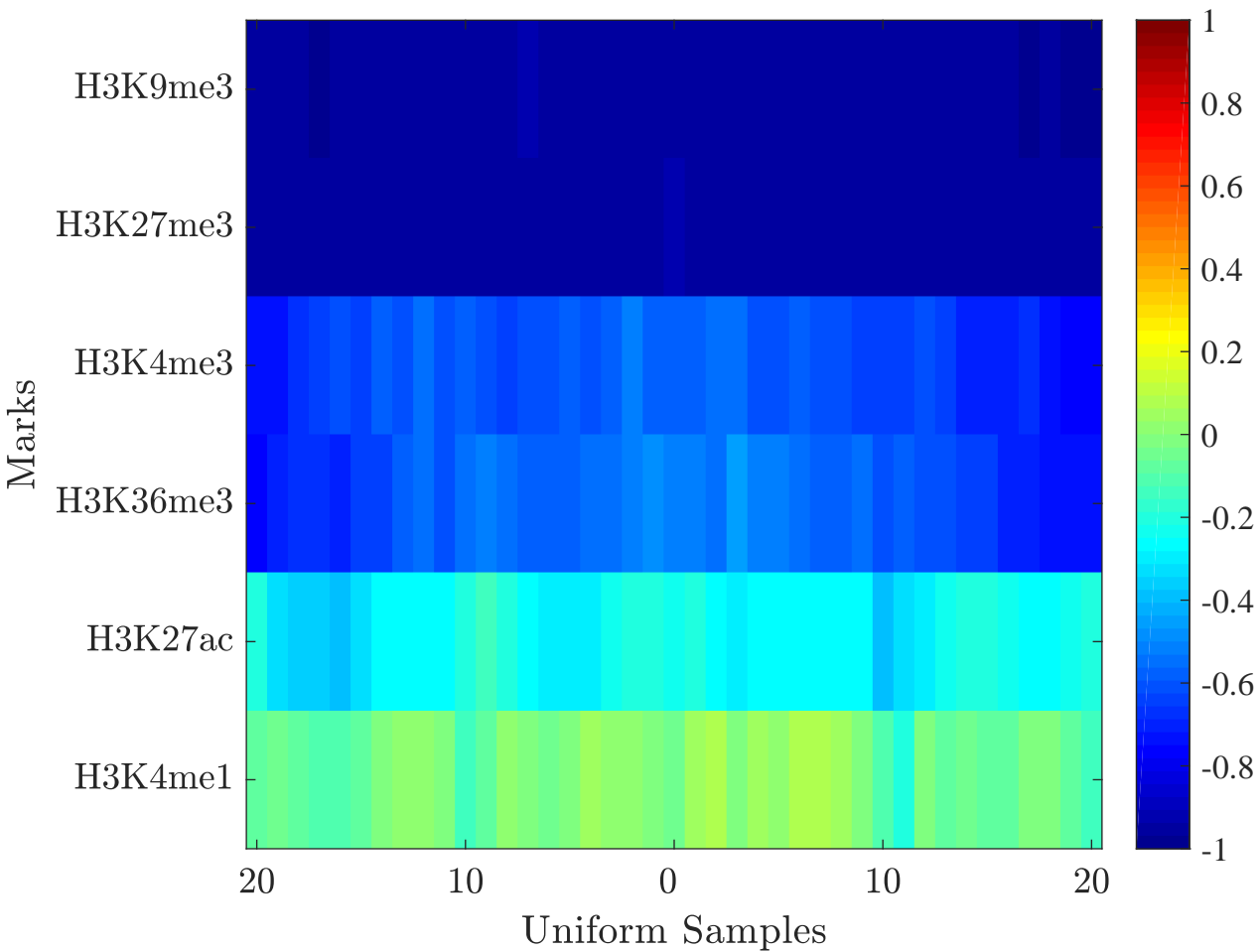

Supplement: Supplementary file 4 — HebbPlots of low-CpG promoters. This compressed file (.tar.gz) includes HebbPlots of low-CpG promoters active in 57 tissues/cell types. (TAR 2971 kb) [file 12859_2018_2312_MOESM4_ESM.tar › file5/E085.pdf]

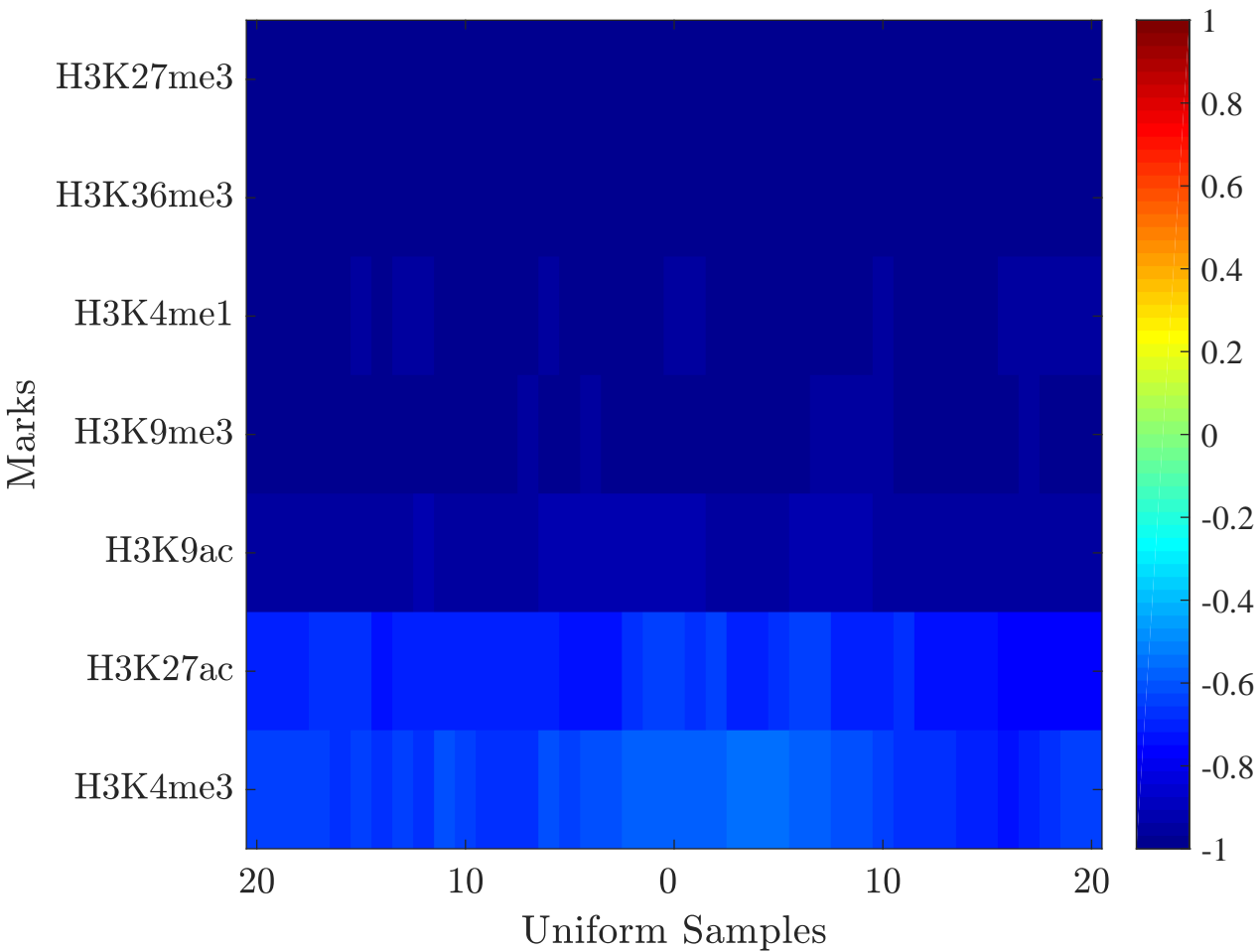

Supplement: Supplementary file 4 — HebbPlots of low-CpG promoters. This compressed file (.tar.gz) includes HebbPlots of low-CpG promoters active in 57 tissues/cell types. (TAR 2971 kb) [file 12859_2018_2312_MOESM4_ESM.tar › file5/E087.pdf]

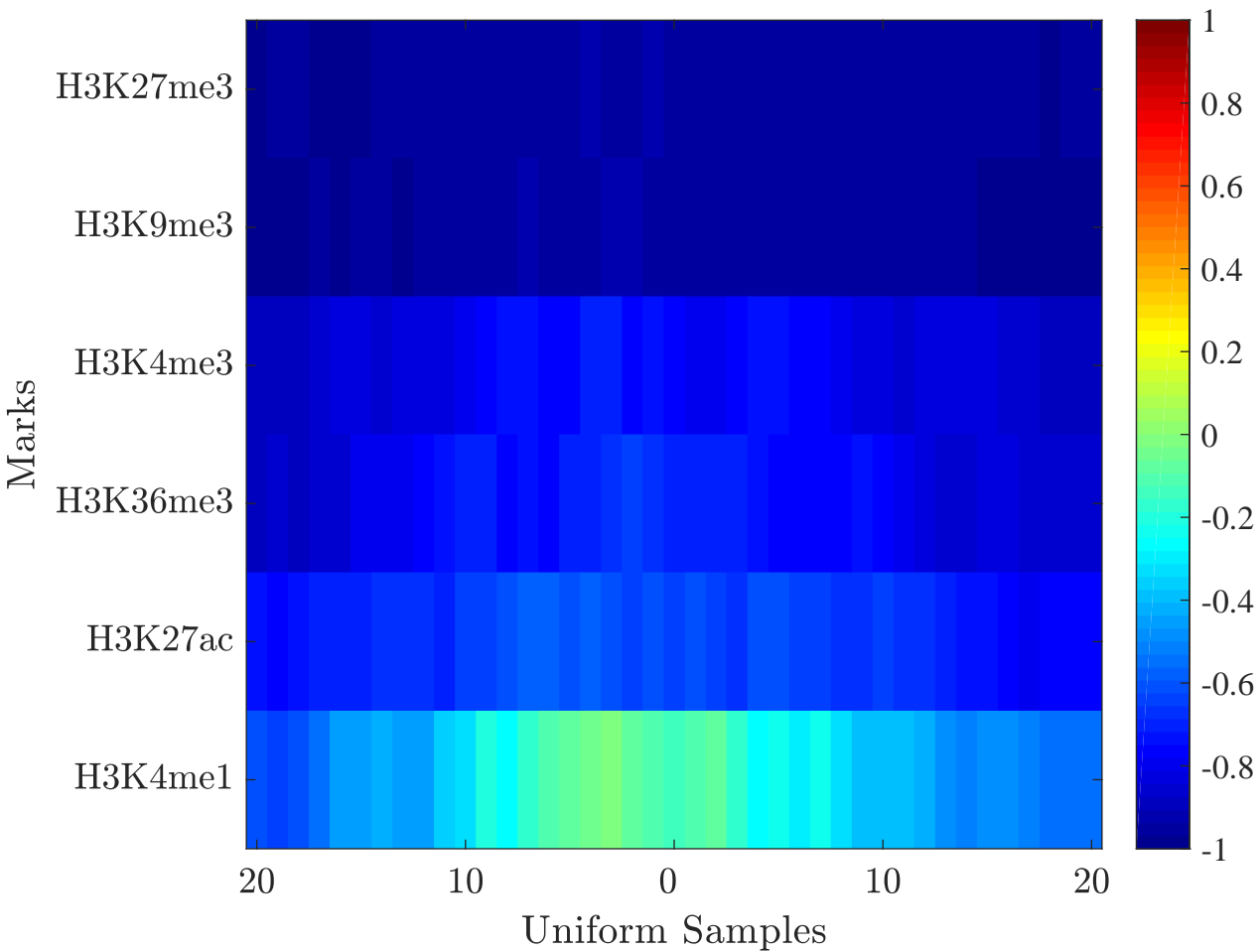

Supplement: Supplementary file 4 — HebbPlots of low-CpG promoters. This compressed file (.tar.gz) includes HebbPlots of low-CpG promoters active in 57 tissues/cell types. (TAR 2971 kb) [file 12859_2018_2312_MOESM4_ESM.tar › file5/E094.pdf]

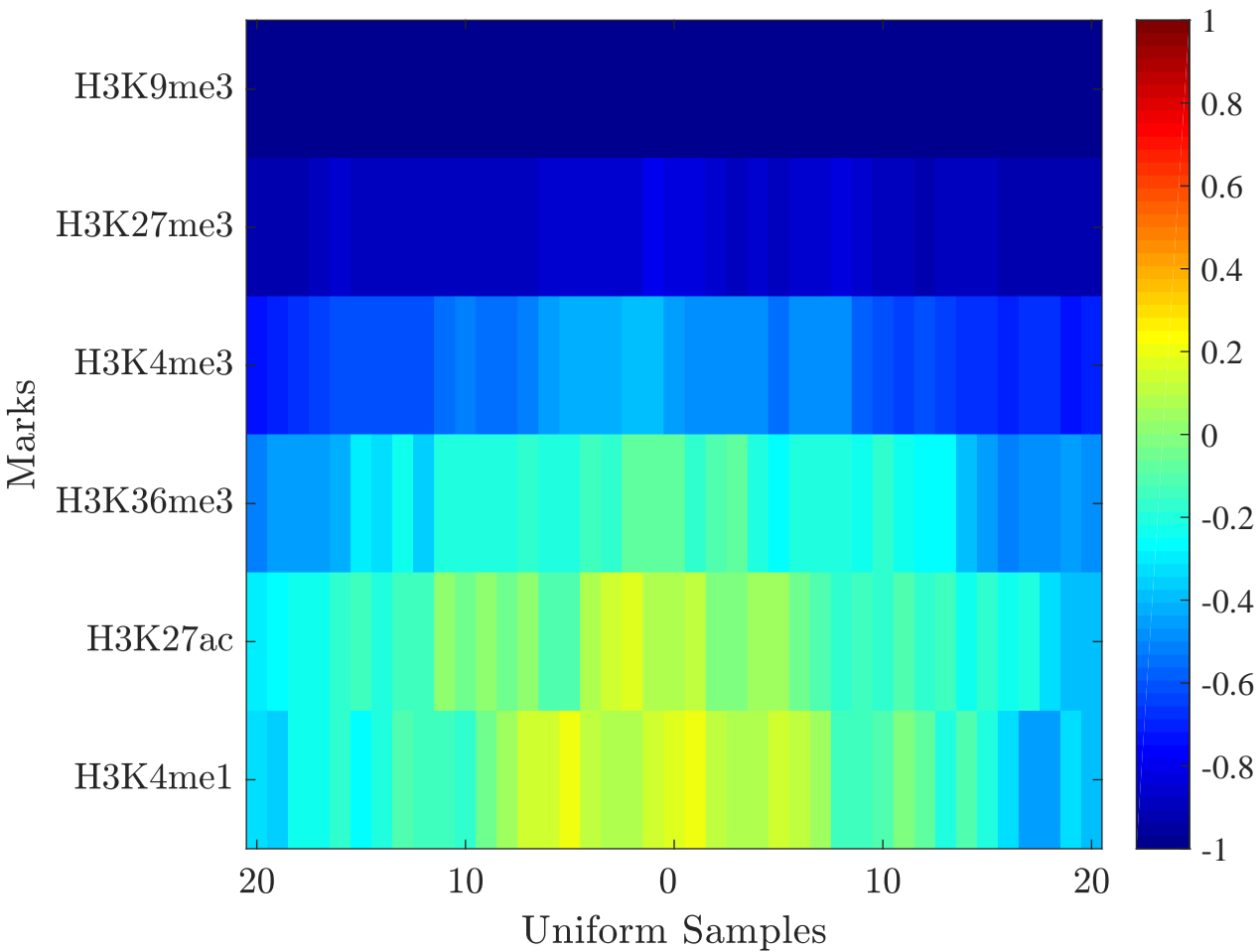

Supplement: Supplementary file 4 — HebbPlots of low-CpG promoters. This compressed file (.tar.gz) includes HebbPlots of low-CpG promoters active in 57 tissues/cell types. (TAR 2971 kb) [file 12859_2018_2312_MOESM4_ESM.tar › file5/E095.pdf]

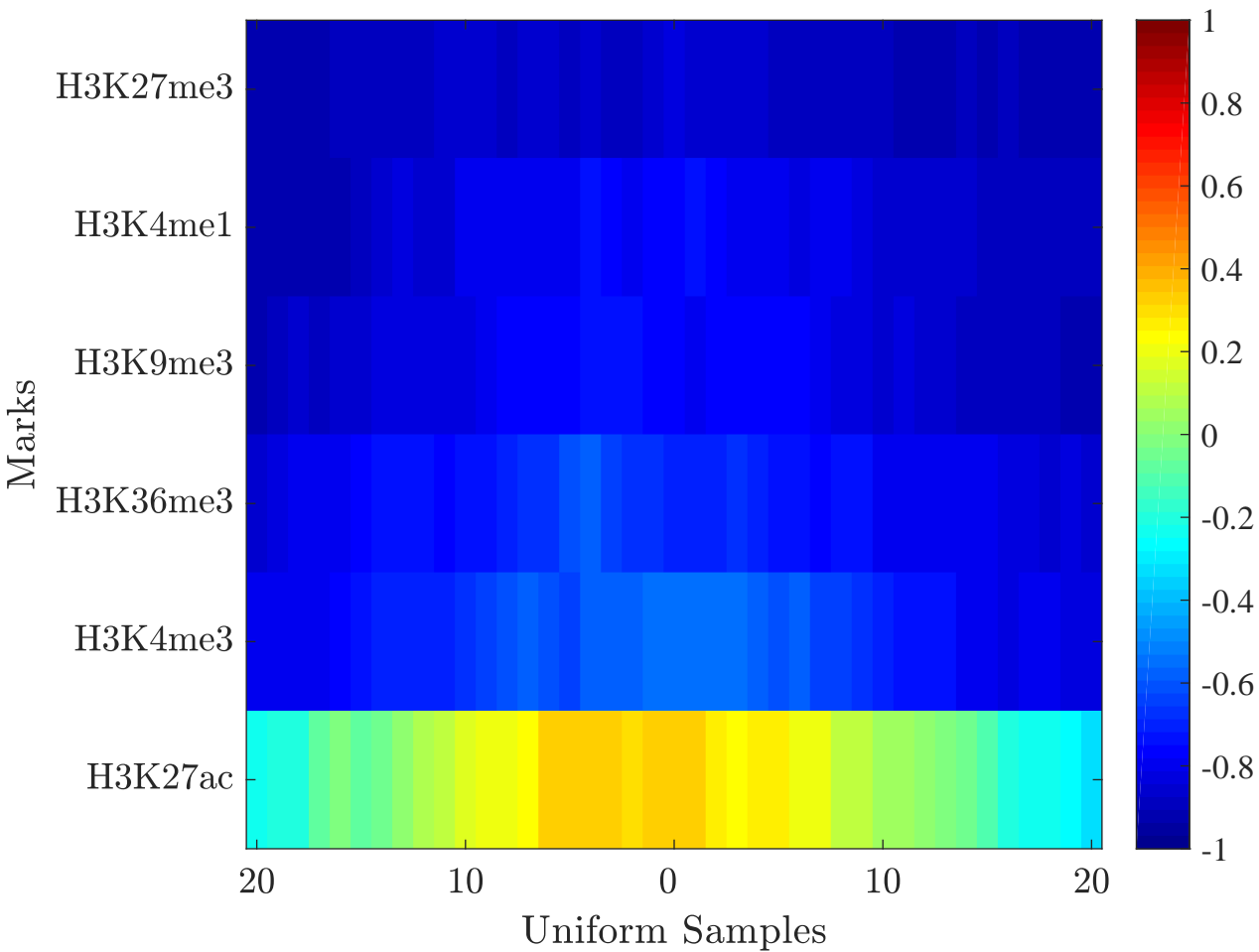

Supplement: Supplementary file 4 — HebbPlots of low-CpG promoters. This compressed file (.tar.gz) includes HebbPlots of low-CpG promoters active in 57 tissues/cell types. (TAR 2971 kb) [file 12859_2018_2312_MOESM4_ESM.tar › file5/E096.pdf]

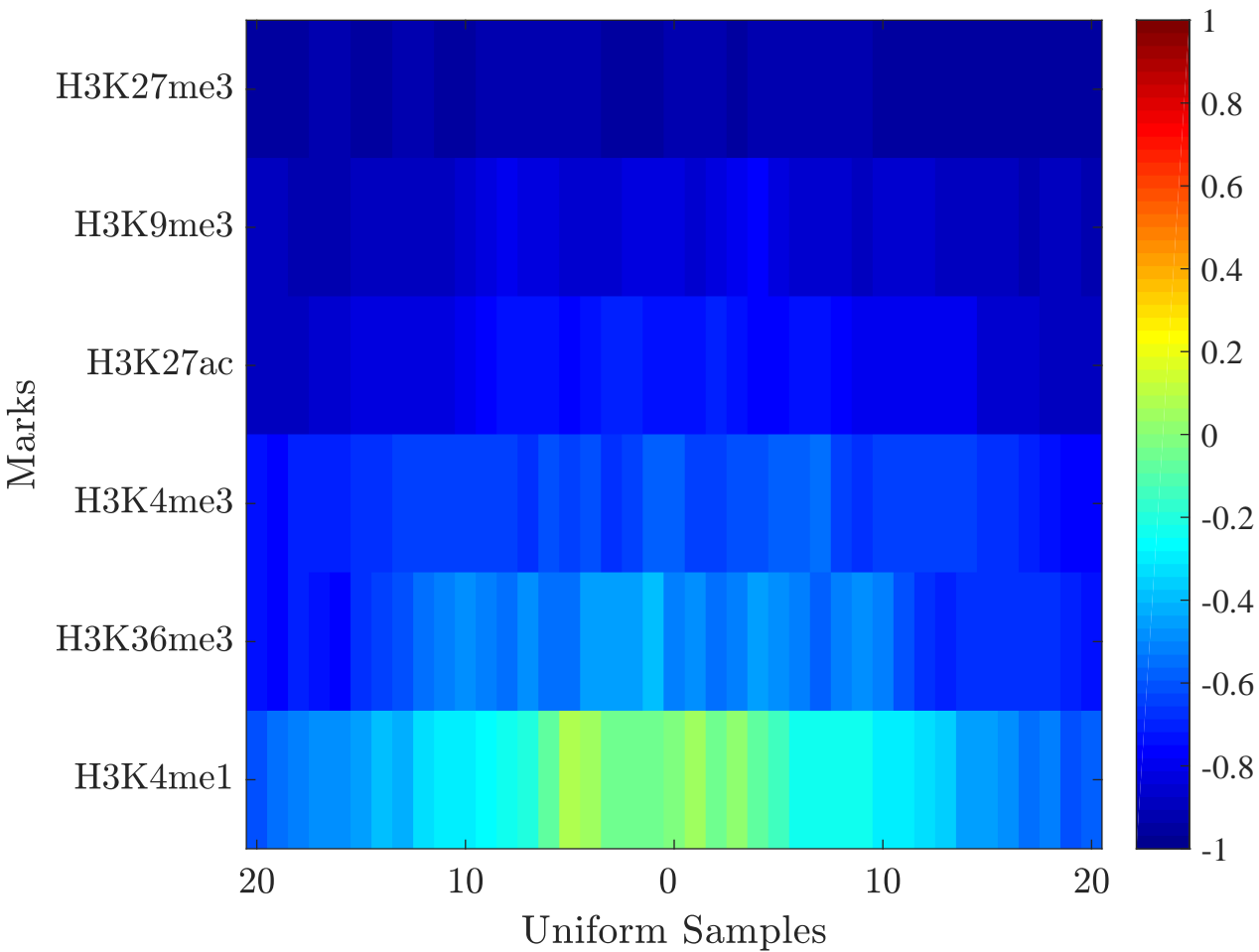

Supplement: Supplementary file 4 — HebbPlots of low-CpG promoters. This compressed file (.tar.gz) includes HebbPlots of low-CpG promoters active in 57 tissues/cell types. (TAR 2971 kb) [file 12859_2018_2312_MOESM4_ESM.tar › file5/E097.pdf]

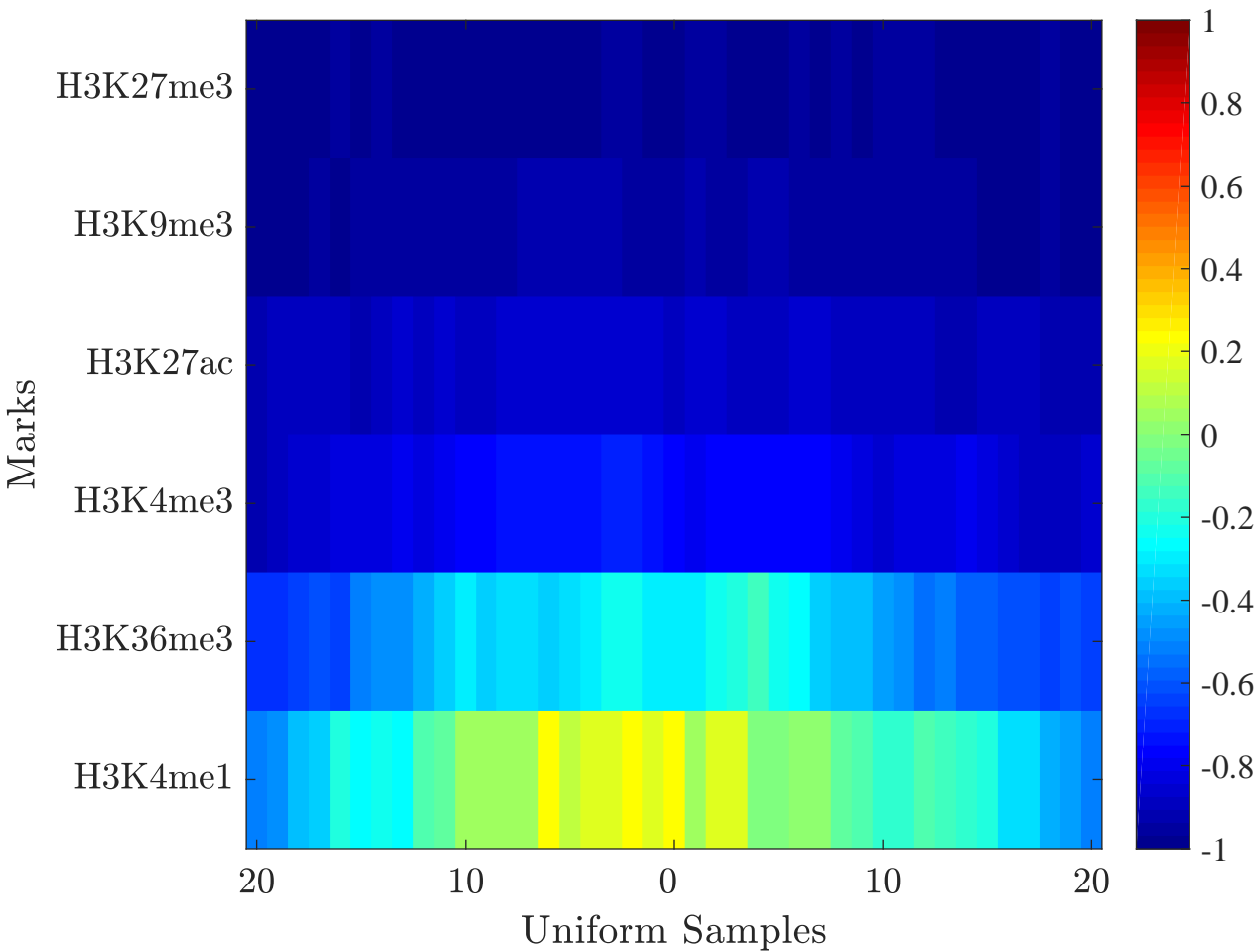

Supplement: Supplementary file 4 — HebbPlots of low-CpG promoters. This compressed file (.tar.gz) includes HebbPlots of low-CpG promoters active in 57 tissues/cell types. (TAR 2971 kb) [file 12859_2018_2312_MOESM4_ESM.tar › file5/E098.pdf]

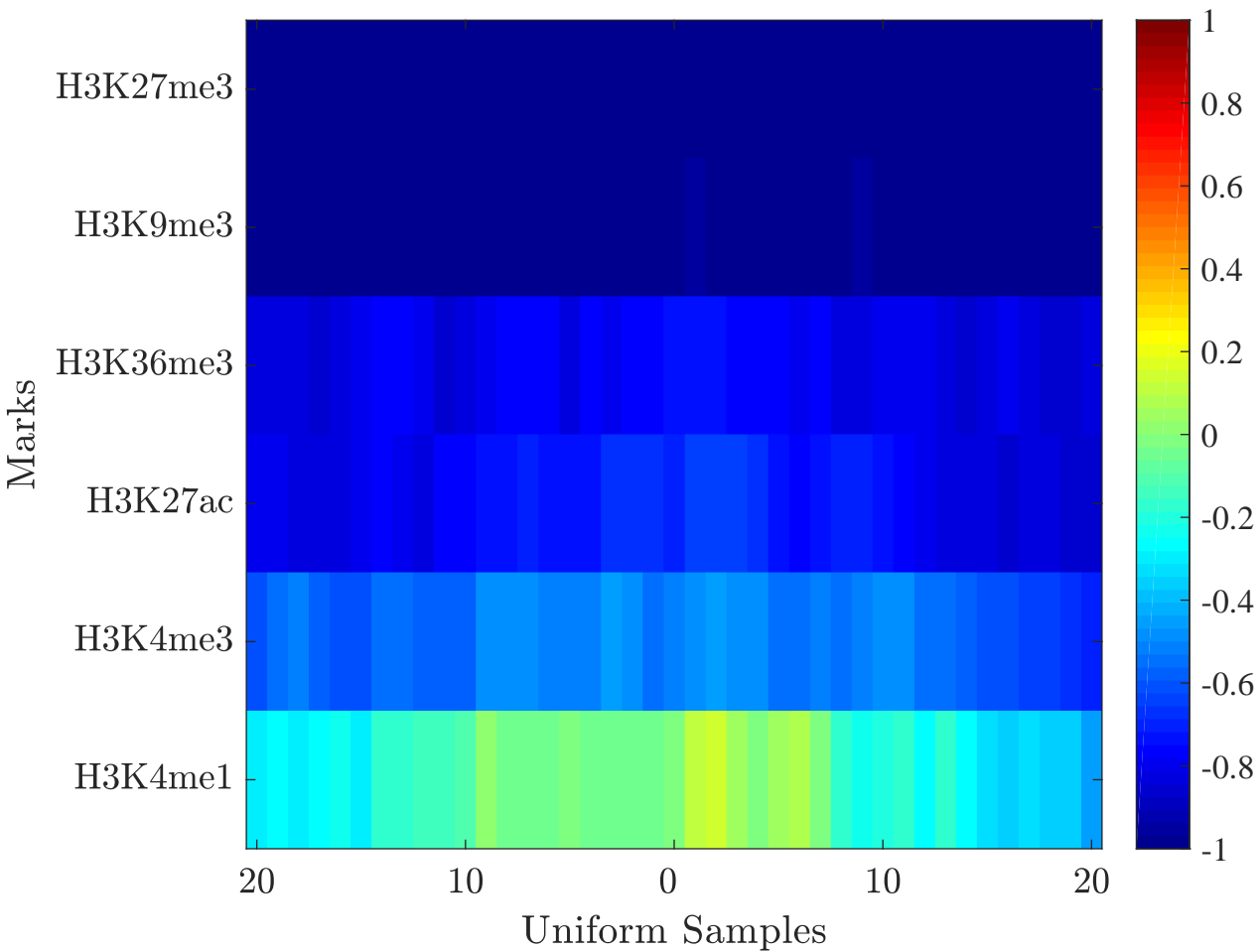

Supplement: Supplementary file 4 — HebbPlots of low-CpG promoters. This compressed file (.tar.gz) includes HebbPlots of low-CpG promoters active in 57 tissues/cell types. (TAR 2971 kb) [file 12859_2018_2312_MOESM4_ESM.tar › file5/E100.pdf]

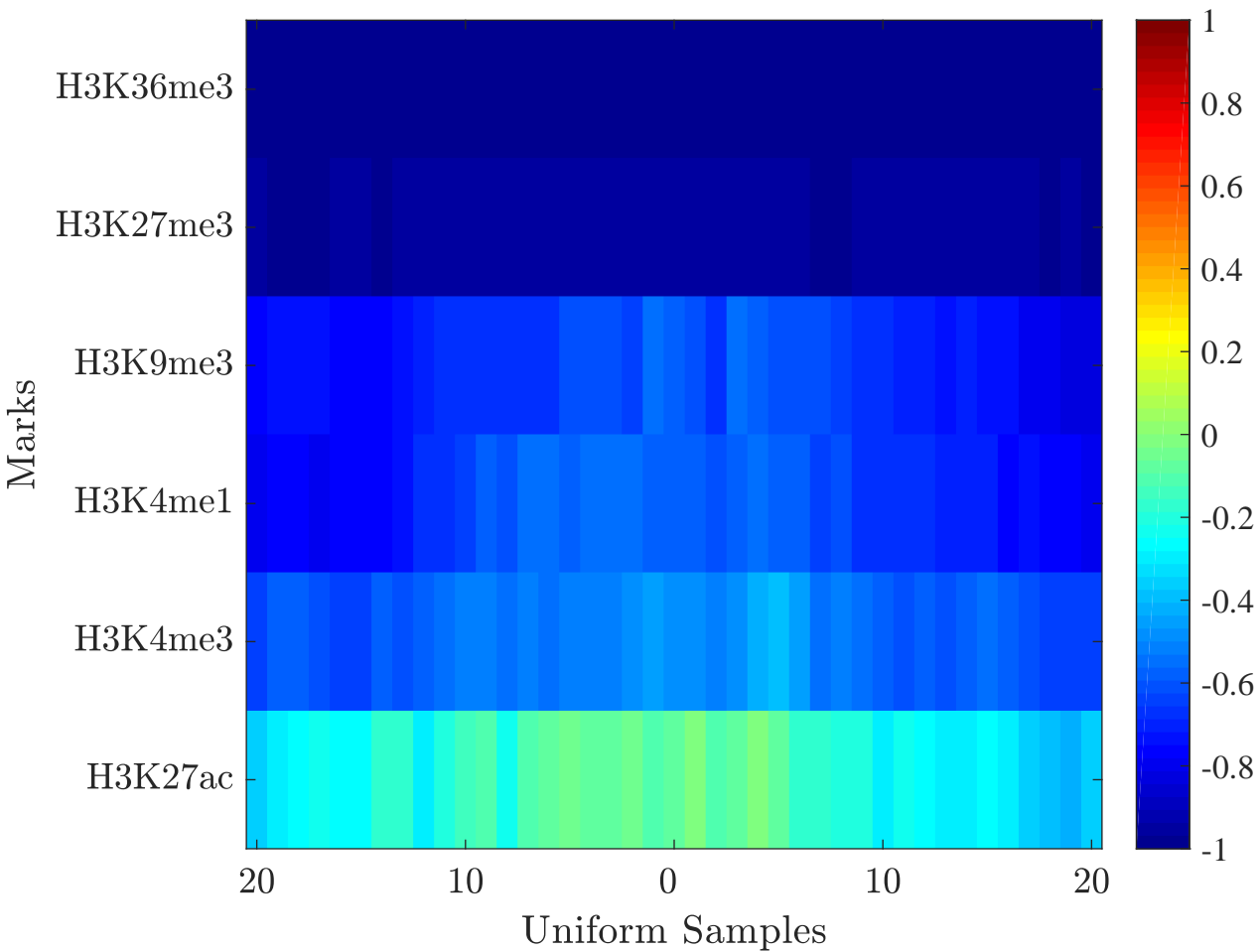

Supplement: Supplementary file 4 — HebbPlots of low-CpG promoters. This compressed file (.tar.gz) includes HebbPlots of low-CpG promoters active in 57 tissues/cell types. (TAR 2971 kb) [file 12859_2018_2312_MOESM4_ESM.tar › file5/E104.pdf]

Marks

H3K27me3

H3K9me3

H3K27ac

H3K4me3

H3K36me3

H3K4me1

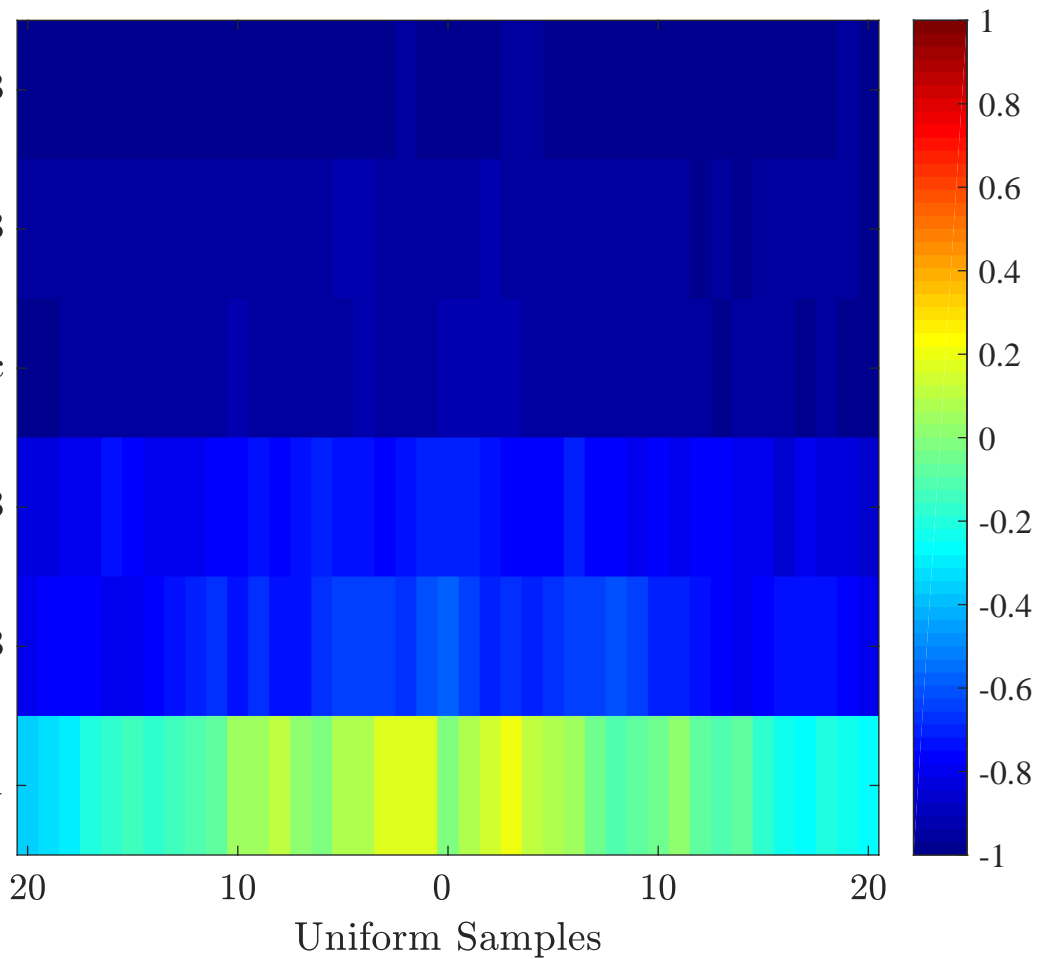

Supplement: Supplementary file 4 — HebbPlots of low-CpG promoters. This compressed file (.tar.gz) includes HebbPlots of low-CpG promoters active in 57 tissues/cell types. (TAR 2971 kb) [file 12859_2018_2312_MOESM4_ESM.tar › file5/E105.pdf]

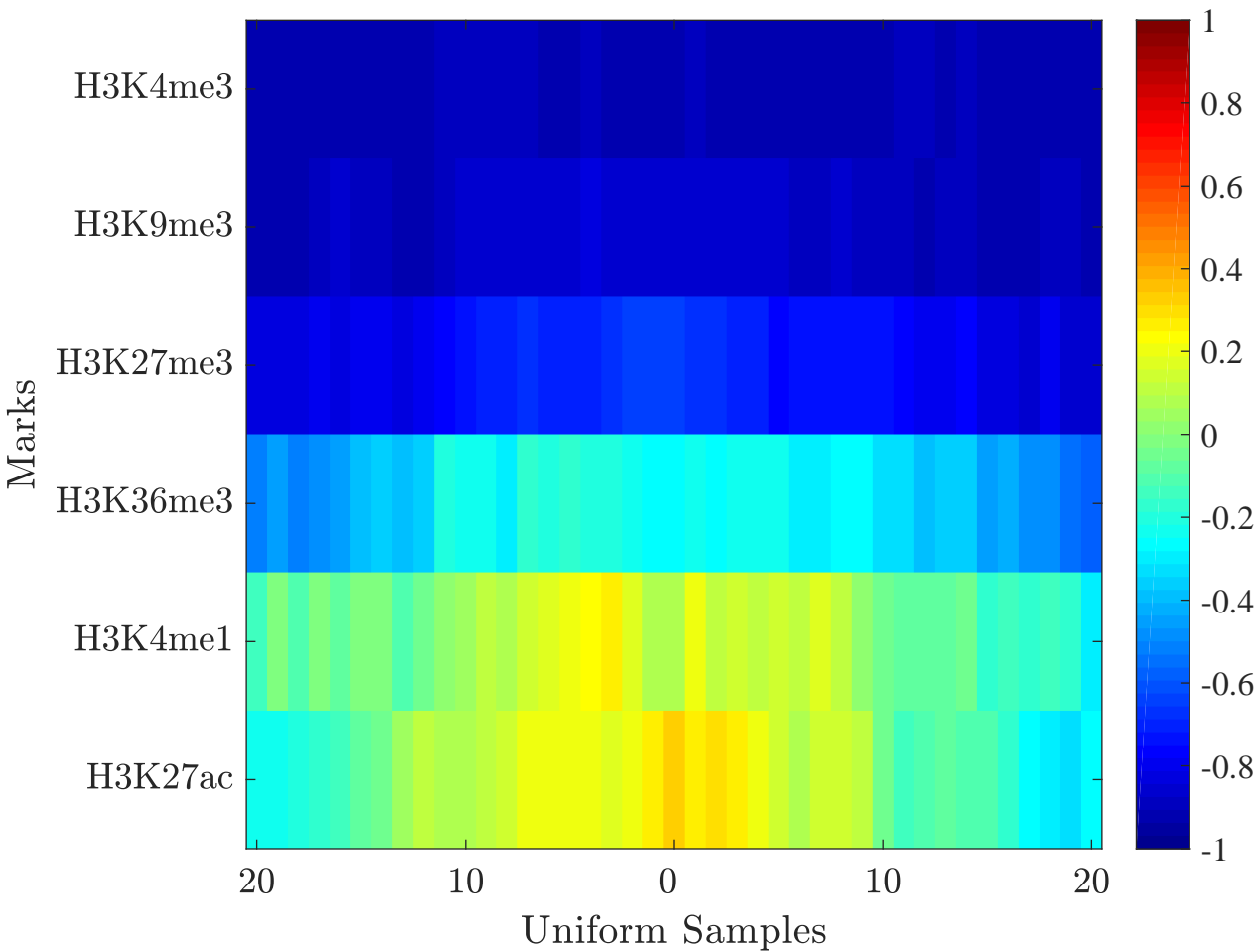

Supplement: Supplementary file 4 — HebbPlots of low-CpG promoters. This compressed file (.tar.gz) includes HebbPlots of low-CpG promoters active in 57 tissues/cell types. (TAR 2971 kb) [file 12859_2018_2312_MOESM4_ESM.tar › file5/E106.pdf]

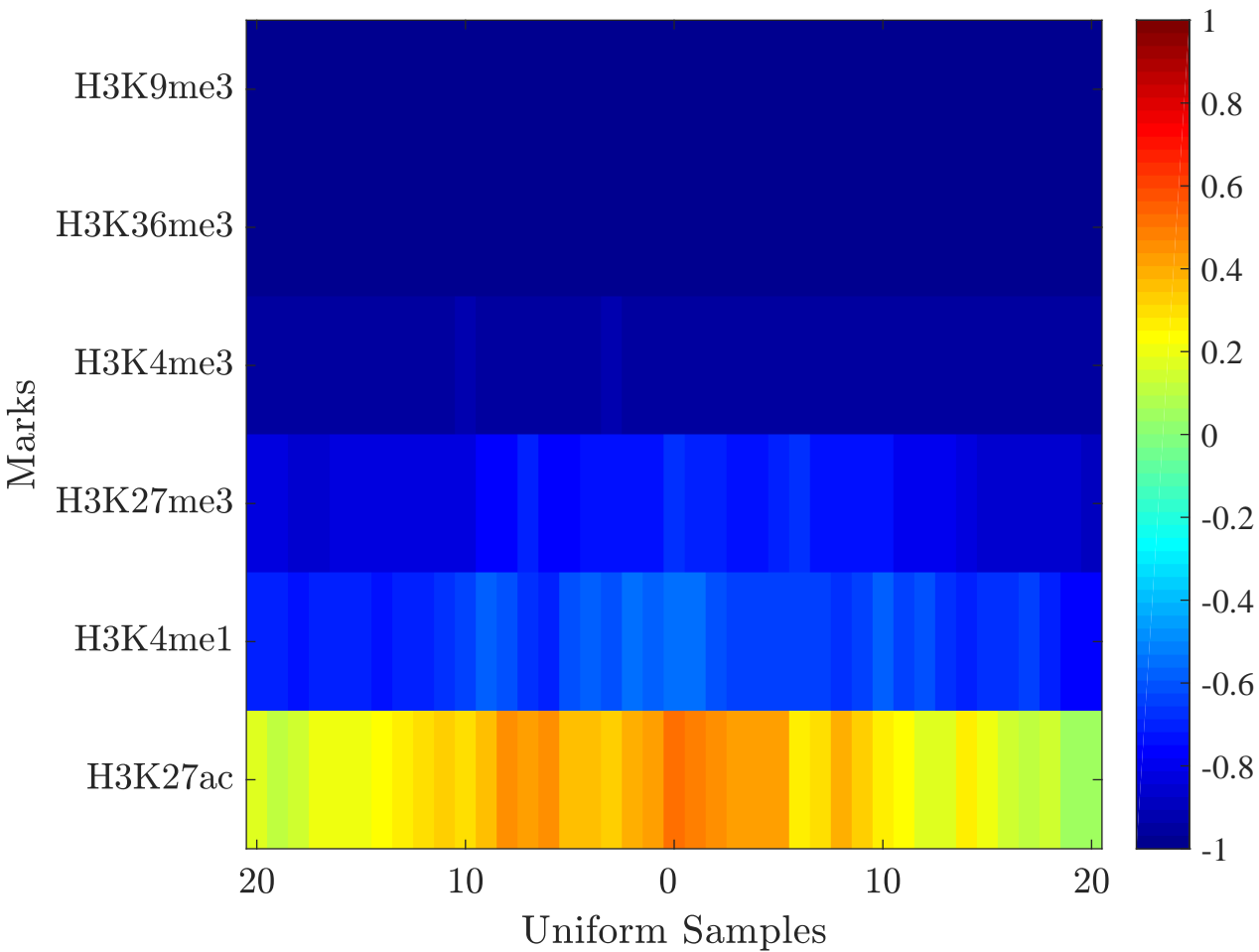

Supplement: Supplementary file 4 — HebbPlots of low-CpG promoters. This compressed file (.tar.gz) includes HebbPlots of low-CpG promoters active in 57 tissues/cell types. (TAR 2971 kb) [file 12859_2018_2312_MOESM4_ESM.tar › file5/E109.pdf]

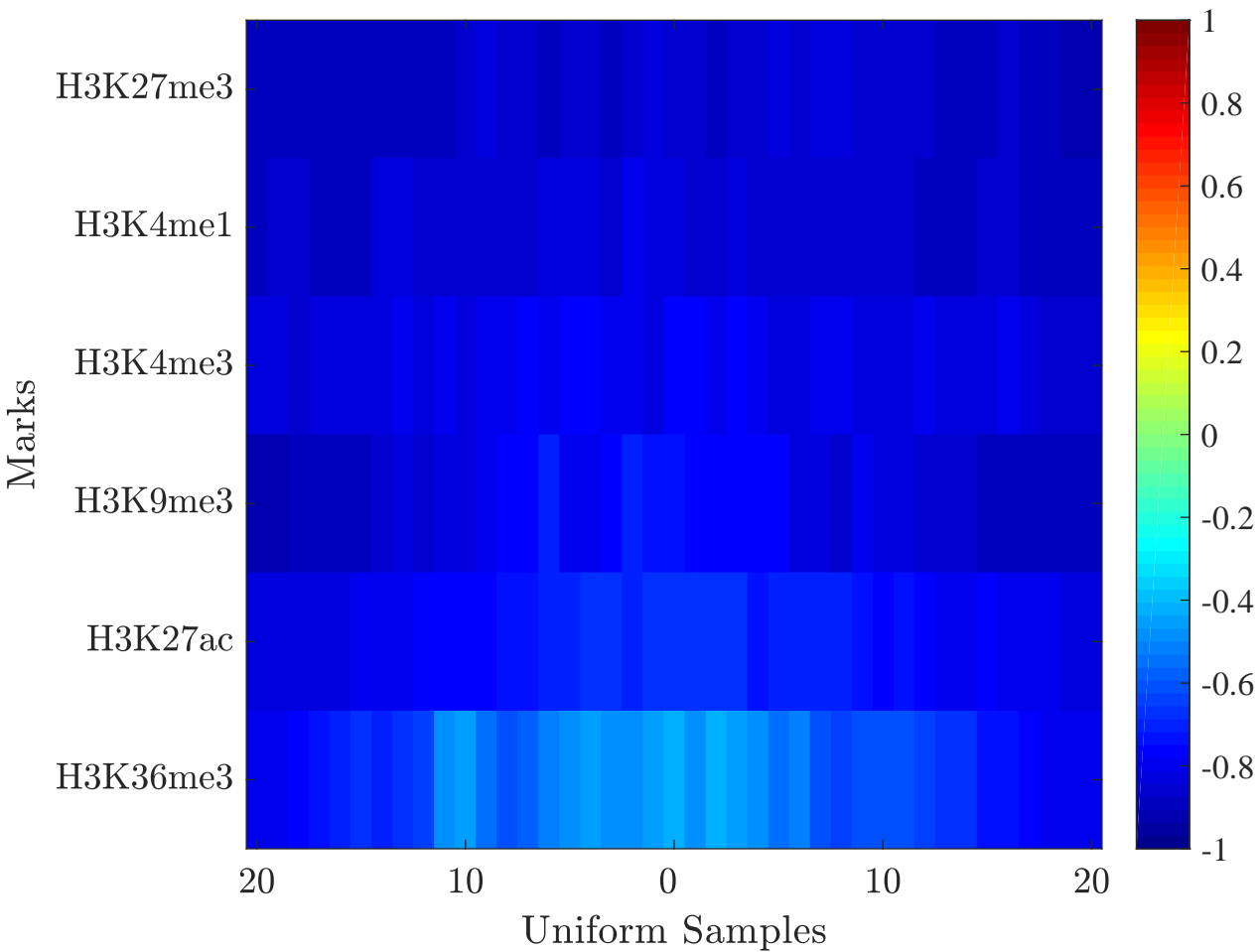

Supplement: Supplementary file 4 — HebbPlots of low-CpG promoters. This compressed file (.tar.gz) includes HebbPlots of low-CpG promoters active in 57 tissues/cell types. (TAR 2971 kb) [file 12859_2018_2312_MOESM4_ESM.tar › file5/E112.pdf]

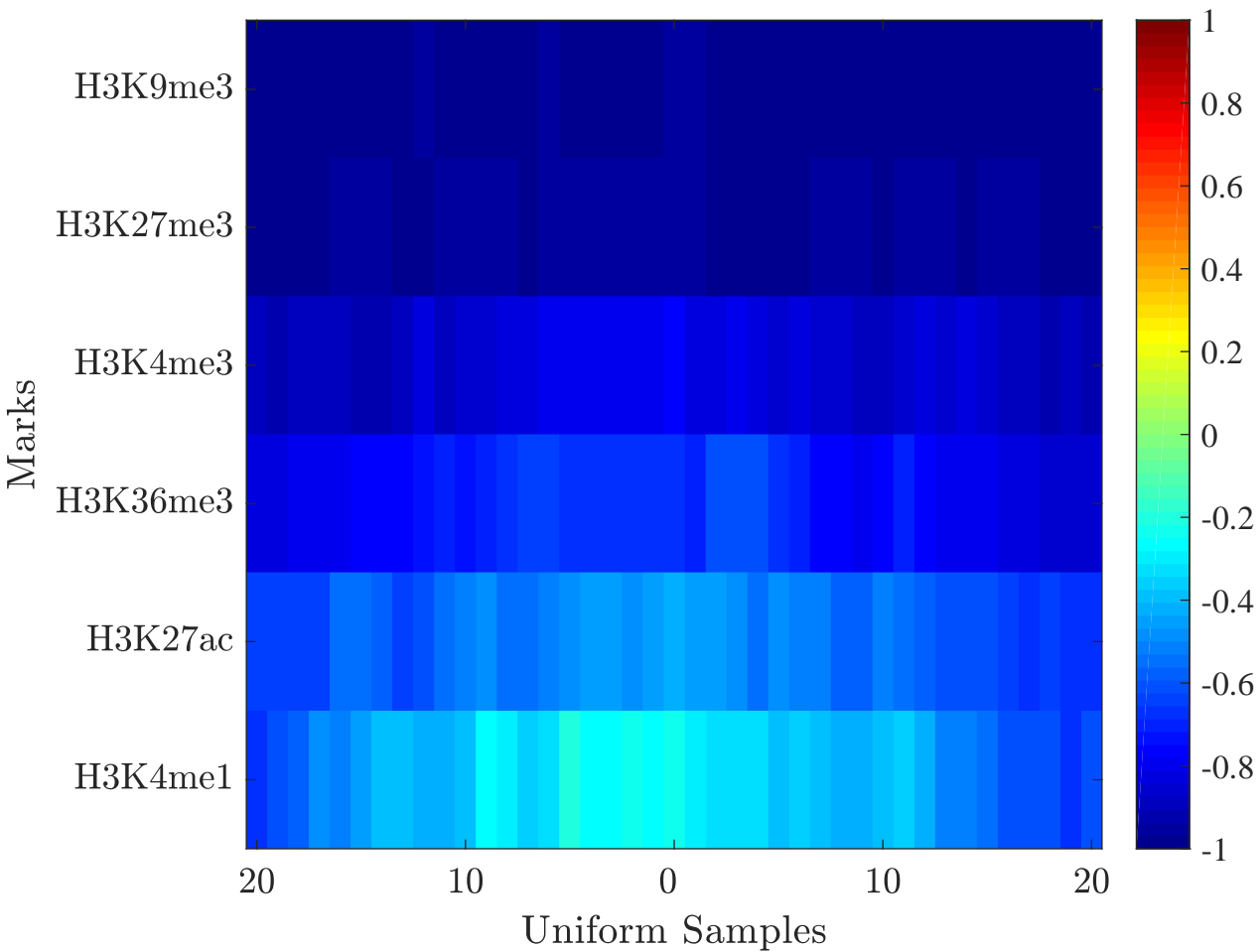

Supplement: Supplementary file 4 — HebbPlots of low-CpG promoters. This compressed file (.tar.gz) includes HebbPlots of low-CpG promoters active in 57 tissues/cell types. (TAR 2971 kb) [file 12859_2018_2312_MOESM4_ESM.tar › file5/E113.pdf]

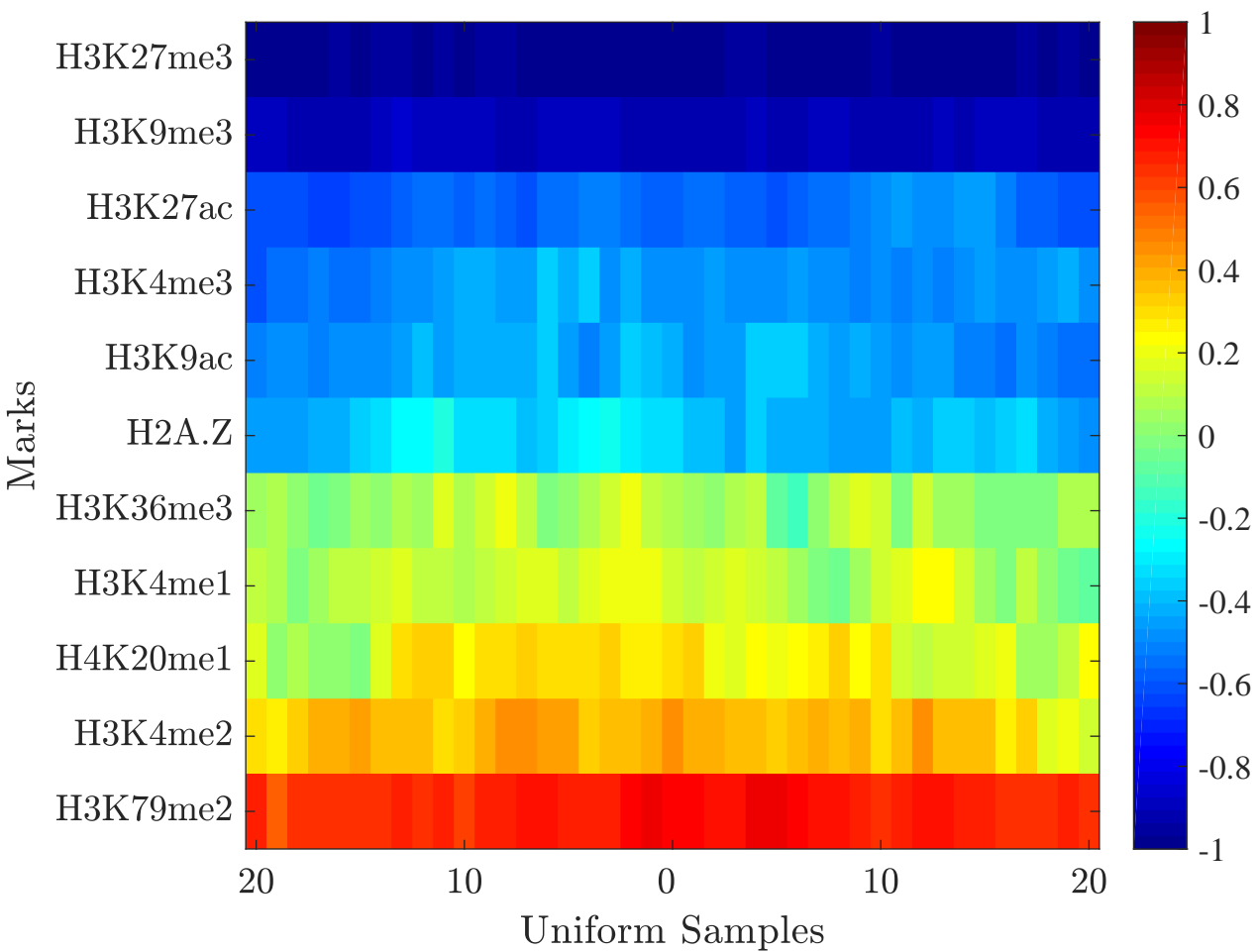

Supplement: Supplementary file 4 — HebbPlots of low-CpG promoters. This compressed file (.tar.gz) includes HebbPlots of low-CpG promoters active in 57 tissues/cell types. (TAR 2971 kb) [file 12859_2018_2312_MOESM4_ESM.tar › file5/E114.pdf]

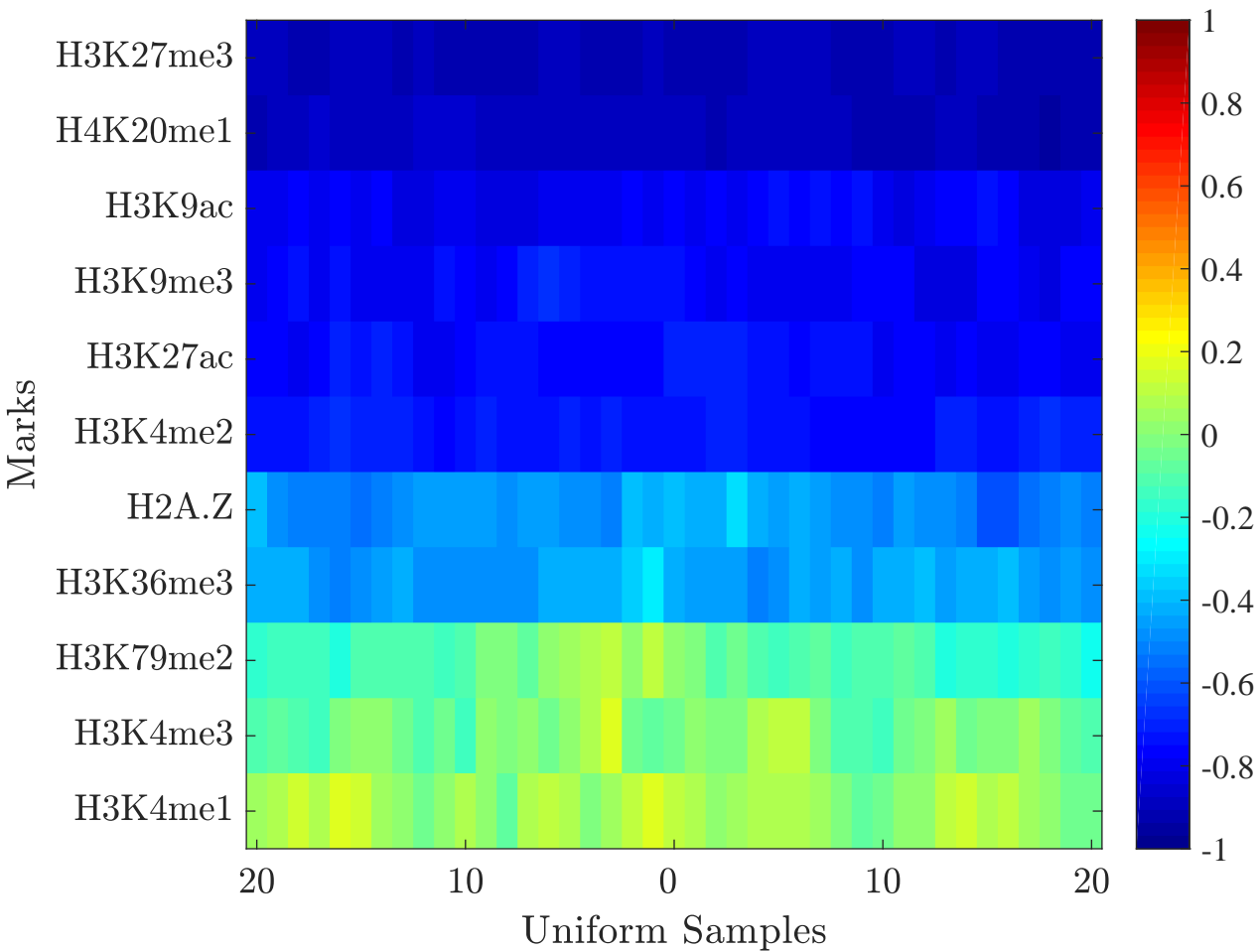

Supplement: Supplementary file 4 — HebbPlots of low-CpG promoters. This compressed file (.tar.gz) includes HebbPlots of low-CpG promoters active in 57 tissues/cell types. (TAR 2971 kb) [file 12859_2018_2312_MOESM4_ESM.tar › file5/E116.pdf]

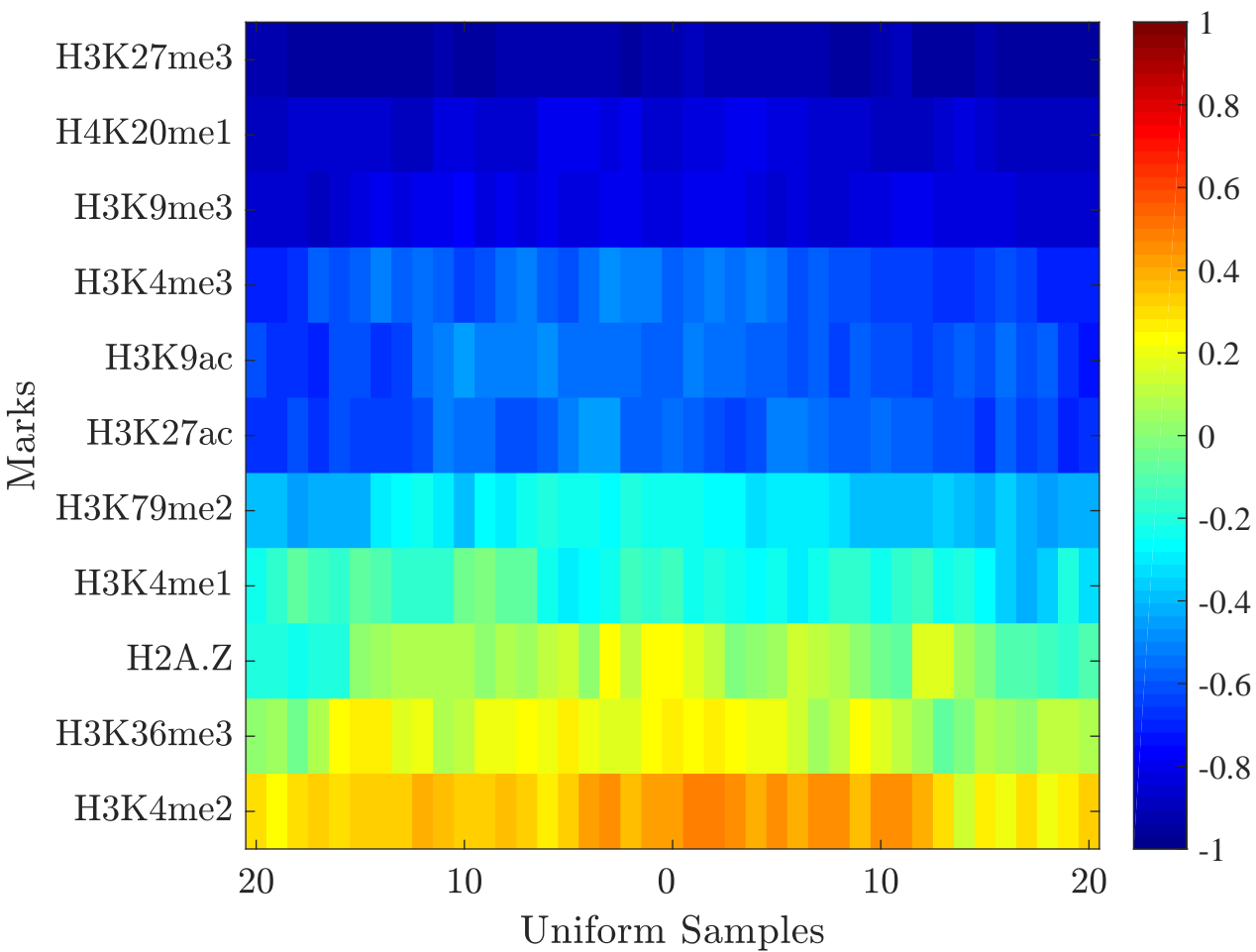

Supplement: Supplementary file 4 — HebbPlots of low-CpG promoters. This compressed file (.tar.gz) includes HebbPlots of low-CpG promoters active in 57 tissues/cell types. (TAR 2971 kb) [file 12859_2018_2312_MOESM4_ESM.tar › file5/E117.pdf]

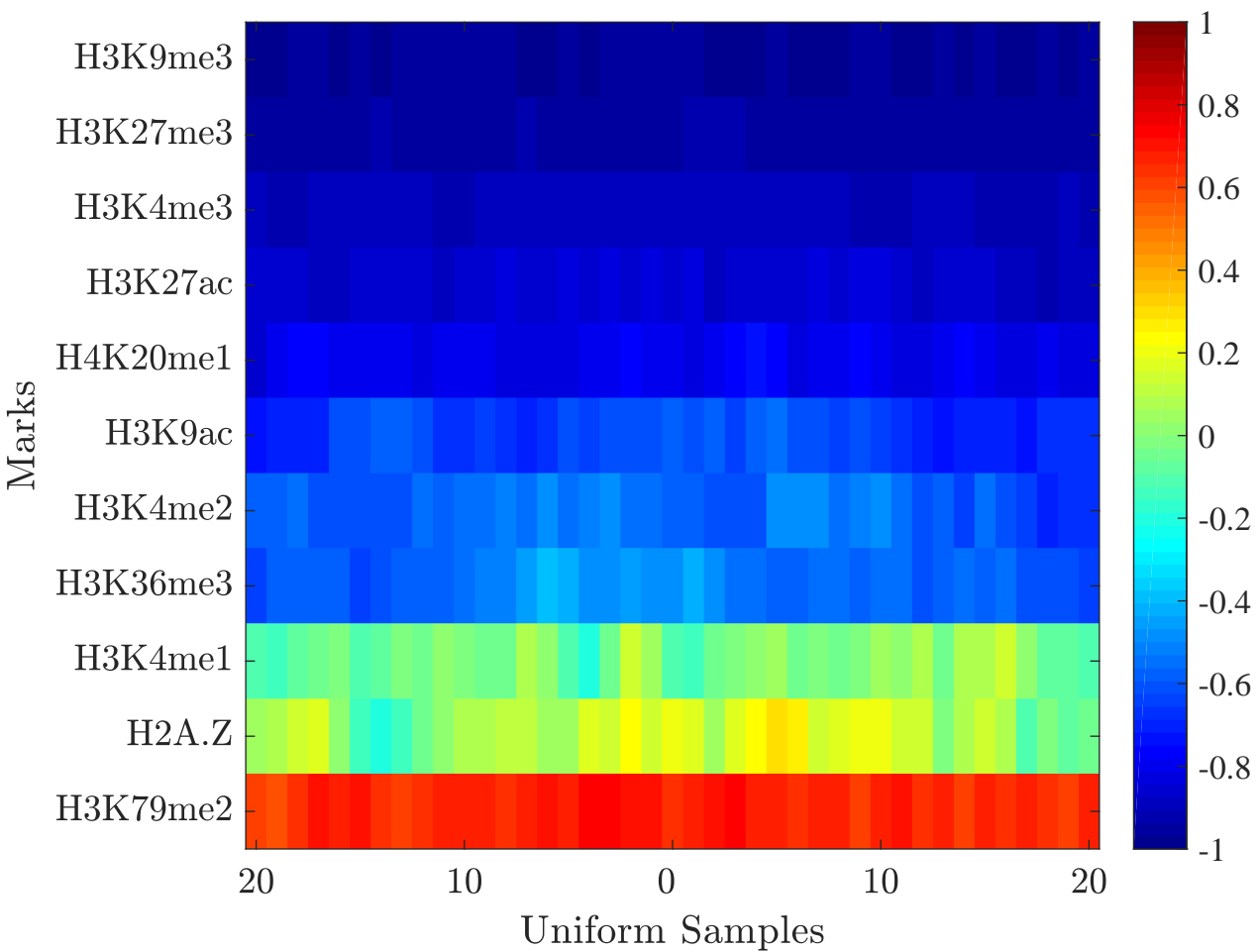

Supplement: Supplementary file 4 — HebbPlots of low-CpG promoters. This compressed file (.tar.gz) includes HebbPlots of low-CpG promoters active in 57 tissues/cell types. (TAR 2971 kb) [file 12859_2018_2312_MOESM4_ESM.tar › file5/E118.pdf]

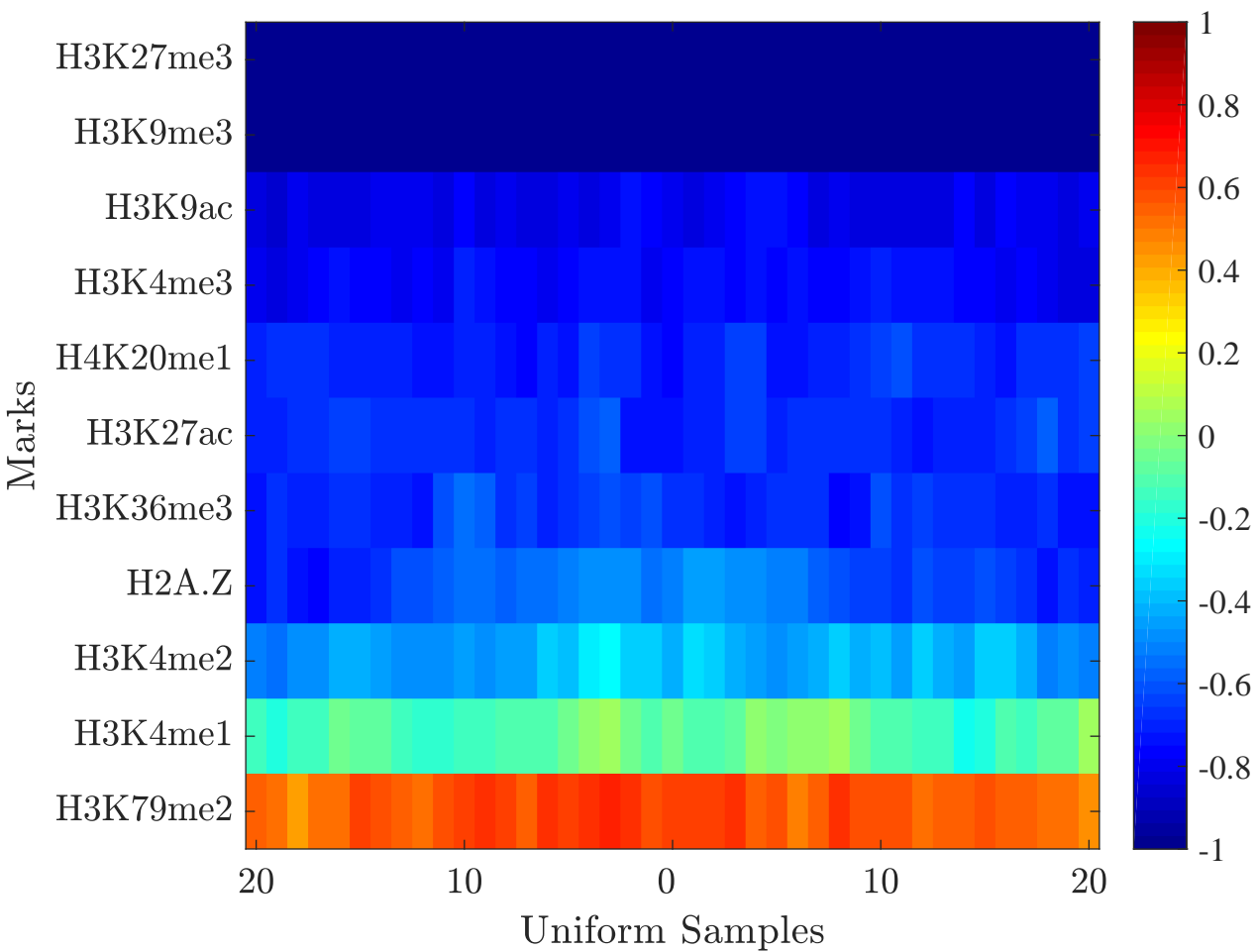

Supplement: Supplementary file 4 — HebbPlots of low-CpG promoters. This compressed file (.tar.gz) includes HebbPlots of low-CpG promoters active in 57 tissues/cell types. (TAR 2971 kb) [file 12859_2018_2312_MOESM4_ESM.tar › file5/E119.pdf]

Marks

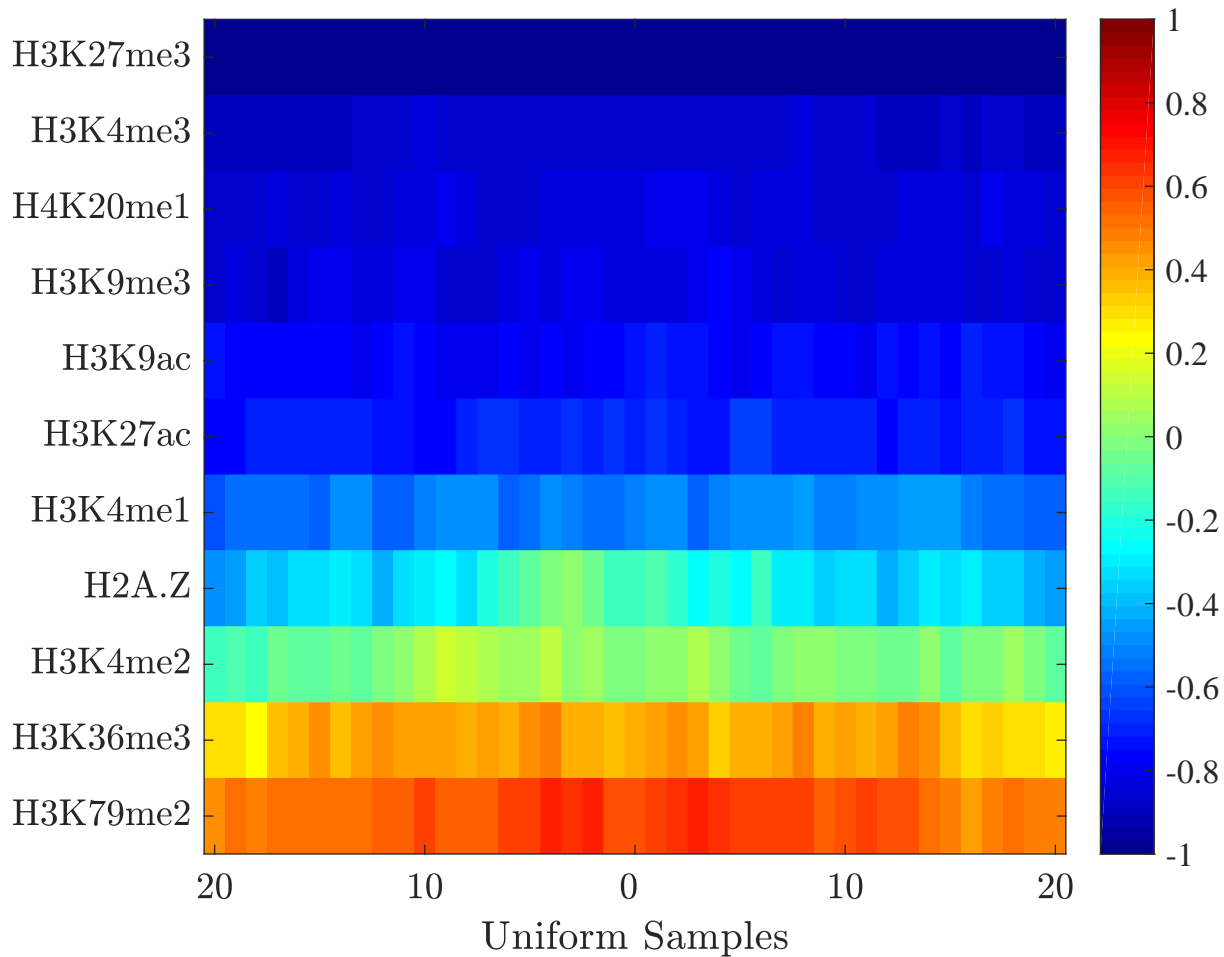

Supplement: Supplementary file 4 — HebbPlots of low-CpG promoters. This compressed file (.tar.gz) includes HebbPlots of low-CpG promoters active in 57 tissues/cell types. (TAR 2971 kb) [file 12859_2018_2312_MOESM4_ESM.tar › file5/E120.pdf]

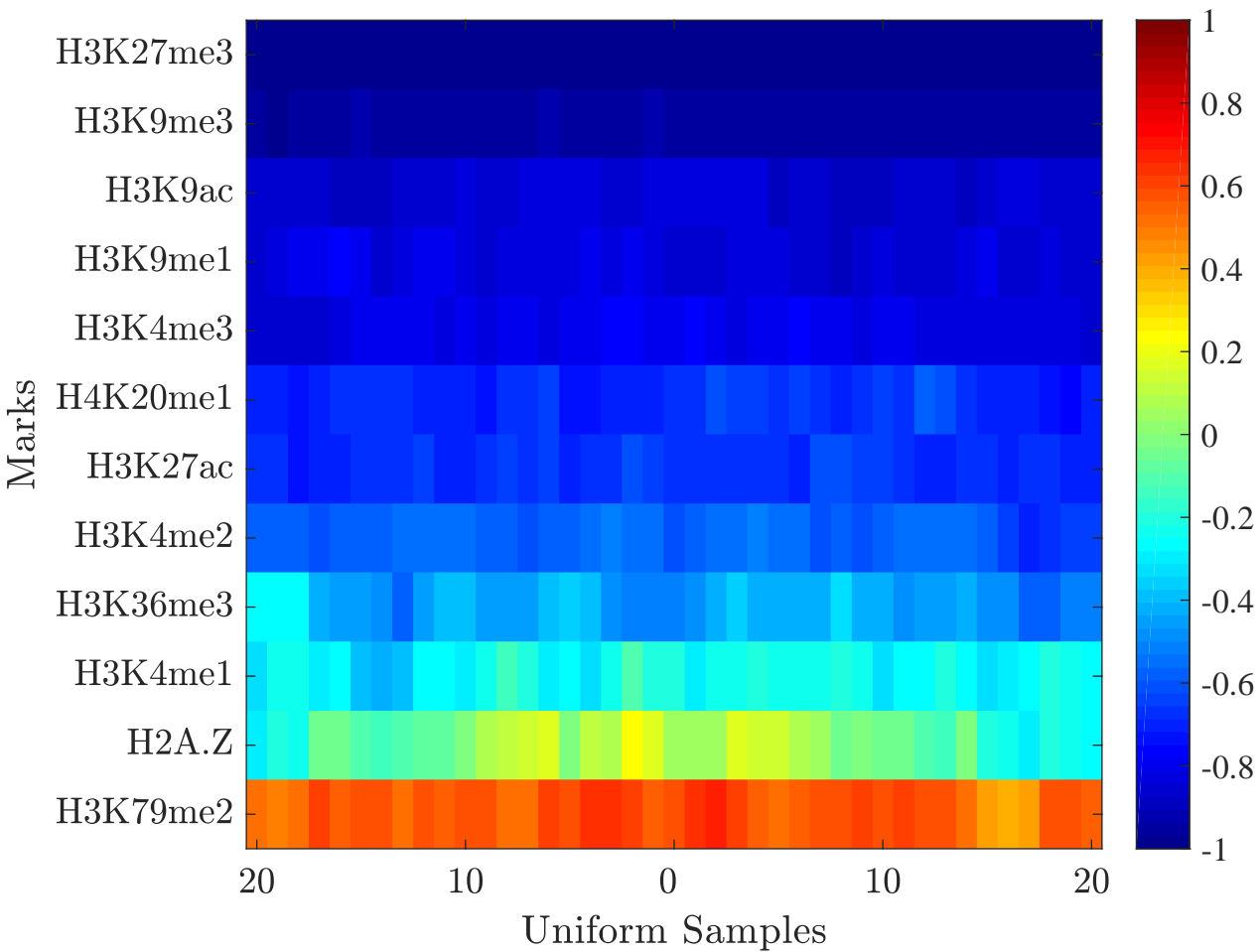

Supplement: Supplementary file 4 — HebbPlots of low-CpG promoters. This compressed file (.tar.gz) includes HebbPlots of low-CpG promoters active in 57 tissues/cell types. (TAR 2971 kb) [file 12859_2018_2312_MOESM4_ESM.tar › file5/E122.pdf]

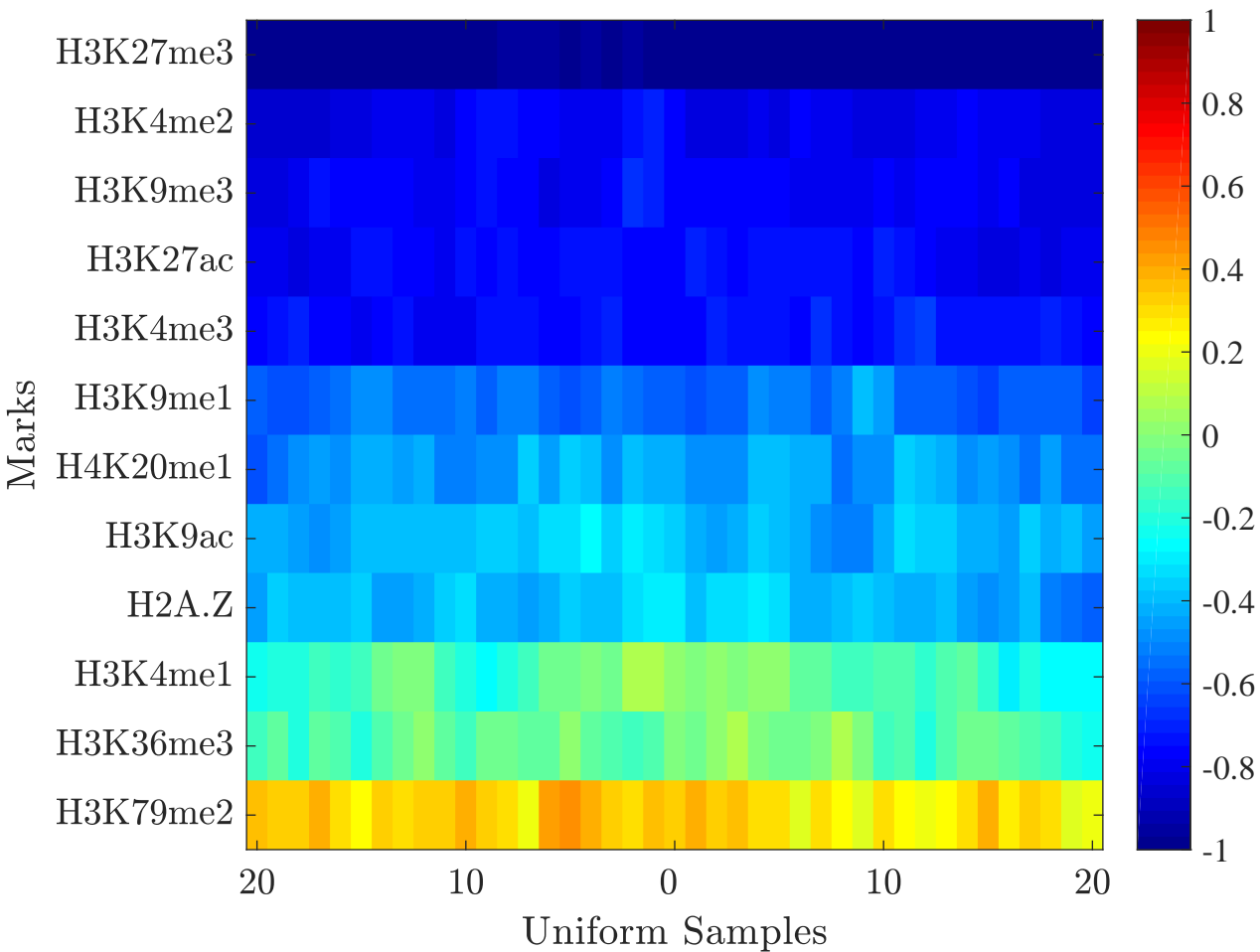

Supplement: Supplementary file 4 — HebbPlots of low-CpG promoters. This compressed file (.tar.gz) includes HebbPlots of low-CpG promoters active in 57 tissues/cell types. (TAR 2971 kb) [file 12859_2018_2312_MOESM4_ESM.tar › file5/E123.pdf]

Marks

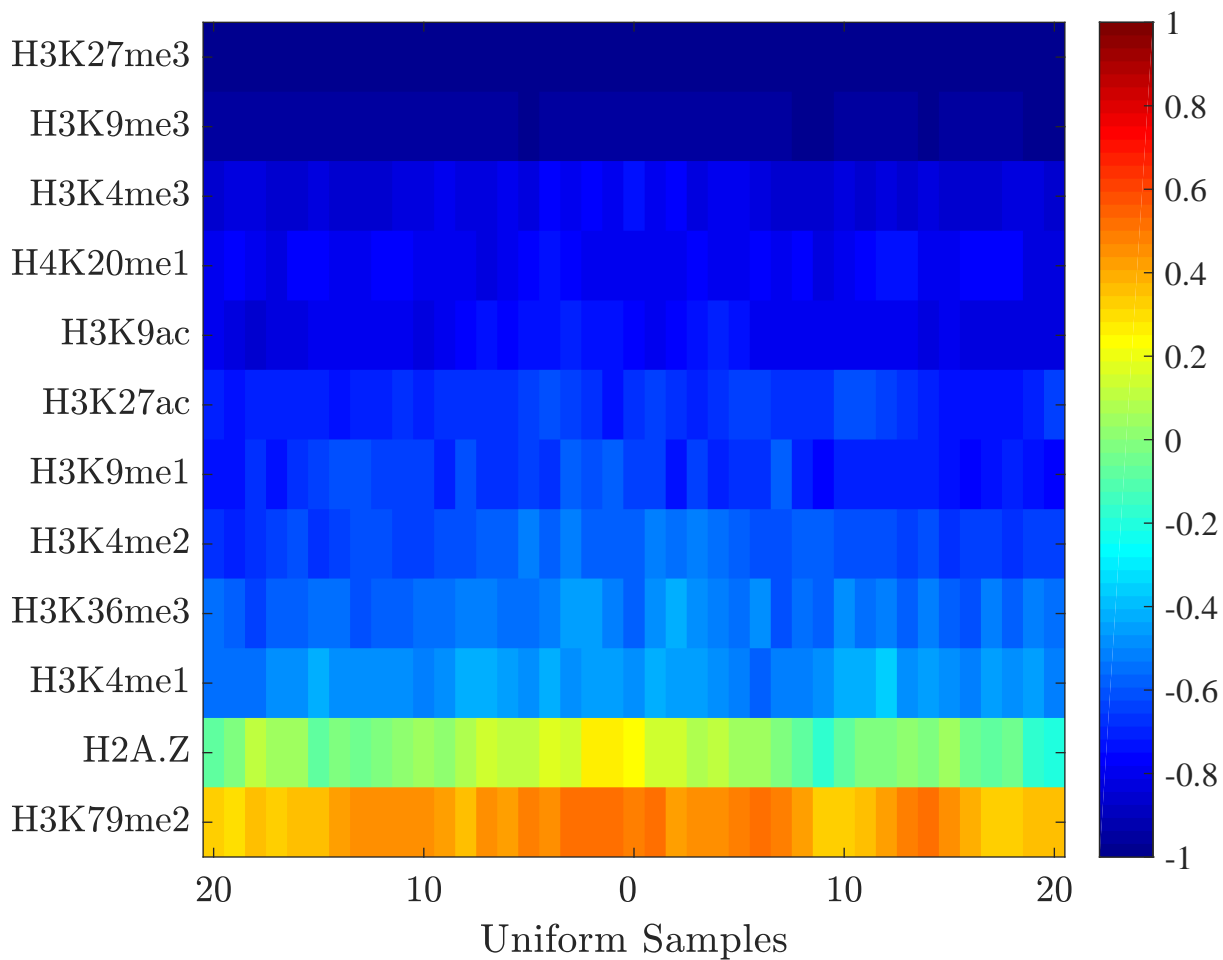

Supplement: Supplementary file 4 — HebbPlots of low-CpG promoters. This compressed file (.tar.gz) includes HebbPlots of low-CpG promoters active in 57 tissues/cell types. (TAR 2971 kb) [file 12859_2018_2312_MOESM4_ESM.tar › file5/E127.pdf]

Marks

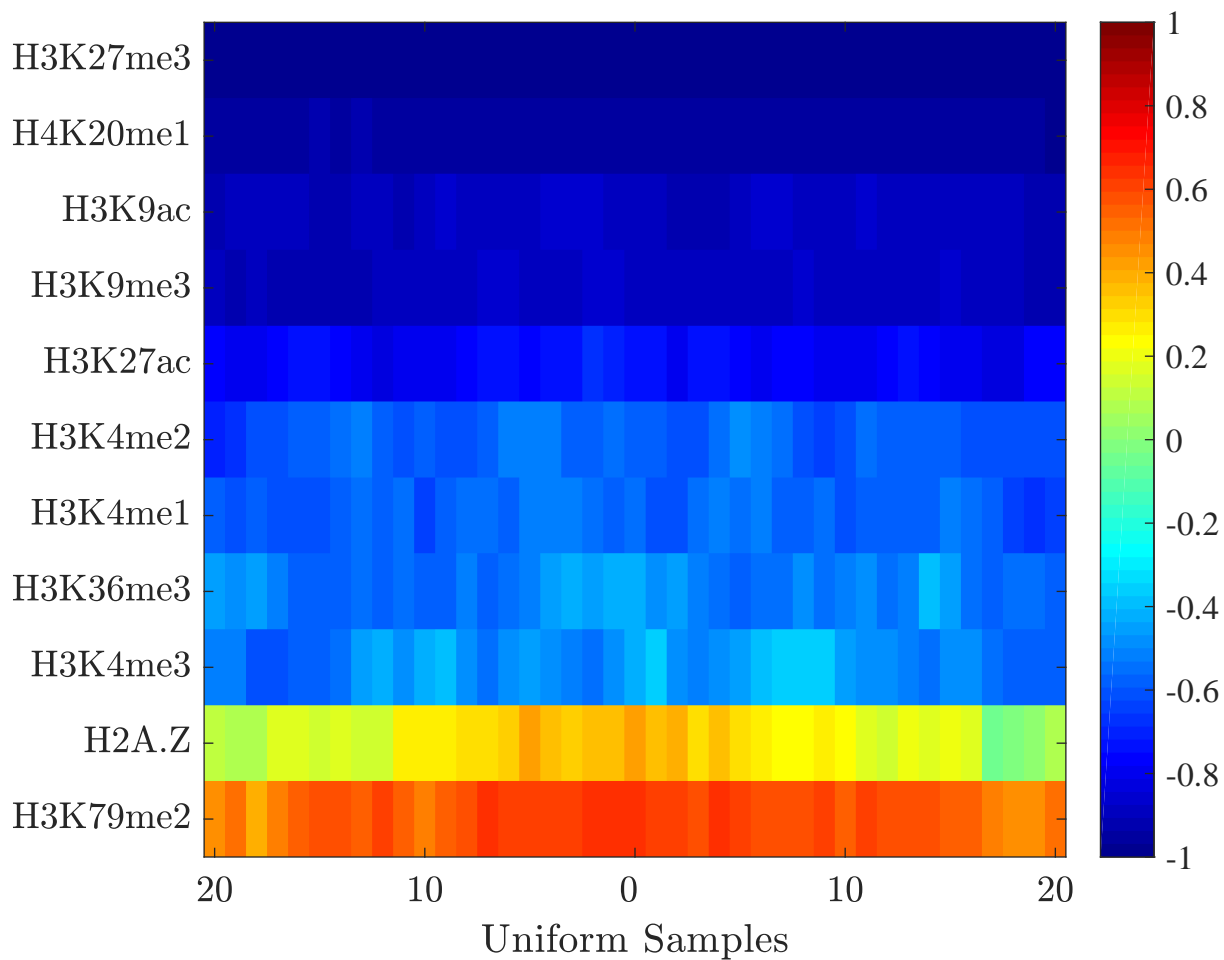

Supplement: Supplementary file 4 — HebbPlots of low-CpG promoters. This compressed file (.tar.gz) includes HebbPlots of low-CpG promoters active in 57 tissues/cell types. (TAR 2971 kb) [file 12859_2018_2312_MOESM4_ESM.tar › file5/E128.pdf]

Marks

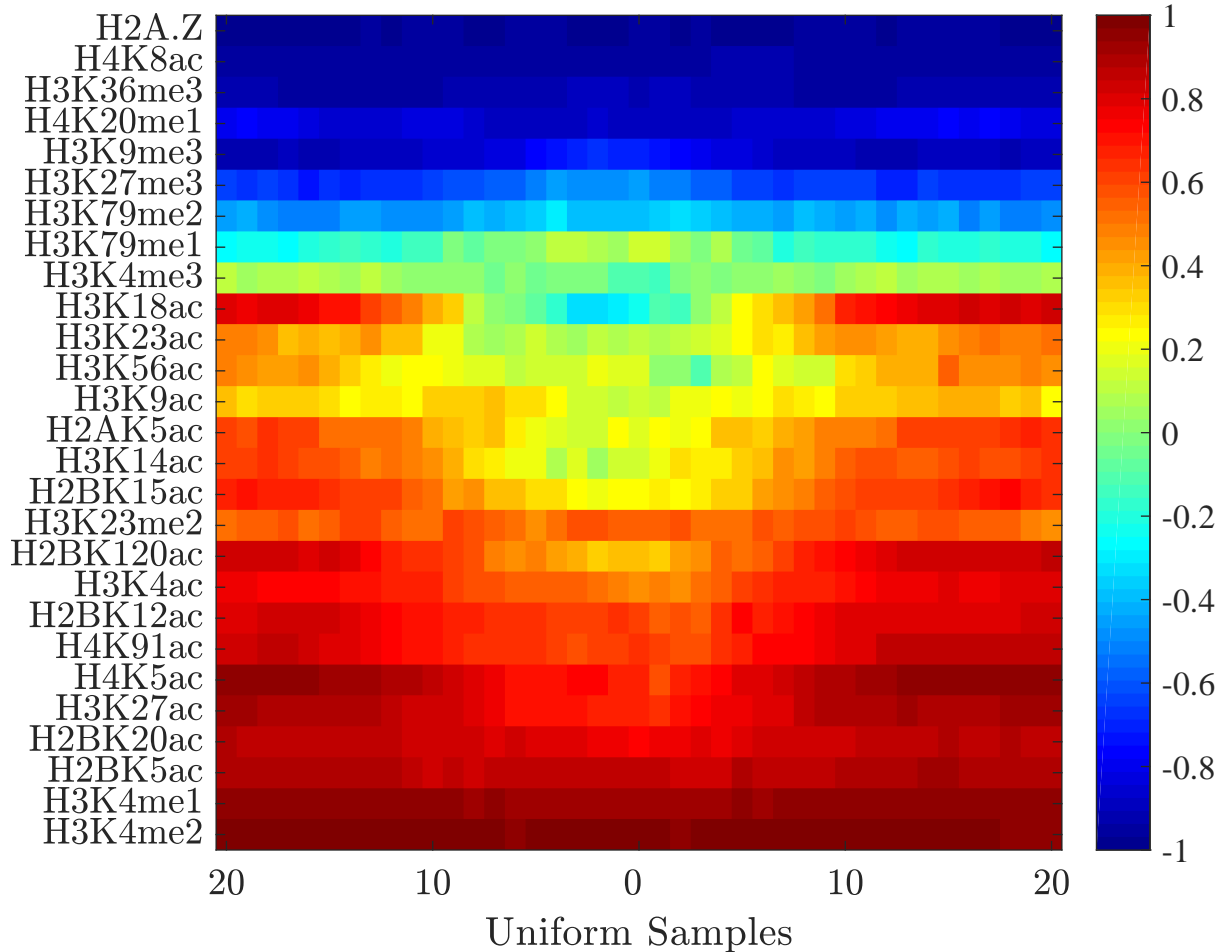

Supplement: Supplementary file 5 — HebbPlots of active enhancers. This compressed file (.tar.gz) includes HebbPlots of enhancers active in eight tissues/cell types. (TAR 439 kb) [file 12859_2018_2312_MOESM5_ESM.tar › file6/E003.pdf]

Marks

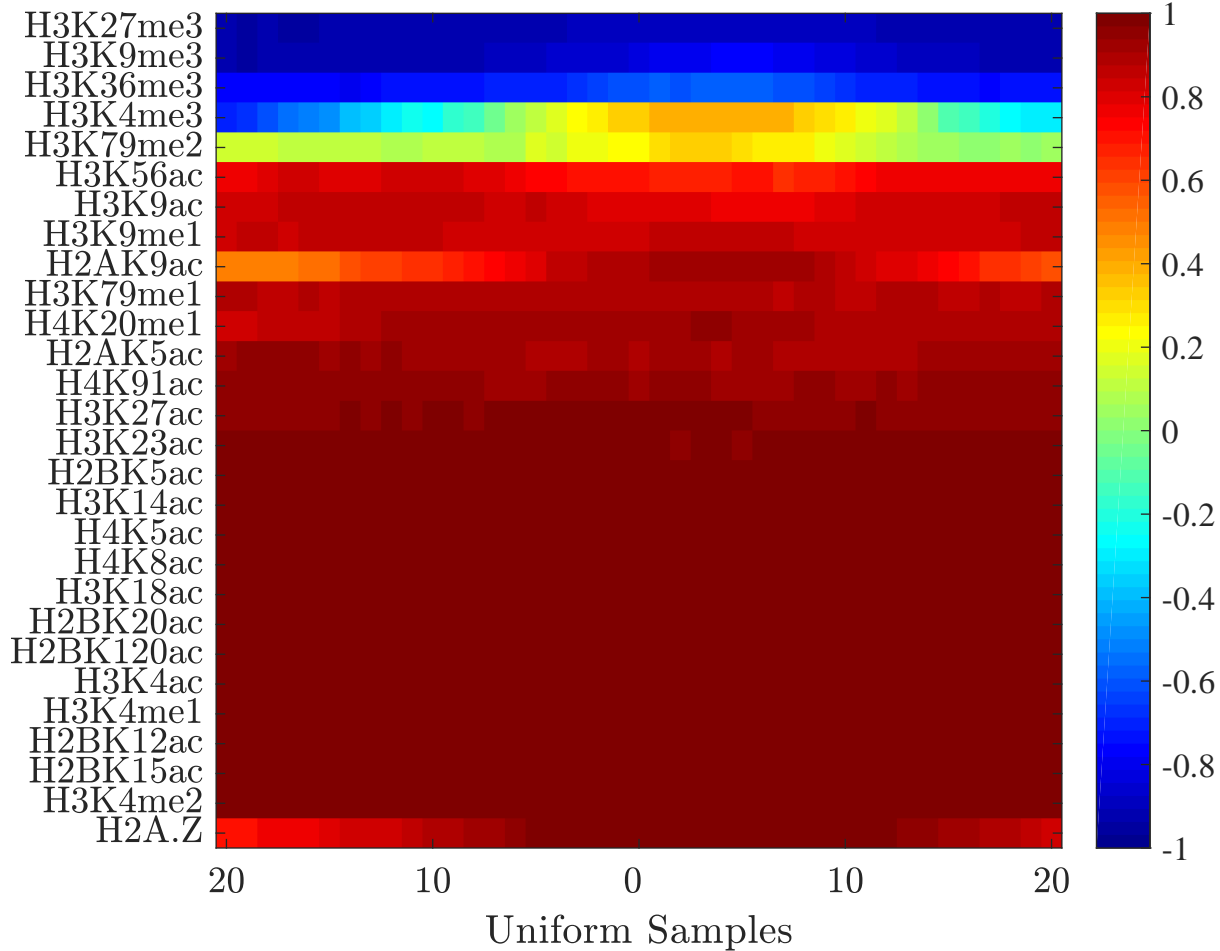

Supplement: Supplementary file 5 — HebbPlots of active enhancers. This compressed file (.tar.gz) includes HebbPlots of enhancers active in eight tissues/cell types. (TAR 439 kb) [file 12859_2018_2312_MOESM5_ESM.tar › file6/E017.pdf]

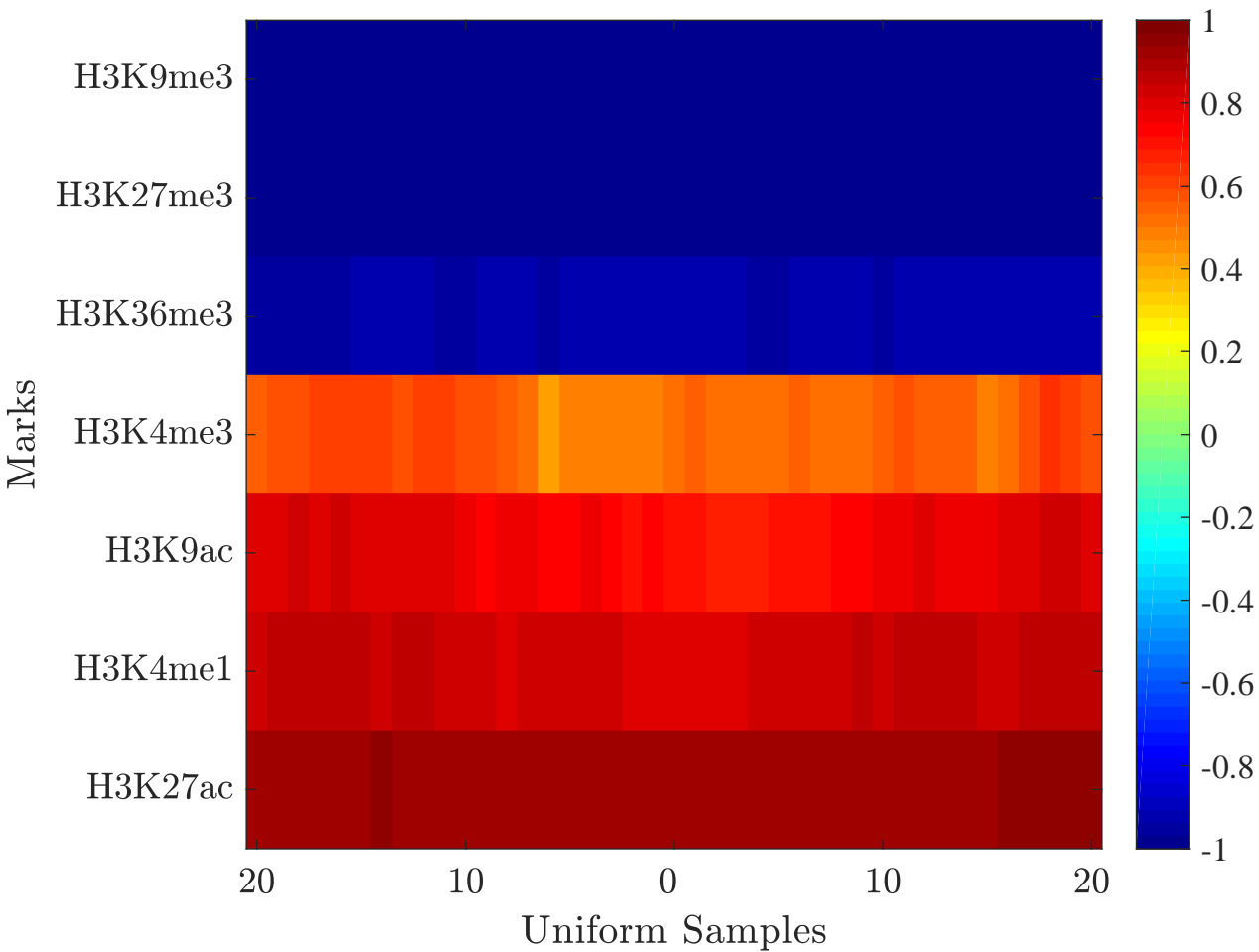

Supplement: Supplementary file 5 — HebbPlots of active enhancers. This compressed file (.tar.gz) includes HebbPlots of enhancers active in eight tissues/cell types. (TAR 439 kb) [file 12859_2018_2312_MOESM5_ESM.tar › file6/E066.pdf]

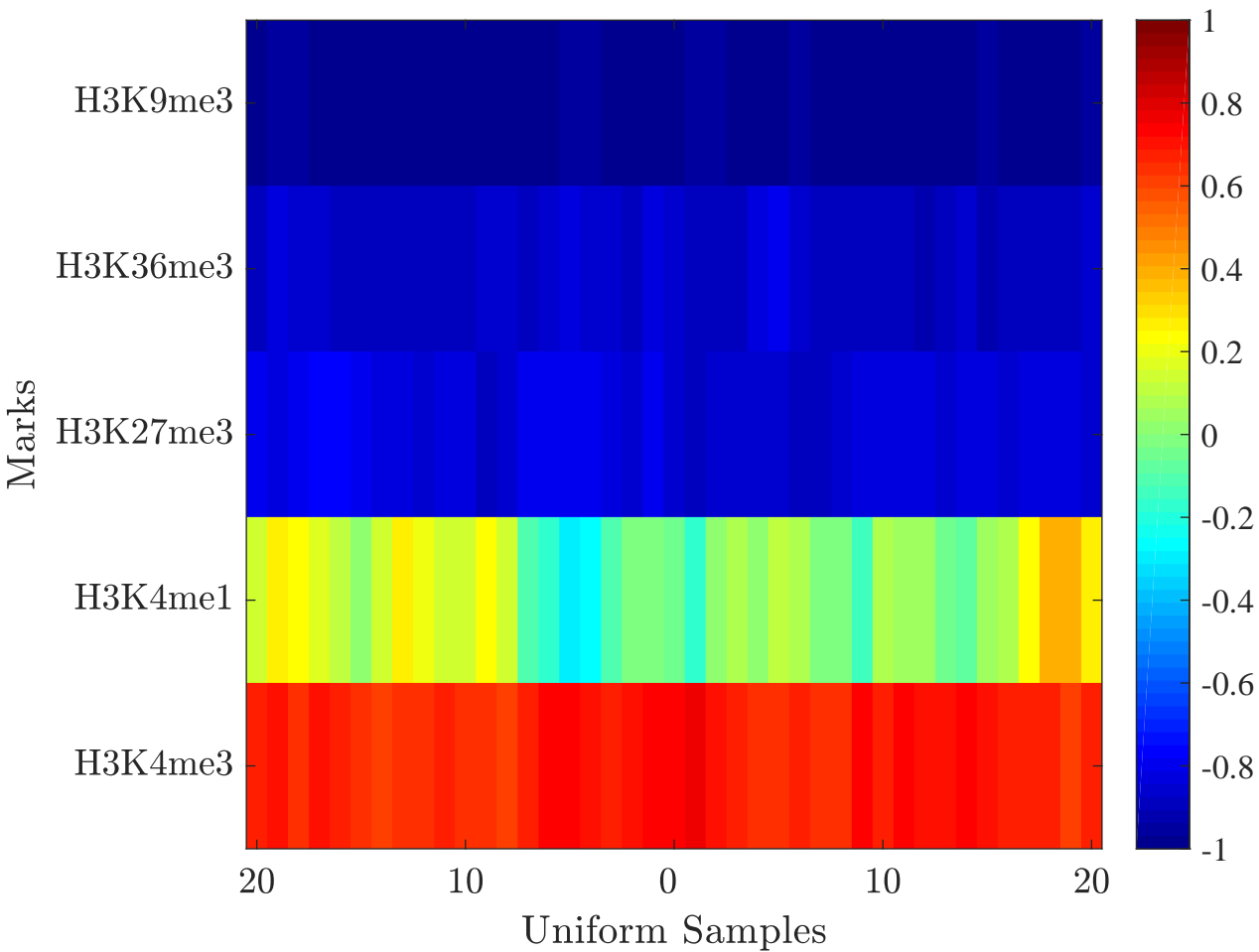

Supplement: Supplementary file 5 — HebbPlots of active enhancers. This compressed file (.tar.gz) includes HebbPlots of enhancers active in eight tissues/cell types. (TAR 439 kb) [file 12859_2018_2312_MOESM5_ESM.tar › file6/E082.pdf]

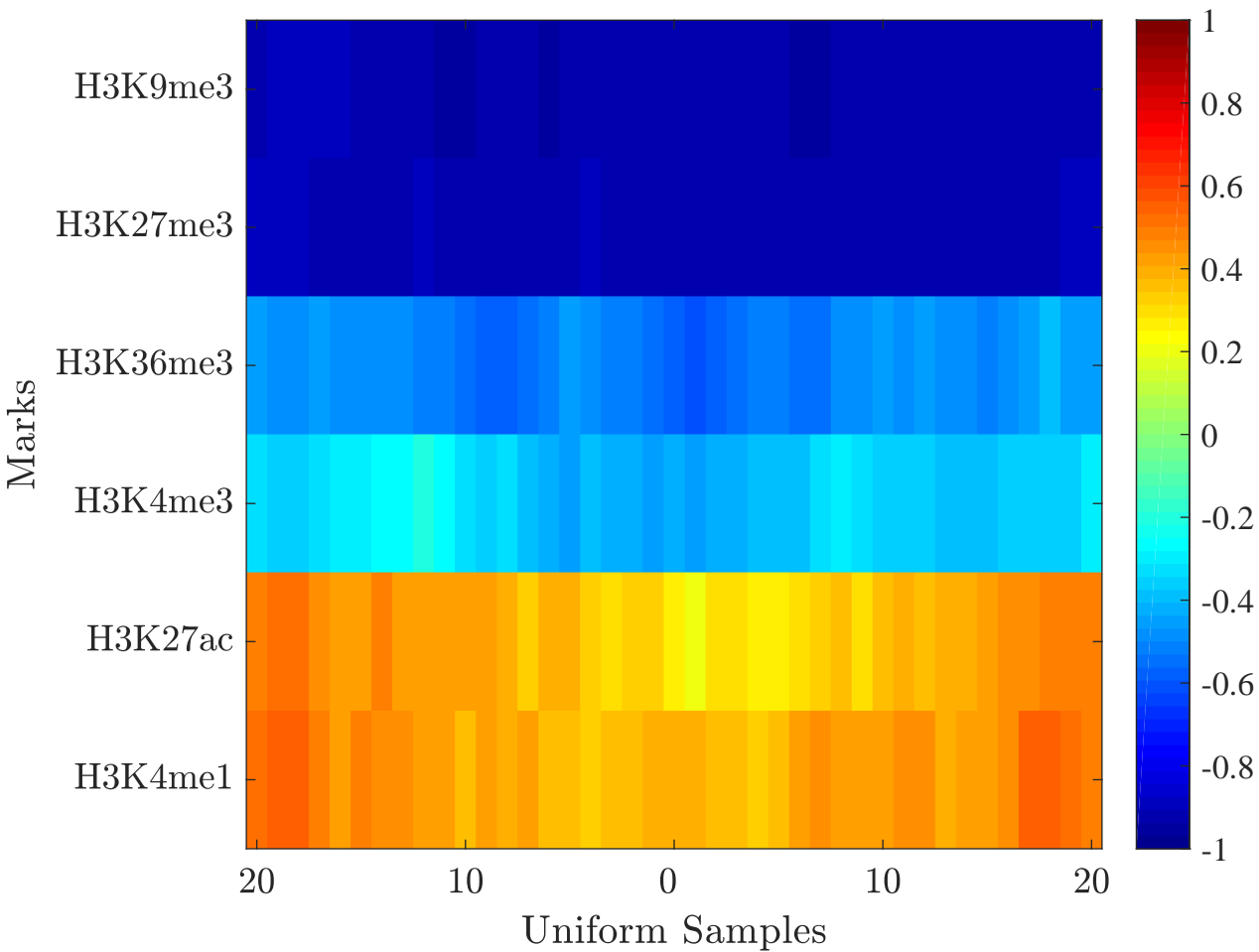

Supplement: Supplementary file 5 — HebbPlots of active enhancers. This compressed file (.tar.gz) includes HebbPlots of enhancers active in eight tissues/cell types. (TAR 439 kb) [file 12859_2018_2312_MOESM5_ESM.tar › file6/E085.pdf]

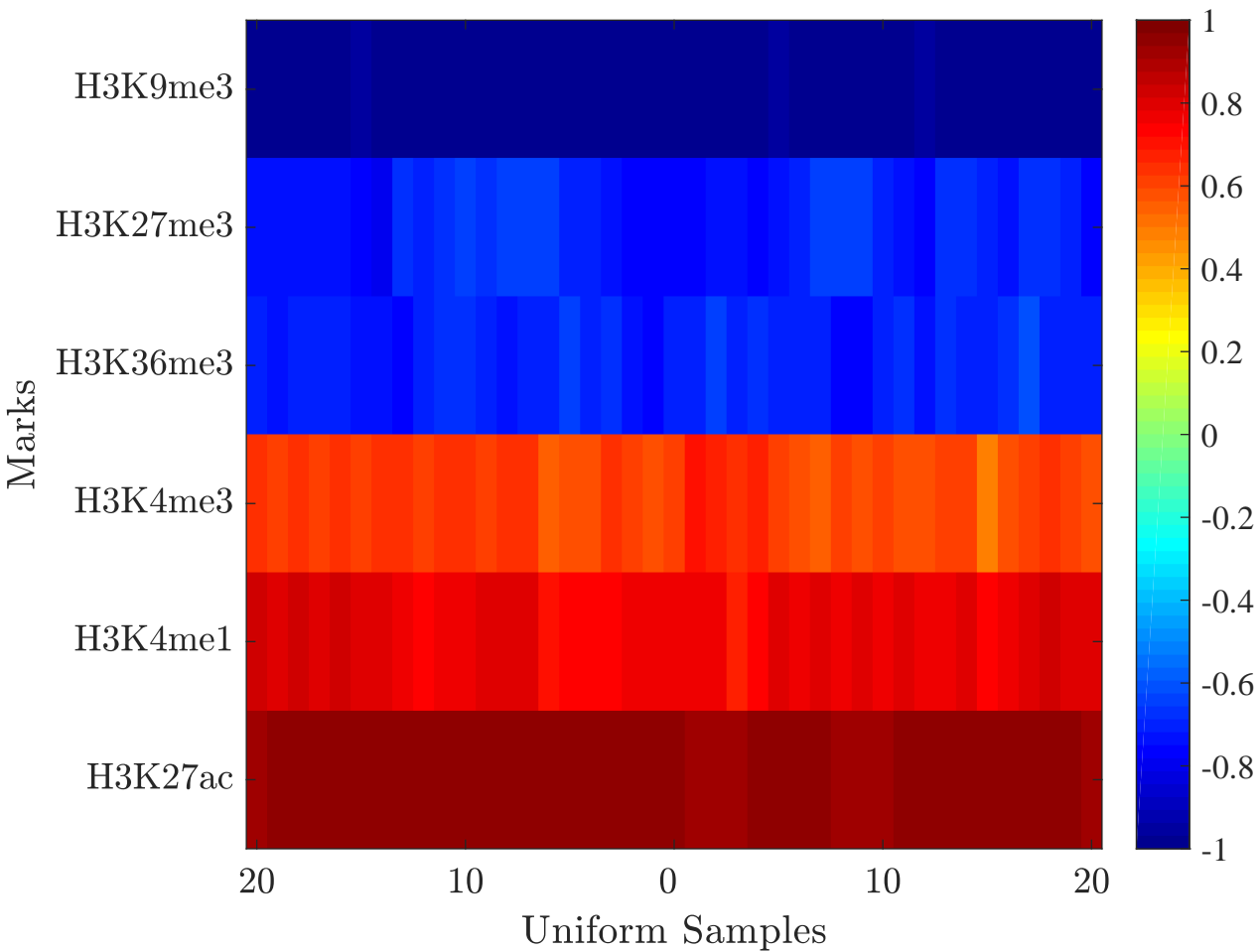

Supplement: Supplementary file 5 — HebbPlots of active enhancers. This compressed file (.tar.gz) includes HebbPlots of enhancers active in eight tissues/cell types. (TAR 439 kb) [file 12859_2018_2312_MOESM5_ESM.tar › file6/E095.pdf]

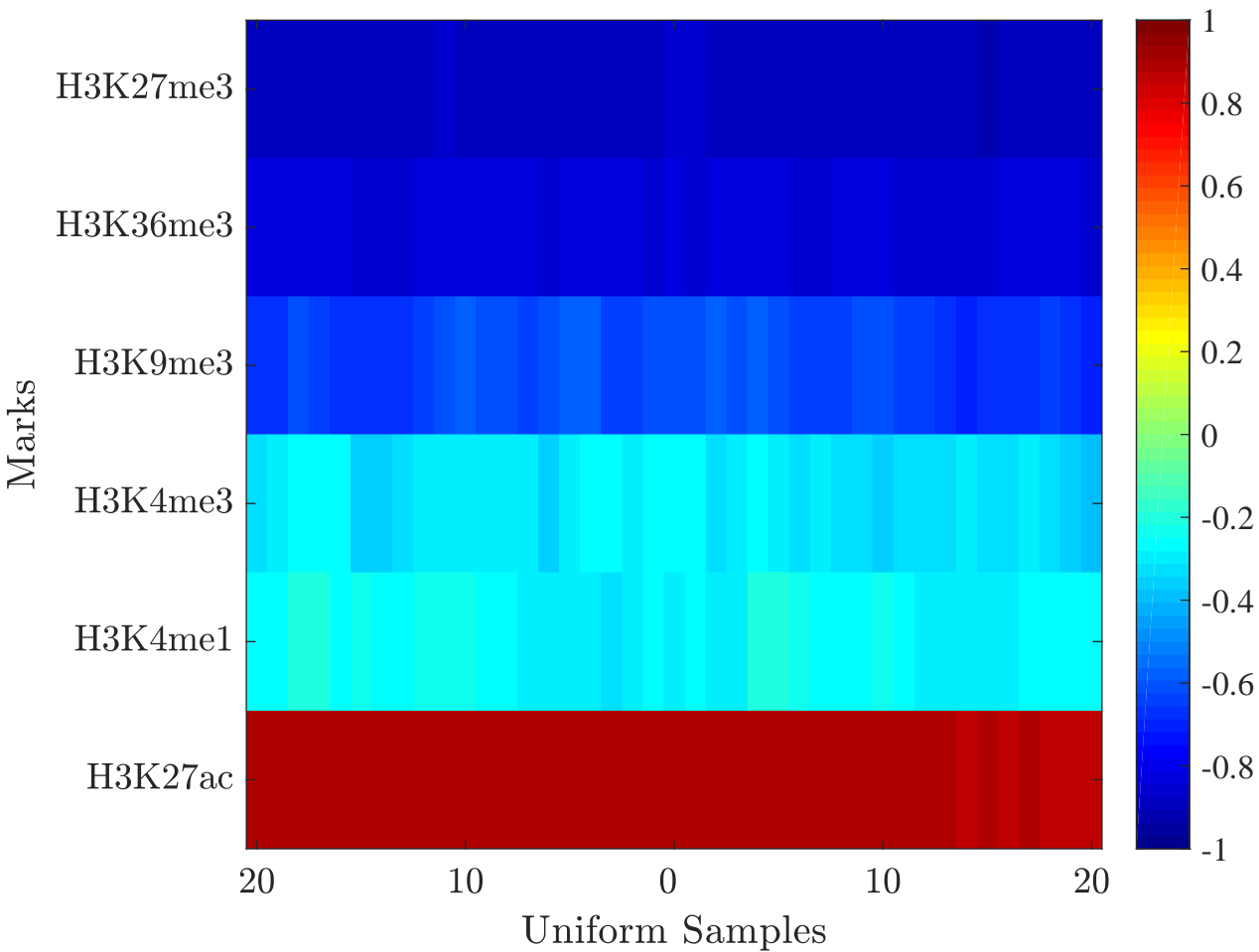

Supplement: Supplementary file 5 — HebbPlots of active enhancers. This compressed file (.tar.gz) includes HebbPlots of enhancers active in eight tissues/cell types. (TAR 439 kb) [file 12859_2018_2312_MOESM5_ESM.tar › file6/E096.pdf]

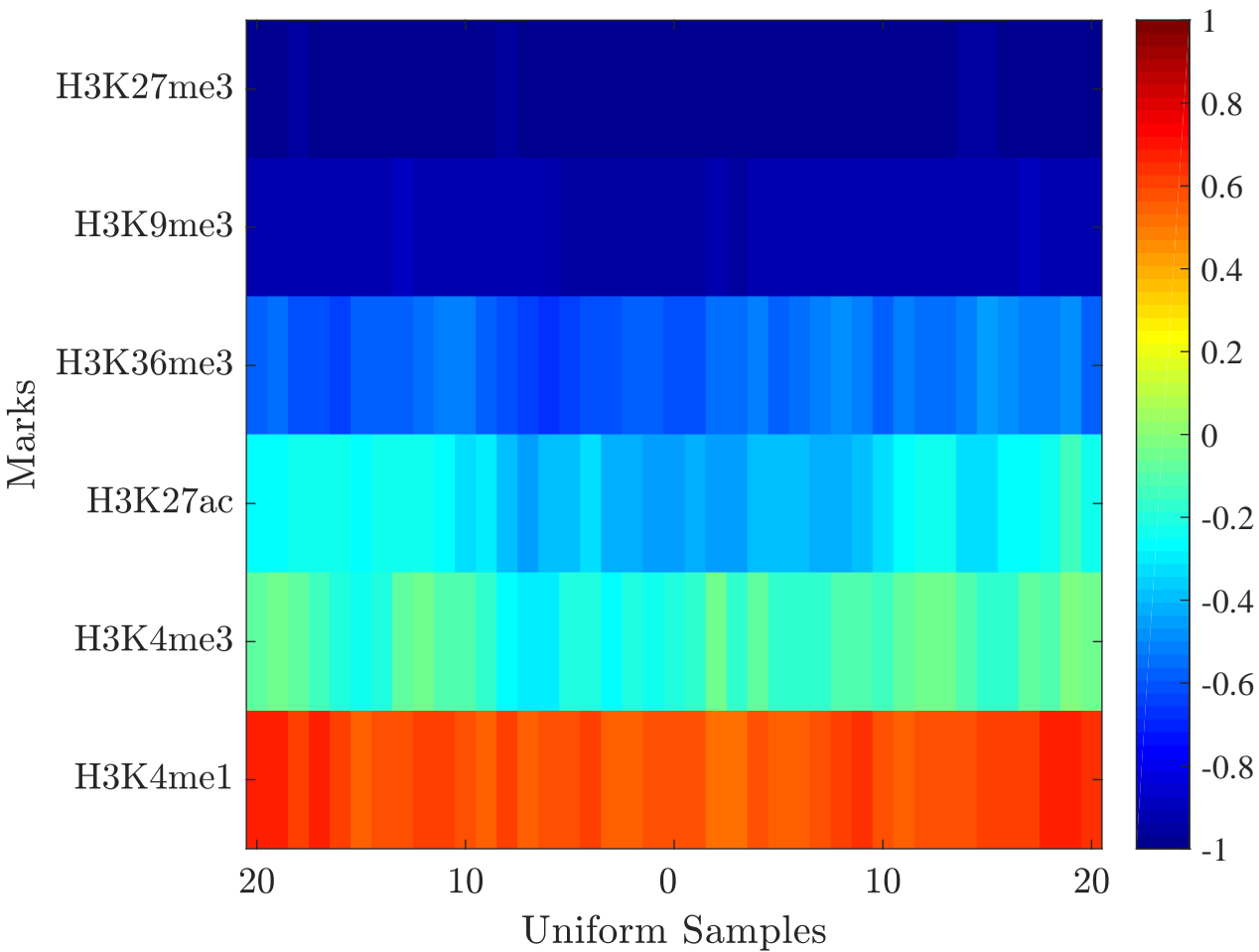

Supplement: Supplementary file 5 — HebbPlots of active enhancers. This compressed file (.tar.gz) includes HebbPlots of enhancers active in eight tissues/cell types. (TAR 439 kb) [file 12859_2018_2312_MOESM5_ESM.tar › file6/E098.pdf]

Marks

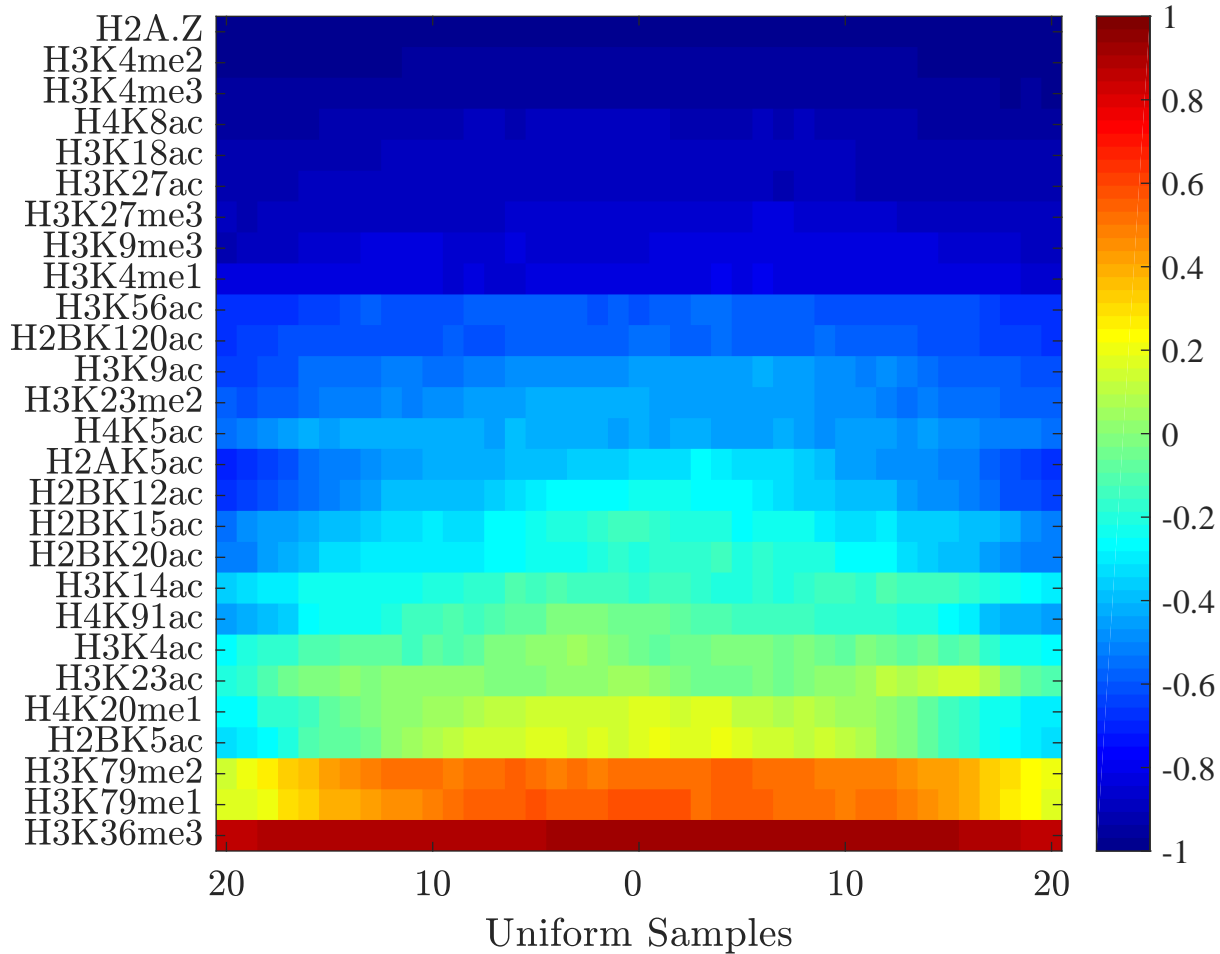

Supplement: Supplementary file 7 — HebbPlots of coding regions of active genes. This compressed file (.tar.gz) includes HebbPlots of genes active in 57 tissues/cell types. (TAR 2696 kb) [file 12859_2018_2312_MOESM7_ESM.tar › file8/E003.pdf]

Marks

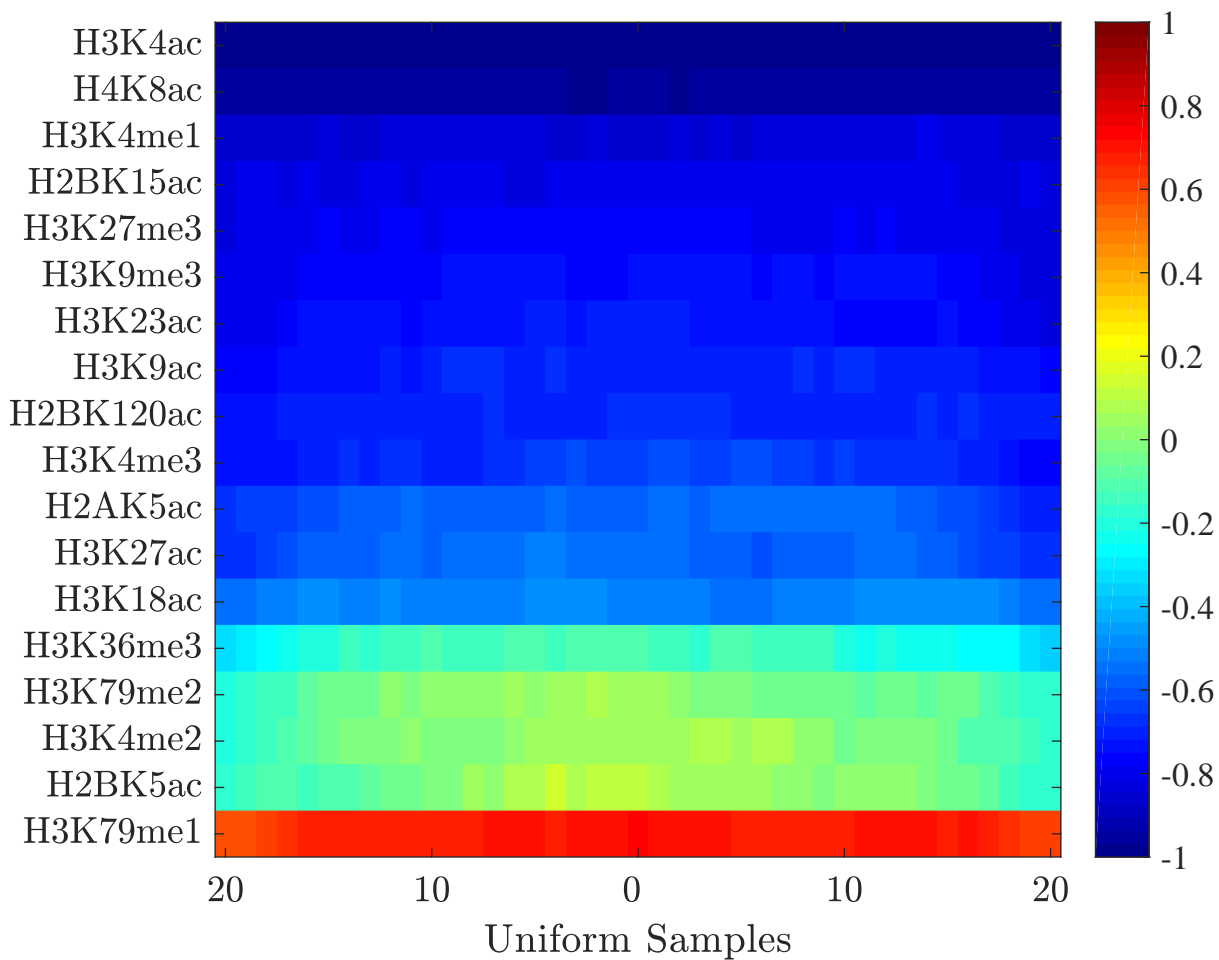

Supplement: Supplementary file 7 — HebbPlots of coding regions of active genes. This compressed file (.tar.gz) includes HebbPlots of genes active in 57 tissues/cell types. (TAR 2696 kb) [file 12859_2018_2312_MOESM7_ESM.tar › file8/E004.pdf]

Marks

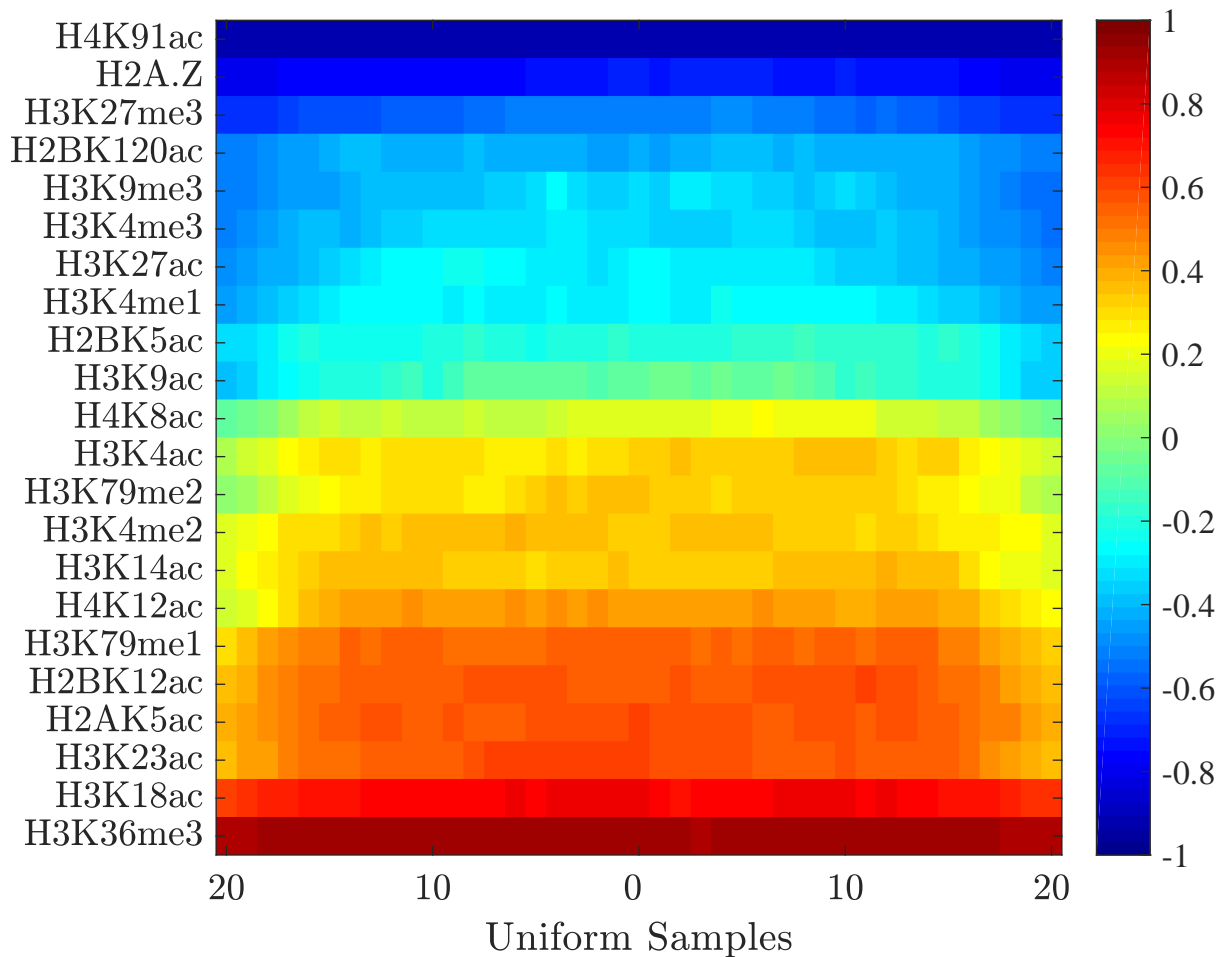

Supplement: Supplementary file 7 — HebbPlots of coding regions of active genes. This compressed file (.tar.gz) includes HebbPlots of genes active in 57 tissues/cell types. (TAR 2696 kb) [file 12859_2018_2312_MOESM7_ESM.tar › file8/E005.pdf]

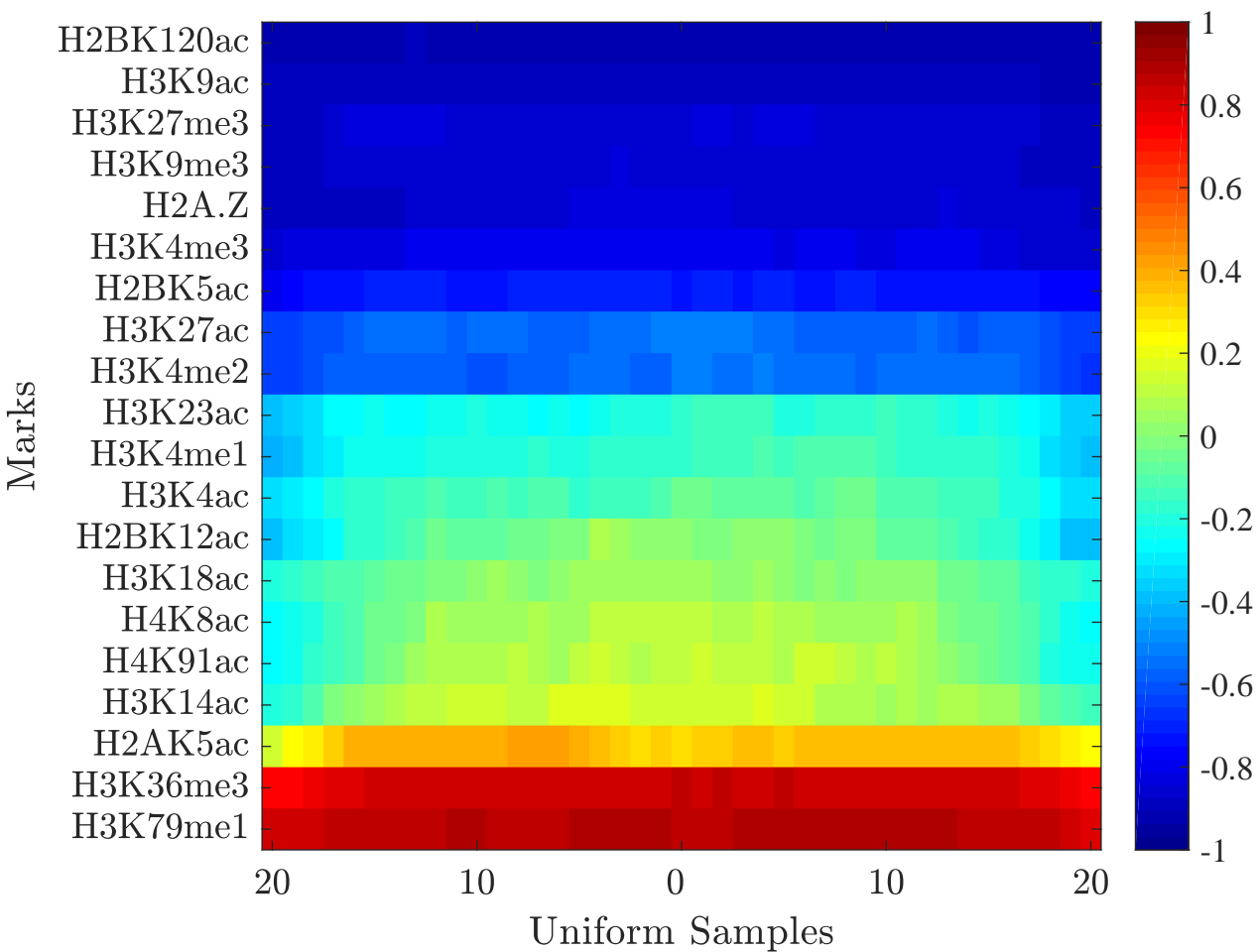

Supplement: Supplementary file 7 — HebbPlots of coding regions of active genes. This compressed file (.tar.gz) includes HebbPlots of genes active in 57 tissues/cell types. (TAR 2696 kb) [file 12859_2018_2312_MOESM7_ESM.tar › file8/E006.pdf]

Marks

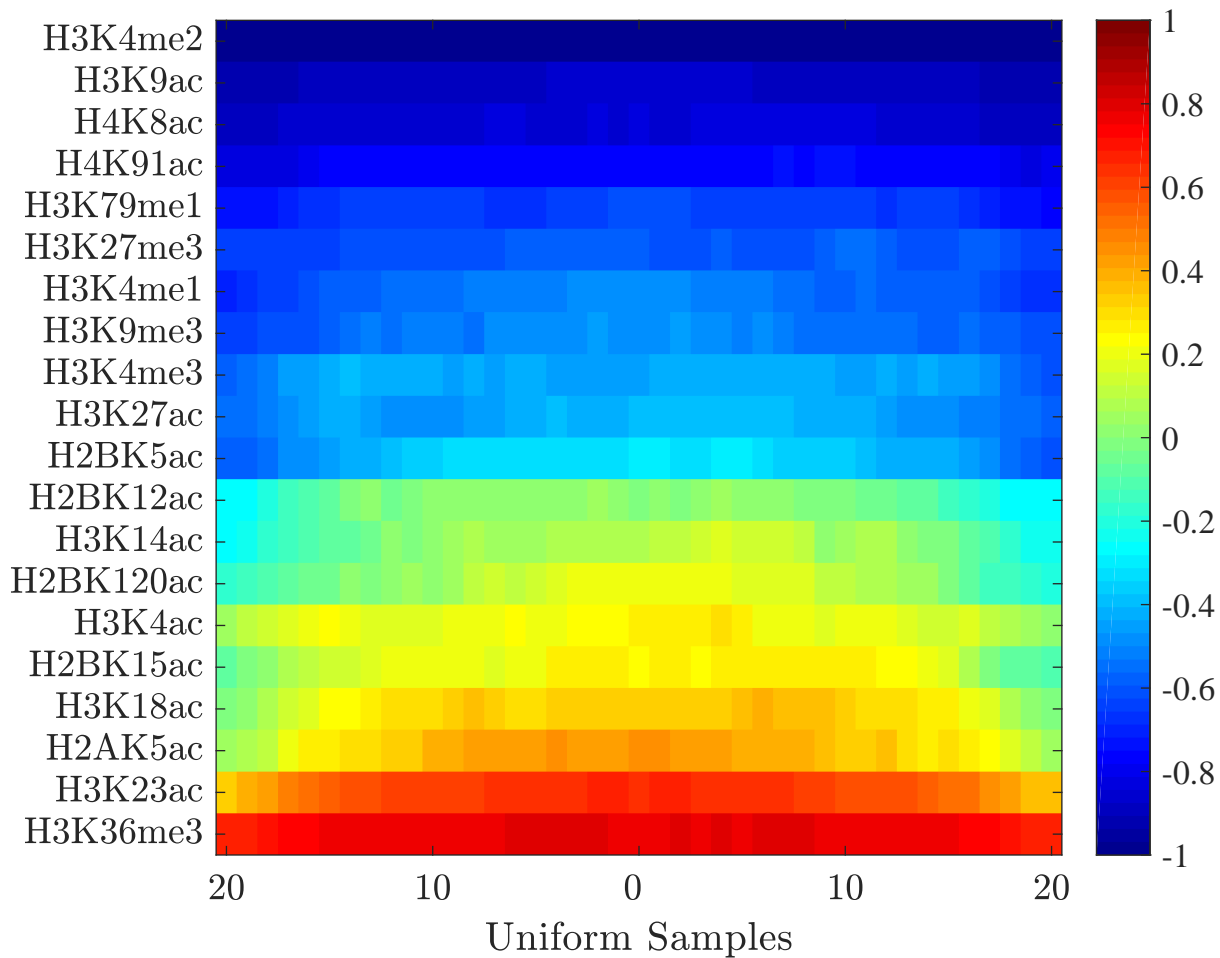

Supplement: Supplementary file 7 — HebbPlots of coding regions of active genes. This compressed file (.tar.gz) includes HebbPlots of genes active in 57 tissues/cell types. (TAR 2696 kb) [file 12859_2018_2312_MOESM7_ESM.tar › file8/E007.pdf]

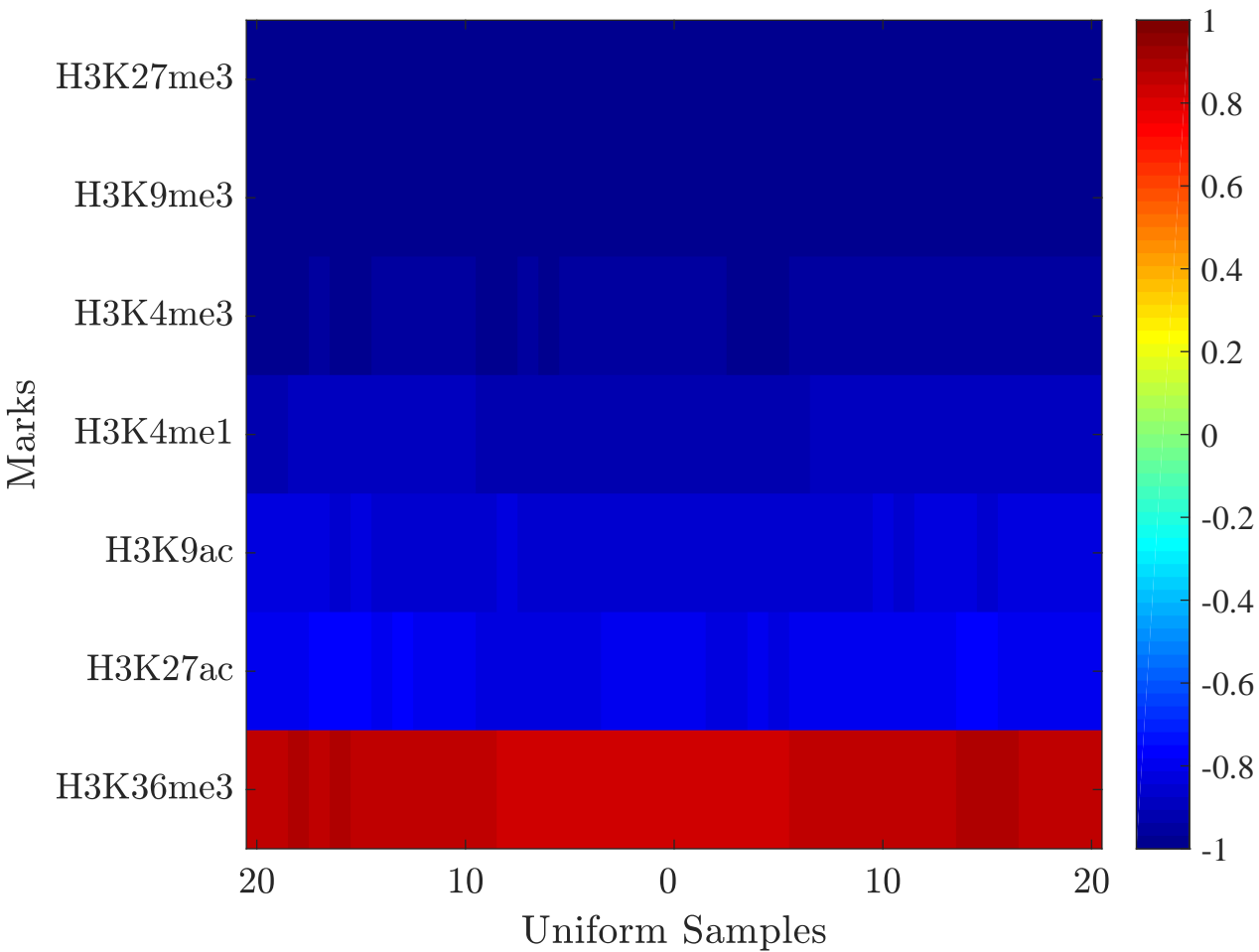

Supplement: Supplementary file 7 — HebbPlots of coding regions of active genes. This compressed file (.tar.gz) includes HebbPlots of genes active in 57 tissues/cell types. (TAR 2696 kb) [file 12859_2018_2312_MOESM7_ESM.tar › file8/E011.pdf]

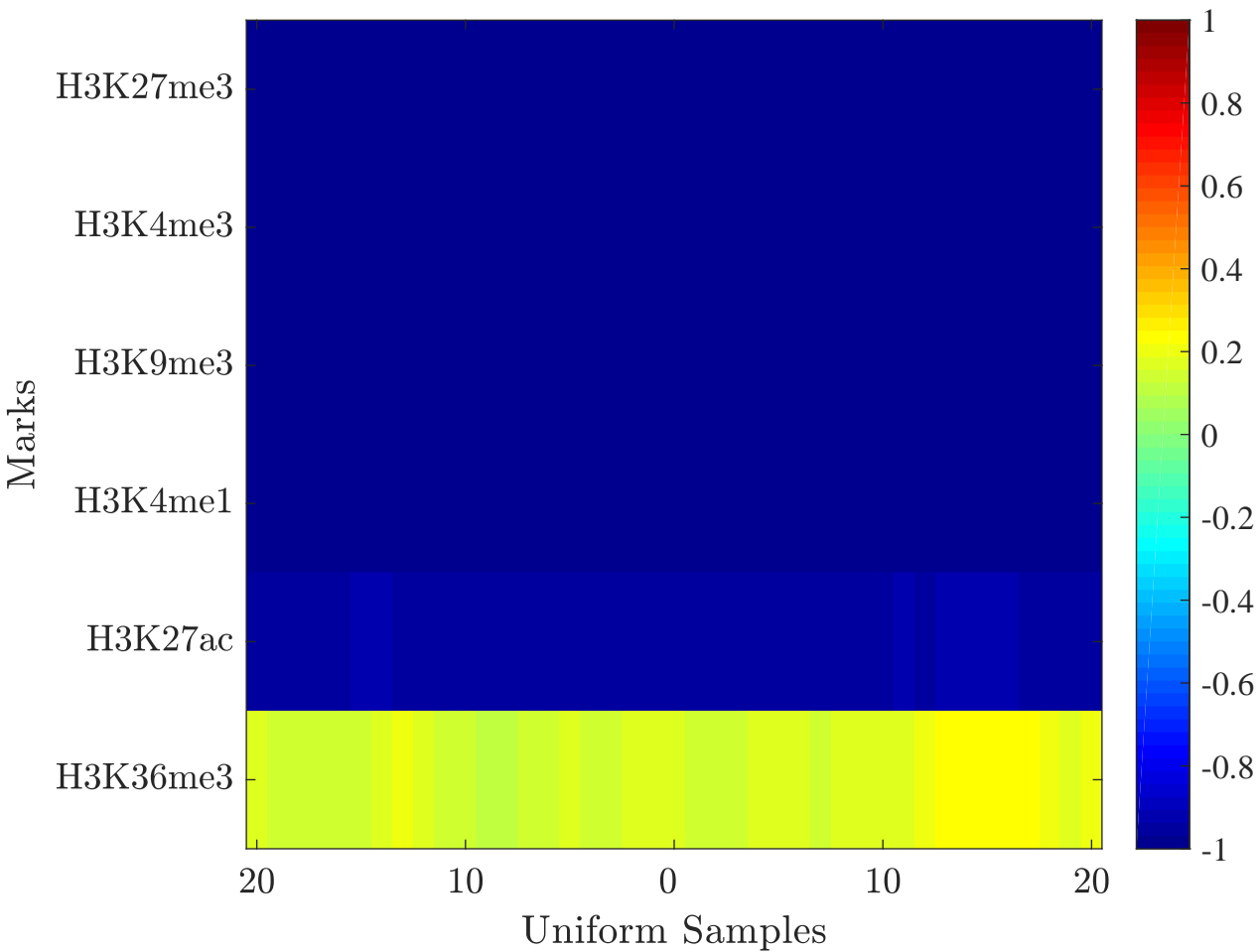

Supplement: Supplementary file 7 — HebbPlots of coding regions of active genes. This compressed file (.tar.gz) includes HebbPlots of genes active in 57 tissues/cell types. (TAR 2696 kb) [file 12859_2018_2312_MOESM7_ESM.tar › file8/E012.pdf]

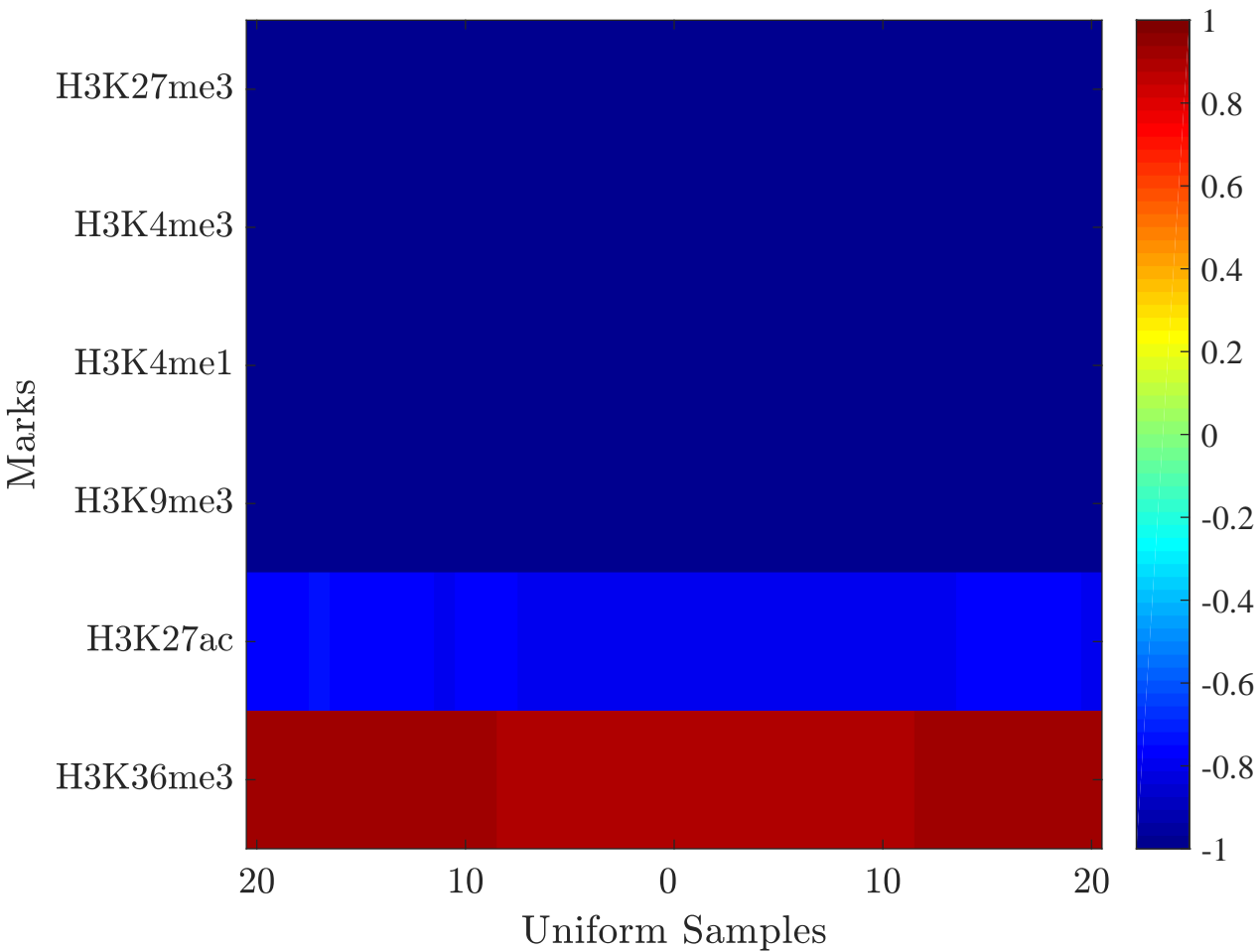

Supplement: Supplementary file 7 — HebbPlots of coding regions of active genes. This compressed file (.tar.gz) includes HebbPlots of genes active in 57 tissues/cell types. (TAR 2696 kb) [file 12859_2018_2312_MOESM7_ESM.tar › file8/E013.pdf]

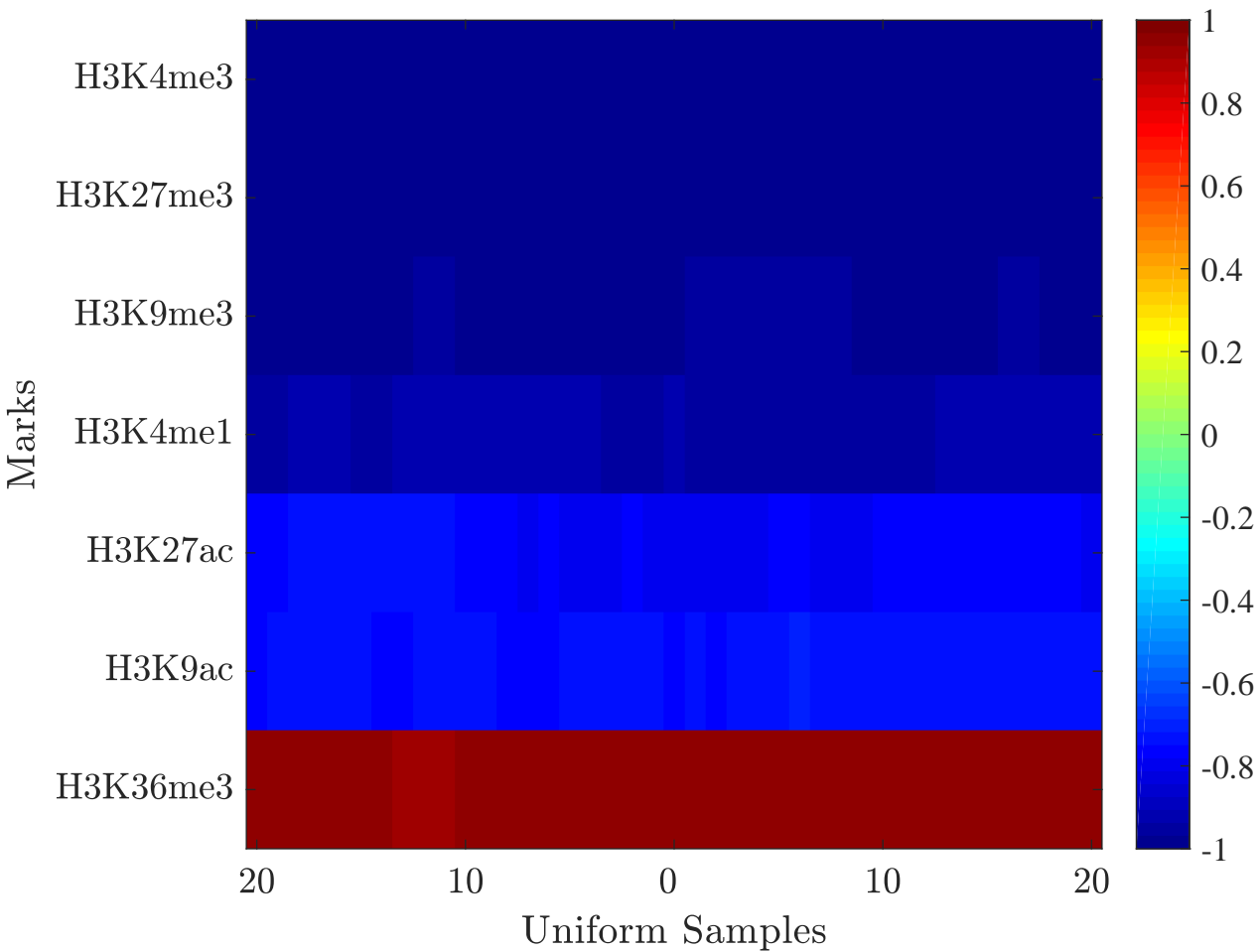

Supplement: Supplementary file 7 — HebbPlots of coding regions of active genes. This compressed file (.tar.gz) includes HebbPlots of genes active in 57 tissues/cell types. (TAR 2696 kb) [file 12859_2018_2312_MOESM7_ESM.tar › file8/E016.pdf]

Marks

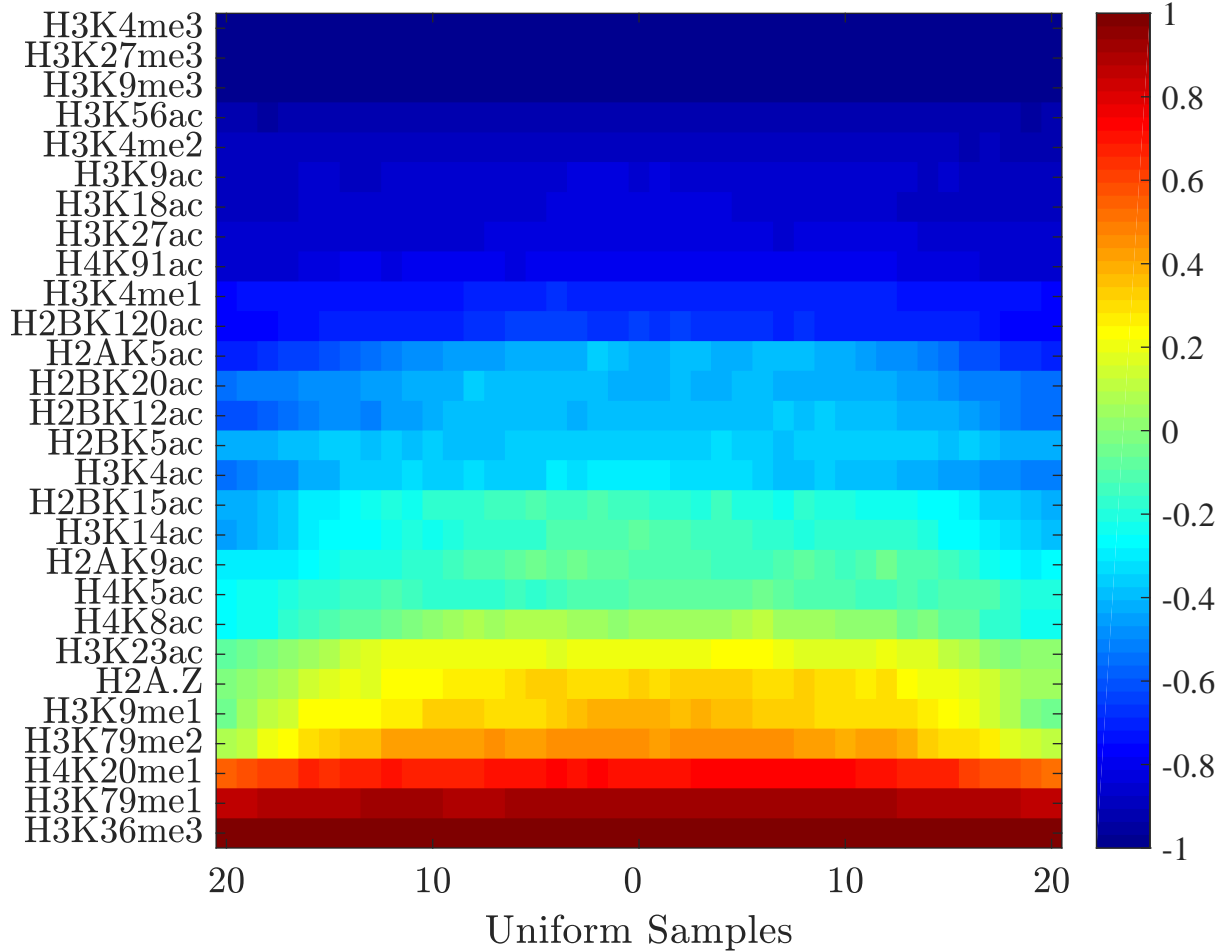

Supplement: Supplementary file 7 — HebbPlots of coding regions of active genes. This compressed file (.tar.gz) includes HebbPlots of genes active in 57 tissues/cell types. (TAR 2696 kb) [file 12859_2018_2312_MOESM7_ESM.tar › file8/E017.pdf]

Marks

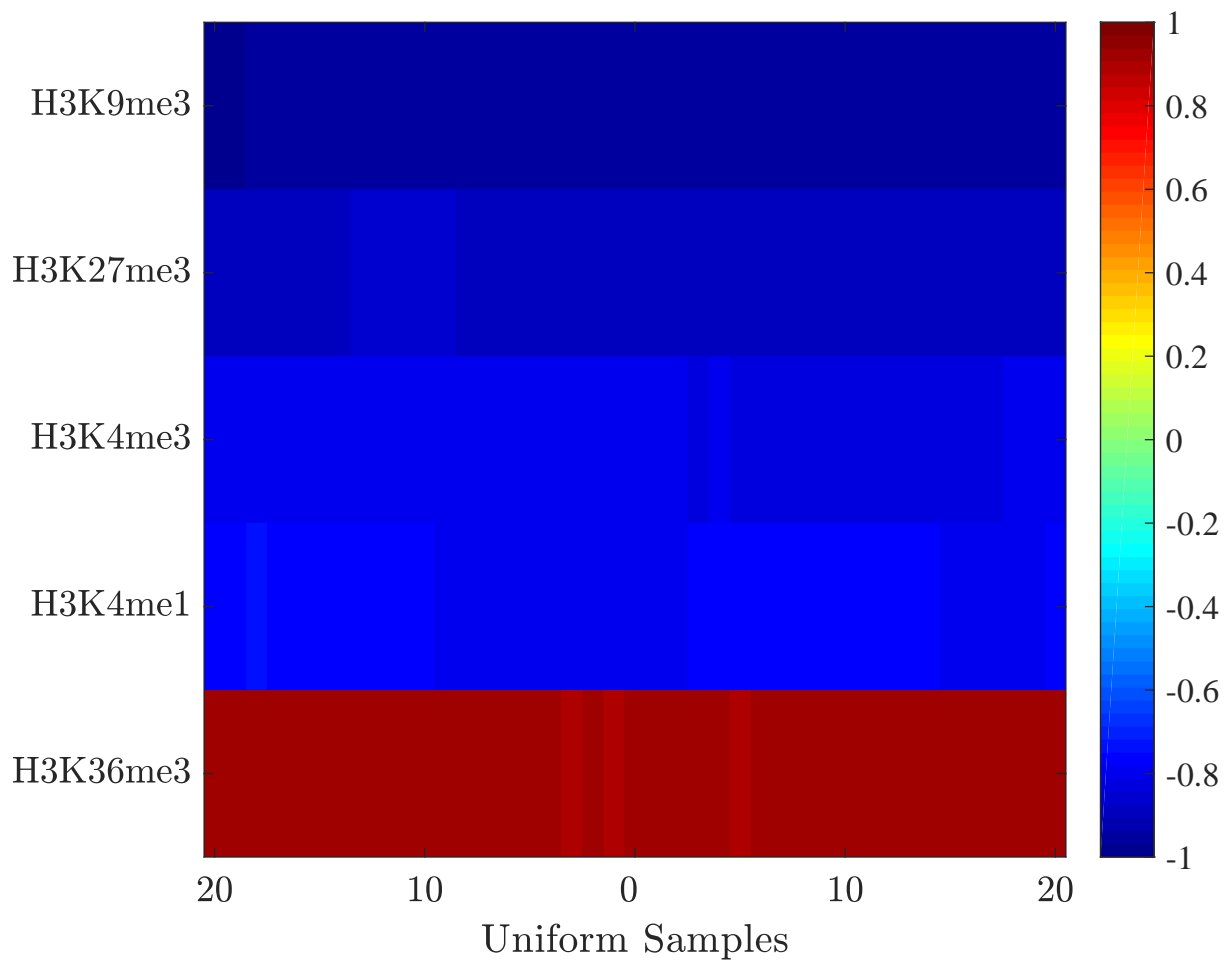

Supplement: Supplementary file 7 — HebbPlots of coding regions of active genes. This compressed file (.tar.gz) includes HebbPlots of genes active in 57 tissues/cell types. (TAR 2696 kb) [file 12859_2018_2312_MOESM7_ESM.tar › file8/E024.pdf]

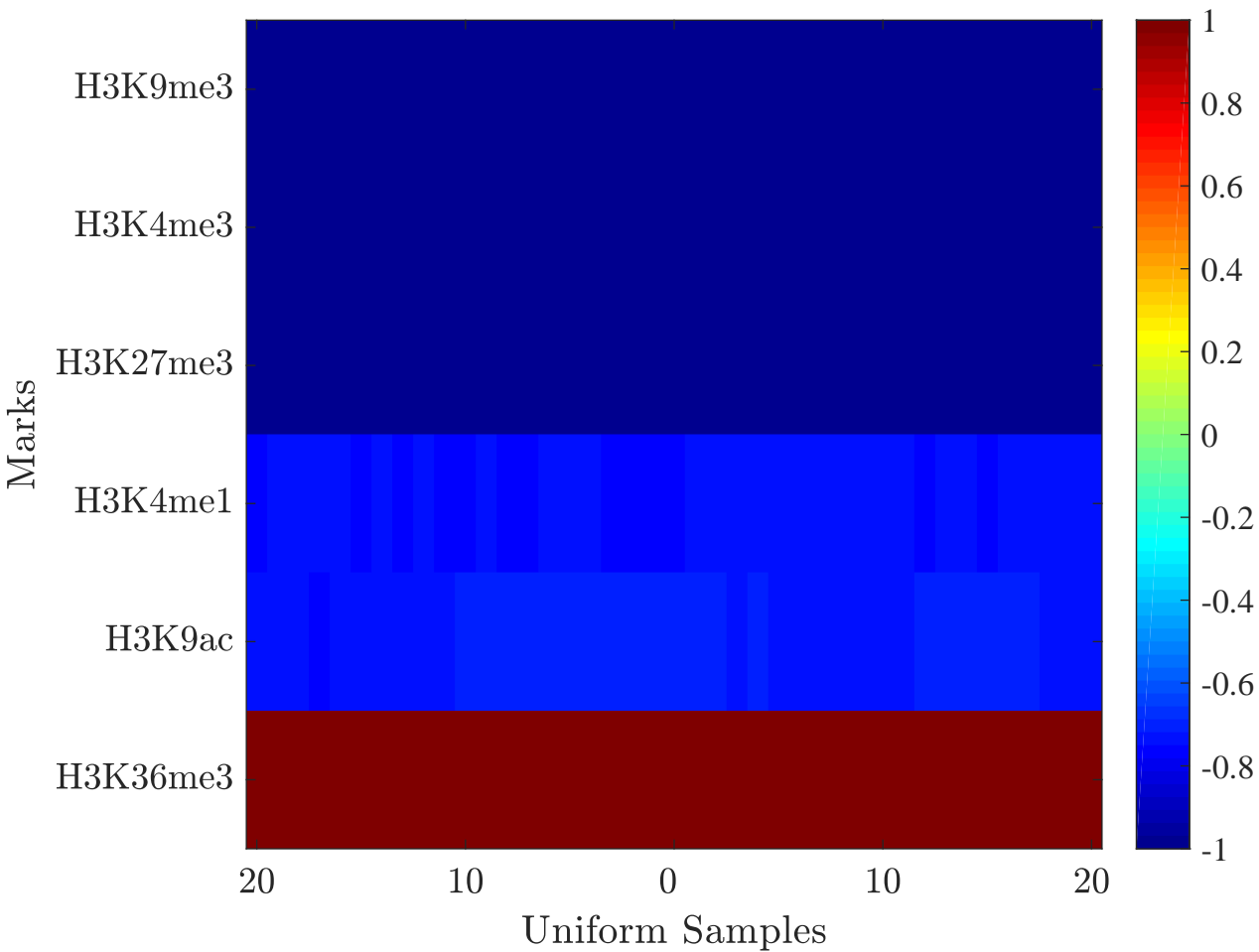

Supplement: Supplementary file 7 — HebbPlots of coding regions of active genes. This compressed file (.tar.gz) includes HebbPlots of genes active in 57 tissues/cell types. (TAR 2696 kb) [file 12859_2018_2312_MOESM7_ESM.tar › file8/E027.pdf]

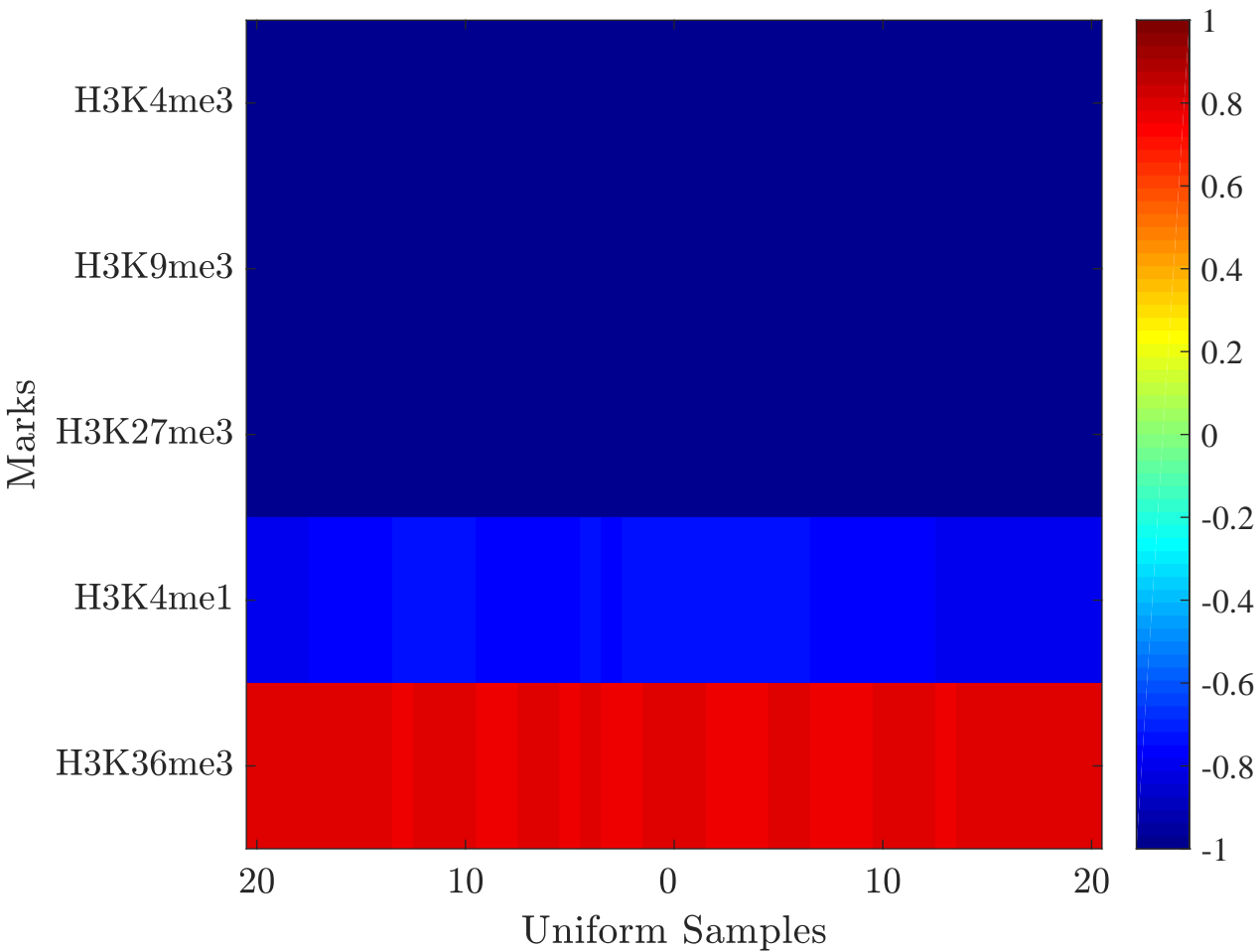

Supplement: Supplementary file 7 — HebbPlots of coding regions of active genes. This compressed file (.tar.gz) includes HebbPlots of genes active in 57 tissues/cell types. (TAR 2696 kb) [file 12859_2018_2312_MOESM7_ESM.tar › file8/E028.pdf]

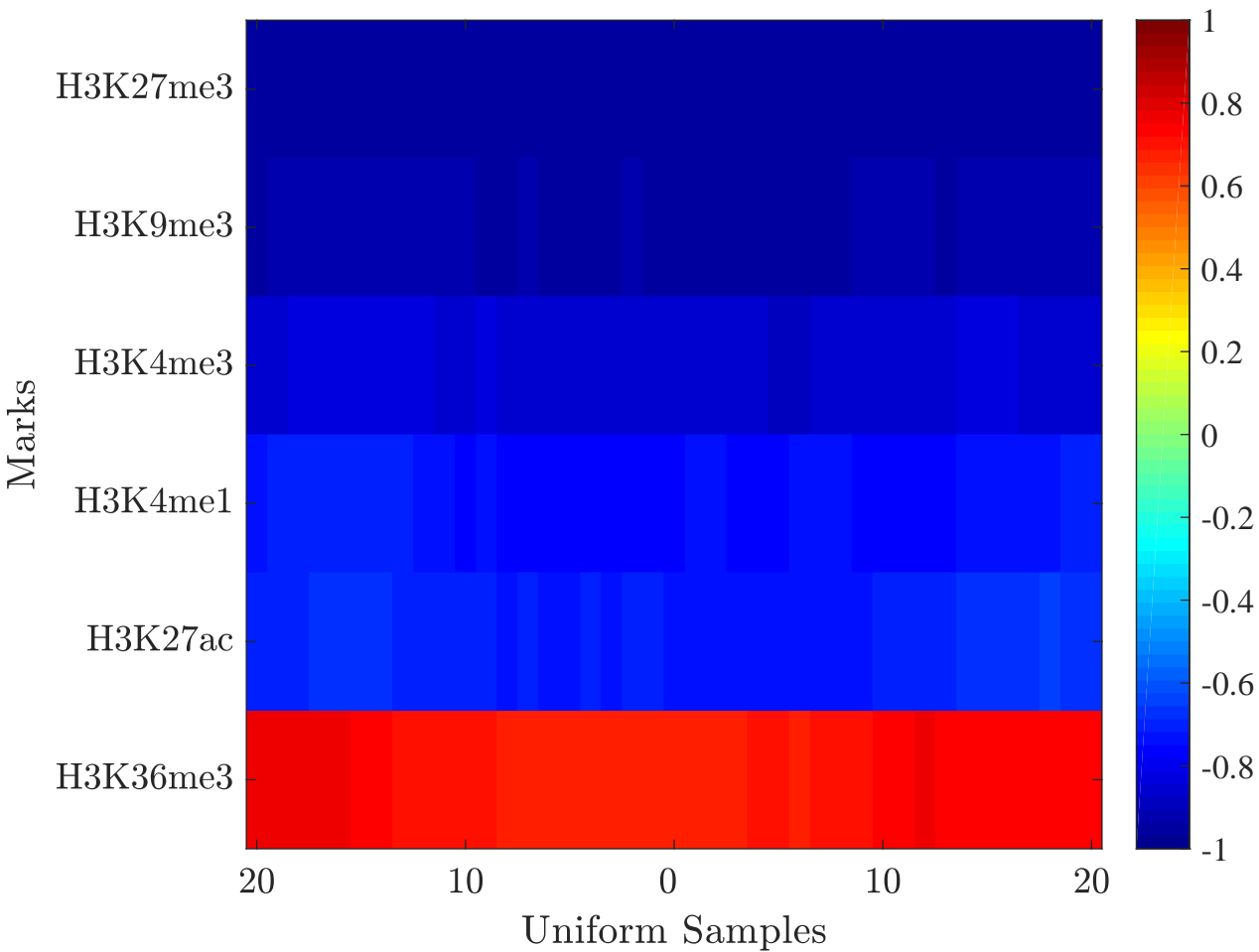

Supplement: Supplementary file 7 — HebbPlots of coding regions of active genes. This compressed file (.tar.gz) includes HebbPlots of genes active in 57 tissues/cell types. (TAR 2696 kb) [file 12859_2018_2312_MOESM7_ESM.tar › file8/E037.pdf]

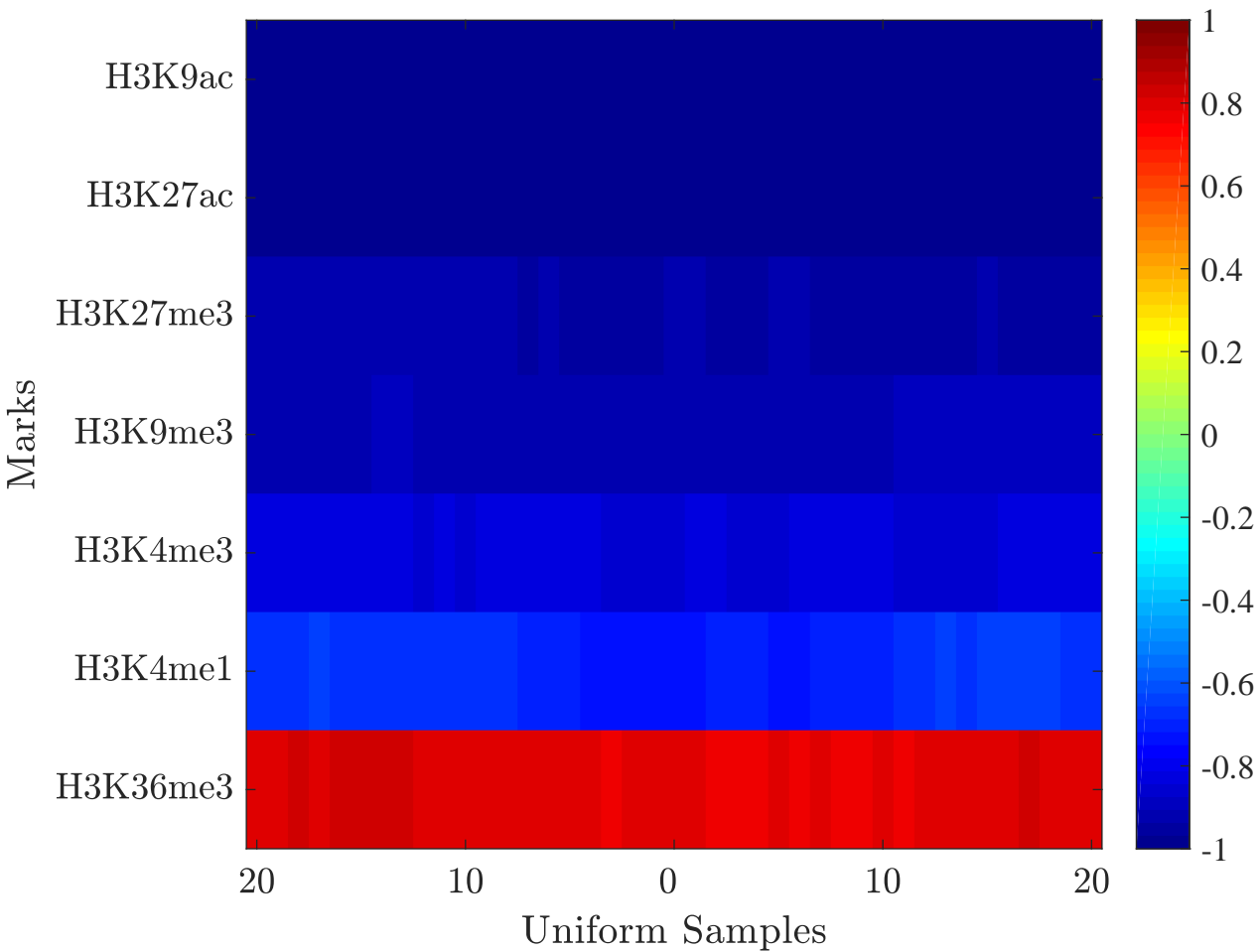

Supplement: Supplementary file 7 — HebbPlots of coding regions of active genes. This compressed file (.tar.gz) includes HebbPlots of genes active in 57 tissues/cell types. (TAR 2696 kb) [file 12859_2018_2312_MOESM7_ESM.tar › file8/E038.pdf]

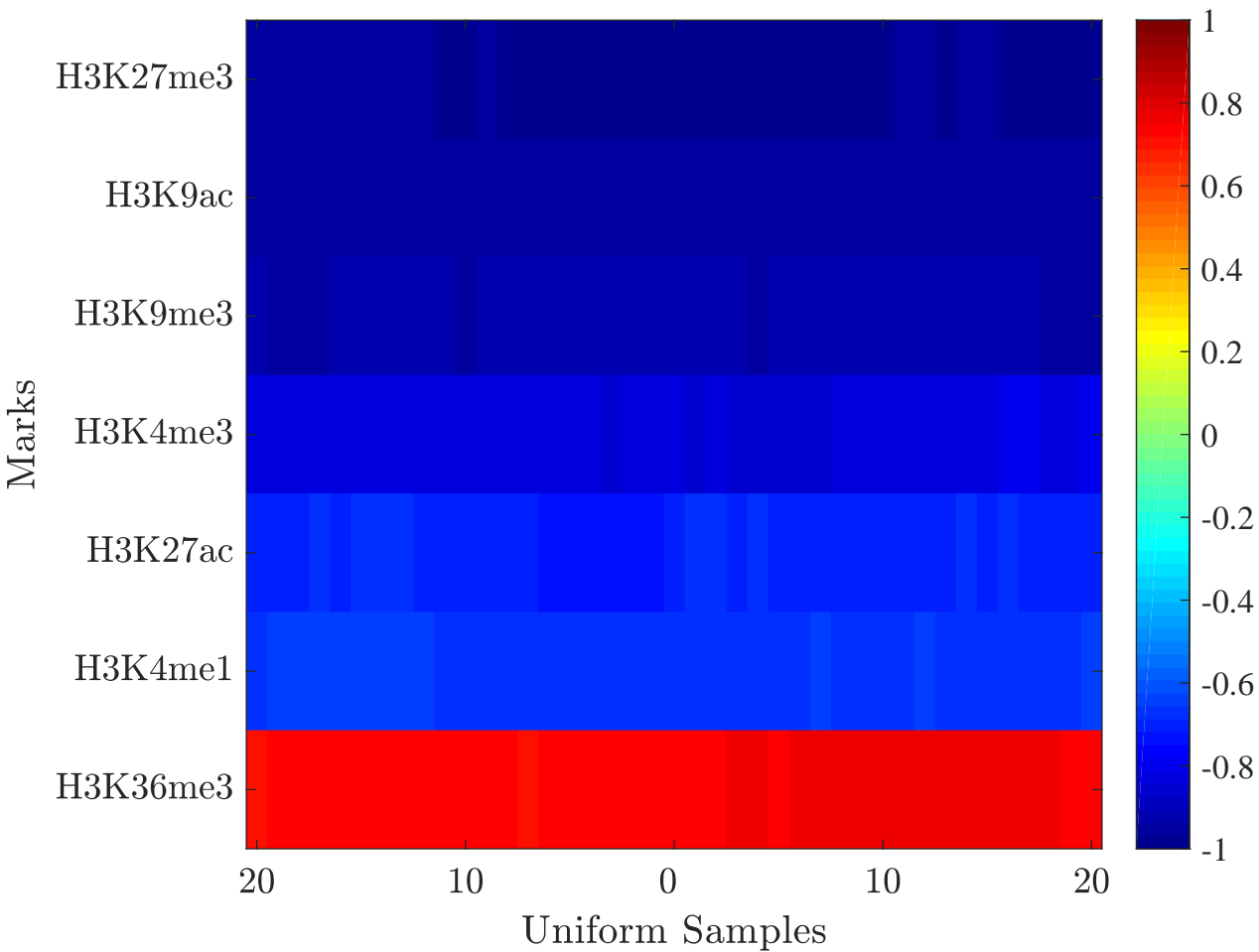

Supplement: Supplementary file 7 — HebbPlots of coding regions of active genes. This compressed file (.tar.gz) includes HebbPlots of genes active in 57 tissues/cell types. (TAR 2696 kb) [file 12859_2018_2312_MOESM7_ESM.tar › file8/E047.pdf]

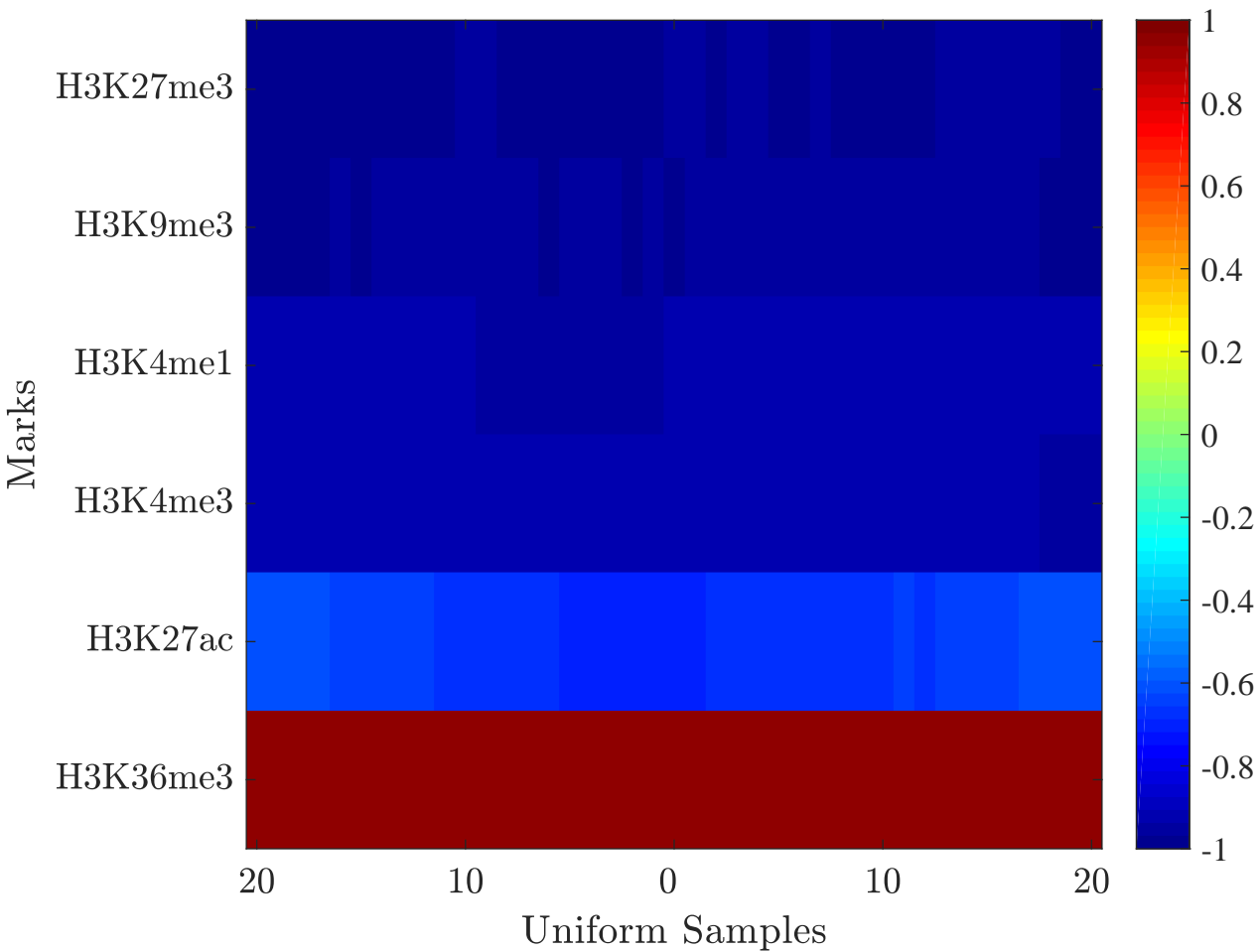

Supplement: Supplementary file 7 — HebbPlots of coding regions of active genes. This compressed file (.tar.gz) includes HebbPlots of genes active in 57 tissues/cell types. (TAR 2696 kb) [file 12859_2018_2312_MOESM7_ESM.tar › file8/E050.pdf]

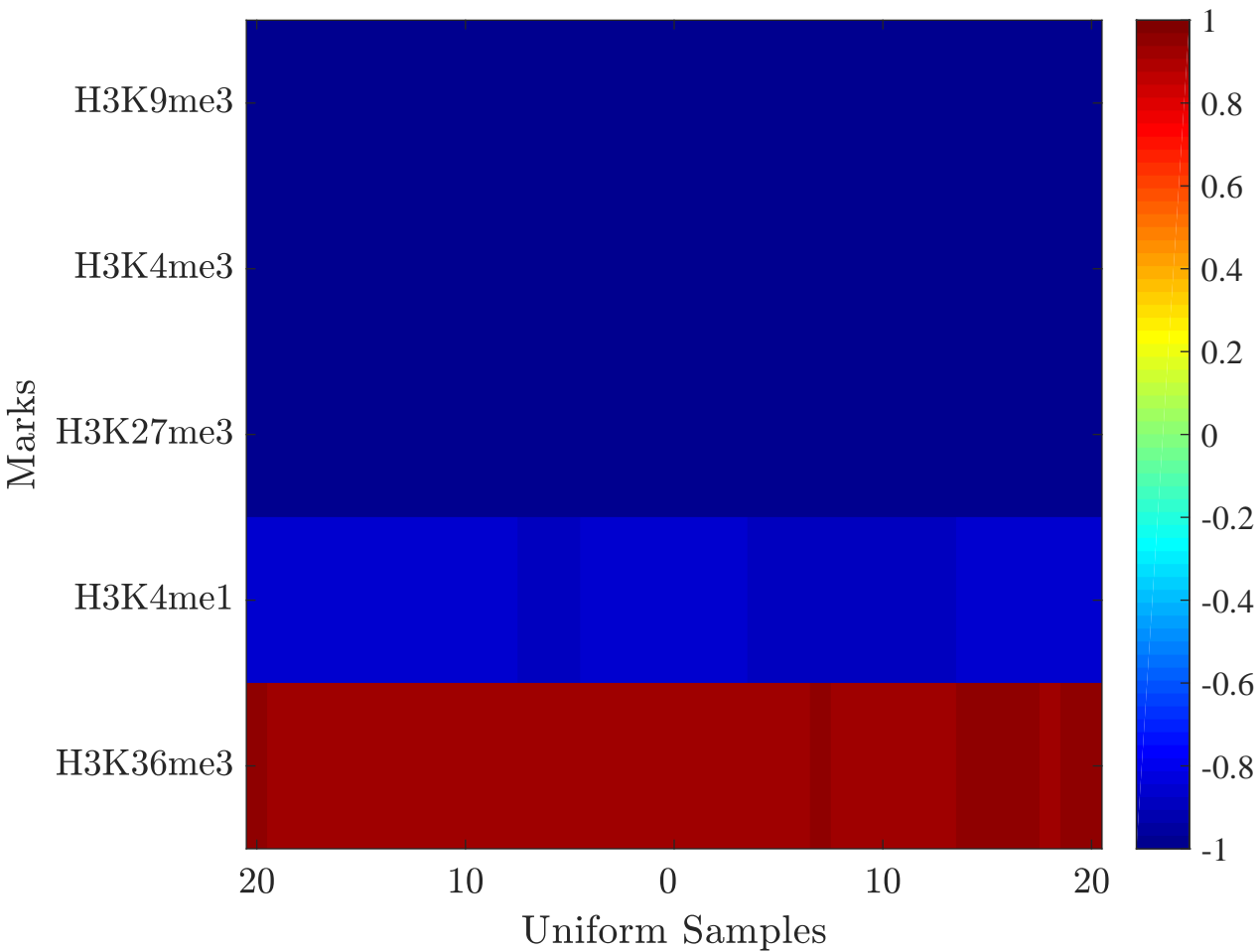

Supplement: Supplementary file 7 — HebbPlots of coding regions of active genes. This compressed file (.tar.gz) includes HebbPlots of genes active in 57 tissues/cell types. (TAR 2696 kb) [file 12859_2018_2312_MOESM7_ESM.tar › file8/E053.pdf]

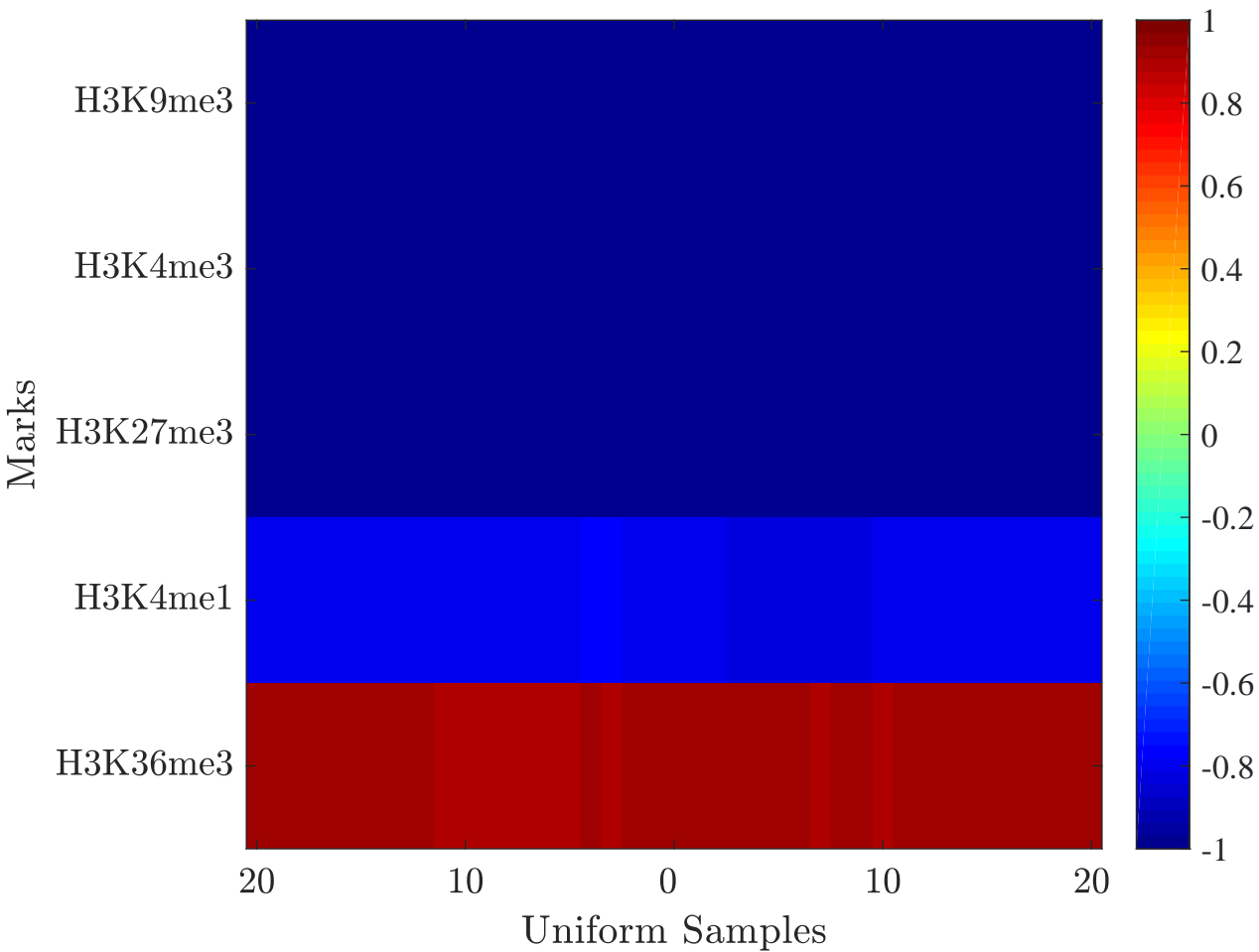

Supplement: Supplementary file 7 — HebbPlots of coding regions of active genes. This compressed file (.tar.gz) includes HebbPlots of genes active in 57 tissues/cell types. (TAR 2696 kb) [file 12859_2018_2312_MOESM7_ESM.tar › file8/E054.pdf]

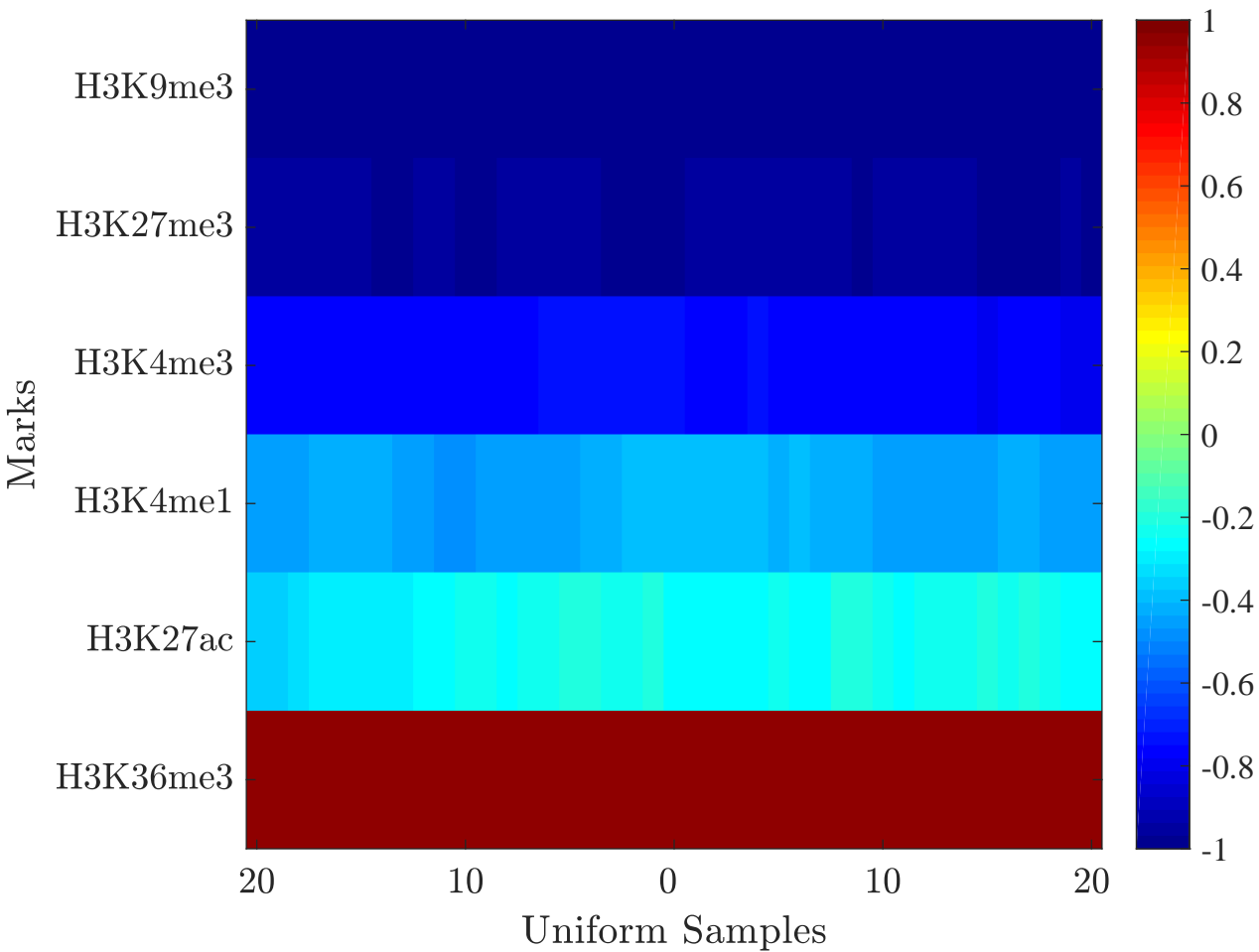

Supplement: Supplementary file 7 — HebbPlots of coding regions of active genes. This compressed file (.tar.gz) includes HebbPlots of genes active in 57 tissues/cell types. (TAR 2696 kb) [file 12859_2018_2312_MOESM7_ESM.tar › file8/E055.pdf]

Marks

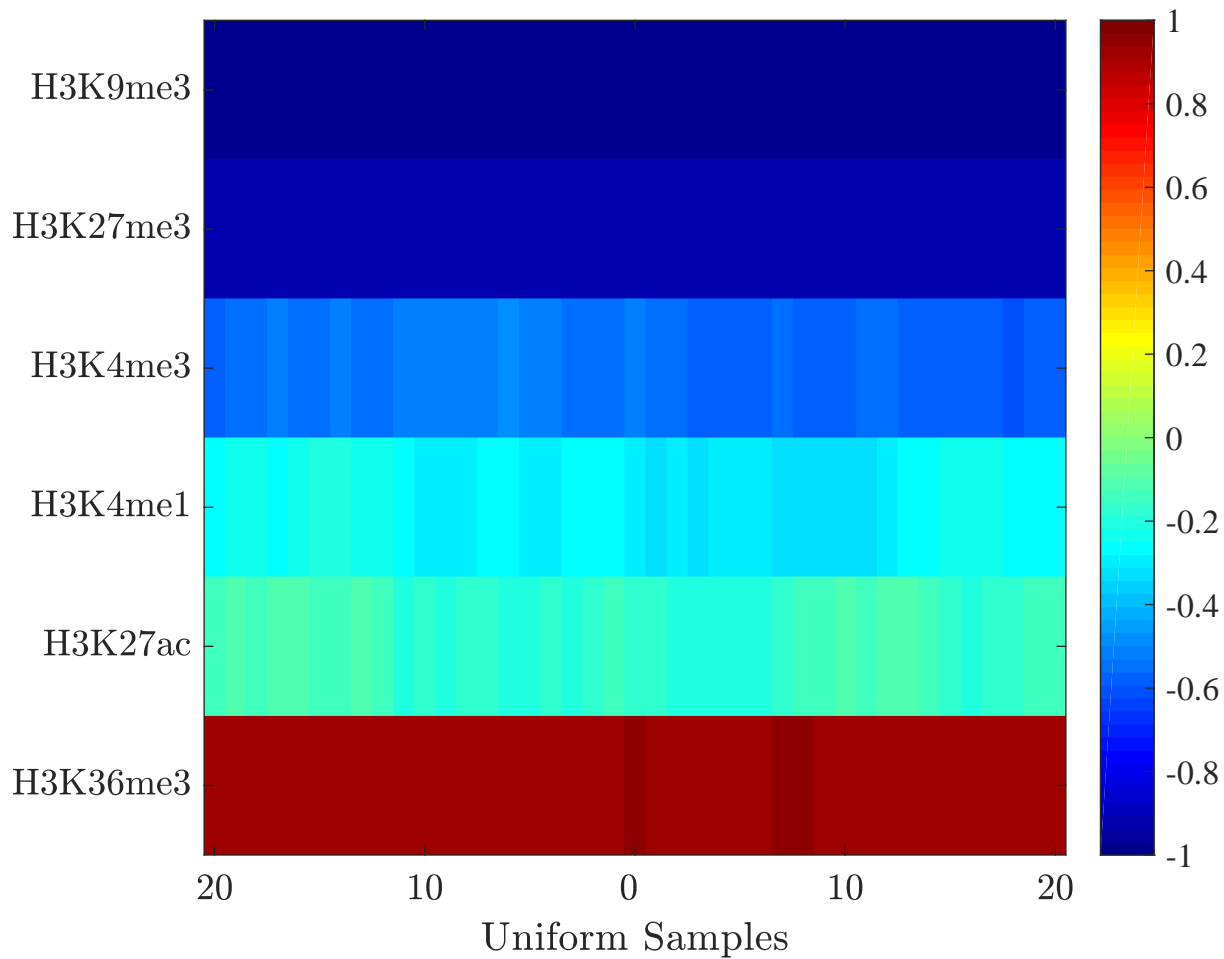

Supplement: Supplementary file 7 — HebbPlots of coding regions of active genes. This compressed file (.tar.gz) includes HebbPlots of genes active in 57 tissues/cell types. (TAR 2696 kb) [file 12859_2018_2312_MOESM7_ESM.tar › file8/E056.pdf]

Marks

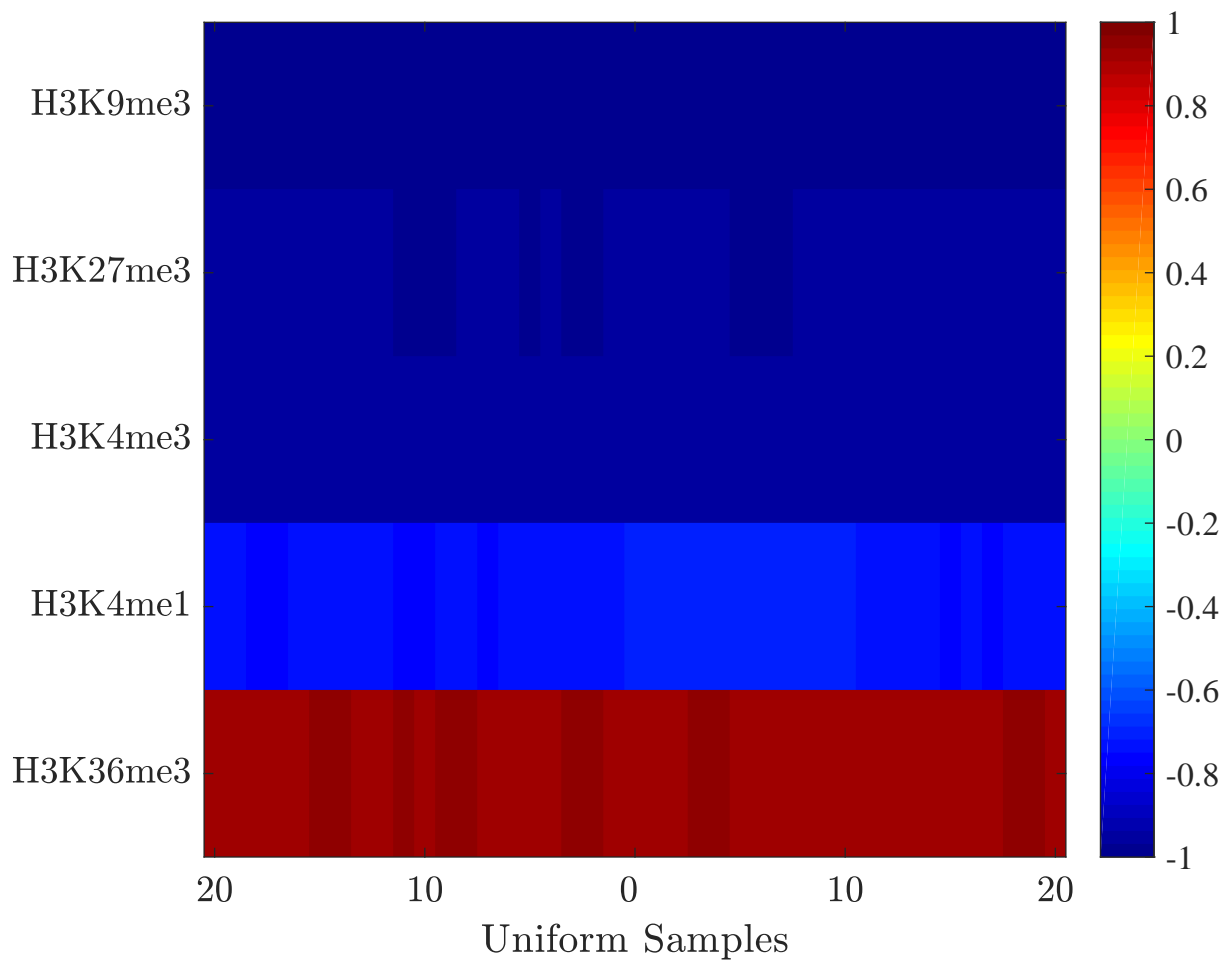

Supplement: Supplementary file 7 — HebbPlots of coding regions of active genes. This compressed file (.tar.gz) includes HebbPlots of genes active in 57 tissues/cell types. (TAR 2696 kb) [file 12859_2018_2312_MOESM7_ESM.tar › file8/E057.pdf]

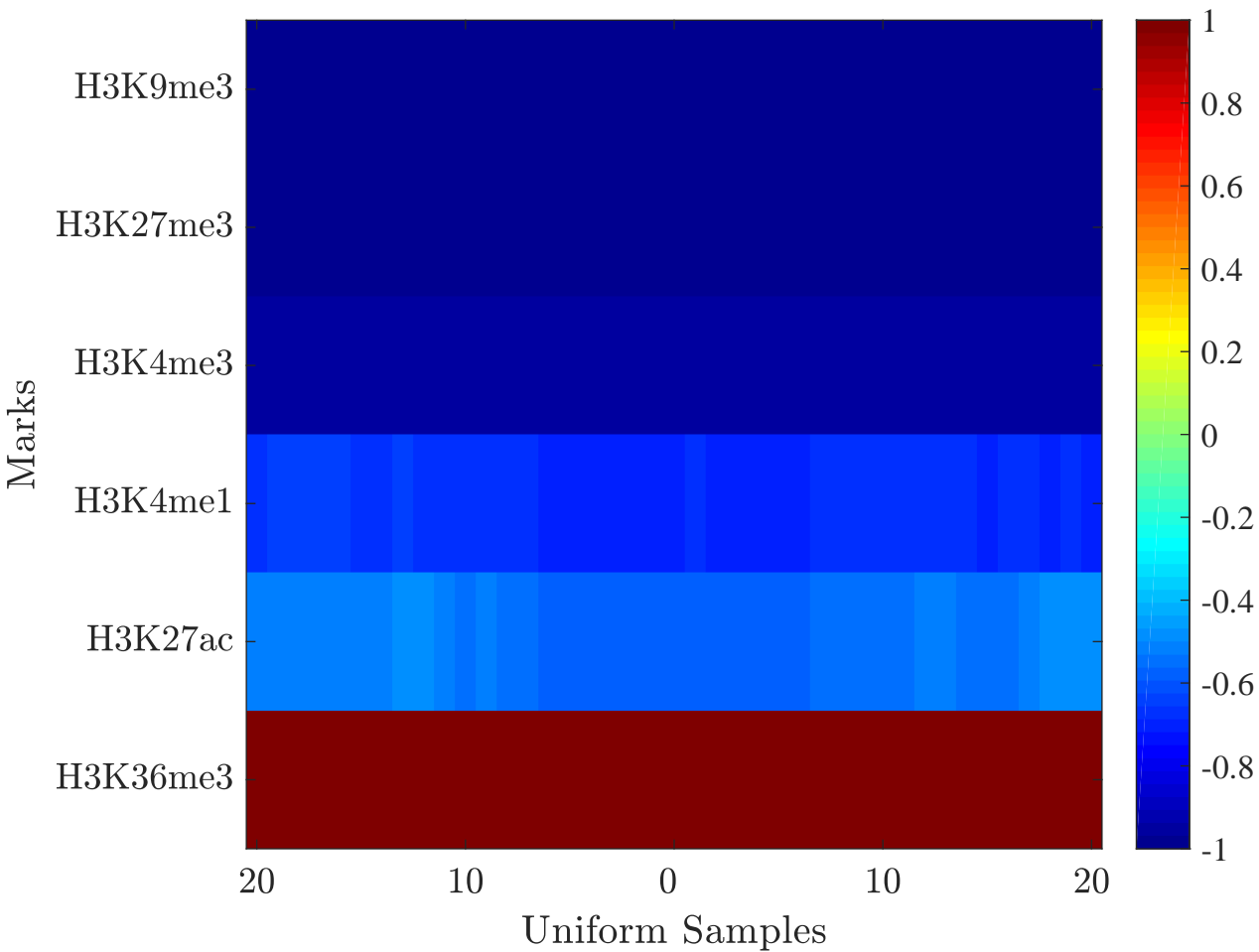

Supplement: Supplementary file 7 — HebbPlots of coding regions of active genes. This compressed file (.tar.gz) includes HebbPlots of genes active in 57 tissues/cell types. (TAR 2696 kb) [file 12859_2018_2312_MOESM7_ESM.tar › file8/E058.pdf]

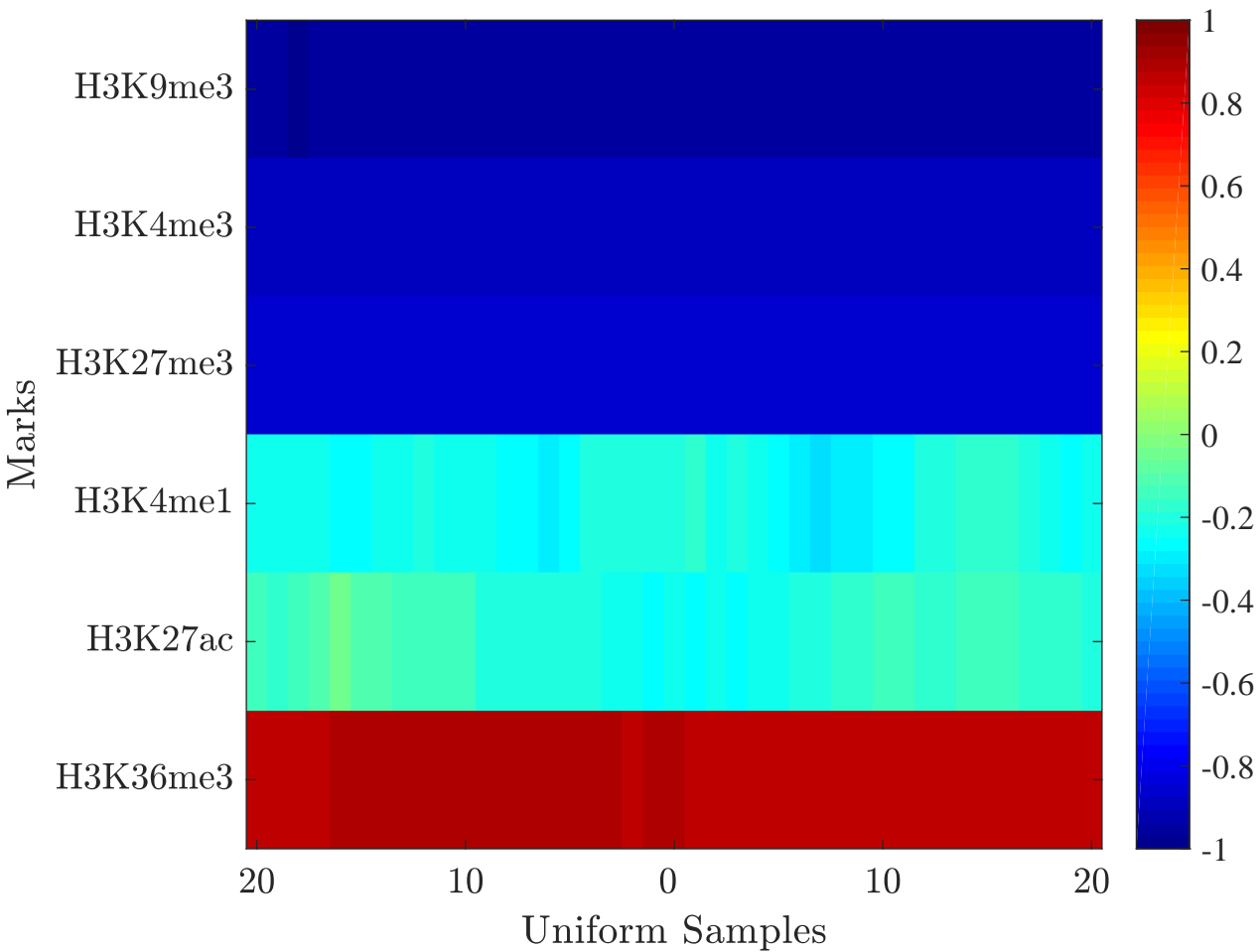

Supplement: Supplementary file 7 — HebbPlots of coding regions of active genes. This compressed file (.tar.gz) includes HebbPlots of genes active in 57 tissues/cell types. (TAR 2696 kb) [file 12859_2018_2312_MOESM7_ESM.tar › file8/E059.pdf]

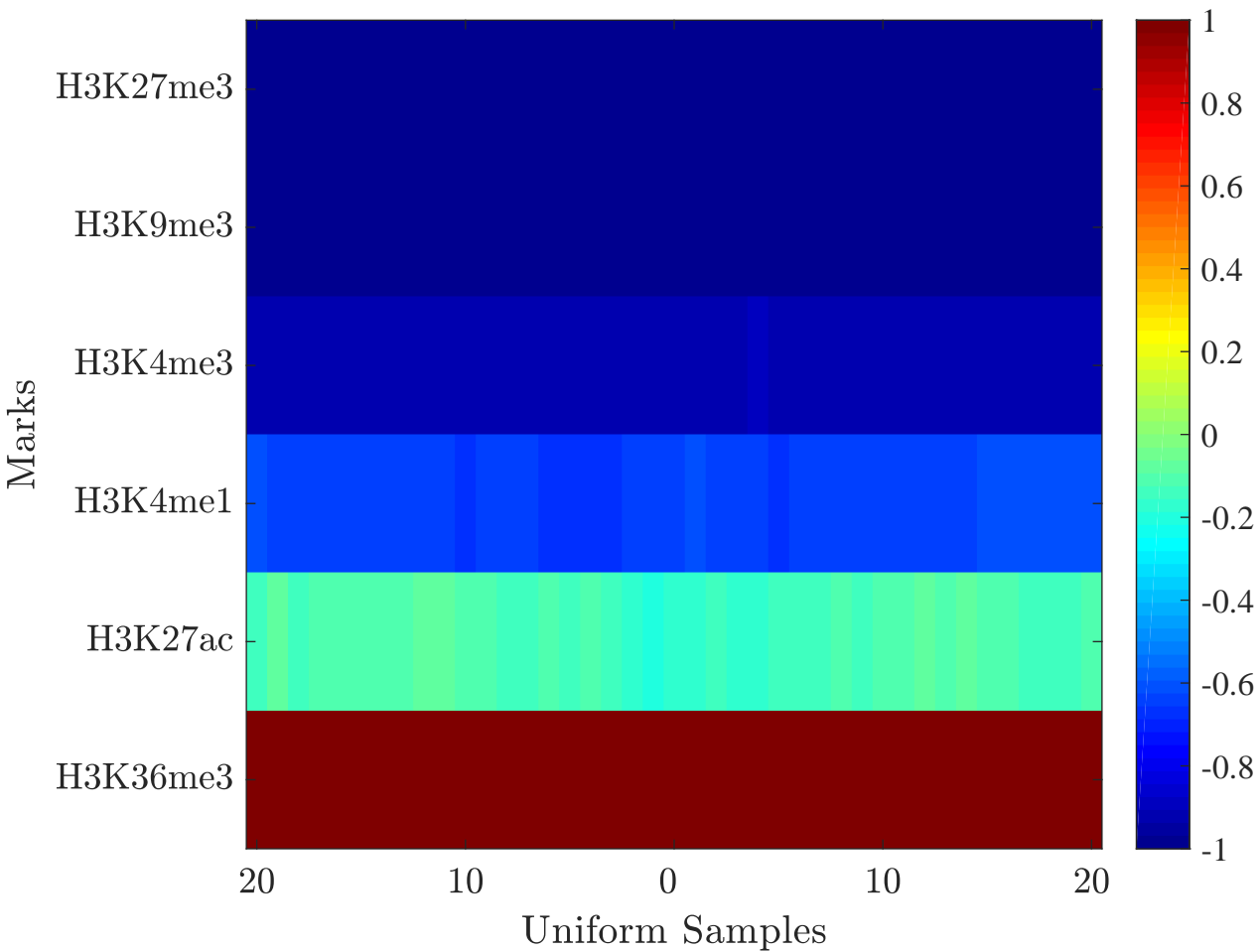

Supplement: Supplementary file 7 — HebbPlots of coding regions of active genes. This compressed file (.tar.gz) includes HebbPlots of genes active in 57 tissues/cell types. (TAR 2696 kb) [file 12859_2018_2312_MOESM7_ESM.tar › file8/E061.pdf]

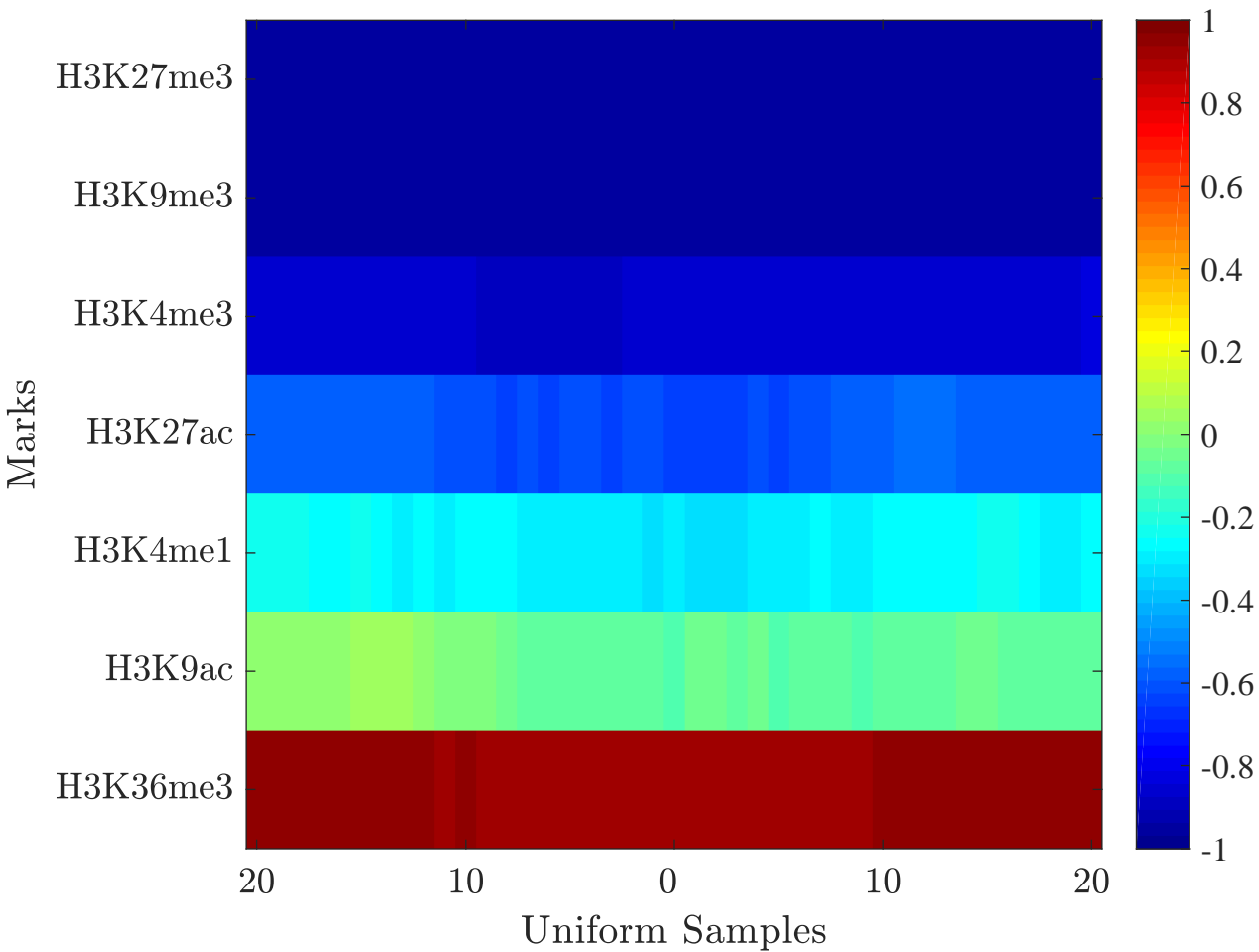

Supplement: Supplementary file 7 — HebbPlots of coding regions of active genes. This compressed file (.tar.gz) includes HebbPlots of genes active in 57 tissues/cell types. (TAR 2696 kb) [file 12859_2018_2312_MOESM7_ESM.tar › file8/E062.pdf]

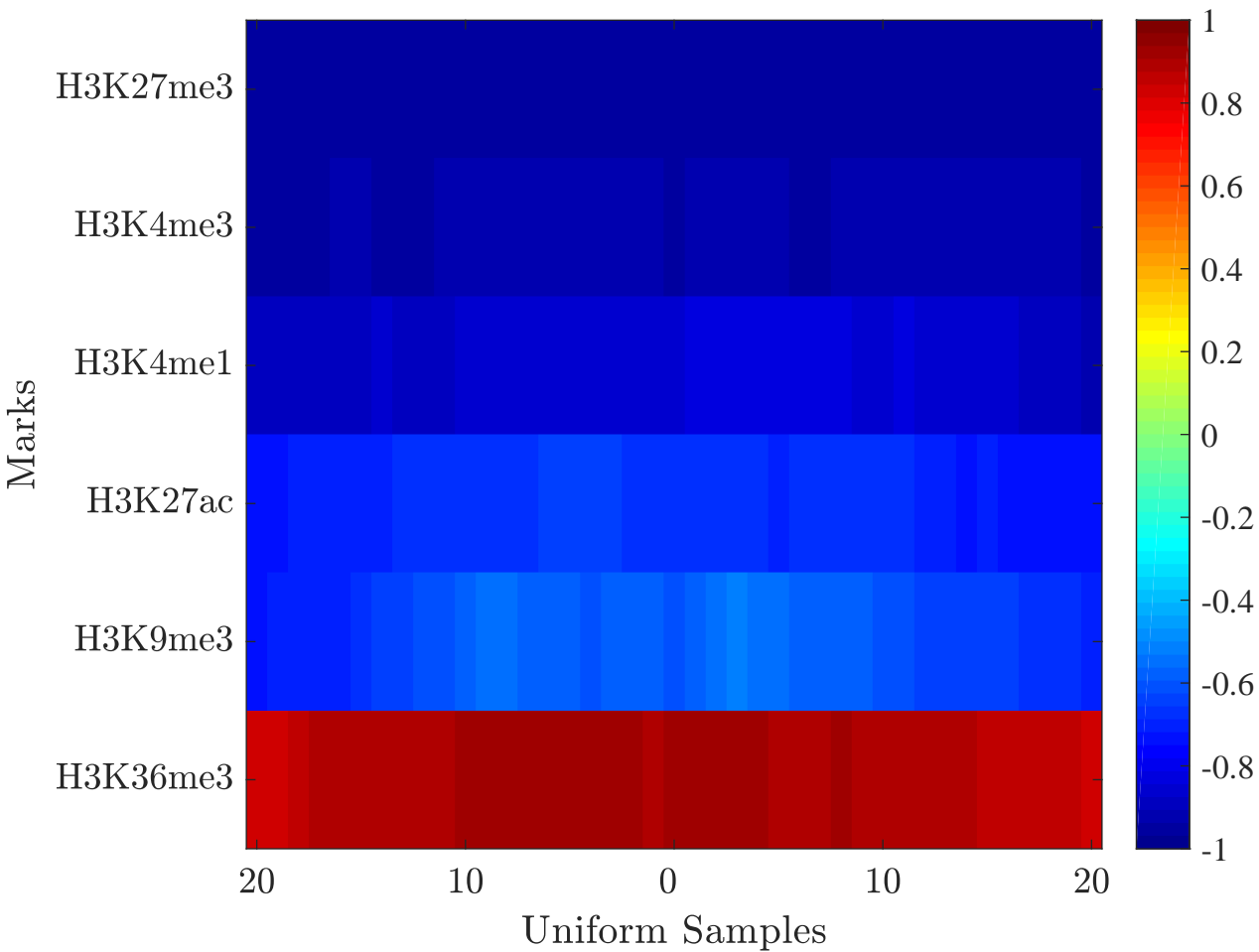

Supplement: Supplementary file 7 — HebbPlots of coding regions of active genes. This compressed file (.tar.gz) includes HebbPlots of genes active in 57 tissues/cell types. (TAR 2696 kb) [file 12859_2018_2312_MOESM7_ESM.tar › file8/E065.pdf]

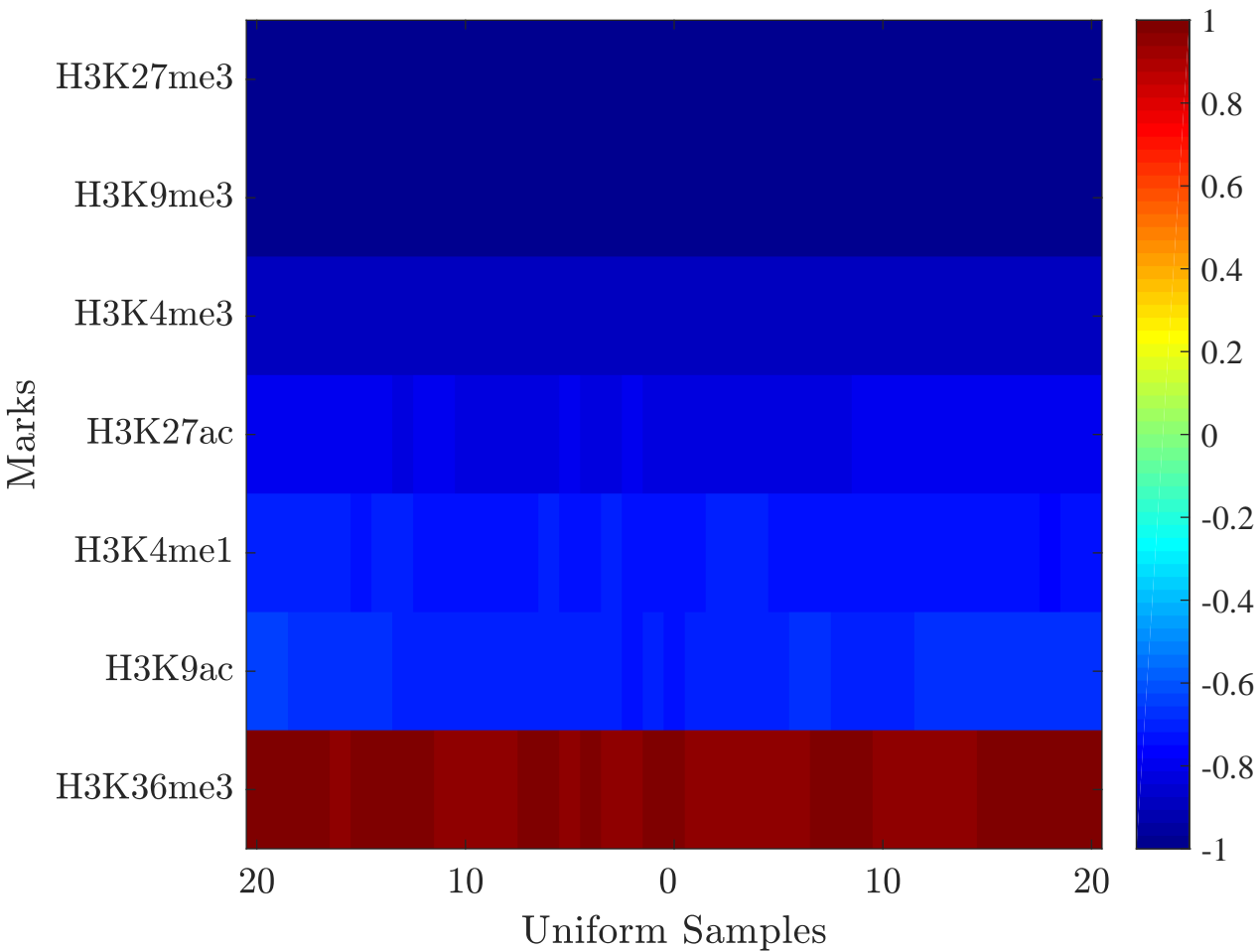

Supplement: Supplementary file 7 — HebbPlots of coding regions of active genes. This compressed file (.tar.gz) includes HebbPlots of genes active in 57 tissues/cell types. (TAR 2696 kb) [file 12859_2018_2312_MOESM7_ESM.tar › file8/E066.pdf]

Marks

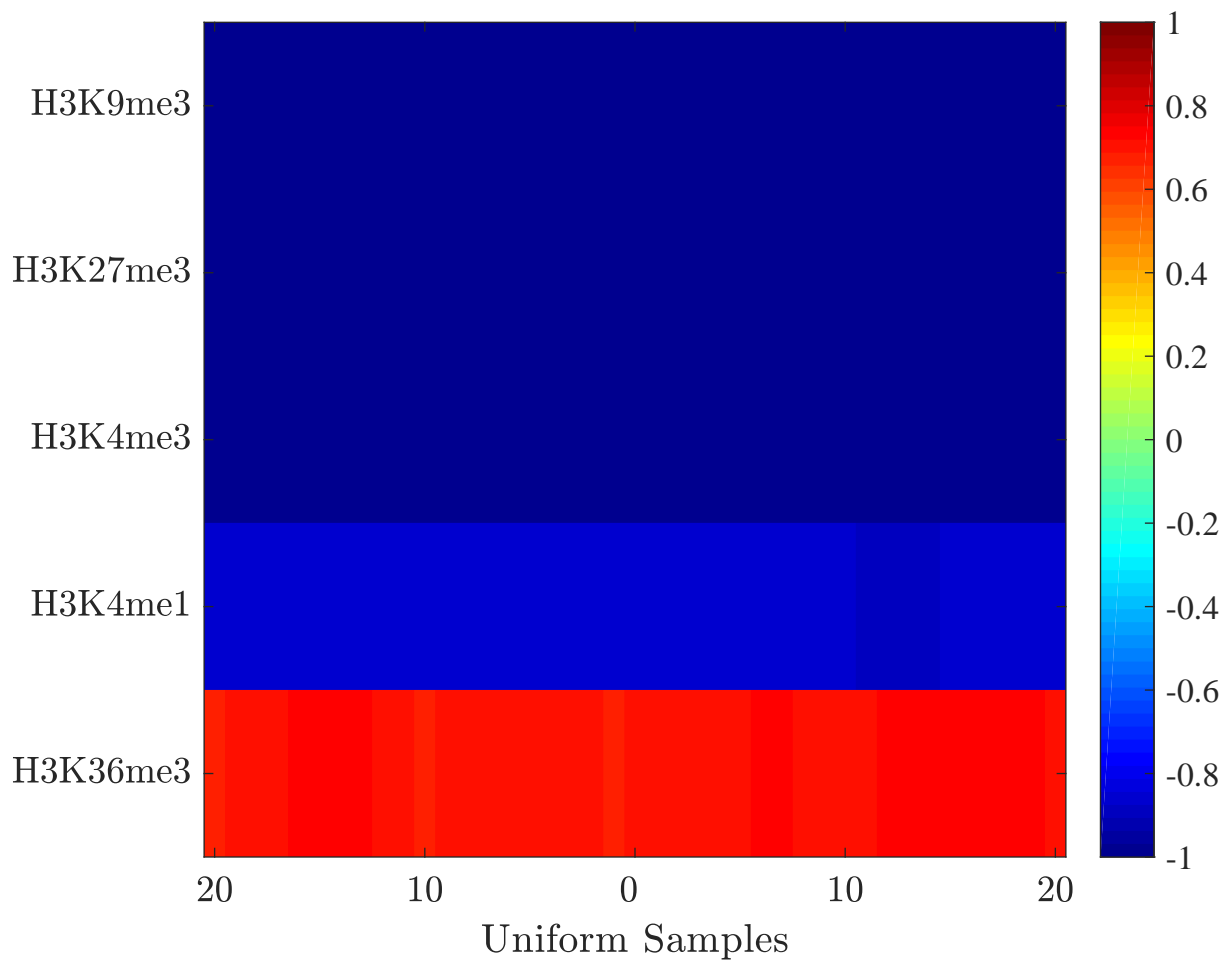

Supplement: Supplementary file 7 — HebbPlots of coding regions of active genes. This compressed file (.tar.gz) includes HebbPlots of genes active in 57 tissues/cell types. (TAR 2696 kb) [file 12859_2018_2312_MOESM7_ESM.tar › file8/E070.pdf]

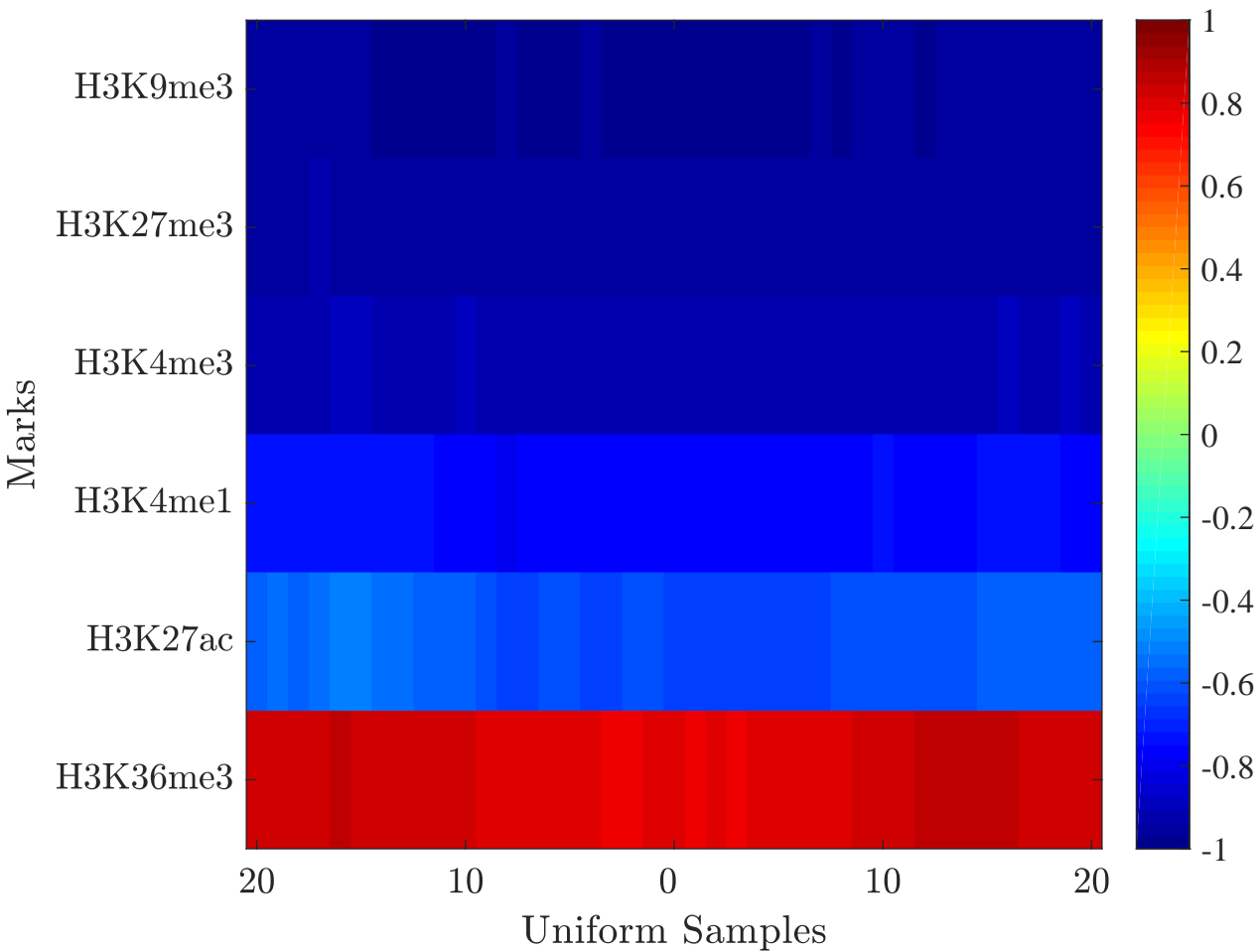

Supplement: Supplementary file 7 — HebbPlots of coding regions of active genes. This compressed file (.tar.gz) includes HebbPlots of genes active in 57 tissues/cell types. (TAR 2696 kb) [file 12859_2018_2312_MOESM7_ESM.tar › file8/E071.pdf]

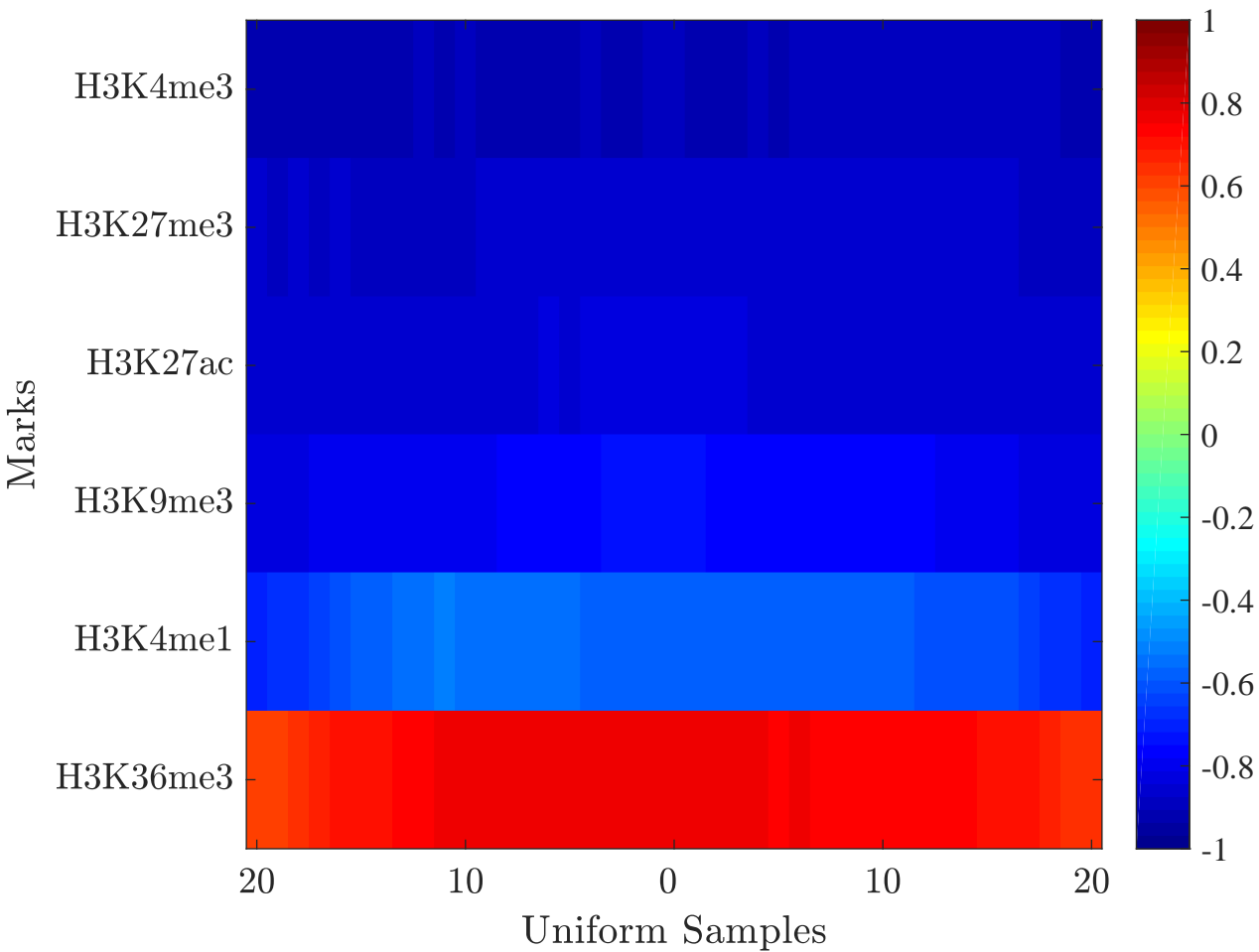

Supplement: Supplementary file 7 — HebbPlots of coding regions of active genes. This compressed file (.tar.gz) includes HebbPlots of genes active in 57 tissues/cell types. (TAR 2696 kb) [file 12859_2018_2312_MOESM7_ESM.tar › file8/E079.pdf]

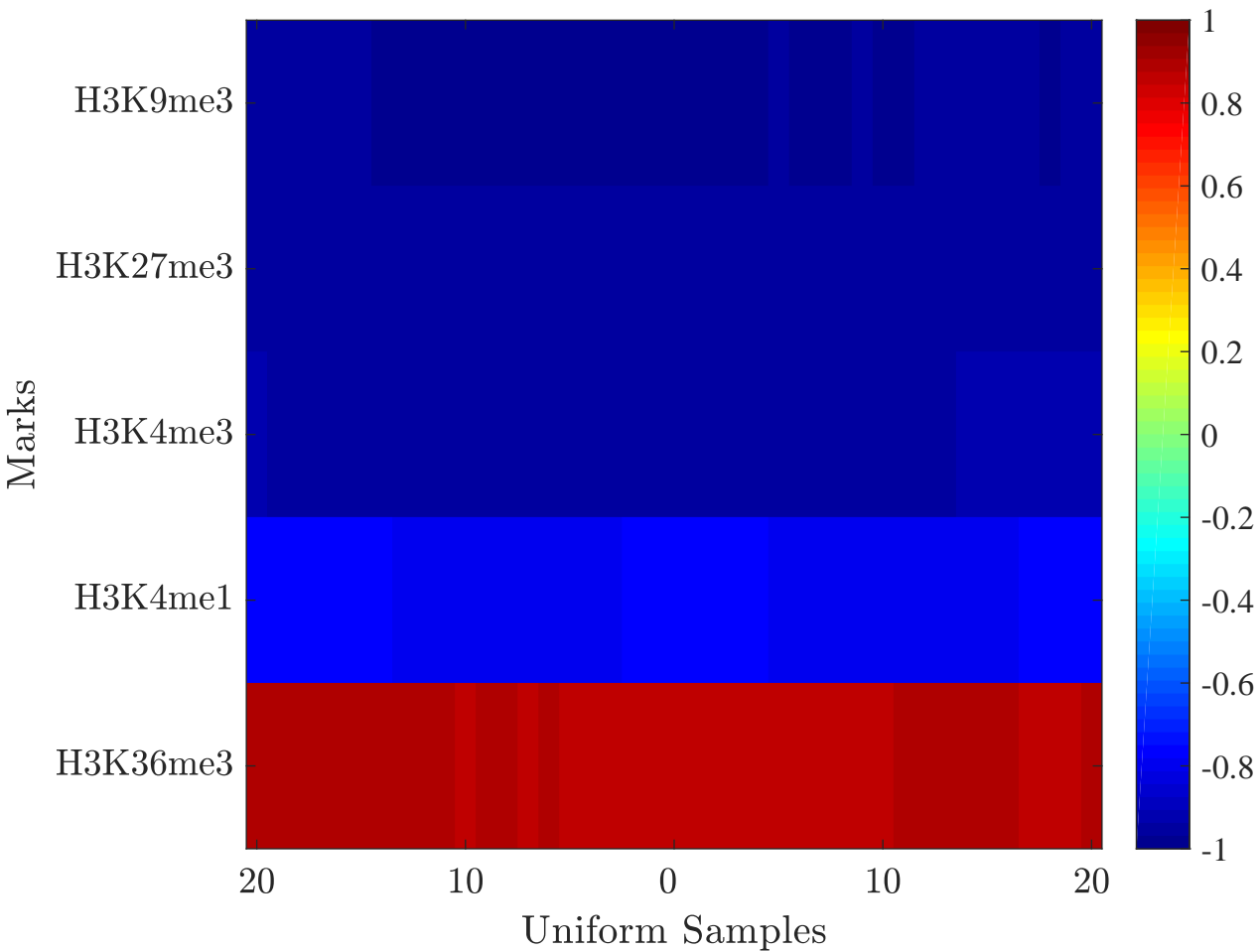

Supplement: Supplementary file 7 — HebbPlots of coding regions of active genes. This compressed file (.tar.gz) includes HebbPlots of genes active in 57 tissues/cell types. (TAR 2696 kb) [file 12859_2018_2312_MOESM7_ESM.tar › file8/E082.pdf]

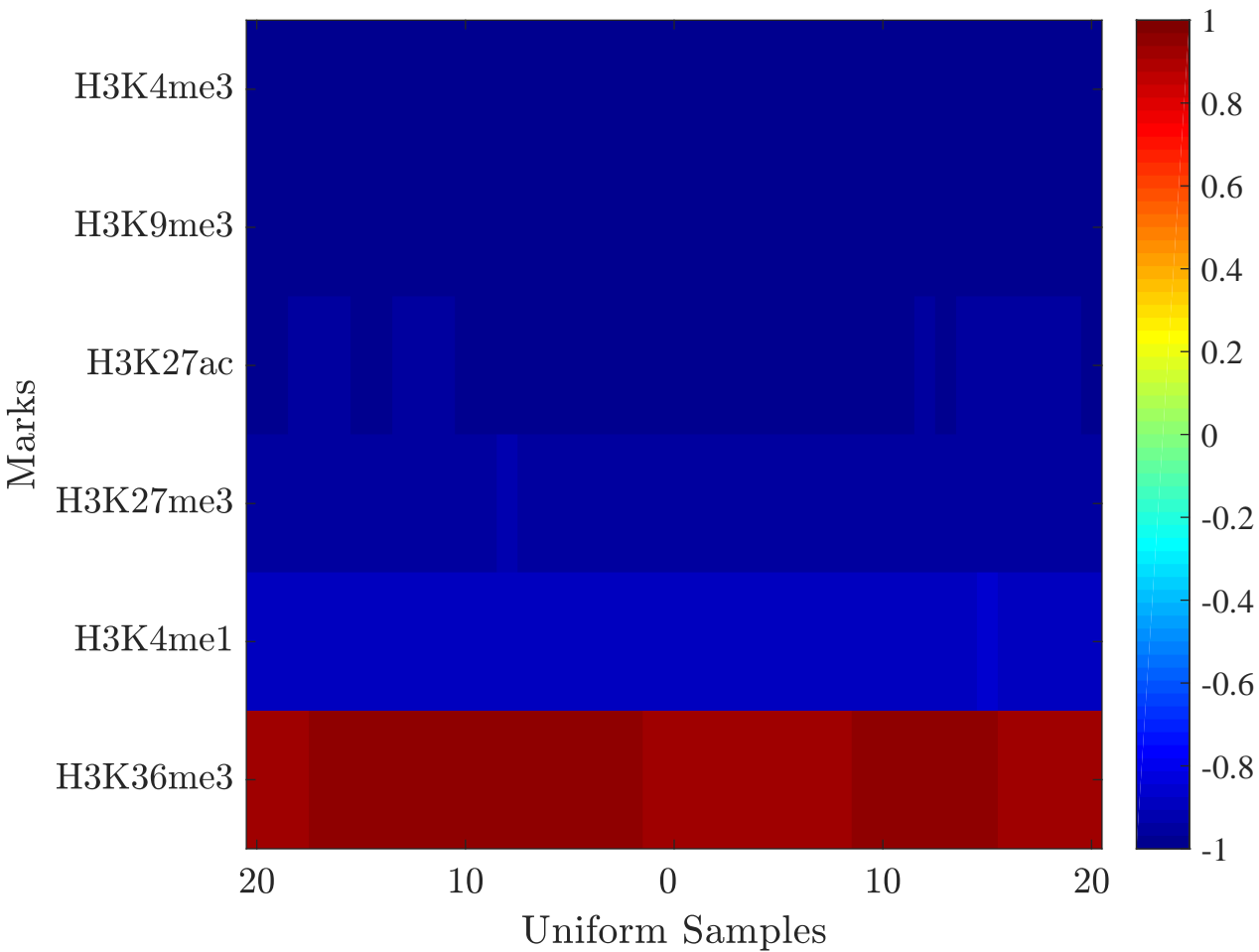

Supplement: Supplementary file 7 — HebbPlots of coding regions of active genes. This compressed file (.tar.gz) includes HebbPlots of genes active in 57 tissues/cell types. (TAR 2696 kb) [file 12859_2018_2312_MOESM7_ESM.tar › file8/E084.pdf]

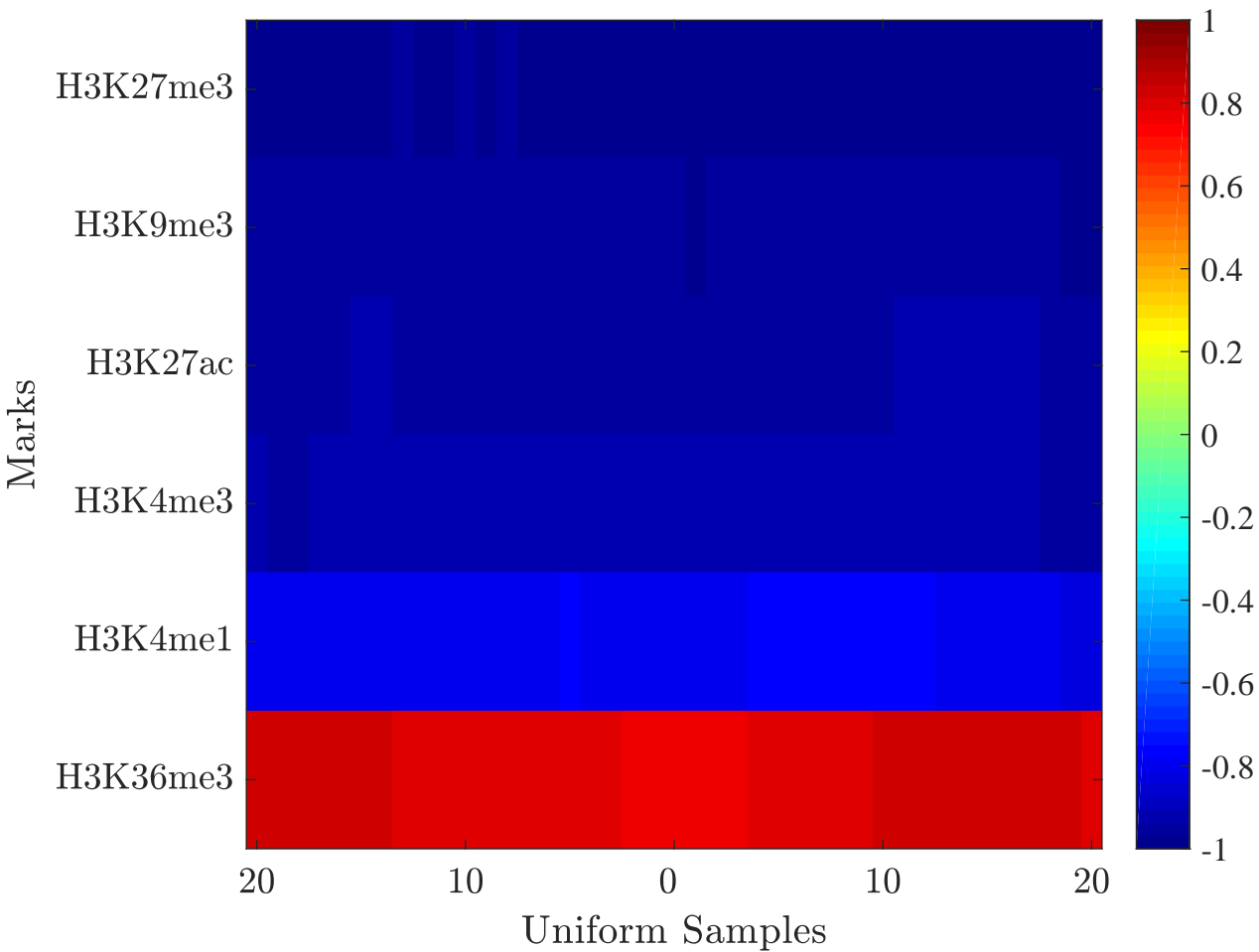

Supplement: Supplementary file 7 — HebbPlots of coding regions of active genes. This compressed file (.tar.gz) includes HebbPlots of genes active in 57 tissues/cell types. (TAR 2696 kb) [file 12859_2018_2312_MOESM7_ESM.tar › file8/E085.pdf]

Marks

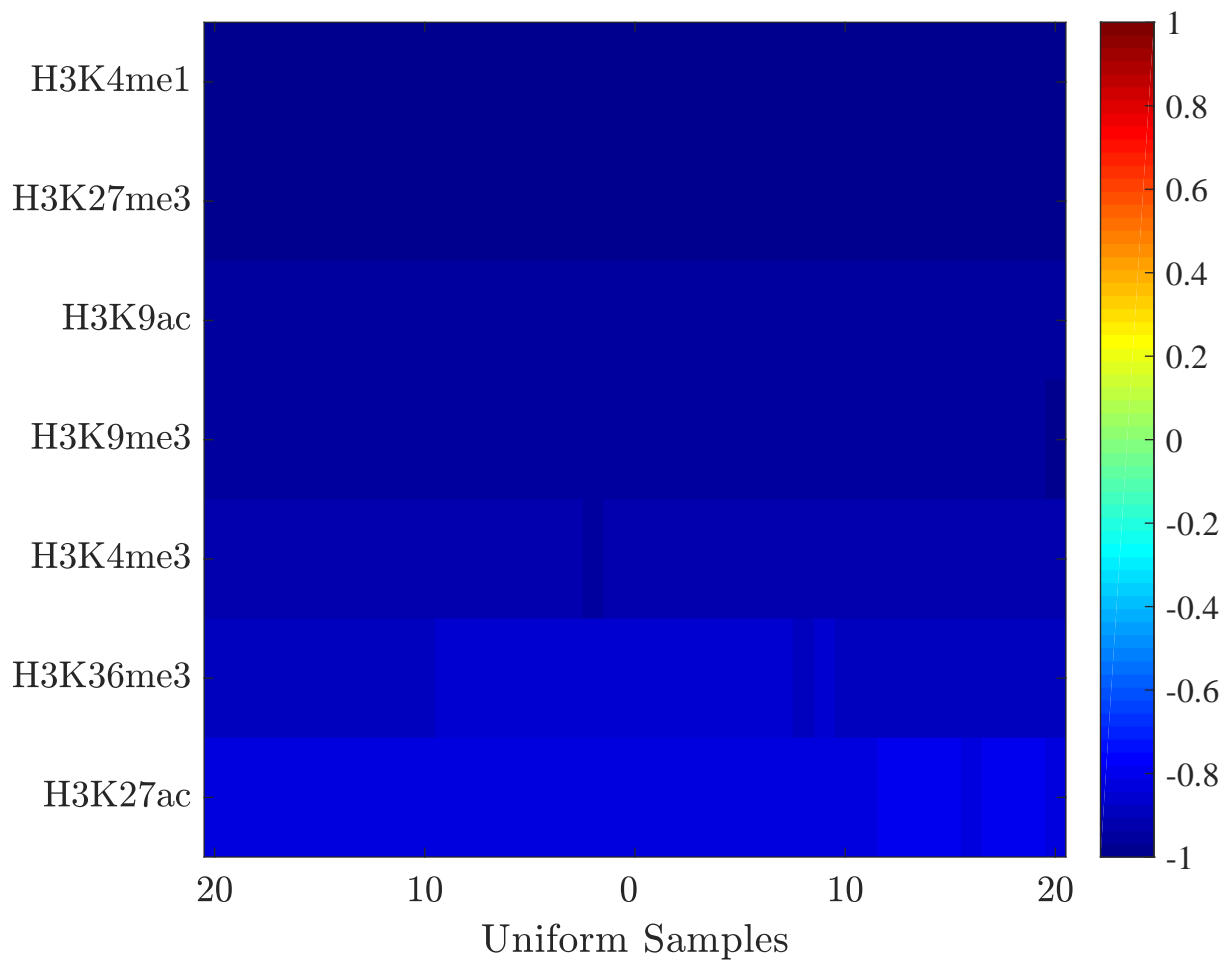

Supplement: Supplementary file 7 — HebbPlots of coding regions of active genes. This compressed file (.tar.gz) includes HebbPlots of genes active in 57 tissues/cell types. (TAR 2696 kb) [file 12859_2018_2312_MOESM7_ESM.tar › file8/E087.pdf]

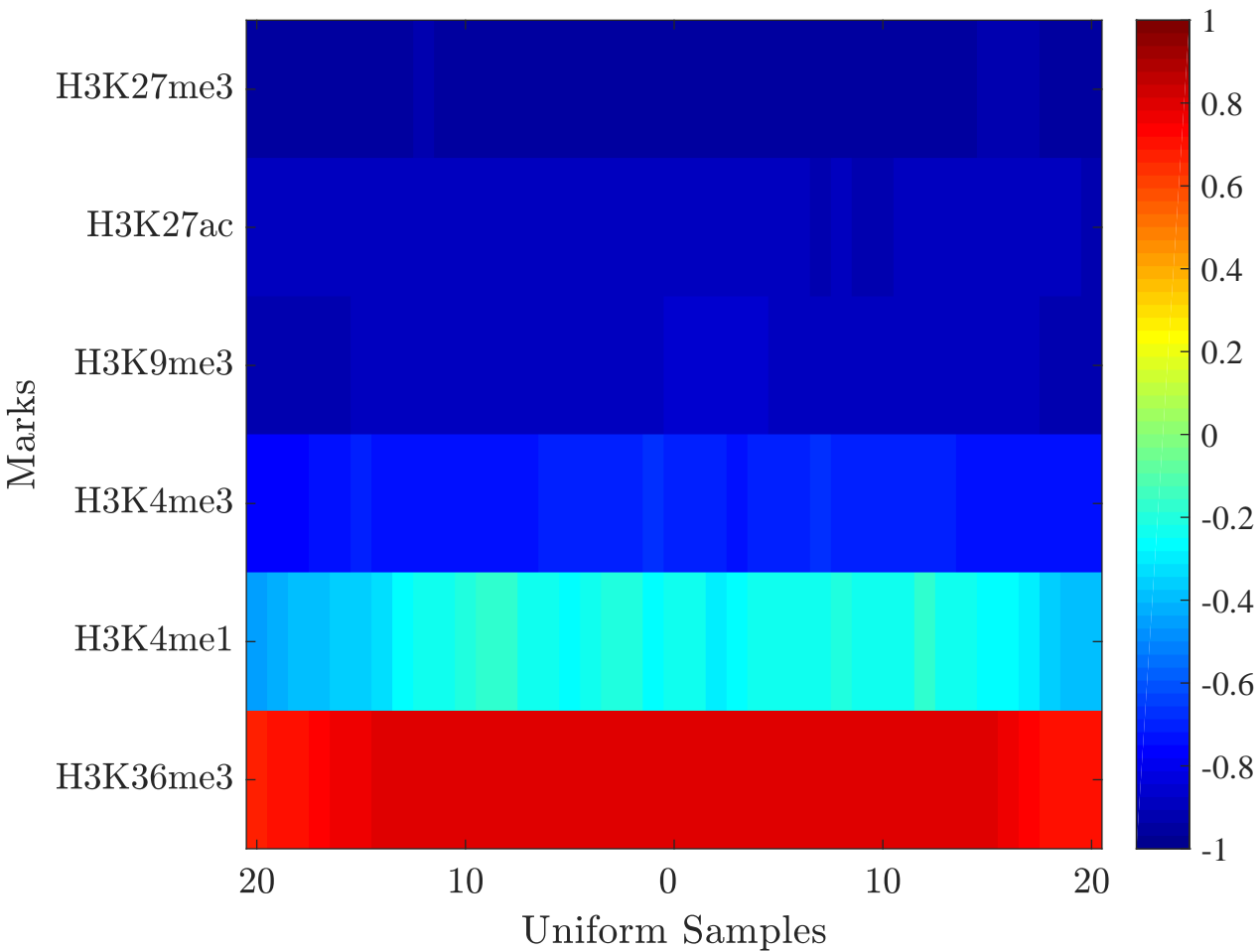

Supplement: Supplementary file 7 — HebbPlots of coding regions of active genes. This compressed file (.tar.gz) includes HebbPlots of genes active in 57 tissues/cell types. (TAR 2696 kb) [file 12859_2018_2312_MOESM7_ESM.tar › file8/E094.pdf]

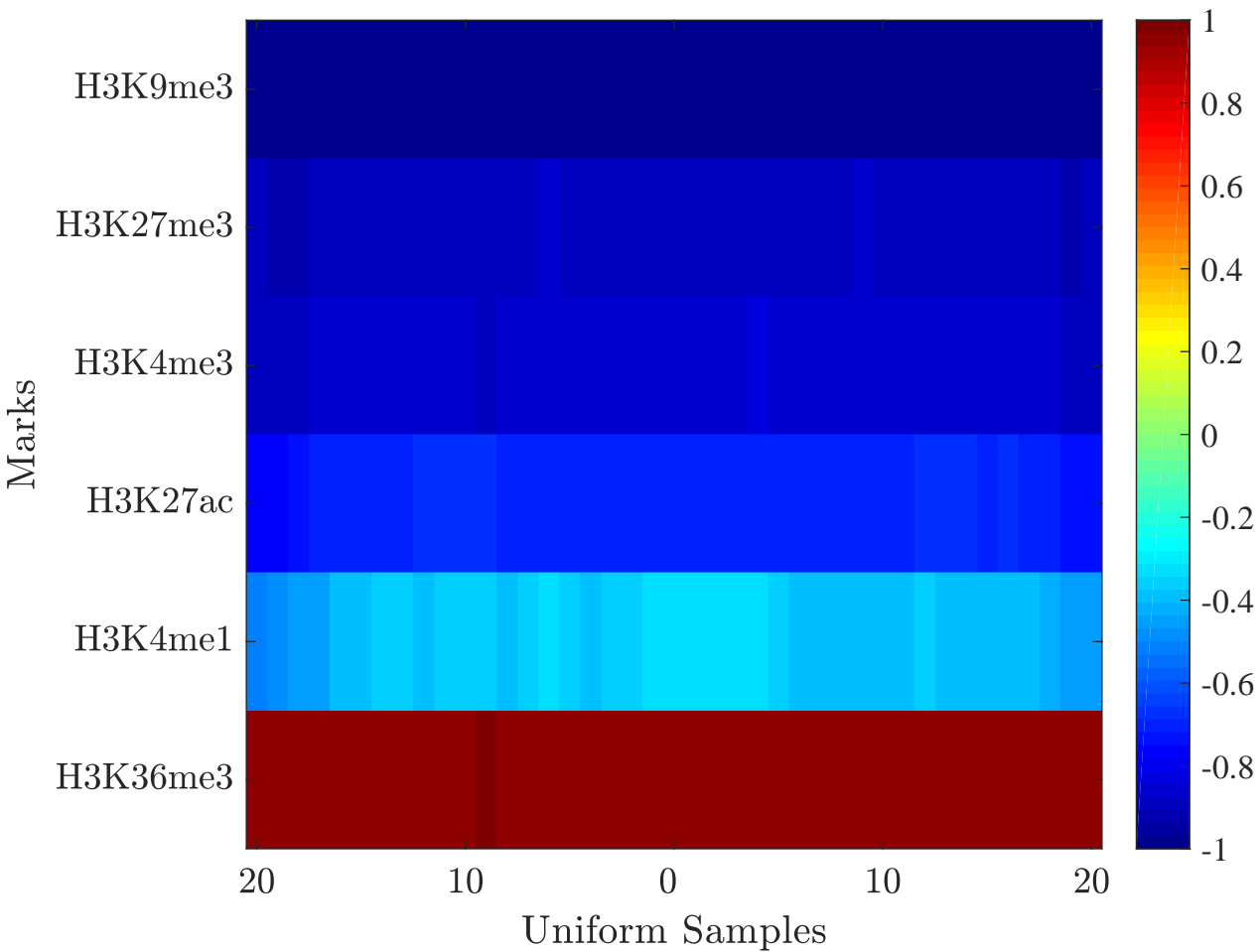

Supplement: Supplementary file 7 — HebbPlots of coding regions of active genes. This compressed file (.tar.gz) includes HebbPlots of genes active in 57 tissues/cell types. (TAR 2696 kb) [file 12859_2018_2312_MOESM7_ESM.tar › file8/E095.pdf]

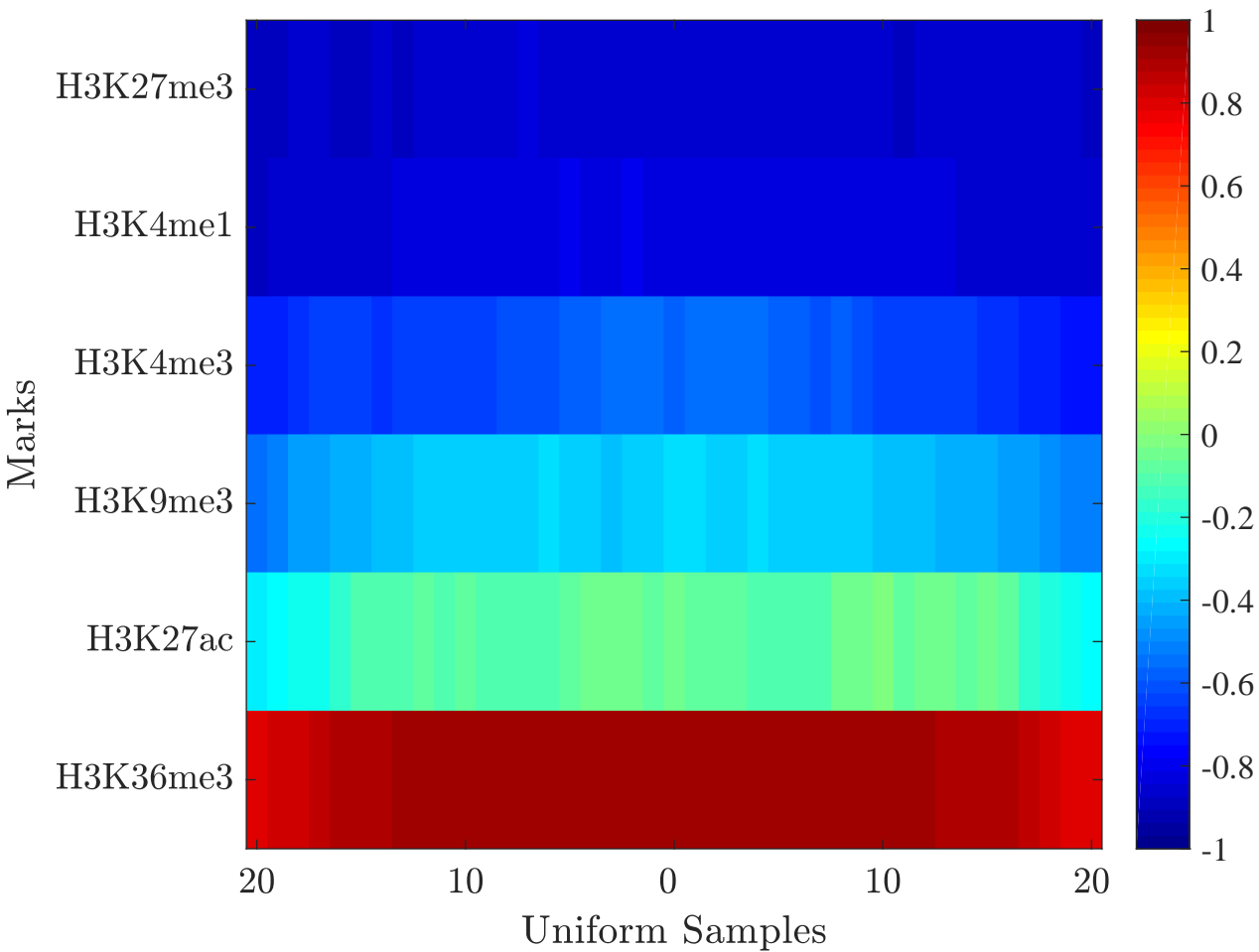

Supplement: Supplementary file 7 — HebbPlots of coding regions of active genes. This compressed file (.tar.gz) includes HebbPlots of genes active in 57 tissues/cell types. (TAR 2696 kb) [file 12859_2018_2312_MOESM7_ESM.tar › file8/E096.pdf]

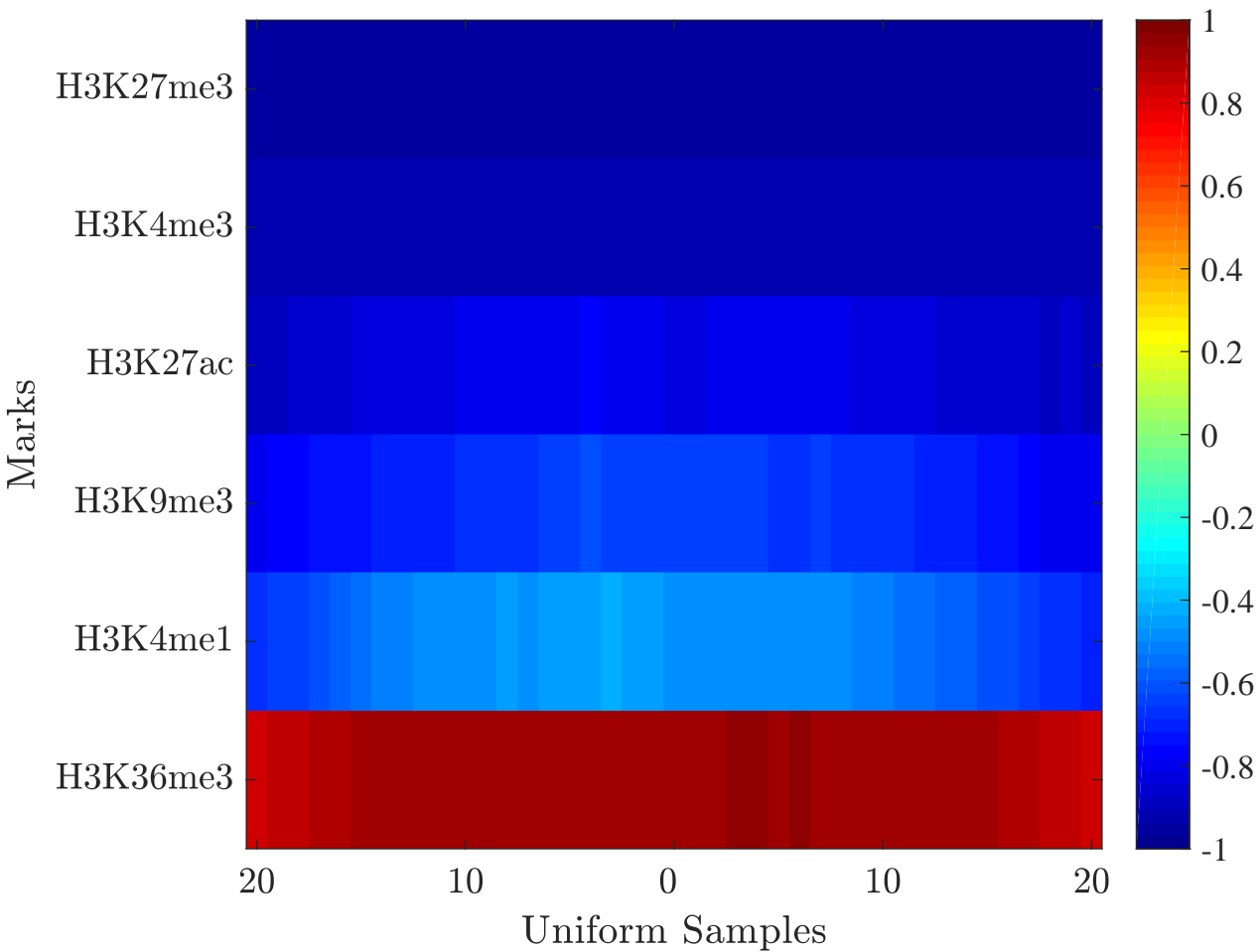

Supplement: Supplementary file 7 — HebbPlots of coding regions of active genes. This compressed file (.tar.gz) includes HebbPlots of genes active in 57 tissues/cell types. (TAR 2696 kb) [file 12859_2018_2312_MOESM7_ESM.tar › file8/E097.pdf]

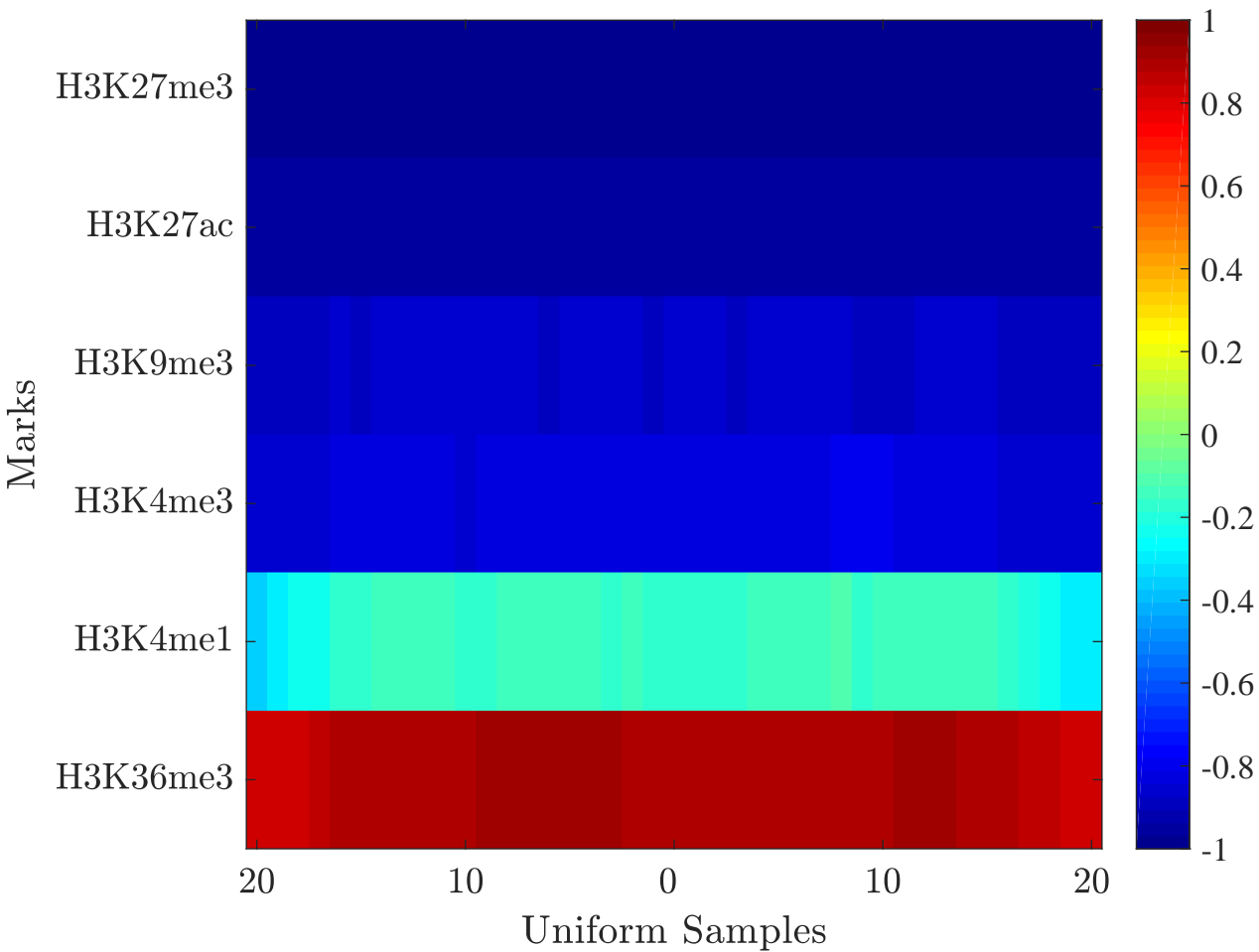

Supplement: Supplementary file 7 — HebbPlots of coding regions of active genes. This compressed file (.tar.gz) includes HebbPlots of genes active in 57 tissues/cell types. (TAR 2696 kb) [file 12859_2018_2312_MOESM7_ESM.tar › file8/E098.pdf]

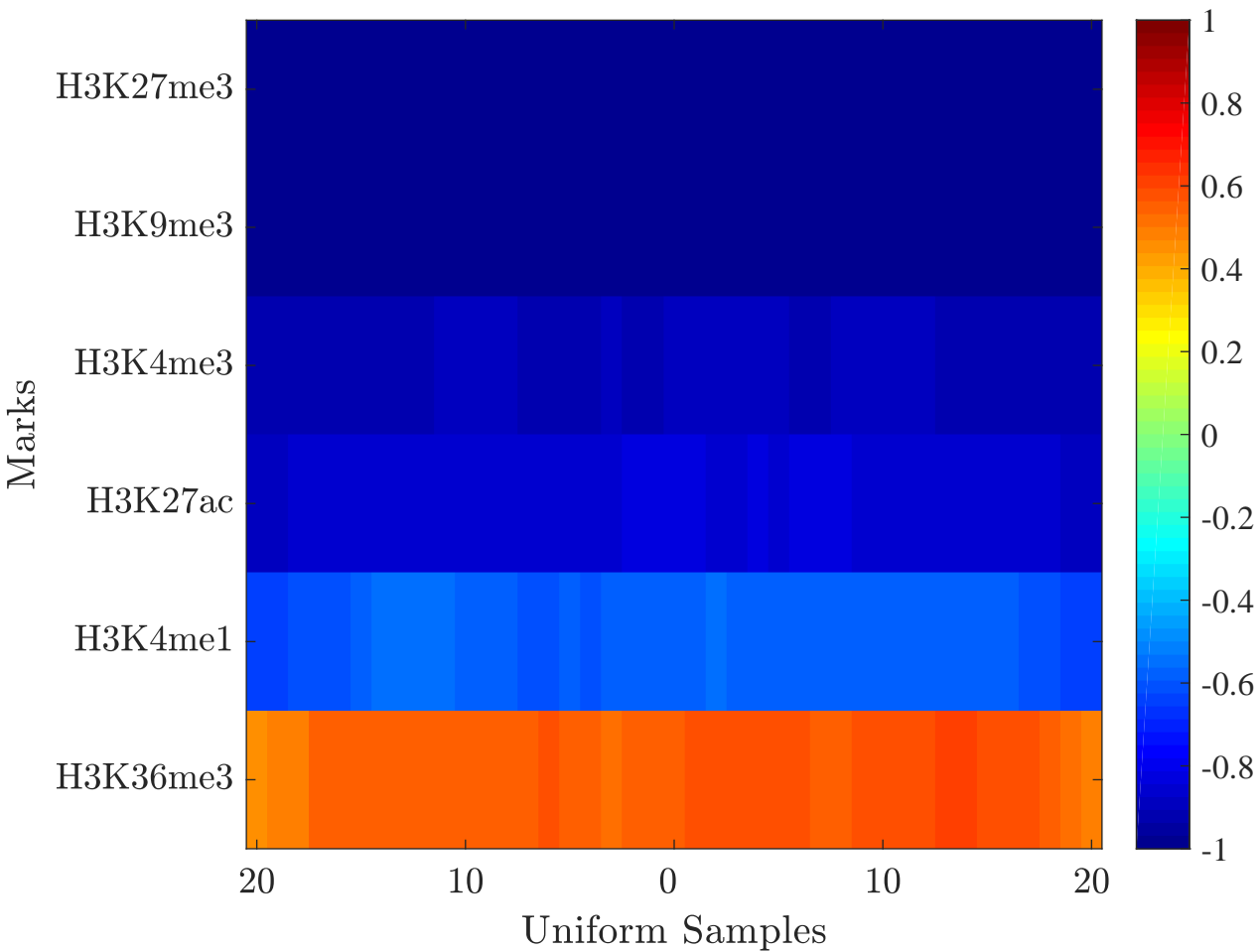

Supplement: Supplementary file 7 — HebbPlots of coding regions of active genes. This compressed file (.tar.gz) includes HebbPlots of genes active in 57 tissues/cell types. (TAR 2696 kb) [file 12859_2018_2312_MOESM7_ESM.tar › file8/E100.pdf]

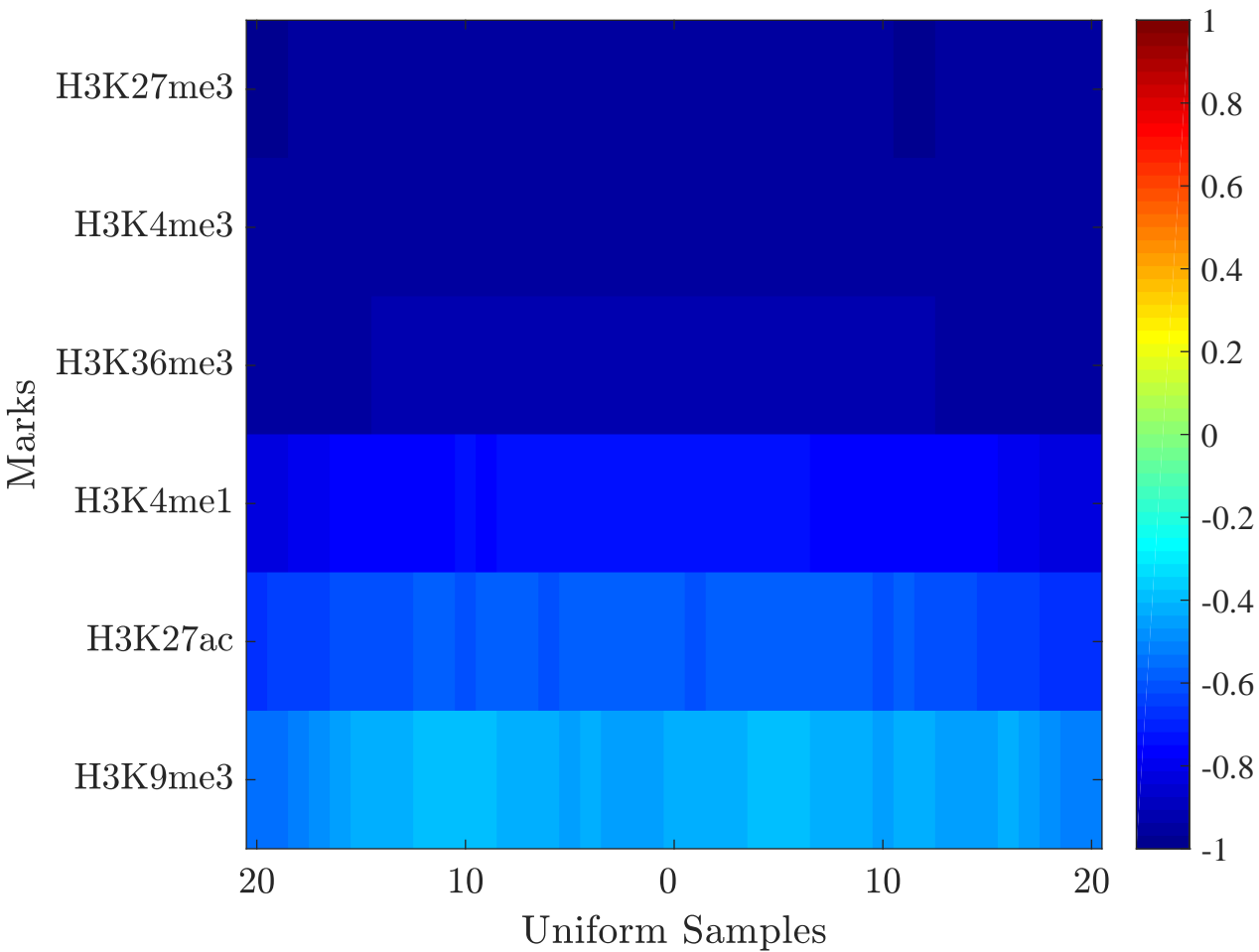

Supplement: Supplementary file 7 — HebbPlots of coding regions of active genes. This compressed file (.tar.gz) includes HebbPlots of genes active in 57 tissues/cell types. (TAR 2696 kb) [file 12859_2018_2312_MOESM7_ESM.tar › file8/E104.pdf]

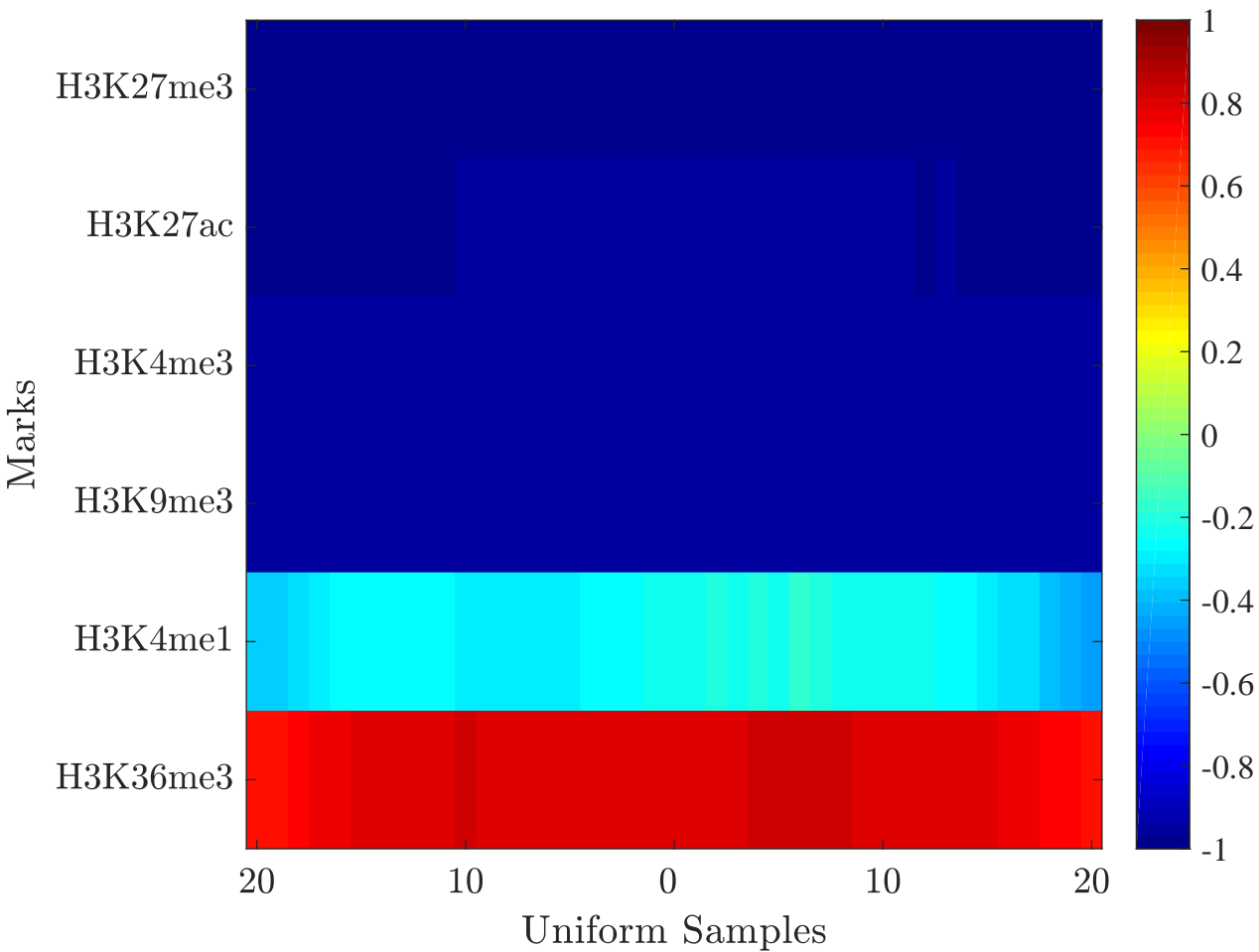

Supplement: Supplementary file 7 — HebbPlots of coding regions of active genes. This compressed file (.tar.gz) includes HebbPlots of genes active in 57 tissues/cell types. (TAR 2696 kb) [file 12859_2018_2312_MOESM7_ESM.tar › file8/E105.pdf]

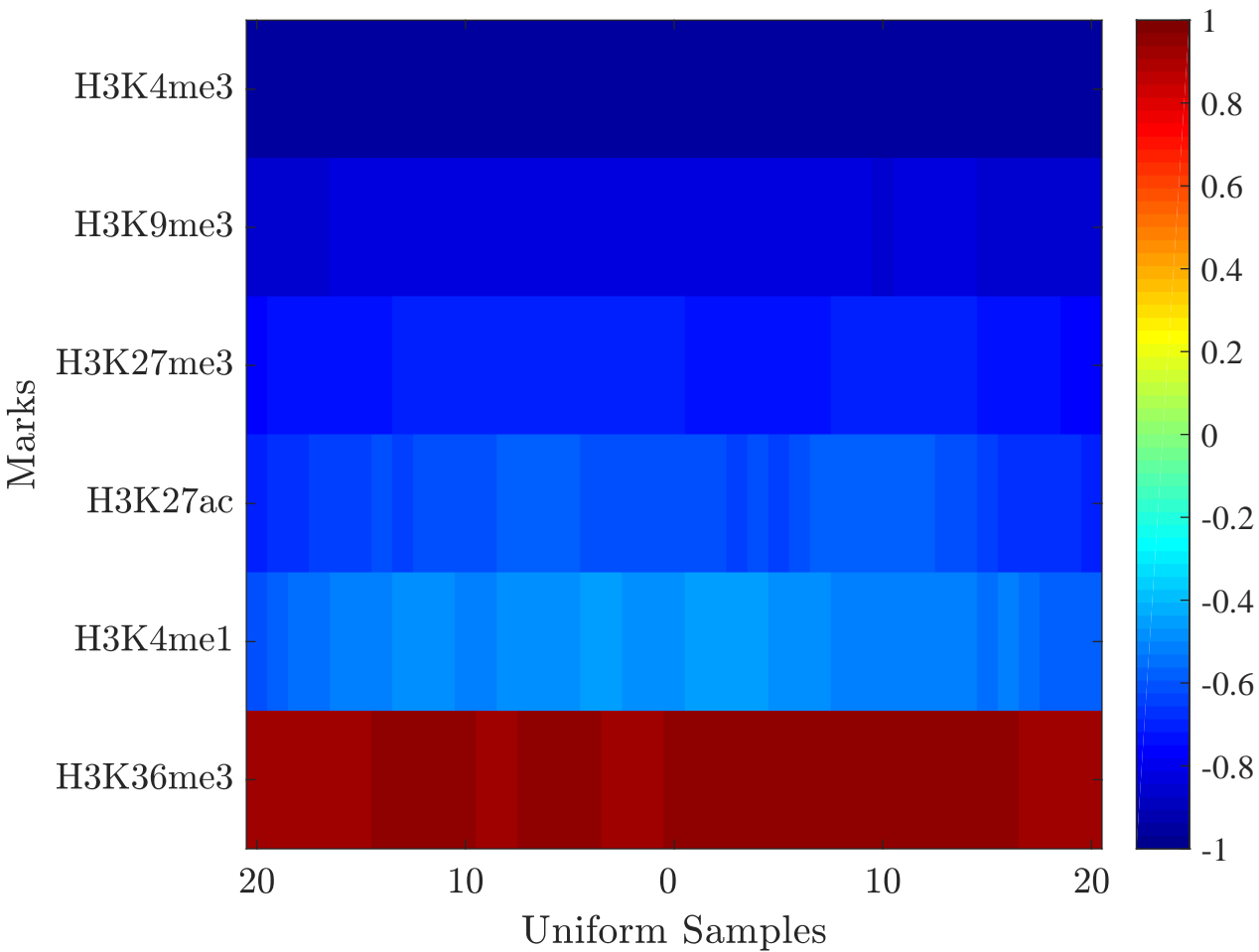

Supplement: Supplementary file 7 — HebbPlots of coding regions of active genes. This compressed file (.tar.gz) includes HebbPlots of genes active in 57 tissues/cell types. (TAR 2696 kb) [file 12859_2018_2312_MOESM7_ESM.tar › file8/E106.pdf]

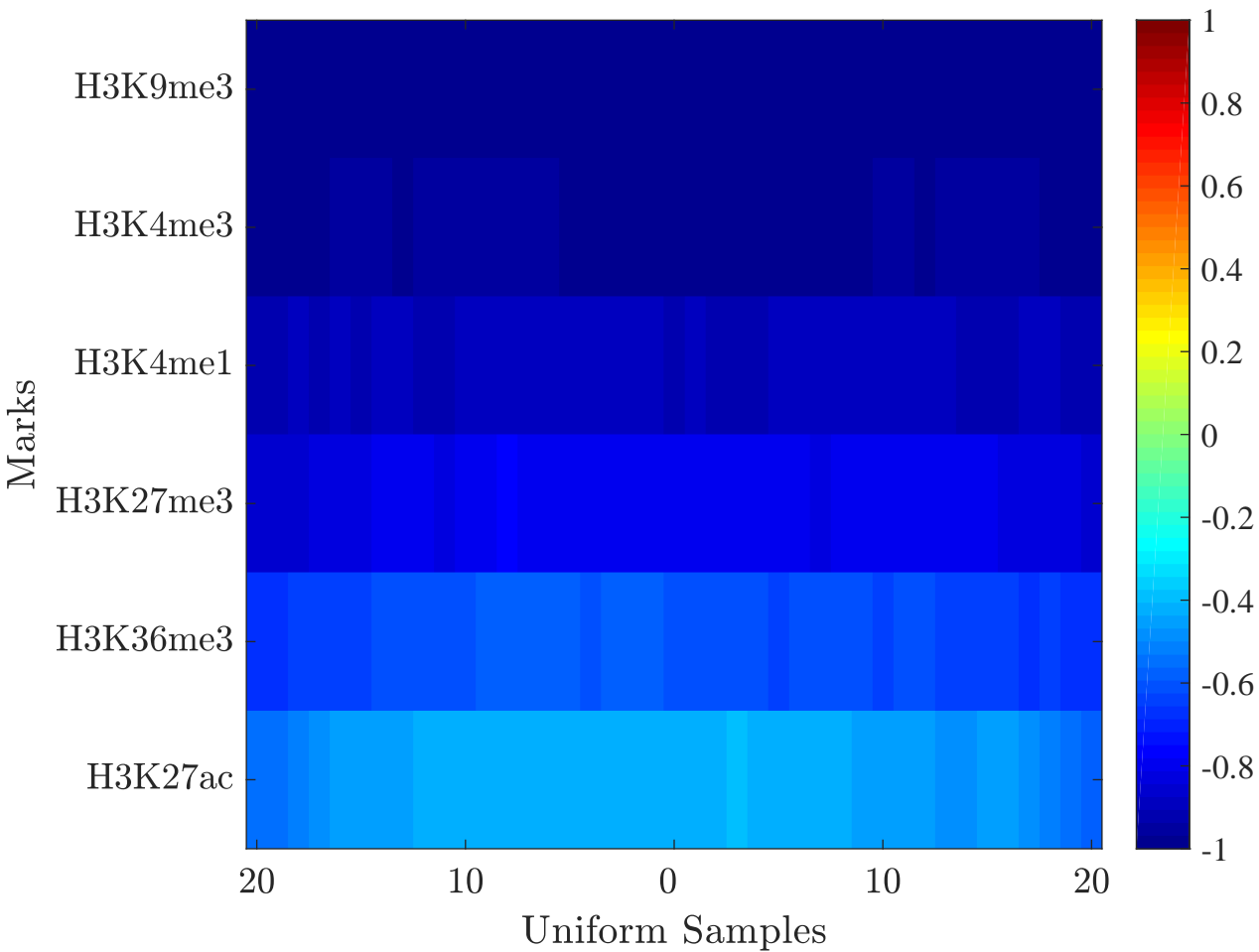

Supplement: Supplementary file 7 — HebbPlots of coding regions of active genes. This compressed file (.tar.gz) includes HebbPlots of genes active in 57 tissues/cell types. (TAR 2696 kb) [file 12859_2018_2312_MOESM7_ESM.tar › file8/E109.pdf]

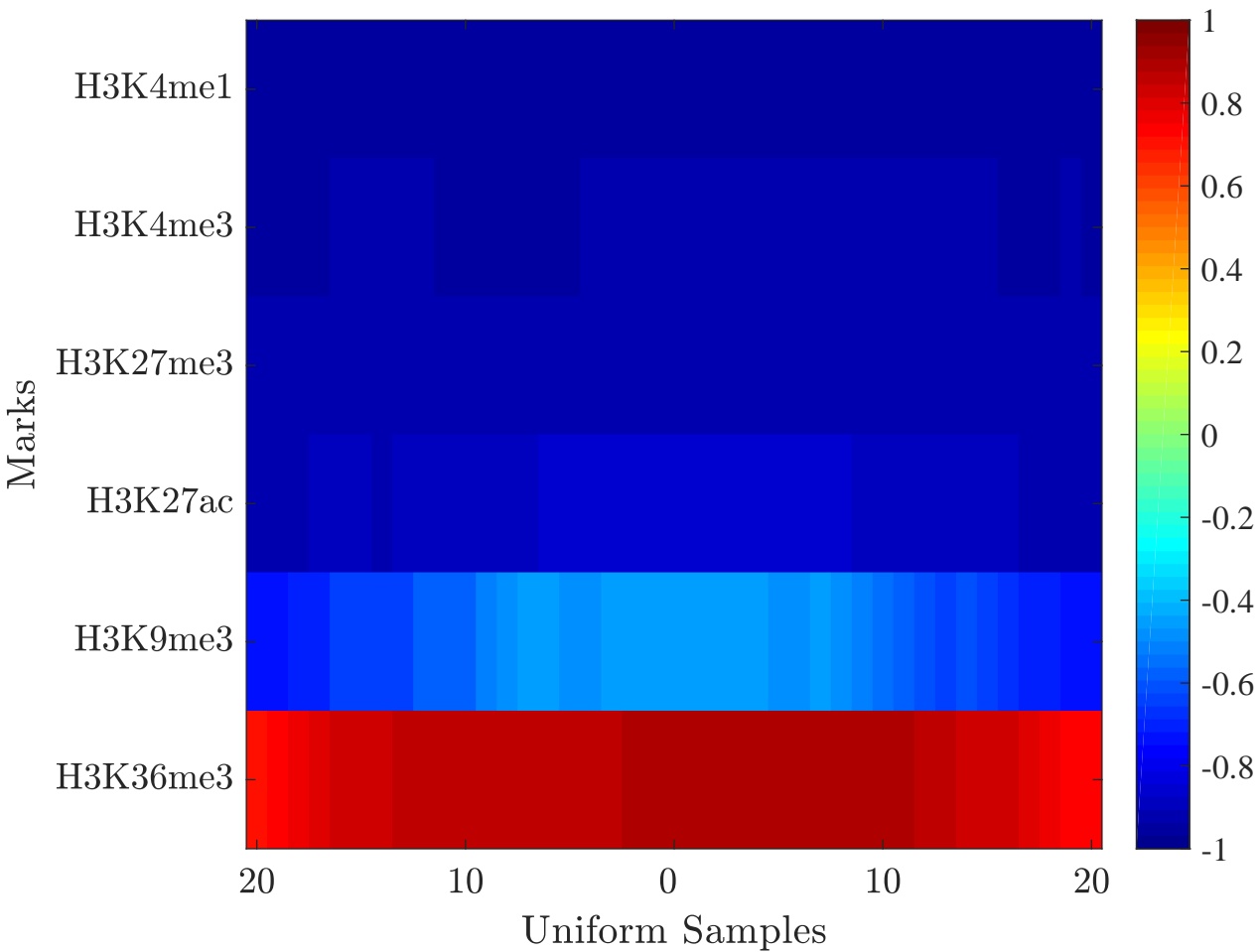

Supplement: Supplementary file 7 — HebbPlots of coding regions of active genes. This compressed file (.tar.gz) includes HebbPlots of genes active in 57 tissues/cell types. (TAR 2696 kb) [file 12859_2018_2312_MOESM7_ESM.tar › file8/E112.pdf]

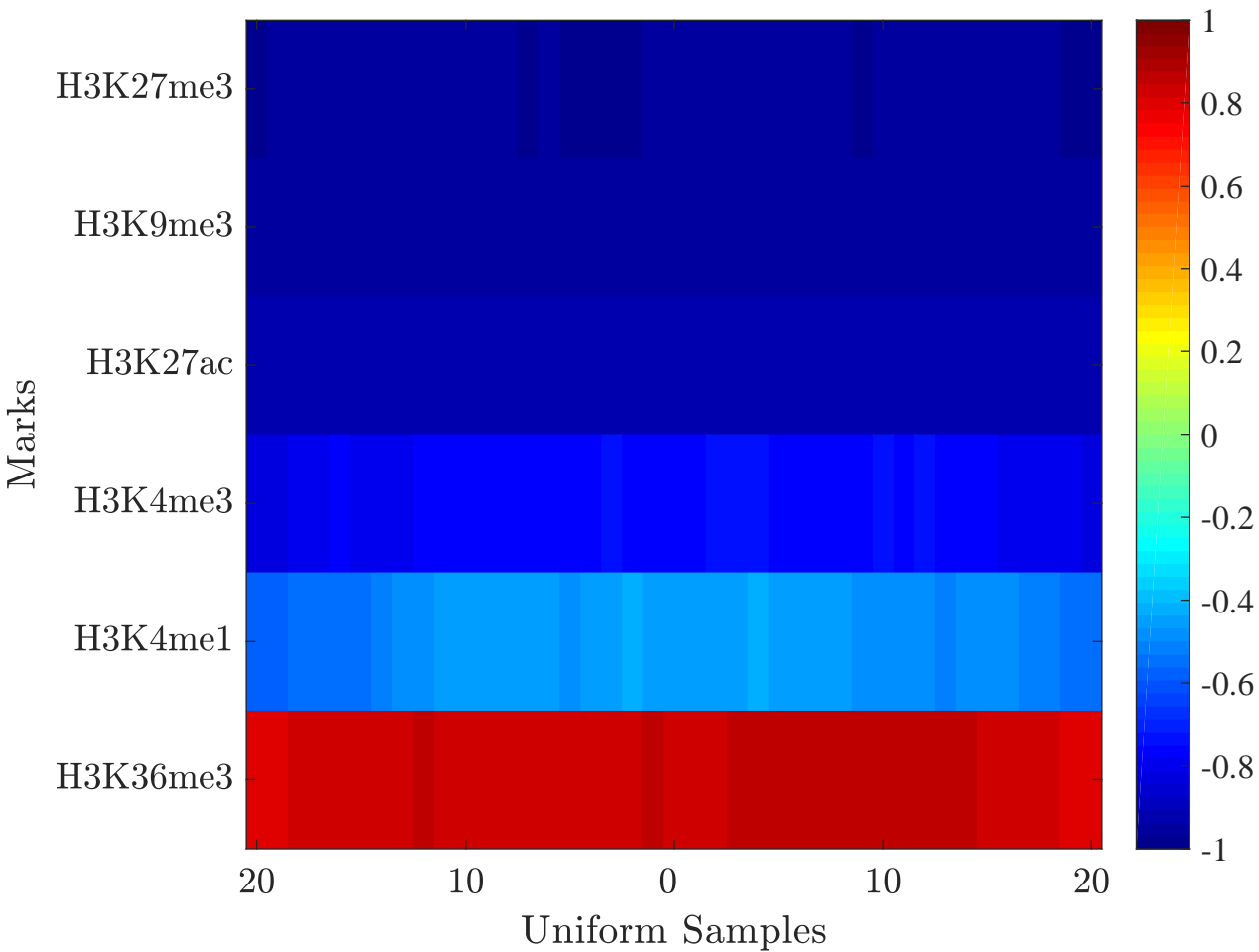

Supplement: Supplementary file 7 — HebbPlots of coding regions of active genes. This compressed file (.tar.gz) includes HebbPlots of genes active in 57 tissues/cell types. (TAR 2696 kb) [file 12859_2018_2312_MOESM7_ESM.tar › file8/E113.pdf]

Marks

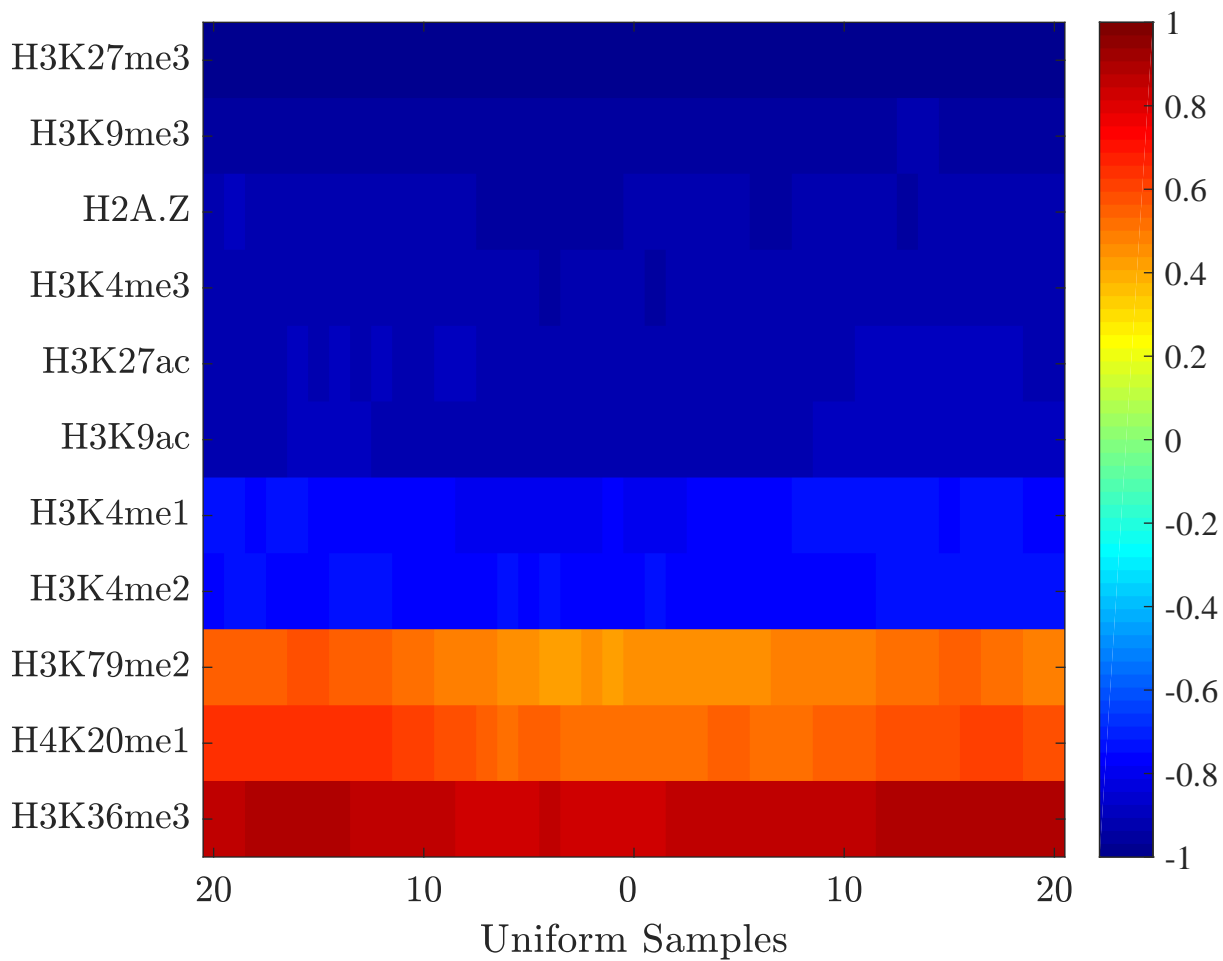

Supplement: Supplementary file 7 — HebbPlots of coding regions of active genes. This compressed file (.tar.gz) includes HebbPlots of genes active in 57 tissues/cell types. (TAR 2696 kb) [file 12859_2018_2312_MOESM7_ESM.tar › file8/E114.pdf]

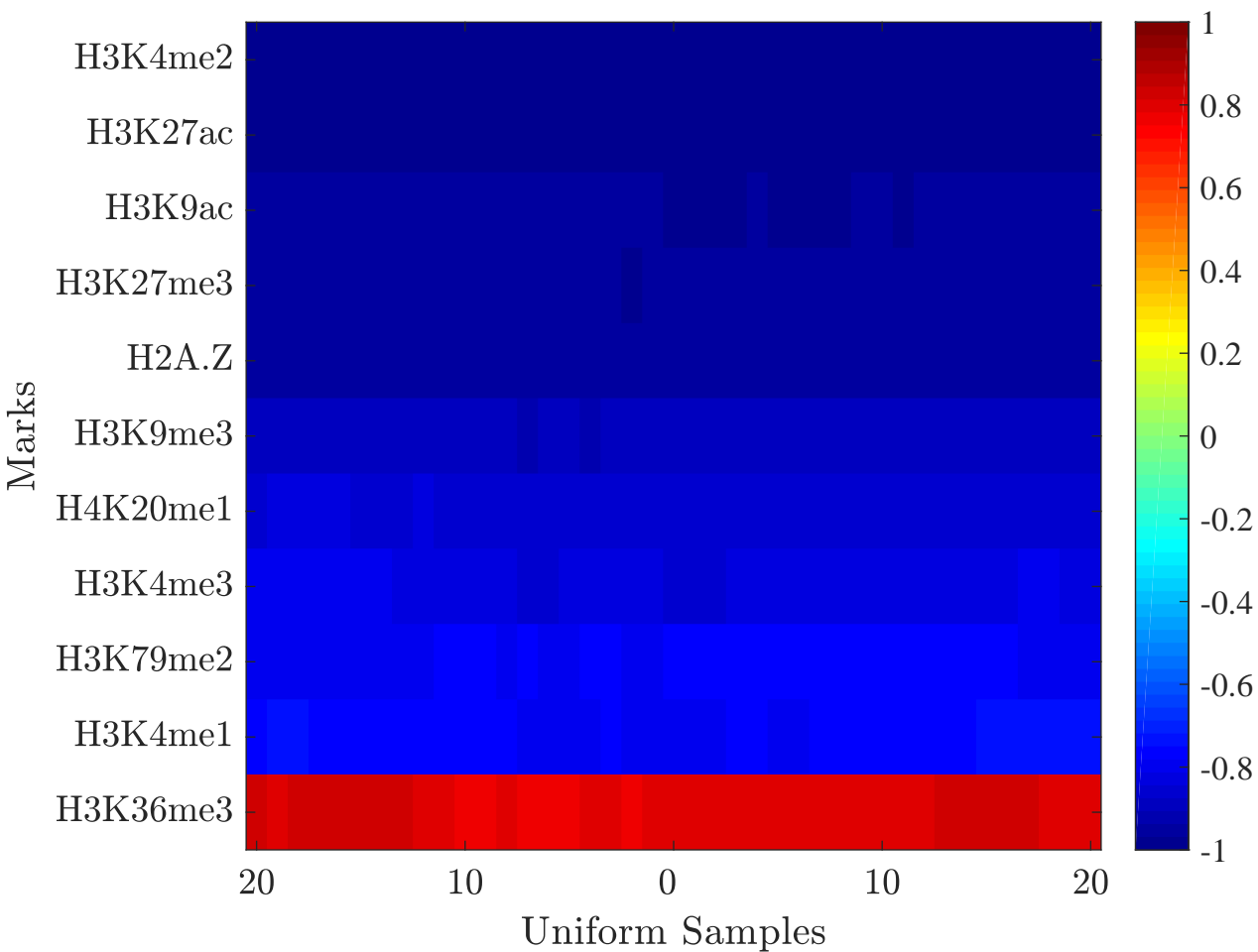

Supplement: Supplementary file 7 — HebbPlots of coding regions of active genes. This compressed file (.tar.gz) includes HebbPlots of genes active in 57 tissues/cell types. (TAR 2696 kb) [file 12859_2018_2312_MOESM7_ESM.tar › file8/E116.pdf]

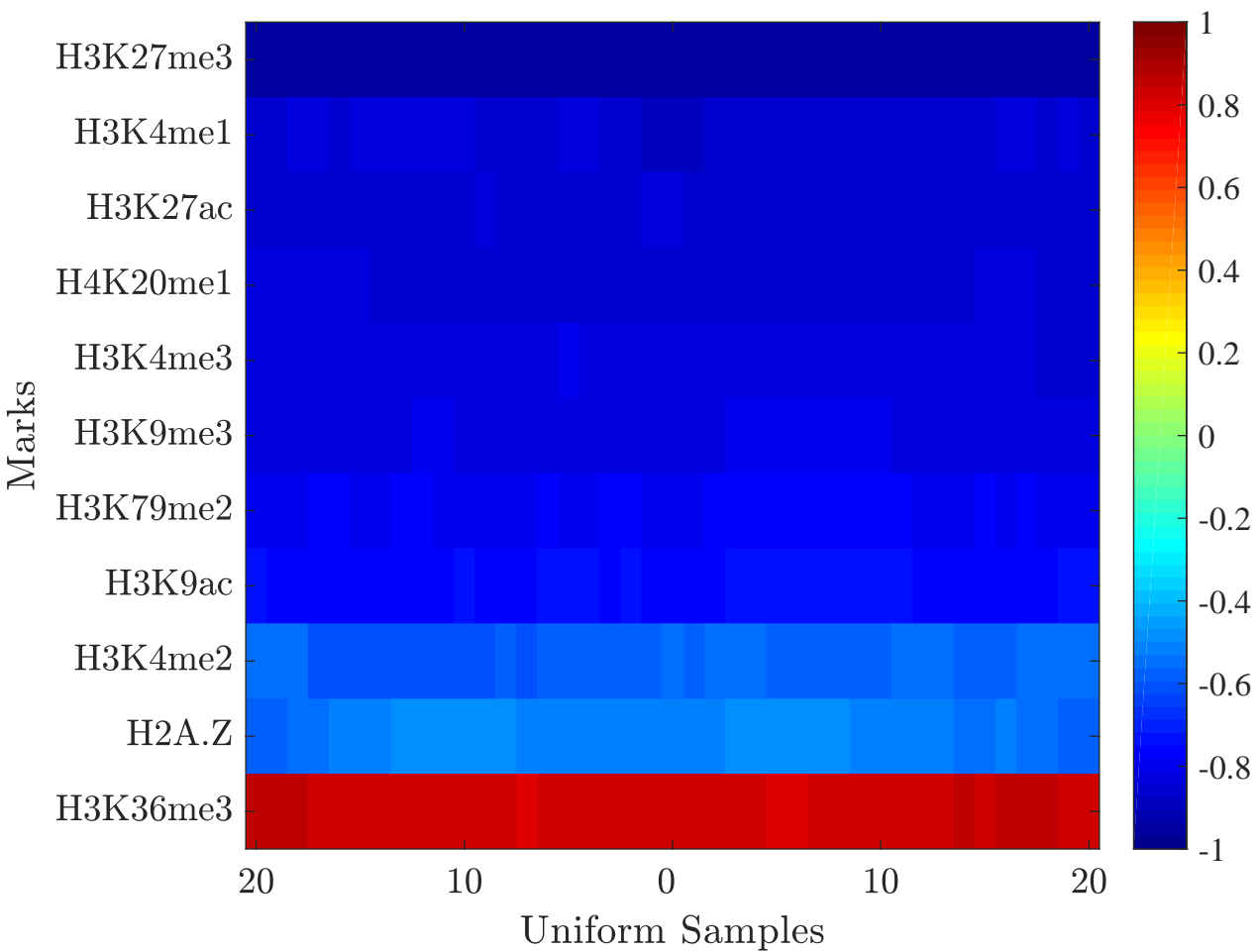

Supplement: Supplementary file 7 — HebbPlots of coding regions of active genes. This compressed file (.tar.gz) includes HebbPlots of genes active in 57 tissues/cell types. (TAR 2696 kb) [file 12859_2018_2312_MOESM7_ESM.tar › file8/E117.pdf]

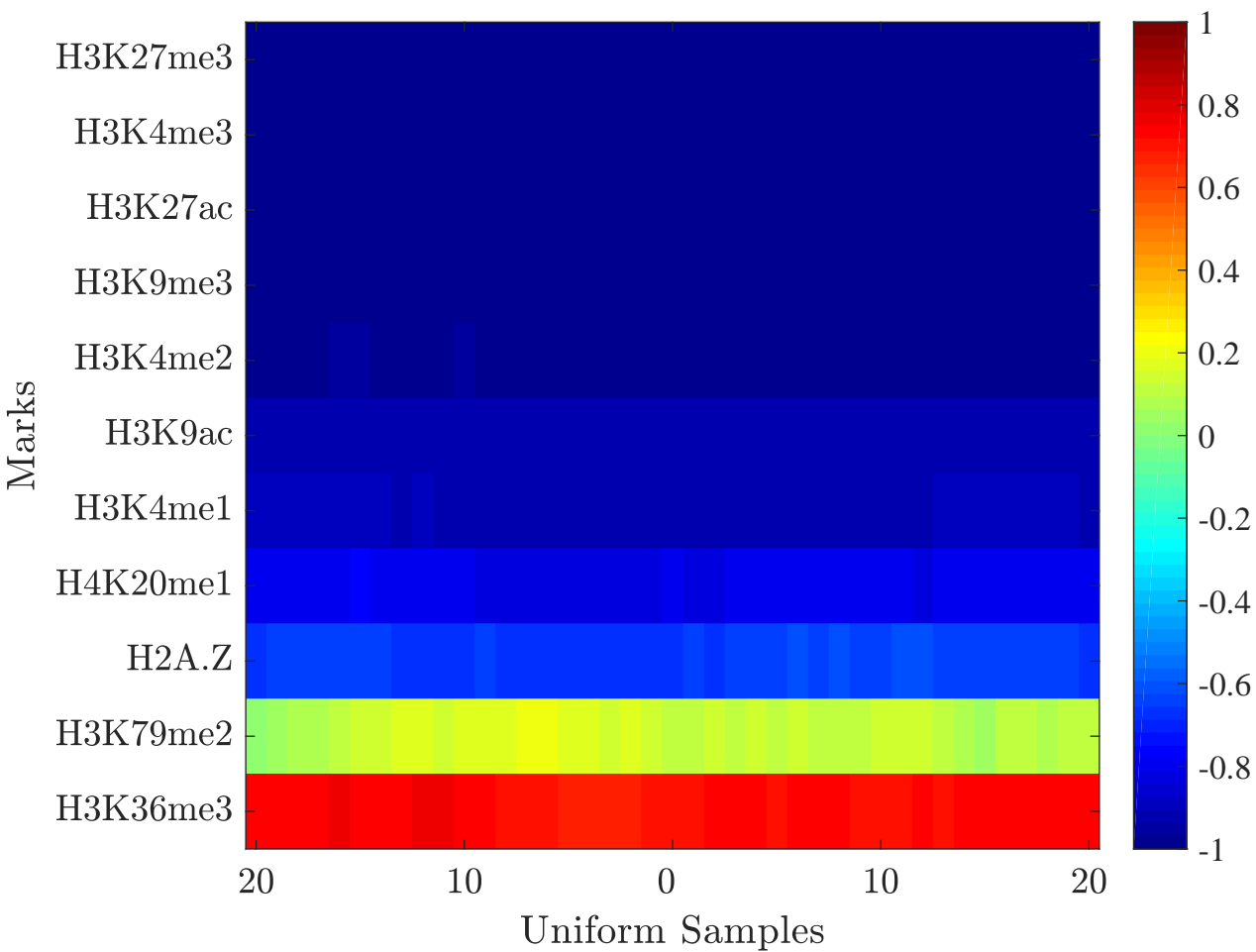

Supplement: Supplementary file 7 — HebbPlots of coding regions of active genes. This compressed file (.tar.gz) includes HebbPlots of genes active in 57 tissues/cell types. (TAR 2696 kb) [file 12859_2018_2312_MOESM7_ESM.tar › file8/E118.pdf]

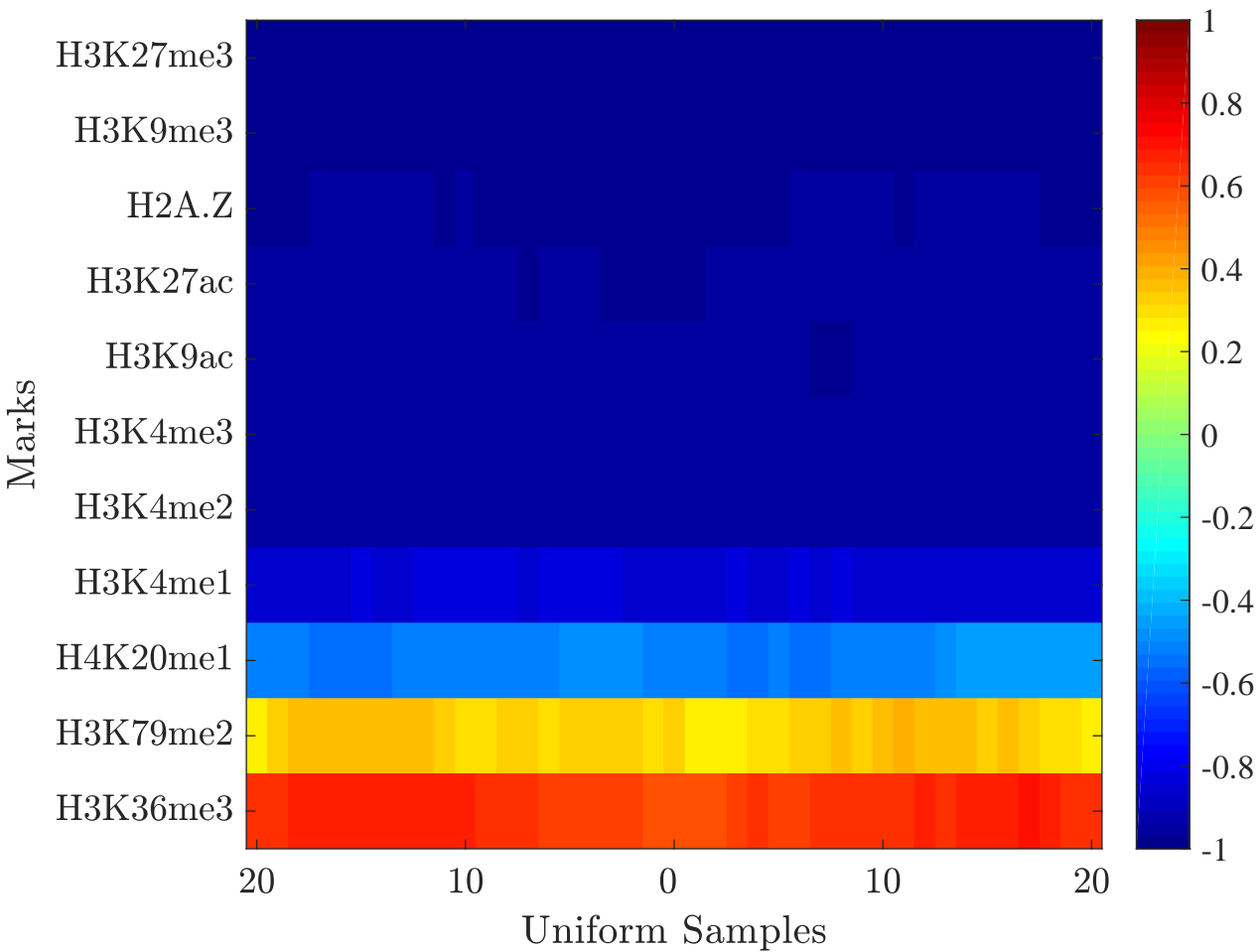

Supplement: Supplementary file 7 — HebbPlots of coding regions of active genes. This compressed file (.tar.gz) includes HebbPlots of genes active in 57 tissues/cell types. (TAR 2696 kb) [file 12859_2018_2312_MOESM7_ESM.tar › file8/E119.pdf]

Marks

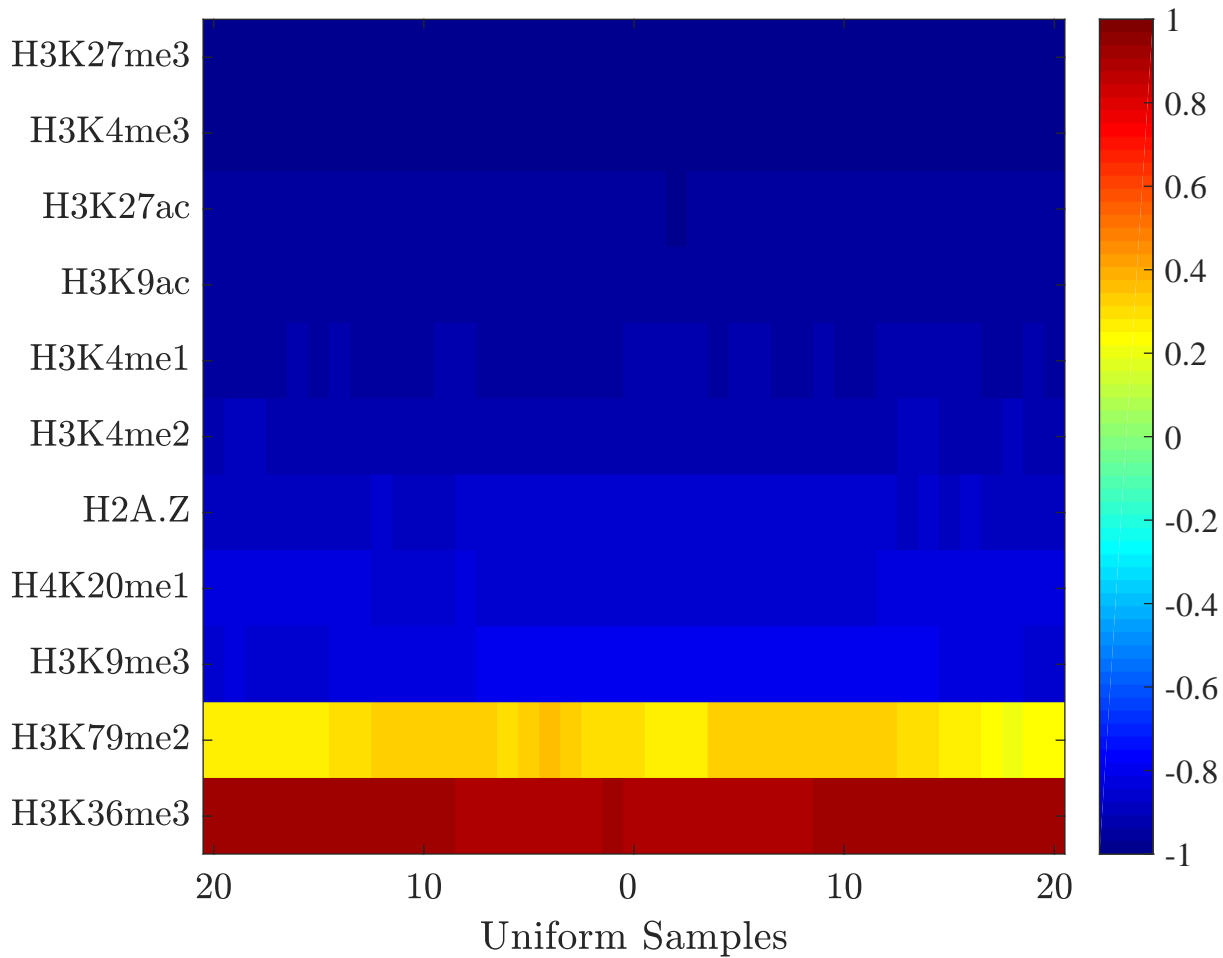

Supplement: Supplementary file 7 — HebbPlots of coding regions of active genes. This compressed file (.tar.gz) includes HebbPlots of genes active in 57 tissues/cell types. (TAR 2696 kb) [file 12859_2018_2312_MOESM7_ESM.tar › file8/E120.pdf]

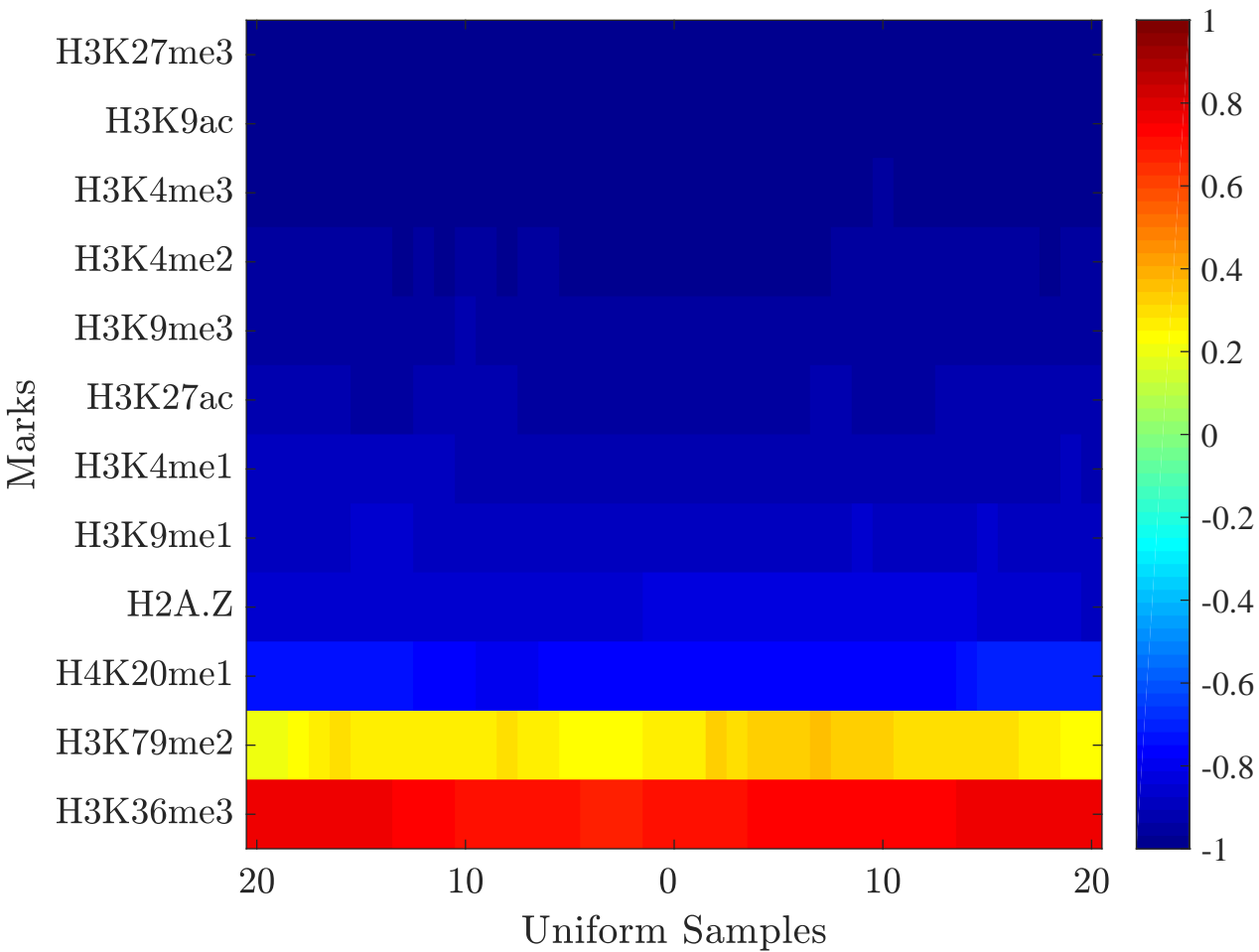

Supplement: Supplementary file 7 — HebbPlots of coding regions of active genes. This compressed file (.tar.gz) includes HebbPlots of genes active in 57 tissues/cell types. (TAR 2696 kb) [file 12859_2018_2312_MOESM7_ESM.tar › file8/E122.pdf]

Marks

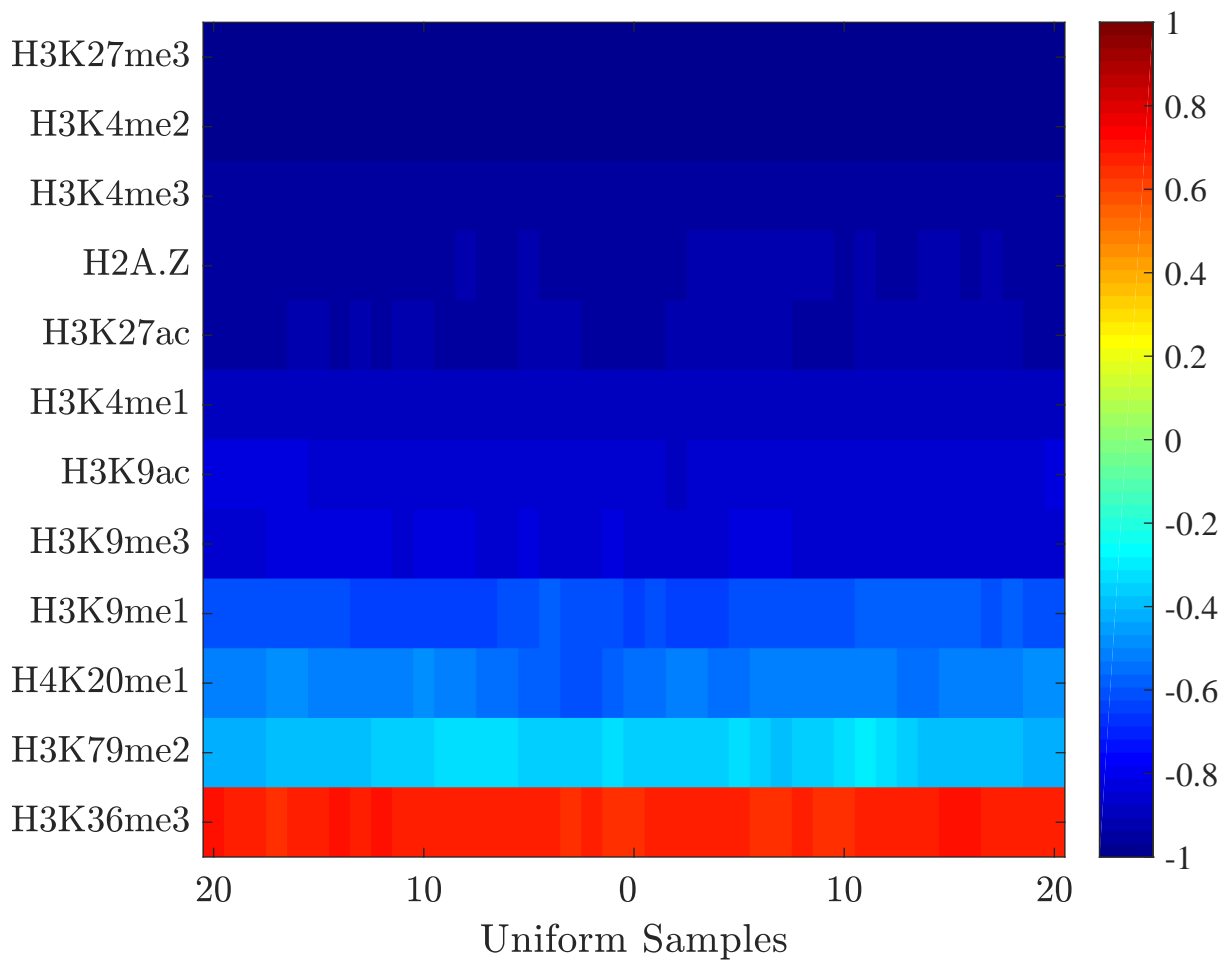

Supplement: Supplementary file 7 — HebbPlots of coding regions of active genes. This compressed file (.tar.gz) includes HebbPlots of genes active in 57 tissues/cell types. (TAR 2696 kb) [file 12859_2018_2312_MOESM7_ESM.tar › file8/E123.pdf]

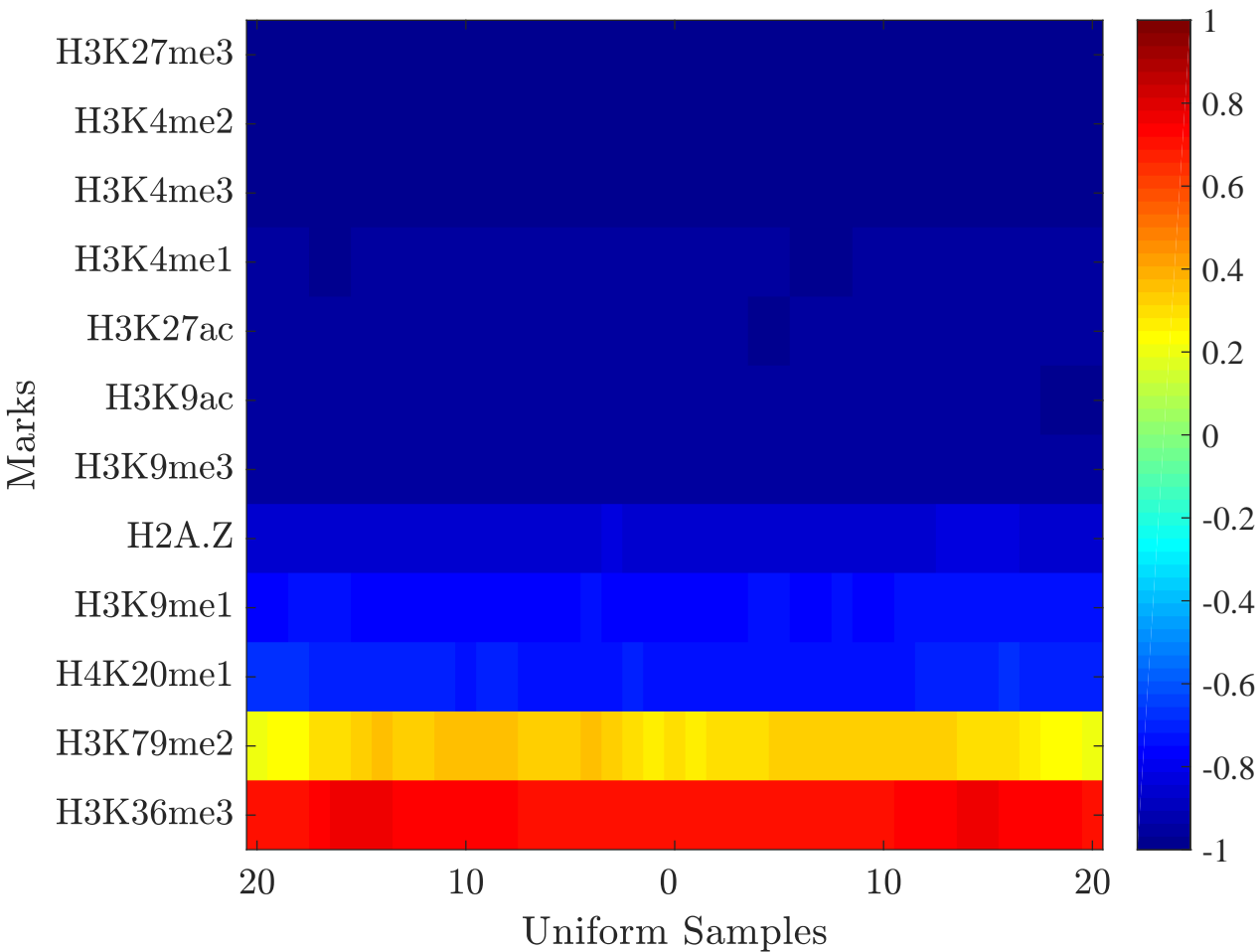

Supplement: Supplementary file 7 — HebbPlots of coding regions of active genes. This compressed file (.tar.gz) includes HebbPlots of genes active in 57 tissues/cell types. (TAR 2696 kb) [file 12859_2018_2312_MOESM7_ESM.tar › file8/E127.pdf]

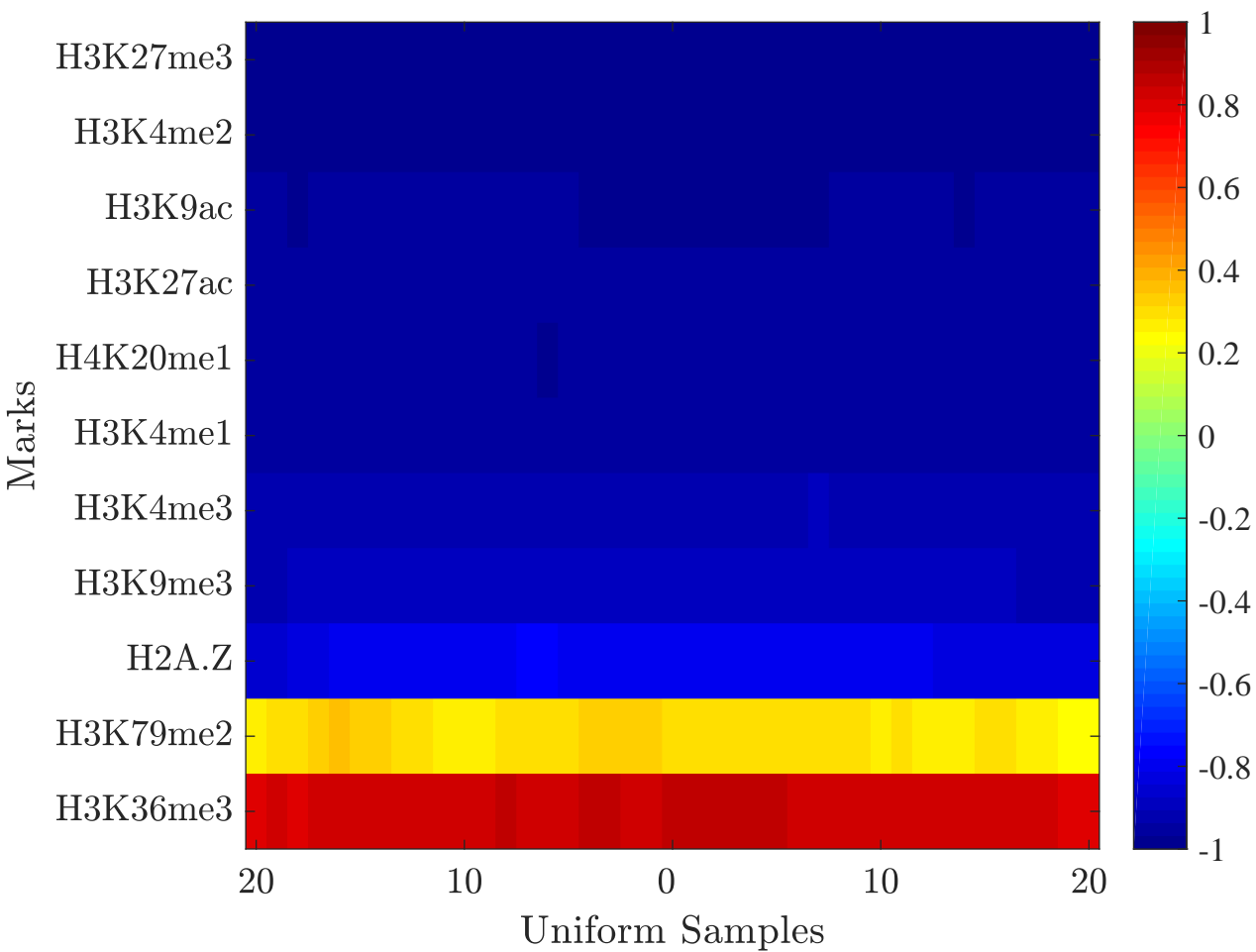

Supplement: Supplementary file 7 — HebbPlots of coding regions of active genes. This compressed file (.tar.gz) includes HebbPlots of genes active in 57 tissues/cell types. (TAR 2696 kb) [file 12859_2018_2312_MOESM7_ESM.tar › file8/E128.pdf]

Marks

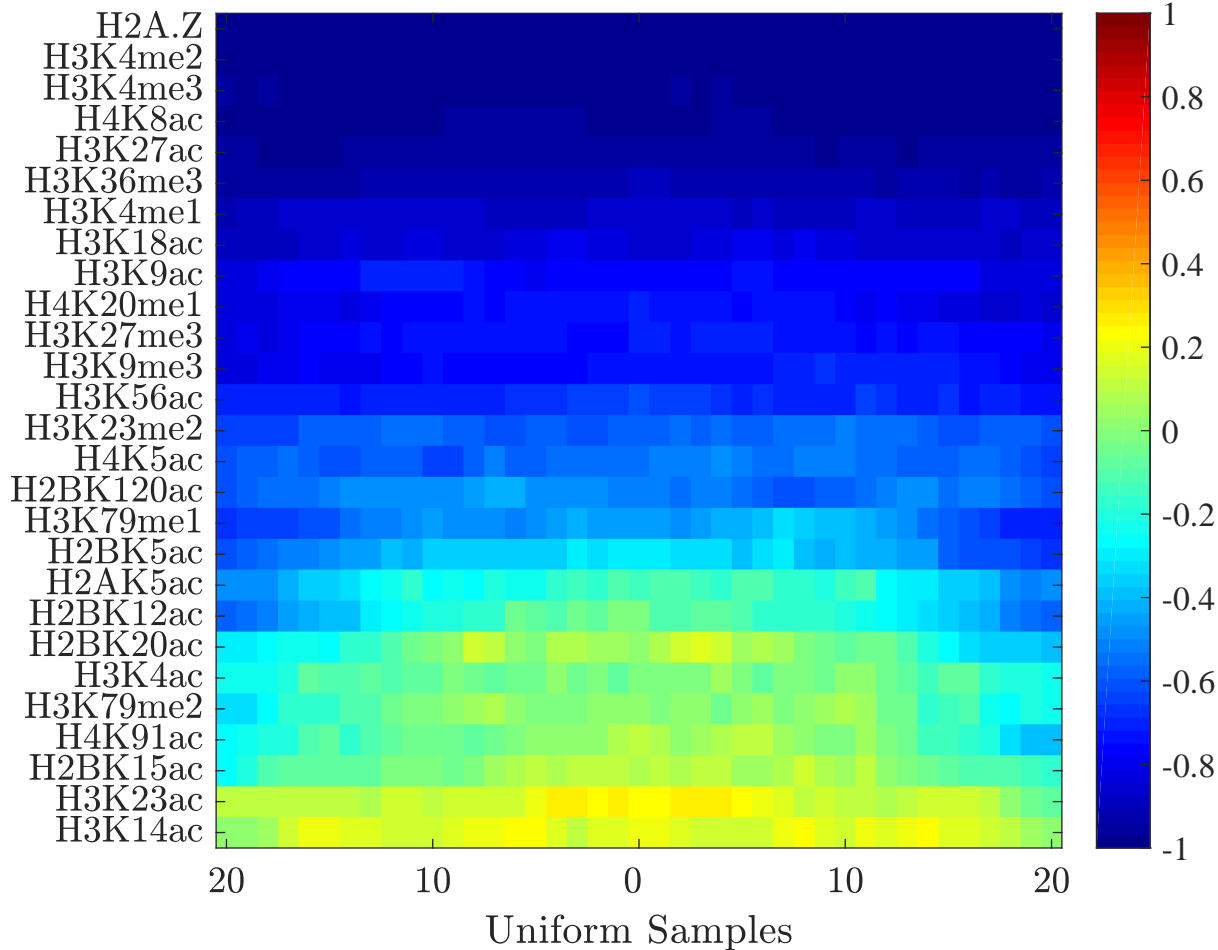

Supplement: Supplementary file 8 — HebbPlots of coding regions of inactive genes. This compressed file (.tar.gz) includes HebbPlots of genes inactive in 57 tissues/cell types. (TAR 2715 kb) [file 12859_2018_2312_MOESM8_ESM.tar › file9/E003.pdf]

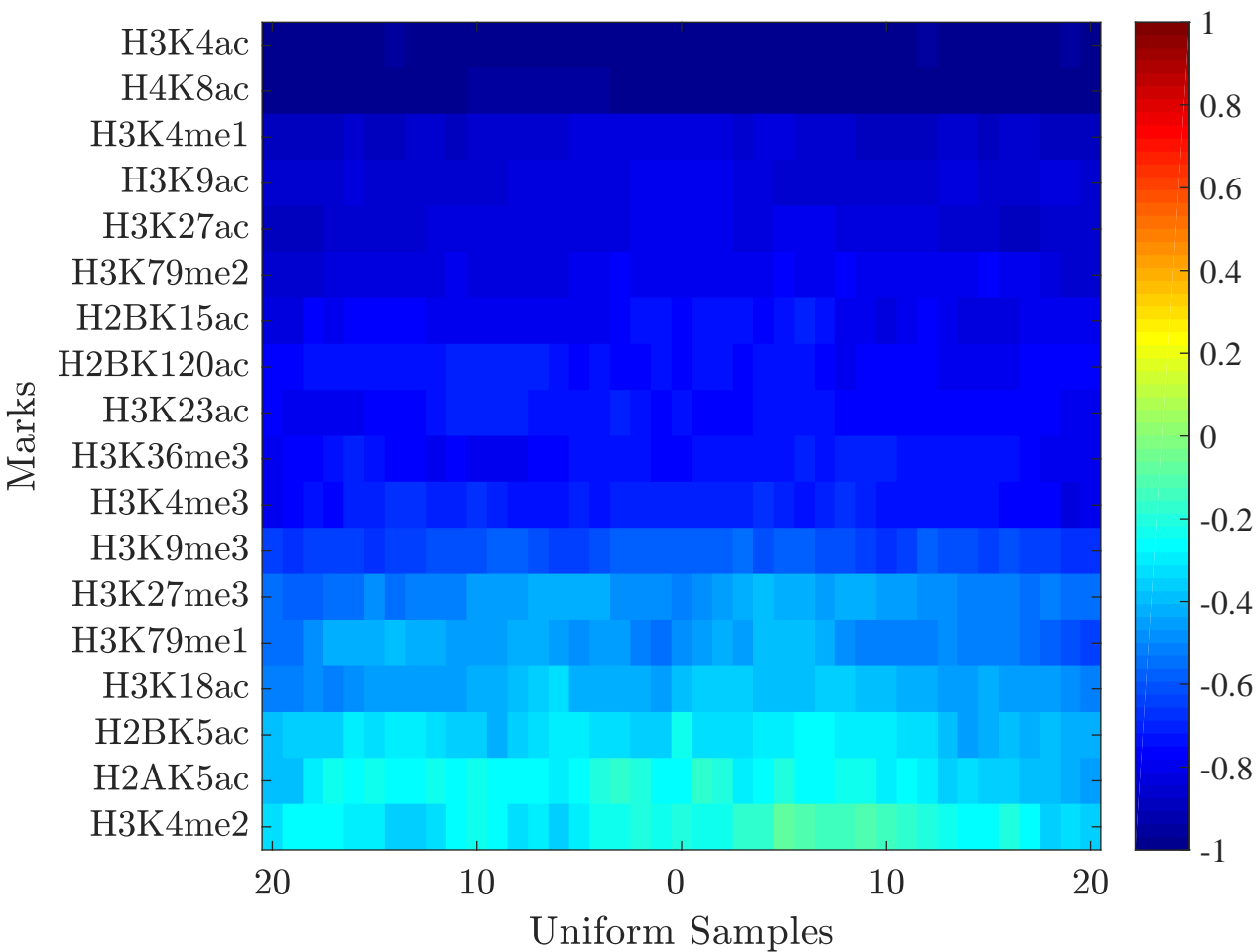

Supplement: Supplementary file 8 — HebbPlots of coding regions of inactive genes. This compressed file (.tar.gz) includes HebbPlots of genes inactive in 57 tissues/cell types. (TAR 2715 kb) [file 12859_2018_2312_MOESM8_ESM.tar › file9/E004.pdf]

Marks

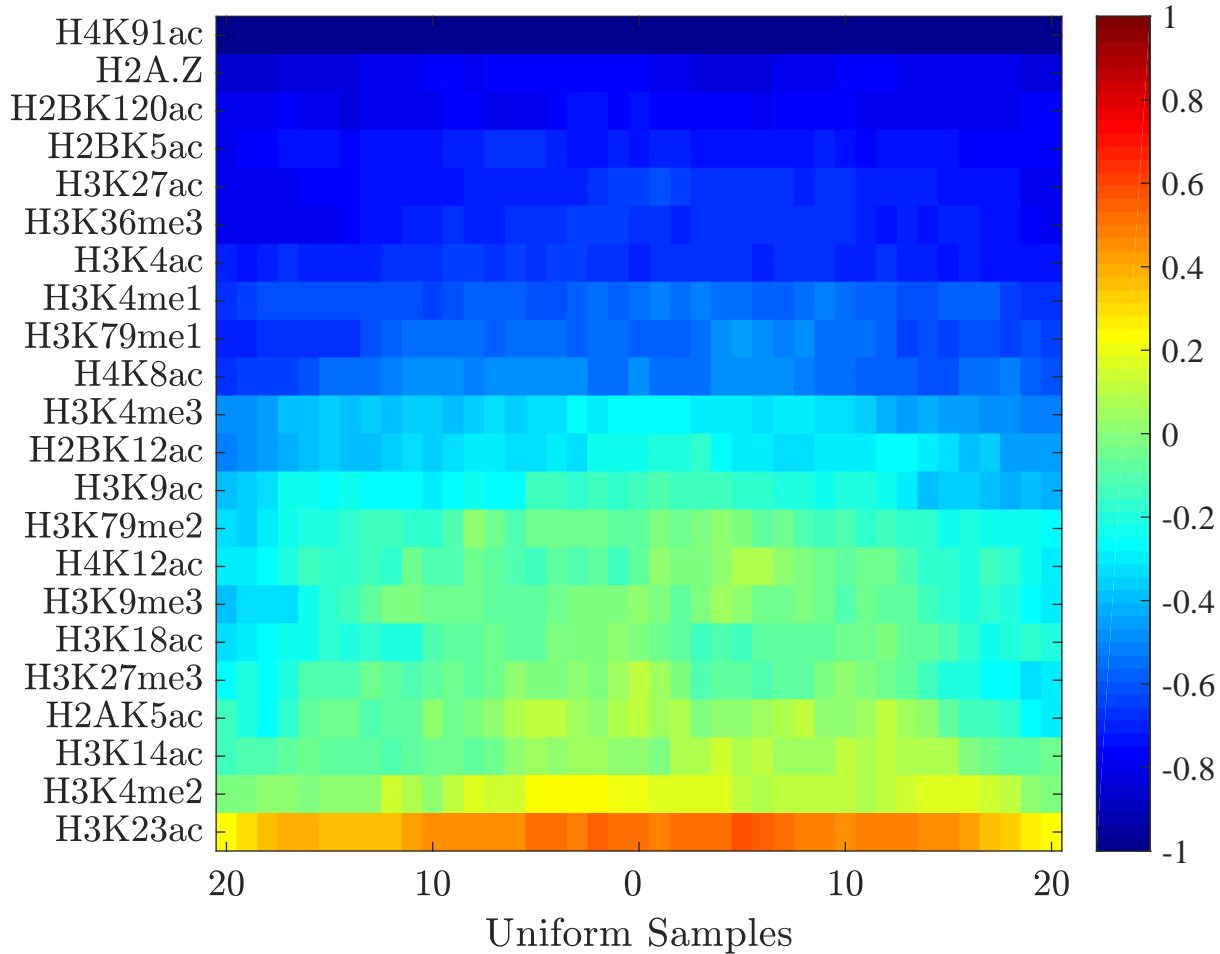

Supplement: Supplementary file 8 — HebbPlots of coding regions of inactive genes. This compressed file (.tar.gz) includes HebbPlots of genes inactive in 57 tissues/cell types. (TAR 2715 kb) [file 12859_2018_2312_MOESM8_ESM.tar › file9/E005.pdf]

Marks

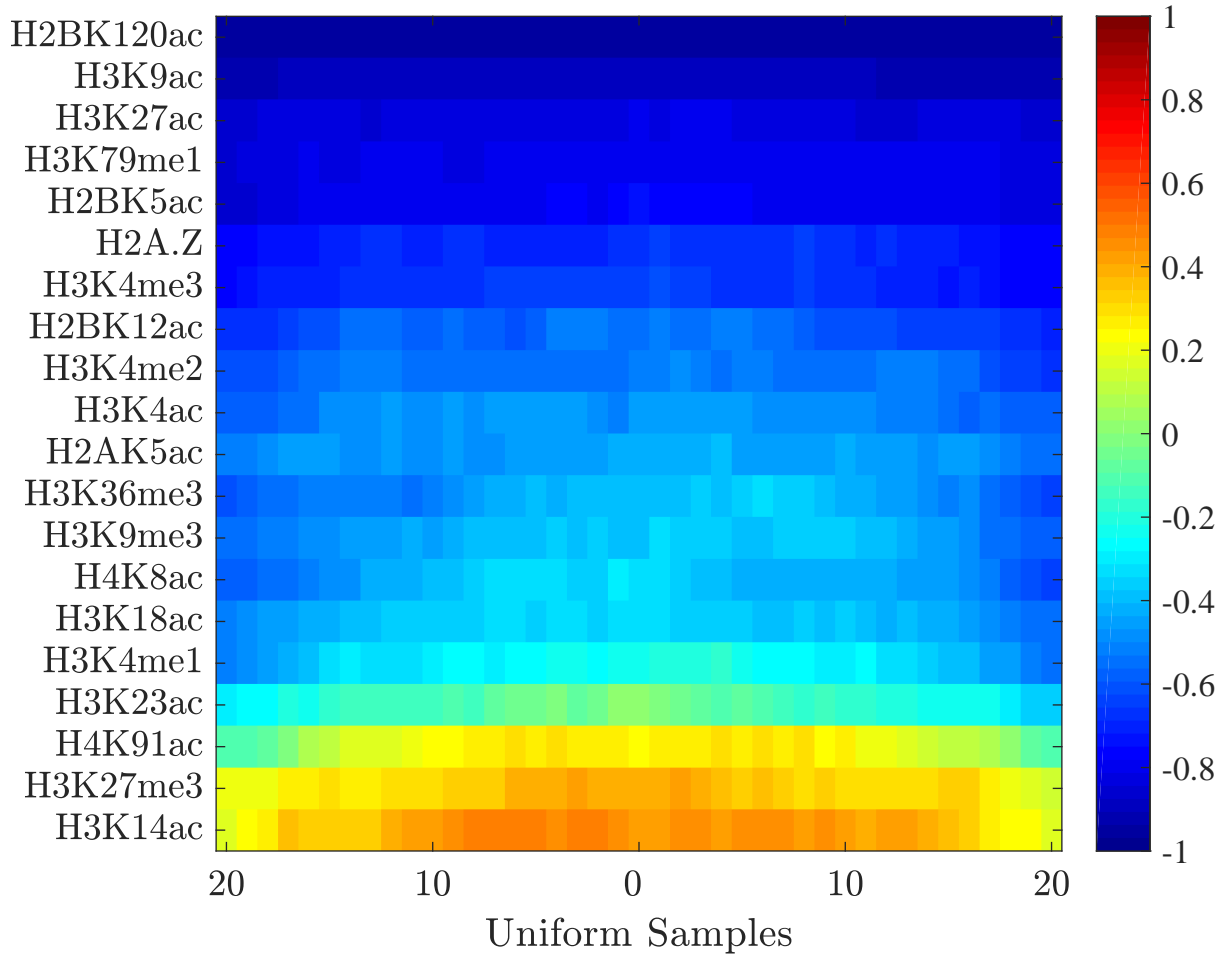

Supplement: Supplementary file 8 — HebbPlots of coding regions of inactive genes. This compressed file (.tar.gz) includes HebbPlots of genes inactive in 57 tissues/cell types. (TAR 2715 kb) [file 12859_2018_2312_MOESM8_ESM.tar › file9/E006.pdf]

Marks

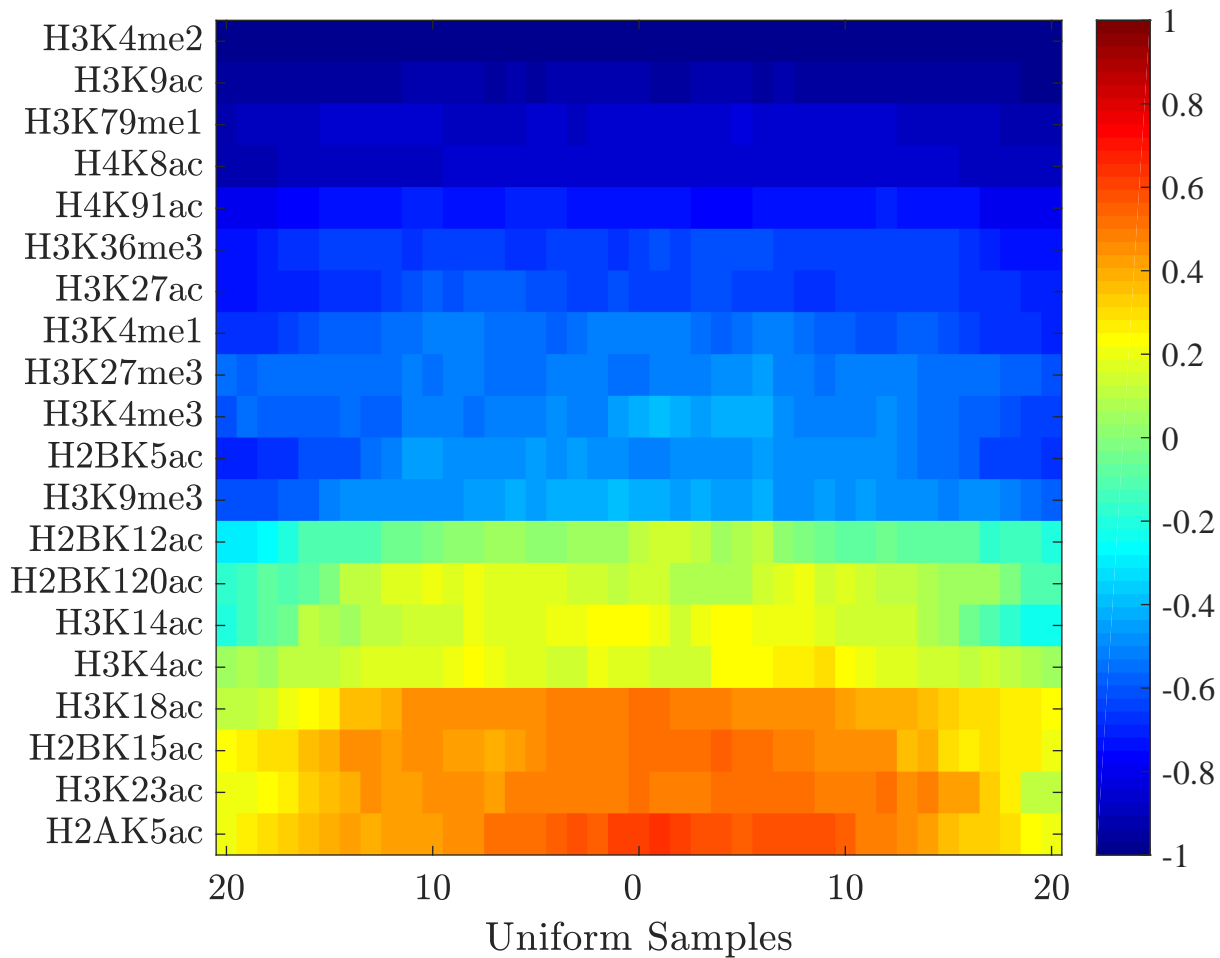

Supplement: Supplementary file 8 — HebbPlots of coding regions of inactive genes. This compressed file (.tar.gz) includes HebbPlots of genes inactive in 57 tissues/cell types. (TAR 2715 kb) [file 12859_2018_2312_MOESM8_ESM.tar › file9/E007.pdf]

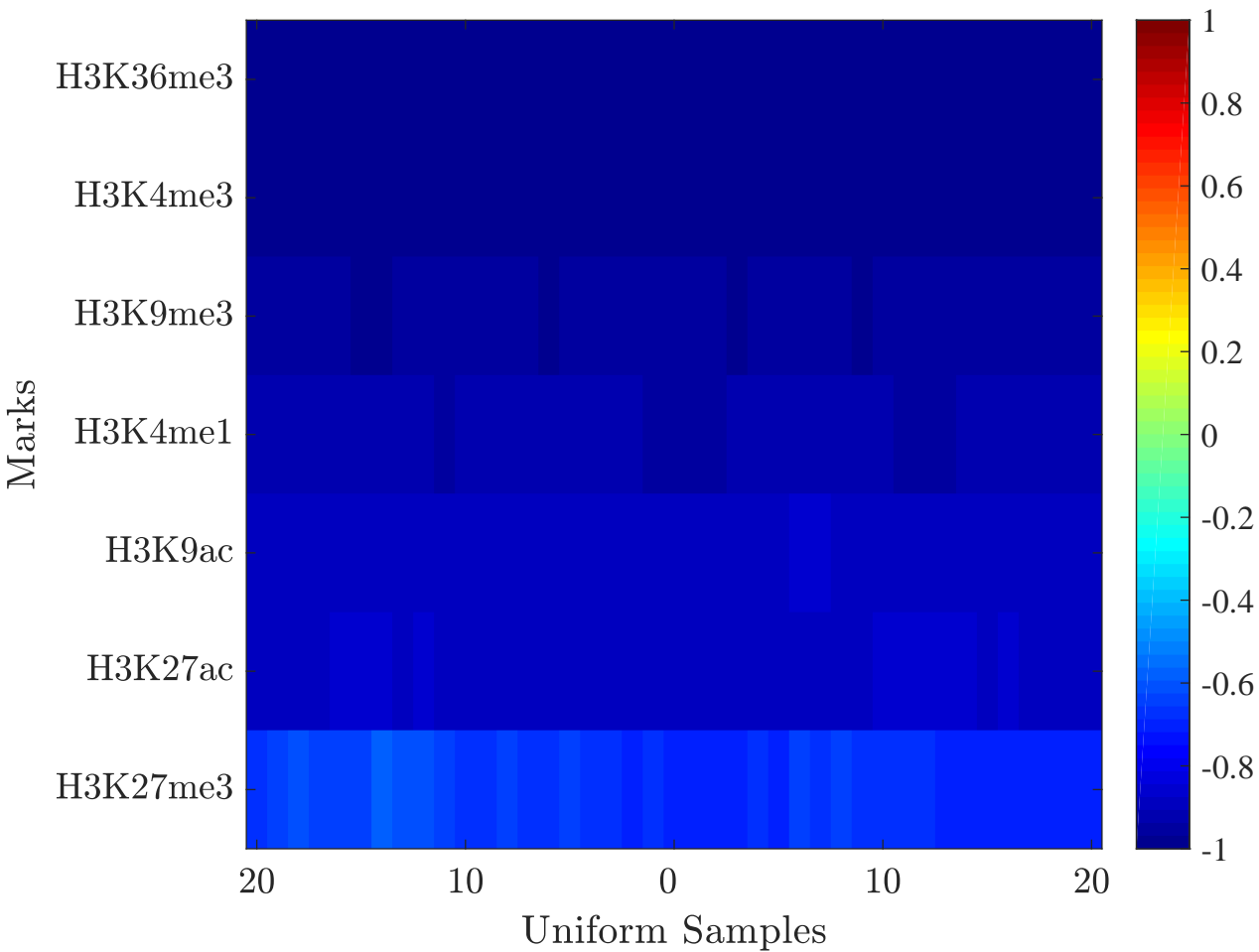

Supplement: Supplementary file 8 — HebbPlots of coding regions of inactive genes. This compressed file (.tar.gz) includes HebbPlots of genes inactive in 57 tissues/cell types. (TAR 2715 kb) [file 12859_2018_2312_MOESM8_ESM.tar › file9/E011.pdf]

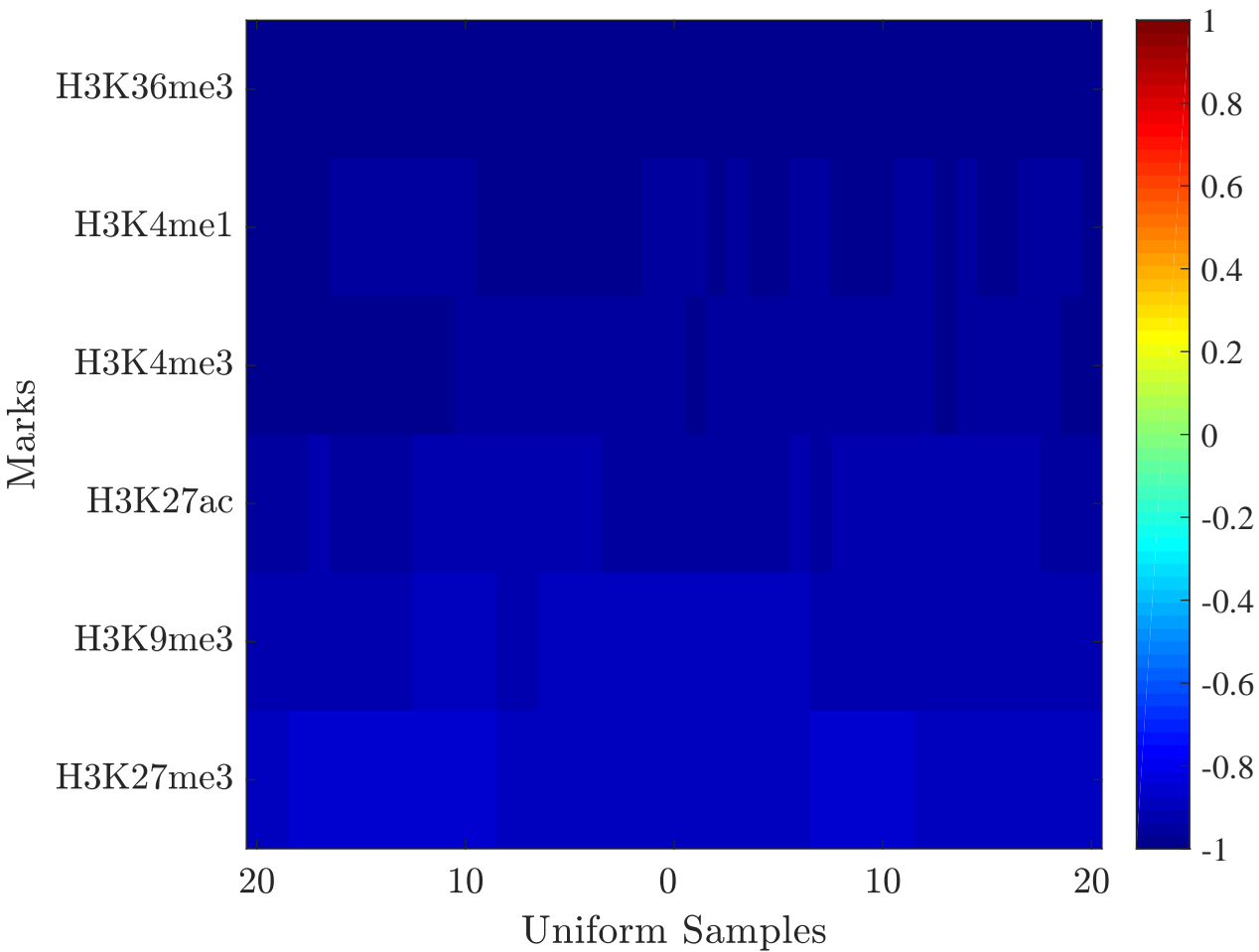

Supplement: Supplementary file 8 — HebbPlots of coding regions of inactive genes. This compressed file (.tar.gz) includes HebbPlots of genes inactive in 57 tissues/cell types. (TAR 2715 kb) [file 12859_2018_2312_MOESM8_ESM.tar › file9/E012.pdf]
