# Supplementary figures and images for: HebbPlot: an intelligent tool for learning and visualizing chromatin mark signatures (part 4 of 4)
Source: BMC Bioinformatics. 2018 Sep 3;19:310. doi: 10.1186/s12859-018-2312-1 (PMC6122555; doi:10.1186/s12859-018-2312-1)

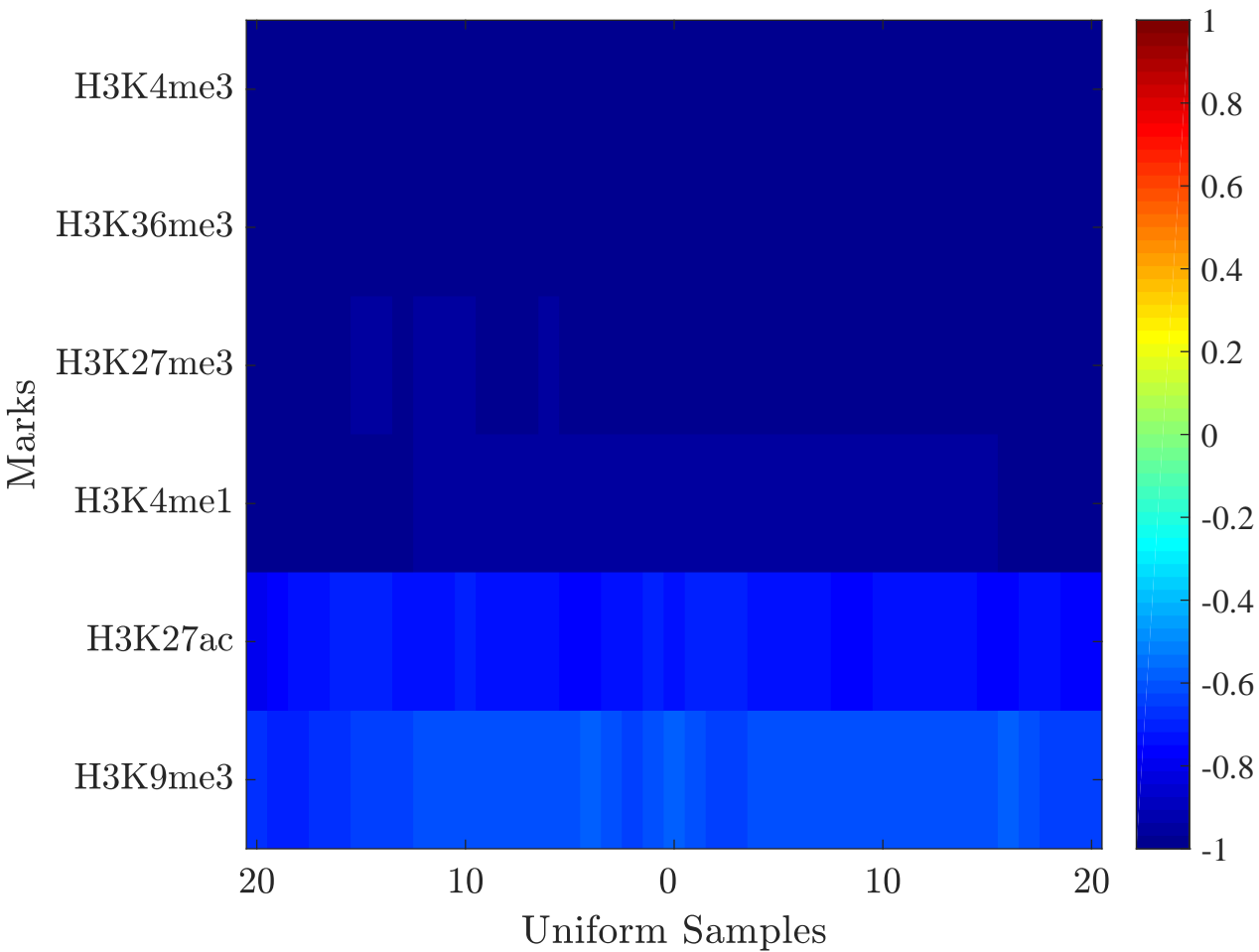

Supplement: Supplementary file 8 — HebbPlots of coding regions of inactive genes. This compressed file (.tar.gz) includes HebbPlots of genes inactive in 57 tissues/cell types. (TAR 2715 kb) [file 12859_2018_2312_MOESM8_ESM.tar › file9/E013.pdf]

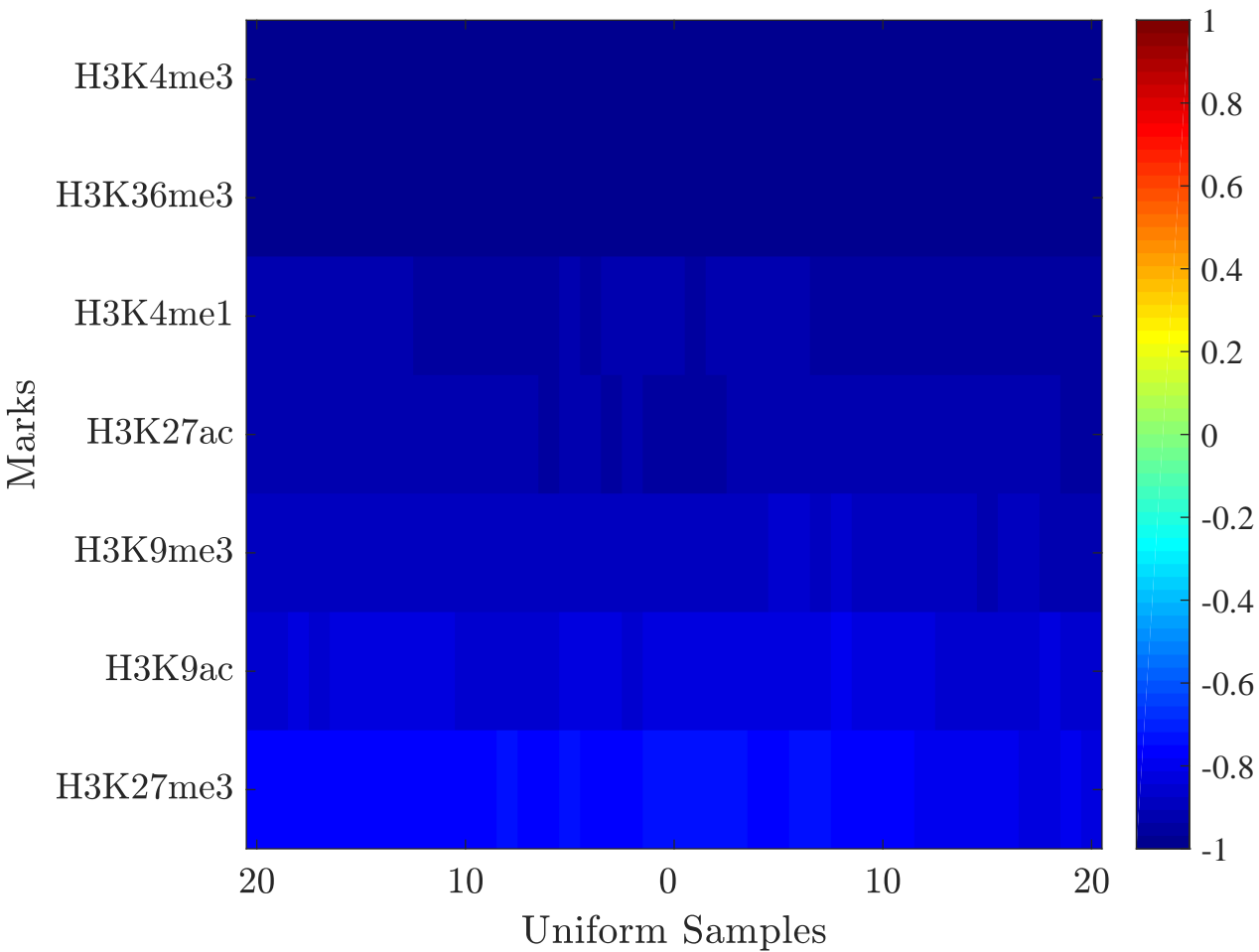

Supplement: Supplementary file 8 — HebbPlots of coding regions of inactive genes. This compressed file (.tar.gz) includes HebbPlots of genes inactive in 57 tissues/cell types. (TAR 2715 kb) [file 12859_2018_2312_MOESM8_ESM.tar › file9/E016.pdf]

Marks

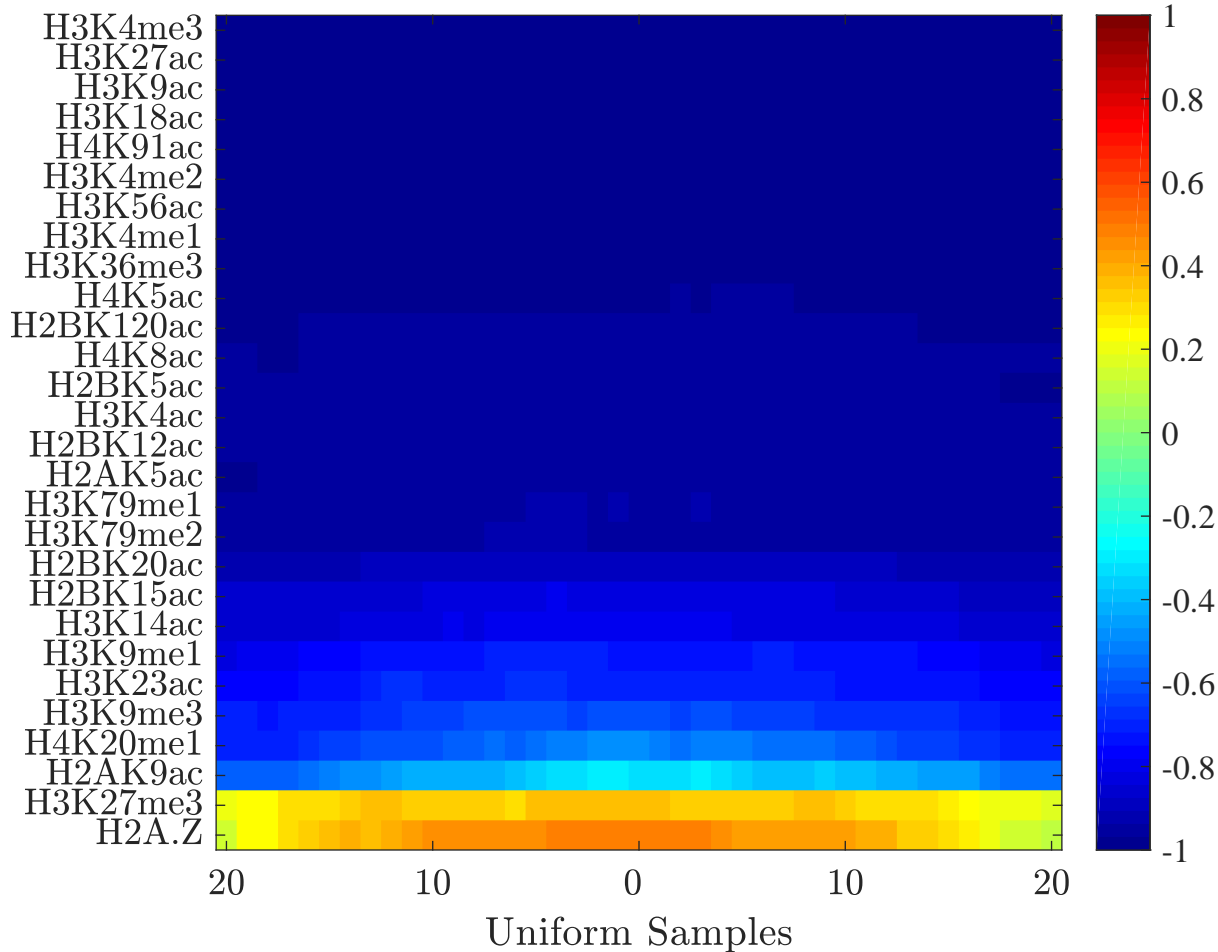

Supplement: Supplementary file 8 — HebbPlots of coding regions of inactive genes. This compressed file (.tar.gz) includes HebbPlots of genes inactive in 57 tissues/cell types. (TAR 2715 kb) [file 12859_2018_2312_MOESM8_ESM.tar › file9/E017.pdf]

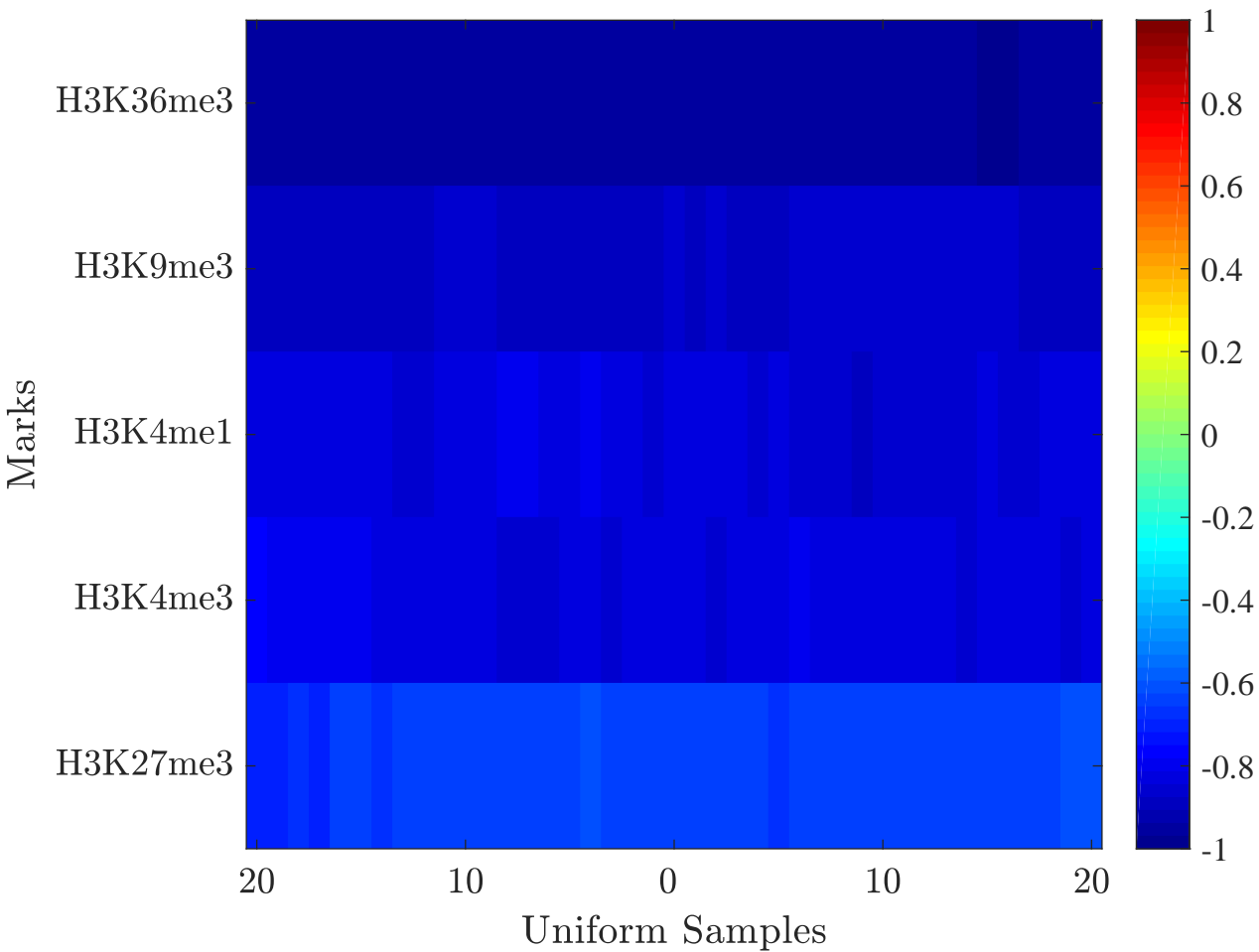

Supplement: Supplementary file 8 — HebbPlots of coding regions of inactive genes. This compressed file (.tar.gz) includes HebbPlots of genes inactive in 57 tissues/cell types. (TAR 2715 kb) [file 12859_2018_2312_MOESM8_ESM.tar › file9/E024.pdf]

Marks

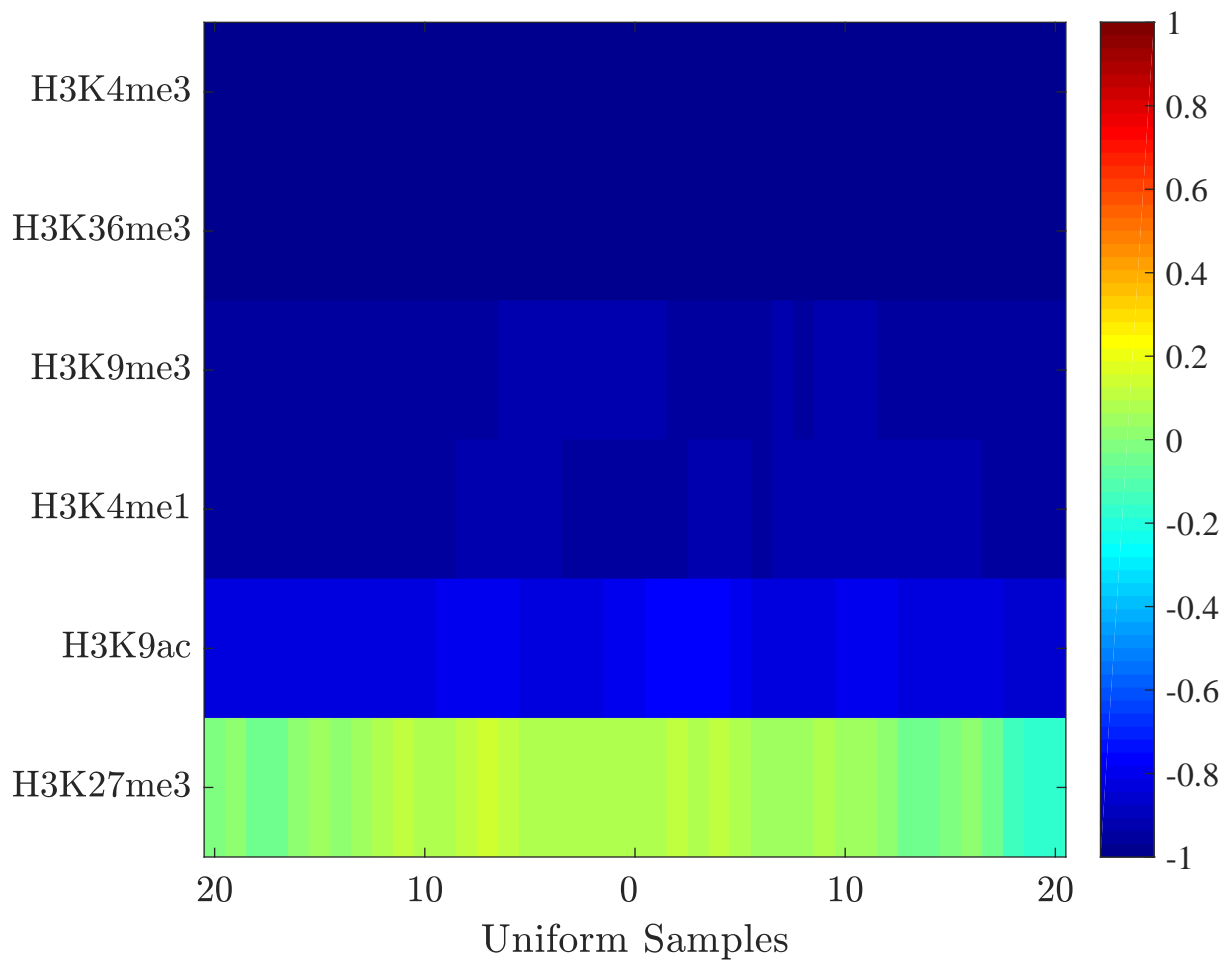

Supplement: Supplementary file 8 — HebbPlots of coding regions of inactive genes. This compressed file (.tar.gz) includes HebbPlots of genes inactive in 57 tissues/cell types. (TAR 2715 kb) [file 12859_2018_2312_MOESM8_ESM.tar › file9/E027.pdf]

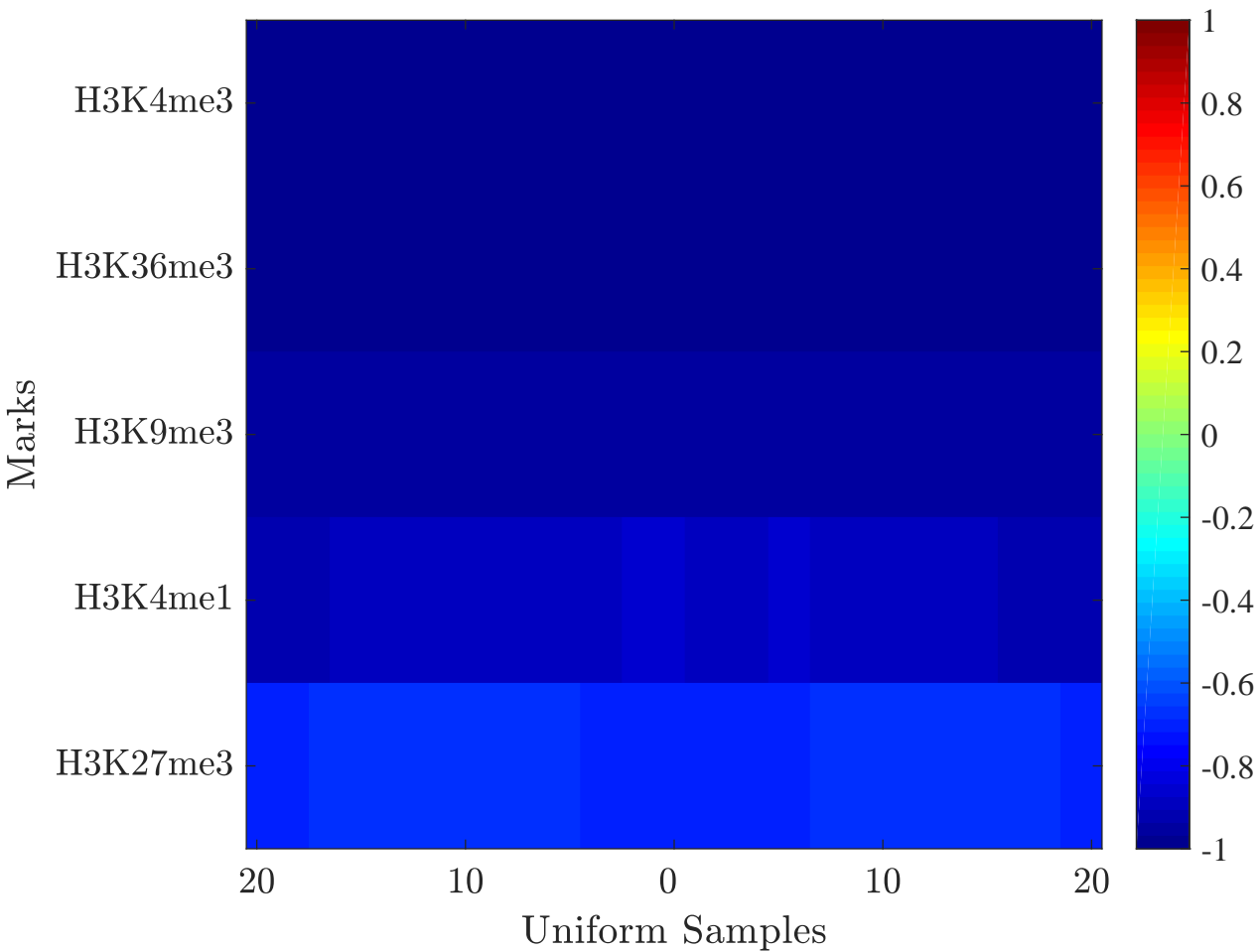

Supplement: Supplementary file 8 — HebbPlots of coding regions of inactive genes. This compressed file (.tar.gz) includes HebbPlots of genes inactive in 57 tissues/cell types. (TAR 2715 kb) [file 12859_2018_2312_MOESM8_ESM.tar › file9/E028.pdf]

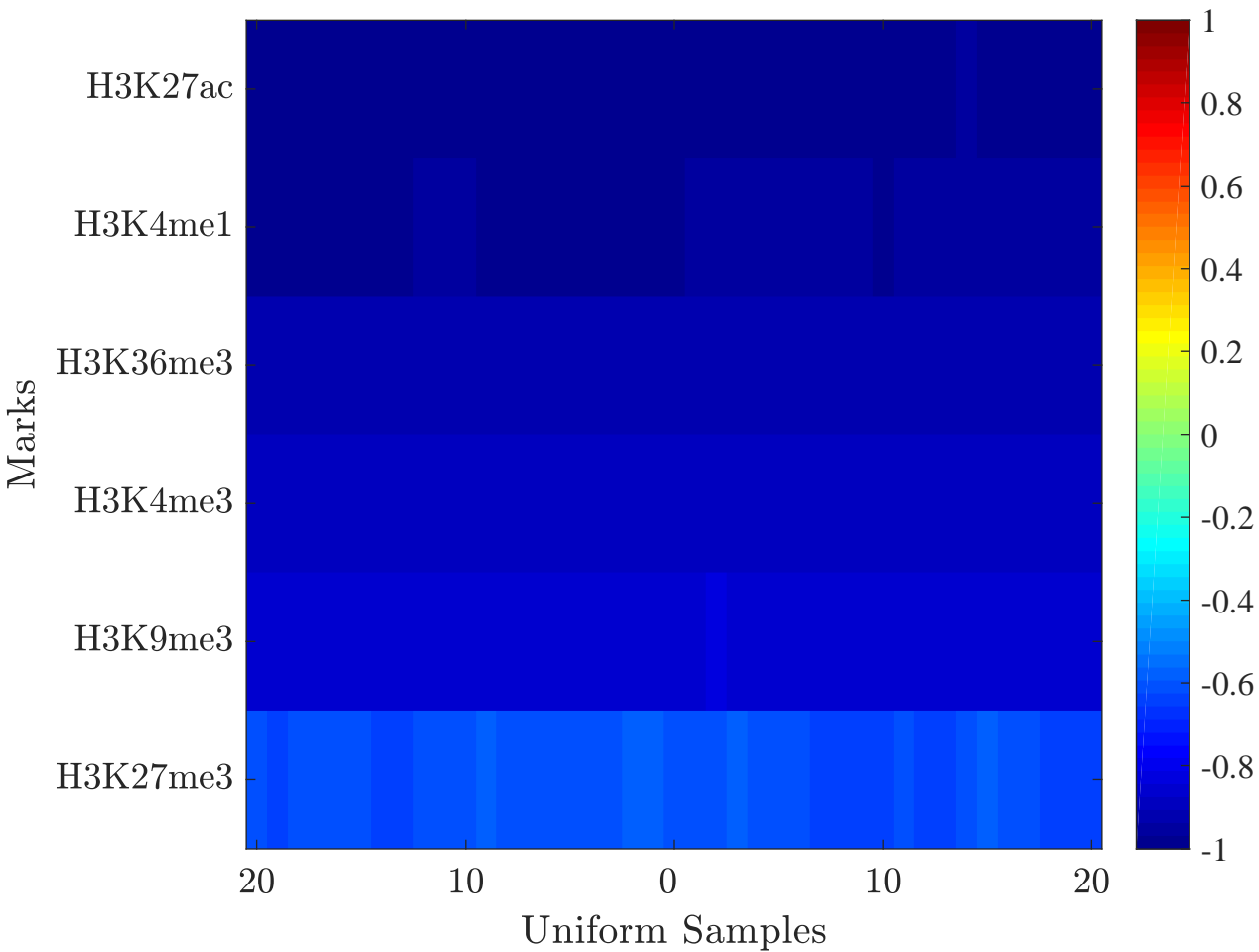

Supplement: Supplementary file 8 — HebbPlots of coding regions of inactive genes. This compressed file (.tar.gz) includes HebbPlots of genes inactive in 57 tissues/cell types. (TAR 2715 kb) [file 12859_2018_2312_MOESM8_ESM.tar › file9/E037.pdf]

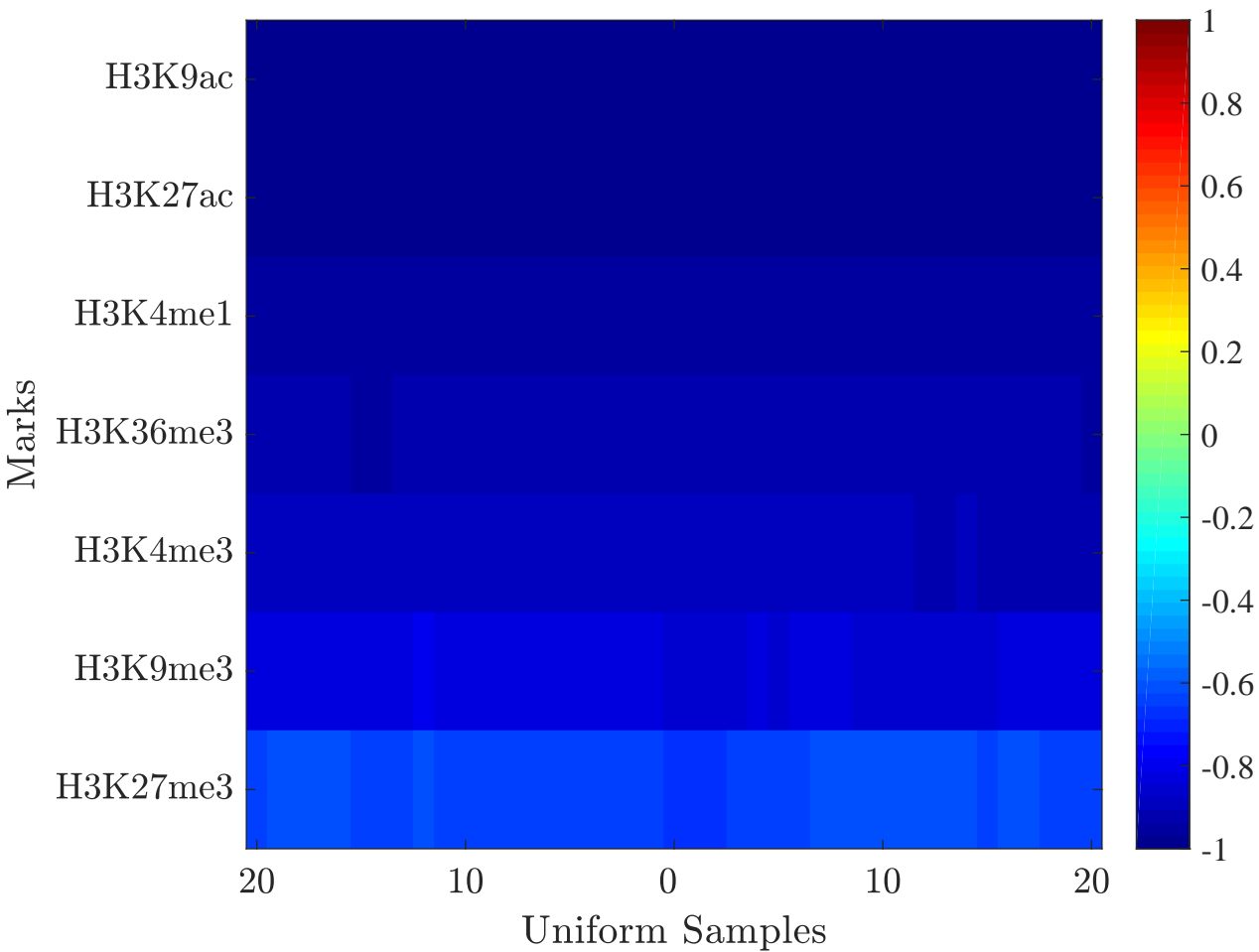

Supplement: Supplementary file 8 — HebbPlots of coding regions of inactive genes. This compressed file (.tar.gz) includes HebbPlots of genes inactive in 57 tissues/cell types. (TAR 2715 kb) [file 12859_2018_2312_MOESM8_ESM.tar › file9/E038.pdf]

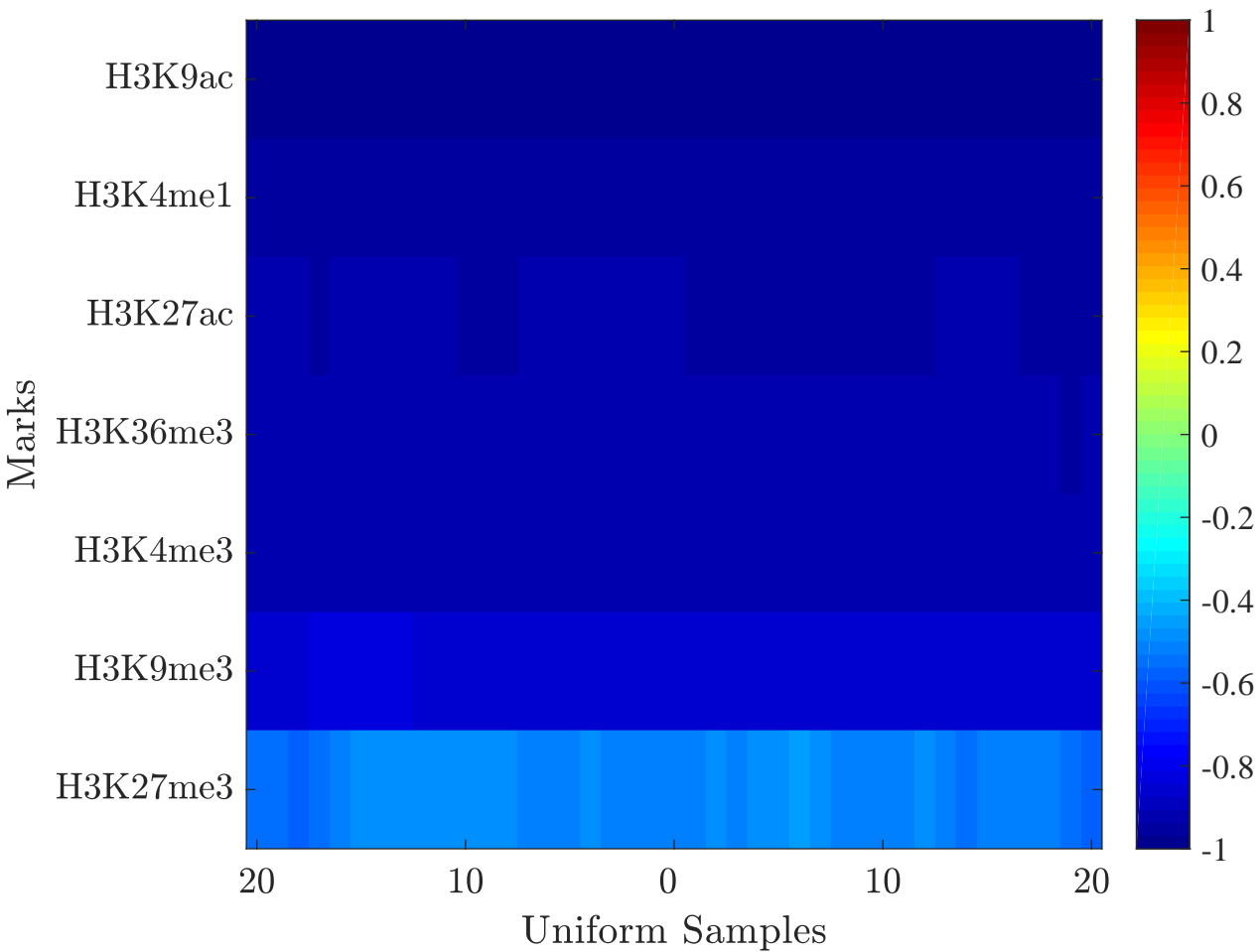

Supplement: Supplementary file 8 — HebbPlots of coding regions of inactive genes. This compressed file (.tar.gz) includes HebbPlots of genes inactive in 57 tissues/cell types. (TAR 2715 kb) [file 12859_2018_2312_MOESM8_ESM.tar › file9/E047.pdf]

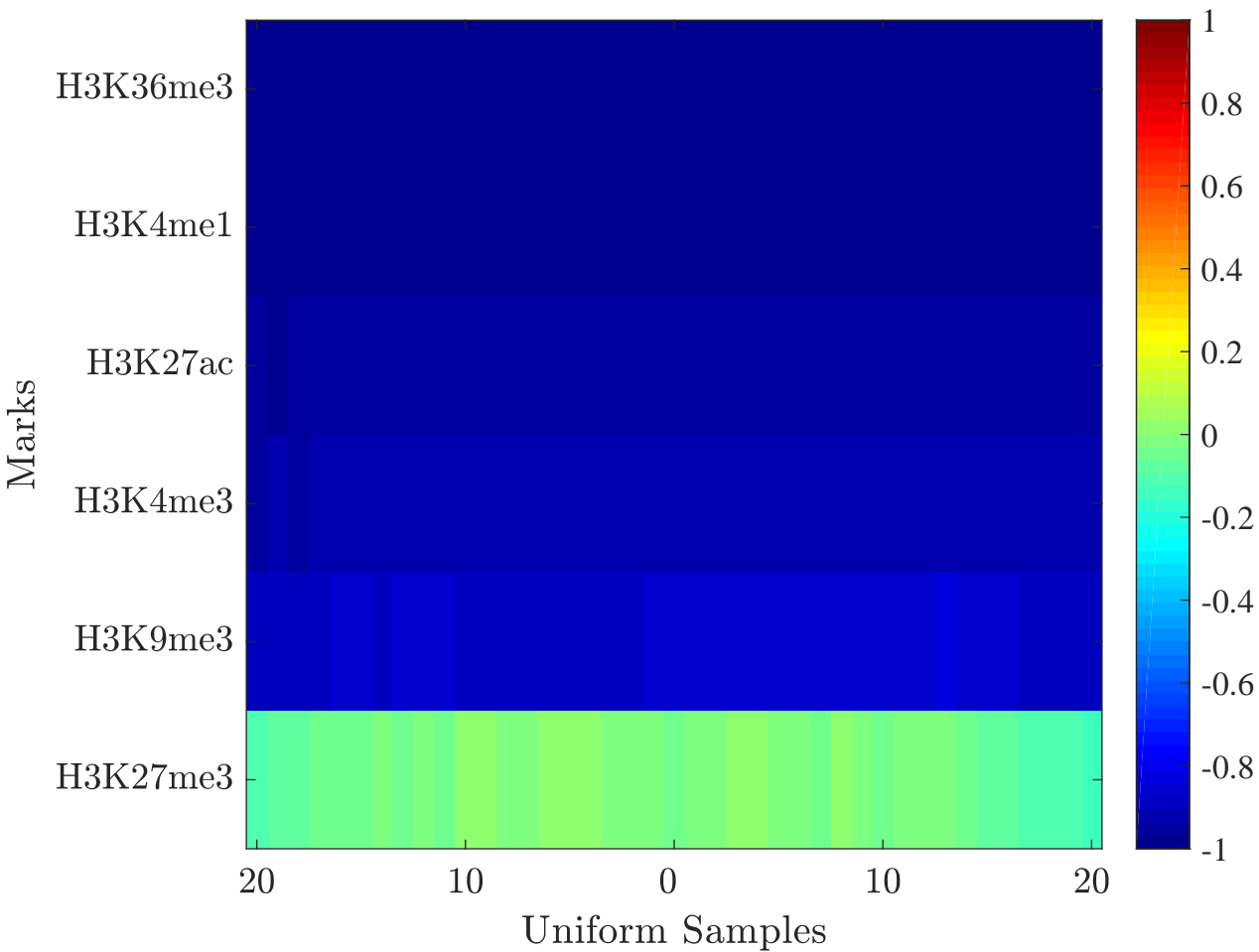

Supplement: Supplementary file 8 — HebbPlots of coding regions of inactive genes. This compressed file (.tar.gz) includes HebbPlots of genes inactive in 57 tissues/cell types. (TAR 2715 kb) [file 12859_2018_2312_MOESM8_ESM.tar › file9/E050.pdf]

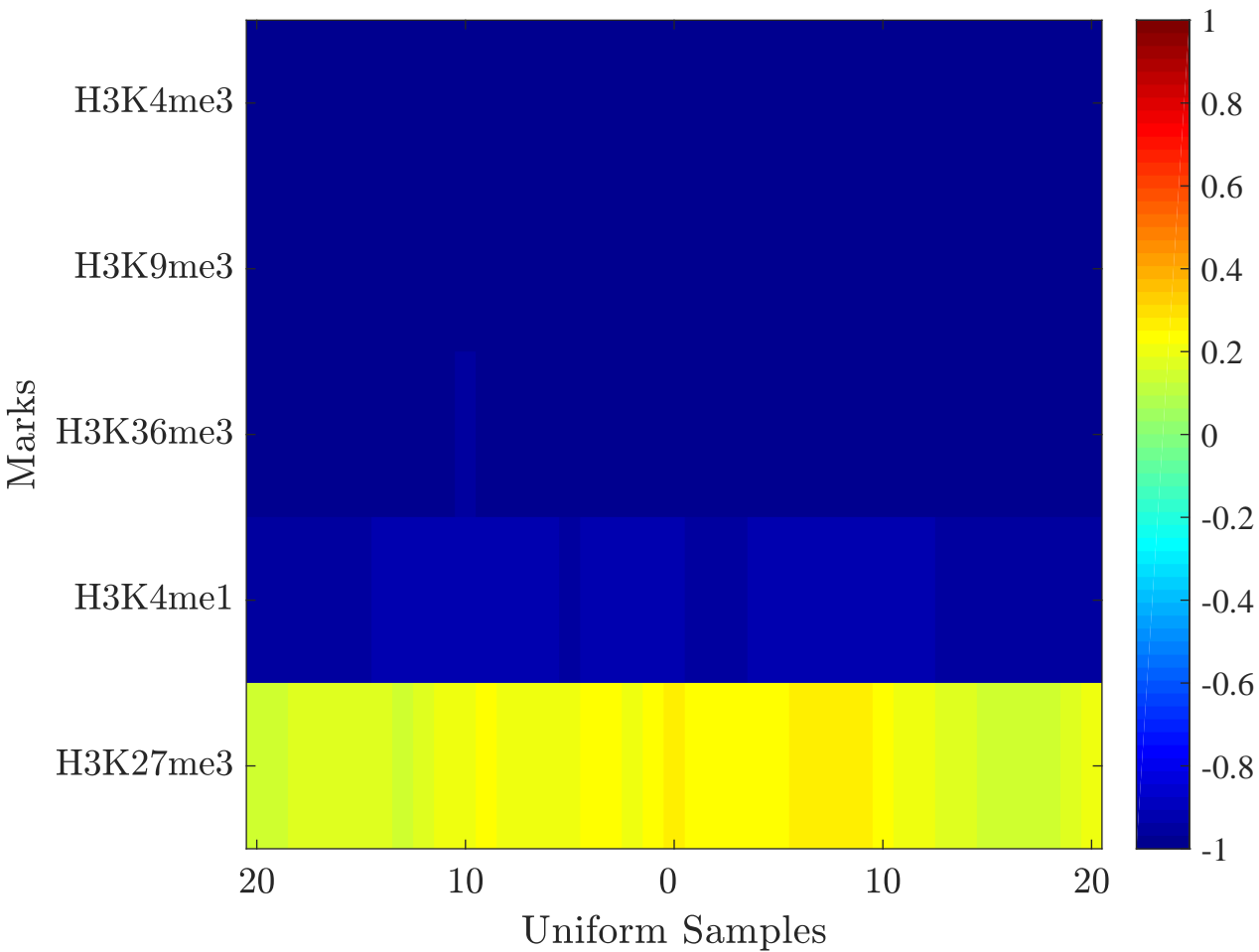

Supplement: Supplementary file 8 — HebbPlots of coding regions of inactive genes. This compressed file (.tar.gz) includes HebbPlots of genes inactive in 57 tissues/cell types. (TAR 2715 kb) [file 12859_2018_2312_MOESM8_ESM.tar › file9/E053.pdf]

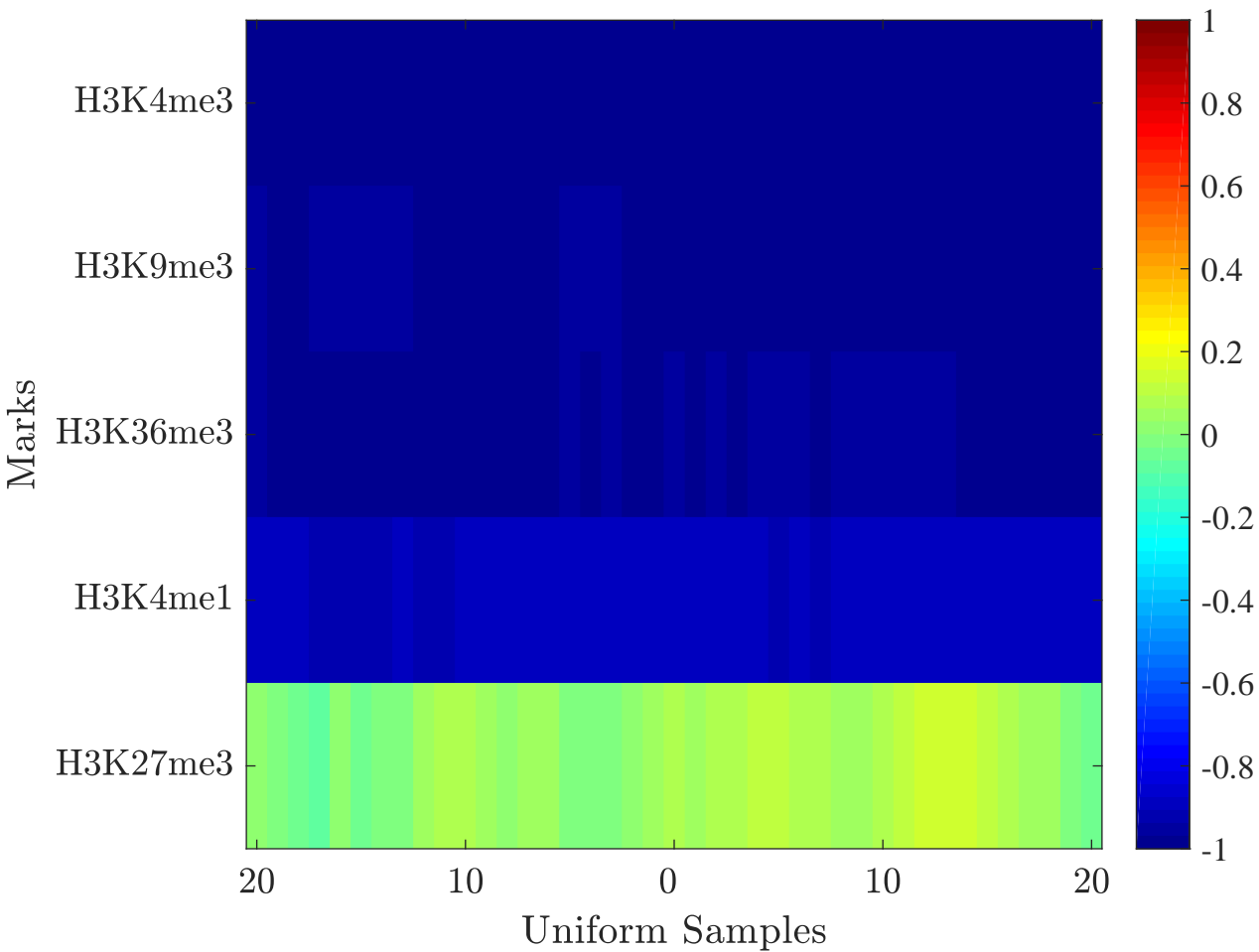

Supplement: Supplementary file 8 — HebbPlots of coding regions of inactive genes. This compressed file (.tar.gz) includes HebbPlots of genes inactive in 57 tissues/cell types. (TAR 2715 kb) [file 12859_2018_2312_MOESM8_ESM.tar › file9/E054.pdf]

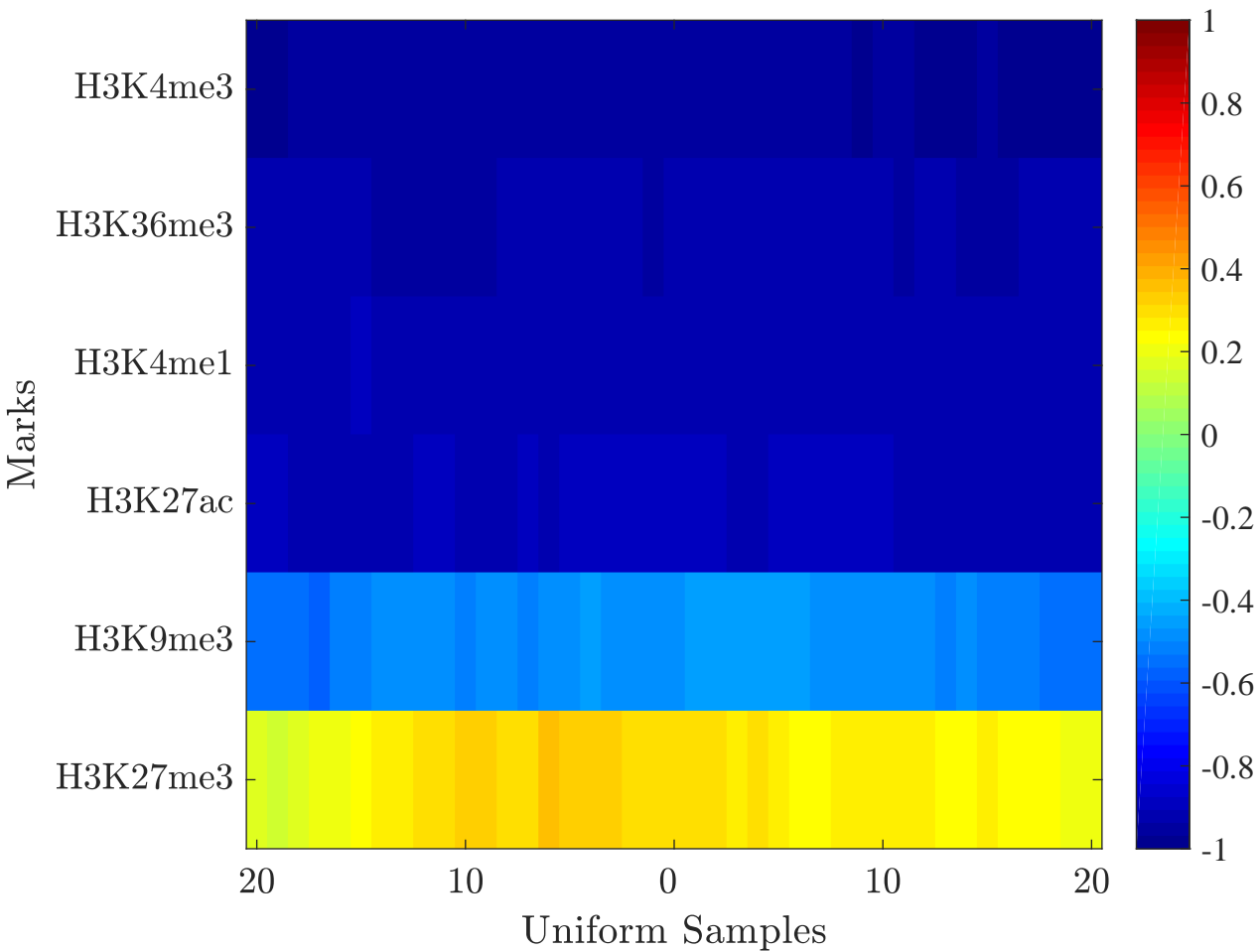

Supplement: Supplementary file 8 — HebbPlots of coding regions of inactive genes. This compressed file (.tar.gz) includes HebbPlots of genes inactive in 57 tissues/cell types. (TAR 2715 kb) [file 12859_2018_2312_MOESM8_ESM.tar › file9/E055.pdf]

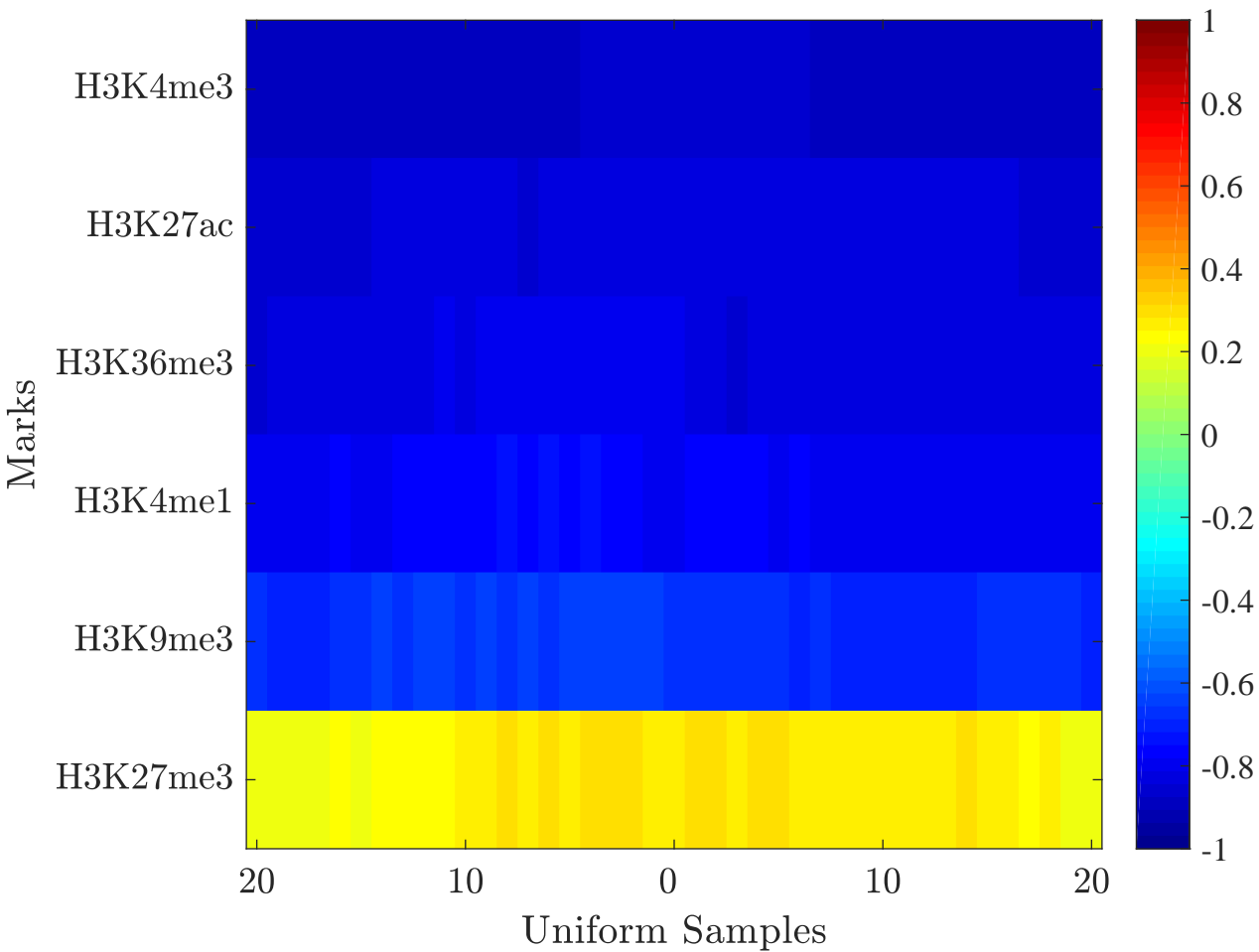

Supplement: Supplementary file 8 — HebbPlots of coding regions of inactive genes. This compressed file (.tar.gz) includes HebbPlots of genes inactive in 57 tissues/cell types. (TAR 2715 kb) [file 12859_2018_2312_MOESM8_ESM.tar › file9/E056.pdf]

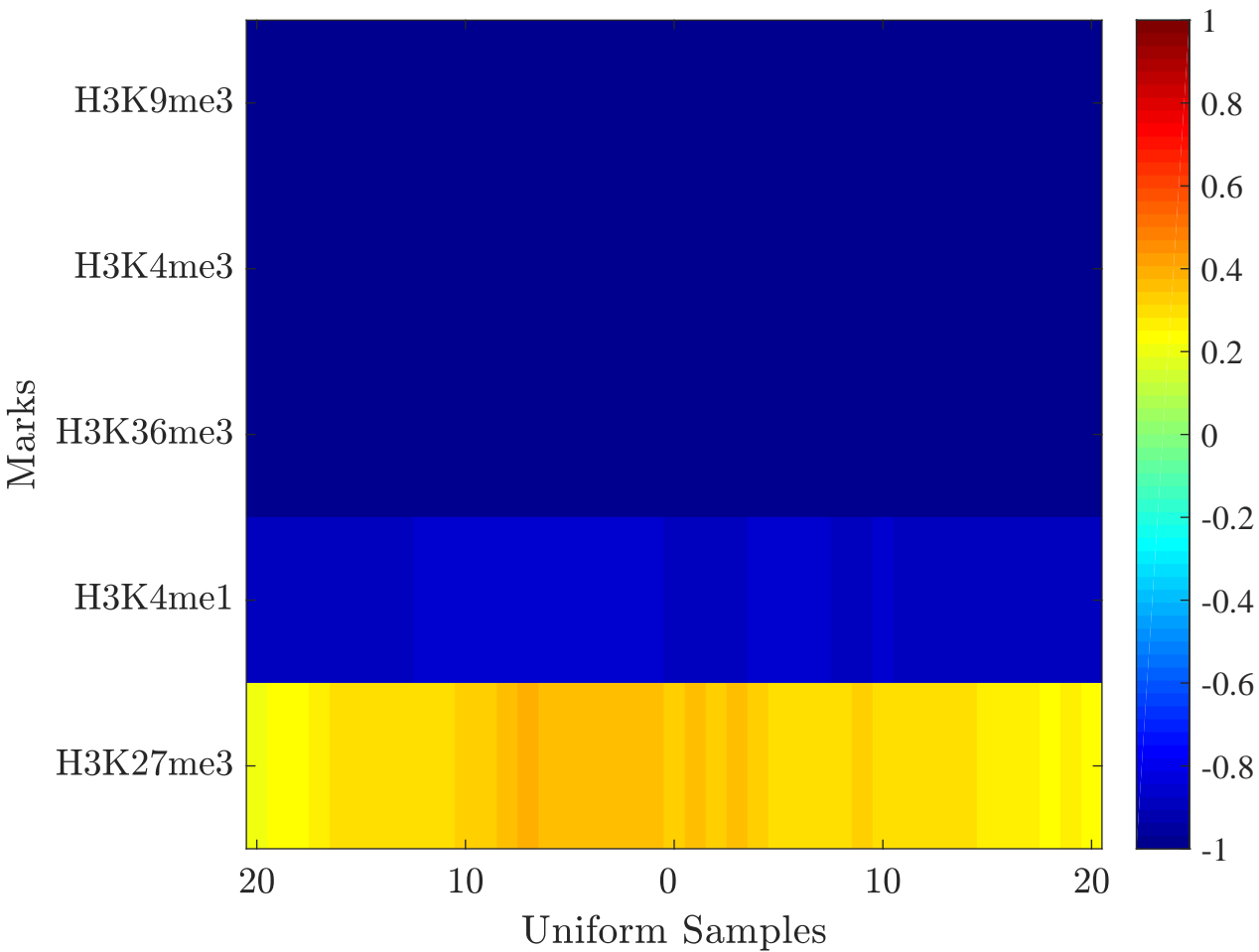

Supplement: Supplementary file 8 — HebbPlots of coding regions of inactive genes. This compressed file (.tar.gz) includes HebbPlots of genes inactive in 57 tissues/cell types. (TAR 2715 kb) [file 12859_2018_2312_MOESM8_ESM.tar › file9/E057.pdf]

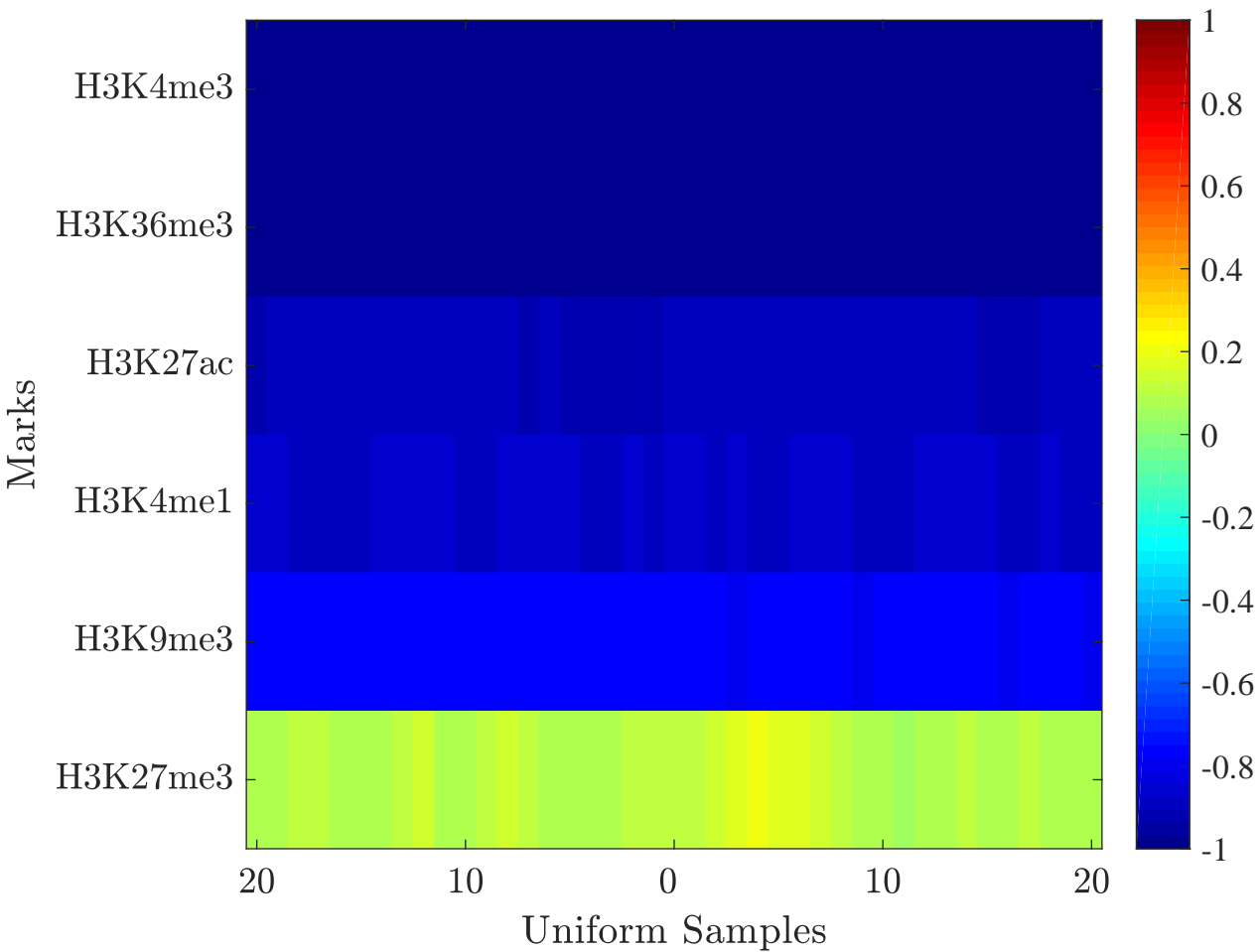

Supplement: Supplementary file 8 — HebbPlots of coding regions of inactive genes. This compressed file (.tar.gz) includes HebbPlots of genes inactive in 57 tissues/cell types. (TAR 2715 kb) [file 12859_2018_2312_MOESM8_ESM.tar › file9/E058.pdf]

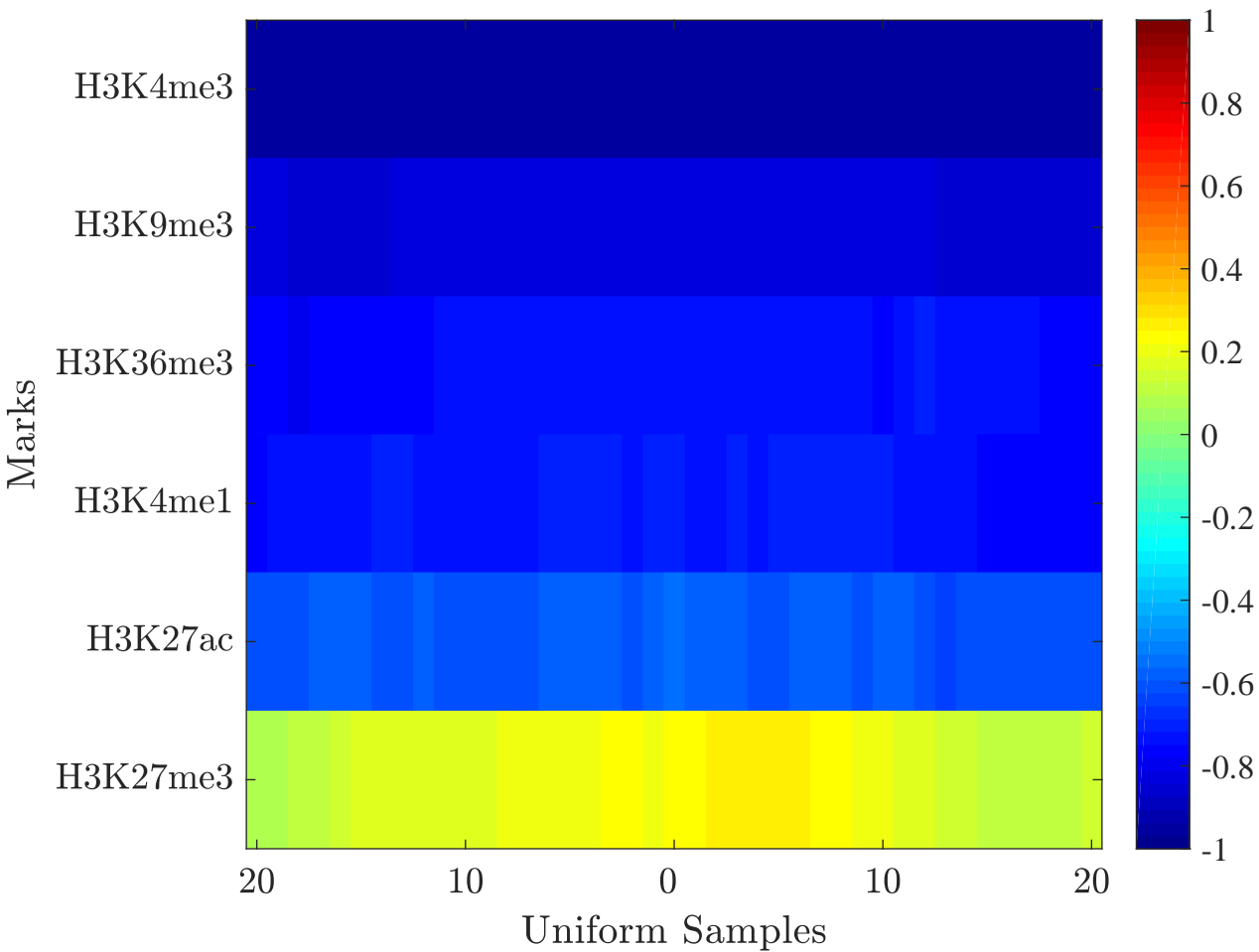

Supplement: Supplementary file 8 — HebbPlots of coding regions of inactive genes. This compressed file (.tar.gz) includes HebbPlots of genes inactive in 57 tissues/cell types. (TAR 2715 kb) [file 12859_2018_2312_MOESM8_ESM.tar › file9/E059.pdf]

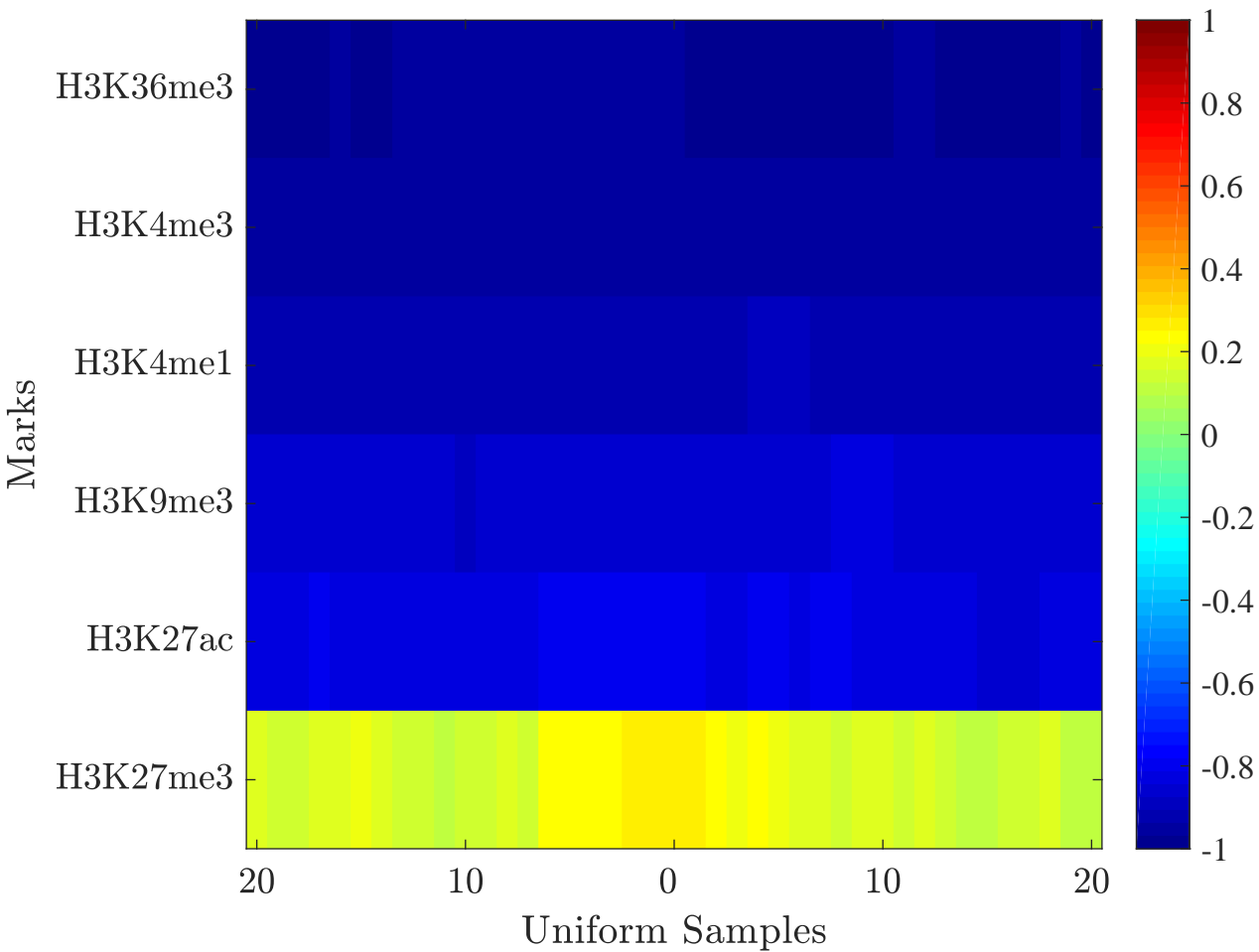

Supplement: Supplementary file 8 — HebbPlots of coding regions of inactive genes. This compressed file (.tar.gz) includes HebbPlots of genes inactive in 57 tissues/cell types. (TAR 2715 kb) [file 12859_2018_2312_MOESM8_ESM.tar › file9/E061.pdf]

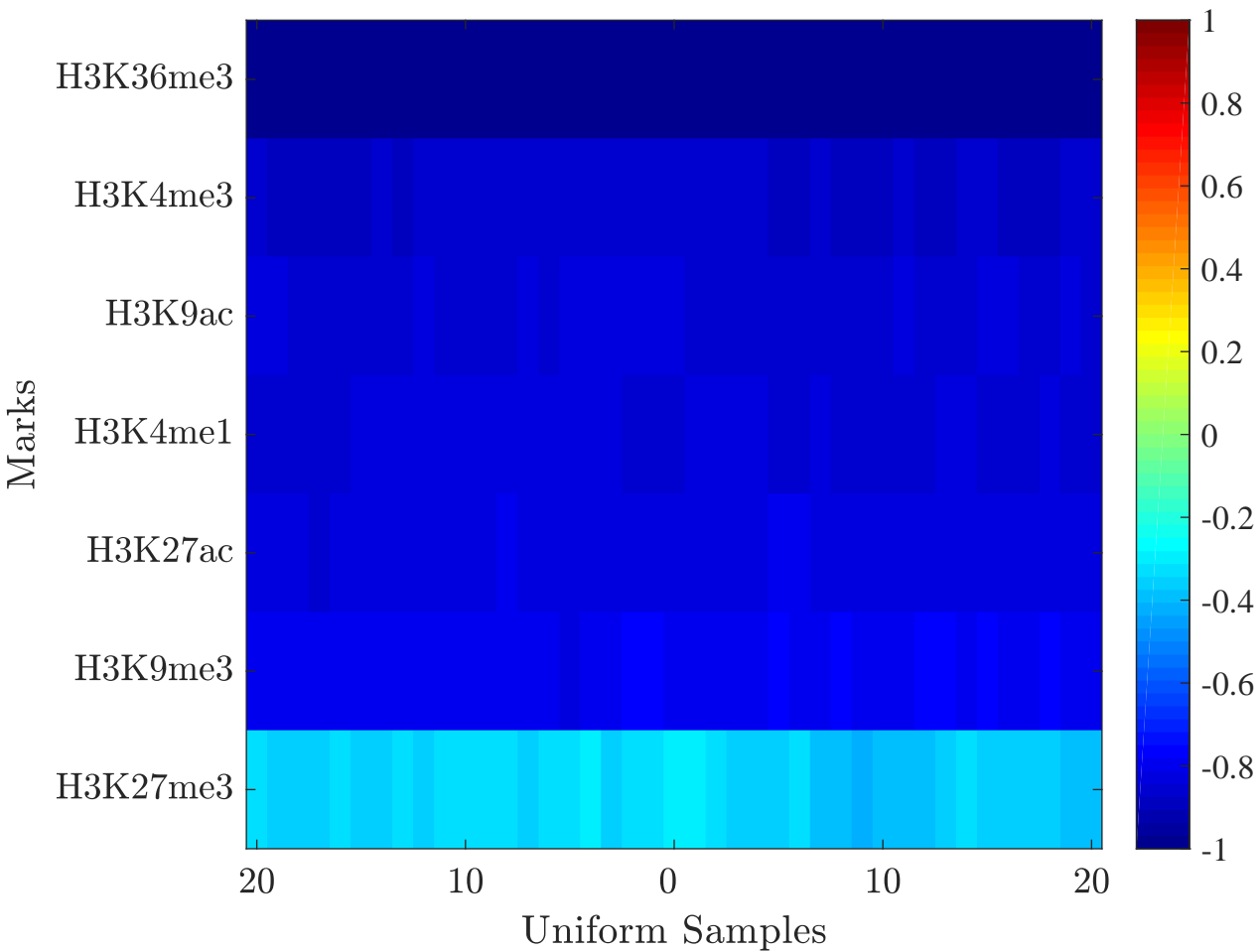

Supplement: Supplementary file 8 — HebbPlots of coding regions of inactive genes. This compressed file (.tar.gz) includes HebbPlots of genes inactive in 57 tissues/cell types. (TAR 2715 kb) [file 12859_2018_2312_MOESM8_ESM.tar › file9/E062.pdf]

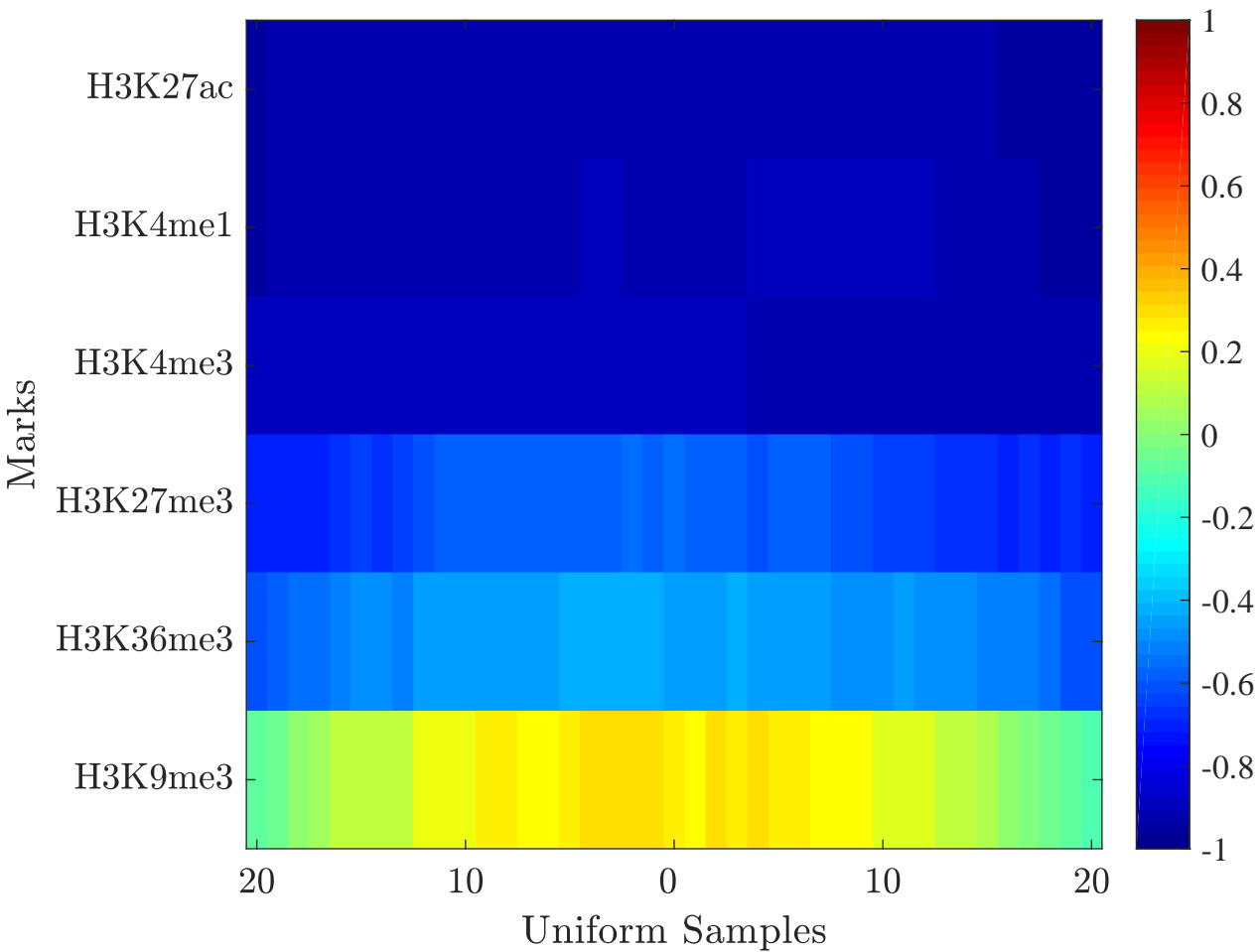

Supplement: Supplementary file 8 — HebbPlots of coding regions of inactive genes. This compressed file (.tar.gz) includes HebbPlots of genes inactive in 57 tissues/cell types. (TAR 2715 kb) [file 12859_2018_2312_MOESM8_ESM.tar › file9/E065.pdf]

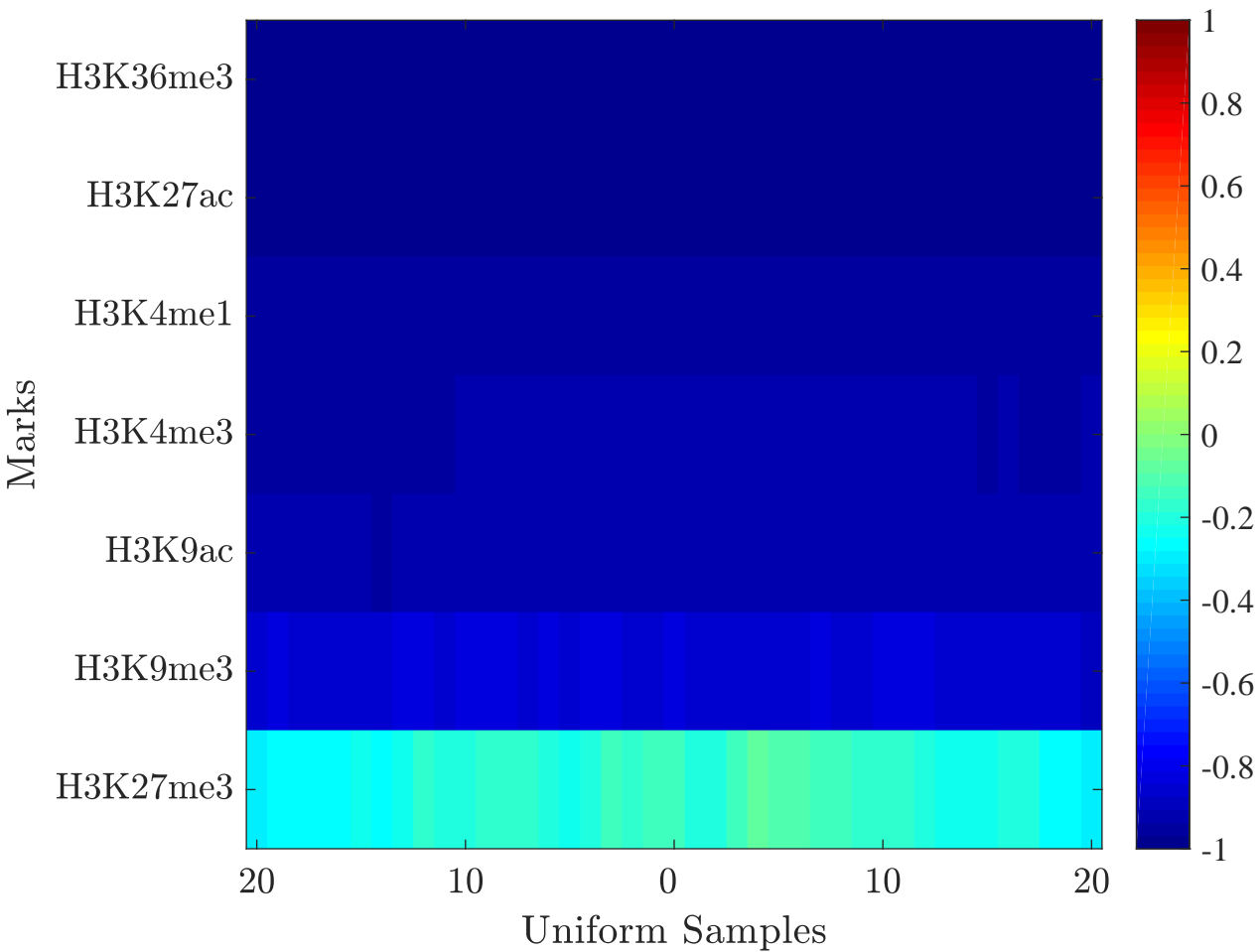

Supplement: Supplementary file 8 — HebbPlots of coding regions of inactive genes. This compressed file (.tar.gz) includes HebbPlots of genes inactive in 57 tissues/cell types. (TAR 2715 kb) [file 12859_2018_2312_MOESM8_ESM.tar › file9/E066.pdf]

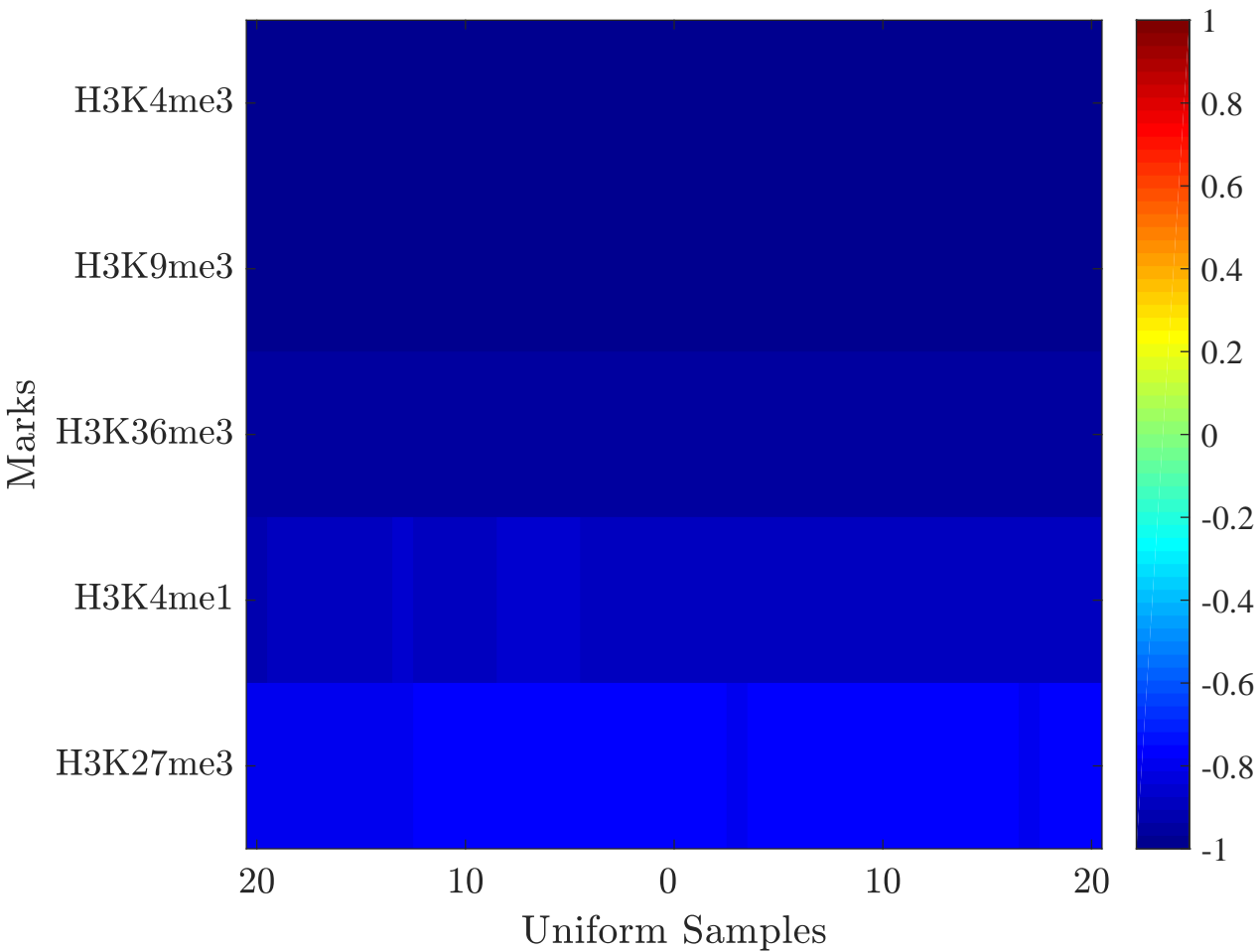

Supplement: Supplementary file 8 — HebbPlots of coding regions of inactive genes. This compressed file (.tar.gz) includes HebbPlots of genes inactive in 57 tissues/cell types. (TAR 2715 kb) [file 12859_2018_2312_MOESM8_ESM.tar › file9/E070.pdf]

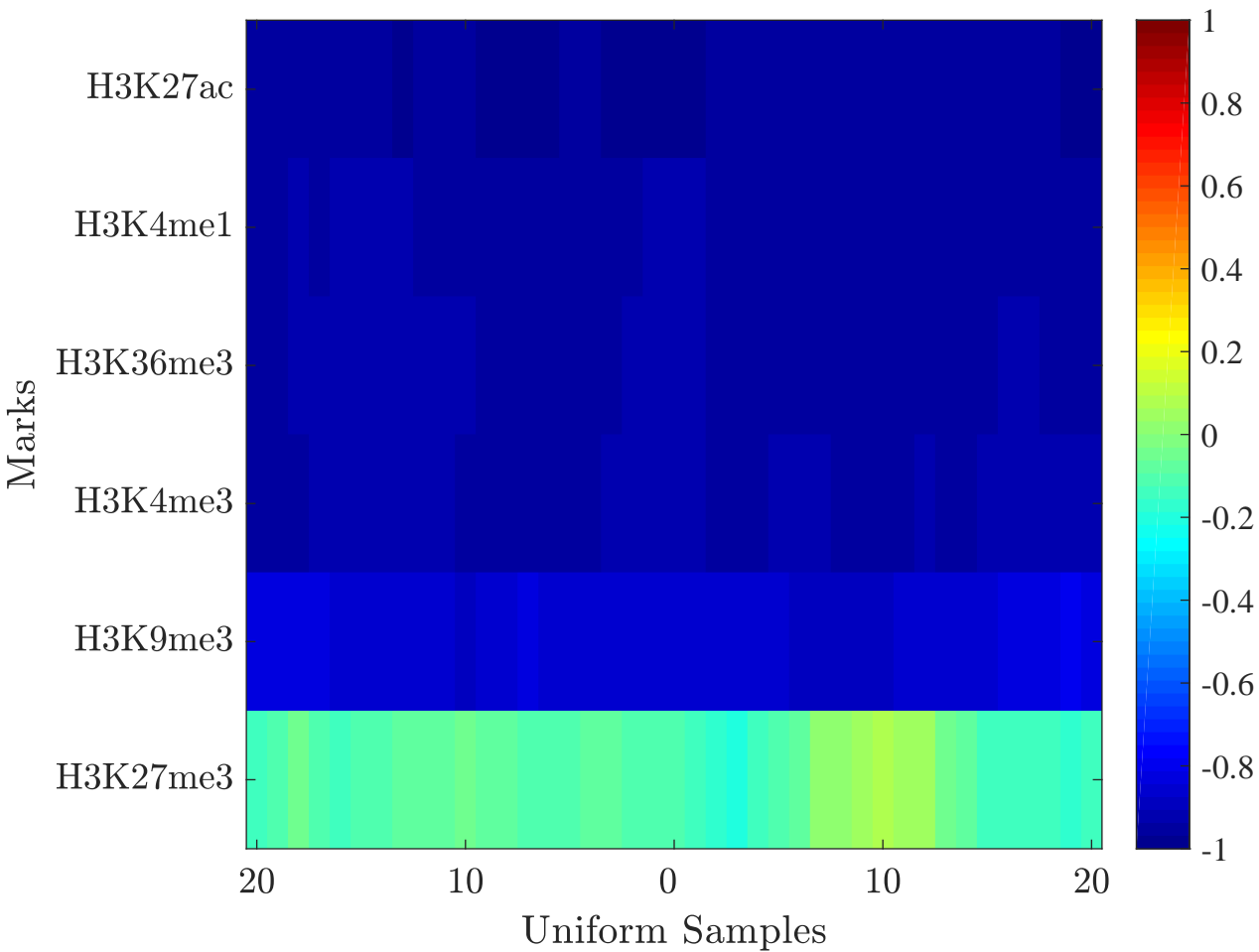

Supplement: Supplementary file 8 — HebbPlots of coding regions of inactive genes. This compressed file (.tar.gz) includes HebbPlots of genes inactive in 57 tissues/cell types. (TAR 2715 kb) [file 12859_2018_2312_MOESM8_ESM.tar › file9/E071.pdf]

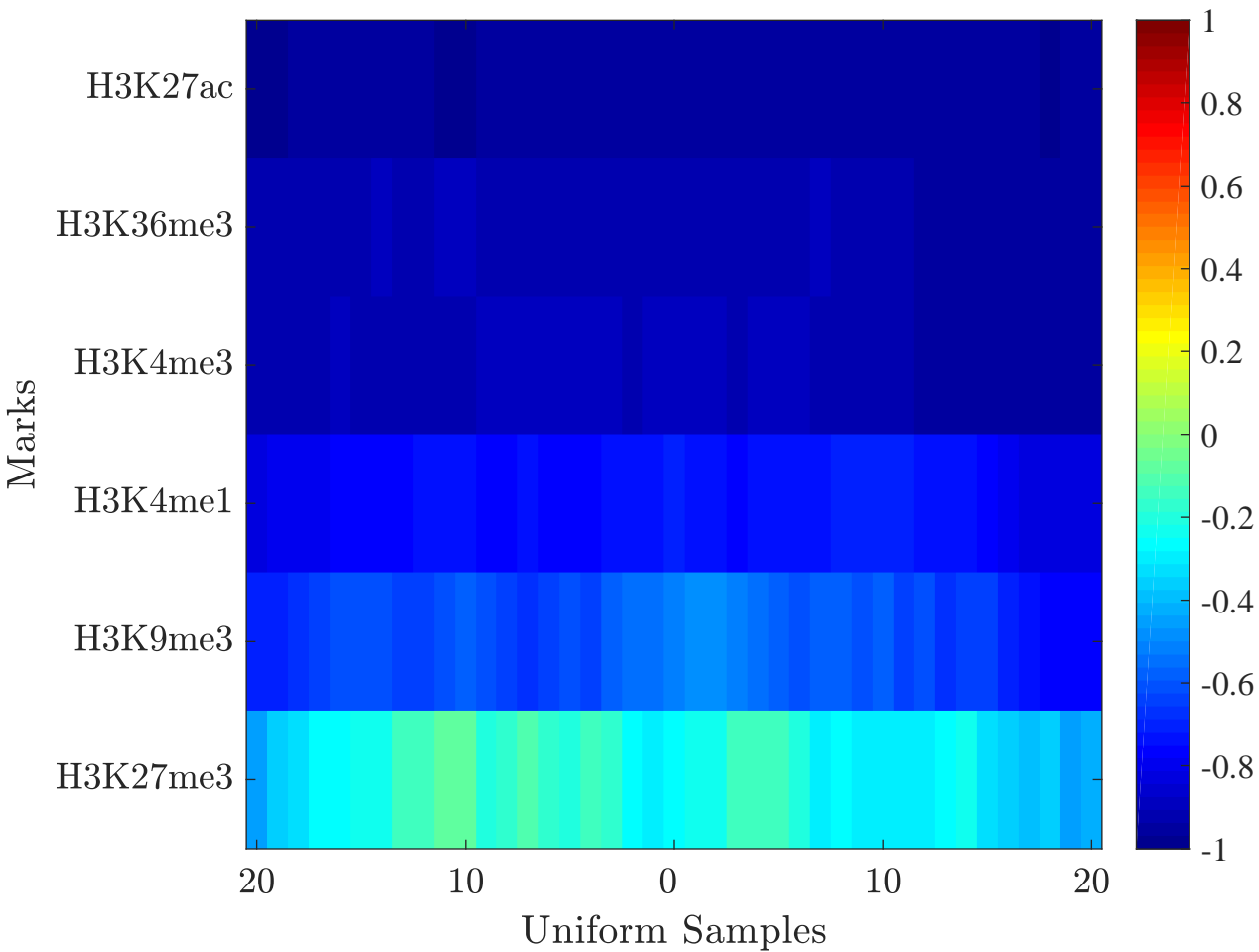

Supplement: Supplementary file 8 — HebbPlots of coding regions of inactive genes. This compressed file (.tar.gz) includes HebbPlots of genes inactive in 57 tissues/cell types. (TAR 2715 kb) [file 12859_2018_2312_MOESM8_ESM.tar › file9/E079.pdf]

Marks

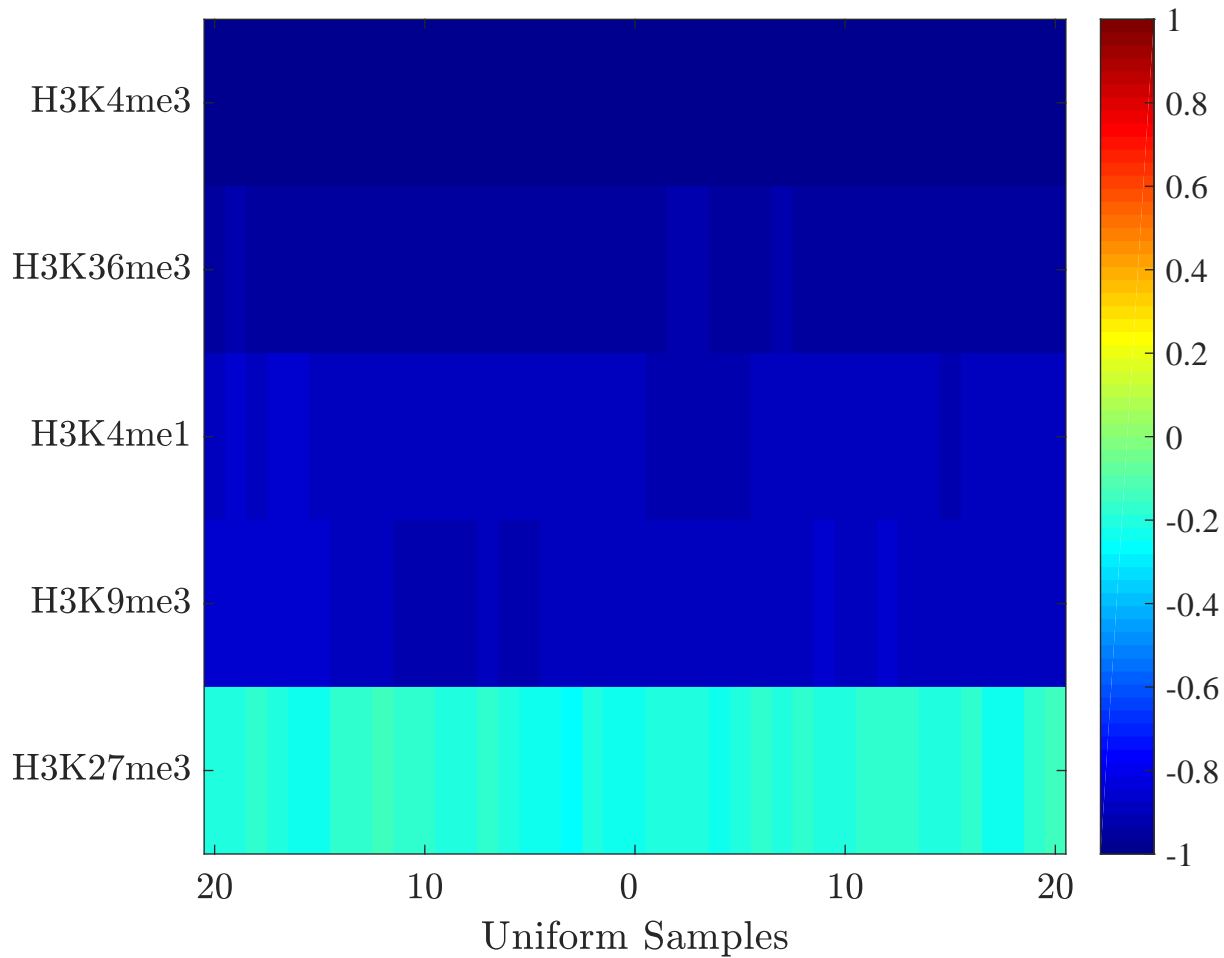

Supplement: Supplementary file 8 — HebbPlots of coding regions of inactive genes. This compressed file (.tar.gz) includes HebbPlots of genes inactive in 57 tissues/cell types. (TAR 2715 kb) [file 12859_2018_2312_MOESM8_ESM.tar › file9/E082.pdf]

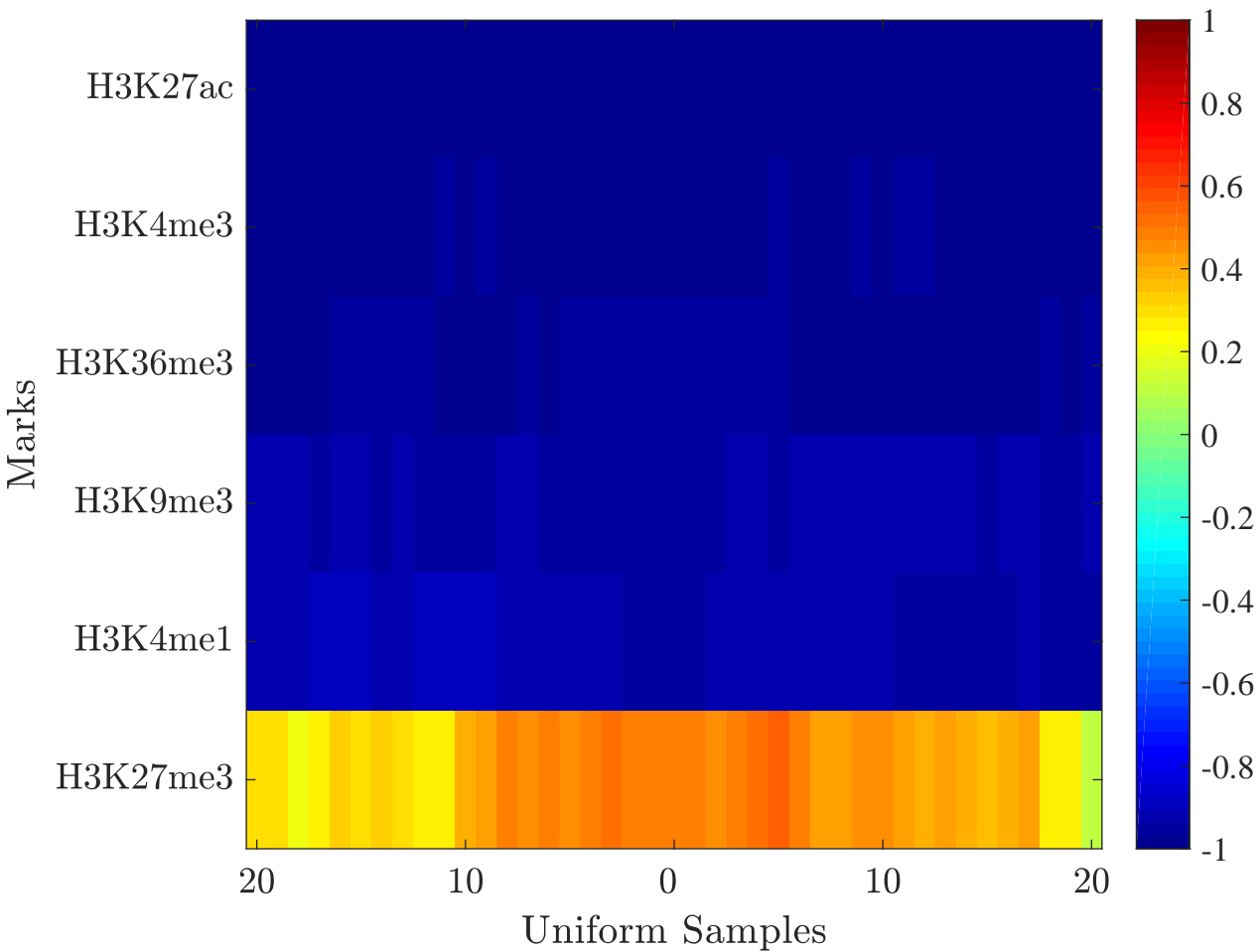

Supplement: Supplementary file 8 — HebbPlots of coding regions of inactive genes. This compressed file (.tar.gz) includes HebbPlots of genes inactive in 57 tissues/cell types. (TAR 2715 kb) [file 12859_2018_2312_MOESM8_ESM.tar › file9/E084.pdf]

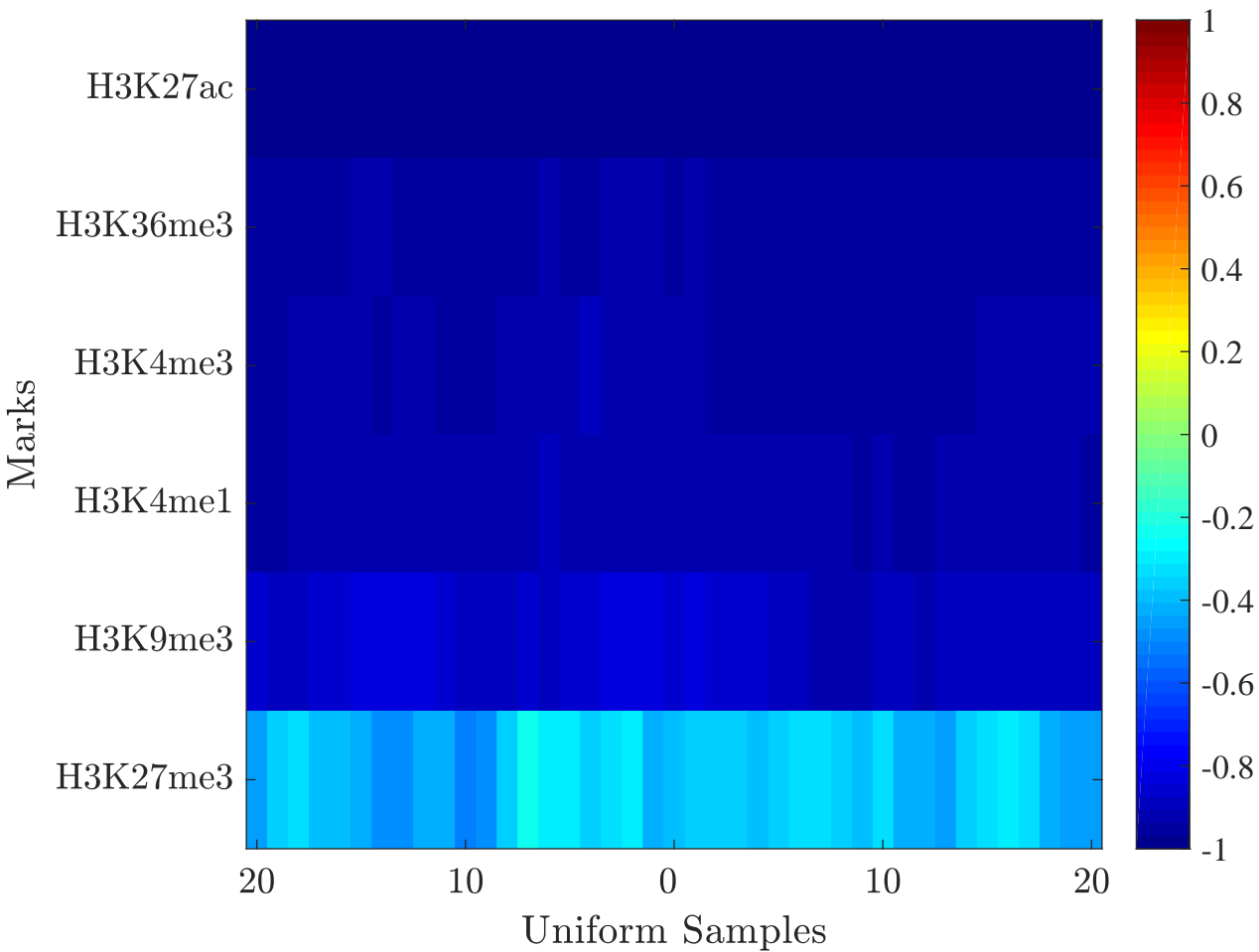

Supplement: Supplementary file 8 — HebbPlots of coding regions of inactive genes. This compressed file (.tar.gz) includes HebbPlots of genes inactive in 57 tissues/cell types. (TAR 2715 kb) [file 12859_2018_2312_MOESM8_ESM.tar › file9/E085.pdf]

Marks

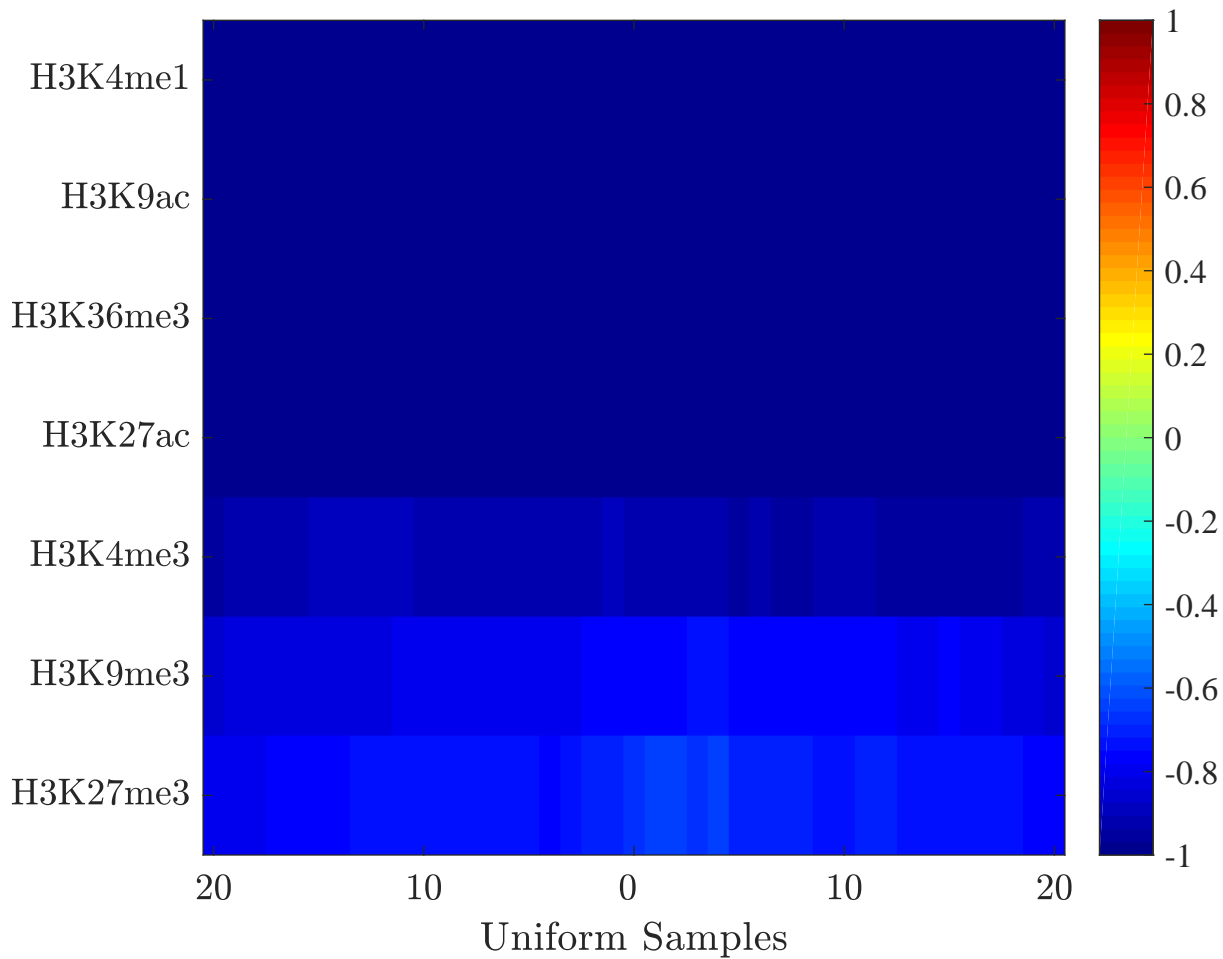

Supplement: Supplementary file 8 — HebbPlots of coding regions of inactive genes. This compressed file (.tar.gz) includes HebbPlots of genes inactive in 57 tissues/cell types. (TAR 2715 kb) [file 12859_2018_2312_MOESM8_ESM.tar › file9/E087.pdf]

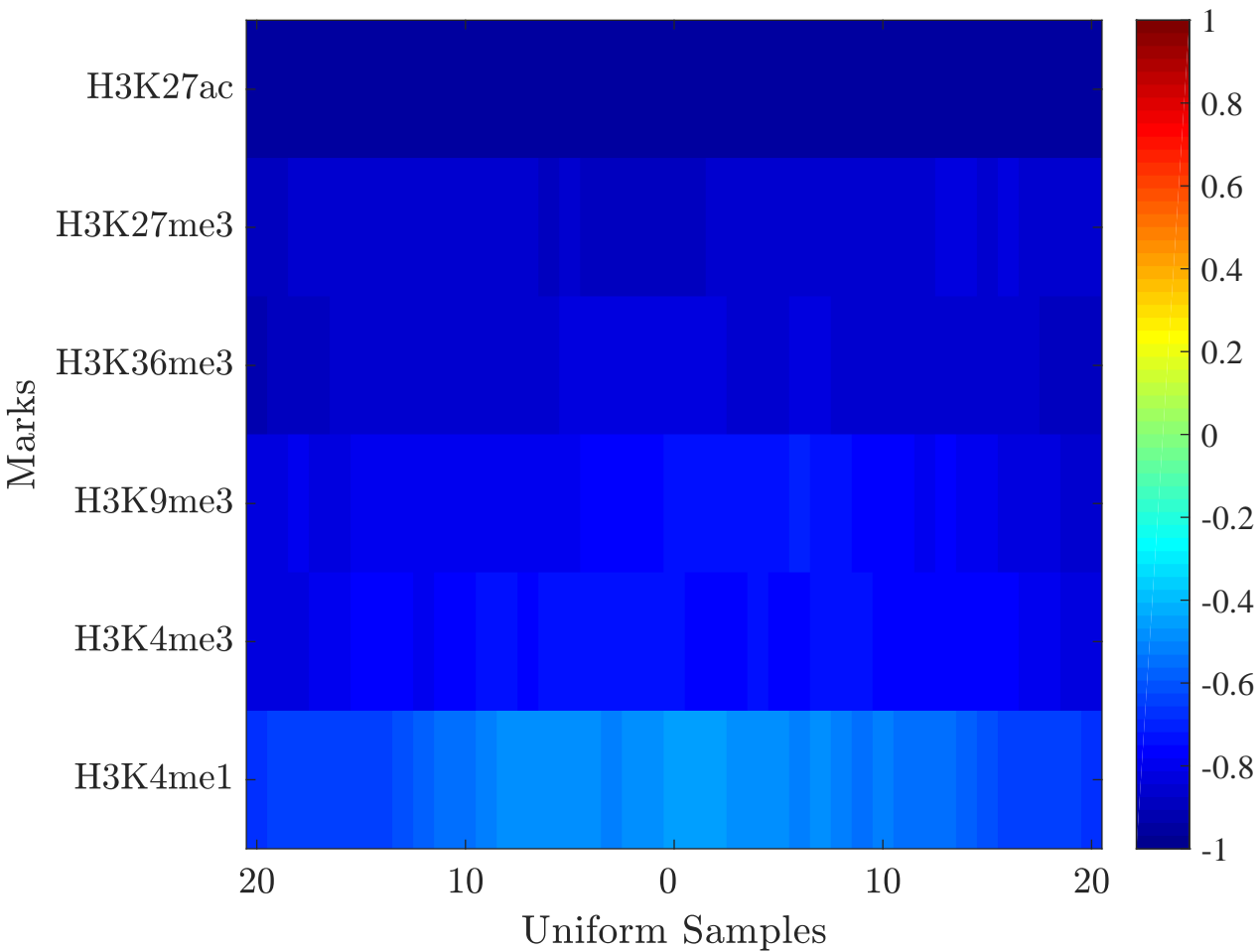

Supplement: Supplementary file 8 — HebbPlots of coding regions of inactive genes. This compressed file (.tar.gz) includes HebbPlots of genes inactive in 57 tissues/cell types. (TAR 2715 kb) [file 12859_2018_2312_MOESM8_ESM.tar › file9/E094.pdf]

Marks

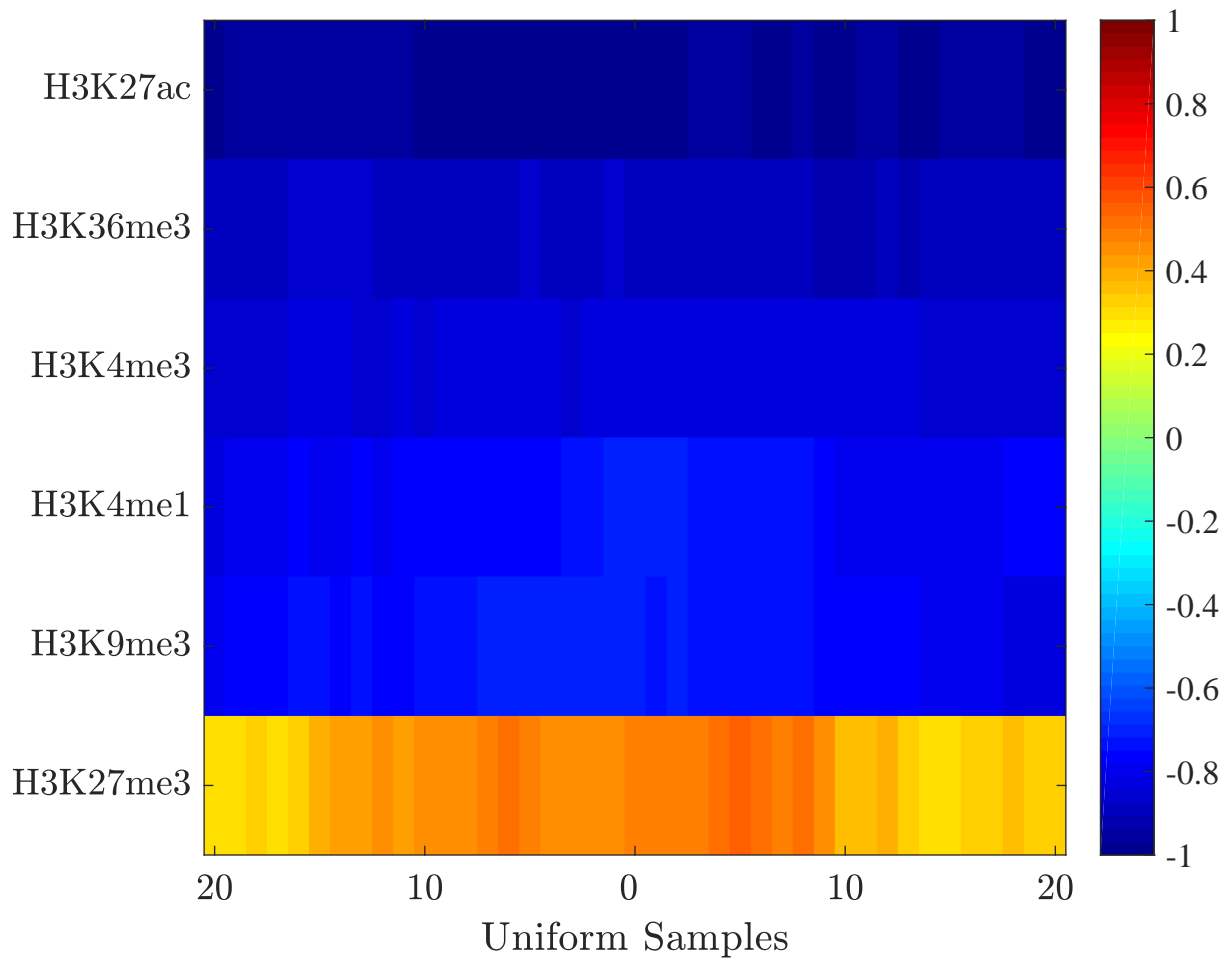

Supplement: Supplementary file 8 — HebbPlots of coding regions of inactive genes. This compressed file (.tar.gz) includes HebbPlots of genes inactive in 57 tissues/cell types. (TAR 2715 kb) [file 12859_2018_2312_MOESM8_ESM.tar › file9/E095.pdf]

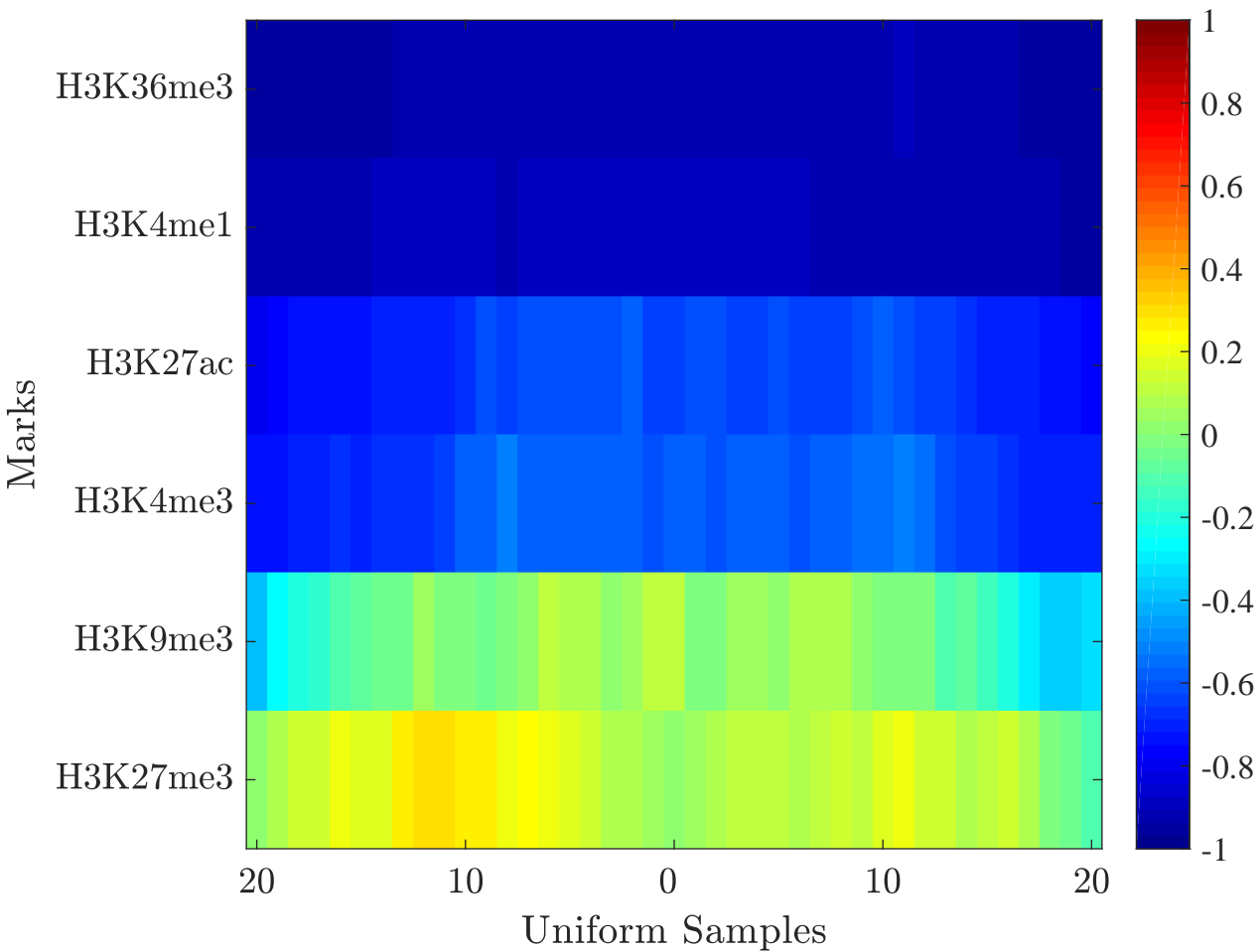

Supplement: Supplementary file 8 — HebbPlots of coding regions of inactive genes. This compressed file (.tar.gz) includes HebbPlots of genes inactive in 57 tissues/cell types. (TAR 2715 kb) [file 12859_2018_2312_MOESM8_ESM.tar › file9/E096.pdf]

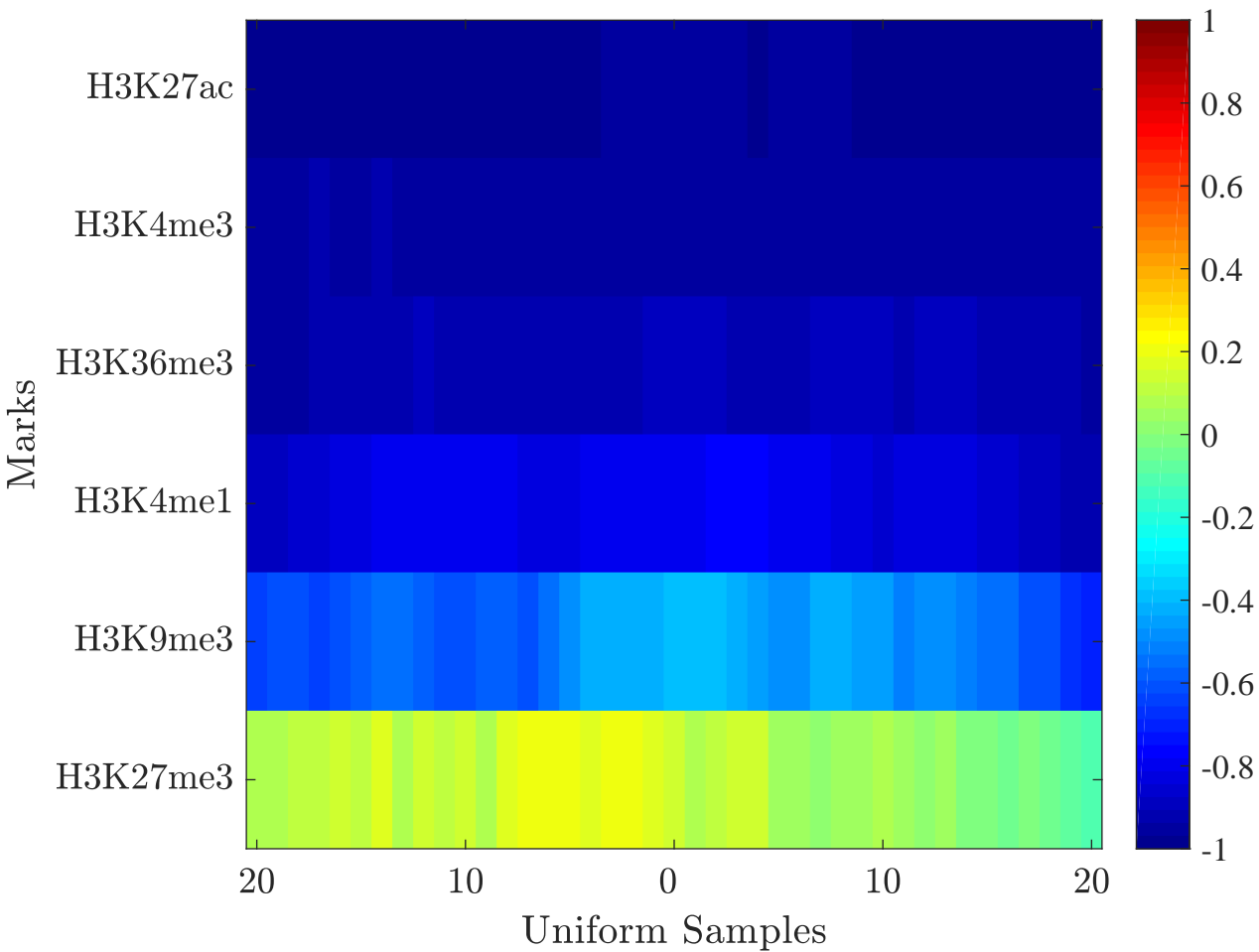

Supplement: Supplementary file 8 — HebbPlots of coding regions of inactive genes. This compressed file (.tar.gz) includes HebbPlots of genes inactive in 57 tissues/cell types. (TAR 2715 kb) [file 12859_2018_2312_MOESM8_ESM.tar › file9/E097.pdf]

Marks

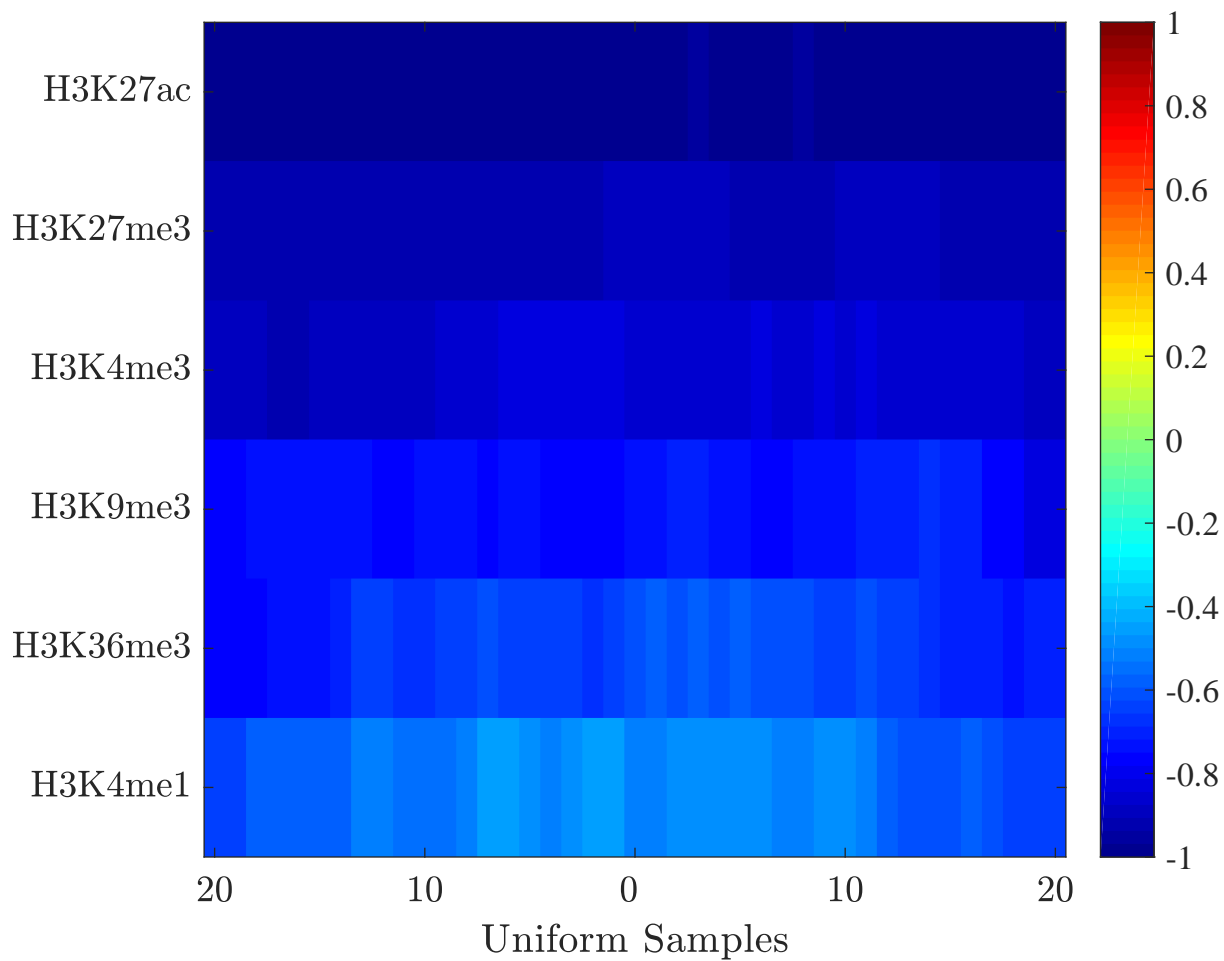

Supplement: Supplementary file 8 — HebbPlots of coding regions of inactive genes. This compressed file (.tar.gz) includes HebbPlots of genes inactive in 57 tissues/cell types. (TAR 2715 kb) [file 12859_2018_2312_MOESM8_ESM.tar › file9/E098.pdf]

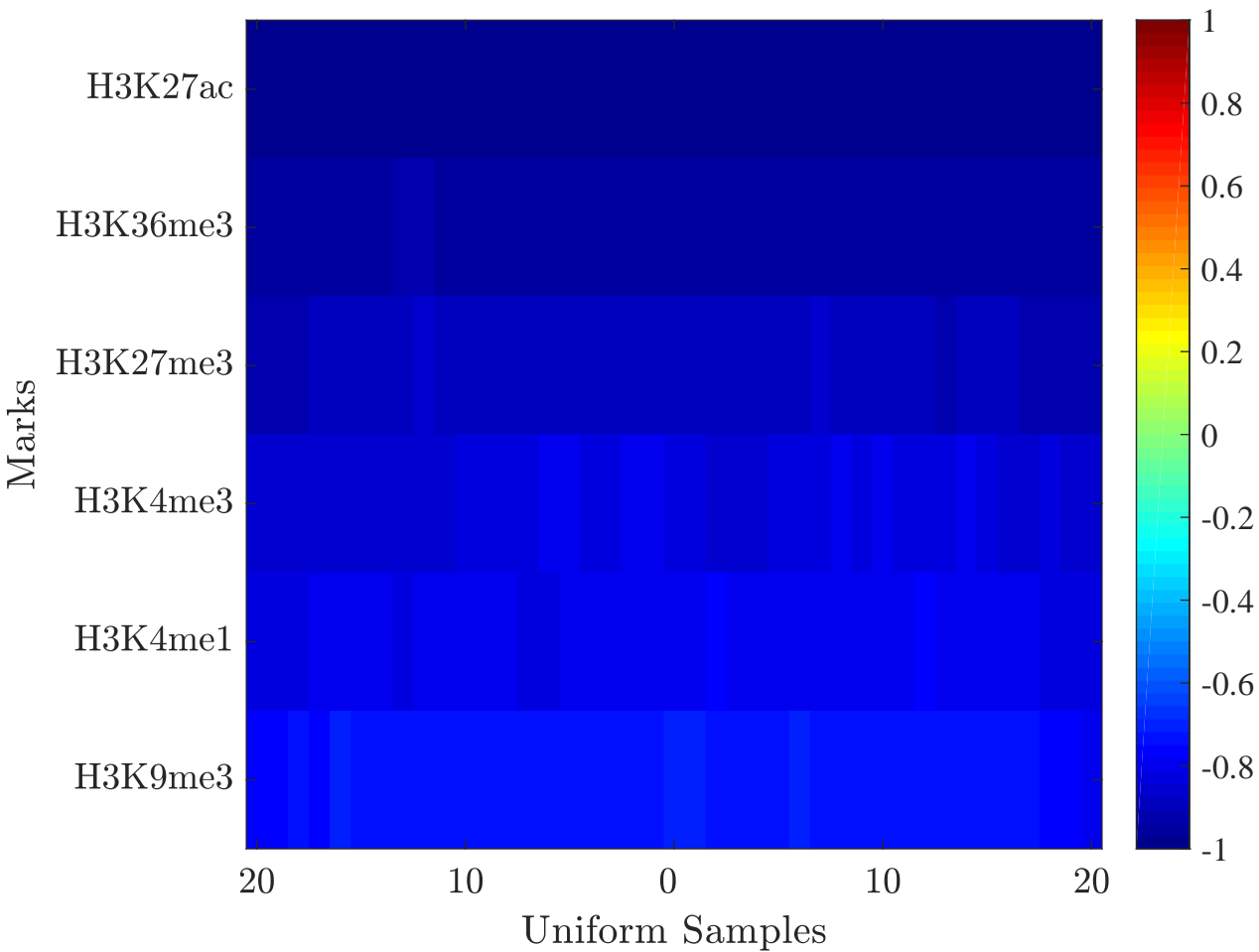

Supplement: Supplementary file 8 — HebbPlots of coding regions of inactive genes. This compressed file (.tar.gz) includes HebbPlots of genes inactive in 57 tissues/cell types. (TAR 2715 kb) [file 12859_2018_2312_MOESM8_ESM.tar › file9/E100.pdf]

Marks

H3K36me3

H3K4me3

H3K27ac

H3K4me1

H3K9me3

H3K27me3

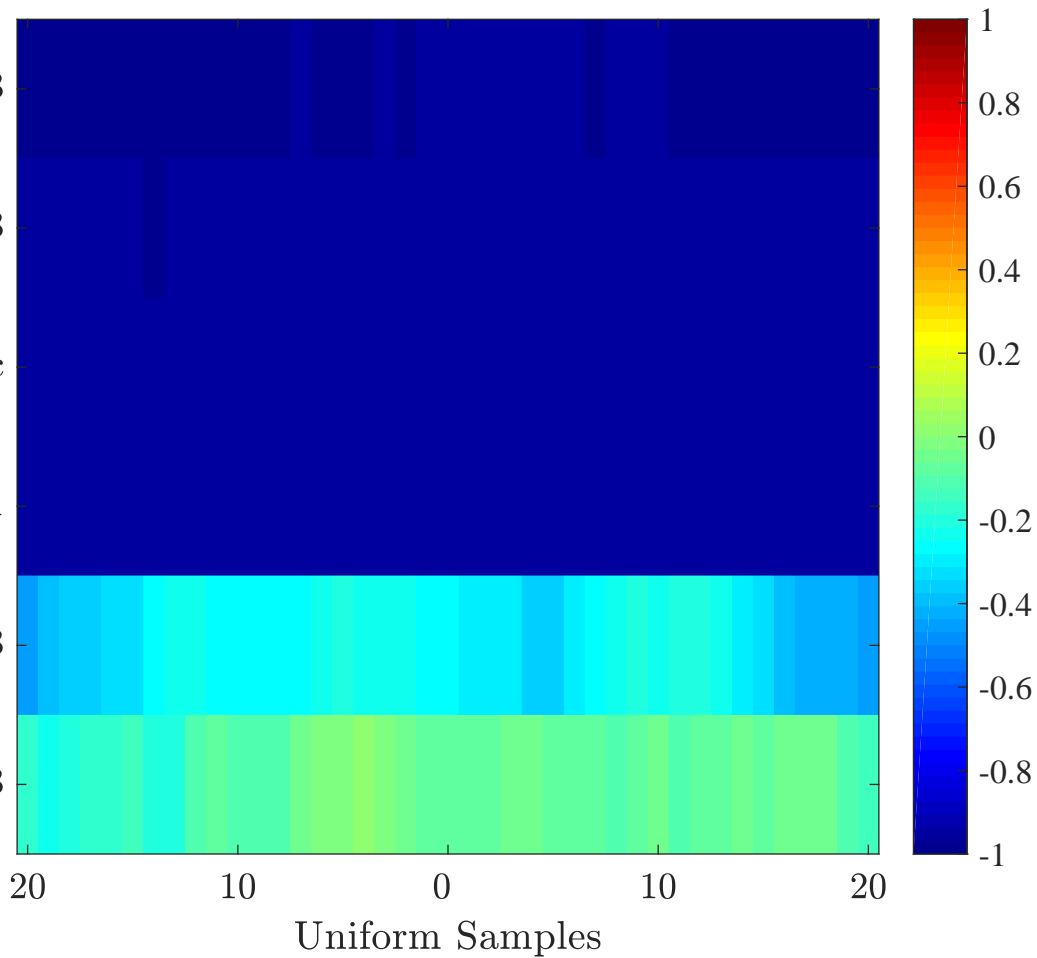

Supplement: Supplementary file 8 — HebbPlots of coding regions of inactive genes. This compressed file (.tar.gz) includes HebbPlots of genes inactive in 57 tissues/cell types. (TAR 2715 kb) [file 12859_2018_2312_MOESM8_ESM.tar › file9/E104.pdf]

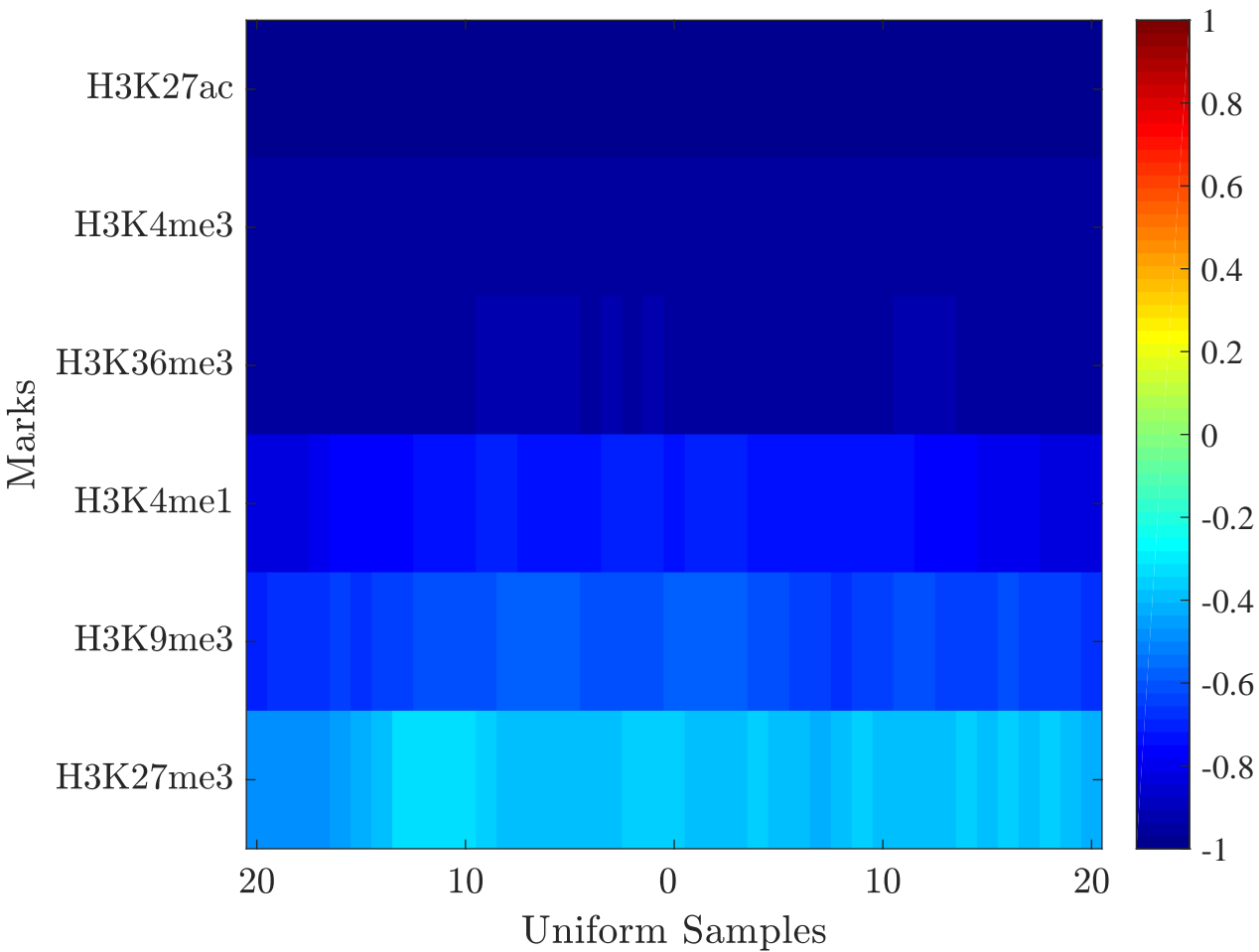

Supplement: Supplementary file 8 — HebbPlots of coding regions of inactive genes. This compressed file (.tar.gz) includes HebbPlots of genes inactive in 57 tissues/cell types. (TAR 2715 kb) [file 12859_2018_2312_MOESM8_ESM.tar › file9/E105.pdf]

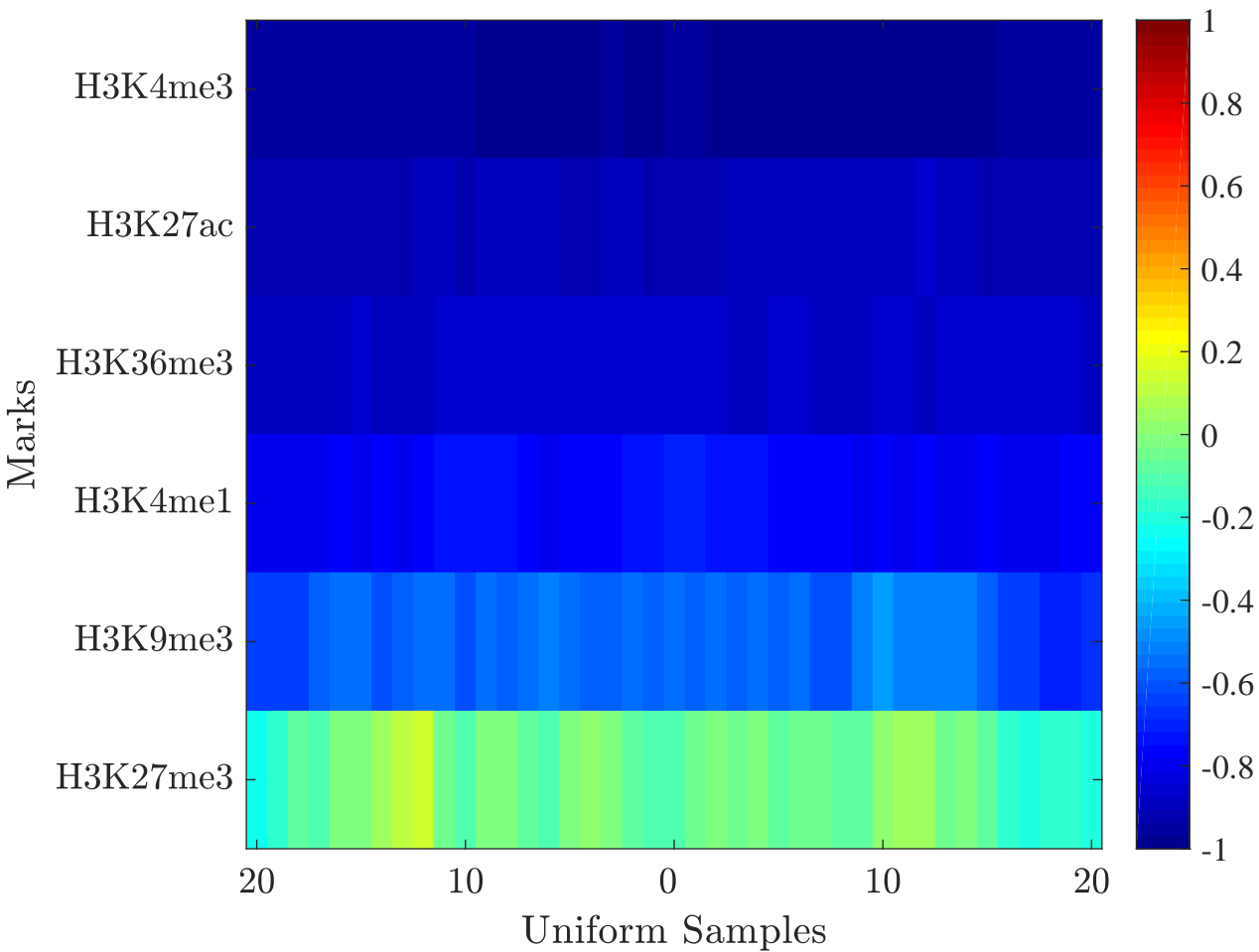

Supplement: Supplementary file 8 — HebbPlots of coding regions of inactive genes. This compressed file (.tar.gz) includes HebbPlots of genes inactive in 57 tissues/cell types. (TAR 2715 kb) [file 12859_2018_2312_MOESM8_ESM.tar › file9/E106.pdf]

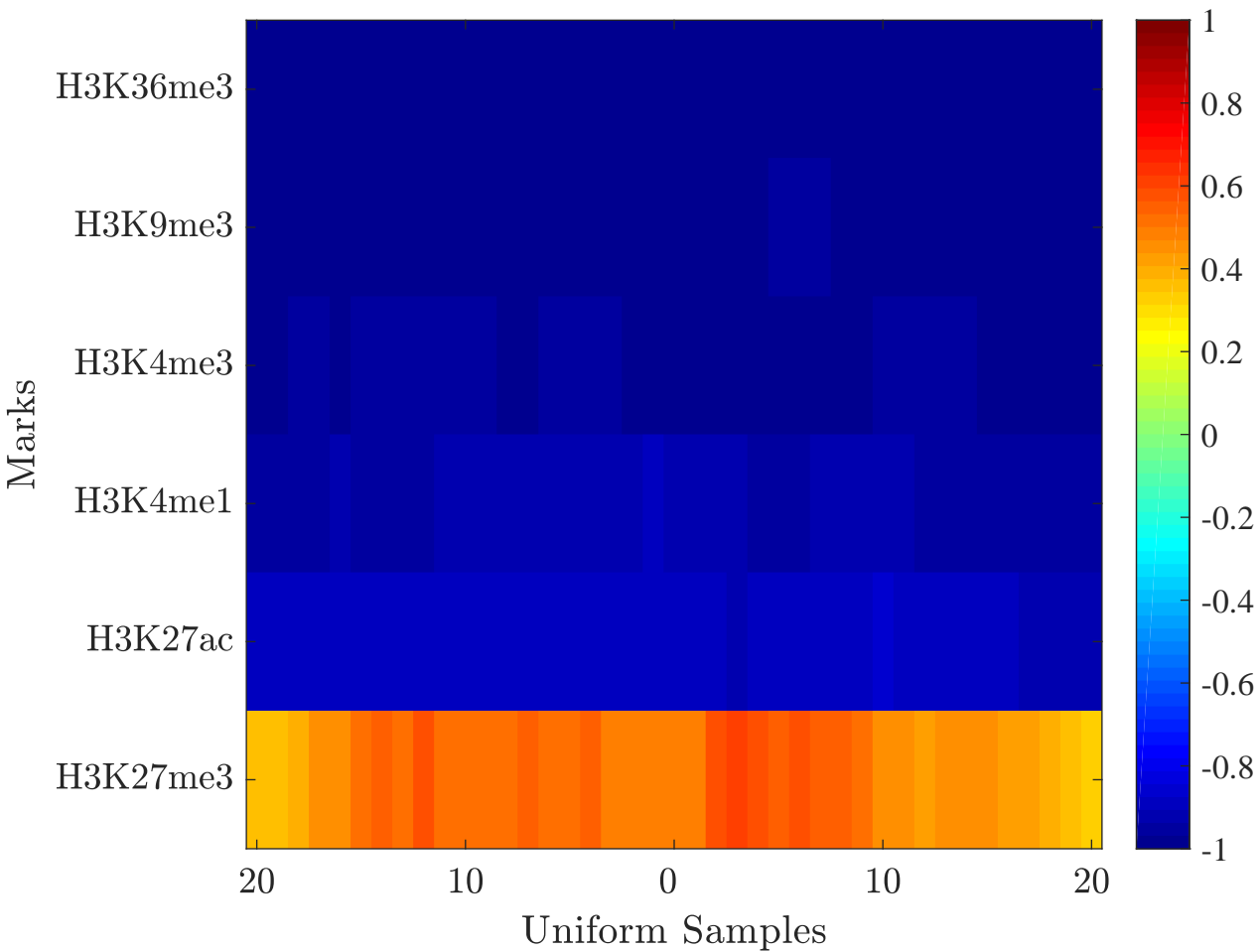

Supplement: Supplementary file 8 — HebbPlots of coding regions of inactive genes. This compressed file (.tar.gz) includes HebbPlots of genes inactive in 57 tissues/cell types. (TAR 2715 kb) [file 12859_2018_2312_MOESM8_ESM.tar › file9/E109.pdf]

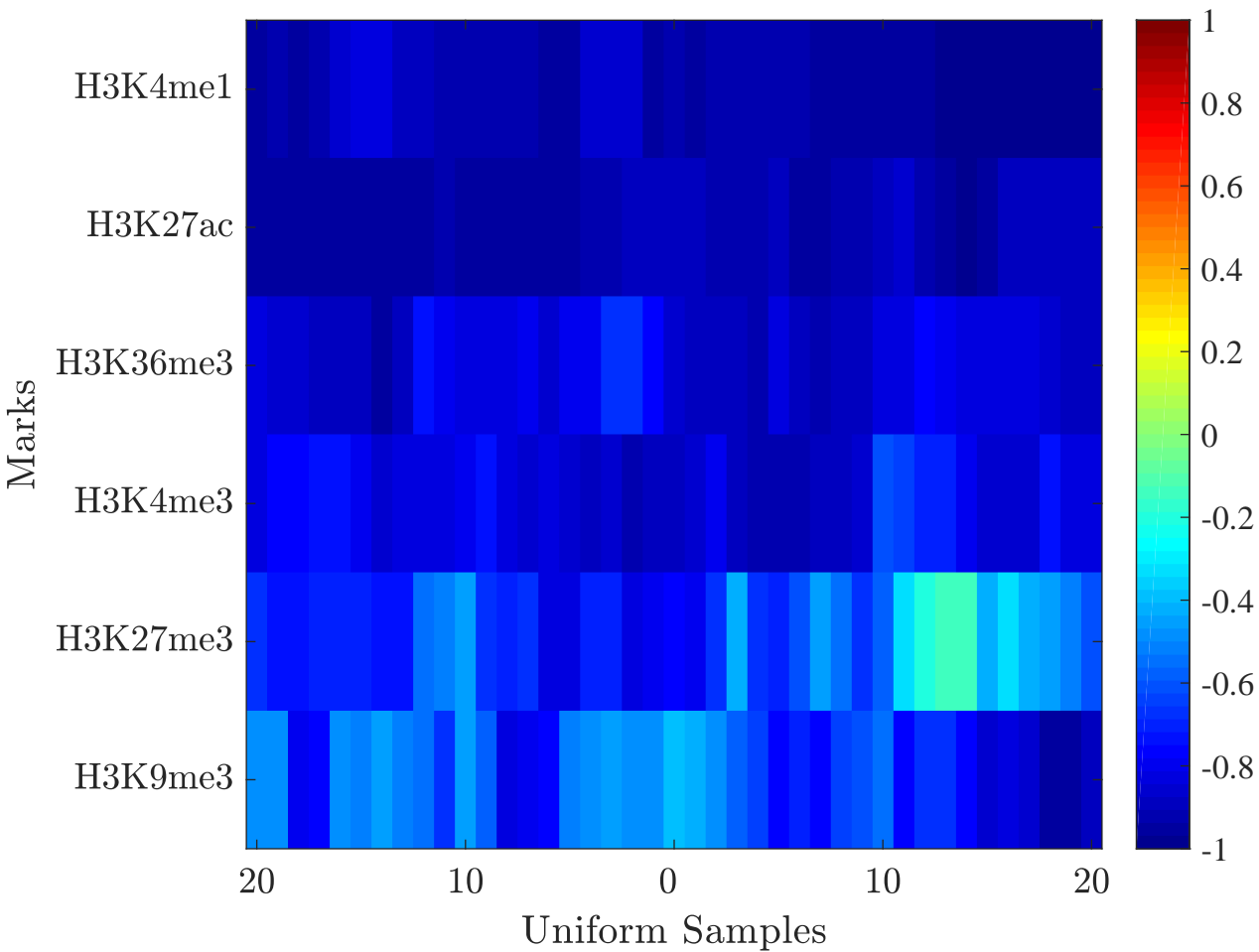

Supplement: Supplementary file 8 — HebbPlots of coding regions of inactive genes. This compressed file (.tar.gz) includes HebbPlots of genes inactive in 57 tissues/cell types. (TAR 2715 kb) [file 12859_2018_2312_MOESM8_ESM.tar › file9/E112.pdf]

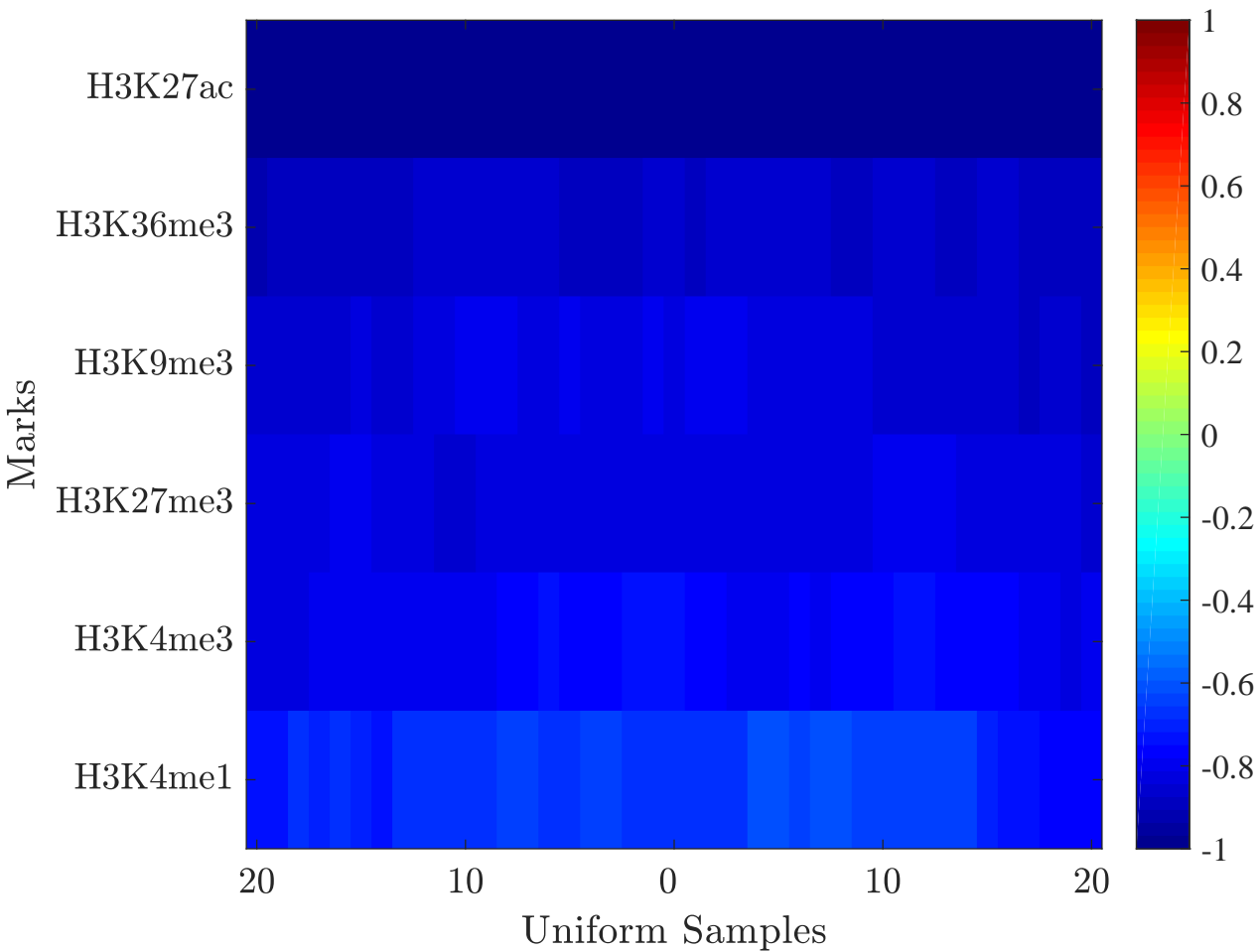

Supplement: Supplementary file 8 — HebbPlots of coding regions of inactive genes. This compressed file (.tar.gz) includes HebbPlots of genes inactive in 57 tissues/cell types. (TAR 2715 kb) [file 12859_2018_2312_MOESM8_ESM.tar › file9/E113.pdf]

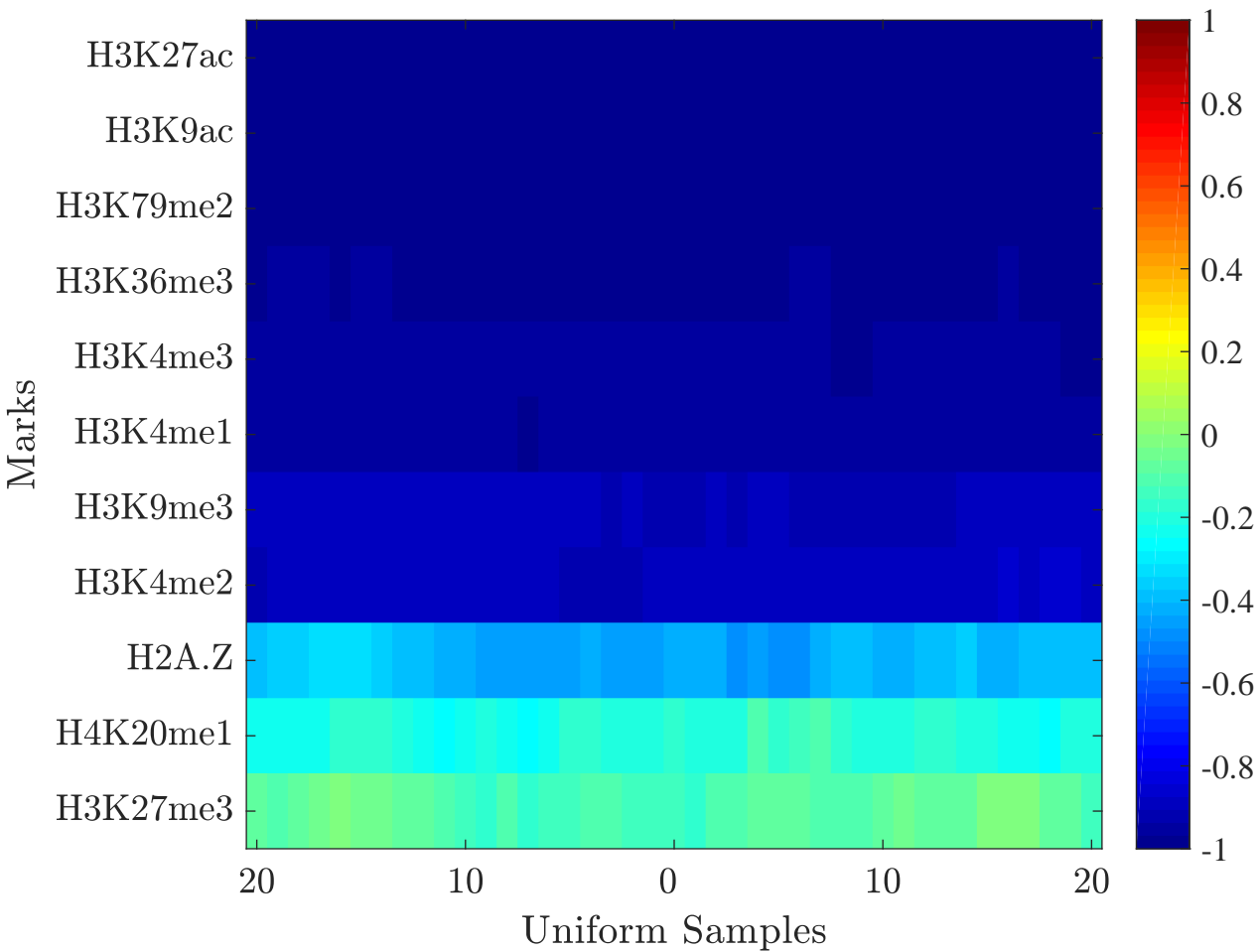

Supplement: Supplementary file 8 — HebbPlots of coding regions of inactive genes. This compressed file (.tar.gz) includes HebbPlots of genes inactive in 57 tissues/cell types. (TAR 2715 kb) [file 12859_2018_2312_MOESM8_ESM.tar › file9/E114.pdf]

Marks

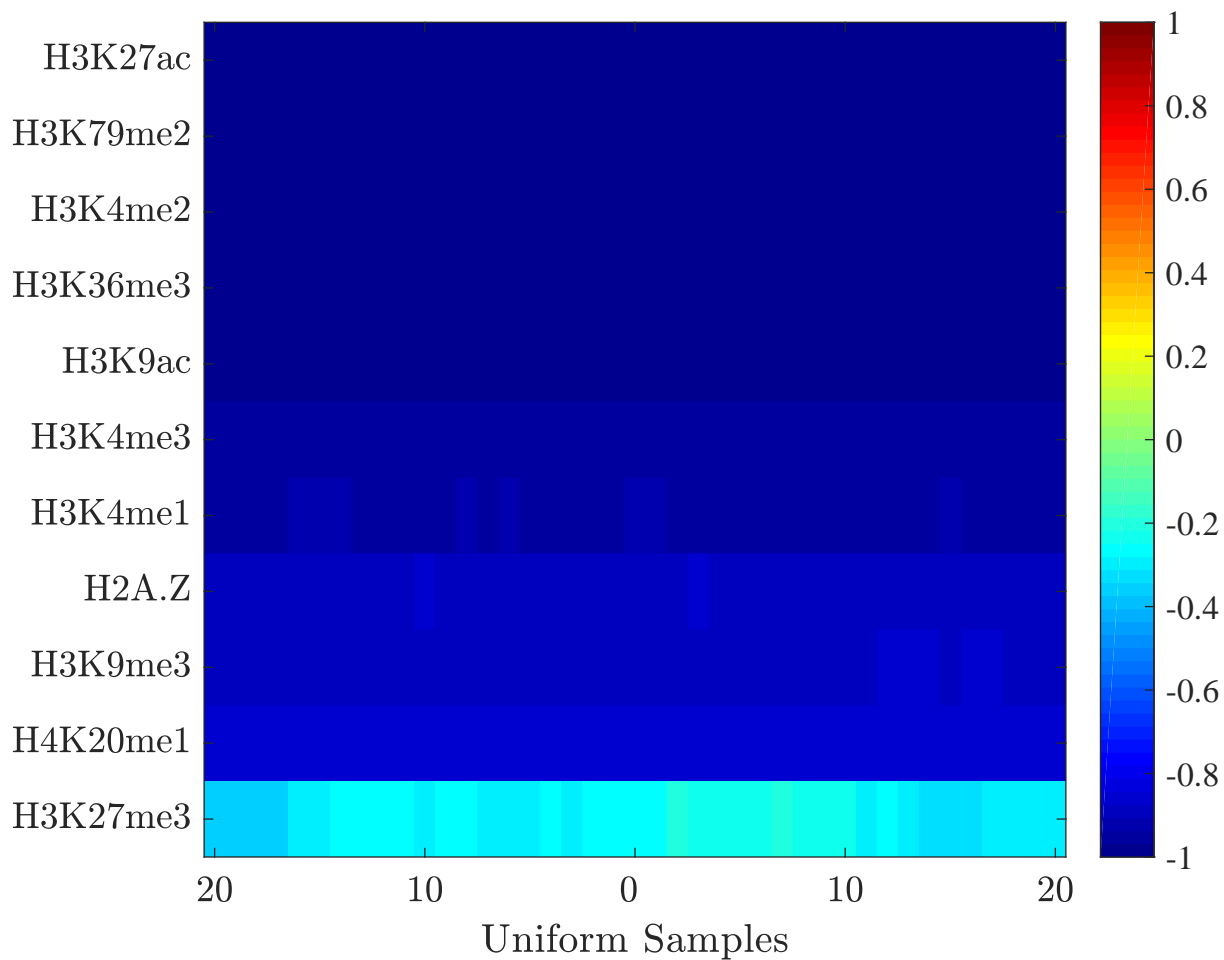

Supplement: Supplementary file 8 — HebbPlots of coding regions of inactive genes. This compressed file (.tar.gz) includes HebbPlots of genes inactive in 57 tissues/cell types. (TAR 2715 kb) [file 12859_2018_2312_MOESM8_ESM.tar › file9/E116.pdf]

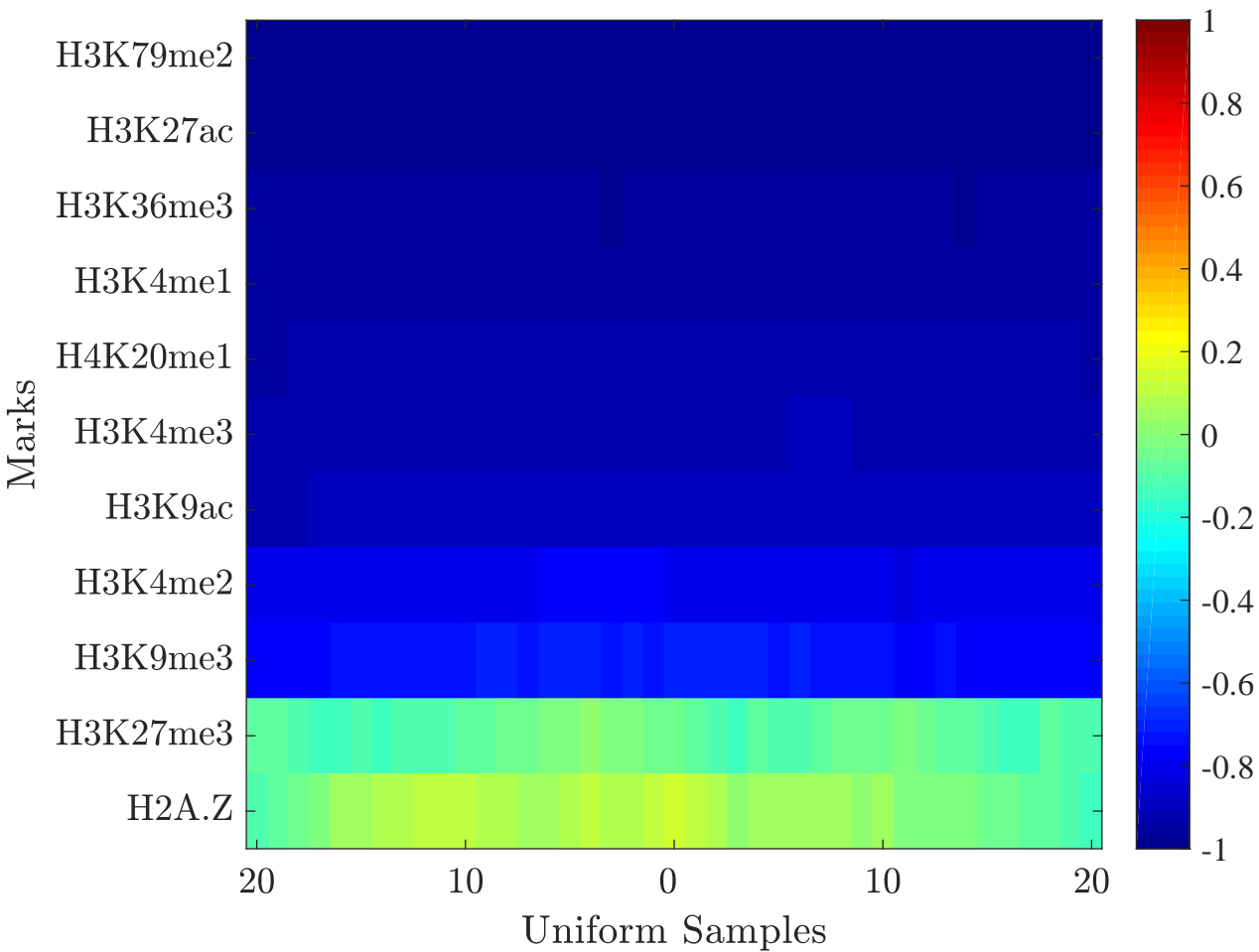

Supplement: Supplementary file 8 — HebbPlots of coding regions of inactive genes. This compressed file (.tar.gz) includes HebbPlots of genes inactive in 57 tissues/cell types. (TAR 2715 kb) [file 12859_2018_2312_MOESM8_ESM.tar › file9/E117.pdf]

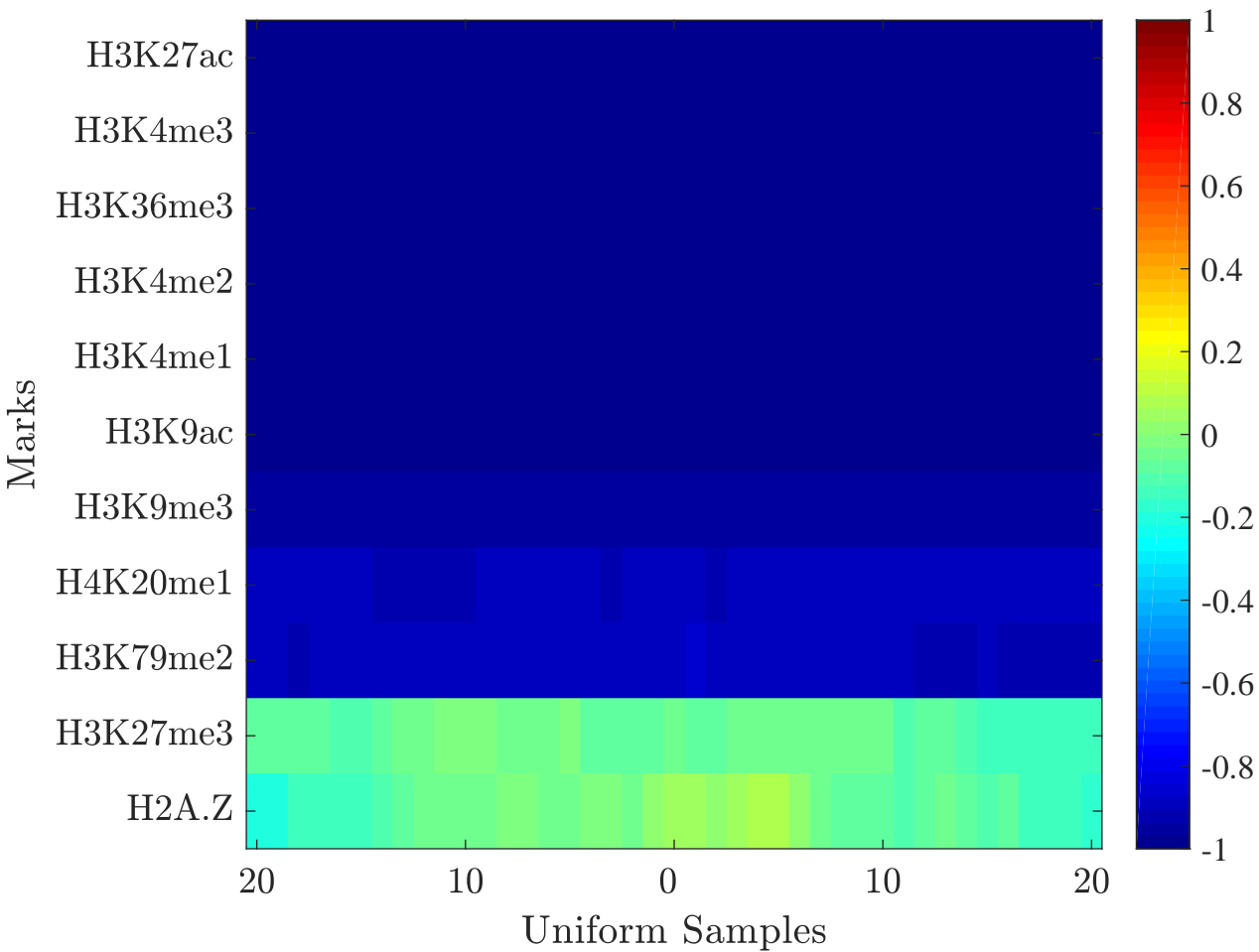

Supplement: Supplementary file 8 — HebbPlots of coding regions of inactive genes. This compressed file (.tar.gz) includes HebbPlots of genes inactive in 57 tissues/cell types. (TAR 2715 kb) [file 12859_2018_2312_MOESM8_ESM.tar › file9/E118.pdf]

Marks

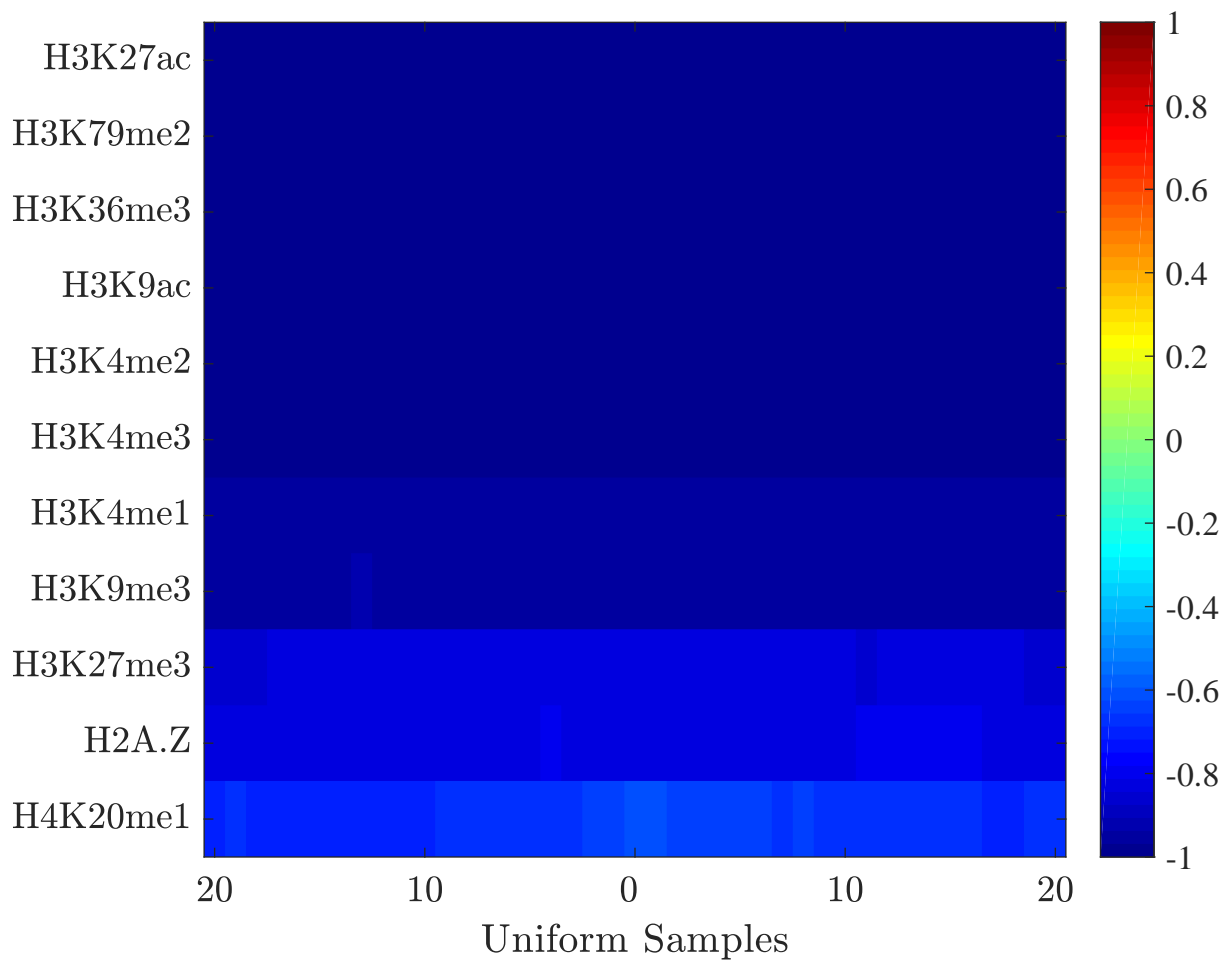

Supplement: Supplementary file 8 — HebbPlots of coding regions of inactive genes. This compressed file (.tar.gz) includes HebbPlots of genes inactive in 57 tissues/cell types. (TAR 2715 kb) [file 12859_2018_2312_MOESM8_ESM.tar › file9/E119.pdf]

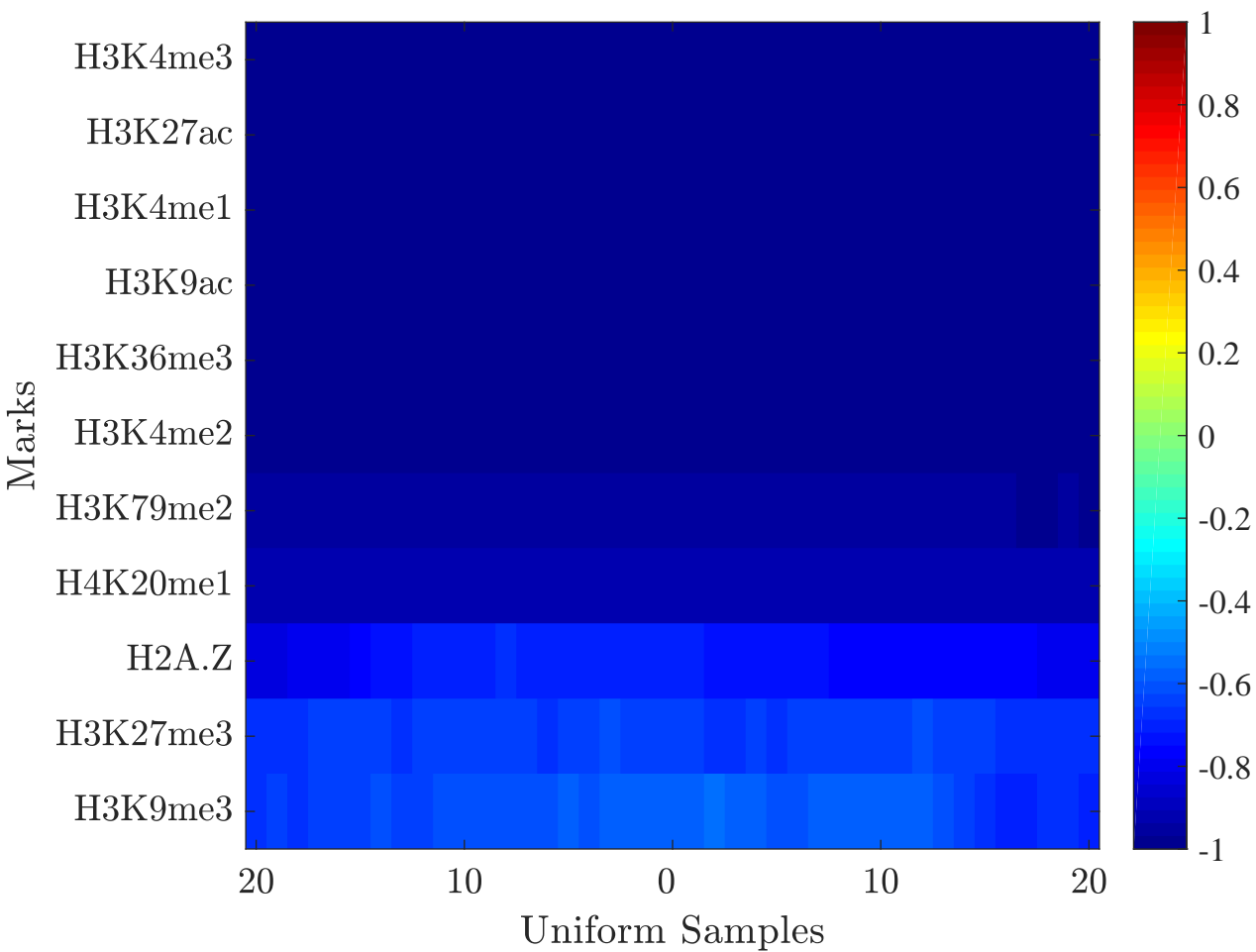

Supplement: Supplementary file 8 — HebbPlots of coding regions of inactive genes. This compressed file (.tar.gz) includes HebbPlots of genes inactive in 57 tissues/cell types. (TAR 2715 kb) [file 12859_2018_2312_MOESM8_ESM.tar › file9/E120.pdf]

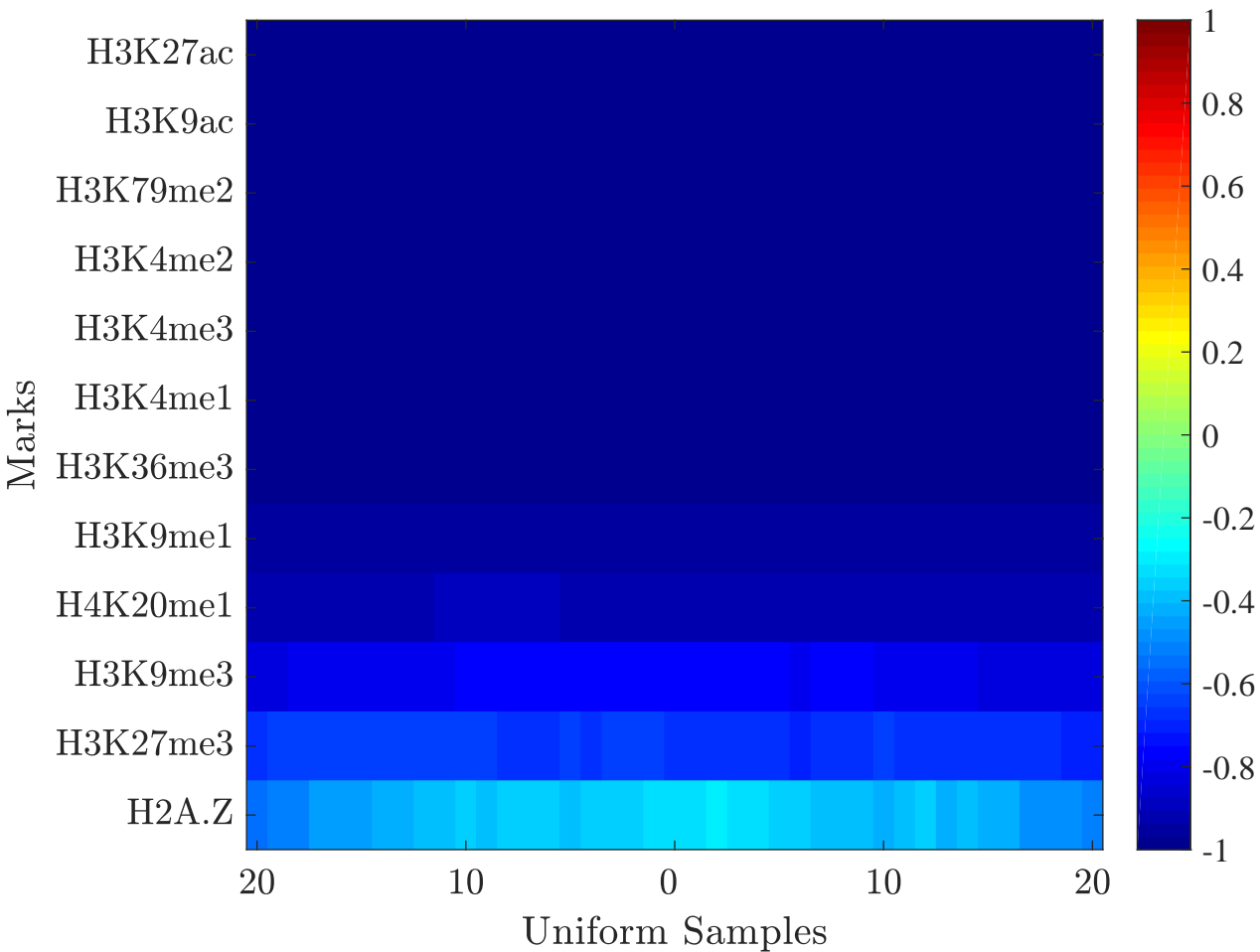

Supplement: Supplementary file 8 — HebbPlots of coding regions of inactive genes. This compressed file (.tar.gz) includes HebbPlots of genes inactive in 57 tissues/cell types. (TAR 2715 kb) [file 12859_2018_2312_MOESM8_ESM.tar › file9/E122.pdf]

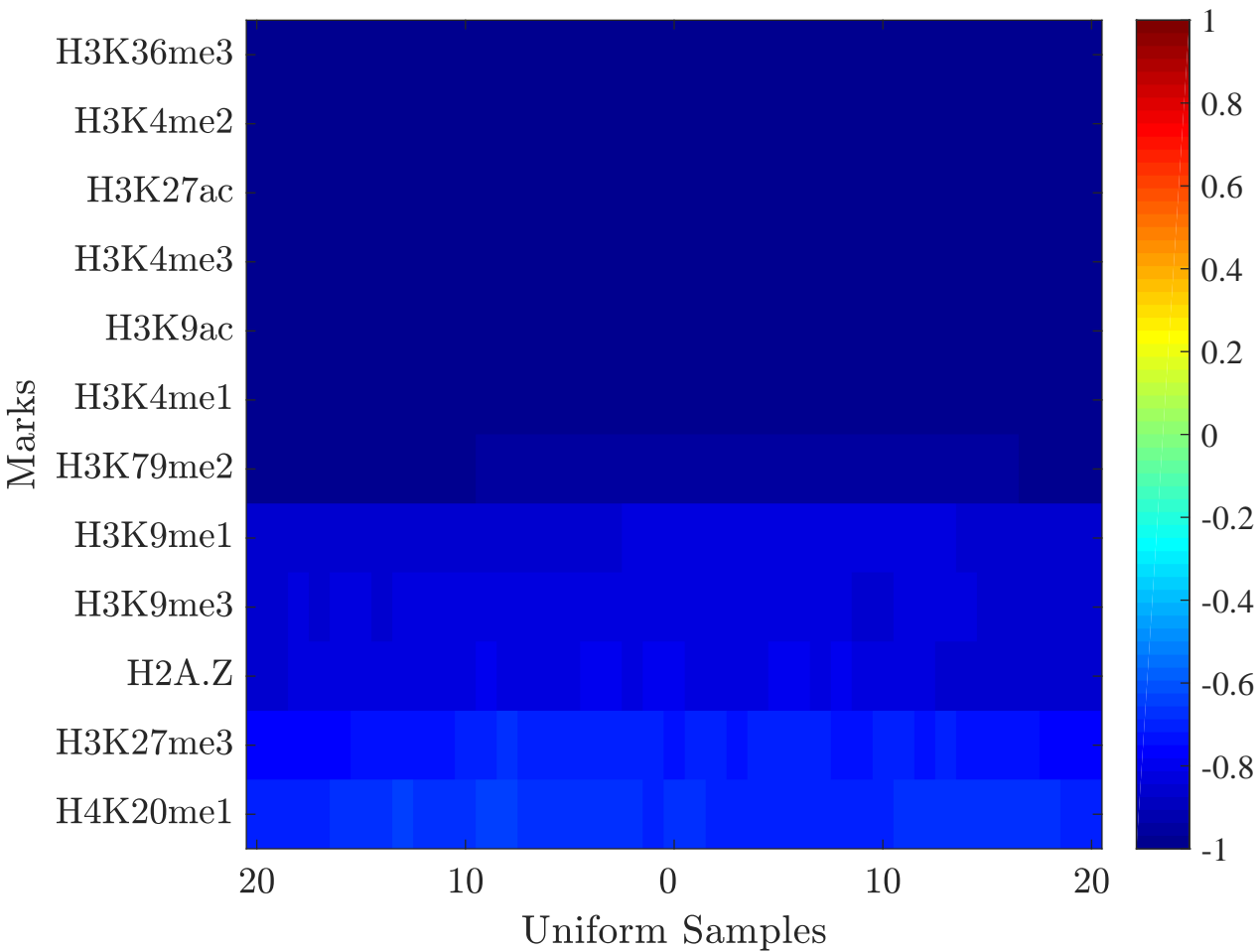

Supplement: Supplementary file 8 — HebbPlots of coding regions of inactive genes. This compressed file (.tar.gz) includes HebbPlots of genes inactive in 57 tissues/cell types. (TAR 2715 kb) [file 12859_2018_2312_MOESM8_ESM.tar › file9/E123.pdf]

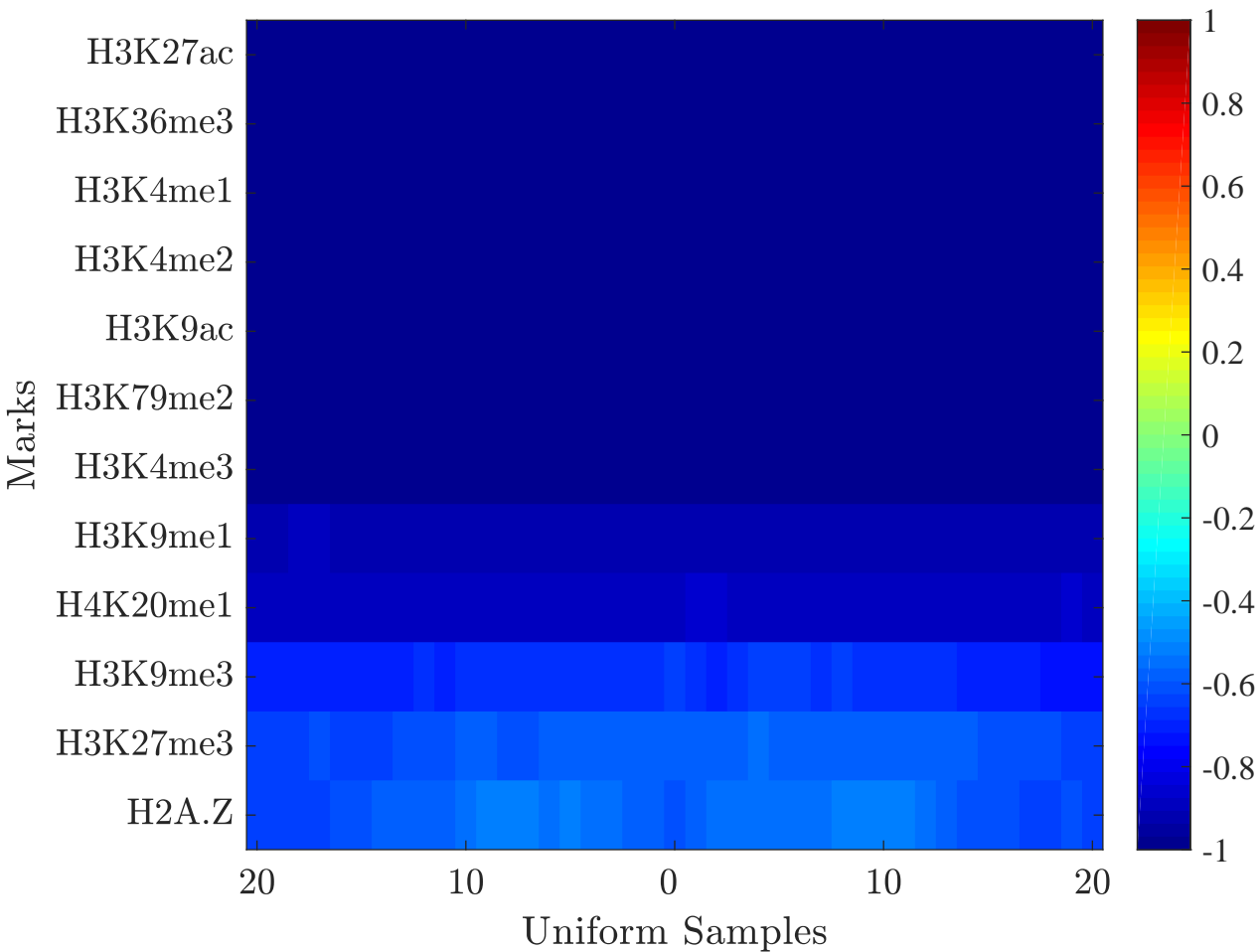

Supplement: Supplementary file 8 — HebbPlots of coding regions of inactive genes. This compressed file (.tar.gz) includes HebbPlots of genes inactive in 57 tissues/cell types. (TAR 2715 kb) [file 12859_2018_2312_MOESM8_ESM.tar › file9/E127.pdf]

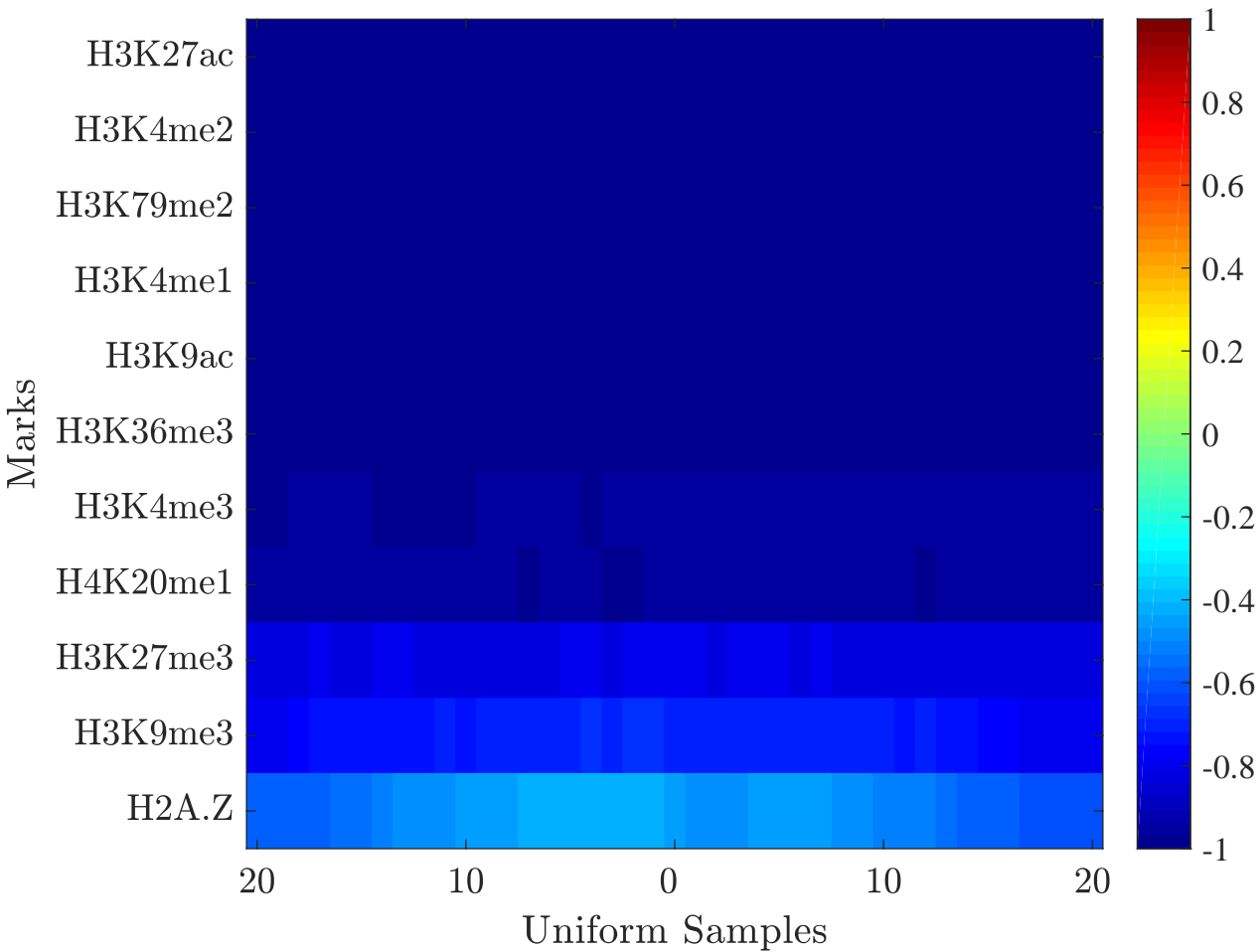

Supplement: Supplementary file 8 — HebbPlots of coding regions of inactive genes. This compressed file (.tar.gz) includes HebbPlots of genes inactive in 57 tissues/cell types. (TAR 2715 kb) [file 12859_2018_2312_MOESM8_ESM.tar › file9/E128.pdf]
