# Supplementary material for: HebbPlot: an intelligent tool for learning and visualizing chromatin mark signatures
Source: BMC Bioinformatics. 2018 Sep 3;19:310. doi: 10.1186/s12859-018-2312-1 (PMC6122555; doi:10.1186/s12859-018-2312-1)
Supplement: Supplementary file 10 — Figure S2. HebbPlots of promoters active in H1 cell line. These plots were generated from promoters with different sizes. Each HebbPlot was generated from a set of promoters, all of which have the same size and are centered on the transcription start sites. (PDF 5010 kb) [file 12859_2018_2312_MOESM10_ESM.pdf]

Supplementary Figure 2: HebbPlots of promoters active in H1 cell line. These plots were generated from promoters with different sizes. Each HebbPlot was generated from a set of promoters, all of which have the same size and are centered on the transcription start sites.

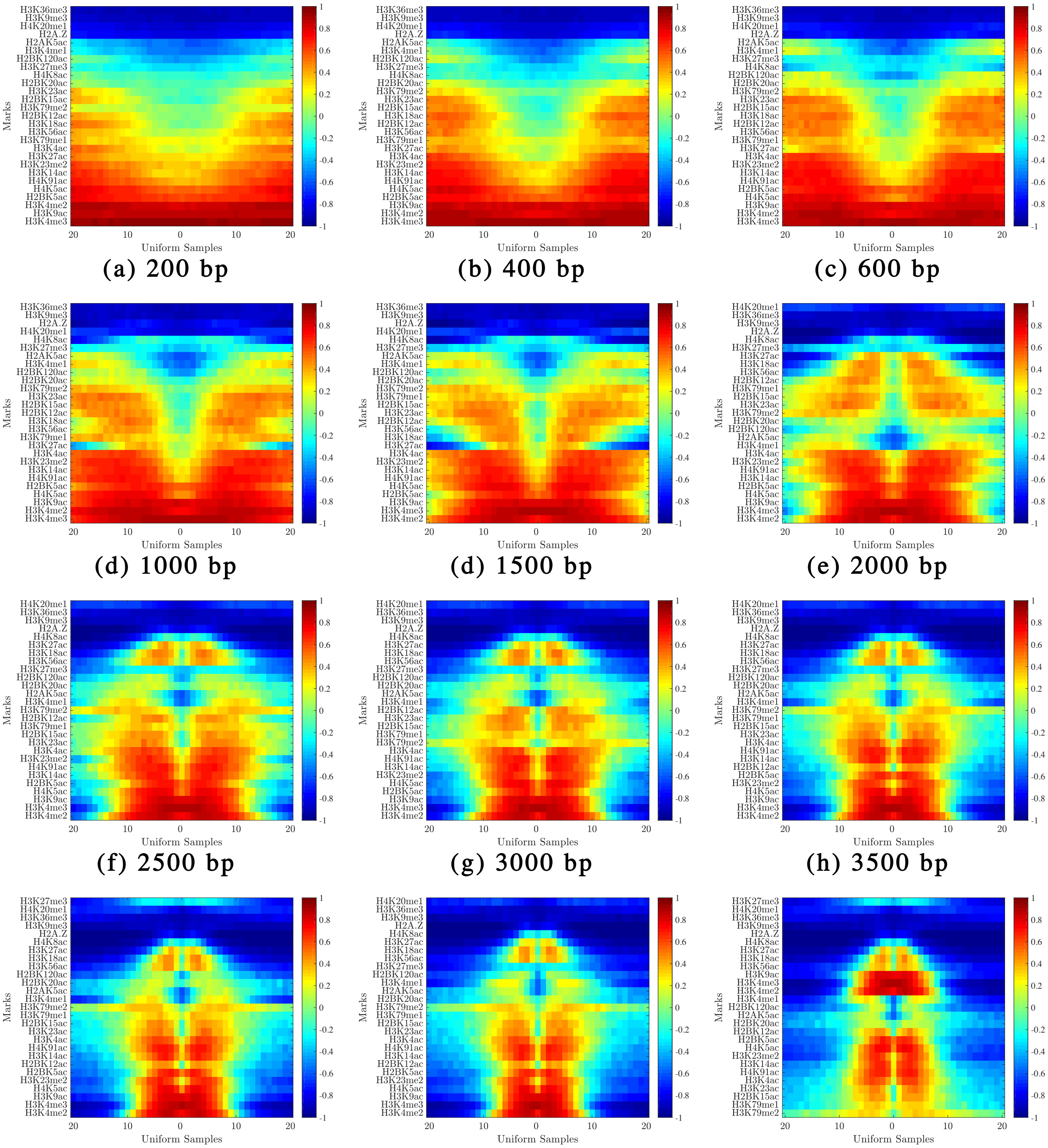

(a) 200 bp (b) 400 bp (c) 600 bp

(d) 1000 bp (d) 1500 bp (e) 2000 bp

(f) 2500 bp (g) 3000 bp (h) 3500 bp

(j) 4000 bp (k) 4500 bp (l) 5000 bp
